# Albiflorenes A–L, polyoxygenated cyclohex(a/e)ne diterpene esters from *Kaempferia albiflora*

**DOI:** 10.1038/s41598-024-64889-6

**Published:** 2024-06-17

**Authors:** Pornpuk Booranaseensuntorn, Jutatip Boonsombat, Sanit Thongnest, Paratchata Batsomboon, Nanthawan Reuk-Ngam, Panita Khlaychan, Saroj Ruchisansakun, Prasat Kittakoop, Supanna Techasakul, Chulabhorn Mahidol, Somsak Ruchirawat

**Affiliations:** 1 Program in Chemical Sciences, Chulabhorn Graduate Institute, Bangkok, Thailand; 2 Chulabhorn Research Institute, Bangkok, Thailand; 3 Center of Excellence on Environmental Health and Toxicology (EHT), OPS, MHESI, Bangkok, Thailand; 4 Department of Plant Science, Faculty of Science, Mahidol University, Bangkok, Thailand

**Keywords:** Chemical biology, Microbiology, Plant sciences, Environmental sciences, Chemistry

## Abstract

Twelve polyoxygenated cyclohex(a/e)ne diterpene esters, named albiflorenes A–L (**1**–**12**), were isolated from the whole plants of *Kaempferia albiflora*, known as “Prao Mang Mum.” Their structures and relative stereochemistry were determined by extensive spectroscopic analysis. Furthermore, the comparison of experimental electronic circular dichroism (ECD) curves with the curves predicted by TDDFT was used to determine the absolute configurations. Albiflorenes contain polyoxygenated cyclohexane (or cyclohexene) derivatives, which are linked to either isopimarane or abietane diterpene acid units. The discovery marks the first occurrence of a conjugate between polyoxygenated cyclohexane (or cyclohexene) rings and diterpenoids. Among the isolates, albiflorene C specifically exhibited antibacterial activity against *Bacillus cereus* with MIC and MBC values of 3.13 and 6.25 μg/mL, respectively.

## Introduction

The genus *Kaempferia*, which is part of the Zingiberaceae family, encompasses around 60 species globally. This genus is predominantly found in Southeast Asia, with a notable concentration in Thailand^1,2^. Several species of this genus hold economic value for their medicinal or culinary purposes, for example, *K. parviflora, which* has gained significant interest in the last twenty years as a supplement for enhancing vitality; and *K. galanga*, which is known for its rhizome that serves as a flavor spice of various cooking. In recent years, there has been growing interest in *Kaempferia* plants due to their diverse structural variations with interesting biological properties^3^. Several studies on this genus have led to the discovery of flavonoids^4^, diterpenoids^5–7^, and polyoxygenated cyclohexenes^8,9^, many of which possess antimalarial^4,5^, antimicrobial^5,10^, anti-inflammatory^11–14^, and cytotoxic properties^15^.

The *Kaempferia* genus can be categorized into two subgenera^16^. One is the *Kaempferia* subgen. *Kaempferia*^17^ which generates inflorescence on the pseudostems, while the other is the *Kaempferia* subgen. *Protanthium* (Horan.) Baker^18^ which produces inflorescence directly from the rhizome before emerging of the leafy shoot. The *Kaempferia* subgen. *Protanthium* denotes a minority group, and currently only about 15 species have been acknowledged^18^. To date, there has been no documented research on the chemical profiles of this subgenus.

**Figure 1:**
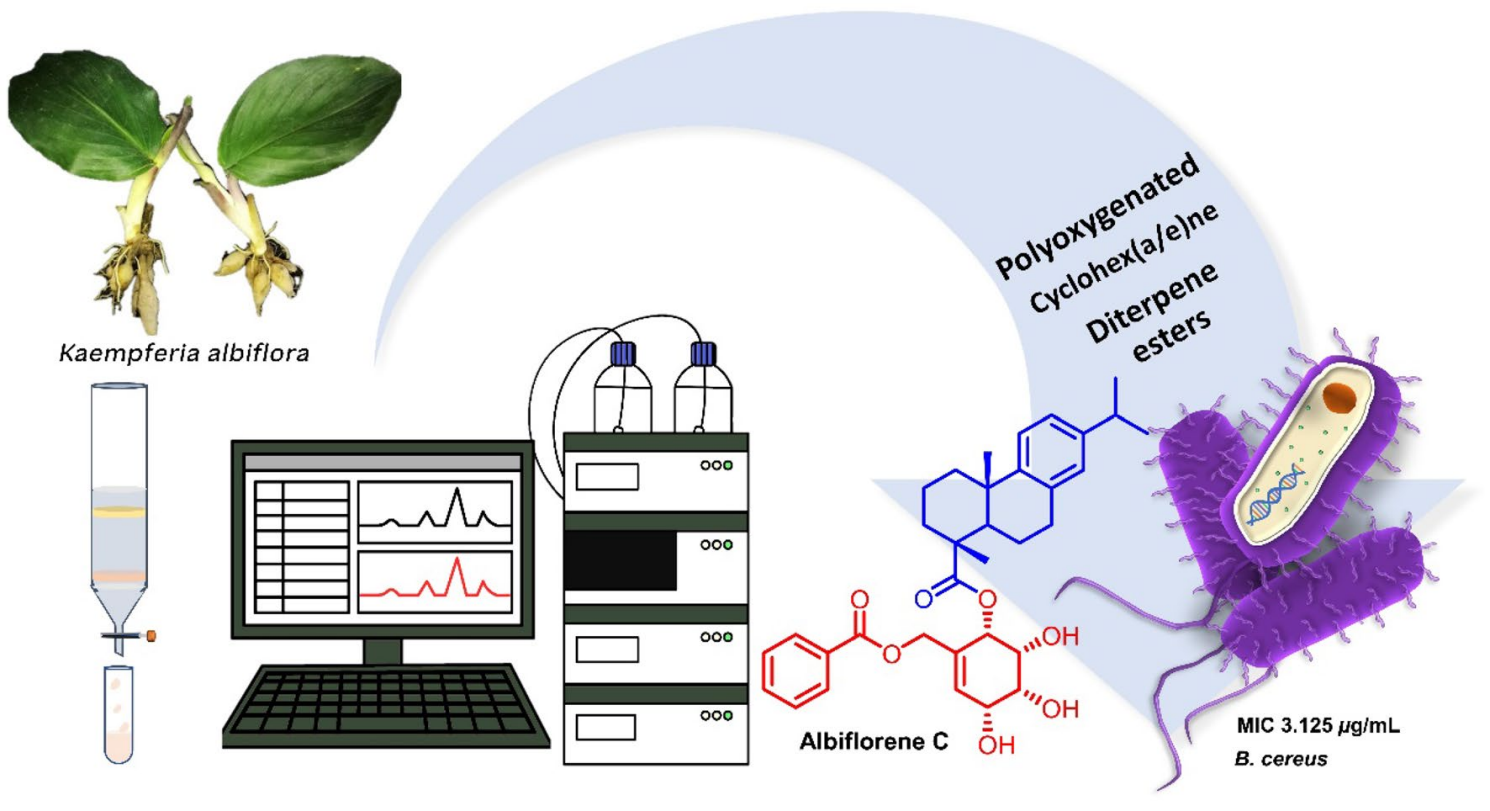
The morphological characteristics of *K. albiflora* and the interesting structure named albiflorene C exhibit notable antibacterial activity against *B. cereus*.

With the significance of discovering novel plant resources that could potentially be employed in the development of medicines, our research group has a sustained interest in exploring bioactive compounds derived from plants within the *Kaempferia* genus. Recently, we have investigated *K. albiflora* (syn. *K. uttaraditensis*)^19^, a plant belonging to the subgenus *Protanthium* of the *Kaempferia* genus, which is endemic to Thailand. This study provided a detailed structural elucidation of twelve unprecedented compounds (**1**–**12**), along with nine previously reported compounds (**13**–**21**), isolated from the combined CH_2_Cl_2_–MeOH (1:1) extract and the EtOAc extract of the whole plants of *K. albiflora.* Bioassay probing the potential antimicrobial activity of the isolates was performed, and the results showed that albiflorene C exhibited good antibacterial activity against *Bacillus cereus* (Fig. 1)*.*

## Results and discussion

By employing successive column chromatography on silica gel, along with gel filtration and reversed-phase HPLC, a total of twelve unprecedented polyoxygenated cyclohex(a/e)ne diterpene esters were effectively isolated, together with nine recognized compounds, from the whole *K. albiflora* plants*.*

**Figure 2:**
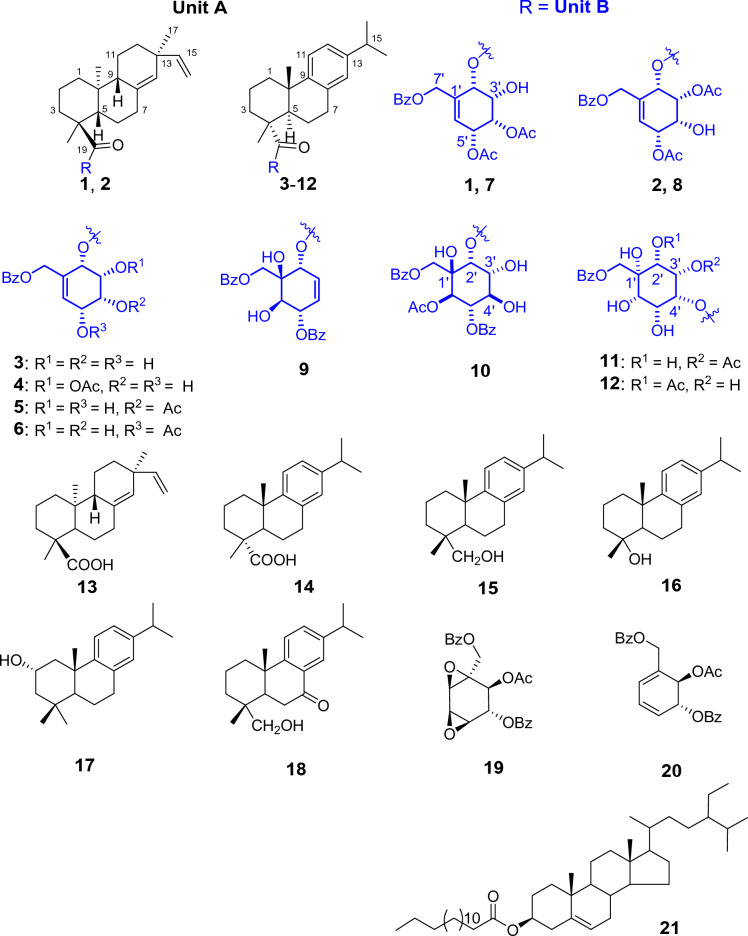
Structural compounds (**1**–**21**) derived from *K. albiflora* through isolation.

Albiflorene A (**1**) (Fig. 2), a yellow powder, had a molecular formula of C_38_H_48_O_9_ inferred from the HRESIMS [M+Na]^+^ ion peak at *m/z* 671.3195 (calcd 671.3191), and supported by ^13^C and DEPT135 NMR data, implying fifteen unsaturation indices. UV absorption bands *λ*_max_ at 229 and 203 nm indicated a characteristic of an aromatic system, and the IR spectrum showed the *ν*_max_ for a hydroxy (3432 cm^–1^) group and a conjugated carbonyl (1722 cm^–1^) functionality. Extensive NMR data were used to clarify the planar structure of **1**, which unveiled its composition comprising two subunits designated as unit A and unit B. In unit A, the ^1^H-NMR (Table 1) showed signals for three methyl groups at *δ*_H_ 1.03 (s, H_3_-17), 1.21 (s, H_3_-18), and 0.82 (s, H_3_-20), a tri-substituted olefin at *δ*_H_ 5.20 (br s, H-14), and a vinyl group with the *δ*_H_ value of 5.75 (dd,* J* = 17.5, 10.6 Hz), 4.89 (dd, *J* = 17.5, 1.4 Hz), and 4.87 (dd, *J* = 10.6, 1.4 Hz). The ^13^C NMR (Table 1), combined with HSQC and DEPT spectra revealed 20 carbon resonances consisting of three methyls, eight methylenes including one alkene (*δ*_C_ 110.3), four methines including two olefinic (*δ*_C_, 129.3, 149.1), and five quaternary carbons including one olefinic (*δ*_C_ 136.7) and one carbonyl (*δ*_C_ 178.8). The above spectroscopic data aligned with a pimarane diterpenoid skeleton. In addition, the ^1^H- and ^13^C-NMR data for unit A closely resembled those of isopimaric acid^20^, implying that the isopimaric acid served as unit A and constituted part of compound **1**. The HMBC correlations confirmed the planar structure of the isopimaric acid portion, and this was substantiated by NOESY correlations of H-5/H-9 and H_3_-18/H_3_-20/H_3_-17 (Fig. S7). The isopimaric acid structure in nature exists in two forms: the normal form, known as sandaracopimaric acid^20^, and the *ent* form, referred to as *ent*-isopimar-8(14),15-dien-19-oic acid (also known as *ent*-isopimaric acid)^21^. These two compounds exhibited identical NMR data and very similar specific optical rotation values (${\left[\alpha \right]}_{\text{D}}^{26}$ –17.7 for sandaracopimaric acid and ${\left[\alpha \right]}_{\text{D}}^{25}$ –12.5 for *ent*-isopimaric acid), making it impossible to determine the absolute configuration using optical rotation alone. Due to the absence of circular dichroism (CD) information, we resorted to employ the electronic circular dichroism (ECD) method to determine the absolute configuration of isopimaric acid structure in unit A. In our study, we fortunately isolated the diterpenoid isopimaric acid **13** (Fig. 2). Following biogenesis reasoning, this compound was anticipated to act as a precursor for polyoxygenated cyclohexene isopimaryl esters found in this plant. Therefore, determining the absolute configuration of **13** could contribute to defining the unit A segment in compounds **1** and **2**. Through a comparison of the calculated ECD data with the experimental results (Fig. S17), it was determined that **13** shared the absolute configuration with *ent*-isopimaric acid (4*S*,5*S*,9*R*,10*S*,13*S*), as indicated by the strong qualitative agreement between their ECD spectra. This finding confirmed that both unit A of **1** and the following unit A of **2** are *ent*-isopimaric acid.

**Table 1:** ^1^H and ^13^C NMR data of compounds **1** and** 2** in CDCl_3_.

No	**1**		**2**	
	*δ*_H_	*δ*_C_	*δ*_H_	*δ*_C_
1	1.73, m 1.11, m	38.4	1.75, br d (12.8) 1.13, m	38.3
2	1.53, m	18.3	1.56, m	18.3
3	1.71, m 1.62, m	37.1	1.70, m 1.60, m	37.1
4	–	48.0	–	47.9
5	1.93, dd (12.5. 2.4)	49.0	1.92, dd (12.5. 2.4)	49.1
6	1.40, m 1.15, m	25.1	1.43, m 1.08, m	25.1
7	2.16, m 2.05, m	35.5	2.15, m 1.99, m	35.5
8	–	136.7	–	136.5
9	1.81, t (7.4)	50.7	1.81, br t (7.5)	50.7
10	–	37.9	–	37.9
11	1.59, m 1.50, m	18.7	1.63, m 1.52, m	18.7
12	1.44, m 1.35, m	34.6	1.47, m 1.38, td (12.3, 3.6)	34.5
13	–	37.5	–	37.5
14	5.20, s	129.3	5.21, br s	129.5
15	5.75, dd (17.5, 10.6)	149.1	5.77, dd (17.4, 10.6)	149.0
16	4.89, dd (17.5, 1.4) 4.87, dd (10.6, 1.4)	110.3	4.92, dd (17.4, 1.4) 4.90, dd (10.6, 1.4)	110.3
17	1.03, s	26.2	1.05, s	26.2
18	1.21, s	17.2	1.21, s	17.2
19	–	178.8	–	177.6
20	0.82, s	15.4	0.84, s	15.4
1ʹ	–	134.4	–	134.6
2ʹ	5.58^a^, br d (5.2)	70.4	5.80, br d (5.4)	64.0
3ʹ	4.13, td (5.2, 2.5)	69.5	5.25, dd (5.4, 2.5)	72.5
4ʹ	5.16, dd (6.0, 2.5)	72.3	4.19, ddd (5.4, 5.0, 2.5)	69.4
5ʹ	5.58^a^, br d (5.2)	68.1	5.46, br t (5.0)	70.9
6ʹ	5.99, br s	125.7	6.00, d (2.6)	125.9
7ʹ	4.81, br s	64.1	4.82, d (13.5) 4.77, d (14.3)	64.0
1ʹʹ	–	129.7	–	129.6
2ʹʹ	8.03, dd (8.2, 1.1)	129.9	8.03, m	129.9
3ʹʹ	7.47, t (7.6)	128.6	7.47, m	128.6
4ʹʹ	7.53, t (7.5)	133.5	7.53, tt (7.5, 1.3)	133.5
5ʹʹ	7.47, t (7.6)	128.9	7.47, m	128.6
6ʹʹ	8.03, dd (8.2, 1.1)	129.9	8.03, m	129.9
7ʹʹ	–	166.1	–	166.0
OCOCH_3_	2.12^b^, s	21.13^c^	2.12^a^, s	21.3^e^
OCOCH_3_	2.14^b^, s	21.11^c^	2.14^a^, s	21.1^e^
OCOCH_3_	–	170.4^d^	–	171.0^f^
OCOCH_3_	–	170.2^d^	–	170.4^f^

^a,b,c,d,e,f^Overlap and Interchangeable values in the same column.

**Figure 3:**
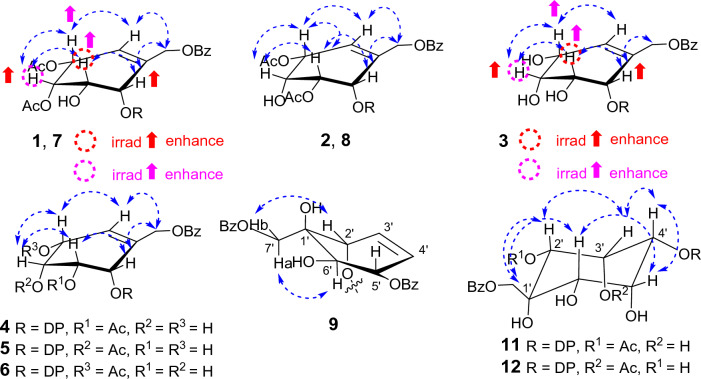
Selected NOESY correlations for polyoxygenated cyclohex(a/e)ne rings of **1**–**9**, **11**, and **12**.

In unit B, signals corresponding to four oxymethines were observed at the following chemical shifts: *δ*_H_ 5.58 (d, *J* = 5.2 Hz, H-2ʹ), 4.13 (td, *J* = 5.2, 2.5 Hz, H-3ʹ), 5.16 (dd, *J* = 6.0, 2.5 Hz, H-4ʹ), and 5.58 (d, *J* = 5.2 Hz, H-5ʹ). Furthermore, an oxymethylene signal appeared at *δ*_H_ 4.81 (H-7ʹ), and an olefinic methine signal was observed at *δ*_H_ 5.99 (H-6ʹ). Two acetyl groups were also identified by signals at *δ*_H_ 2.12 and 2.14. The presence of one aromatic group was also evident from the ^1^H NMR signals in the range of *δ*_H_ 7.47–8.03 (5H) (Table 1). These data suggested the polyoxygenated cyclohexene moiety with aromatic and acetoxy substituents. The occurrence of a polyoxygenated cyclohexene moiety was confirmed through cross peaks in the HMBC correlations from OH-3ʹ to C-2ʹ and C-4ʹ; from H-2ʹ/H-5ʹ to C-3ʹ, C-4ʹ, and C-7ʹ; from H-3′ to C-1′; H-4′ to C-2ʹ, C-5′, and CO; from H-6ʹ to C-4ʹ, C-5ʹ, and C-7ʹ; and from H-7′ to C-1′, C-6′, and CO-7ʹ′. Lastly, the connection of two structural parts was identified as an ester bond at C-19, evidenced by the HMBC cross peak between H-2ʹ (unit B) and C-19 (unit A). The relative configuration of polyoxygenated cyclohexene moiety was identified through an analysis of the coupling constant data and the NOESY correlations (Fig. S7). The small coupling constant value of *J*_H-5ʹ/H-6ʹ_, which appeared as a broad singlet, indicated pseudo-axial orientation of H-5ʹ. This orientation permits a dihedral angle of approximately 90 degrees^22^ between the allylic proton H-5ʹ and the adjacent olefinic proton H-6ʹ within the preferred conformation of cyclohexene ring as shown in Fig. 3. Furthermore, The NOESY correlations observed between H-2ʹ/H-3ʹ, H-3ʹ/H-4ʹ, and H-4ʹ/H-5ʹ (Fig. 3), combined with the coupling constants of *J*_H-2ʹ/H-3ʹ_ = 5.2 Hz, *J*_H-3ʹ/H-4ʹ_ = 2.5 Hz (Table 1), indicated a *cis* relationship among them. The *J*_H-4ʹ/H-5ʹ_ coupling constants exhibited some differences (6.0 and 5.2 Hz, respectively), but these values still implied a *cis* relationship. Also, the COSY analysis clearly demonstrated the correlation between these relevant signals. The above observation suggested that H-2ʹ, H-3ʹ, H-4ʹ, and H-5ʹ were axially oriented on the same side. The similar multiplicities and coupling constants in the ^1^H NMR spectra of **1**’s cyclohexene ring (H-2ʹ to H-6ʹ) to those of conduritol D (cyclohex-5-ene-1,2,3,4-tetraol)^23^, which exclusively adopted an all-*cis* stereochemistry, strongly suggested a shared relative configuration between unit B of **1** and conduritol D.

The remaining orientation to be determined is the arrangement of the ester bond linking C-19 to C-2ʹ of the cyclohexene moiety. Since the cyclohexene moiety (unit B) could attach to *ent*-isopimaric acid (unit A) in two possible orientations, this resulted in two potential absolute configurations for compound **1** as (4*S*,5*S*,9*R*,10*S*,13*S*,2ʹ*S*,3ʹ*S*,4ʹ*S*,5ʹ*R*) and (4*S*,5*S*,9*R*,10*S*,13*S*,2ʹ*R*,3ʹ*R*,4ʹ*R*,5ʹ*S*). However, due to its conformational flexibility of the ester linkage, NOESY correlations could not be used to establish their relationship. Furthermore, a biphasic pattern observed in the ECD spectrum of **1** made it unfeasible to ascertain the absolute configuration of unit B by comparing with calculated spectrum. As a consequence, on the basis of the aforementioned data, the absolute configuration of **1** could be identified as (4*S*,5*S*,9*R*,10*S*,13*S*,2ʹS*,3ʹS*,4ʹS*,5ʹR*).

**Figure 4:**
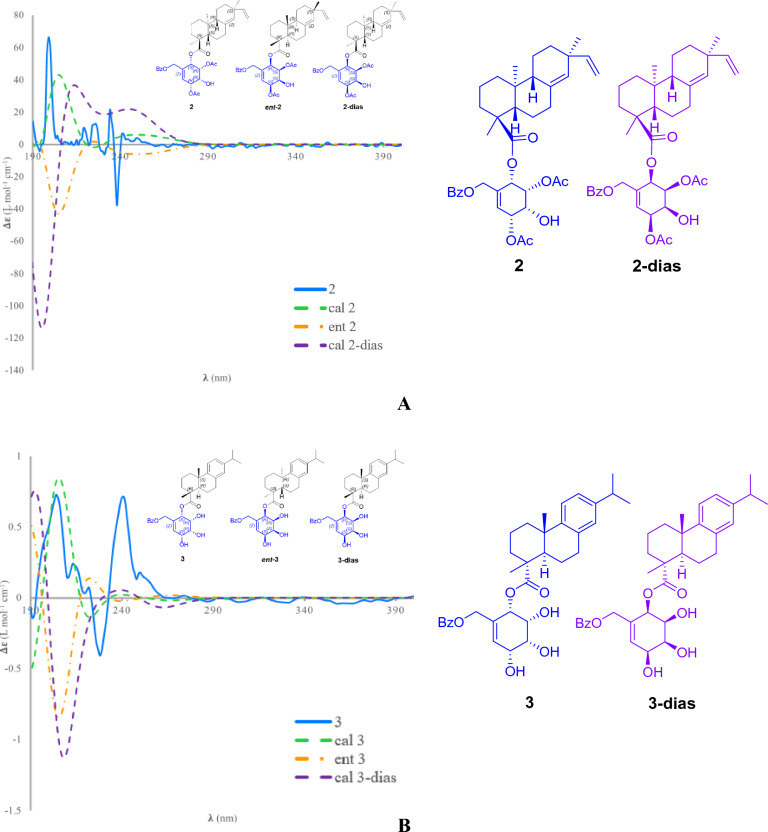

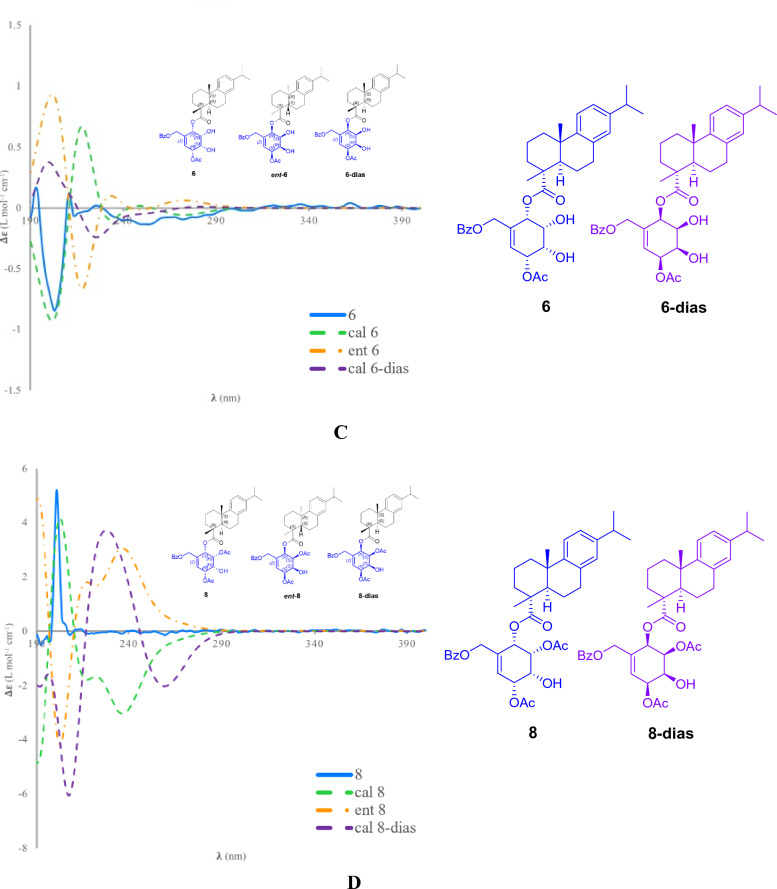
Comparison of the experimental and calculated ECD curves of compounds **2** (**A**), **3** (**B**), **6** (**C**), and **8** (**D**).

Albiflorene B (**2**) (Fig. 2) was obtained as a white powder. The molecular formula of C_38_H_48_O_9_ was deduced from the HRESIMS [M+Na]^+^ at *m/z* 671.3190 (calcd 671.3191). Compound **2** displayed almost identical UV absorption bands and IR spectrum, as well as 1D- and 2D-NMR spectra, to compound **1**, as illustrated in Fig. S12–S22, suggesting a structural similarity of these two compounds, comprising *ent*-isopimaric acid (unit A) and the cyclohexene moiety (unit B). The only difference observed was the location of two acetoxy groups at C-3ʹ and C-5ʹ on the cyclohexene ring in **2** instead. Verification of these acetoxy positions was achieved through the HMBC cross-peaks of *δ*_H_ 5.25 (H-3ʹ) and 5.46 (H-5ʹ) with the respective acetyl carbonyls at *δ*_C_ 170.4 and 171.0, and the COSY spin-coupling system from H-2ʹ to H-6ʹ for C-1ʹ to C-6ʹ which indicated their connectivity. The relative configuration of cyclohexene moiety was clearly identifiable through its coupling constants and NOESY correlations. The small coupling constant of *J*_H-2ʹ/H-3ʹ_ (5.4 Hz), *J*_H-3ʹ/H-4ʹ_ (2.5 Hz), and *J*_H-4ʹ/H-5ʹ_ (5.4 Hz) indicated a *cis* orientation of these protons, with H-5ʹ positioned in a pseudo-axial configuration due to the small coupling constant of olefinic proton H-6ʹ (2.6 Hz) (Table 1). A NOESY experiment (Fig. S18), performed on **2**, enabled the assignment of its relative stereochemistry in the same manner as described for **1**. Similar to compound **1**, the structure of compound **2** consisted of two units linked by an ester bond, resulting in two potential absolute configurations for compound **2** as (4*S*,5*S*,9*R*,10*S*,13*S*,2ʹ*S*,3ʹ*R*,4ʹ*R*,5ʹ*R*) and (4*S*,5*S*,9*R*,10*S*,13*S*,2ʹ*R*,3ʹ*S*,4ʹ*S*,5ʹ*S*), defined as **2** and **2**-dias, respectively, as depicted in Fig. 4A. Following this, the calculated ECD spectra of these two possible structures, **2** and **2**-dias, were established. By comparison them with the experimental ECD spectrum, the absolute configuration of **2** was then determined to be (4*S*,5*S*,9*R*,10*S*,13*S*,2ʹ*S*,3ʹ*R*,4ʹ*R*,5ʹ*R*).

**Figure 5:**
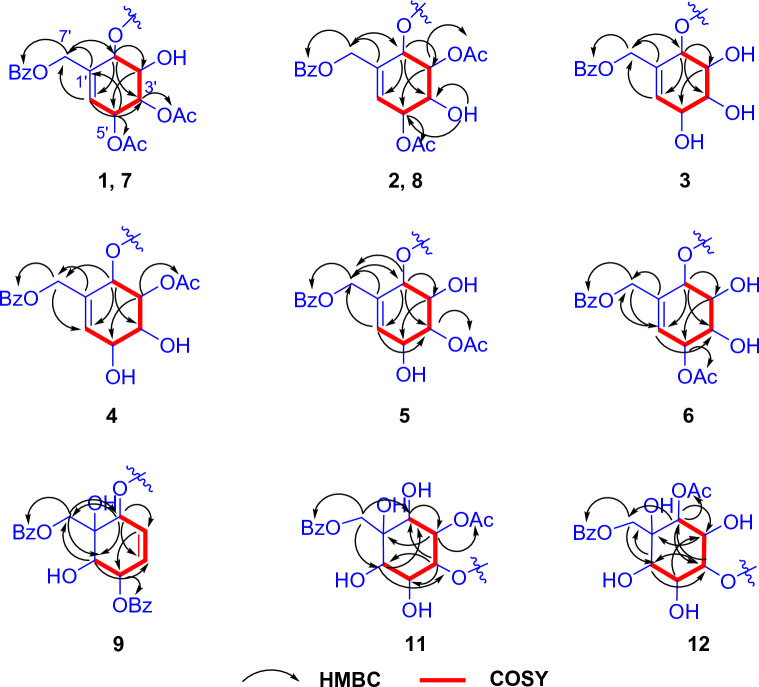
Key COSY (red bonds) and HMBC (black arrows) correlations of polyoxygenated cyclohexane ring (Unit B) of selected compounds.

Albiflorene C (**3**) (Fig. 2) presented a molecular formula C_34_H_43_O_7_, determined from the HRESIMS ion at *m/z* 563.3007 [M+Na]^+^ (calcd for 563.3003) and 1D-NMR data. Its IR spectrum showed absorption bands at 3357 cm^–1^ for hydroxy, 1717 cm^–1^ for carbonyl, and 1636 cm^–1^ for olefinic and aromatic moieties. The NMR data (Table 1) of unit A in compound **3** indicated the presence of three aromatic protons [*δ*_H_ 6.85 (d, *J* = 1.5 Hz, 1H), 7.00 (s, 1H), and 7.15 (s, 1H)], an isopropyl group [*δ*_H_ 1.21 (s, each of 3H) and 2.80 (m, 1H)], and two additional methyl singlets [*δ*_H_ 0.80, (s, 3H) and 1.21 (s, 3H)]. The 1D- and 2D-NMR spectra revealed 20 carbons, including a carboxylic carbonyl (*δ*_C_ 178.6, C-19) and six aromatic carbons between *δ*_C_ 124.1 and 146.6. These characteristics are typical of an abietane diterpenoid, resembling dehydroabietic acid^24,25^. Additionally, in the course of our isolation, the diterpenoid dehydroabietic acid (**14**) was also isolated. Its structure and absolute configuration (4*R*,5*R*,10*S*), were verified by comparing spectroscopic data, including ECD patterns, with previously recorded information. Consequently, in conjunction with biogenetic reasoning suggesting that this compound serves as another precursor for polyoxygenated cyclohexane diterpenoid esters (**3**–**12**) found within this plant, the structure and absolute stereochemistry of unit A segment in compound **3** were subsequently affirmed as dehydroabietic acid. The ^1^H-NMR data of unit B in **3** showed resonances indicative of a benzoyl group (5H) resonating between *δ*_H_ 7.41 and 8.03, along with a polyoxygenated cyclohexene moiety. This includes AB doublet methylene protons [*δ*_H_ 4.73 and 4.80 (2H, d, *J* = 13.5 Hz, H_2_-7ʹ)], four oxymethines [*δ*_H_ 5.49 (d, *J* = 3.6 Hz, H-2ʹ), 4.04 (t, *J* = 3.2 Hz, H-3ʹ), 3.75 (dd, *J* = 6.0, 2.6 Hz, H-4ʹ), and 4.39 (d, *J* = 6.0 Hz, H-5ʹ)], and one olefinic proton [*δ*_H_ 6.04 (dd, *J* = 1.9 Hz, H-6ʹ)]. These data suggested the similarity of its planar structure to 7-*O*-benzoyl streptol^26^. However, the observed small coupling constant values (*J*_H-2ʹ/H-3ʹ_ = 3.2 Hz and *J*_H-3ʹ/H-4ʹ_ = 2.6 Hz, and *J*_H-4ʹ/H-5ʹ_ = 6.0 Hz) indicated co-facial *cis* relationships of these protons. Their co-directional orientation was further confirmed by NOE experiments. Irradiation at δ 3.75 (H-4ʹ) enhanced signals at δ 4.02 (H-3ʹ) and 4.39 (H-5ʹ), while irradiation at δ 4.02 (H-3ʹ), enhanced signals at δ 5.49 (H-2ʹ) and H-4ʹ. The connection between structural units A and B recognized as the ester bond at C-19, was verified through the cross peak in the HMBC spectrum between H-2ʹ and C-19 (Fig. 5). Finally, by comparing theoretical and experimental ECD curves (Fig. 4B), the structure and absolute configuration (4*R*,5*R*,10*S*,2ʹ*S*,3ʹ*R*,4ʹ*R*,5ʹ*R*) of compound **3** was verified as shown.

Albiflorene D (**4**) (Fig. 2), a white powder, had a molecular formula of C_36_H_44_O_8_ inferred from HRESIMS and 1D-NMR data. Analysis of the 1D-NMR data indicated structural similarity between compounds **4** and **3**, with the observed 42 Da difference in molecular weights between the two compounds implying that **4** likely contained an additional acetyl substitution compare to **3**. The ^1^H-NMR spectrum of unit B revealed resonances corresponding to methines (four oxygenated) [*δ*_H_ 5.59 (d, *J* = 3.9 Hz), 5.20 (dd, *J* = 3.9, 2.5 Hz), 4.35 (br s), and 3.92 (dd, *J* = 6.8, 3.5 Hz)], an oxymethylene (*δ*_H_ 4.77, br s), an olefinic methine (*δ*_H_ 6.08, d, *J* = 2.2 Hz), and one acetyl group (*δ*_H_ 2.12, s) (Table 2). The ^13^C-NMR spectrum subsequently supported these findings, revealing signals for five oxygenated carbons (*δ*_C_ 64.2, 68.4, 69.0, 72.0, and 72.7), one benzoyl group (*δ*_C_ 128.6 × 2, 129.78, 129.9 × 2, 133.4, and 166.1), two alkene carbons (*δ*_C_ 130.6 and 131.4), and an acetyl group (*δ*_C_ 21.0 and 171.0). The connectivity (indicated by red lines) from C-2ʹ to C-6ʹ was verified by analyzing COSY correlations, and was further substantiated through the HMBC cross-peak (Fig. 5). The HMBC correlations between *δ*_H_ 4.77 (H_2_-7ʹ) and *δ*_C_ 68.4 (C-2ʹ), 130.6 (C-6ʹ), and 131.4 (C-1ʹ), confirmed the linkage of C-2ʹ, C-6ʹ, and C-7ʹ through quaternary center C-1ʹ. The observed cross-peaks of H_2_-7ʹ and both of H-2ʹʹ/H-6ʹʹ with OCO-7ʹʹ (*δ*_C_ 166.1) determined the location of the benzoyloxy group at C-7ʹ. Additionally, the position of the acetoxy group at C-3ʹ was identified by the correlations from H-3ʹ (*δ*_H_ 5.20) and the acetyl methyl group (*δ*_H_ 2.12) to OCO-3ʹ (*δ*_C_ 171.0) (Fig. 5). Comparing the coupling constants of the methine protons from H-2ʹ to H-5ʹ in **4** with those in **3**, the observed coupling constants *J*_H-2ʹ/H-3ʹ_ = 3.9 Hz and *J*_H-3ʹ/H-4ʹ_ = 2.5 Hz indicated a cofacial *cis* relationship, suggesting that unit B of **4** shared the same relative configuration as **3**. The molecular formula of albiflorene E (**5**) and albiflorene F (**6**) were determined as C_36_H_44_O_8_ by HRESIMS. Examining the NMR data (Table 2) for these compounds revealed their close resemblance to albiflorene D (**4**), with the only variation being the occurrence of an acetoxy group at different locations; C-4ʹ (*δ*_H_ 4.95/*δ*_C_ 69.1) for compound **5** and C-5ʹ (*δ*_H_ 5.44/*δ*_C_ 71.4) for compound **6**. These placements were established through HMBC correlations (Fig. 5) from H-4ʹ (δ_H_ 4.95) to the carbonyl carbon at δ_C_ 171.1 in **5** and H-5ʹ (*δ*_H_ 5.44) to the carbonyl carbon at *δ*_C_ 171.4 in **6**, together with the COSY correlations (Fig. 5) involving H-3ʹ (*δ*_H_ 4.18)/H-4ʹ (*δ*_H_ 4.95)/H-5ʹ (*δ*_H_ 4.52) in **5** and H-4ʹ (*δ*_H_ 3.93)/H-5ʹ (*δ*_H_ 5.44)/H-6ʹ (*δ*_H_ 5.95) in **6**. Furthermore, the ^1^H NMR spectra of the polyoxygenated cyclohexene moiety (unit B) in compounds **5** and **6** showed similar multiplicities and coupling constants to those of albiflorenes (**1**–**4**), indicating their consistent configurations. Consequently, the relative configurations of unit B in compounds **5** and **6** were established (Fig. 2).

**Table 2:** ^1^H and ^13^C NMR data of compounds **3–7** in CDCl_3_.

No	**3**		**4**		**5**		**6**		**7**	
	*δ*_H_	*δ*_C_	*δ*_H_	*δ*_C_	*δ*_H_	*δ*_C_	*δ*_H_	*δ*_C_	*δ*_H_	*δ*_C_
1	2.28, d (12.5) 1.45, m	37.8	2.28, m 1.46, m	37.9	2.30, m 1.47, m	38.0	2.30, d (12.4) 1.47, m	37.8	2.30, m 1.52, m	37.9
2	1.76–1.66, m	18.5	1.75, m 1.68, m	18.6	1.76–1.66, m	18.7	1.72, m 1.67, m	18.4	1.75, m	18.6
3	1.74, m 1.62, m	36.5	1.73, m 1.63, m	36.7	1.74–1.63, m	36.9	1.75, m 1.60, m	36.3	1.78, m 1.71, m	36.7
4	––	48.0	–	48.1	–	48.2	–	48.0	–	48.2
5	2.21, dd (12.4, 1.8)	44.8	2.23, dd (12.4, 1.8)	44.9	2.28, dd (12.5, 2.0)	44.6	2..23, dd (1.83, 10.65)	44.8	2.34, m	44.6
6	1.82, m 1.35, m	21.9	1.83, m 1.35, m	22.1	1.82, m 1.44, m	22.0	1.82, m 1.36, m	21.8	1.86, m	21.9
7	2.80, m	30.1	2.86–2.78, m	30.2	2.85, m	30.2	2.86–2.79, m	30.1	2.92, m	30.1
8	–	134.4	–	134.5	–	134.9	–	134.5	–	134.9
9	–	146.6	**–**	146.7	–	146.8	–	146.7	–	146.8
10	–	36.9	–	37.1	–	37.0	–	36.9	–	37.0
11	7.15, d (8.2)	124.2	7.15, d (8.2)	124.4	7.15, d (8.2)	124.3	7.18, d (8.20)	124.2	7.19, d (8.1)	124.3
12	7.00, dd (8.2, 1.7)	124.1	7.00, dd (8.2, 1.3)	124.2	7.00, dd (8.2, 1.8)	124.1	7.03, dd (8.1, 1.8)	124.1	7.03, d (8.1, 1.5)	124.1
13	–	145.8	–	146.0	–	145.9	–	145.9	–	145.9
14	6.85, d (1.5)	127.0	6.86, d (1.3)	127.2	6.87, d (1.4)	127.2	6.90, d (1.36)	127.0	6.93, s	127.1
15	2.80, m	33.4	2.82, m	33.6	2.82, m	33.6	2.82, s	33.4	2.85, s	33.6
16	1.21, s	23.96^a^	1.22, s	24.12^a^	1.22, s	24.11^a^	1.23^a^, s	23.94^a^	1.26^e^, s	24.1^b^
17	1.21, s	23.92^a^	1.21, s	24.08^a^	1.21, s	24.08^a^	1.21^a^, s	23.96^a^	1.24^e^, s	24.07^b^
18	1.26, s	16.5	1.27, s	16.7	1.28, s	16.6	1.28, s	16.5	1.22, s	16.6
19	–	178.6	–	177.4	–	178.6	–	178.6	–	178.6
20	1.18, s	25.2	1.19, s	25.5	1.19, s	25.4	1.19, s	25.3	1.30, s	25.4
1ʹ	–	130.9	–	131.4	–	132.0	–	133.9	–	134.5
2ʹ	5.49, d (3.6)	71.0^b^	5.59, d (3.9)	68.4	5.51, d (4.8)	68.4	5.62, d (4.8)	70.6	5.63, d (5.5)	70.6
3ʹ	4.02, t (3.2)	71.1^b^	5.20, dd (3.9, 2.5)	72.7	4.18, ddd (4.8, 2.6, 2.4)	70.9	3.98, dd (4.8, 2.7)	71.2	4.18, br s	69.3
4ʹ	3.75, dd (6.0, 2.6)	73.0	3.92, dd (6.8, 2.5)	72.0	4.95, dd (6.4, 2.6)	69.1	3.93, dd (5.0, 2.7)	70.8	5.18, dd (5.8, 2.5)	72.4
5ʹ	4.39, d (6.0)	68.7	4.35, br s	69.0	4.52, br t	66.6	5.44, t (5.0)	71.4	5.58, t (5.0)	68.0
6ʹ	6.04, d (1.9)	130.7	6.08, d (2.2)	130.6	6.06, d (2.3)	129.8	5.95, d (2.6)	125.9	6.01, d (2.5)	125.7
7ʹ	4.80, d (13.5) 4.73, d (13.5)	64.3	4.77, s	64.2	4.83, d (13.4) 4.77, d (13.4)	64.2	4.82, d (13.9) 4.76, d (13.5)	64.1	4.84, d (14.0) 4.78, d (14.0)	64.1
1ʹʹ	–	129.6	–	129.77	–	129.7	–	129.6	–	129.6
2ʹʹ	8.03, m	129.7	8.04, dd (8.4, 1.3)	129.9	8.04, dd (8.2, 1.1)	129.9	8.03, m	129.7	8.05, m	127.9
3ʹʹ	7.41, t (7.9)	128.5	7.44, t (8.0)	128.6	7.44, tt (8.1, 1.3)	128.6	7.45, m	128.5	7.47, m	128.6
4ʹʹ	7.53, m	133.2	7.58, tt (8.2, 1.3)	133.4	7.58, tt (8.2, 1.3)	133.4	7.58, tt (7.4, 1.3)	133.3	7.60, m	133.4
5ʹʹ	7.41, t (7.91)	128.5	7.44, t (8.0)	128.6	7.44, t (7.9)	128.6	7.45, m	128.5	7.47, m	128.6
6ʹʹ	8.03, m	129.7	8.04, dd (8.4, 1.3)	129.9	8.04, dd (8.2, 1.1)	129.9	8.03, m	129.7	8.05, m	129.9
7ʹʹ	–	166.1	–	166.1	–	166.1	–	165.9	–	166.1
OCOCH_3_			2.12, s	21.1	2.17, s	21.3	2.12, s	21.1		21.1^c^
OCOCH_3_										21.19^c^
OCOCH_3_				171.0		171.1		171.4		170.0^d^
OCOCH_3_										170.3^d^

^a,b,c,d,e,f^Overlap and interchangeable values in the same column.

Given the close resemblance of the structures of compounds **4**–**6** with **3**, which shared the same unit A and differed slightly in the placement of hydroxy and acetoxy substituents on unit B, along with the biogenesis rationale of the compounds being generated from the same plant, the absolute configurations of compounds **4**–**6** were proposed as (4*R*,5*R*,10*S*,2ʹ*S*,3ʹ*R*,4ʹ*R*,5ʹ*R*), (4*R*,5*R*,10*S*,2ʹ*S*,3ʹ*S*,4ʹ*R*,5ʹ*R*) and (4*R*,5*R*,10*S*,2ʹ*S*,3ʹ*R*,4ʹ*S*,5ʹ*R*), respectively. The reaffirmation of the absolute configuration could be observed in the ECD curve of **6** which displayed the first negative Cotton effect at λ_max_ 203 nm (∆*ε* = –25.3), corresponding well to the theoretical ECD curve (Fig. 4C).

Based on the HRESIMS ion peak of albiflorene G (**7**) at *m/z* 669.3051 [M+Na]^+^ (calcd, 669.3034) and albiflorene H (**8**) at *m/z* 647.3196 [M + H]^+^ (calcd, 647.3215), a molecular formula of C_38_H_46_O_9_ was suggested for both compounds. Careful analysis of the NMR data (Tables 2 and 3) revealed that signals in **7** and **8** exhibited structural similarities with albiflorenes A (**1**) and B (**2**), respectively. However, a notable difference was observed in the core structure unit A, where dehydroabietic acid replaced *ent*-isopimaric acid (Fig. 2). HMBC correlations of **7** from H-2ʹ (*δ*_H_ 5.63) to C-19 (*δ*_C_ 178.6), and of **8** from H-2ʹ (*δ*_H_ 5.79) to C-19 (*δ*_C_ 177.3), verified that the polyoxygenated cyclohexene and the dehydroabietic moieties were linked through C-2ʹ–O–C-19. The planar structure of polyoxygenated cyclohexene diterpene ester was determined from the COSY and HMBC correlations, as shown in Fig. 5. Additionally, the almost identical multiplicities and coupling constants of the cyclohexene ring proton resonances (H-2ʹ to H-6ʹ) of **7** and **8** in comparison to those found in albiflorenes A (**1**) and B (**2**), suggested that they possessed identical relative configurations. Furthermore, in addition to the NOESY correlations observed between H-2ʹ/H-3ʹ, H-3ʹ/H-4ʹ, and H-4ʹ/H-5ʹ in both compounds, compound **7** also exhibited an additional correlation between H-2ʹ and H-4ʹ, while compound **8** showed a correlation between H-3ʹ and H-5ʹ. These correlations provided confirmation of their co-facial alignment within the six-membered ring. Additional support was observed in the NOE experiments, where irradiation at *δ*_H_ 4.18 (H-3ʹ) amplified signals at *δ*_H_ 5.63 (H-2ʹ) and 5.18 (H-4ʹ), while irradiation at *δ*_H_ 5.18 (H-4ʹ) amplified signals at *δ*_H_ 2.39 (H-3ʹ) and 5.58 (H-4ʹ). For the orientation of the ester bond linking C-19 of dehydroabietic acid to C-2ʹ of the cyclohexene moiety, similar to compound **1**, where unit B consisted of identical cyclohexene moiety, the absolute configuration of the unit B in compound **7** could not be determined through comparison with calculated spectra. Consequently, the absolute configuration of compound **7** was assigned as (4*R*,5*R*,10*S,*2ʹS*,3ʹS*,4ʹS*,5ʹR*). On the other hand, in the case of compound **8** where the structure of cyclohexene ring is identical to compound **2**, the determination of the absolute configuration of **8** could be achieved by comparing the experimental ECD spectra with the calculated ECD spectra of two possible absolute configurations for compound **8**, (4*R*,5*R*,10*S*,2ʹ*S*,3ʹ*R*,4ʹ*R*,5ʹ*R*) and (4*R*,5*R*,10*S*,2ʹ*R*,3ʹ*S*,4ʹ*S*,5ʹ*S*) (Fig. 4D). The matching ECD curves, notably at λ_max_ ~ 200 nm, facilitated the determination of the absolute configuration of compound **8** as 4*R*,5*R*,10*S*,2ʹ*S*,3ʹ*R*,4ʹ*R*,5ʹ*R*.

**Table 3:** ^1^H and ^13^C NMR data of compounds **8–12** in CDCl_3_.

No	**8**		**9**		**10**		**11**		**12**	
	*δ*_H_	*δ*_C_	*δ*_H_	*δ*_C_	*δ*_H_	*δ*_C_	*δ*_H_	*δ*_C_	*δ*_H_	*δ*_C_
1	2.29, m 1.47, m	37.9	2.29, d (12.80) 1.49–1.46, m	37.9	2.31, m 1.50, m	37.9	2.25, d (12.5) 1.40, m	38.0	2.26, d (4.9) 1.47, m	38.0
2	1.74, m 1.68, m	18.6	1.78, m 1.75–1.69, m	18.6	1.80 m 1.73, m	18.7	1.68, m 1.62, m	18.6	1.734–1.68, m 1.64–1.62	18.6
3	1.69, m 1.63, m	36.7	1.75–1.69, m 1.66, m	37.0	1.78–1.71, m	37.6	1.66, m 1.58, m	36.5	1.734–1.68, m 1.64–1.62	36.8
4	–	48.1	–	48.0	–	48.1	–	48.0	–	48.1
5	2.24, dd (12.5, 1.9)	44.8	2.23, dd (12.5, 1.9)	44.9	2.25, dd (12.4, 1.9)	45.0	2.14, dd (12.4, 2.0)	45.2	2.19, dd (12.5, 2.0)	45.1
6	1.81, m 1.33, m	22.1	1.85, m 1.49–1.46, m	22.0	1.87, td (12.3, 6.6) 1.64, dd (13.2, 7.4)	22.1	1.79, m 1.46, m	21.7	1.80, m 1.49, m	21.8
7	2.86–2.74, m	30.2	2.87, m	30.0	2.98, ddd (17.0, 11.0, 7.0) 2.89, dd (17.0, 7.0)	30.3	2.86, dd (9.0, 4.3)	30.3	2.87, m	30.3
8	–	134.6	–	134.6	–	134.9	–	134.7	–	134.7
9	–	146.7	–	146.7	–	146.8	–	146.8	–	146.7
10	–		–	37.1	–	37.2	–	37.1	–	37.1
11	7.16, d(8.2)	124.3^*a*^	7.15, d (8.2)	124.2^*a*^	7.17, d (8.2)	124.3^*b*^	7.14, d (8.3)	124.3	7.14, d (8.2)	124.2
12	7.01, dd (8.1, 1.3)	124.2^*a*^	7.00, dd (8.1,1.7)	124.1^*a*^	7.01, dd (8.1, 1.7)	124.0^*b*^	7.00, dd (8.04, 2.0)	124.2	6.99, dd (8.1, 1.0)	124.2
13	–	146.0	–	146.0	–	145.9	–	146.0	–	146.0
14	6.87, d (1.3)	127.2	6.88, d (1.2)	127.2	6.90, d (1.3)	127.2	6.88, d (2.0)	127.1	6.87, d (1.3)	127.1
15	2.86–2.74, m	33.6	2.81, q (7.0)	33.6	2.83, q (7.0)	33.6	2.81, q (7.0)	33.6	2.81, q (7.0)	33.6
16	1.23^a^	24.1	1.21, s	224.1	1.22^a^, s	24.1	1.21, s	24.1	1.22, s	24.1
17	1.21^a^	24.1	1.21, s	24.1	1.21^a^, s	24.1	1.21, s	24.1	1.21, s	24.1
18	1.27, s	16.7	1.31, s	16.8	1.41, s	17.1	1.16, s	16.6	1.21, s	16.7
19	–	177.3	–	177.8	–	177.6	–	177.8	–	178.5
20	1.19, s	25.5	1.20, s	25.5	1.25, s	25.7	1.15, s	25.3	1.17, s	25.4
1ʹ	–	134.4	–	75.1	–	75.3	–	63.5	–	61.4
2ʹ	5.79, d (5.3)	67.7	5.57, d (3.1)	70.5	5.67, d (3.3)	70.7	4.23, t (5.0)	67.4	5.45, d (5.5)	70.7
3ʹ	5.22, dd (5.3, 2.5)	72.7	5.93, dd (10.1, 3.1)	126.5	4.28, dt (9.6, 3.3)	70.8	5.13, dd (5.0, 2.0)	72.3	3.96, td (5.1, 2.0)	69.3
4ʹ	4.1, m	69.3	5.97, ddd (10.1, 3.1, 1.0)	128.7	3.96, td (9.6, 3.5)	72.6	5.15, dd (5.0, 2.0)	71.5	5.04, dd (6.0, 2.0)	74.5
5ʹ	5.43, dd (5.0, 3.0)	70.8	5.74, dt (6.3, 1.0)	73.9	5.62, t (9.9)	73.8	4.12, m	67.4	4.18, ddd (6.0, 6.0, 3.0)	67.0
6ʹ	5.97, m	126.2	4.20, d (6.3)	71.7	5.45, d (9.9)	71.8	3.78, d (2.4)	62.8	3.67, d (3.3)	61.1
7ʹ	4.76, m	64.1	4.95, d (12.2) 4.39, d (12.2)	66.9	4.59, d (12.2) 4.08, d (12.2)	65.7	4.66, d (12.1) 4.41, d (12.1)	64.0	4.49, d (12.2) 4.38, d (12.2	64.2
1ʹʹ	–	129.7	–	129.5	–	129.2^c^	–	129.3	–	129.4
2ʹʹ	8.03, m	129.9	8.04, m	130.0	8.05–8.04, m	130.1	8.02, m	129.9	8.01, m	129.9
3ʹʹ	7.45, m	128.6	7.42–7.38, m	128.6	7.47–7.45, m	128.5	7.45, m	128.7	7.44, m	128.7
4ʹʹ	7.58, m	133.4	7.57–7.53, m	133.6^b^	7.61–7.57, m	133.8^d^	7.59, m	133.7	7.58, m	133.6
5ʹʹ	7.45, m	128.6	7.42–7.38, m	128.6	7.47–7.45, m	128.5	7.45, m	128.7	7.44, m	128.7
6ʹʹ	8.03, m	129.9	8.04, m	130.0	8.05–8.04, m	130.1	8.02, m	129.9	8.01, m	129.9
7ʹʹ	–	166.0	–	167.3	–	167.3	–	166.1	–	166.0
OCOCH_3_	2.13^b^	21.2^b^			1.96, s	20.7	1.94, s	20.8	2.17, s	21.0
OCOCH_3_	2.12^b^	21.1^b^			–	170.1	–	170.1	–	171.0
OCOCH_3_		170.5^c^								
OCOCH_3_		171.1^c^								
1ʹʹʹ			–	129.5	–	129.1^*c*^				
2ʹʹʹ			7.94, m	129.9	8.05–8.04, m	130.1				
3ʹʹʹ			7.42–7.38, m	128.6	7.47–7.45, m	128.5				
4ʹʹʹ			7.57–7.53, m	133.5^*b*^	7.61–7.57, m	133.7^*d*^				
5ʹʹʹ			7.42–7.38, m	128.6	7.47–7.45, m	128.5				
6ʹʹʹ			7.94, m	133.5	8.05–8.04, m	130.1				
7ʹʹʹ			–	167.3	–	166.9				
OH-1ʹ					3.12, br s					
OH-3ʹ					2.42, br s					
OH-4ʹ					2.69, br s					

^a,b,c,d,e,f^Overlap and Interchangeable values in the same column.

Albiflorene I (**9**) was isolated as a yellow powder and its HRESIMS spectrum showed a [M+Na]^+^ peak at m/z 689.3071, consistent with a molecular formula of C_41_H_46_O_8_. Preliminary assessment of the spectroscopic analysis of **9**, utilizing 1D- and 2D-NMR data, revealed dehydroabietic acid and a polyoxygenated cyclohexene moiety (Table 3). This compound resembled the core structure of **3** except for the inclusion of an extra benzoyl group and a disubstituted double bond instead of trisubstituted double bond in the polyoxygenated cyclohexene ring (unit B). The ^1^H-NMR data (Table 3) of unit B showed resonances for two benzoyl groups [*δ*_H_ 7.94 (2H, m), 7.42–7.38 (4H, m), 7.57–7.53 (2H, m), and 8.04 (2H, m)], two olefinic protons [*δ*_H_ 5.93 and 5.97 (each 1H, dd,* J* = 10.1, 3.1 Hz)], three oxymethine protons [*δ*_H_ 4.20 (d, *J* = 6.3 Hz), 5.57 (d, *J* = 3.1 Hz), and 5.74 (dt, *J* = 6.3, 1.0 Hz)], and two diastereotopic methylene protons [*δ*_H_ 4.39 and 4.95 (each 1H, d, *J* = 12.2 Hz)]. The ^13^C-NMR data (Table 3) of unit B exhibited resonances for two benzoyl groups [*δ*_C_ 128.6 × 2, 129.5 × 2, 129.9, 130.0, 133.5, 133.6, and 167.3 × 2], two olefinic carbons (*δ*_C_ 126.5 and 128.7), an oxygenated carbon (*δ*_C_ 75.1), three oxymethine carbons (*δ*_C_ 70.5, 71.7, and 73.9), and an oxymethylene carbon (*δ*_C_ 66.9). These NMR data presented were characteristics of a polyoxygenated cyclohexene ring, resembling those found in (–)-6-acetylzeylenol^27,28^, with a distinct difference involving the conjugation of the hydroxy group at C-2ʹ, forming an ester linkage (C-2ʹ–OCO-19), with dehydroabietic acid in **9** instead of acetyl group in (–)-6-acetylzeylenol. This ester linkage was confirmed by the HMBC correlation of H-2ʹ (*δ*_H_ 5.57) with the dehydroabietic acid carbonyl carbon (*δ*_C_ 177.8). The positions of benzoyloxy groups at C-1ʹ (*δ*_C_ 75.1) and C-5ʹ (*δ*_C_ 73.9) were established through the HMBC correlations from H-2ʹ to C-7ʹ (*δ*_C_ 66.9) and from H-5ʹ to C-3ʹ (*δ*_C_ 128.7), and the olefinic protons at H-3ʹ (*δ*_H_ 5.97) and H-4ʹ (*δ*_H_ 5.93 /*δ*_C_ 126.5) were identified by their cross-peaks with C-2ʹ (*δ*_C_ 70.5) and C-5ʹ. Furthermore, the correlations of H-2ʹ/H-3ʹ and H-5ʹ/H-6ʹ in the COSY spectrum indicated connectivity of the protons and carbons in cyclohexene ring. The relative stereochemistry of the cyclohexene ring was deduced from the coupling constant between H-2ʹ and H-3ʹ (*J*_H-2ʹ/H-3ʹ_ = 3.1 Hz) and between H-5ʹ with H-6ʹ (*J*_H-5ʹ/H-6ʹ_ = 6.3 Hz), which indicated that H-2ʹ adopted a *pseudo*-equatorial orientation, and existed a *trans* diaxial relationship between H-5ʹ and H-6ʹ in the favored half-chair conformation (Fig. 3). The methylene protons (H_2_-7ʹ) of the (benzoyloxy)methyl group showed a cross-peak with H-6ʹ in NOESY spectrum, indicating their positioning on the same face of the cyclohexene ring. The above data confirmed their closed resemblance of their structure and relative stereochemistry of the polyoxygenated cyclohexene moiety in **9** to that of (–)-6-acetylzeylenol. However, their optical rotations displayed opposite directions (${\left[\alpha \right]}_{\text{D}}^{26}$= + 38.4 for **9** and ${\left[\alpha \right]}_{\text{D}}^{23}$= – 63.5 for (–)-6-acetylzeylenol), suggesting that unit B in compound **9** likely had an opposite stereochemistry. Based on the provided data, the structure of compound **9** was proposed to have the (4*R*,5*R*,10*S*,1ʹ*R*,2ʹ*R*,5ʹ*S*,6ʹ*R*) absolute configuration.

**Figure 6:**
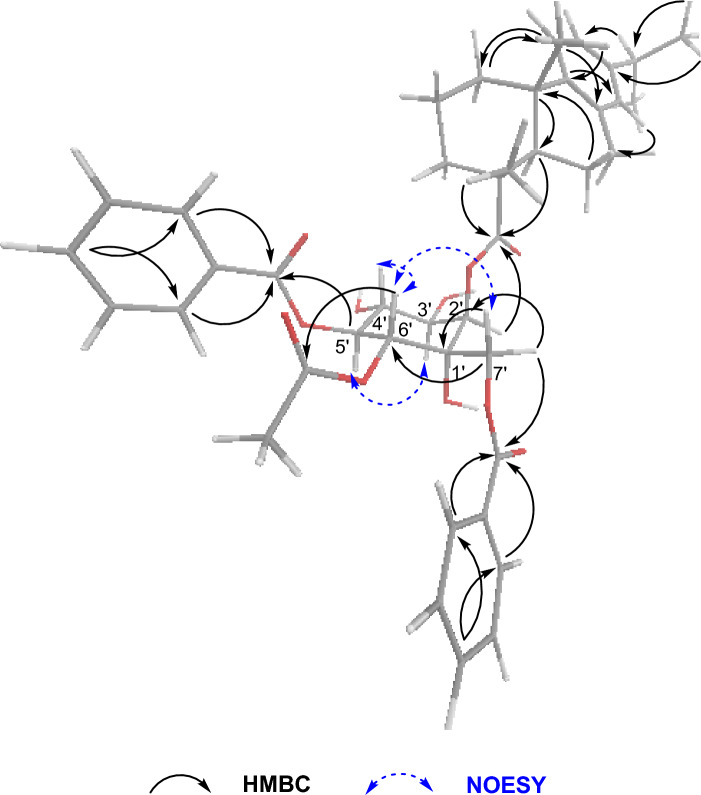
Selected HMBC & NOESY correlations for the assignment of **10**.

Albiflorene J (**10**) was obtained as a yellow powder. Its HRESIMS spectrum revealed a peak [M+Na]^+^ at *m/z* 743.3426 (calcd, 743.3426), corresponding to the molecular formula of C_43_H_51_O_11_. The NMR data (Table 3) of compound **10** revealed its constitution, comprising a dehydroabietic acid (unit A) and a polyoxygenated cyclohexane moiety (unit B) moieties. In unit B, signals corresponding to two benzoyl groups, one acetate group, five oxygenated methines, one oxygenated methylene, and one oxygenated quaternary were identified (Fig. 2). A contiguous spin system formed by H-2ʹ (*δ*_H_ 5.67)/H-3ʹ (*δ*_H_ 4.28)/H-4ʹ (*δ*_H_ 3.96)/H-5ʹ (*δ*_H_ 5.62)/H-6ʹ (*δ*_H_ 5.45) as evidenced by COSY and HSQC correlations (Fig. 5), allowed the identification of the positions of OH-3ʹ and OH-4ʹ on their respective carbons. The two benzoyloxy groups were placed at C-5ʹ (*δ*_C_ 73.8) and C-7ʹ (*δ*_C_ 65.7) by HMBC correlations of H-5ʹ with *δ*_C_ 166.9 (CO) and H_2_-7ʹ (*δ*_H_ 4.08 and 4.59) with *δ*_C_ 167.3 (CO), respectively. Additionally, the acetoxy group was identified to be located at C-6ʹ (*δ*_C_ 71.7) by the HMBC correlation between H-6ʹ and the acetyl carbonyl at *δ*_C_ 170.1 (Fig. 6). NOESY experiments involving H-2ʹ/H-5ʹ, H-3ʹ/H-5ʹ, H-4ʹ/H-6ʹ, and H-6ʹ/H_2_-7ʹ, while showing the absence of a cross peak between H_2_-7ʹ and H-2ʹ, provided evidence for a chair conformation of the cyclohexane ring and indicated *α*-linkage at C-2ʹ with the diterpene acid ester (Fig. 6). In this conformation, the protons of C-4ʹ, C-6ʹ, and CH_2_-7ʹ linked to C-1ʹ were aligned on the same face, while the protons of C-2ʹ, C-3ʹ, and C-5ʹ were oriented in the opposite direction. Therefore, the relative stereochemistry of unit B of compound **10** was established. The ECD pattern observed in the spectrum of compound **10** could not provide a conclusive determination of the absolute configuration of unit B by comparing it with the calculated spectrum. As a result, the absolute configuration of compound **10** was assigned as (4*R*,5*R*,10*S*,1ʹS*,2ʹR*,3ʹR*,4ʹR*,5ʹS*,6ʹR*).

Albiflorene K (**11**), a yellow powder, had the molecular formula C_36_H_46_O_10_ inferred from HRESIMS. The ^1^H- and ^13^C-NMR spectra of **11** (Table 3) revealed a dehydroabietic acid (unit A) and a polyoxygenated cyclohexane moiety (unit B), similar to albiflorene J (**10**). However, differences associated with the substitutions on the cyclohexane ring in unit B and the linkage of the two units were observed. In unit B, the ^1^H-NMR resonance assignments (H-2ʹ to H-6ʹ) were identified using sequential correlations in the COSY spectrum (Fig. 5). The chemical shift of H-2ʹ suggested that there was no ester bond formation on the hydroxy group at C-2ʹ, which was now positioned in the *β*-orientation. The position of acetoxy substitutions were determined through HMBC spectra which showed long-range correlations from the ring methine protons H-3ʹ (*δ*_H_ 5.13) to an acetyl carbonyl at *δ*_C_ 170.1, confirming the location of acetoxy group at C-3ʹ. Furthermore, the HMBC cross-peaks from H-4ʹ (*δ*_H_ 3.15) to C-19 (*δ*_C_ 177.8) verified the connection between the polyoxygenated cyclohexane and the dehydroabietic moieties through C-4ʹ–O–CO-19 rather than the C-2ʹ–O–CO-19 linkage previously identified in compounds **1**–**10**. Another isomer, compound **12**, showed a molecular formula of C_36_H_46_O_10_ inferred from HRESIMS analysis. Although, its ^1^H and ^13^C NMR spectra displayed similarities to those of **11**, the resonance of H-2ʹ of **12** exhibited a downfield shifted instead of that of H-3ʹ, indicating the placement of an acetoxy group at C-2ʹ in **12**. This acetoxy group at C-2ʹ was further confirmed by a cross-peak detected in the HMBC spectrum from H-2ʹ (*δ*_H_ 5.45) to the acetyl carbonyl (*δ*_C_ 171.0). Additionally, HMBC correlations between H-4ʹ (*δ*_H_ 5.04) and C-19 (*δ*_C_ 178.5) also verified the linkage between the polyoxygenated cyclohexene and the dehydroabietic moieties via C-4ʹ–O–CO-19. In the NOESY spectra of **11** and **12**, the presence of a cross peak between H-7ʹ and H-2ʹ, and between H-7ʹ and H-6ʹ were observed. These cross-peaks clearly indicated an *β*-orientation of these protons. Furthermore, H-2ʹ, H-3ʹ, H-4ʹ, and H-5ʹ were found to be on the same side of the structure as in *β-*orientation according to the small coupling constants ranging from 2.0 to 6.0 Hz observed between these protons in both **11** and **12**. Therefore, the relative stereochemistry of unit B of both compounds was established. Unfortunately, comparing the quantum chemical calculations of the ECD spectra with the experimental spectra of these two compounds proved difficult in ascertaining the absolute configurations of unit B. Consequently, the absolute stereochemistry of compounds **11** and **12** were identified as (4*R*,5*R*,10*S*,1ʹS*,2ʹR*,3ʹS*,4ʹR*,5ʹR*,6ʹS*), and (4*R*,5*R*,10*S*,1ʹS*,2ʹR*,3ʹR*,4ʹR*,5ʹR*,6ʹS*), respectively.

It is worth mentioning that polyoxygenated cyclohex(a/e)nes, existing as individual molecules, are prevalent in the Annonaceae^29^ family, and are found in lower abundance in the Zingiberaceae^28^ and Euphorbiaceae^30^ families. The polyoxygenated cyclohex(a/e)ne diterpene esters **1**–**12,** represent the first occurrence of a polyoxygenated cyclohex(a/e)ne and diterpenoid conjugate. Their existence should potentially serve as a chemotaxonomic marker for *K. albiflora*.

The nine, previously recognized, compounds isolated from *K. albiflora* were identified as *ent*-isopimar-8(14),15-dien-19-oic acid (**13**)^21^, dehydroabietic acid (**14**)^24^, dehydroabietinol (**15**)^31^, 18-norabieta-8,11,13-trien-4-ol (**16**)^32^, pomiferin D (**17**)^33^, 7-oxodehydroabietinol (**18**)^31^, boesenboxide (**19**)^34^, (–)-1,6-desoxytingtanoxide (**20**)^35,36^, and stigmasterol stearate (**21**)^37^ by comparison of their experimental and reported spectroscopic data.

Except for compounds **2**, **4**, and **5**, which were available in limited quantities, the remaining seventeen compounds (**1**, **3**, and **6**–**20**) were evaluated for their antimicrobial activity against eight bacterial strains, including gram-positive bacteria *Staphylococcus aureus*, *Staphylococcus epidermidis*, *Enterococcus faecalis*, and *Bacillus cereus*, as well as gram-negative bacteria *Pseudomonas aeruginosa*, *Escherichia coli*, *Shigella flexneri*, and *Salmonella* Typhimurium. Among the polyoxygenated cyclohexane diterpene esters, compound **3** showed the most potent inhibitory activity against *B. cereus* strain with MIC value of 3.13 μg/mL and MBC value of 6.25 μg/mL (see Table S1). These values suggested that compound **3** exhibited bactericidal activity against bacterial organisms, as its MIC index (MIC/MBC) is less than four^38^. The other polyoxygenated cyclohex(a/e)ne diterpene esters (**1** and **6**–**12**) did not exhibit noteworthy antibacterial activity and their MIC values exceeded 200 μg/mL. Interestingly, the substituents on the cyclohexene moiety appeared to be crucial as only the presence of free hydroxy substituents (as in **3**) showed enhanced activity, whereas the replacement of the OH group with the OAc group at any position(s) resulted in the decrease of activity, as observed in compounds **3**–**8**. For other known compounds isolated from *K. albiflora*, *ent*-isopimar-8(14),15-dien-19-oic acid (**13**) exhibited inhibitory activity against the *B. cereus* strain with MIC and MBC values of 6.25 and 25.0 μg/mL, respectively (see Table S1). In addition to this, it was previously reported to be active against both pathogens, *Streptococcus mutans* ATCC 25175 and *Actinomyces viscosus* ATCC 27044, with MIC values of 125.0 μg/mL^21^. It should be pointed out that the dehydroabietic acid (**14**), the diterpenoid core structure of compounds **3**–**12,** showed less potent inhibitory activity compared to compound **13**, the diterpenoid core structure of compounds **1**–**2**. Therefore, exploring other polyoxygenated cyclohex(a/e)ne substituents on compound **13** might be a worthwhile avenue for further investigation.

## METHODS

### General experimental procedures

Sephadex LH-20 (GE Healthcare Bio-Sciences AB) and silica gel 60 (Merck, 0.063–0.200 mm and less than 0.063 mm) were used for column chromatography (CC). TLC was visualized under UV light at 254 and 366 nm to monitor the outcomes, followed by spraying with Godin’s reagent and heating to identify UV-negative compounds and detect color changes in UV-positive spots. Analytical and semipreparative HPLC were performed using Waters Delta 1525 pumps with a Waters 2998 photodiode array detector set at 210 nm to monitor the compounds of interest. NMR spectra were acquired using Bruker Advance 400 and 600 NMR spectrometers. HRESIMS analysis was achieved using Bruker Micro TOF_LC_ spectrometer. Optical rotation was measured on a JASCO P-1020 polarimeter and IR spectra on a Perkin-Elmer Spectrum One Spectrophotometer using the ATR (a universal attenuated total reflectance) technique. ECD spectra were recorded on a JASCO J-810 spectropolarimeter.

### Plant materials

The whole plants of *K. albiflora* Jenjitt. & Ruchis., belonging to the Zingiberaceae, were collected in May 2021 from Mueang Tak district of Tak province, Thailand, at the GPS location (Lat. 16°52′22.7″N, Long, 99°19′27.4″E). Researchers have obtained permission, along with a consent form, to utilize the benefits of Thai medicinal plants from the Department of Agriculture of Thailand. Identification of the plant material was conducted by a taxonomist, Assist. Prof. Dr. Saroj Ruchisansakun. A voucher specimen (accession SR 1511) has been placed in the collection at the Suan Luang Rama IX Botanic Garden, Thailand. All procedures for plant collection were performed in accordance with relevant institutional, national, and international guidelines and legislation.

### Extraction and isolation

The 7.7 kg of the shade-dried whole plants of *K. albiflora* were macerated and soaked in 22 L of 95% EtOH, 13.0 L of 50% CH_2_Cl_2_-MeOH, and 4.0 L of EtOAc for 48 h (2 times) at room temperature, respectively. After filtration, the solvent was evaporated and dried under vacuum, resulting in 163.8 g of EtOH extract, 42.5 g of 50% CH_2_Cl_2_–MeOH extract, and 5.0 g of EtOAc extract.

A total of 53.52 g of whole plant extracts from *K. albiflora*, derived by combining 50% CH_2_Cl_2_–MeOH and EtOAc crude extracts due to their closely matching NMR spectra, underwent CC using a silica gel column with a gradient of hexane–CH_2_Cl_2_ (100:0 → 0:100) followed by CH_2_Cl_2_–MeOH (100:0 → 80:20), resulting in 39 fractions (F1–F39). F3 (emerging in 20% CH_2_Cl_2_–hexane, 734.1 mg) was separated on a silica gel column eluting with a gradient of hexane–CH_2_Cl_2_ (100:0 → 0:100), yielding 10 fractions (F3.1–F3.10). Compound **21** (123.7 mg) was obtained from F3.10. F13 (emerging in 70% CH_2_Cl_2_–hexane, 257.9 mg) was separated using preparative HPLC (YMC-Pack ODS-A, 20 × 250 mm) using a gradient of MeCN–H_2_O (80:20 → 100:0) over 30 min followed by 100% MeCN for 20 min, at a flow rate of 10 mL/min, yielding compounds **16** (*t*_R_ 15.84 min, 5.6 mg) and **17** (*t*_R_ 19.55 min, 8.4 mg). Additionally, compound **20** (2.3 mg) was purified by analytical HPLC, transitioning from *t*_R_ 11.28 min to *t*_R_ 21.81 min), using a gradient of MeCN–H_2_O (80:20 → 100:0) over 30 min. F15 (emerging in 80% CH_2_Cl_2_–hexane, 324.2 mg) was separated using preparative HPLC (C18, Hichrome, 10 × 250 mm) using a gradient of MeCN–H_2_O (40:60 → 100:0) over 100 min followed by 100% MeCN for 15 min, at a flow rate of 10 mL/min, to obtain compounds **14** (*t*_R_ 56.73 min, 45.7 mg) and **13** (*t*_R_ 73.0 min, 41.2 mg). Additionally, a peak at *t*_R_ 80.27 min was further purified using analytical HPLC (C18, YMC, 4.6 × 250 mm, gradient, 90:10 to 100:0 MeCN–H_2_O in 30 min, flow rate 1 mL/min] yielding compounds **3** (*t*_R_ 11.0 min, 8.5 mg) and **6** (*t*_R_ 16.48 min, 2.2 mg). F16 (emerging in 85% CH_2_Cl_2_–hexane, 326.3 mg) was separated using preparative HPLC (C18, YMC, 20 × 250 mm) using a gradient of MeCN–H_2_O (90:10 → 100:0) over 30 min followed by 100% MeCN for 20 min, at a flow rate of 10 mL/min, yielding compounds **14** (*t*_R_ 11.0 min, 94.8 mg), **15** (*t*_R_ 19.63 min, 0.6 mg), and **13** (*t*_R_ 21.53 min, 21.2 mg), respectively. F20 (emerging in 1% MeOH–CH_2_Cl_2_, 175.9 mg) was separated using preparative HPLC (C18, YMC, 20 × 250 mm) using a gradient of MeCN–H_2_O (40:60 → 100:0) over 100 min followed by 100% MeCN for 15 min, at a flow rate of 10 mL/min, yielding compounds **19** (*t*_R_ 38.25 min, 35.8 mg) and **14** (*t*_R_ 60.7 min, 52.3 mg). A portion of F22 (emerging in 3% MeOH–CH_2_Cl_2_, 11.77 g), (1000 mg × 3 times) were chromatographed over Sephadex LH-20, using a MeOH–CH_2_Cl_2_ (80:20) solvent system, to afford 5–7 subfractions F22.1 (7 subfractions, F22.1.1–F22.1.7); F22.2 (5 subfractions, F22.2.1–F22.2.5); F22.3 (5 subfractions, F22.3.1–F22.3.5). Then, subfraction F22.1.5 (641 mg) was purified through preparative HPLC (C18, YMC, 20 × 250 mm) using a gradient of MeCN–H_2_O (30:70 → 100:0) over 120 min at a flow rate of 10 mL/min, yielding compounds **18** (*t*_R_ 55.02 min, 2.0 mg), **8** (*t*_R_ 85.84 min, 36.7 mg), and** 7** (*t*_R_ 86.84 min, 30.0 mg). Subfraction F22.2.4 (596.2 mg) was chromatographed over Sephadex LH-20, using a MeOH–CH_2_Cl_2_ (80:20) solvent system, to provide four subfractions (F22.2.4.1–F22.2.4.4). Subfraction F22.2.4.3 (406 mg) was further separated using preparative HPLC (C18, YMC, 21.2 × 250 mm) using a gradient of MeCN–H_2_O (40:60 → 100:0) over 100 min, followed by 100% MeCN for 15 min at a flow rate of 10 mL/min, yielding compounds **12** (*t*_R_ 68.09 min, 4.5 mg), **11** (*t*_R_ 70.60 min, 4.3 mg), **10** (*t*_R_ 76.17 min, 4.0 mg), and **1** (*t*_R_ 98.18 min, 5.2 mg), respectively. Last, subfraction F22.3.3 (902 mg) was repeated subjection to CC over Sephadex LH-20, eluting with MeOH–CH_2_Cl_2_ (80:20) to afford five subfractions (F22.3.3.1–F22.3.3.5). Subfraction F22.3.3.3 (389.6 mg) was purified by analytical HPLC (C18, YMC, 0.4 × 250 mm, gradient 30:70 to 100:0 MeCN–H_2_O in 100 min, followed by 100% MeCN for 15 min, flow rate 1 mL/min) to give 12 subfractions (F22.3.3.3.1–F22.3.3.3.12). Subfraction F22.3.3.3.9 (110.3 mg) was further purified by HPLC (C18, YMC, 0.4 × 250 mm) using a gradient of MeCN–H_2_O (90:10 → 100:0) 60 min followed by 100% MeCN for 20 min at a flow rate 1 mL/min, to give compounds **4** (*t*_R_ 49.75 min, 0.9 mg) and **5** (*t*_R_ 52.56 min, 3.4 mg). Additionally, a peak at *t*_R_ 101.5 min was further purified by analytical HPLC (C18, YMC, 4.6 × 250 mm, gradient 90:10 to 100:0 MeCN–H_2_O, in 60 min, flow rate 1 mL/min) to provide compounds **2** (*t*_R_ 31.3 min, 1.1 mg) and **1** (*t*_R_ 32.3 min, 1.3 mg). Subfraction F22.3.3.4 (419.1 mg) was separated by analytical HPLC (C18, YMC, 4.6 × 250 mm) using a gradient of 40:60 to 100:0 MeCN–H_2_O over 60 min at a flow rate of 1 mL/min, to obtain compound **9** (*t*_R_ 75.1 min, 1.0 mg).

Albiflorene A or 2ʹ-(*ent*-isopimar-8(14),15-dien-19-carbonyl)oxy-4ʹ,5ʹ-diacetoxy-3ʹ-hydroxycyclohex-1ʹ-en-1ʹ-yl methyl benzoate (**1**): A yellow powder; ${\left[\alpha \right]}_{\text{D}}^{23.6}$–25.4 (*c* 0.16, MeOH); UV (MeOH) *λ*_max_ (log *ε*) 229 (0.18) nm; CD (*c* 1.20 mM, MeOH) *λ*_max_ (mdeg) 204 (–33.59), 200 (+ 35.49) nm; IR (ATR) ν_max_ 3432, 2927, 1722, 1451, 1370, 1315, 1266, 1228, 1175, 1109, 1057, 1027, 979, 712 cm^−1^; ^1^H- and ^13^C-NMR data, see Table 1; HRESIMS *m/z* 671.3195 [M+Na]^+^ (calcd for C_38_H_48_O_9_Na, 671.3191, *∆* = 0.60 ppm).

Albiflorene B or 2ʹ-(*ent*-isopimar-8(14),15-dien-19-carbonyl)oxy-3ʹ,5ʹ-diacetoxy-4ʹ-hydroxycyclohex-1ʹ-en-1ʹ-yl methyl benzoate (**2**): A white powder; ${\left[\alpha \right]}_{\text{D}}^{24}$+19.5 (*c* 0.085, MeOH); UV (MeOH) *λ*_max_ (log *ε*) 228 (0.71), 204 (1.01) nm; CD (*c* 0.026 mM, MeOH) *λ*_max_ (mdeg) 238 (–25.39), 199 (+ 61.63) nm; IR (ATR) ν_max_ 3360, 3193, 2921, 2851, 1727, 1659, 1633, 1470, 1450, 1370, 1268, 1231, 1175, 1110, 1026, 712 cm^−1^; ^1^H- and ^13^C-NMR data, see Table 1; HRESIMS *m/z* 671.3190 [M+Na]^+^ (calcd for C_38_H_48_O_9_Na, 671.3191, *∆* = –0.13 ppm).

Albiflorene C or 2ʹ-(abieta-8,11,13-trien-19-carbonyl)oxy-3ʹ,4ʹ,5ʹ-trihydroxycyclohex-1ʹ-en-1ʹ-yl methyl benzoate (**3**): A white powder; ${\left[\alpha \right]}_{\text{D}}^{24}$+20.6 (*c* 0.47, MeOH); UV (MeOH) *λ*_max_ (log *ε*) 221 (0.83), 203 (1.15) nm; CD (*c* 0.74 mM, MeOH) *λ*_max_ (mdeg) 240 (+ 17.28), 229 (–9.94), 203 (+ 17.17) nm; IR (ATR) ν_max_ 3357, 2928, 1717, 1636, 1497, 1451, 1315, 1269, 1243, 1173, 1106, 1070, 968, 822, 710 cm^−1^; ^1^H- and ^13^C-NMR data, see Table 2; HRESIMS *m/z* 563.3007 [M+Na]^+^ (calcd for C_34_H_43_O_7_Na, 563.3003, *∆* = 0.69 ppm).

Albiflorene D or 2ʹ-(abieta-8,11,13-trien-19-carbonyl)oxy-3ʹ-acetoxy-4ʹ,5ʹ-dihydroxycyclohex-1ʹ-en-1ʹ-yl methyl benzoate (**4**): A white powder; ${\left[\alpha \right]}_{\text{D}}^{24}$+108.4 (*c* 0.05, MeOH); UV (MeOH) *λ*_max_ (log *ε*) 219 (0.93) nm; CD (*c* 0.83 mM, MeOH) *λ*_max_ (mdeg) 203 (–12.67), 199 (+ 50.13) nm; IR (ATR) ν_max_ 3421, 2929, 1723, 1452, 1371, 1268, 1241, 1174, 1108, 1037, 712 cm^−1^; ^1^H- and ^13^C-NMR data, see Table 2; HRESIMS *m/z* 627.2916 [M+Na]^+^ (calcd for C_36_H_44_O_8_Na, 627.2928, *∆* = –1.93 ppm).

Albiflorene E or 2ʹ-(abieta-8,11,13-trien-19-carbonyl)oxy-4ʹ-acetoxy-3ʹ,5ʹ-dihydroxycyclohex-1ʹ-en-1ʹ-yl methyl benzoate (**5**): A white powder; ${\left[\alpha \right]}_{\text{D}}^{25}$+10.1 (*c* 0.17, MeOH); UV (MeOH) *λ*_max_ (log *ε*) 220 (0.75), 203 (1.05) nm; CD (*c* 1.41 mM, MeOH) *λ*_max_ (mdeg) 203 (–17.05), 199 (+ 40.89) nm; IR (ATR) ν_max_ 3360, 3192, 2920, 2851, 1723, 1659, 1633, 1470, 1424, 1377, 1269, 1243, 1173, 1108, 1036, 970, 821, 711 cm^−1^; ^1^H- and ^13^C-NMR data, see Table 2; HRESIMS *m/z* 627.2931 [M+Na]^+^ (calcd for C_36_H_44_O_8_Na, 627.2928, *∆* = 0.43 ppm).

Albiflorene F or 2ʹ-(abieta-8,11,13-trien-19-carbonyl)oxy-5ʹ-acetoxy-3ʹ,4ʹ-dihydroxycyclohex-1ʹ-en-1ʹ-yl methyl benzoate (**6**): A white powder; ${\left[\alpha \right]}_{\text{D}}^{24}$+2.4 (*c* 0.14, MeOH); UV (MeOH) *λ*_max_ (log *ε*) 221 (0.61), 203 (0.89) nm; CD (*c* 0.83 mM, MeOH) *λ*_max_ (mdeg) 203 (–25.33) nm; IR (ATR) ν_max_ 3364, 2927, 2101, 1725, 1636, 1456, 1370, 1269, 1233, 1171, 1107, 1026, 971, 822, 712 cm^−1^; ^1^H- and ^13^C-NMR data, see Table 2; HRESIMS *m/z* 627.2935 [M+Na]^+^ (calcd for C_36_H_44_O_8_Na, 627.2928, *∆* = 0.98 ppm).

Albiflorene G or 2ʹ-(abieta-8,11,13-trien-19-carbonyl)oxy-4ʹ,5ʹ-diacetoxy-3ʹ-hydroxycyclohex-1ʹ-en-1ʹ-yl methyl benzoate (**7**): A yellow powder; ${\left[\alpha \right]}_{\text{D}}^{24}$ –7.6 (*c* 2.85, MeOH); UV (MeOH) *λ*_max_ (log *ε*) 221 (0.43), 203 (0.72) nm; CD (*c* 0.62 mM, MeOH) *λ*_max_ (mdeg) 249 (+ 18.75), 200 (+ 33.67) nm; IR (ATR) ν_max_ 3444, 2928, 1723, 1451, 1370, 1269, 1237, 1173, 1107, 1070, 1026, 970, 823, 712 cm^−1^; ^1^H- and ^13^C-NMR data, see Table 2; HRESIMS *m/z* 669.3051 [M+Na]^+^ (calcd for C_38_H_46_O_9_Na, 669.3034, *∆* = 2.46 ppm).

Albiflorene H or 2ʹ-(abieta-8,11,13-trien-19-carbonyl)oxy-3ʹ,5ʹ-diacetoxy-4ʹ-hydroxycyclohex-1ʹ-en-1ʹ-yl methyl benzoate (**8**): A yellow powder; ${\left[\alpha \right]}_{\text{D}}^{24}$ –3.9 (*c* 0.15, MeOH); UV (MeOH) *λ*_max_ (log *ε*) 221 (0.67), 203 (0.94) nm; CD (*c* 2.32 mM, MeOH) *λ*_max_ (mdeg) 201 (+ 105.87) nm; IR (ATR) ν_max_ 3405, 2937, 1724, 1451, 1370, 1266, 1228, 1170, 1119, 1107, 1026, 712 cm^−1^; ^1^H- and ^13^C-NMR data, see Table 3; HRESIMS *m/z* 647.3196 [M + H]^+^ (calcd for C_38_H_47_O_9_, 647.3215, *∆* = –2.81 ppm).

Albiflorene I or 2ʹ-(abieta-8,11,13-trien-19-carbonyl)oxy-5ʹ-benzoyl-1ʹ,6ʹ-dihydroxycyclohex-3ʹ-en-1ʹ-yl methyl benzoate (**9**): A yellow powder; ${\left[\alpha \right]}_{\text{D}}^{26}$+38.4 (*c* 0.24, MeOH); UV (MeOH) *λ*_max_ (log *ε*) 226 (0.24) nm; CD (*c* 0.90 mM, MeOH) *λ*_max_ (mdeg) 231 (–5.08), 205 (+ 30.76), 198 (+ 14.47) nm; IR (ATR) ν_max_ 3462, 2925, 2854, 1720, 1602, 1451, 1380, 1316, 1269, 1176, 1110, 1070, 1027, 970, 710 cm^−1^; ^1^H- and ^13^C-NMR data, see Table 3; HRESIMS *m/z* 689.3071 [M+Na]^+^ (calcd for C_41_H_46_O_8_Na, 689.3085, *∆* = –2.06 ppm).

Albiflorene J or 2ʹ-(abieta-8,11,13-trien-19-carbonyl)oxy-6ʹ-acetoxy-5ʹ-benzoyl-1ʹ,3ʹ,4ʹ-trihydroxycyclohexane-1ʹ-yl methyl benzoate (**10**): A yellow powder; ${\left[\alpha \right]}_{\text{D}}^{24}$+21.6 (*c* 0.13, MeOH); UV (MeOH) *λ*_max_ (log *ε*) 225 (0.37) nm; CD (*c* 0.69 mM, MeOH) *λ*_max_ (mdeg) 246 (–20.41), 199 (+ 16.48) nm; IR (ATR) ν_max_ 3443, 2953, 2927, 1723, 1602, 1496, 1451, 1377, 1268, 1225, 1177, 1105, 1070, 1027, 971, 905, 822, 710 cm^−1^; ^1^H- and ^13^C-NMR data, see Table 3; HRESIMS *m/z* 743.3426 [M+Na]^+^ (calcd for C_43_H_51_O_11_Na, 743.3426, *∆* = 0.0 ppm).

Albiflorene K or 4ʹ-(abieta-8,11,13-trien-19-carbonyl)oxy-3ʹ-acetoxy-1ʹ,2ʹ,5ʹ,6ʹ-tetrahydroxycyclohexane-1ʹ-yl methyl benzoate (**11**): A yellow powder; ${\left[\alpha \right]}_{\text{D}}^{26}$+13.3 (*c* 0.22, MeOH); UV (MeOH) *λ*_max_ (log *ε*) 220 (0.29), 203 (0.47) nm; CD (*c* 0.78 mM, MeOH) *λ*_max_ (mdeg) 203 (+ 16.51), 199 (–15.92), 193 (+ 10.91) nm; IR (ATR) ν_max_ 3452, 2956, 2931, 1724, 1451, 1369, 1267, 1241, 1175, 1119, 1051, 1037, 972, 822, 712 cm^−1^; ^1^H- and ^13^C-NMR data, see Table 3; HRESIMS *m/z* 661.2999 [M+Na]^+^ (calcd for C_36_H_46_O_10_Na, 661.2983, *∆* = 2.34 ppm).

Albiflorene L or 4ʹ-(abieta-8,11,13-trien-19-carbonyl)oxy-2ʹ-acetoxy-1ʹ,3ʹ,5ʹ,6ʹ-tetrahydroxycyclohexane-1ʹ-yl methyl benzoate (**12**): A yellow powder; ${\left[\alpha \right]}_{\text{D}}^{24}$–12.7 (*c* 0.07, MeOH); UV (MeOH) *λ*_max_ (log *ε*) 221 (0.43), 203 (0.64) nm; CD (*c* 1.09 mM, MeOH) *λ*_max_ (mdeg) 201 (+ 47.95) nm; IR (ATR) ν_max_ 3422, 2926, 1723, 1665, 1602, 1451, 1379, 1314, 1268, 1245, 1175, 1110, 1069, 1026, 971, 821, 710 cm^−1^; ^1^H- and ^13^C-NMR data, see Table 3; HRESIMS *m/z* 661.2984 [M+Na]^+^ (calcd for C_36_H_46_O_10_Na, 661.2983, *∆* = 0.12 ppm).

### Calculation method

Conformer–Rotamer Ensemble Sampling Tool (CREST) with iMTD-GC conformational search algorithm and GBSA solvent model of methanol was used to examine a conformational ensemble. Gaussian 16 Rev. C.01 program was used to execute all DFT calculations^39,40^. The low-energy conformers within an energy window of 5 kcal/mol were further optimized at ωB97XD/cc-PVDZ level of theory. To confirm the true minimum of electronic potential energy of all optimized conformers, the vibrational frequencies at the same level were calculated, and no imaginary frequency was detected. Each conformer with the population over 2% based on Boltzmann distribution with Gibbs free energies were subject to following ECD calculations. TD-DFT at M06-2x/def2-SVP level of theory with IEFPCM of methanol solvent model with 30 excited states for each conformer was used to calculate simulated ECD spectra of compounds **2**, **3**, **6**, **8**, and** 13**^41–43^. The simulated ECD curves of Boltzmann average for all conformers were plotted with overlapping Gaussian function with an exponential half-width (σ = 0.35) by using SpecDis^44,45^. The direct inversion of simulated ECD spectra were plotted for the related enantiomers. All structures were virtualized by using CYLview^46^.

### Antimicrobial measurements

Eight strains from the American Type Culture Collection (ATCC) were used in susceptibility tests for this study. These were four species of Gram-positive (*Staphylococcus aureus* ATCC6538, *Enterococcus faecalis* ATCC19433*, Staphylococcus epidermidis* ATCC12228*, and Bacillus cereus* ATCC11778) and four species of Gram-negative bacteria (*Salmonella* Typhimurium ATCC13311*, Escherichia coli* ATCC8739, *Shigella flexneri* ATCC12022, and *Pseudomonas aeruginosa* ATCC27853). The susceptibility test of compounds was performed in adherence to the CLIS (Clinical and Laboratory Standards Institute) M100 protocol^47^. The tested bacteria were cultured overnight in an incubator for 18–24 h at 37 ºC on Mueller–Hinton agar (MHA). The harvested bacterial colonies were then placed in sterile normal saline and adjusted to 1 × 10^4^ CFU/mL in Mueller–Hinton broth (MHB). The compounds were present in concentrations ranging from 0.097 to 200 µg/mL. The MICs (minimum inhibitory concentrations) and MBCs (minimum bactericidal concentration) values were determined in triplicate.

## Conclusions

In summary, twelve previously undescribed polyoxygenated cyclohexane diterpene esters have been isolated from *K. Albiflora*. This report represents the first documentation of polyoxygenated cyclohexene diterpene esters in *K. albiflora*, signifying its chemotaxonomic significance. The present phytochemical findings could contribute to the categorization of the genus within *Kaempferia* plants and other related genera in Zingiberaceae family. Albiflorene C demonstrated notable antibacterial effectiveness in inhibiting *B. cereus*. The results from this study provide valuable insights into the chemical constituents present in *K. albiflora* and their antimicrobial characteristics, offering potential for the research and development into practical applications.

### Supplementary Information

Supplementary Information.

## Data Availability

The data that support the findings of this study are available in the Supplementary Information. These included 1D- and 2D-NMR, HRESIMS, CD, and IR spectra of compounds **1–14**, ^1^H- and ^13^C-NMR, and HRESIMS of compounds **15–21**, the results of antimicrobial activities of crude extracts and isolated compounds, and Simulated and calculated ECD spectra of compound **13**.

## References

[1] Techaprasan J, Klinbunga S, Ngamriabsakul C, Jenjittikul T (2010). Genetic variation of *Kaempferia* (Zingiberaceae) in Thailand based on chloroplast DNA (*psbA-trnH* and *petA-psbJ*) Sequences. Genet. Mol. Res..

[2] Singh A (2023). The industrially important genus *Kaempferia*: An ethnopharmacological review. Front. Pharmacol..

[3] Van Anh C, Duc DX, Son NT (2023). *Kaempferia* diterpenoids and flavonoids: An overview on phytochemistry, biosynthesis, synthesis, pharmacology, and pharmacokinetics. Med. Chem. Res..

[4] Yenjai C, Prasanphen K, Daodee S, Wongpanich V, Kittakoop P (2004). Bioactive flavonoids from *Kaempferia parviflora*. Fitoterapia.

[5] Thongnest S, Mahidol C, Sutthivaiyakit S, Ruchirawat S (2005). Oxygenated pimarane diterpenoids from *Kaempferia marginata*. J. Nat. Prod..

[6] Boonsombat J (2017). Roscotanes and roscoranes: Oxygenated abietane and pimarane diterpenoids from *Kaempferia roscoeana*. Phytochemistry.

[7] Booranaseensuntorn P (2022). Diterpenoids an p-methoxycinnamic acid diol ester from *Kaempferia saraburiensis* Picheans. (Zingiberaceae): Structural assignment of saraburol and their biological activities. Phytochemistry.

[8] Pancharoen O, Tuntiwachwuttikul P, Taylor WC (1989). Cyclohexene oxide derivatives from *Kaempferia angustifolia* and *Kaempferia* species. Phytochemistry.

[9] Lallo S, Lee S, Dibwe DF, Tezuka Y, Morita H (2014). A new polyoxygenated cyclohexane and other constituents from *Kaempferia rotunda* and their cytotoxic activity. Nat. Prod. Res..

[10] Wisetsai A (2023). Isopimarane-type diterpenoids from the rhizomes of *Kaempferia galanga* L. and their biological activities. Nat. Prod. Res..

[11] Win NN, Hardianti B, Ngwe H, Hayakawa Y, Morita H (2020). Anti-inflammatory activities of isopimara-8(9),15-diene diterpenoids and mode of action of kaempulchraols B-D from *Kaempferia pulchra* rhizomes. J. Nat Med..

[12] Win NN (2020). Anti-inflammatory activities of isopimara-8(14),-15-diene diterpenoids and mode of action of kaempulchraols P and Q from *Kaempferia pulchra* rhizomes. Bioorg. Med. Chem. Lett..

[13] Chokchaisiri R (2022). Marginaols G-M, anti-inflammatory isopimarane diterpenoids, from the rhizomes of *Kaempferia marginata*. Phytochemistry.

[14] Jongsomjainuk O (2023). Kaemtakols A-D, highly oxidized pimarane diterpenoids with potent anti-inflammatory activity from *Kaemferia takensis*. Nat. Prod. Bioprospect..

[15] Kongwaen P (2023). Cytotoxic isopimarane diterpenoids from *Kaempferia koratensis* rhizomes. Rev. Bras. Farmacogn..

[16] Leong-Škorničková J, Newman M, Leong-Škorničková J, Newman M (2015). Kaempferia L. Gingers of Cambodia.

[17] Kam YK (1980). Taxonomic studies in the genus *Kaempferia* (Zingiberaceae). Notes RBG Edinb..

[18] Boonma T, Saensouk S, Saensouk P (2022). *Kaempferia sipraiana* (Zingiberaceae), a new species from Thailand and a new record of *Kaempferia pseudoparviflora* for Myanmar. Biodiversitas..

[19] Jenjittikul T, Ruchisansakun S (2020). *Kaempferia albiflora* (Zingiberaceae), a new species from Thailand. Kew. Bull..

[20] Sugimoto N (2006). Identification of the main constituents in sandarac resin, a natural gum base. J. Food Hygiene Soc. Jpn..

[21] Liu XT (2007). Antibacterial diterpenoids from *Sagittaria pygmaea*. Planta Med..

[22] Singh J, Dhar KL, Atal CK (1997). Studies on the genus Piper-X. Structure of pipoxide. A new cyclohexene epoxide from *P. hookeri* Linn. Tetrahedron.

[23] Carless HAJ, Busia K, Dove Y, Malik SS (1993). Syntheses of conduritol D derivatives from aromatic compounds. J. Chem. Soc. Perkin Trans..

[24] De Carvalho MS, Baptistella LHB, Imamura PM (2008). ^13^C and ^1^H NMR signal assignments of some new synthetic dehydroabietic acid derivatives. Magn. Reson. Chem..

[25] Imhoff JE, Sun M, Wiese J, Tank M, Zeeck A (2018). First evidence of dehydroabietic acid production by a marine phototrophic gammaproteobacterium, the purple sulfur bacterium *Allochromatium vinosum* MT86. Mar. Drugs.

[26] Kroutil W, Hagmann L, Schuez TC, Jungmann V, Pachlatko JP (2005). Esterification of streptol—A cyclitol derivative–by *Candida rugosa* lipase: Influence of the acyl donor on regioselectivity. J. Mol. Catal. B..

[27] Kijjoa A (2002). Polyoxygenated cyclohexene derivatives from *Ellipeiopsis cherrevensis*. Phytochemistry.

[28] Stevenson P, Veitch NC, Simmonds MS (2007). Polyoxygenated cyclohexane derivatives and other constituents from *Kaempferia rotunda* L. Phytochemistry.

[29] Nyandoro SS (2017). Polyoxygenated cyclohexenes and other constituents of *Cleistochlamys kirkii* leaves. J. Nat. Prod..

[30] Kupchan SM, Hemingway RJ, Smith RM (1969). Tumor inhibitors. XLV. Crotepoxide, a novel cyclohexane diepoxide tumor inhibitor from *Croton macrostachys*. J. Org. Chem..

[31] González MA, Pérez-Guaita D, Correa-Royero J, Zapata B (2010). Synthesis and biological evaluation of dehydroabietic acid derivatives. Eur. J. Med. Chem..

[32] Lee CK, Fang JM, Cheng YS (1995). Norditerpenes from *Juniperus chinensis*. Phytochemistry.

[33] Ulubelen A, Topcu G (1992). Abietane diterpenoids from *Salvia pomifera*. Phytochemistry.

[34] Tuntiwachwuttikul P (1987). Constituents of the Zingiberaceae. XI Structues of (+)-(1R,2S,3R,4S)-2-benzoyloxy-methylcyclohex-5-ene-1,2,3,4-tetrol 4-benzoate [(+)-zeylenol] and (+)-(1R,2R,4R,5S,6R,7R)-4-benzoyloxymethyl-3,8-dioxatricyclo-[5.1.0.0^2,4^]octane-5,6-diol 5-acetate 6-benzoate (Boesenboxide) isolated from a new *Boesenbergia* species. Aust. J. Chem..

[35] Kodpinid M, Sadavongvivad C, Thebtaranonth C, Thebtaranonth Y (1983). Structures of *β*-senepoxide, tingtanoxide, and their diene precursors. Constituents of *Uvaria ferruginea*. Tetrahedron. Lett..

[36] Nguyen NL (2020). Bioassay-guided isolation and HPLC quantification of antiproliferative metabolites from *Stahlianthus thorelii*. Molecules.

[37] Dupont MP, Llabrés G, Delaude C, Tchissambou L, Gastmans JP (1997). Sterolic and triterpenoidic constituents of stem bark of *Drypetes gossweileri*. Planta Med..

[38] Radhakrishnan N, Gnanamani A, Mandal AB (2011). A potential antibacterial agent Embelin, a natural benzoquinone extracted from *Embelia ribes*. Biol. Med..

[39] Frisch, M. J. *et al.* Gaussian 16, Revision C.01, (Gaussian, Inc., 2016).

[40] Dennington, R., Keith Todd, A. & Millam John, M. GaussView, Version 6.1. (Semichem Inc., 2016).

[41] Zhao Y, Truhlar DG (2008). The M06 suite of density functionals for main group thermochemistry, thermochemical kinetics, noncovalent interactions, excited states, and transition elements: Two new functionals and systematic testing of four M06-class functionals and 12 other functionals. Theor. Chem. Acc..

[42] Weigend F (2006). Accurate Coulomb-fitting basis sets for H to Rn. Phys. Chem. Chem. Phys..

[43] Weigend F, Ahlrichs R (2005). Balanced basis sets of split valence, triple zeta valence and quadruple zeta valence quality for H to Rn: Design and assessment of accuracy. Phys. Chem. Chem. Phys..

[44] Bruhn T, Schaumlöffel A, Hemberger Y, Bringmann G (2013). SpecDis: Quantifying the comparison of calculated and experimental electronic circular dichroisom spectra. Chirality.

[45] Bruhn, T., Schaumlöffel, A., Hemberger, Y. & Pescitelli, G. SpecDis—A tool to compare calculated and experimental (chir) optical spectra, http://specdis-software.jimdo.com (2017). 10.1002/chir.22138 23532998

[46] Legault, C. Y. CYLview20 (Université de Sherbrooke, 2020). http://www.cylview.org.

[47] *M100 Performance Standards for Antimicrobial Susceptibility Testing, 32th Edition* (Clinical and Laboratory Standards Institute, 2022).

