# Supplementary material for: Albiflorenes A–L, polyoxygenated cyclohex(a/e)ne diterpene esters from Kaempferia albiflora
Source: Sci Rep. 2024 Jun 17;14:13967. doi: 10.1038/s41598-024-64889-6 (PMC11182770; doi:10.1038/s41598-024-64889-6)
Supplement: Supplementary file 1 — Supplementary Information. [file 41598_2024_64889_MOESM1_ESM.pdf]

Supplementary data

Albiflorenes A–L, Polyoxygenated cyclohex(a/e)ne diterpene esters from *Kaempferia albiflora*

Pornpuk Booranaseensuntorn[1], Jutatip Boonsombat[2,3], Sanit Thongnest[2,3]✉, Paratchata Batsomboon[2,3], Nanthawan Reuk-Ngam[2], Panita Khlaychan[2], Saroj Ruchisansakun[4], Prasat Kittakoop[1,3], Supanna Techasakul[2], Chulabhorn Mahidol1[1,2,3] & Somsak Ruchirawat[1,2,3]

[1] Program in Chemical Sciences, Chulabhorn Graduate Institute, Bangkok, Thailand.

[2]Chulabhorn Research Institute, Bangkok, Thailand.

[3]Center of Excellence on Environmental Health and Toxicology (EHT), OPS, MHESI, Thailand.

[4]Department of Plant Science, Faculty of Science, Mahidol University, Bangkok, Thailand.

✉email: sanit@cri.or.th

Supporting Information Contents

**Tables S1**	Antimicrobial properties (MIC, MBC) of crude extracts on the selected microorganisms	5
**Tables S2**	Antimicrobial properties (MIC, MBC, MFC) of some selected compounds on the selected microorganisms.	6
**Tables S3**	Antimicrobial properties (MIC, MBC, MFC) of the standard reference antibiotics (Ciprofloxacin, Vancomycin, Gentamicin, and Amphotericin B).	7
**Figure S1.**	^{1}H NMR (600 MHz) spectrum of compound **1** in $CDCl_3$	8
**Figure S2.**	^{13}C NMR (150 MHz) spectrum of compound **1** in $CDCl_3$	9
**Figure S3.**	DEPT 135 & 90 NMR spectrum of compound **1** in $CDCl_3$	10
**Figure S4.**	HSQC spectrum of compound **1** in $CDCl_3$	11
**Figure S5.**	COSY spectrum of compound **1** in $CDCl_3$	12
**Figure S6.**	HMBC spectrum of compound **1** in $CDCl_3$	13
**Figure S7.**	NOESY spectrum of **1** in $CDCl_3$	14
**Figure S8.**	IR spectrum of compound **1**	15
**Figure S9.**	HRESIMS spectrum of compound **1**	16
**Figure S10.**	CD spectrum of compound **1**	17
**Figure S11.**	UV spectrum of compound **1**	18
**Figure S12.**	^{1}H NMR (600 MHz) spectrum of compound **2** in $CDCl_3$	19
**Figure S13.**	^{13}C NMR (150 MHz) spectrum of **2** in $CDCl_3$	20
**Figure S14.**	DEPT 135 & 90 spectrum of compound **2** in DCl_3	21
**Figure S15.**	HSQC spectrum of compound **2** in $CDCl_3$	22
**Figure S16.**	COSY spectrum of compound **2** in $CDCl_3$	23
**Figure S17.**	HMBC spectrum of compound **2** in $CDCl_3$	24
**Figure S18.**	NOESY spectrum of compound **2** in $CDCl_3$	25
**Figure S19.**	IR spectrum of compound **2**	26
**Figure S20.**	HRESIMS spectrum of compound **2**	27
**Figure S21.**	CD spectrum of compound **2**	28
**Figure S22.**	UV spectrum of compound **2**	29
**Figure S23.**	^{1}H NMR (600 MHz) spectrum of compound **3** in $CDCl_3$	30
**Figure S24.**	^{13}C NMR (150 MHz) spectrum of compound **3** in $CDCl_3$	31
**Figure S25.**	DEPT 135 & 90 NMR spectrum of compound **3** in $CDCl_3$	32
**Figure S26.**	HSQC spectrum of compound **3** in $CDCl_3$	33
**Figure S27.**	COSY spectrum of compound **3** in $CDCl_3$	34
**Figure S28.**	HMBC spectrum of compound **3** in $CDCl_3$	35
**Figure S29.**	NOESY spectrum of compound **3** in $CDCl_3$	36
**Figure S30.**	IR spectrum of compound **3**	37
**Figure S31.**	HRESIMS spectrum of compound **3**	38
**Figure S32.**	CD spectrum of compound **3**	39
**Figure S33.**	UV spectrum of compound **3**	40
**Figure S34.**	^{1}H NMR (600 MHz) spectrum of compound **4** in $CDCl_3$	41
**Figure S35.**	^{13}C NMR (150 MHz) spectrum of compound **4** in $CDCl_3$	42

**Figure S36.**	DEPT 135 & 90 NMR spectrum of compound **4** in $CDCl_3$	43
**Figure S37.**	HSQC spectrum of compound **4** in $CDCl_3$	44
**Figure S38.**	COSY spectrum of compound **4** in $CDCl_3$	45
**Figure S39.**	HMBC spectrum of compound **4** in $CDCl_3$	46
**Figure S40.**	NOESY spectrum of compound **4** in $CDCl_3$	47
**Figure S41.**	IR spectrum of compound **4**	48
**Figure S42.**	HRESIMS spectrum of compound **4**	49
**Figure S43.**	CD spectrum of compound **4**	50
**Figure S44.**	UV spectrum of compound **4**	51
**Figure S45.**	^{1}H NMR (600 MHz) spectrum of compound **5** in $CDCl_3$	52
**Figure S46.**	^{13}C NMR (150 MHz) spectrum of compound **5** in $CDCl_3$	53
**Figure S47.**	DEPT 135 & 90 spectrum of compound **5** in $CDCl_3$	54
**Figure S48.**	HSQC spectrum of compound **5** in $CDCl_3$	55
**Figure S49.**	COSY spectrum of compound **5** in $CDCl_3$	56
**Figure S50.**	HMBC spectrum of compound **5** in $CDCl_3$	57
**Figure S51.**	NOESY spectrum of compound **5** in $CDCl_3$	58
**Figure S52.**	IR spectrum of compound **5**	59
**Figure S53.**	HRESIMS spectrum of compound **5**	60
**Figure S54.**	CD spectrum of compound **5**	61
**Figure S55.**	UV spectrum of compound **5**	62
**Figure S56.**	^{1}H NMR (600 MHz) spectrum of compound **6** in $CDCl_3$	63
**Figure S57.**	^{13}C NMR (150 MHz) spectrum of compound **6** in $CDCl_3$	64
**Figure S58.**	HSQC spectrum of compound **6** in $CDCl_3$	65
**Figure S59.**	COSY spectrum of compound **6** in $CDCl_3$	66
**Figure S60.**	HMBC spectrum of compound **6** in $CDCl_3$	67
**Figure S61.**	NOESY spectrum of compound **6** in $CDCl_3$	68
**Figure S62.**	IR spectrum of compound **6**	69
**Figure S63.**	HRESIMS spectrum of compound **6**	70
**Figure S64.**	CD spectrum of compound **6**	71
**Figure S65.**	UV spectrum of compound **6**	72
**Figure S66.**	^{1}H NMR (600 MHz) spectrum of compound **7** in $CDCl_3$	73
**Figure S67.**	^{13}C NMR (150 MHz) spectrum of compound **7** in $CDCl_3$	74
**Figure S68.**	DEPT 135 & 90 spectrum of compound **7** in $CDCl_3$	75
**Figure S69.**	HSQC spectrum of compound **7** in $CDCl_3$	76
**Figure S70.**	COSY spectrum of compound **7** in $CDCl_3$	77
**Figure S71.**	HMBC spectrum of compound **7** in $CDCl_3$	78
**Figure S72.**	NOESY spectrum of compound **7** in $CDCl_3$	79
**Figure S73.**	IR spectrum of compound **7**	80
**Figure S74.**	HRESIMS spectrum of compound **7**	81
**Figure S75.**	CD spectrum of compound **7**	82
**Figure S76.**	UV spectrum of compound **7**	83
**Figure S77.**	^{1}H NMR (600 MHz) spectrum of compound **8** in $CDCl_3$	84
**Figure S78.**	^{13}C NMR (150 MHz) spectrum of compound **8** in $CDCl_3$	85
**Figure S79.**	DEPT 135 & 90 NMR spectrum of compound **8** in $CDCl_3$	86
**Figure S80.**	HSQC spectrum of compound **8** in $CDCl_3$	87
**Figure S81.**	COSY spectrum of compound **8** in $CDCl_3$	88
**Figure S82.**	HMBC spectrum of compound **8** in $CDCl_3$	89
**Figure S83.**	NOESY spectrum of compound **8** in $CDCl_3$	90
**Figure S84.**	IR spectrum of compound **8**	91
**Figure S85.**	HRESIMS spectrum of compound **8**	92
**Figure S86.**	CD spectrum of compound **8**	93
**Figure S87.**	UV spectrum of compound **8**	94
**Figure S88.**	^{1}H NMR (600 MHz) spectrum of compound **9** in $CDCl_3$	95
**Figure S89.**	^{13}C NMR (150 MHz) spectrum of compound **9** in $CDCl_3$	96
**Figure S90.**	DEPT 135 & 90 spectrum of compound **9** in $CDCl_3$	97
**Figure S91.**	HSQC spectrum of compound **9** in $CDCl_3$	98
**Figure S92.**	COSY spectrum of compound **9** in $CDCl_3$	99
**Figure S93.**	HMBC spectrum of compound **9** in $CDCl_3$	100
**Figure S94.**	NOESY spectrum of compound **9** in $CDCl_3$	101
**Figure S95.**	IR spectrum of compound **9**	102

**Figure S96.**	HRESIMS spectrum of compound **9**	103
**Figure S97.**	CD spectrum of compound **9**	104
**Figure S98.**	UV spectrum of compound **9**	105
**Figure S99.**	^{1}H NMR (600 MHz) spectrum of compound **10** in $CDCl_3$	106
**Figure S100.**	^{13}C NMR (150 MHz) spectrum of compound **10** in $CDCl_3$	107
**Figure S101.**	DEPT 135 & 90 spectrum of compound **10** in $CDCl_3$	108
**Figure S102.**	HSQC spectrum of compound **10** in $CDCl_3$	109
**Figure S103.**	COSY spectrum of compound **10** in $CDCl_3$	110
**Figure S104.**	HMBC spectrum of compound **10** in $CDCl_3$	111
**Figure S105.**	NOESY spectrum of compound **10** in $CDCl_3$	112
**Figure S106.**	IR spectrum of compound **10**	113
**Figure S107.**	HRESIMS spectrum of compound **10**	114
**Figure S108.**	CD spectrum of compound **10**	115
**Figure S109.**	UV spectrum of compound **10**	116
**Figure S110.**	^{1}H NMR (600 MHz) spectrum of compound **11** in $CDCl_3$	117
**Figure S111.**	^{13}C NMR (150 MHz) spectrum of compound **11** in $CDCl_3$	118
**Figure S112.**	DEPT 135 & 90 spectrum of compound **11** in $CDCl_3$	119
**Figure S113.**	HSQC spectrum of compound **11** in $CDCl_3$	120
**Figure S114.**	COSY spectrum of compound **11** in $CDCl_3$	121
**Figure S115.**	HMBC spectrum of compound **11** in $CDCl_3$	122
**Figure S116.**	NOESY spectrum of compound **11** in $CDCl_3$	123
**Figure S117.**	IR spectrum of compound **11**	124
**Figure S118.**	HRESIMS spectrum of compound **11**	125
**Figure S119.**	CD spectrum of compound **11**	126
**Figure S120.**	UV spectrum of compound **11**	127
**Figure S121.**	^{1}H NMR (600 MHz) spectrum of compound **12** in $CDCl_3$	128
**Figure S122.**	^{13}C NMR (150 MHz) spectrum of compound **12** in $CDCl_3$	129
**Figure S123.**	DEPT 135 & 90 spectrum of compound **12** in $CDCl_3$	130
**Figure S124.**	HSQC spectrum of compound **12** in $CDCl_3$	131
**Figure S125.**	COSY spectrum of compound **12** in $CDCl_3$	132
**Figure S126.**	HMBC spectrum of compound **12** in $CDCl_3$	133
**Figure S127.**	NOESY spectrum of compound **12** in $CDCl_3$	134
**Figure S128.**	IR spectrum of compound **12**	135
**Figure S129.**	HRESIMS spectrum of compound **12**	136
**Figure S130.**	CD spectrum of compound **12**	137
**Figure S131.**	UV spectrum of compound **12**	138
**Figure S132.**	^{1}H NMR (600 MHz) spectrum of compound **13** in $CDCl_3$	139
**Figure S133.**	^{13}C NMR (150 MHz) spectrum of compound **13** in $CDCl_3$	140
**Figure S134.**	DEPT 135 & 90 spectrum of compound **13** in $CDCl_3$	141
**Figure S135.**	HSQC spectrum of compound **13** in $CDCl_3$	142
**Figure S136.**	COSY spectrum of compound **13** in $CDCl_3$	143
**Figure S137.**	HMBC spectrum of compound **13** in $CDCl_3$	144
**Figure S138.**	NOESY spectrum of compound **13** in $CDCl_3$	145
**Figure S139.**	IR spectrum of compound **13**	146
**Figure S140.**	HRESIMS spectrum of compound **13**	147
**Figure S141.**	CD spectrum of compound **13**	148
**Figure S142.**	UV spectrum of compound **13**	149
**Figure S143.**	^{1}H NMR (600 MHz) spectrum of compound **14** in $CDCl_3$	150
**Figure S144.**	^{13}C NMR (150 MHz) spectrum of compound **14** in $CDCl_3$	151
**Figure S145.**	DEPT 135 & 90 spectrum of compound **14** in $CDCl_3$	152
**Figure S146.**	HSQC spectrum of compound **14** in $CDCl_3$	153
**Figure S147.**	COSY spectrum of compound **14** in $CDCl_3$	154
**Figure S148.**	HMBC spectrum of compound **14** in $CDCl_3$	155
**Figure S149.**	NOESY spectrum of compound **14** in $CDCl_3$	156
**Figure S150.**	IR spectrum of compound **14**	157
**Figure S151.**	HRESIMS spectrum of compound **14**	158
**Figure S152.**	CD spectrum of compound **14**	159
**Figure S153.**	UV spectrum of compound **14**	160
**Figure S154.**	^{1}H NMR (600 MHz) spectrum of compound **15** in $CDCl_3$	161
**Figure S155.**	^{13}C NMR (150 MHz) spectrum of compound **15** in $CDCl_3$	162

**Figure S156.**	HRESIMS spectrum of compound **15**	163
**Figure S157.**	^{1}H NMR (600 MHz) spectrum of compound **16** in $CDCl_3$	164
**Figure S158.**	^{13}C NMR (150 MHz) spectrum of compound **16** in $CDCl_3$	165
**Figure S159.**	HRESIMS spectrum of compound **16**	166
**Figure S160.**	^{1}H NMR (600 MHz) spectrum of compound **17** in $CDCl_3$	167
**Figure S161.**	^{13}C NMR (150 MHz) spectrum of compound **17** in $CDCl_3$	168
**Figure S162.**	HRESIMS spectrum of compound **17**	169
**Figure S163.**	^{1}H NMR (600 MHz) spectrum of compound **18** in $CDCl_3$	170
**Figure S164.**	^{13}C NMR (150 MHz) spectrum of compound **18** in $CDCl_3$	171
**Figure S165.**	HRESIMS spectrum of compound **18**	172
**Figure S166.**	^{1}H NMR (600 MHz) spectrum of compound **19** in $CDCl_3$	173
**Figure S167.**	^{13}C NMR (150 MHz) spectrum of compound **19** in $CDCl_3$	174
**Figure S168.**	HRESIMS spectrum of compound **19**	175
**Figure S169.**	^{1}H NMR (600 MHz) spectrum of compound **20** in $CDCl_3$	176
**Figure S170.**	^{13}C NMR (150 MHz) spectrum of compound **20** in $CDCl_3$	177
**Figure S171.**	HRESIMS spectrum of compound **20**	178
**Figure S172.**	^{1}H NMR (600 MHz) spectrum of compound **21** in $CDCl_3$	179
**Figure S173.**	^{13}C NMR (150 MHz) spectrum of compound **21** in $CDCl_3$	180
**Figure S174.**	HRESIMS spectrum of compound **21**	181
**Figure S175.**	Simulated and calculated ECD spectra of compound **13**	182
**Tables S4**	Conformational analysis of **2**	183
**Tables S5**	Conformational analysis of **2-dias**	183
**Tables S6**	Conformational analysis of **3**	184
**Tables S7**	Conformational analysis of **3-dias**	184
**Tables S8**	Conformational analysis of **6**	185
**Tables S9**	Conformational analysis of **6-dias**	185
**Tables S10**	Conformational analysis of **8**	186
**Tables S11**	Conformational analysis of **8-dias**	186
**Tables S12**	Coordinates of compound **2**	187
**Tables S13**	Coordinates of compound **2-dias**	197
**Tables S14**	Coordinates of compound **3**	203
**Tables S15**	Coordinates of compound **3-dias**	205
**Tables S16**	Coordinates of compound **6**	213
**Tables S17**	Coordinates of compound **6-dias**	217
**Tables S18**	Coordinates of compound **8**	225
**Tables S19**	Coordinates of compound **8-dias**	231

Table S1. Antimicrobial properties (MIC, MBC) of crude extracts on the selected microorganisms.

	Minimum inhibitory concentration (μg/mL)/Minimum bactericidal concentration (μg/mL)[a]							
**Samples**	*S. aureus*	*S. epidermidis*	*E. faecalis*	*B. cereus*	*P. aeruginosa*	*E. coli*	*S.* Typhimurium	*S. flexneri*
**WH-EtOH**[b]	>8/>8	>8/>8	>8/>8	0.5/>8	>8/>8	>8/>8	>8/>8	>8/>8
**WH-DCM-MeOH**[c]	>8/>8	>8/>8	>8/>8	1/>8	>8/>8	>8/>8	>8/>8	>8/>8

[a] = Each run was repeated twice (n = 2); [b] = The weight of sample was 50 mg; [c] = The weight of sample was 40 mg

Table S2. Antimicrobial properties (MIC, MBC, MFC) of some selected compounds on the selected microorganisms.

	Minimum inhibitory concentration (μg/mL)/Minimum bactericidal concentration (μg/mL)							
**Compounds**	*S. aureus*	*S. epidermidis*	*E. faecalis*	*B. cereus*	*P. aeruginosa*	*E. coli*	*S. Typhimurium*	*S. flexneri*
**1**	>200/>200	>200/>200	>200/>200	>200/>200				
**3**	>200/-	100/200	>200/-	3.125/6.25	>200/-	>200/-	>200/-	>200/-
**6**	>200/-	>200/-	>200/-	>200/-	>200/-	>200/-	>200/-	>200/-
**7**	>200/-	>200/-	>200/-	>200/-	>200/-	>200/-	>200/-	>200/-
**8**	>200/>200	>200/>200	>200/>200	>200/>200	-	-	-	-
**9**	>200/>200	>200/>200	>200/>200	>200/>200	-	-	-	-
**10**	>200/>200	>200/>200	>200/>200	200/>200	-	-	-	-
**11**	200/>200	>200/>200	200/>200	200/>200	-	-	-	-
**12**	200/>200	200/>200	200/>200	200/>200	-	-	-	-
**13**	50/>200	25/50	25/>200	6.25/25	>200/-	>200/-	200/-	>200/-
**14**	100/>200	100/>200	50/>200	25/50	>200/-	>200/-	200/-	>200/-
**15**	>200/>200	>200/>200	>200/>200	200/>200				
**16**	50/>200	100/>200	/>200	/>200	>200/-	>200/-	>200/-	>200/-
**17**	>200/-	>200/-	>200/-	>200/-	>200/-	>200/-	200/-	>200/-
**18**	>200/>200	>200/>200	>200/>200	200/>200	-	-	-	-
**19**	>200/-	>200/-	>200/-	>200/-	>200/-	>200/-	>200/-	>200/-
**20**	>200/-	>200/-	>200/-	>200/-	>200/-	>200/-	>200/-	>200/-
**21**	-	-	-	-	-	-	-	-

n.a: MIC values > 200 µg/ml; -: not tested.

Table S3. Antimicrobial properties (MIC, MBC, MFC) of the standard reference antibiotics (Ciprofloxacin, Vancomycin, Gentamicin, and Amphotericin B).

	Compound (μg/ml)							
	**Ciprofloxacin**		**Vancomycin**		**Gentamicin**		**Amphotericin B**	
**Bacteria**	**MIC**	**MBC**	**MIC**	**MBC**	**MIC**	**MBC**		
S. aureus	0.195	0.195	0.78	1.56	3.125	6.25		
S. epidermidis	0.39	0.39	1.56	3.125	0.78	1.56		
B. cereus	1.56	3.125			12.5	12.5		
E. faecalis	0.39	0.78			0.39	0.78		
P. aeruginosa								
E. coli								
S. Typhimurium								
S. flexneri								

Table S1. Antimicrobial properties (MIC, MBC) of crude extracts on the selected microorganisms.

	Minimum inhibitory concentration (μg/mL)/Minimum bactericidal concentration (μg/mL)[a]							
**Samples**	*S. aureus*	*S. epidermidis*	*E. faecalis*	*B. cereus*	*P. aeruginosa*	*E. coli*	*S.* Typhimurium	*S. flexneri*
**WH-EtOH**[b]	>8/>8	>8/>8	>8/>8	0.5/>8	>8/>8	>8/>8	>8/>8	>8/>8
**WH-DCM-MeOH**[c]	>8/>8	>8/>8	>8/>8	1/>8	>8/>8	>8/>8	>8/>8	>8/>8

[a] = Each run was repeated twice (n = 2); [b] = The weight of sample was 50 mg; [c] = The weight of sample was 40 mg

Table S2. Antimicrobial properties (MIC, MBC, MFC) of some selected compounds on the selected microorganisms.

	Minimum inhibitory concentration (μg/mL)/Minimum bactericidal concentration (μg/mL)							
**Compounds**	*S. aureus*	*S. epidermidis*	*E. faecalis*	*B. cereus*	*P. aeruginosa*	*E. coli*	*S. Typhimurium*	*S. flexneri*
**1**	>200/>200	>200/>200	>200/>200	>200/>200				
**3**	>200/-	100/200	>200/-	3.125/6.25	>200/-	>200/-	>200/-	>200/-
**6**	>200/-	>200/-	>200/-	>200/-	>200/-	>200/-	>200/-	>200/-
**7**	>200/-	>200/-	>200/-	>200/-	>200/-	>200/-	>200/-	>200/-
**8**	>200/>200	>200/>200	>200/>200	>200/>200	-	-	-	-
**9**	>200/>200	>200/>200	>200/>200	>200/>200	-	-	-	-
**10**	>200/>200	>200/>200	>200/>200	200/>200	-	-	-	-
**11**	200/>200	>200/>200	200/>200	200/>200	-	-	-	-
**12**	200/>200	200/>200	200/>200	200/>200	-	-	-	-
**13**	50/>200	25/50	25/>200	6.25/25	>200/-	>200/-	200/-	>200/-
**14**	100/>200	100/>200	50/>200	25/50	>200/-	>200/-	200/-	>200/-
**15**	>200/>200	>200/>200	>200/>200	200/>200				
**16**	50/>200	100/>200	/>200	/>200	>200/-	>200/-	>200/-	>200/-
**17**	>200/-	>200/-	>200/-	>200/-	>200/-	>200/-	200/-	>200/-
**18**	>200/>200	>200/>200	>200/>200	200/>200	-	-	-	-
**19**	>200/-	>200/-	>200/-	>200/-	>200/-	>200/-	>200/-	>200/-
**20**	>200/-	>200/-	>200/-	>200/-	>200/-	>200/-	>200/-	>200/-
**21**	-	-	-	-	-	-	-	-

n.a: MIC values > 200 μg/ml; -: not tested.

Table S3. Antimicrobial properties (MIC, MBC, MFC) of the standard reference antibiotics (Ciprofloxacin, Vancomycin, Gentamicin, and Amphotericin B).

	Compound (µg/ml)							
	Ciprofloxacin		Vancomycin		Gentamicin		Amphotericin B	
**Bacteria**	**MIC**	**MBC**	**MIC**	**MBC**	**MIC**	**MBC**		
S. aureus	0.195	0.195	0.78	1.56	3.125	6.25		
S. epidermidis	0.39	0.39	1.56	3.125	0.78	1.56		
B. cereus	1.56	3.125			12.5	12.5		
E. faecalis	0.39	0.78			0.39	0.78		
P. aeruginosa								
E. coli								
S. Typhimurium								
S. flexneri								

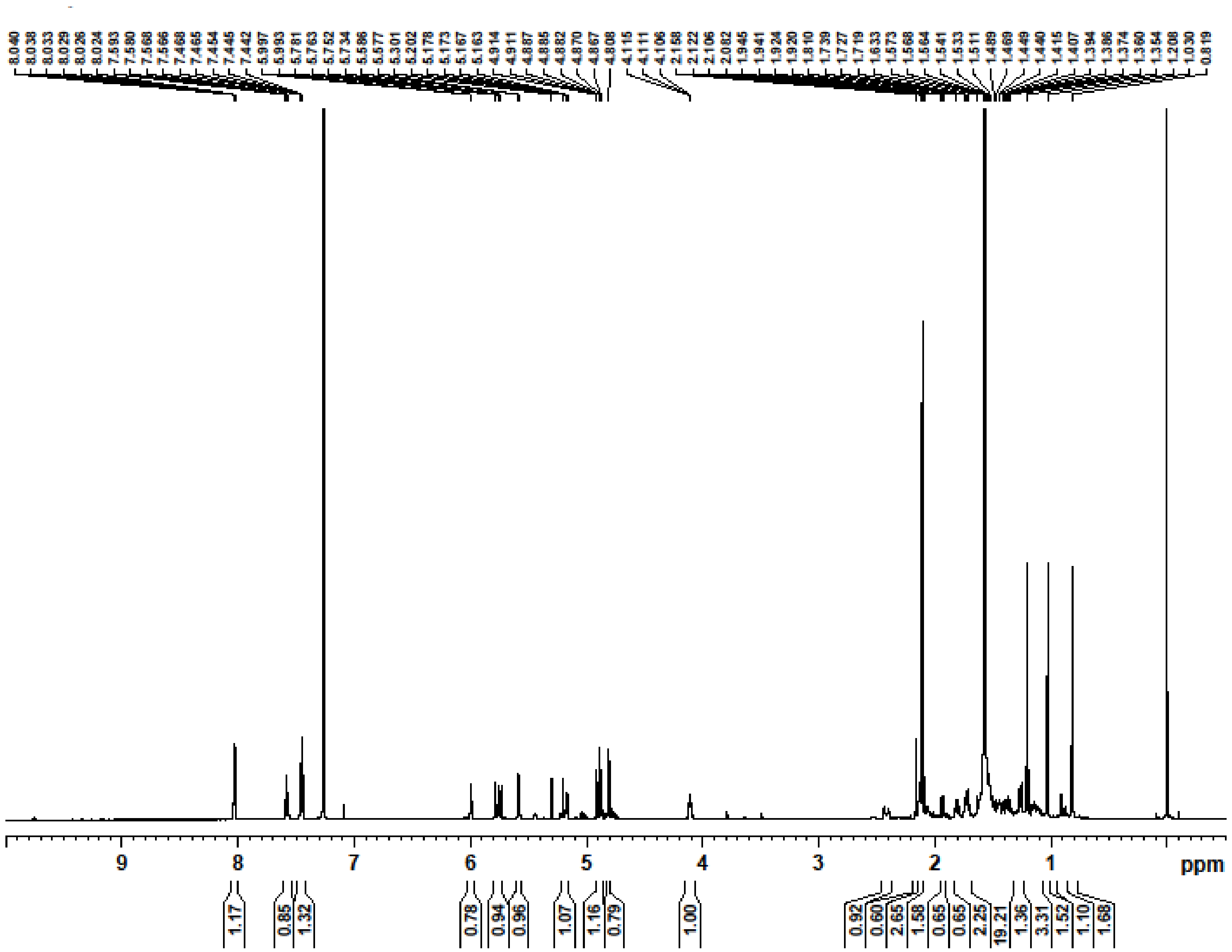

Figure S1. ^1H NMR (600 MHz) spectrum of compound **1** in $CDCl_3$

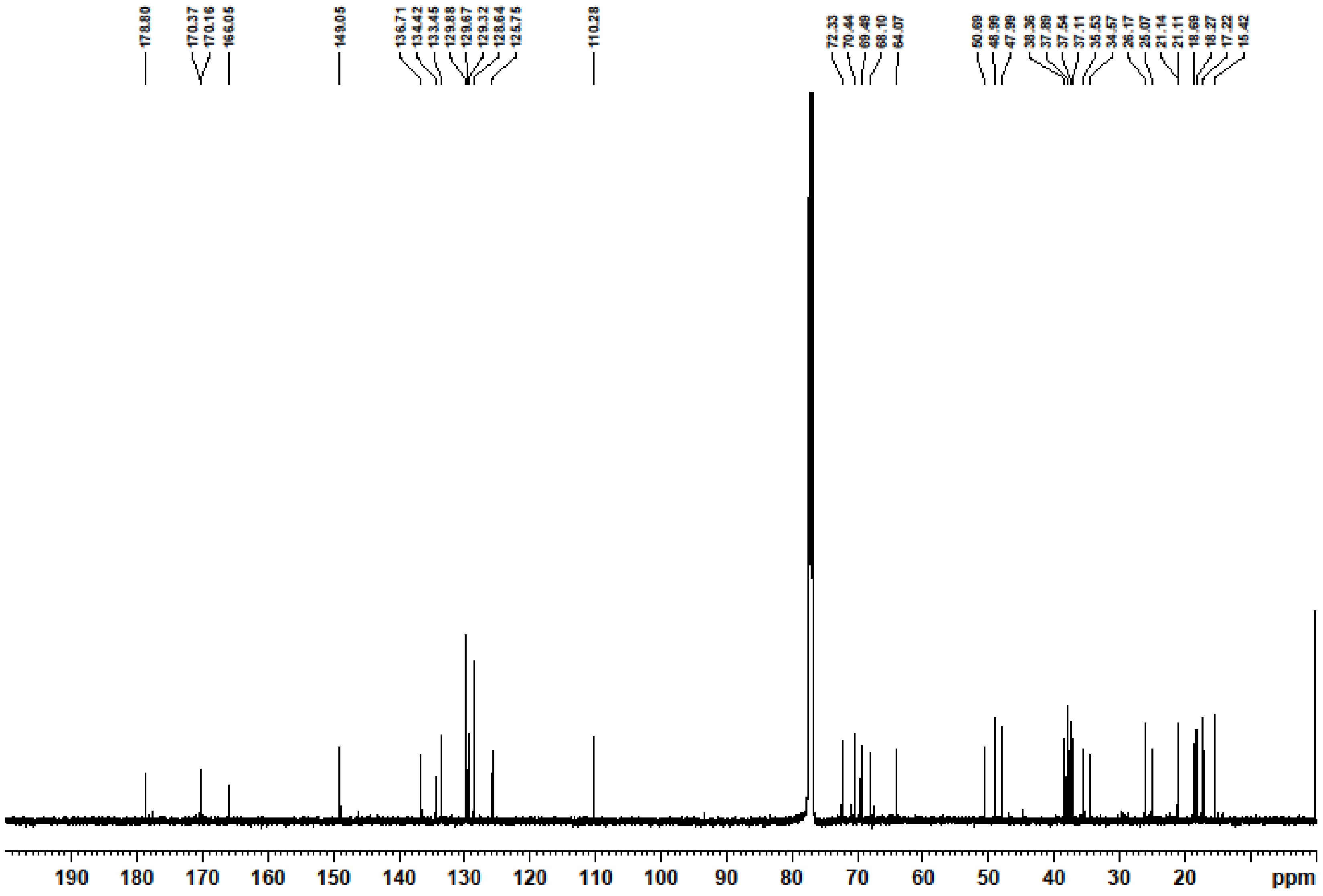

Figure S2. ^{13}C NMR (150 MHz) spectrum of compound **1** in $CDCl_3$

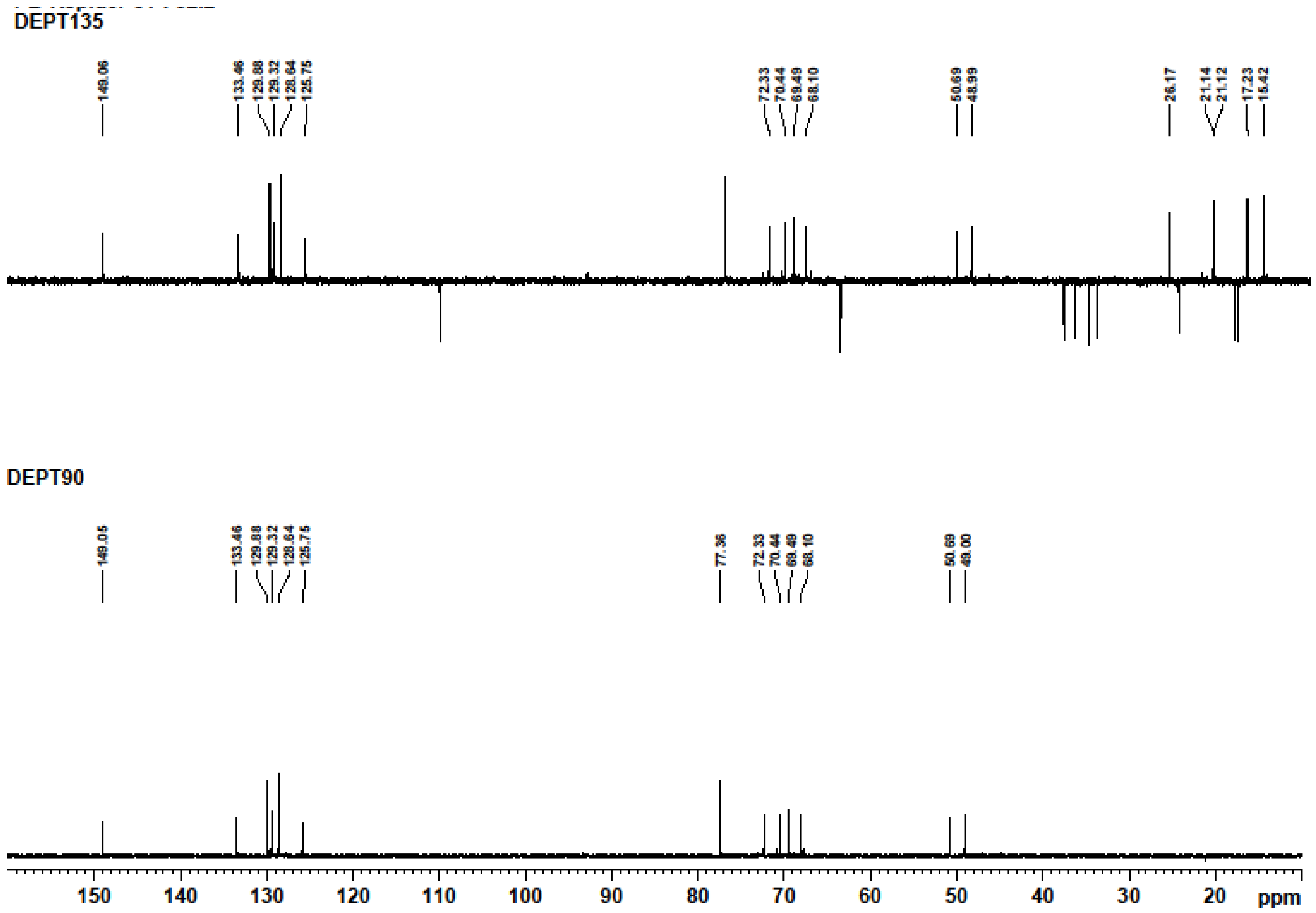

Figure S3. DEPT 135 & 90 NMR spectrum of compound **1** in $CDCl_3$

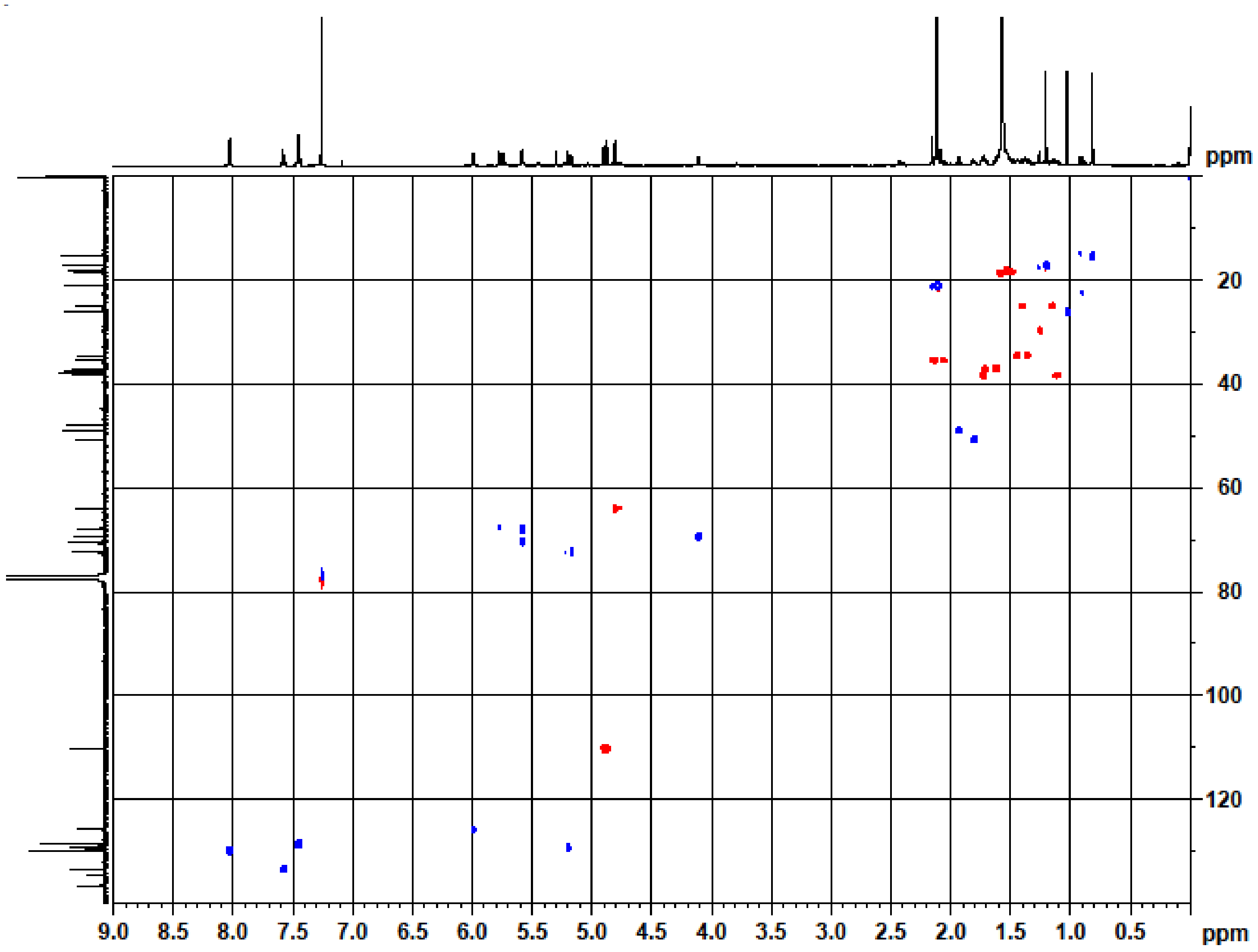

Figure S4. HSQC spectrum of compound **1** in $CDCl_3$

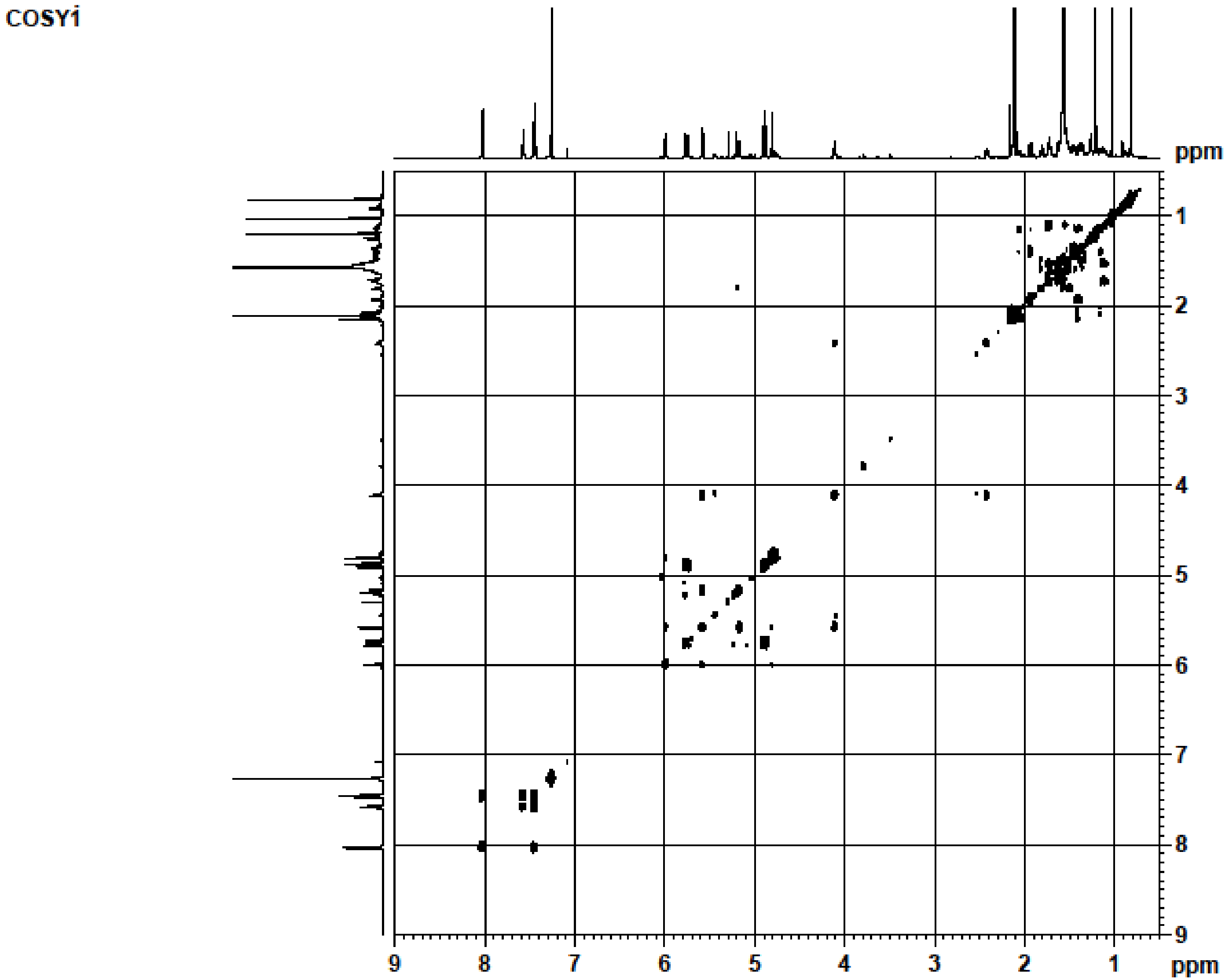

Figure S5. COSY spectrum of compound **1** in $CDCl_3$

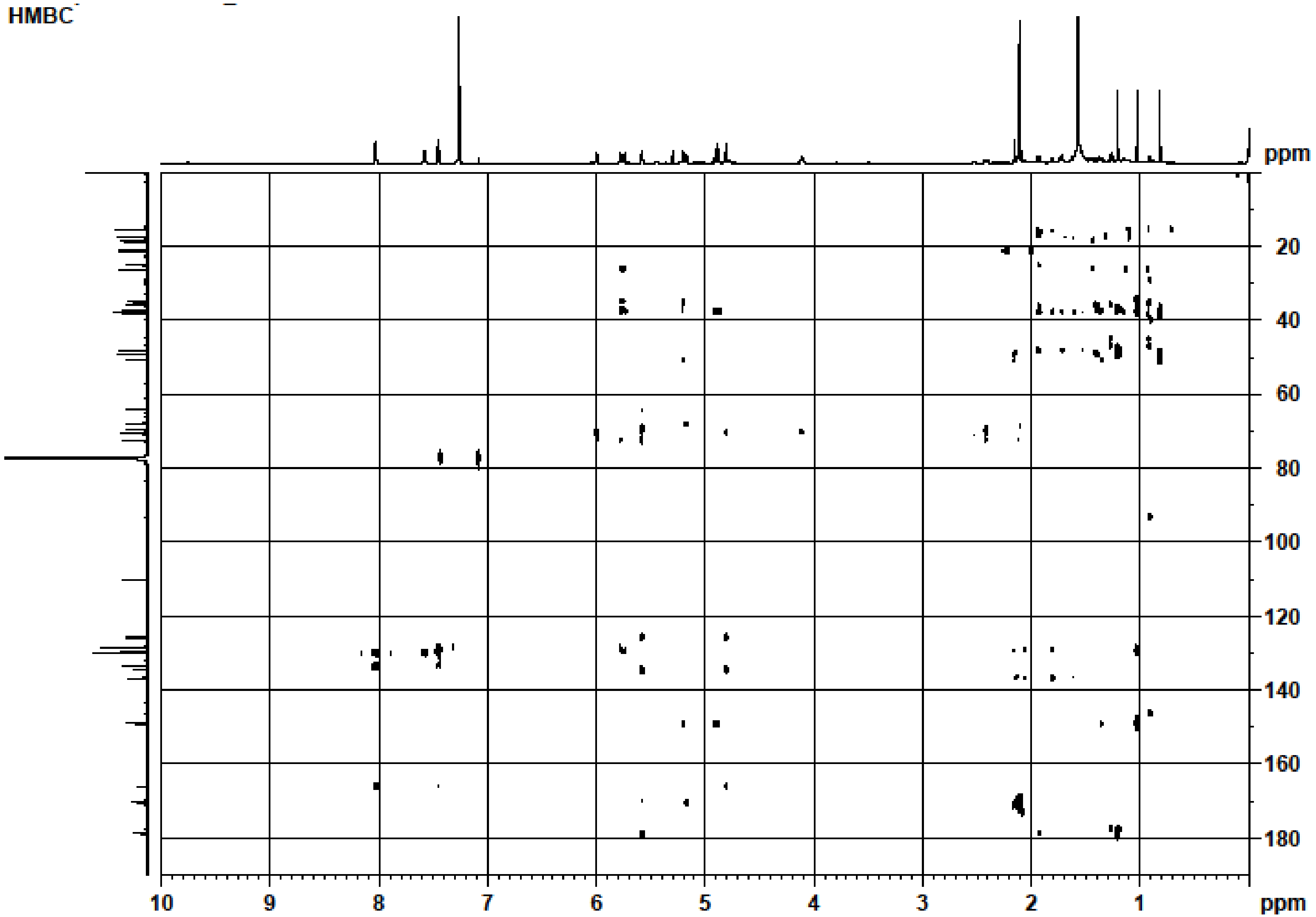

Figure S6. HMBC spectrum of compound **1** in $CDCl_3$

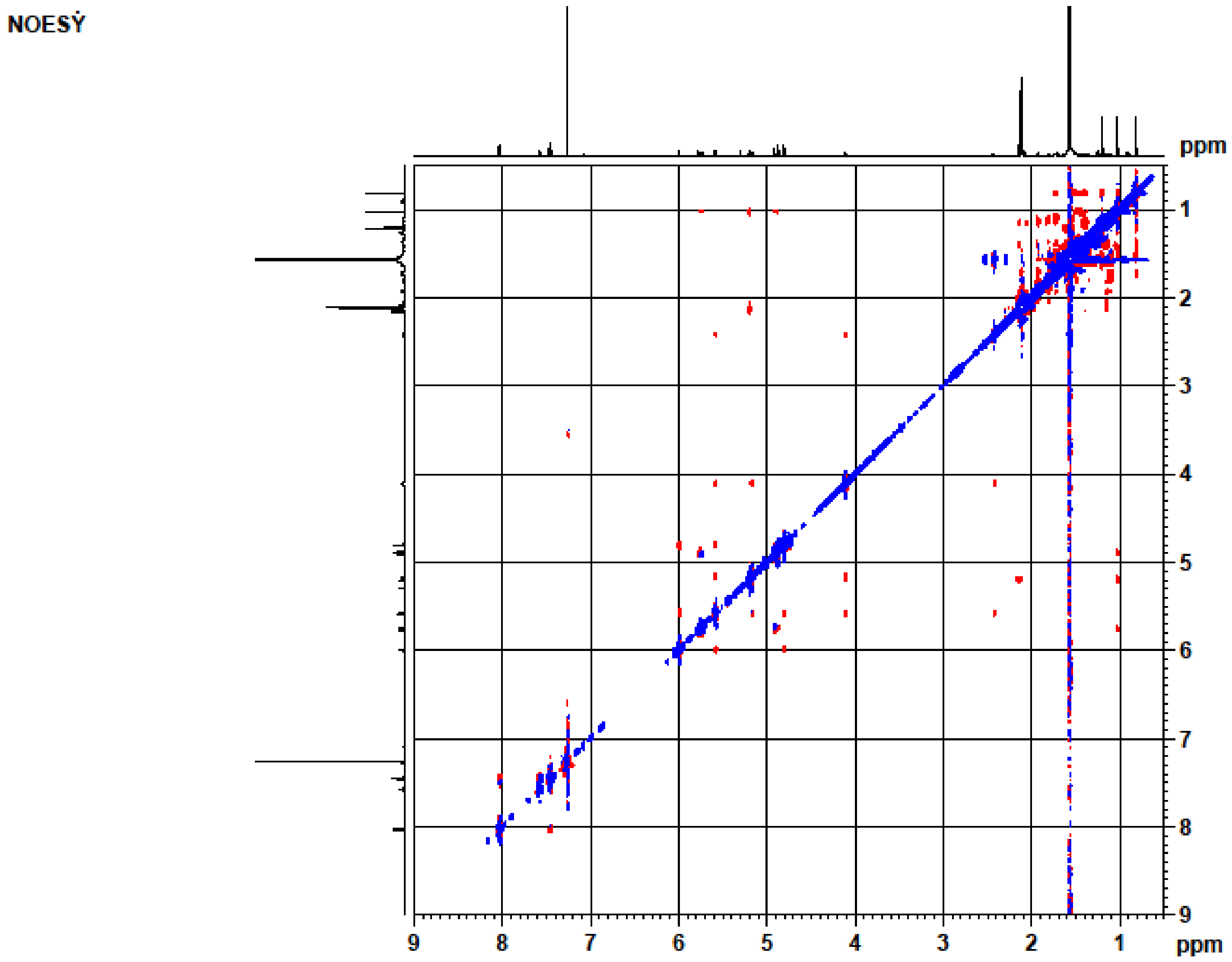

Figure S7. NOESY spectrum of **1** in $CDCl_3$

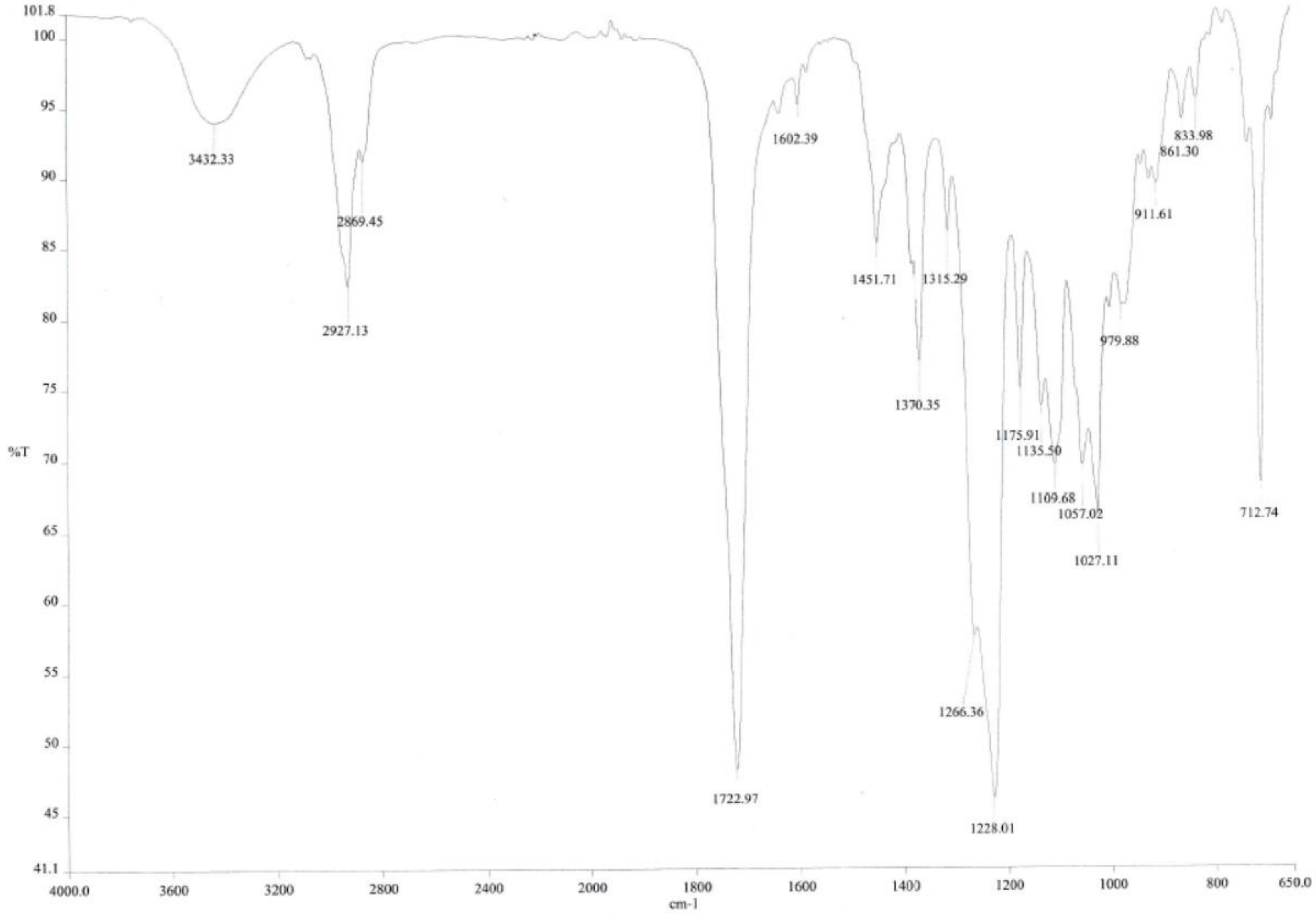

Figure S8. IR spectrum of compound **1**

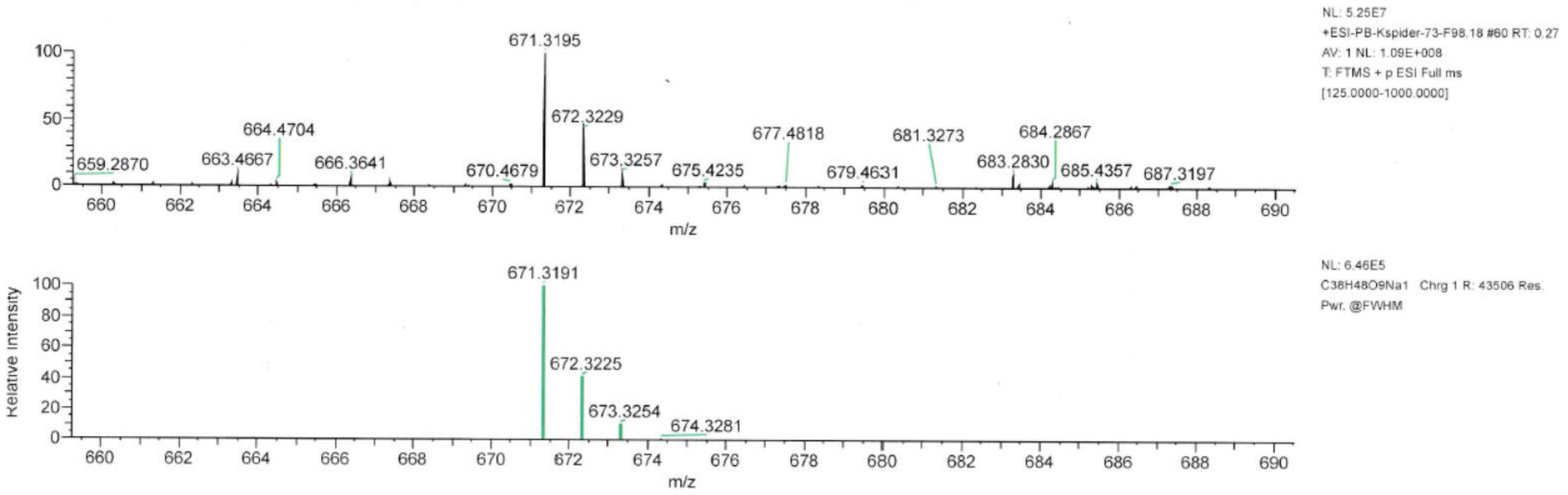

Peak Mass	Display Formula	Delta [ppm]	Theo. mass	MSMS Matched Fragments
671.3195	$C_{38}H_{48}O_9{}^{23}Na$ [M+Na]+	0.60	671.31905	(Collection)
671.3195	$C_{22}H_{55}O_{22}$	2.25	671.31795	(Collection)
671.3195	$C_{40}H_{47}O_9$	-2.98	671.32146	(Collection)
671.3195	$C_{13}H_{60}O_{27}{}^{23}Na$	-2.92	671.32142	(Collection)

Figure S9. HRESIMS spectrum of compound **1**

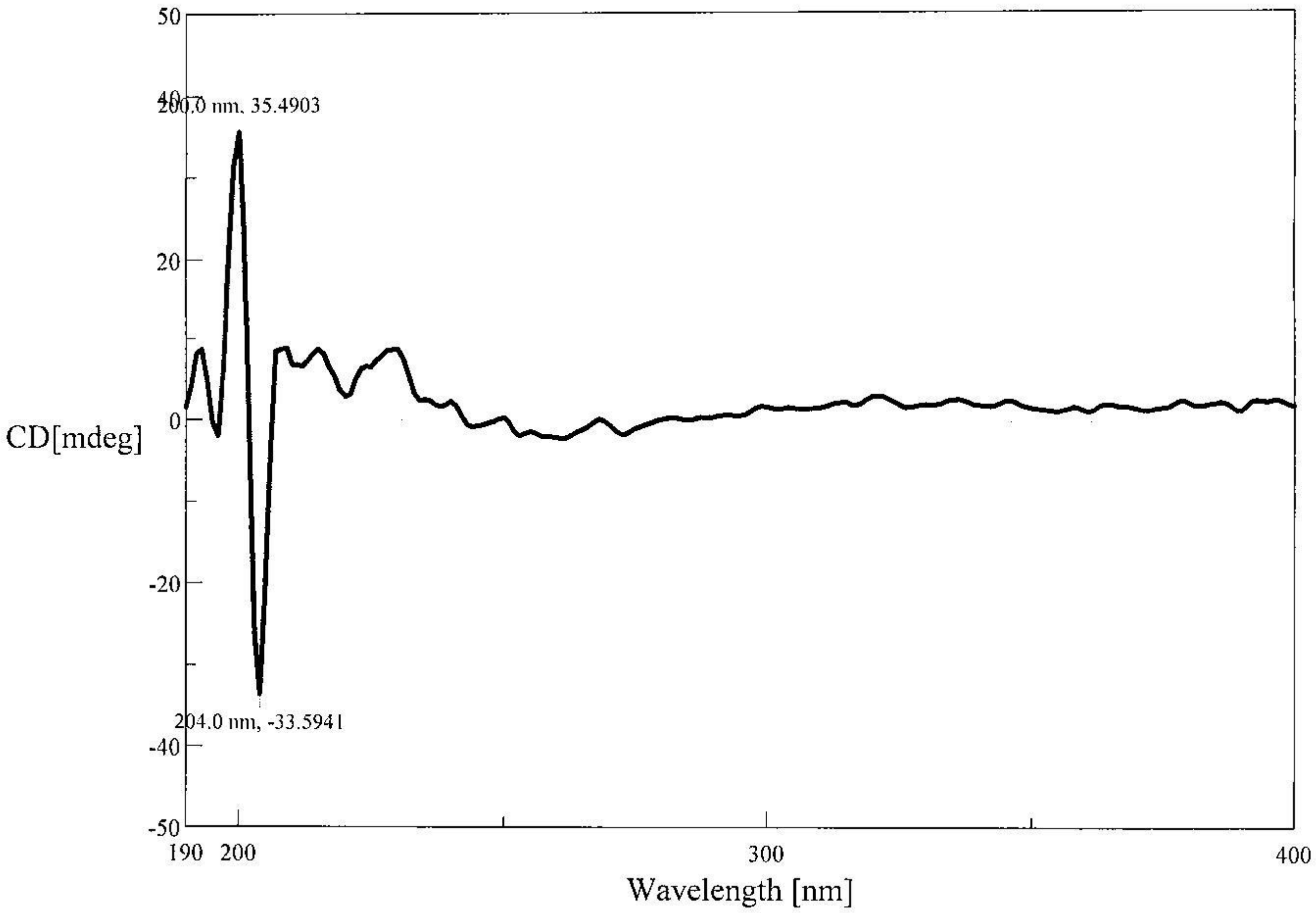

Figure S10. CD spectrum of compound **1**

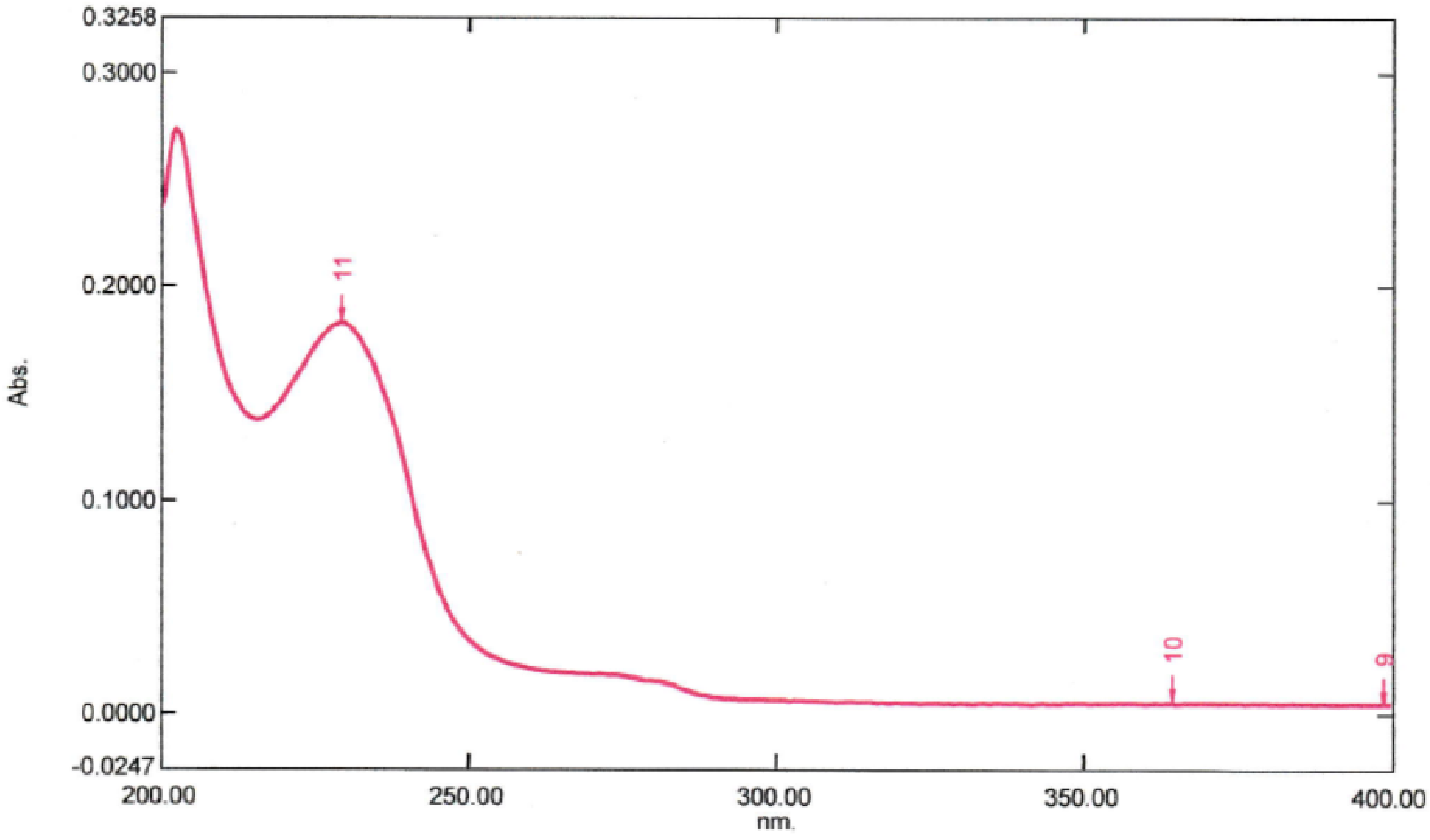

Figure S11. UV spectrum of compound **1**

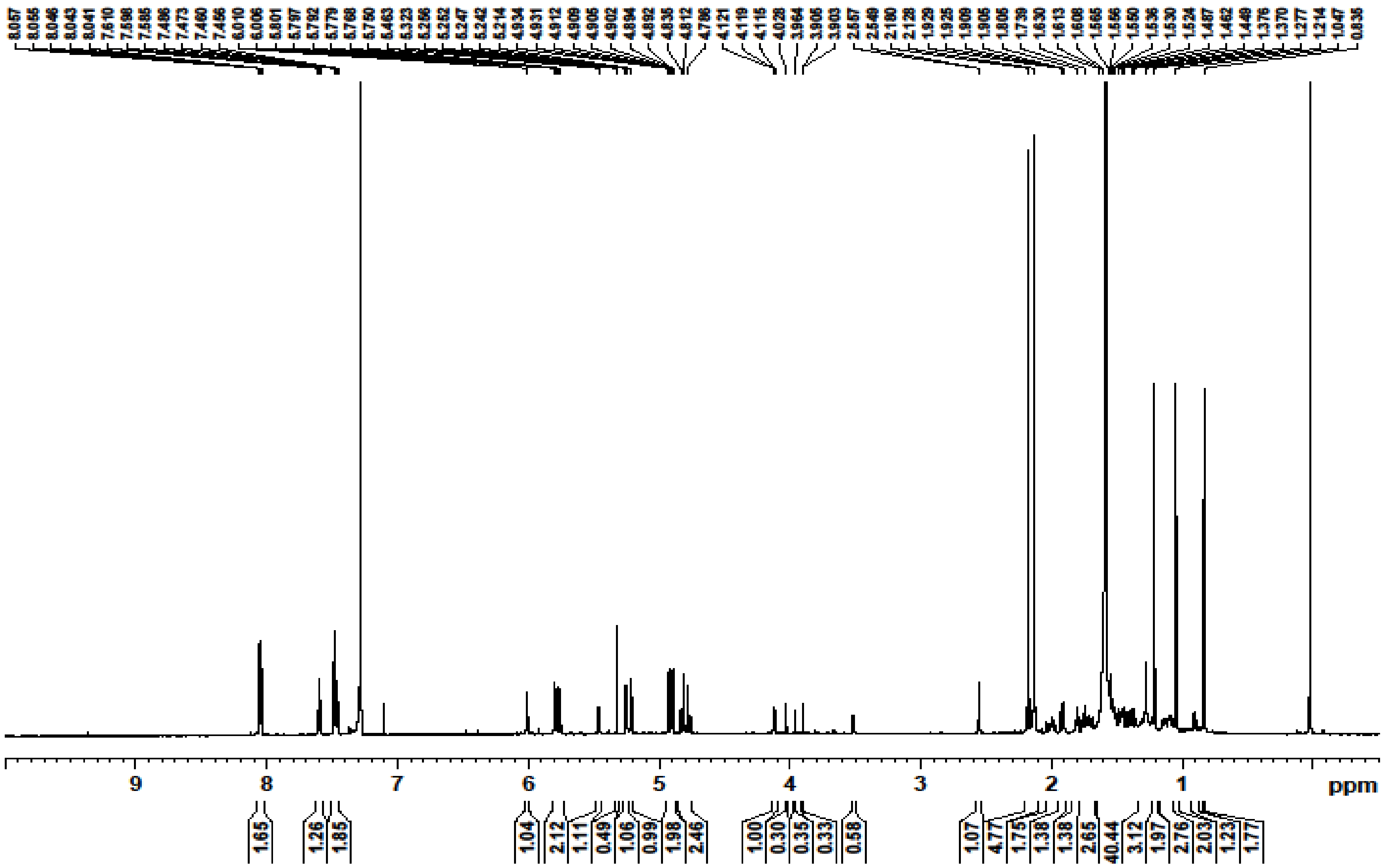

Figure S12. ^{1}H NMR (600 MHz) spectrum of compound **2** in $CDCl_3$

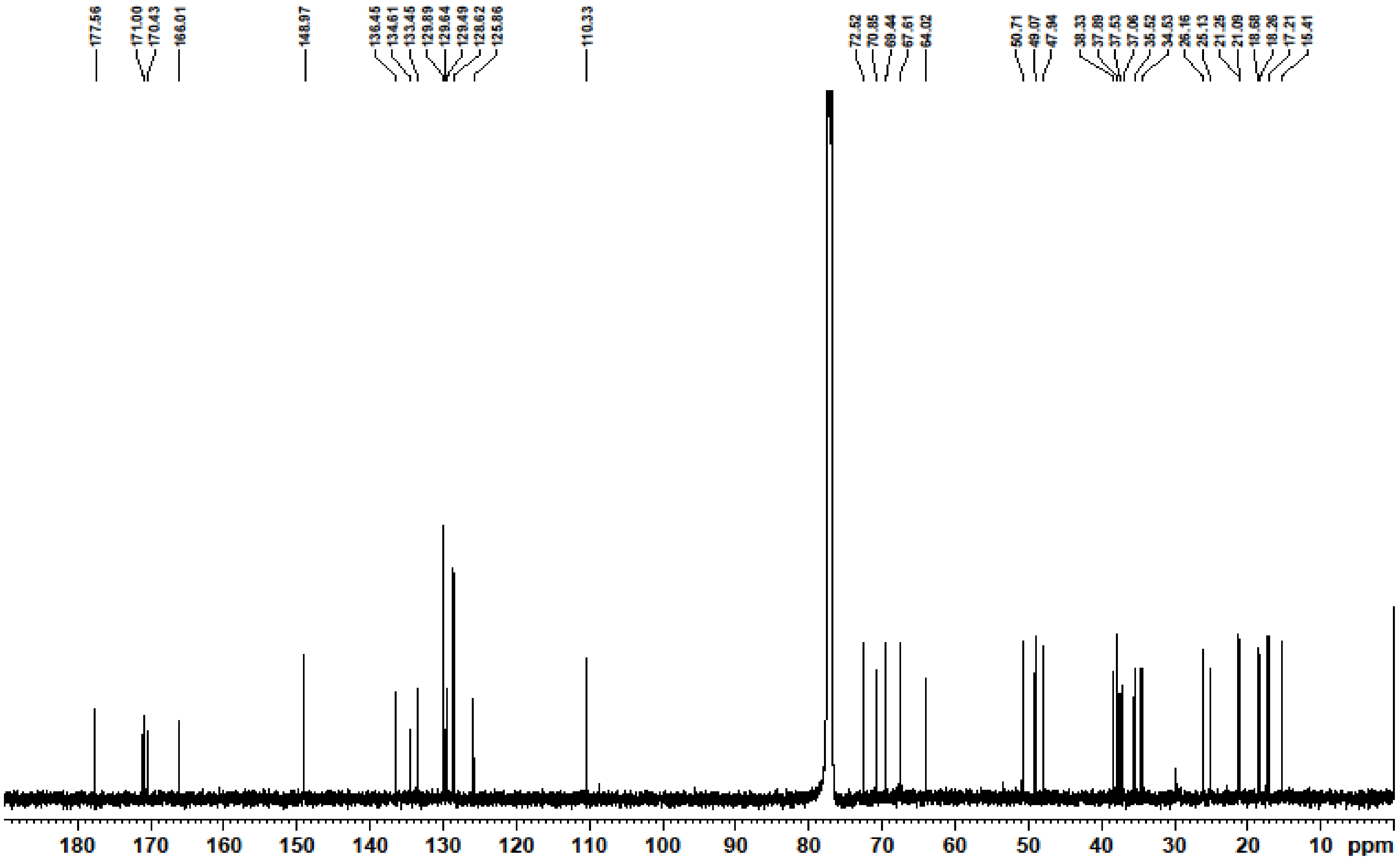

Figure S13. ^{13}C NMR (150 MHz) spectrum of compound **2** in $CDCl_3$

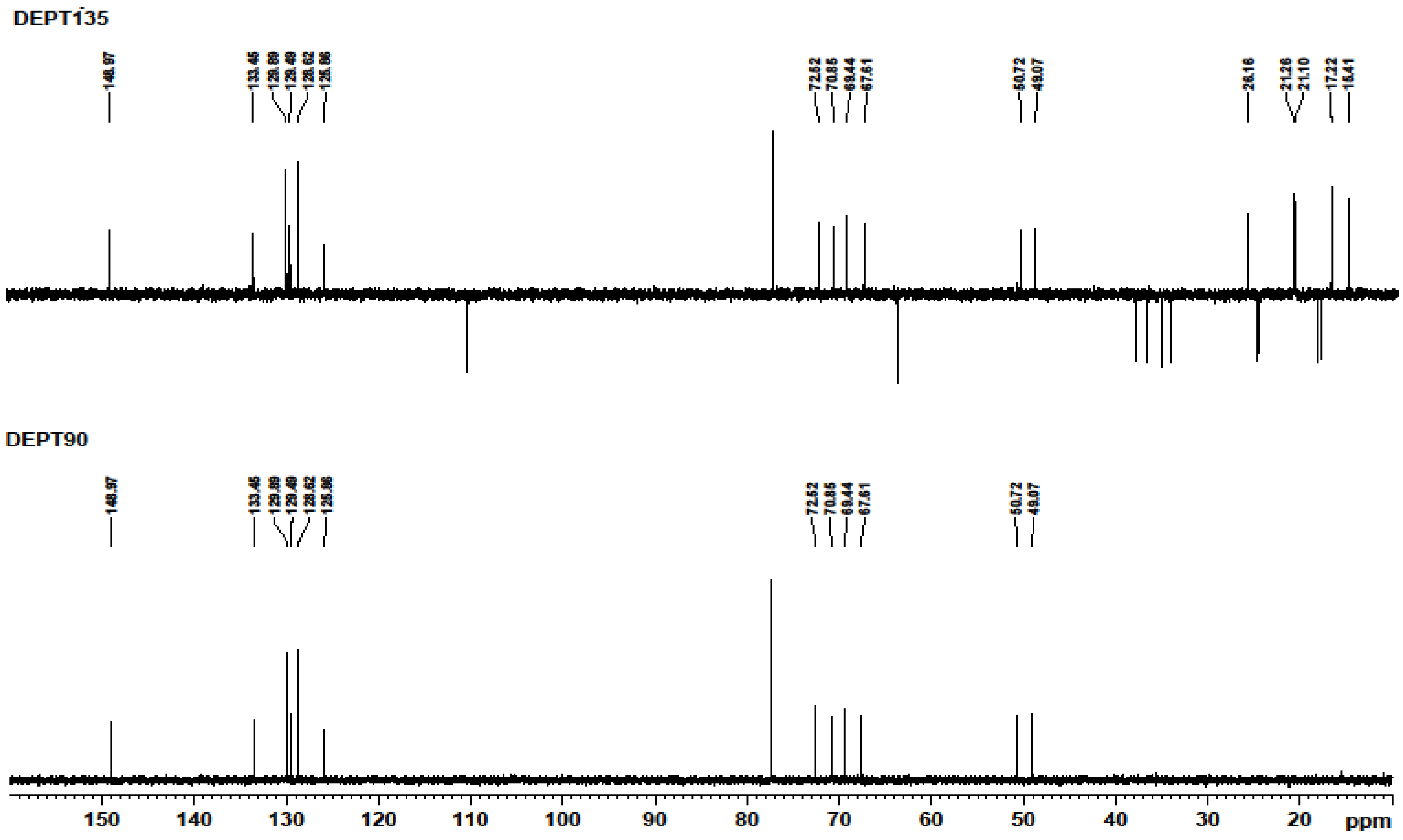

Figure S14. DEPT 135 & 90 NMR spectrum of compound **2** in $CDCl_3$

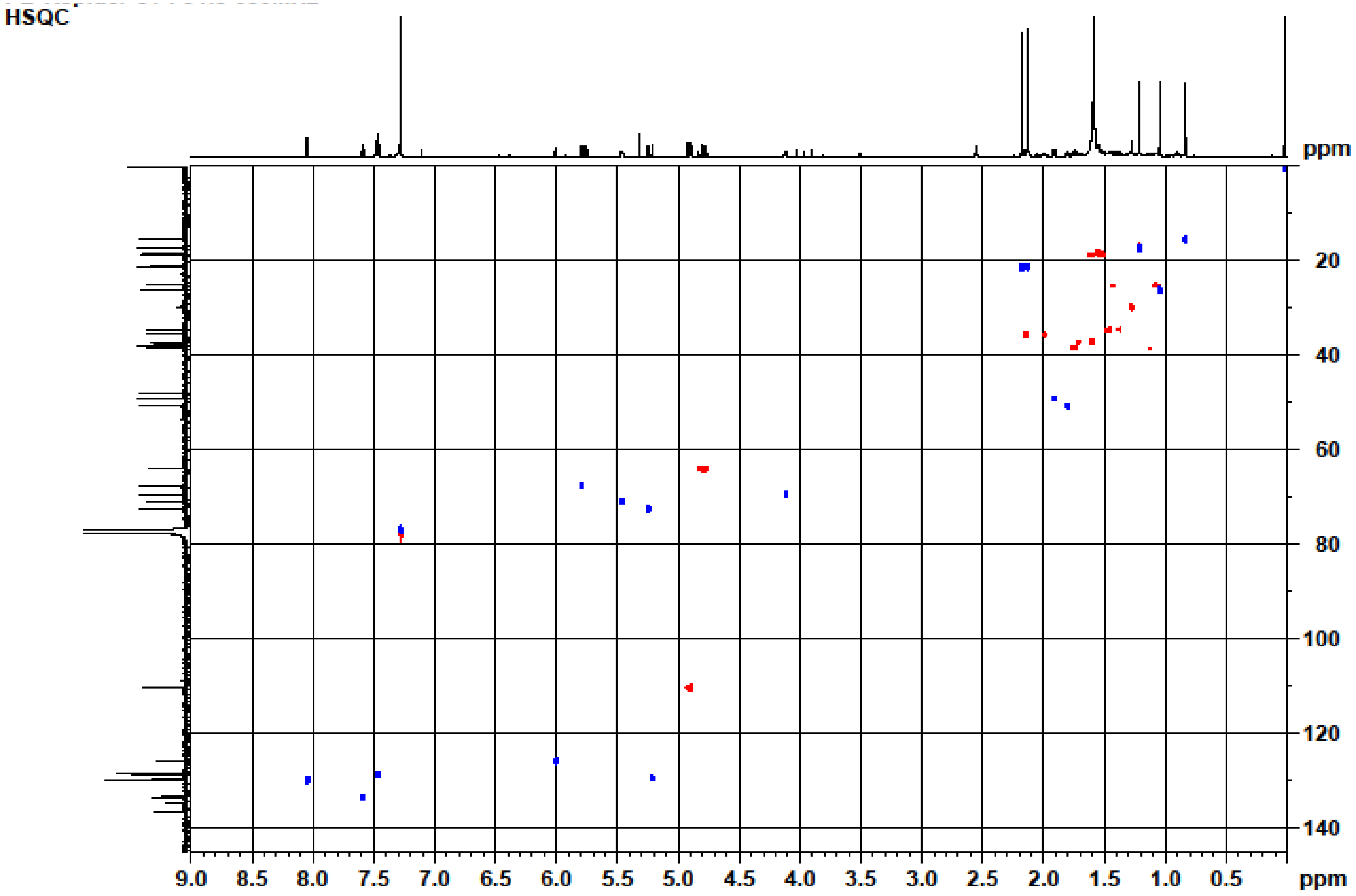

Figure S15. HSQC spectrum of compound **2** in $CDCl_3$

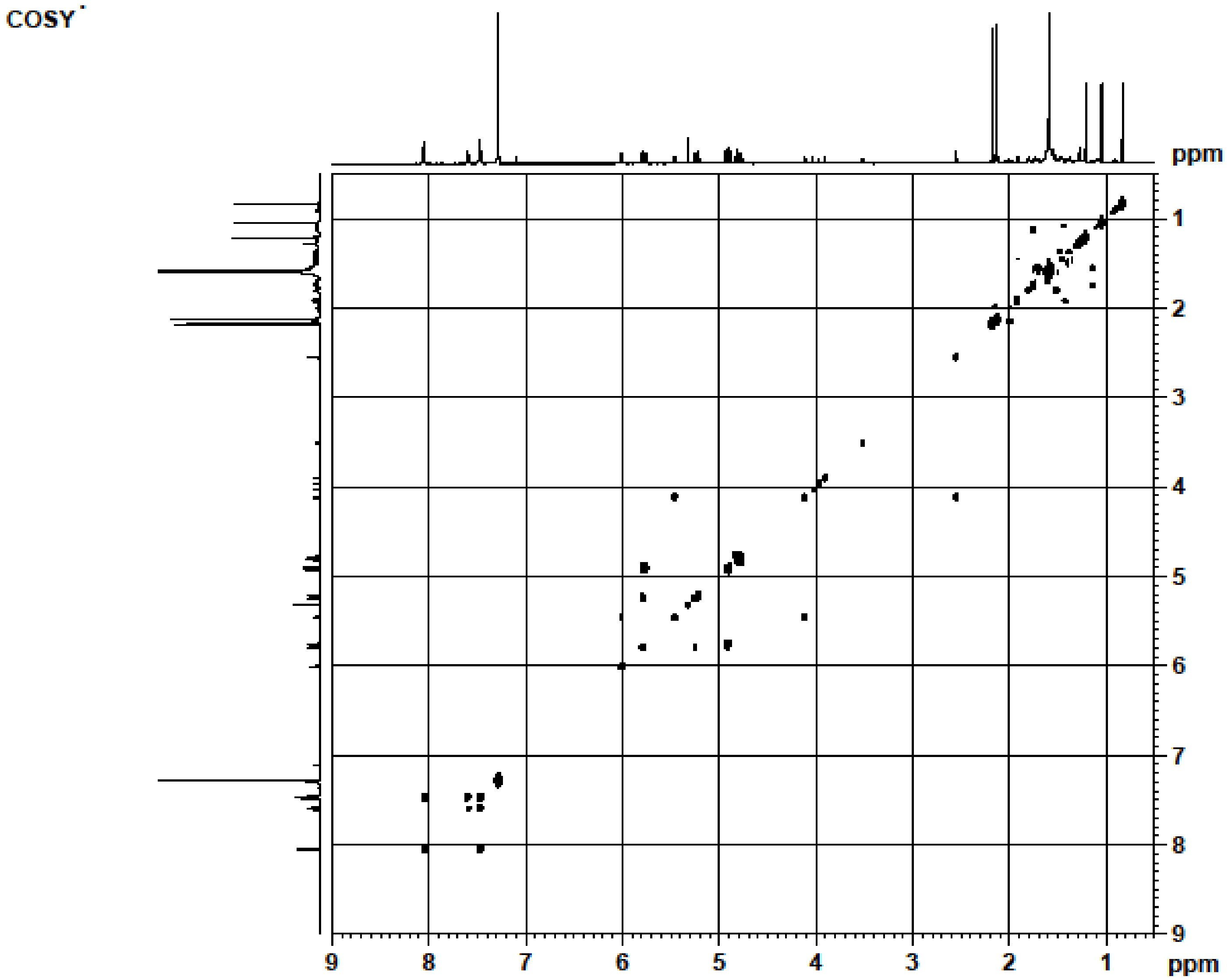

Figure S16. COSY spectrum of compound **2** in $CDCl_3$

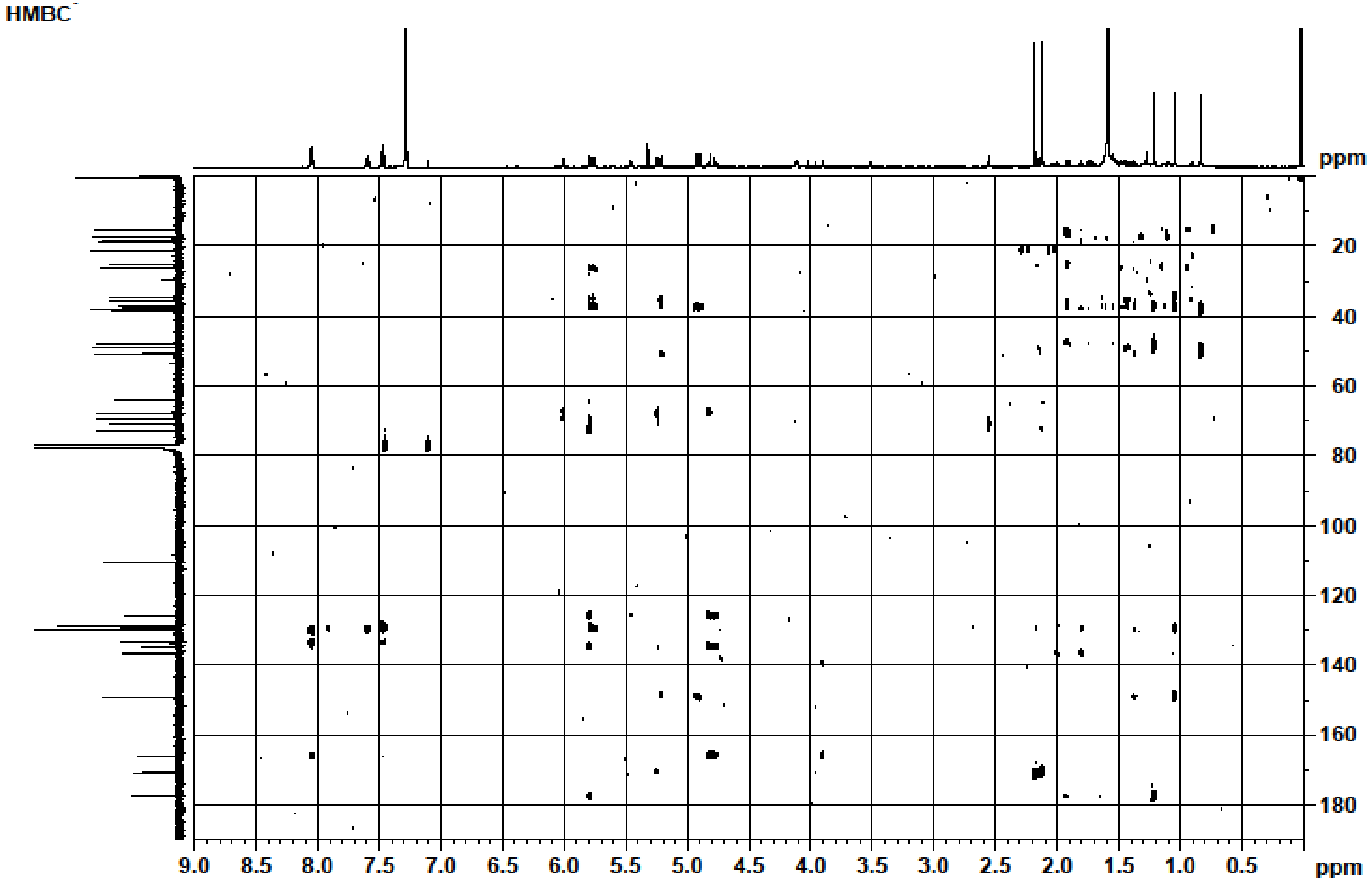

Figure S17. HMBC spectrum of compound **2** in $CDCl_3$

NOESY

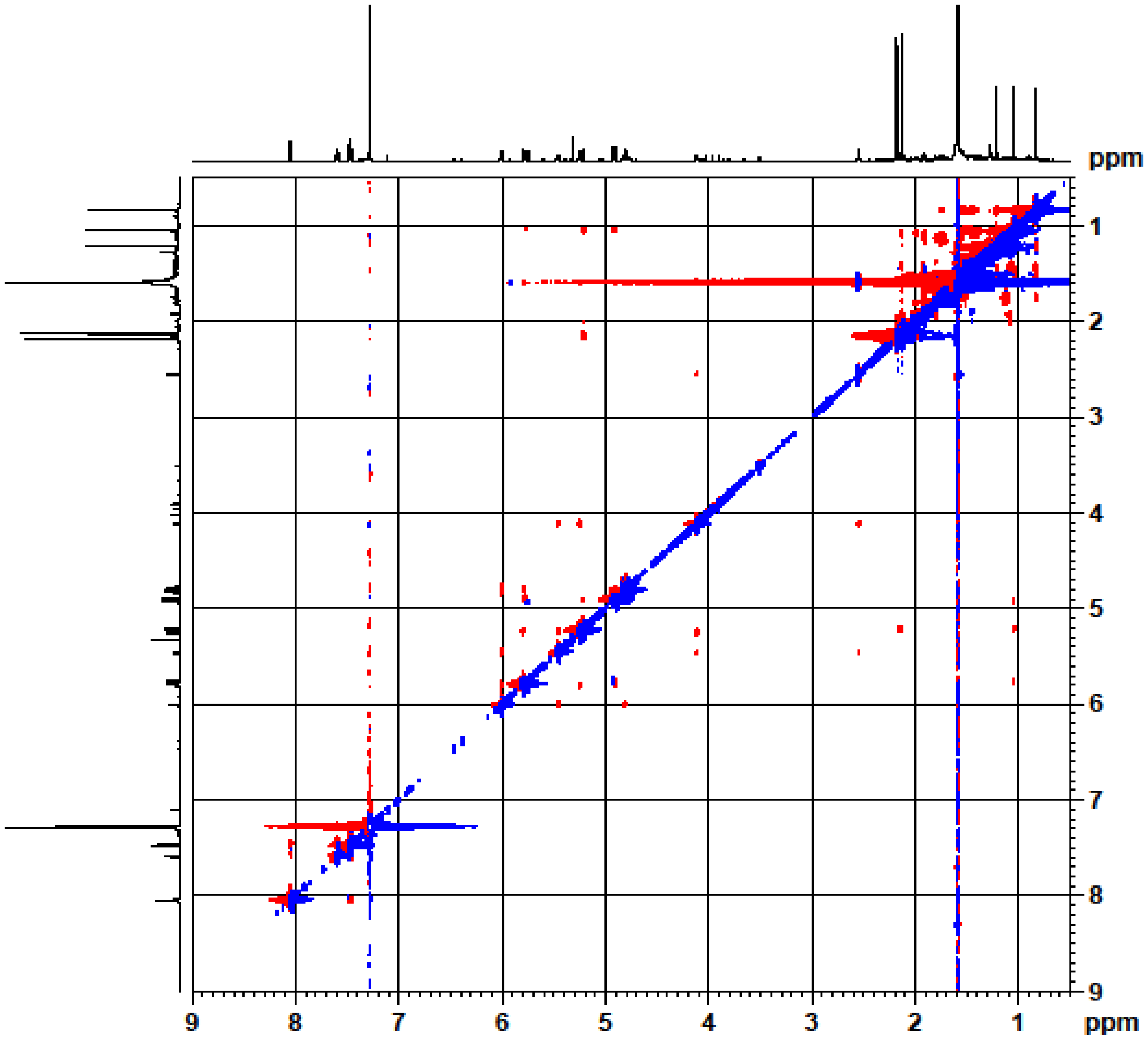

Figure S18. NOESY spectrum of **2** in $CDCl_3$

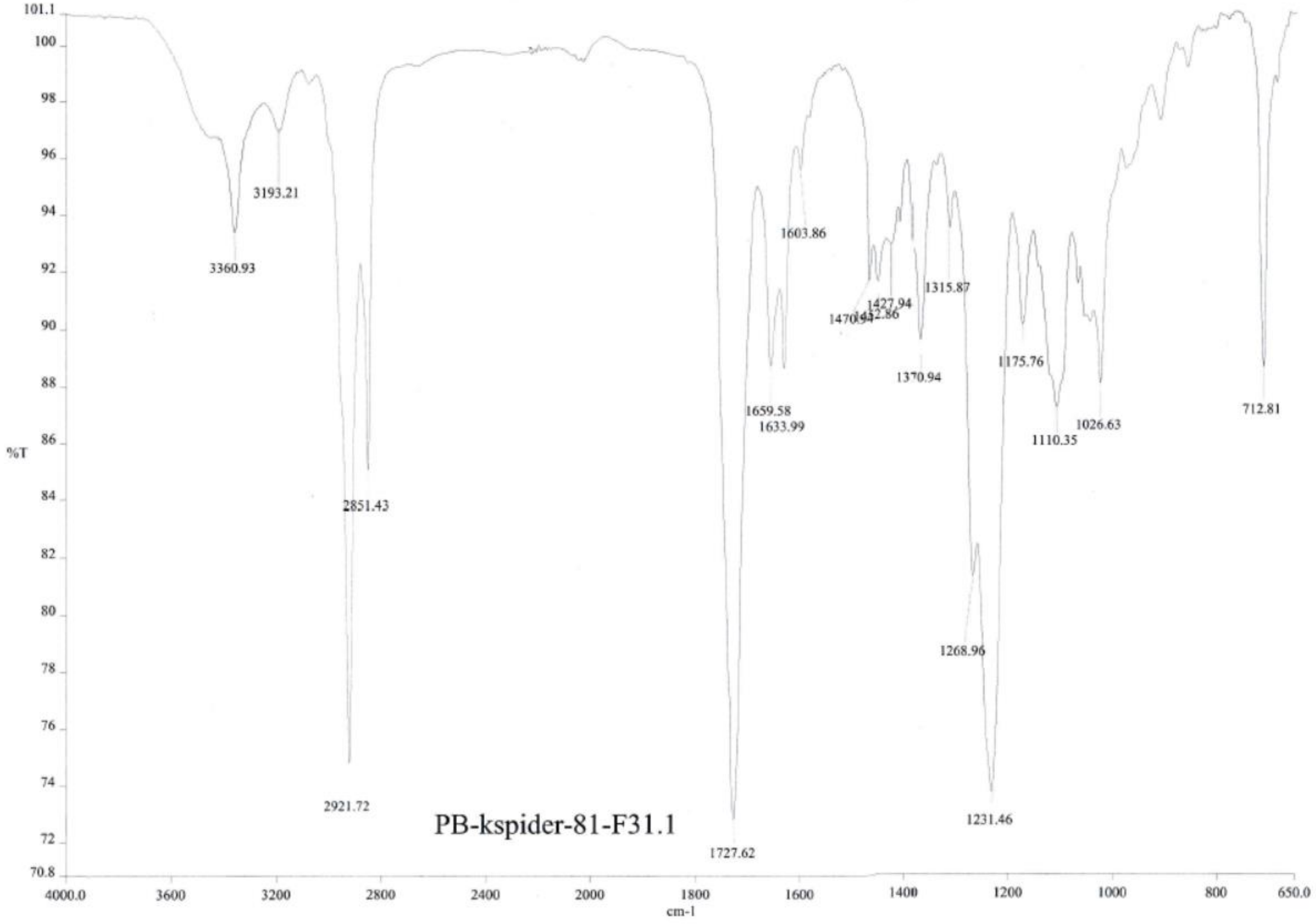

Figure S19. IR spectrum of compound **2**

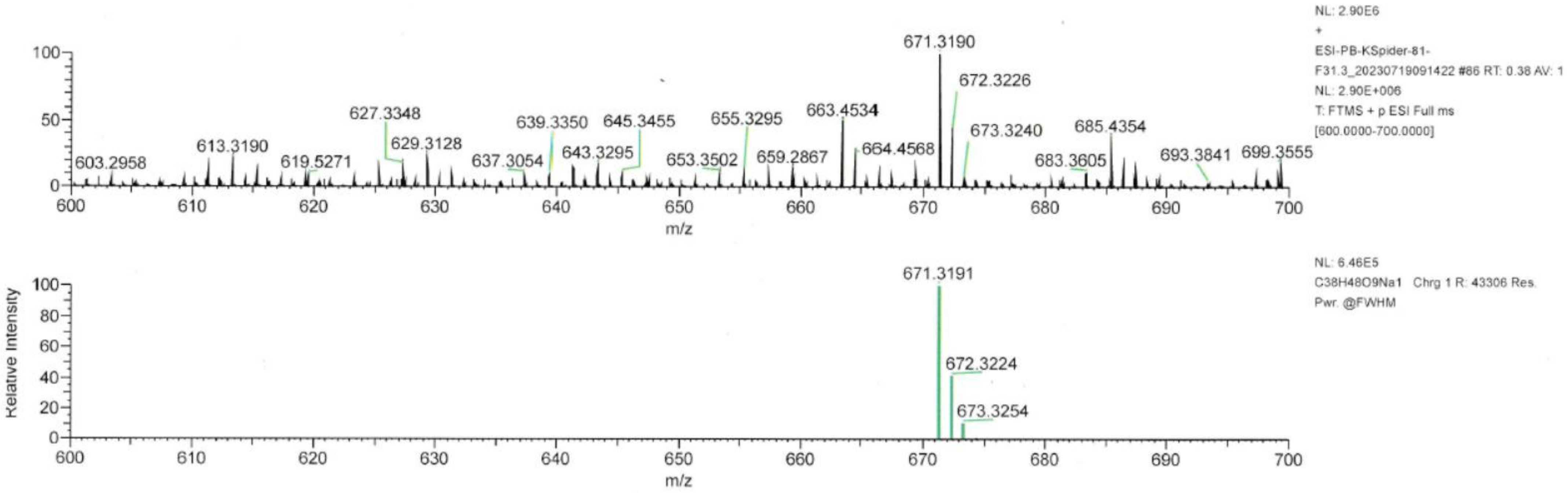

Peak Mass	Display Formula	Delta [ppm]	Theo. mass	MSMS Matched Fragments
671.3190	$C_{38}H_{48}O_9{}^{23}Na$ [M+Na]+	-0.13	671.31905	(Collection)
671.3190	$C_{22}H_{55}O_{22}$	1.52	671.31795	(Collection)
671.3190	$C_{40}H_{47}O_9$	-3.71	671.32146	(Collection)
671.3190	$C_{13}H_{60}O_{27}{}^{23}Na$	-3.65	671.32142	(Collection)

Figure S20. HRESIMS spectrum of compound **2**

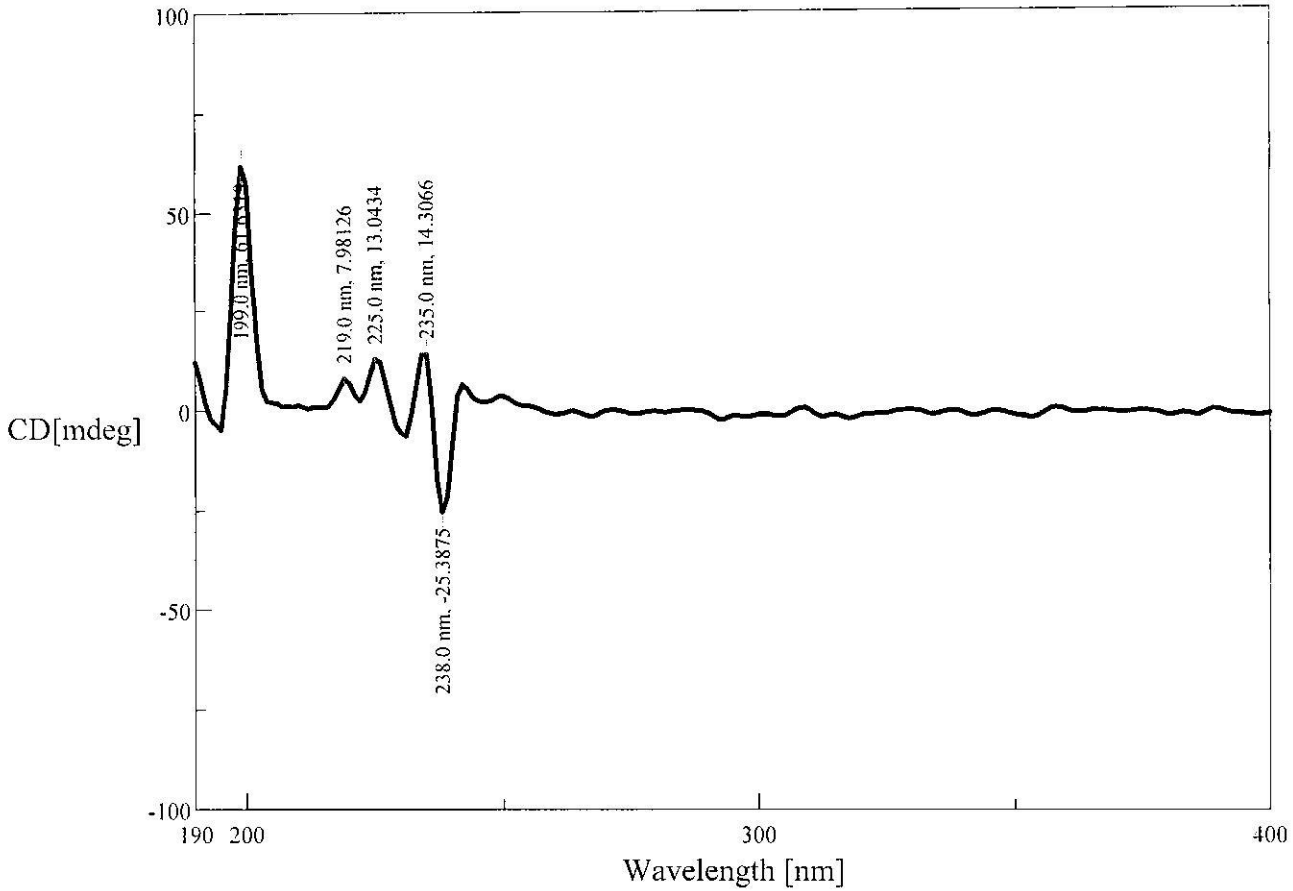

Figure S21. CD spectrum of compound **2**

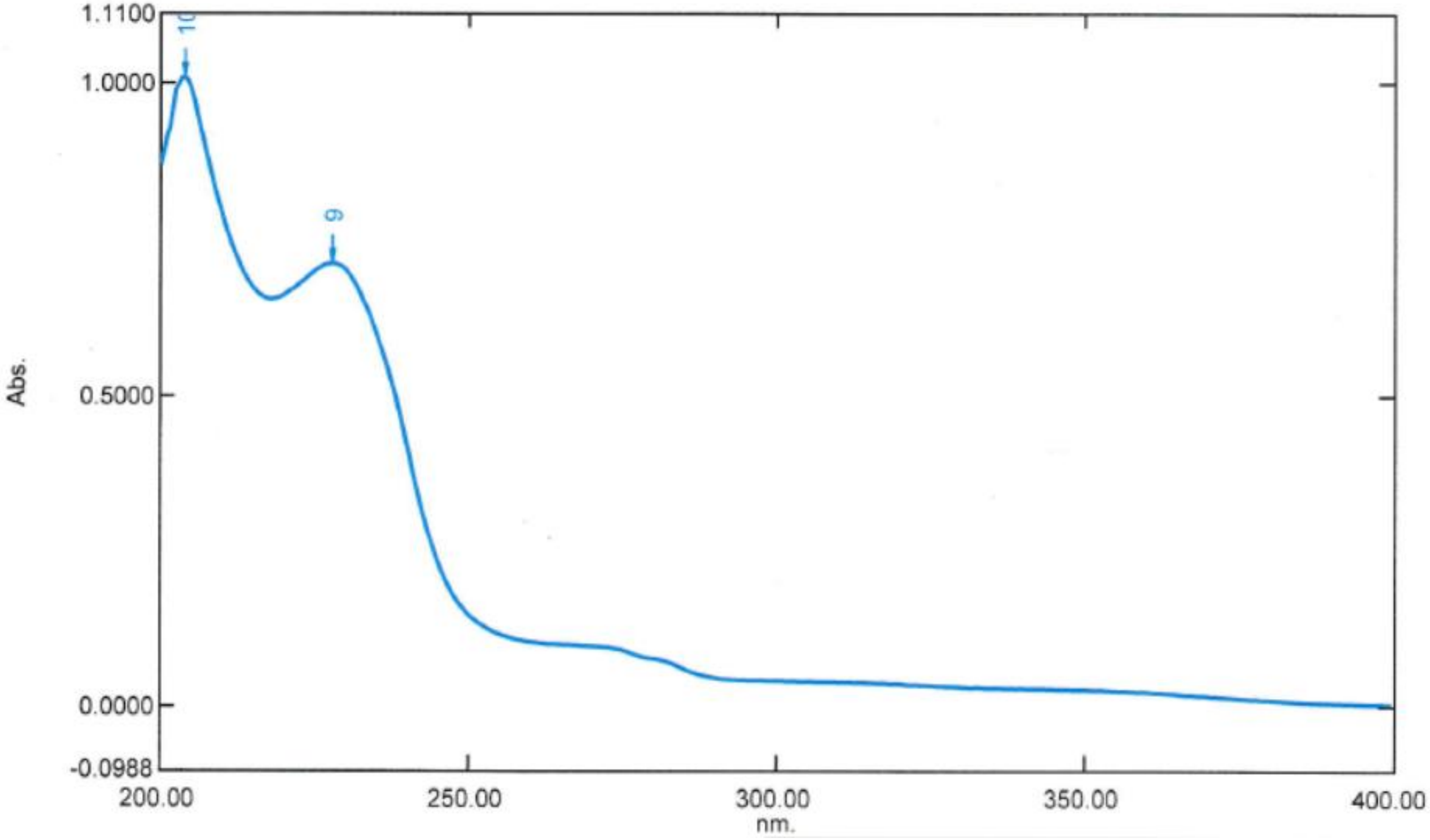

Figure S22. UV spectrum of compound **2**

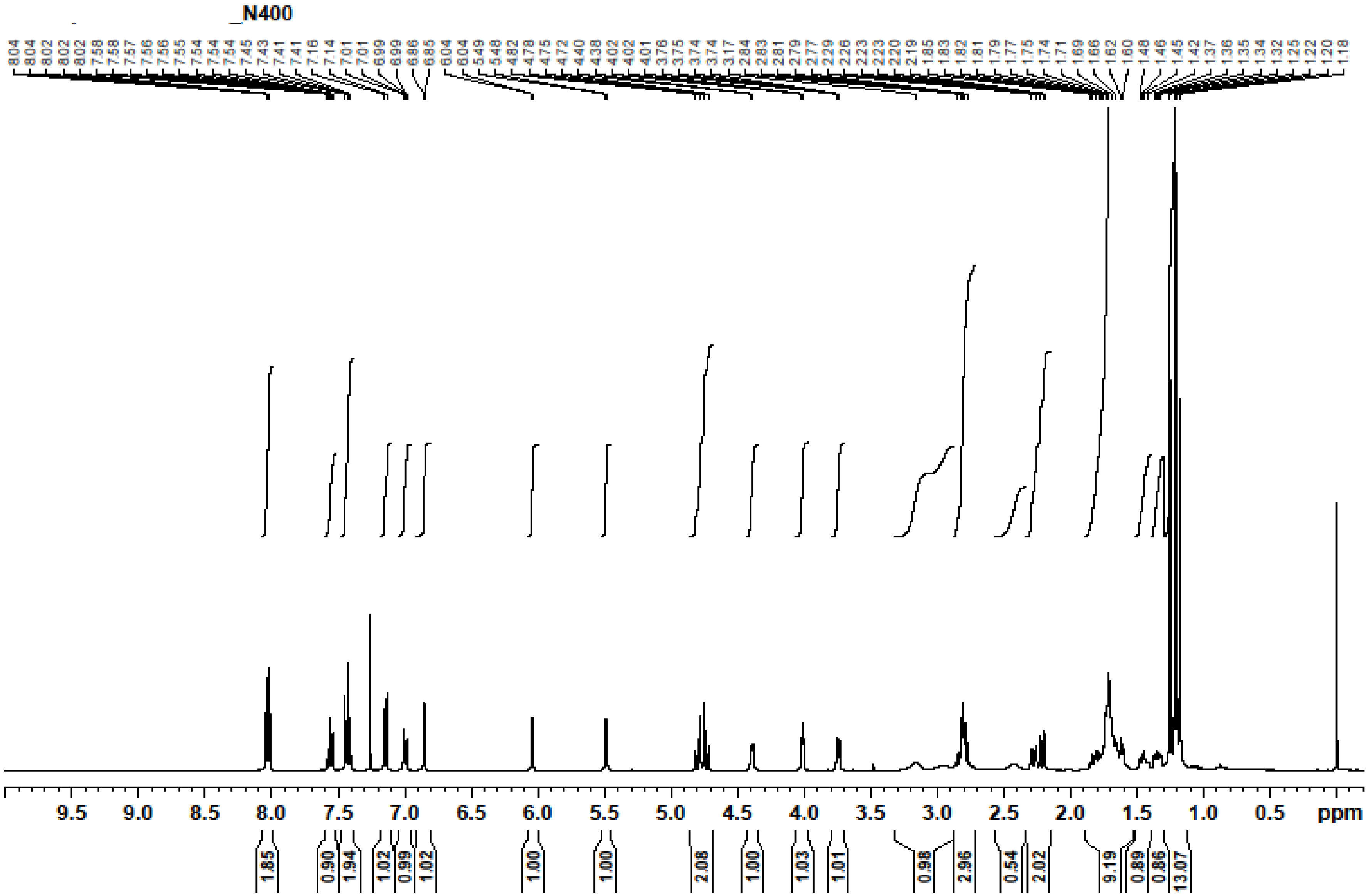

Figure 23. ^{1}H NMR (400 MHz) spectrum of compound **3** in $CDCl_3$

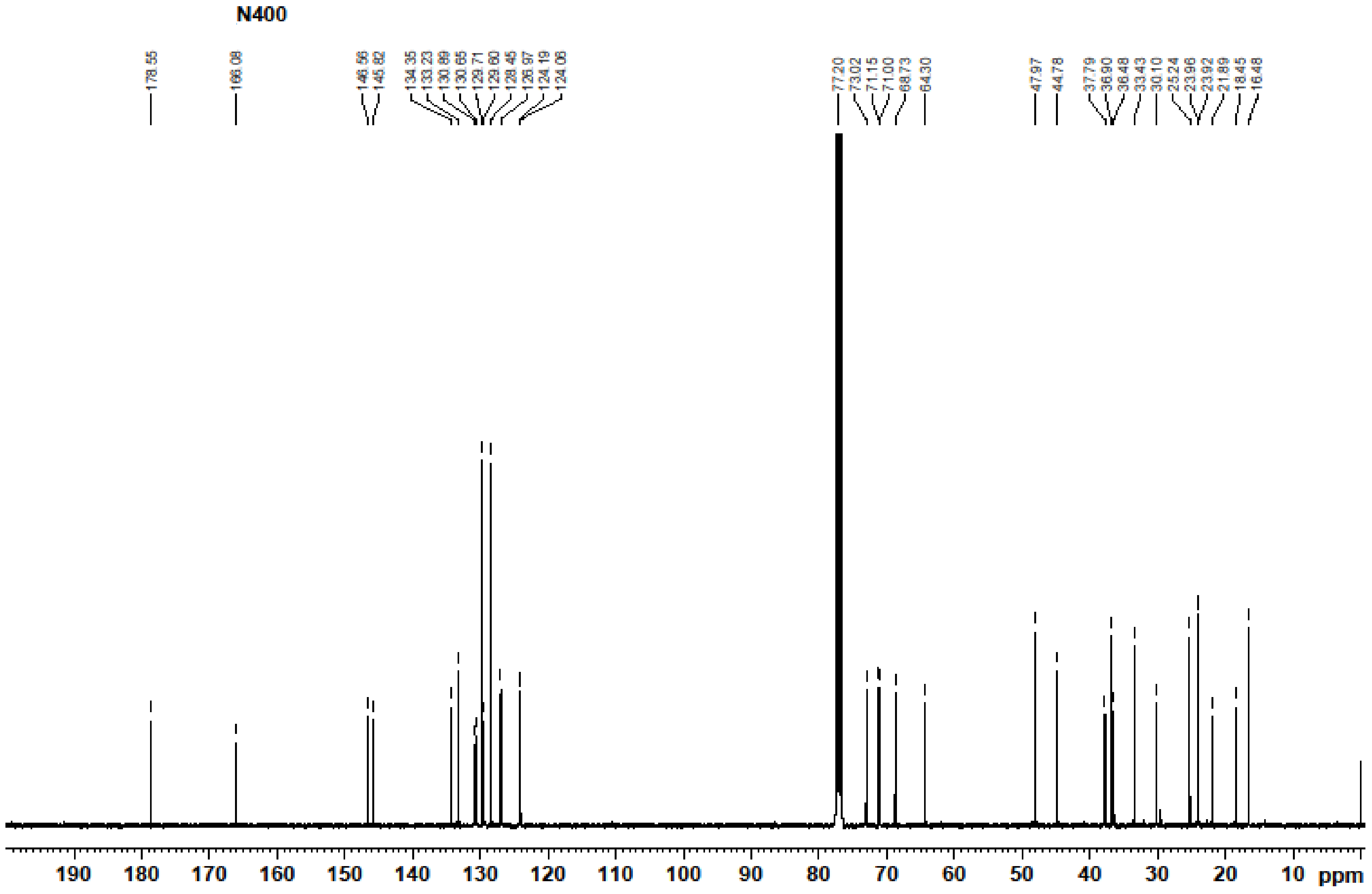

Figure 24. ^{13}C NMR (100 MHz) spectrum of compound **3** in $CDCl_3$

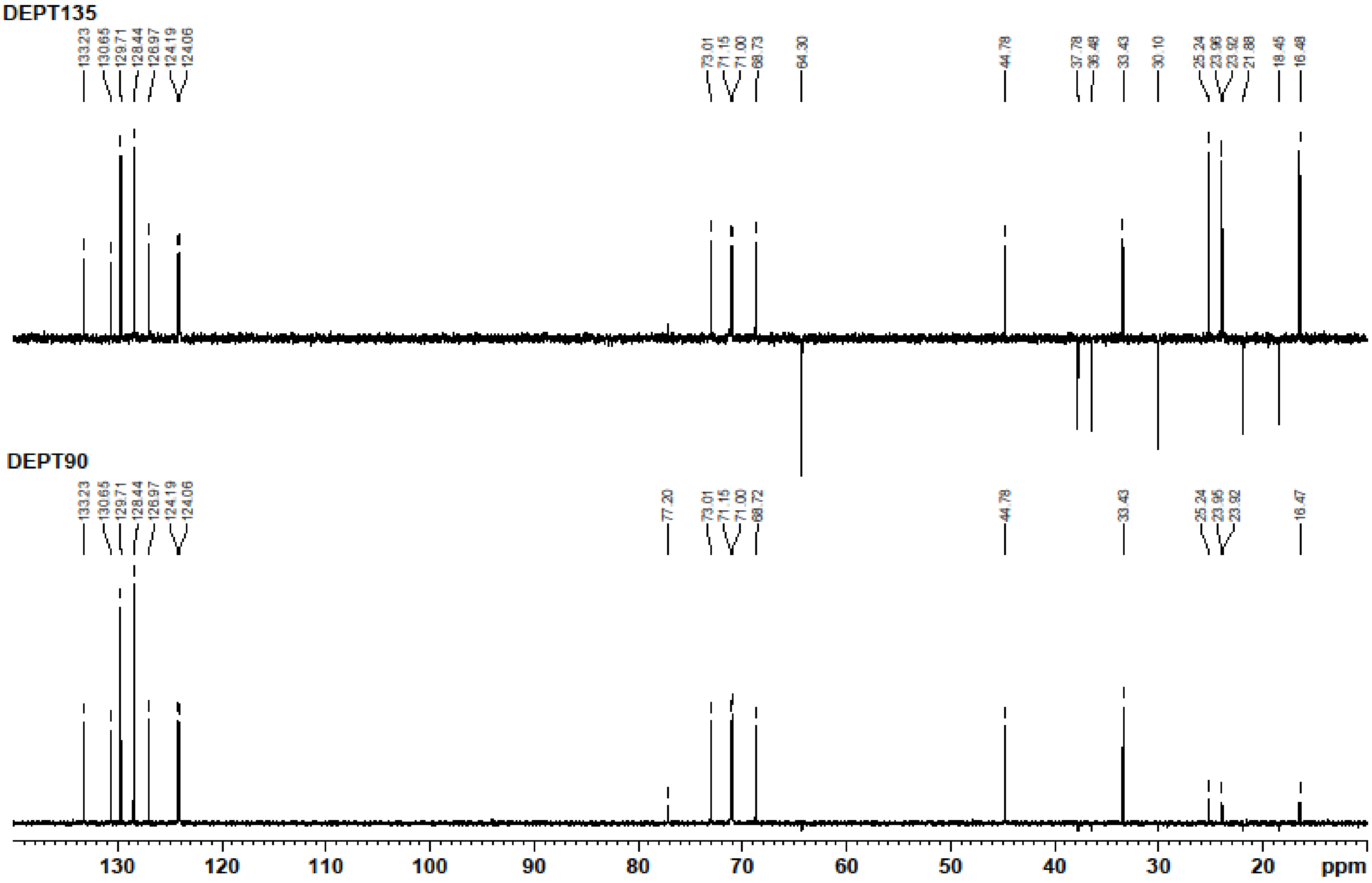

Figure S25. DEPT 135 & 90 NMR spectrum of compound 3 in $CDCl_3$

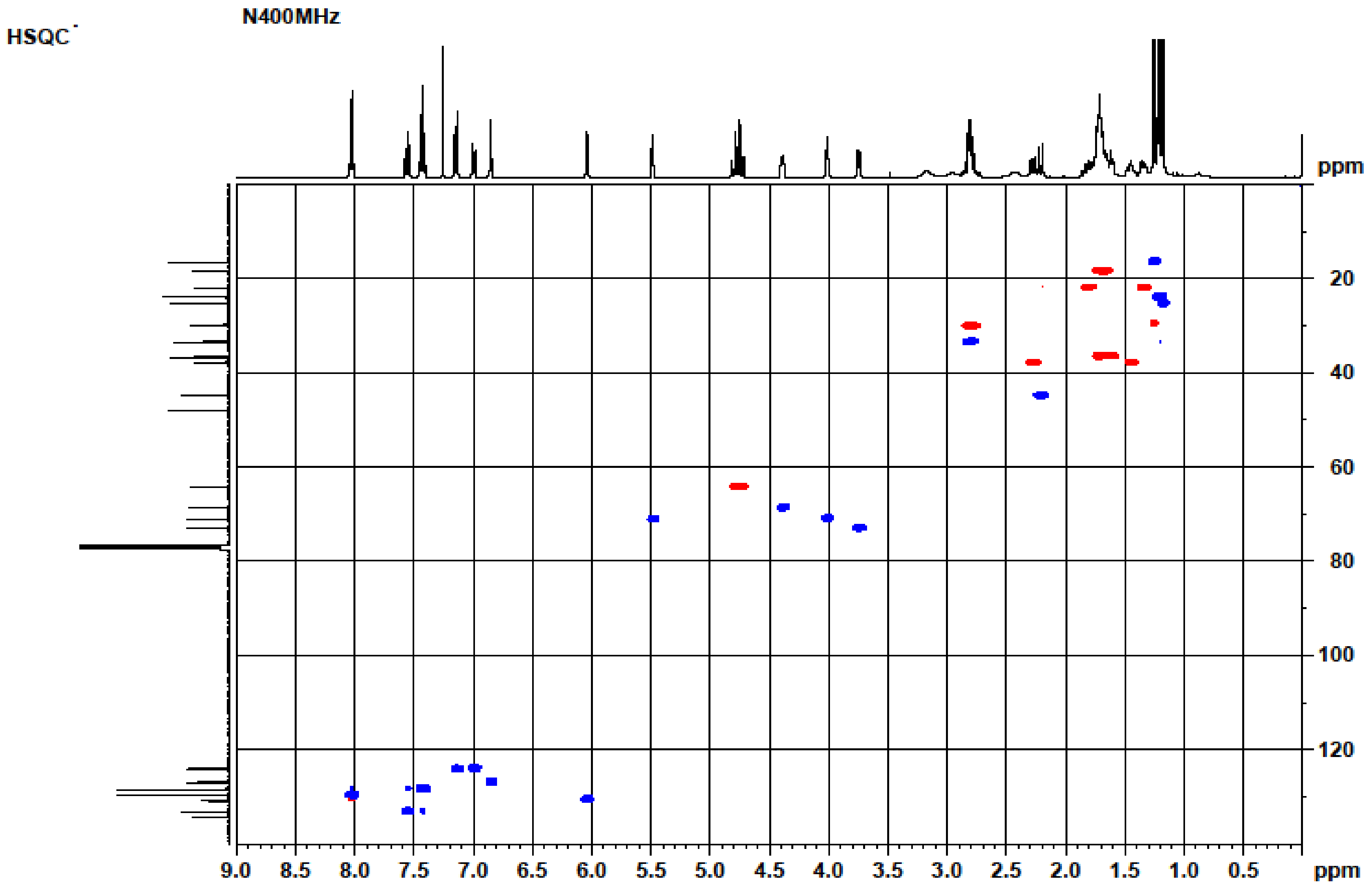

Figure S26. HSQC spectrum of compound **3** in $CDCl_3$

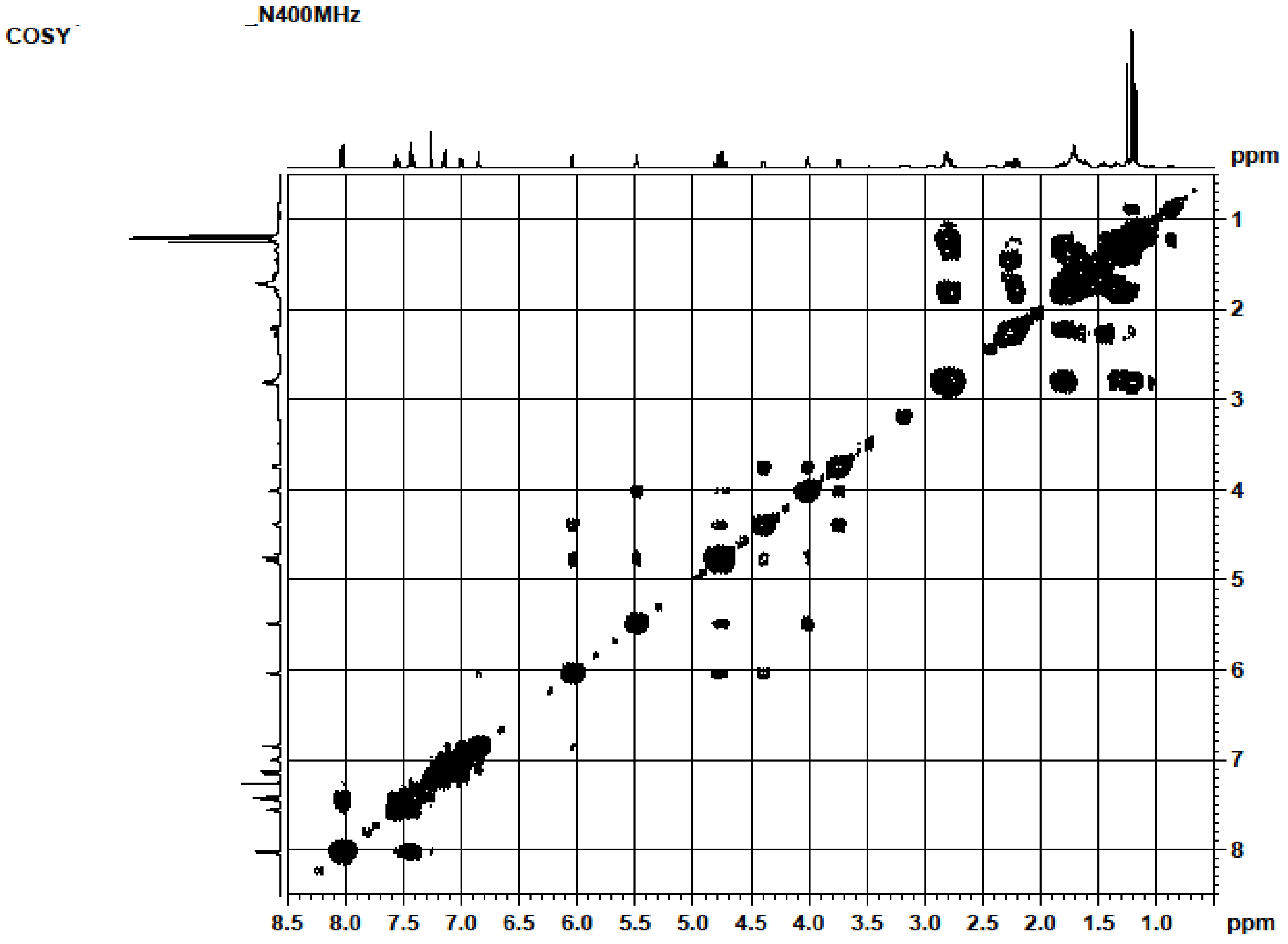

Figure S27. COSY spectrum of compound **3** in $CDCl_3$

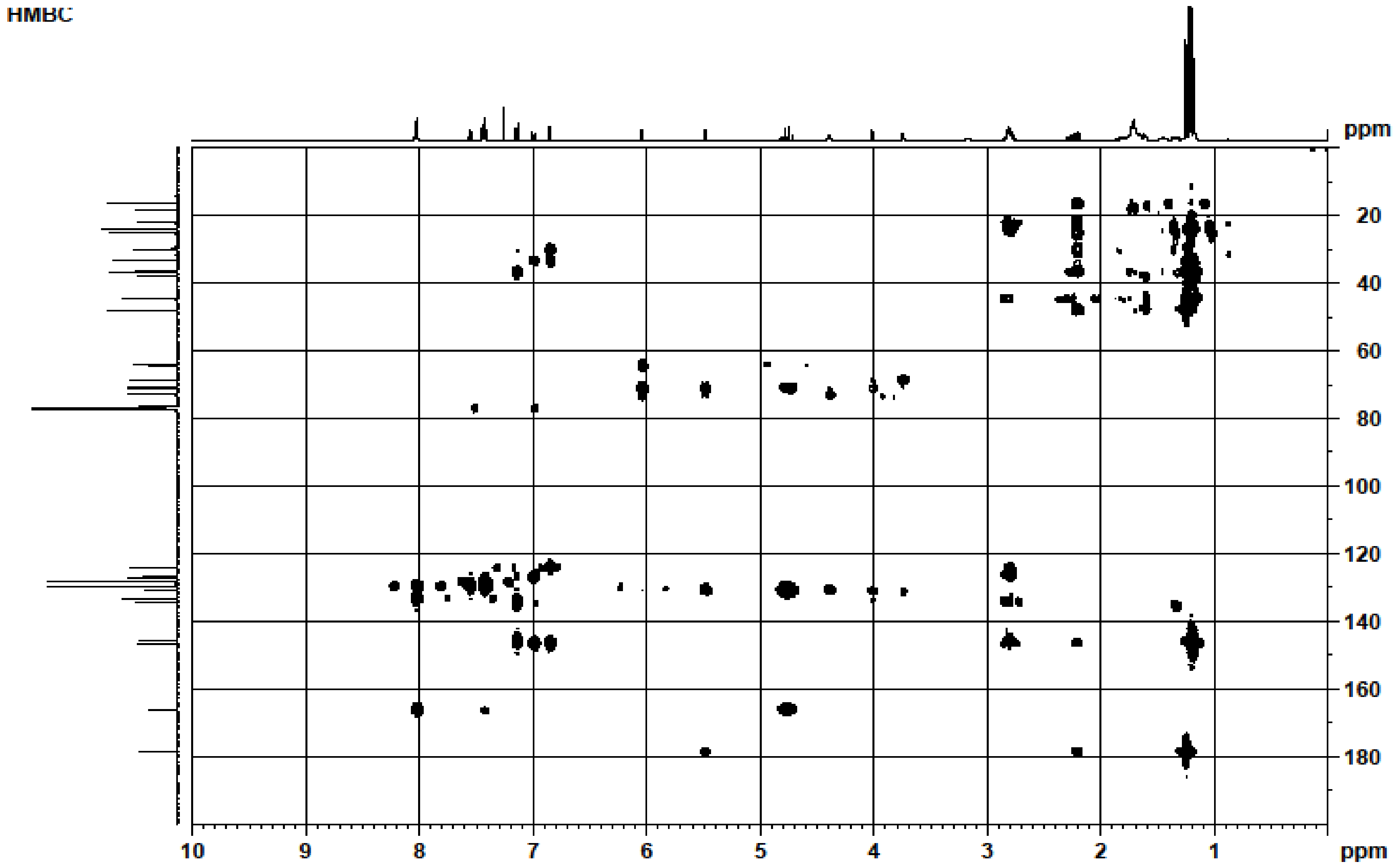

Figure S28. HMBC spectrum of compound **3** in $CDCl_3$

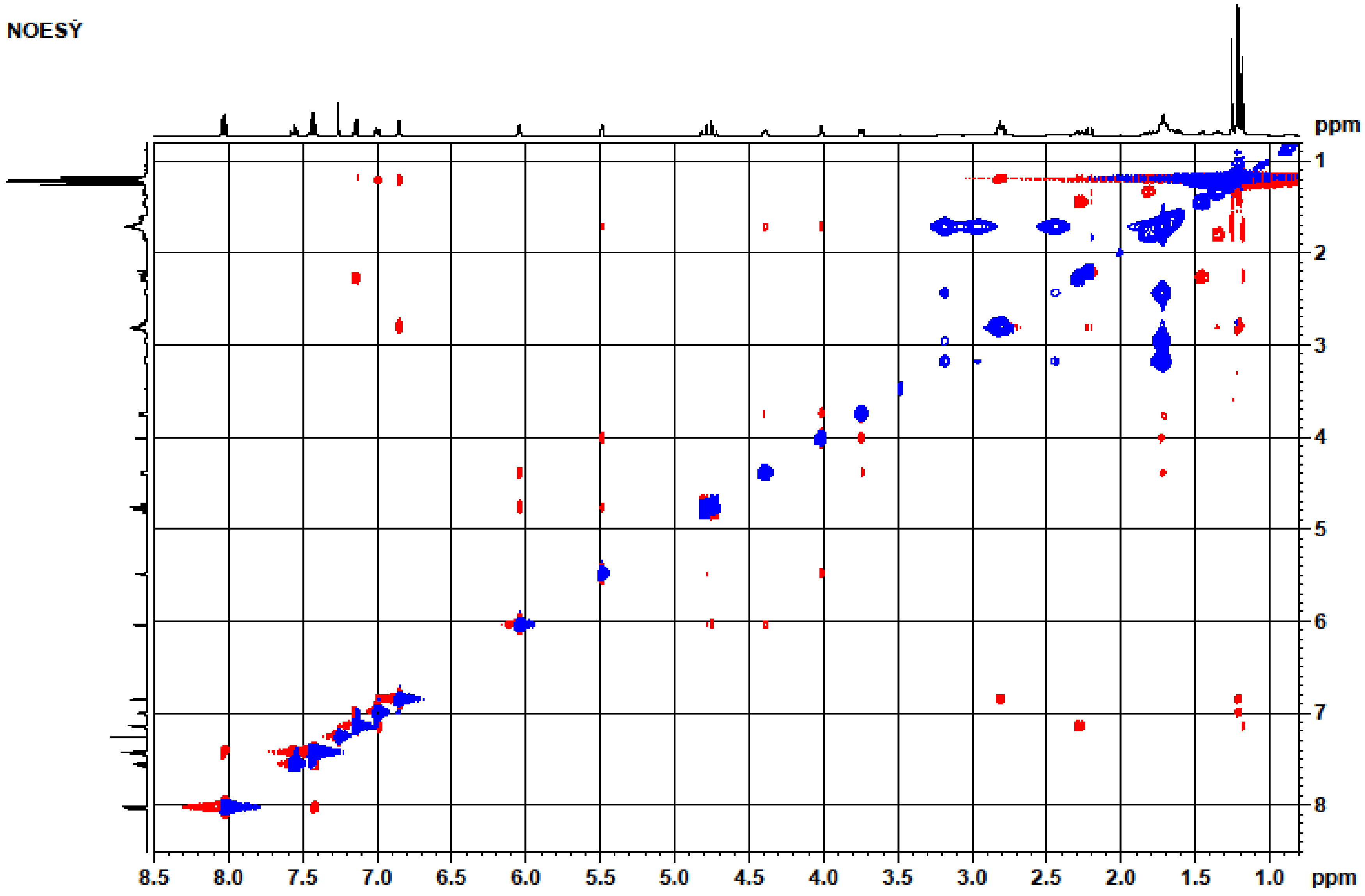

Figure S29. NOESY spectrum of **3** in $CDCl_3$

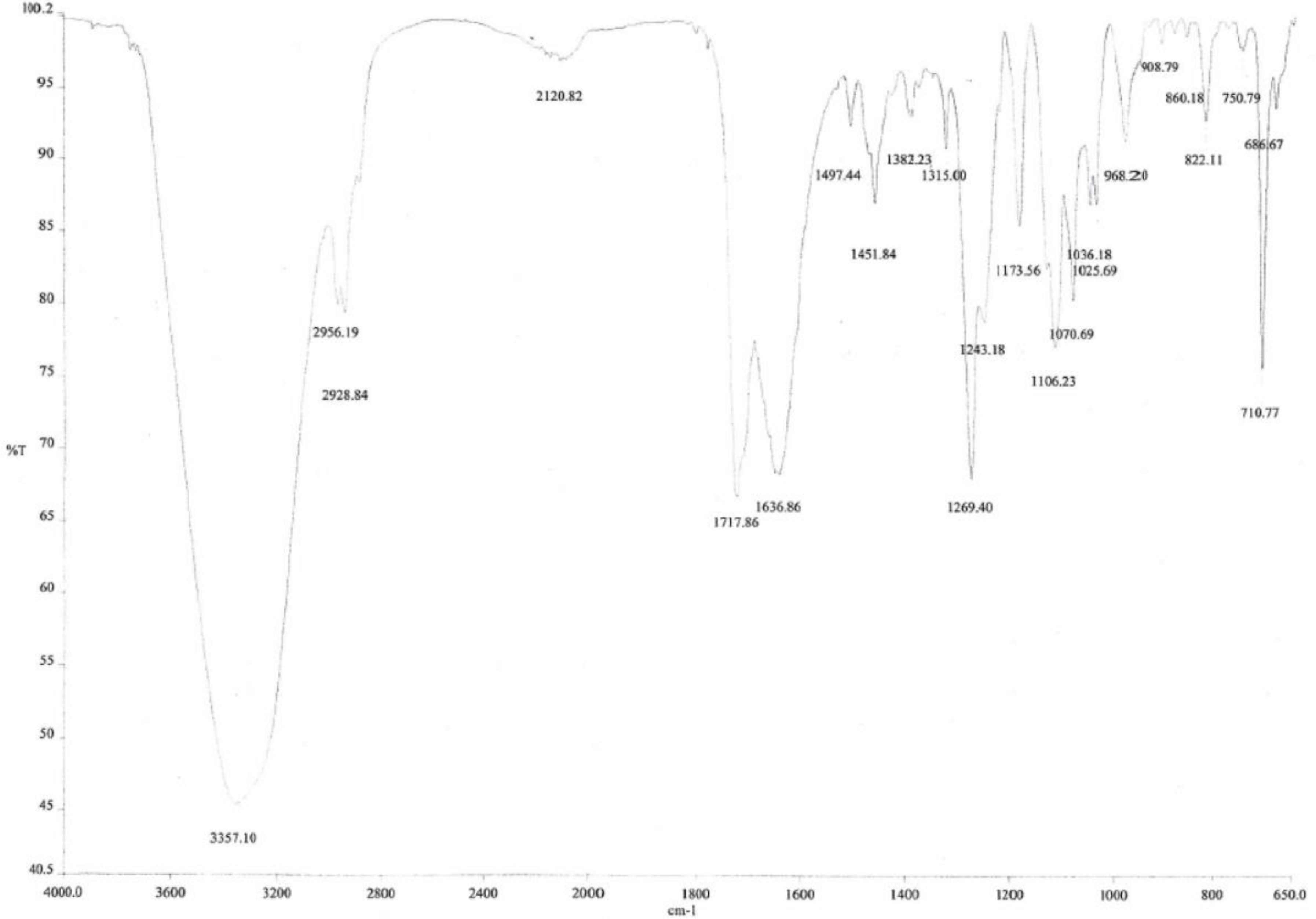

Figure S30. IR spectrum of compound **3**

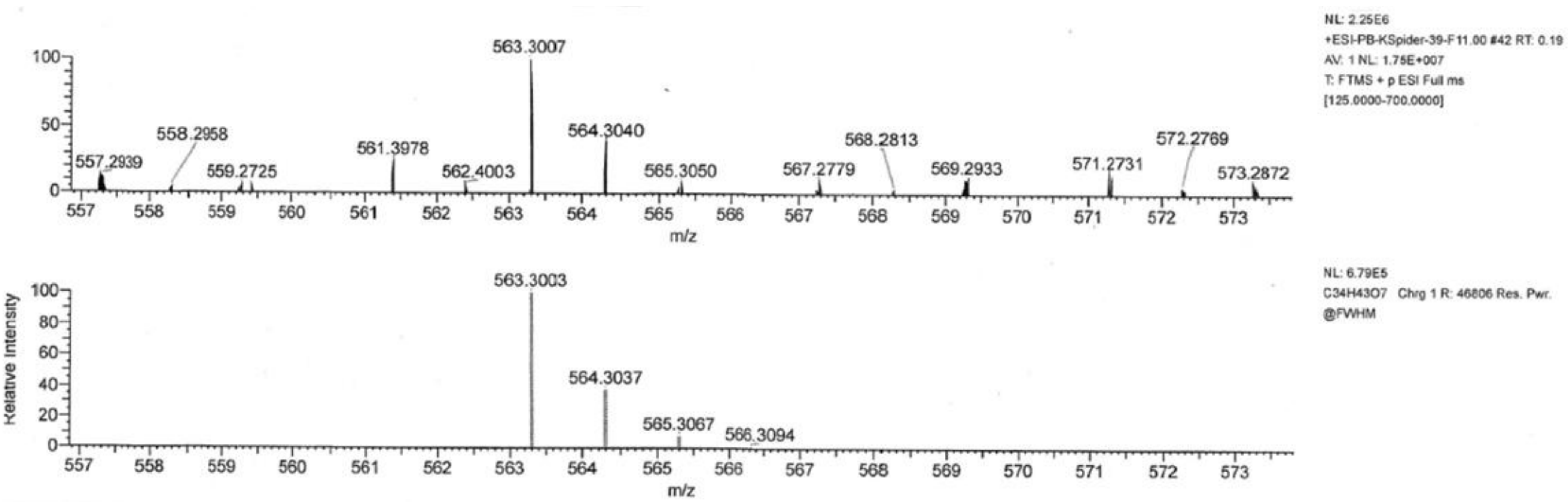

Peak Mass	Display Formula	Delta [ppm]	Theo. mass	MSMS Matched Fragments
563.3007	$C_{34}H_{43}O_7$ [M+H]+	0.69	563.30033	(Collection)
563.3007	$C_9H_{55}O_{25}$	-3.50	563.30269	(Collection)

Figure S31. HRESIMS spectrum of compound **3**

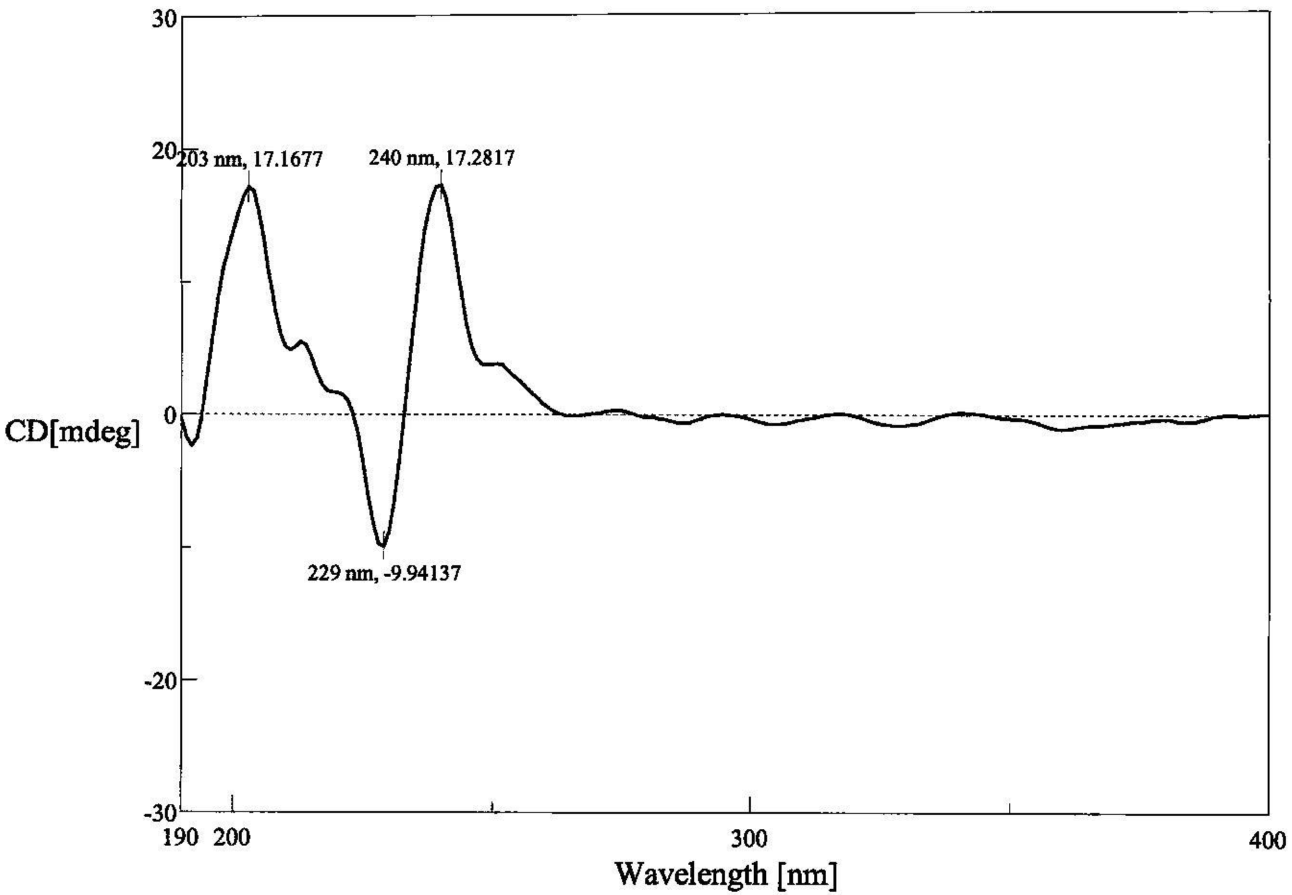

Figure S32. CD spectrum of compound **3**

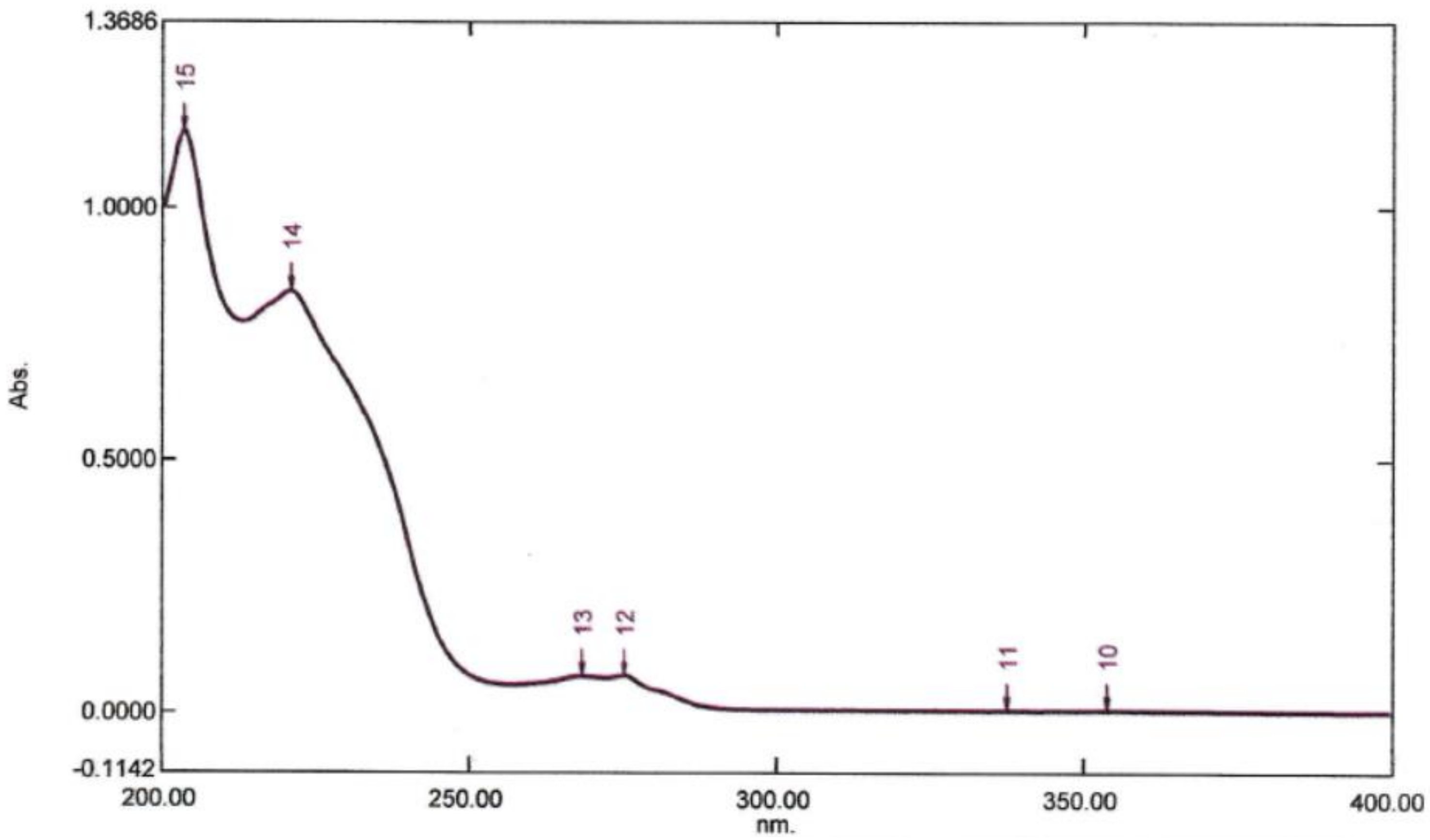

Figure S33. UV spectrum of compound **3**

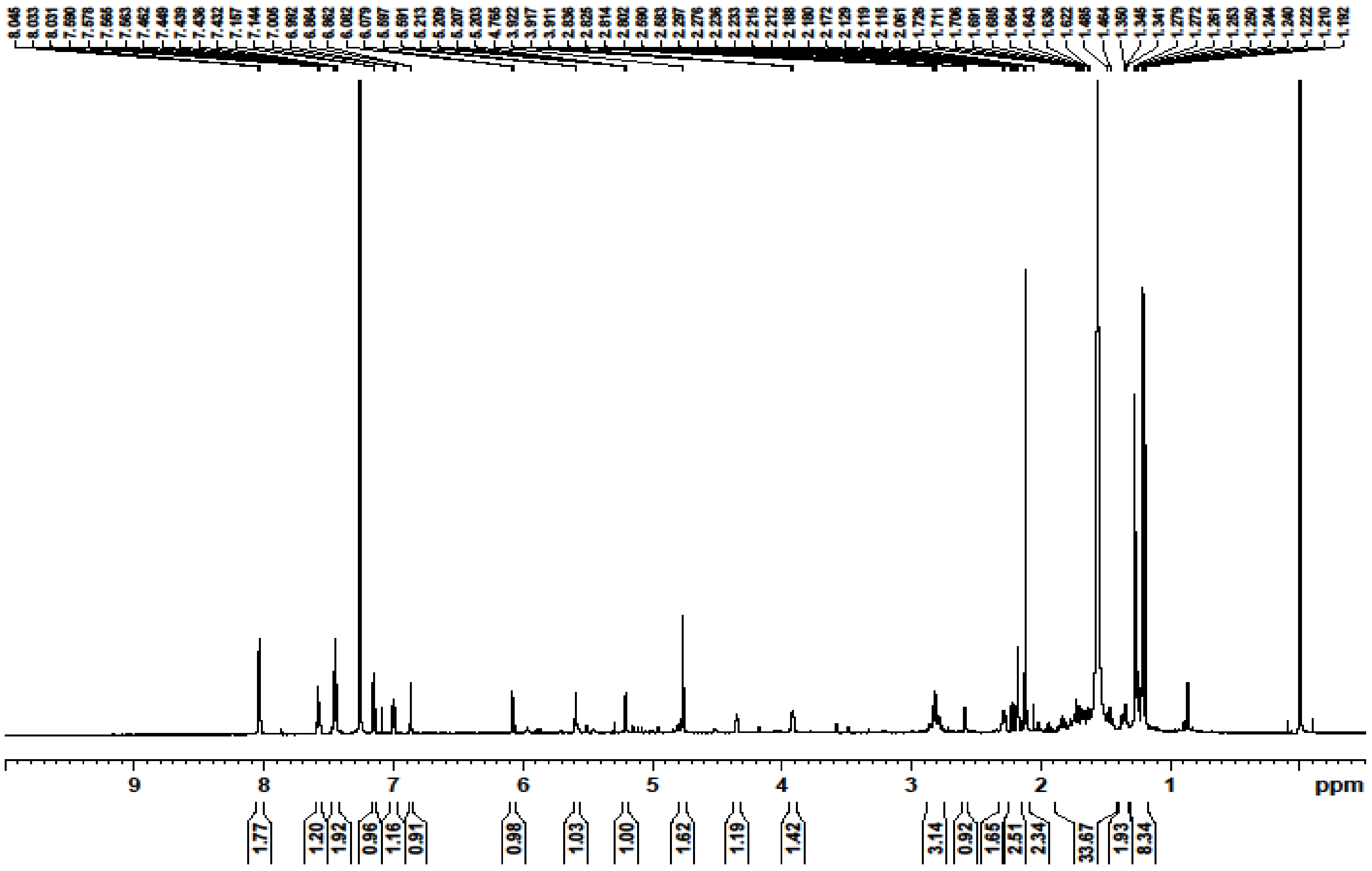

Figure S34. ^{1}H NMR (600 MHz) spectrum of compound **4** in $CDCl_3$

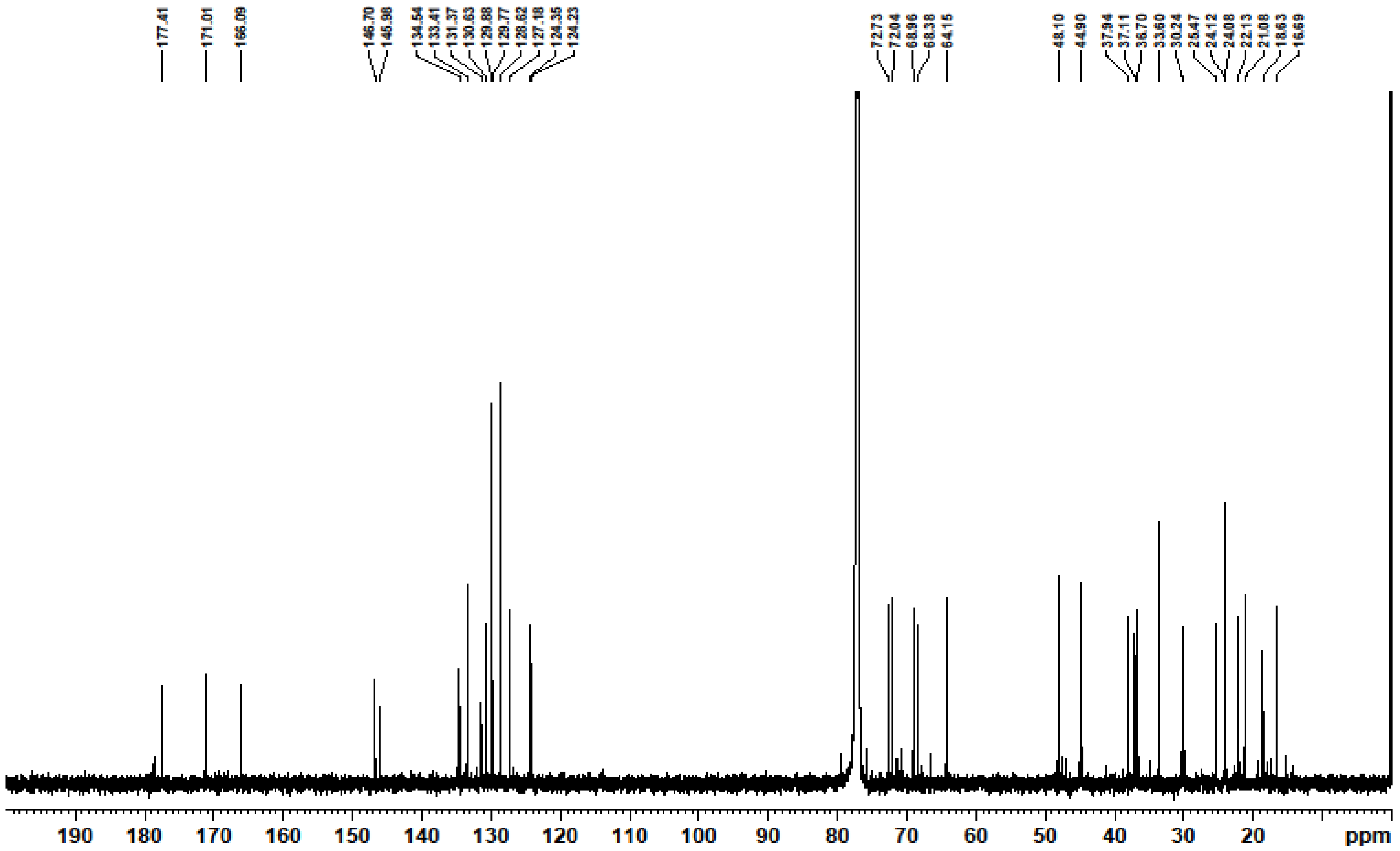

Figure S35. ^{13}C NMR (150 MHz) spectrum of compound **4** in $CDCl_3$

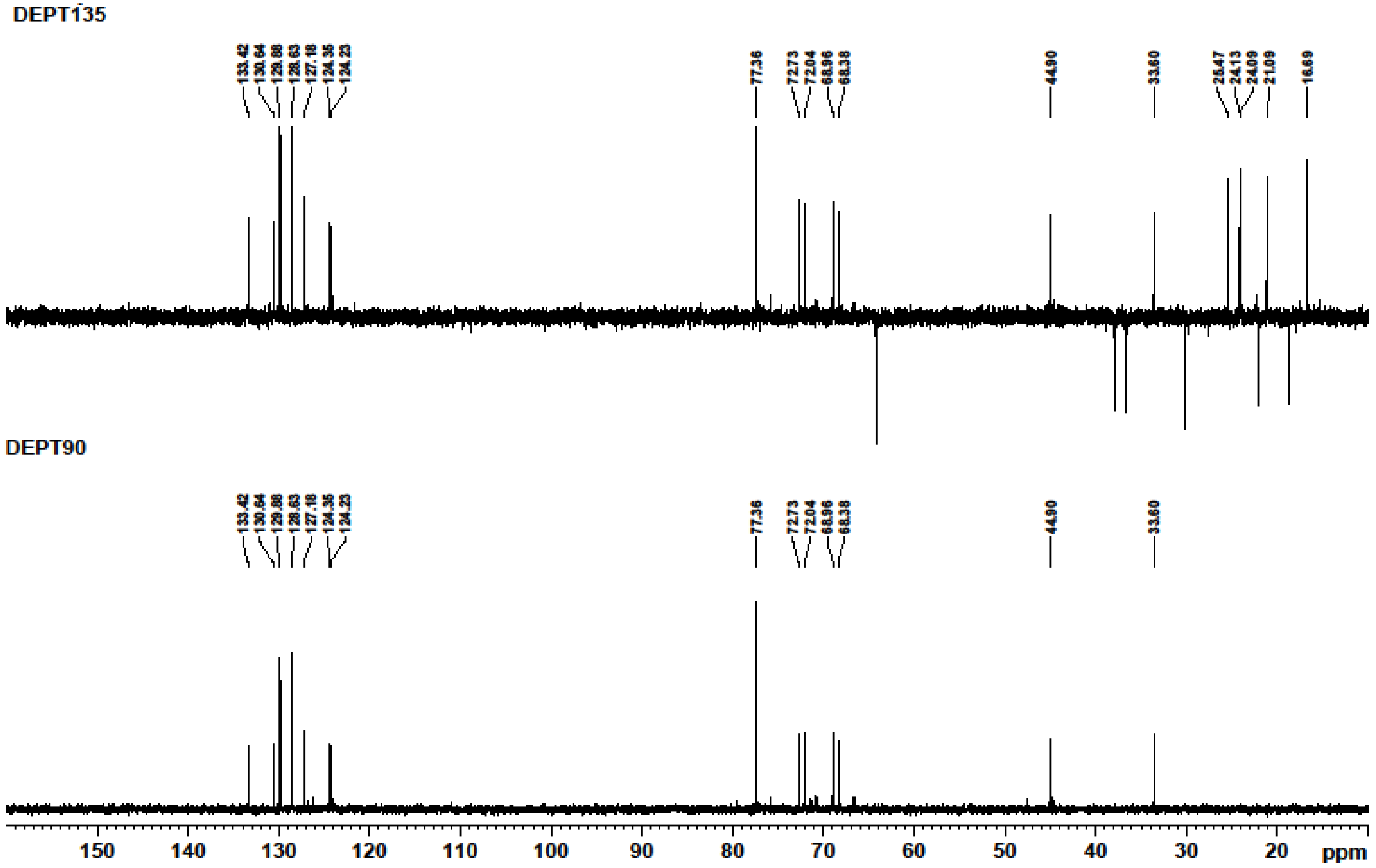

Figure S36. DEPT 135 & 90 NMR spectrum of compound **4** in $CDCl_3$

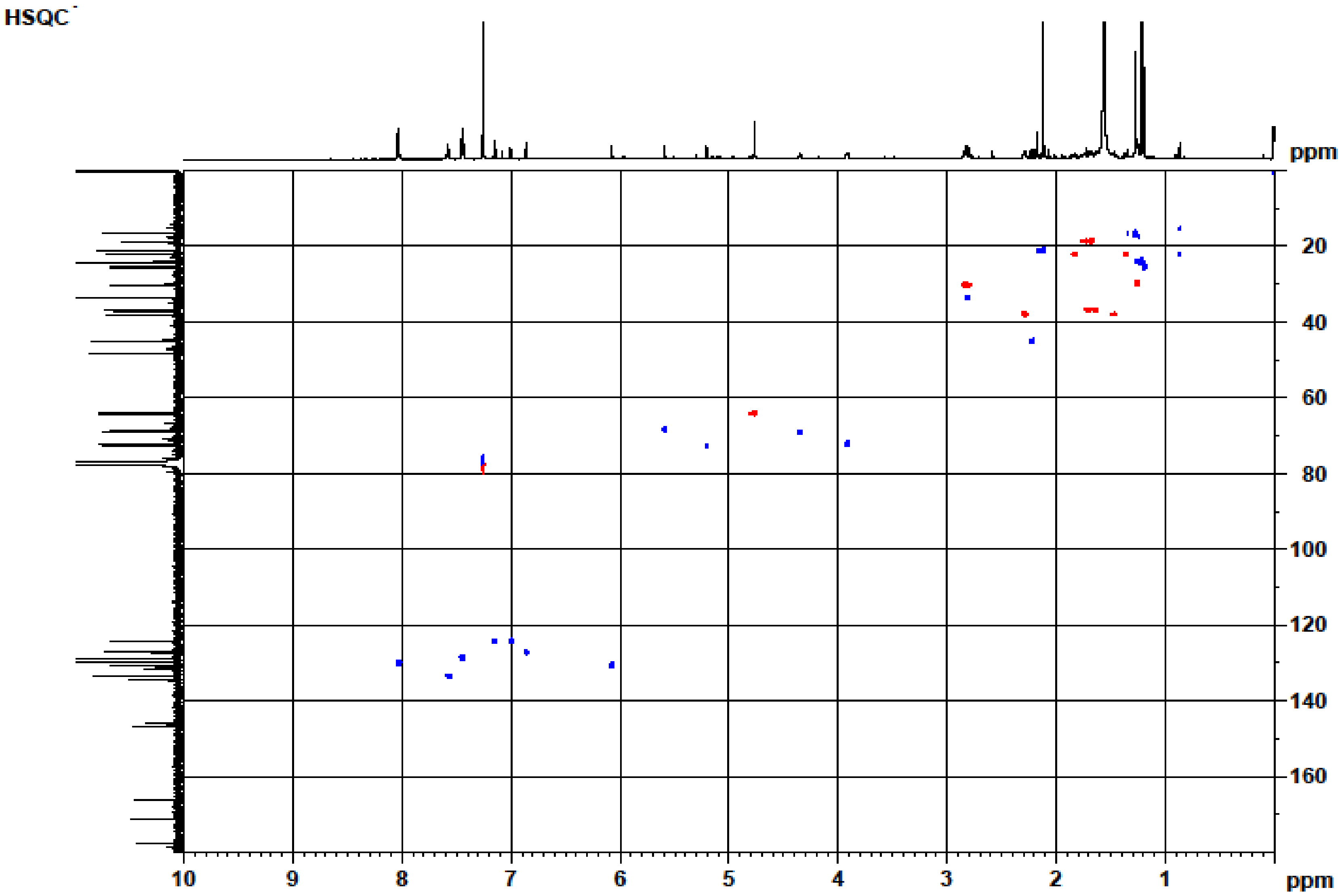

Figure S37. HSQC spectrum of compound 4 in $CDCl_3$

COSY

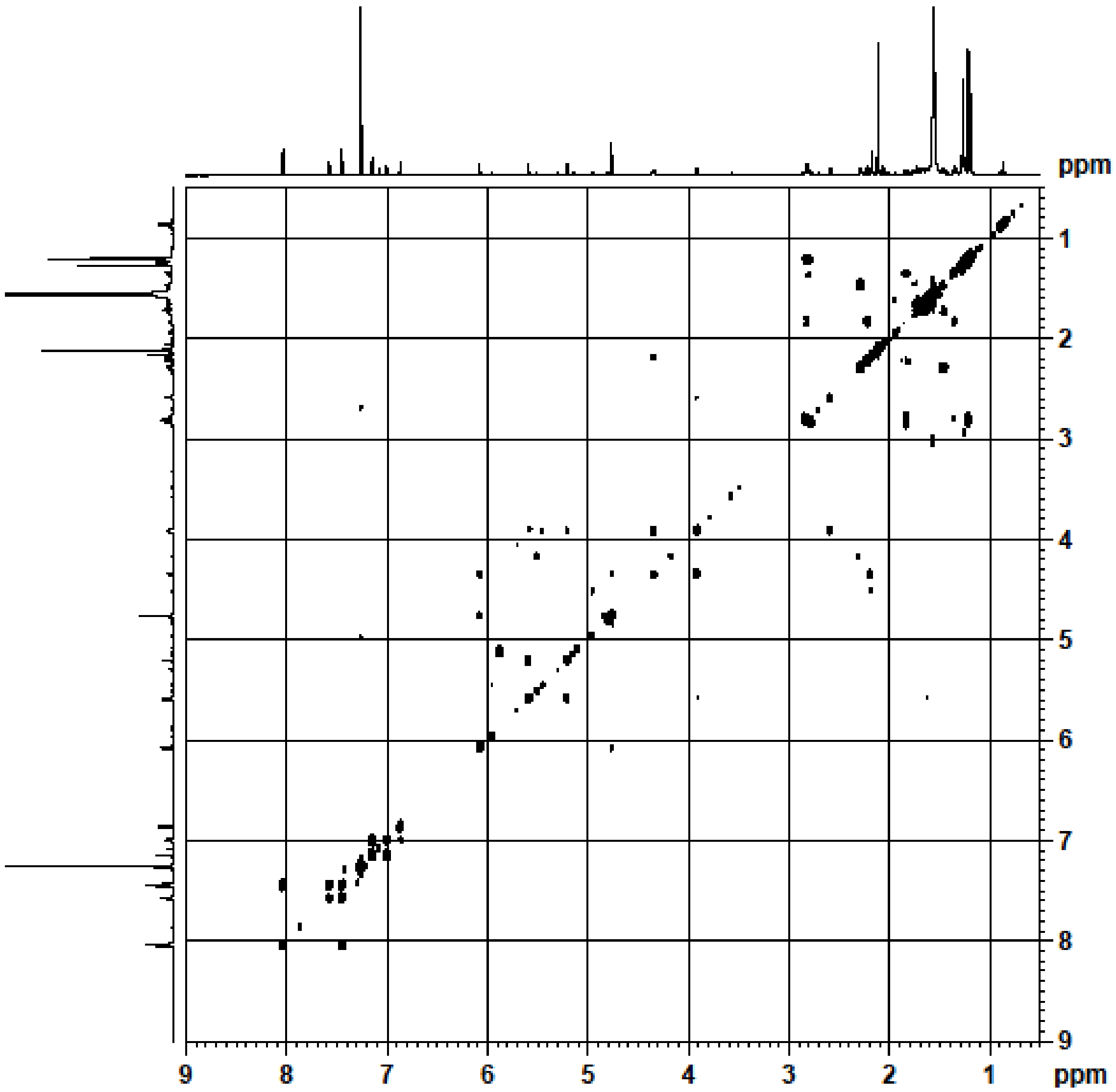

Figure S38. COSY spectrum of compound **4** in $CDCl_3$

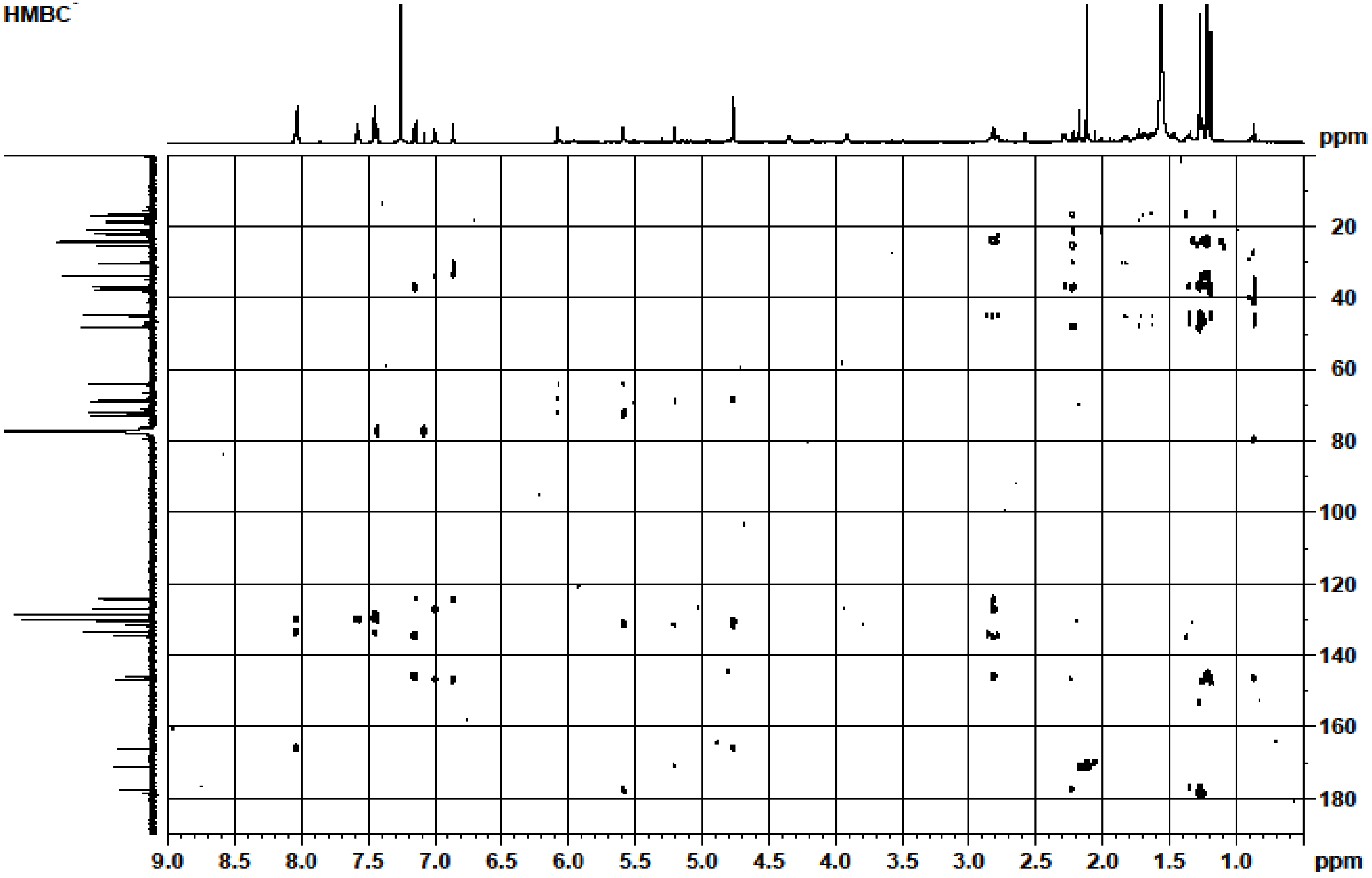

Figure S39. HMBC spectrum of compound **4** in $CDCl_3$

NOESY

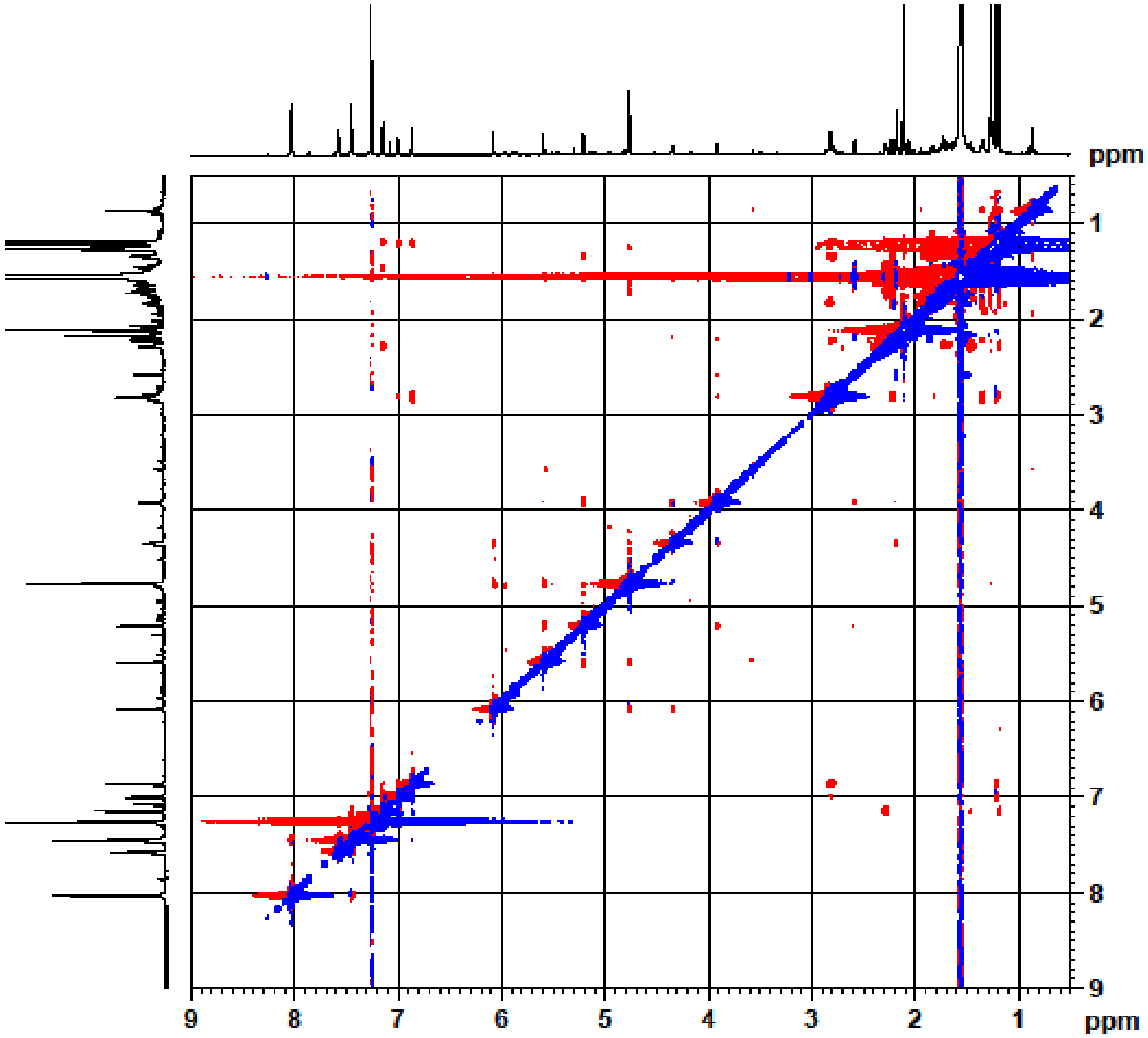

Figure S40. NOESY spectrum of **4** in $CDCl_3$

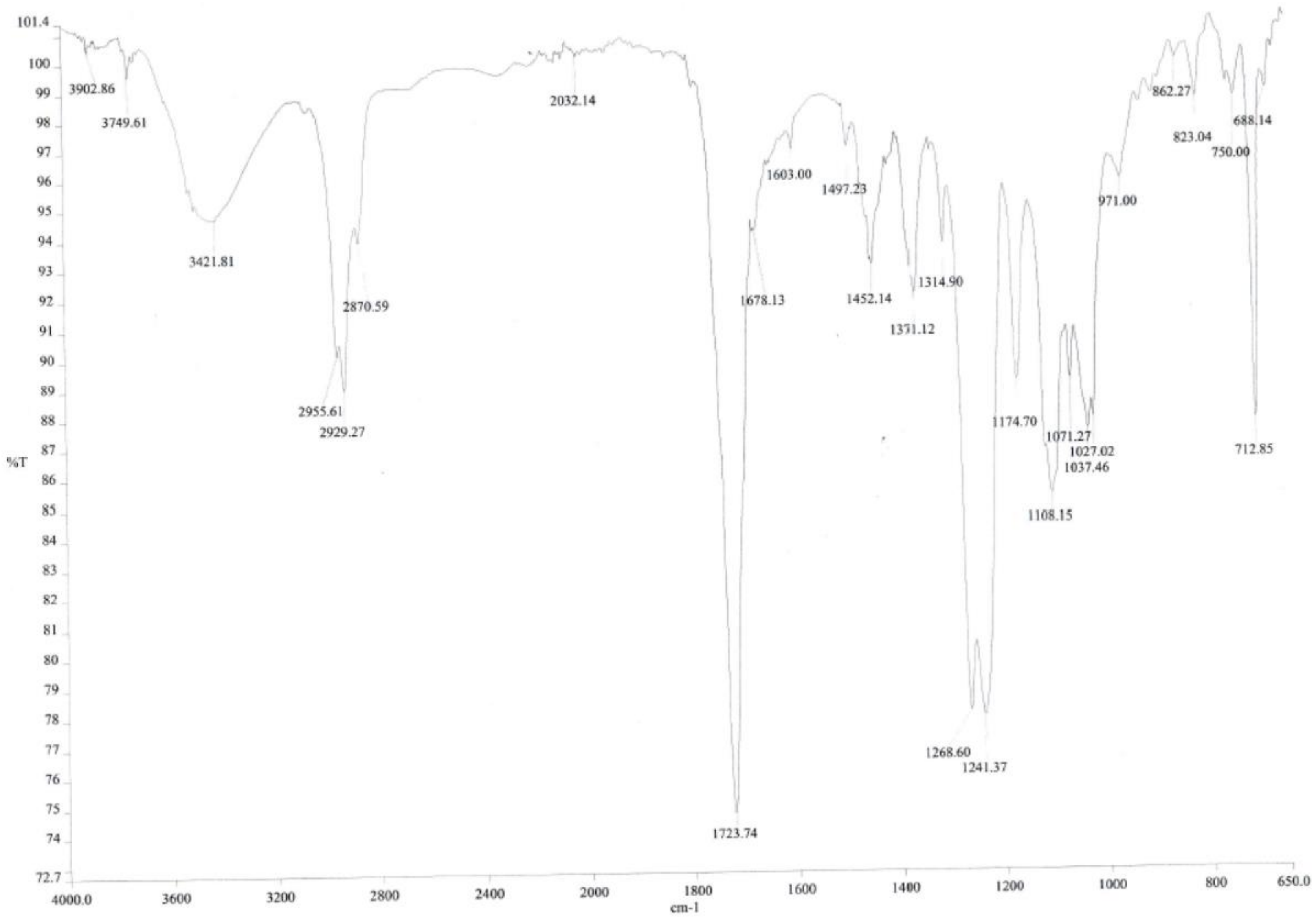

Figure S41. IR spectrum of compound **4**

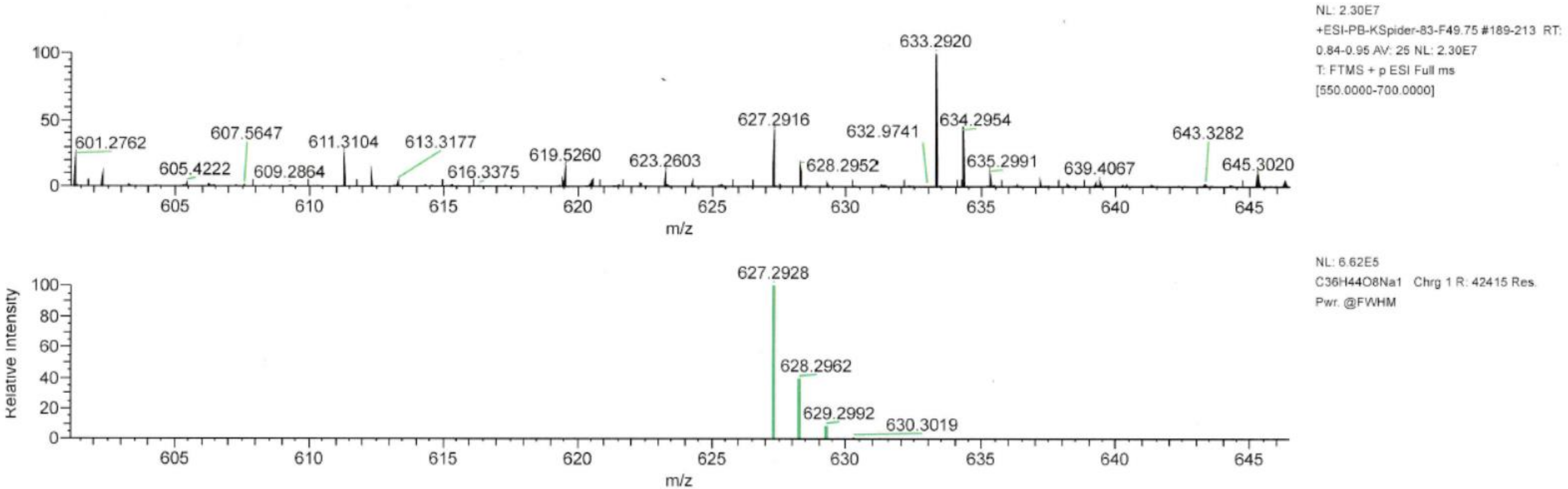

Peak Mass	Display Formula	Delta [ppm]	Theo. mass	MSMS Matched Fragments
627.2916	$C_{45}H_{39}O_3$	3.60	627.28937	(Collection)
627.2916	$C_{36}H_{44}O_8{}^{23}Na$ [M+Na]$^+$	-1.93	627.29284	(Collection)

Figure S42. HRESIMS spectrum of compound **4**

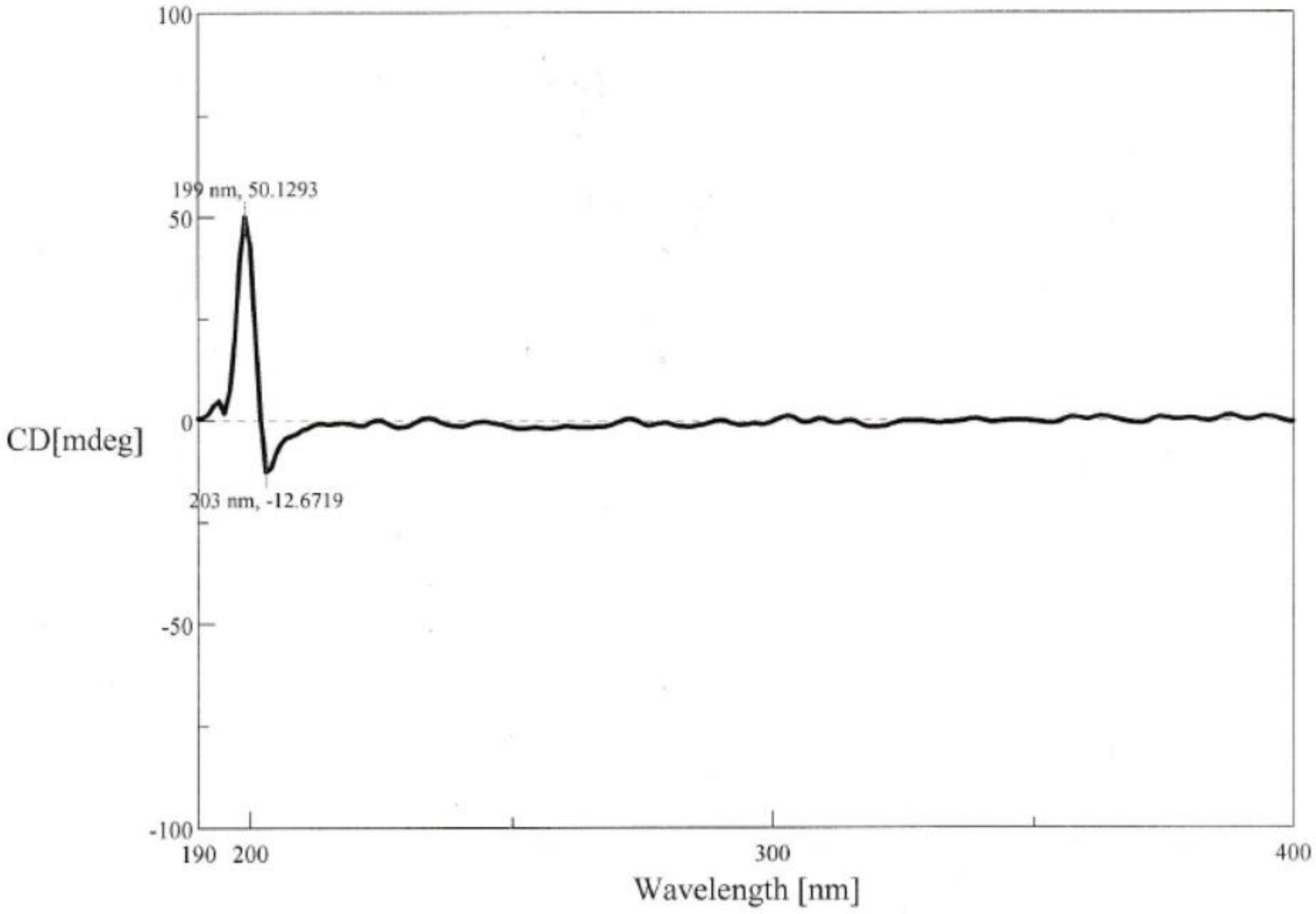

Figure S43. CD spectrum of compound **4**

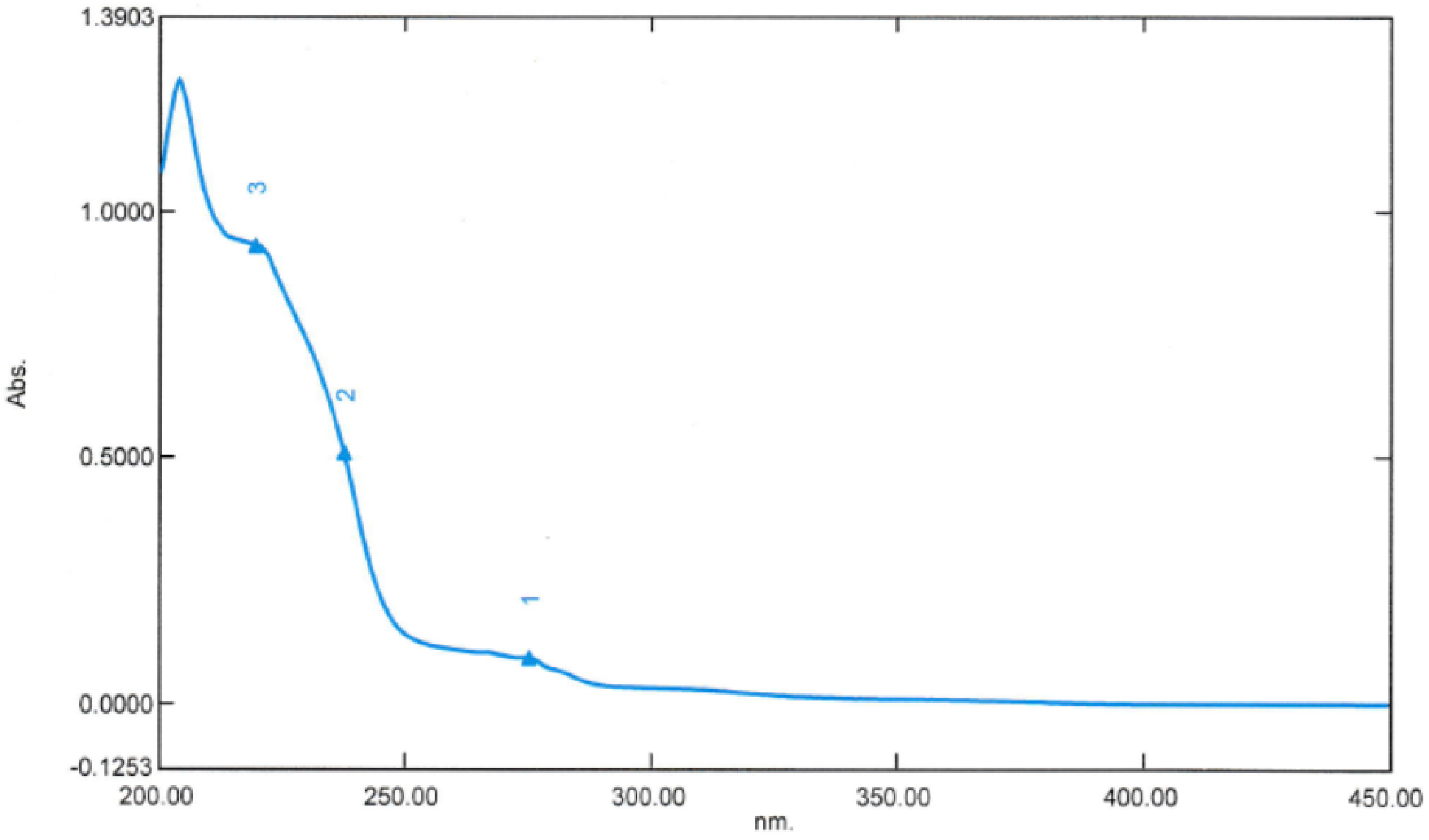

Figure S44. UV spectrum of compound **4**

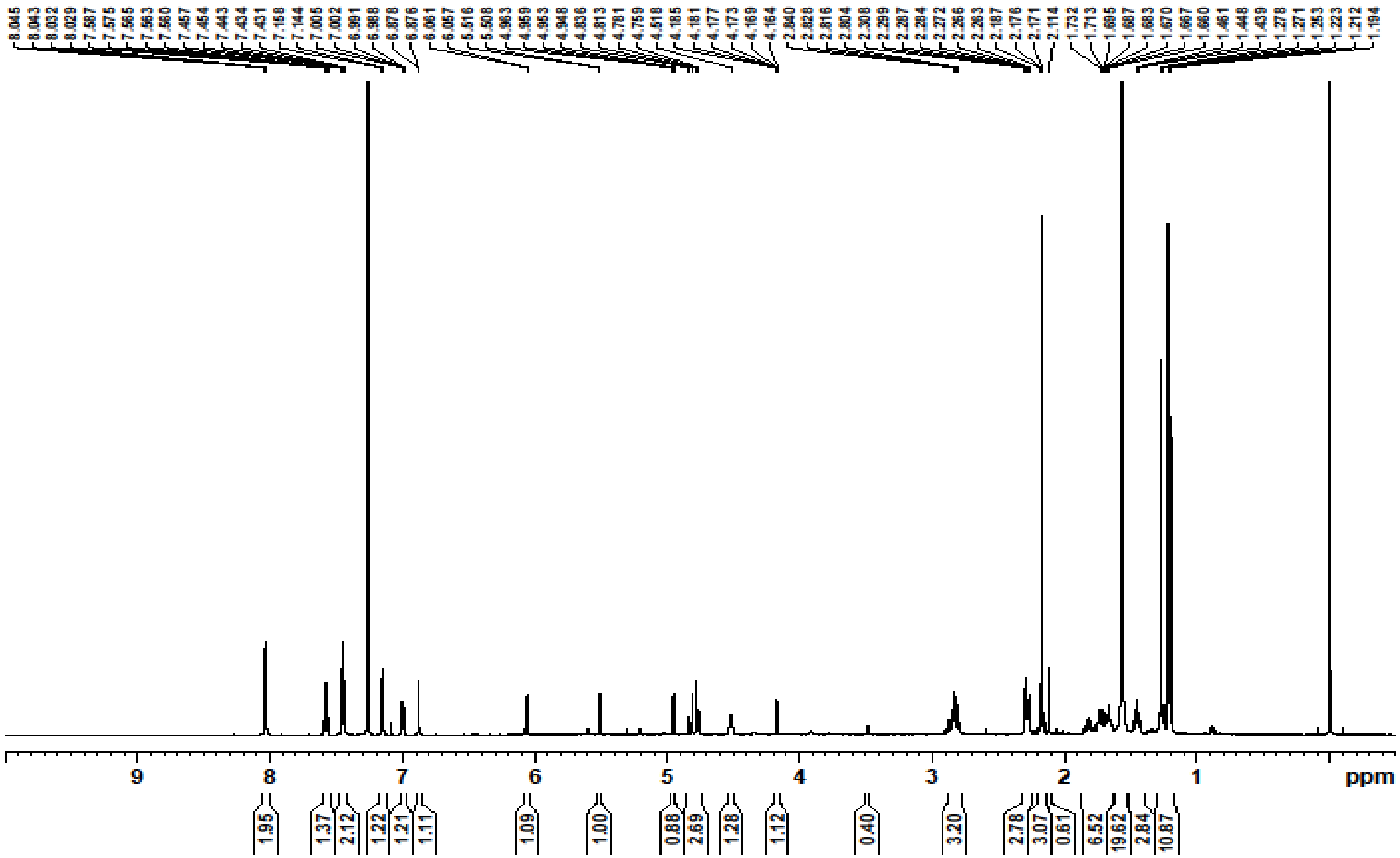

Figure S45. ^{1}H NMR (600 MHz) spectrum of compound **5** in $CDCl_3$

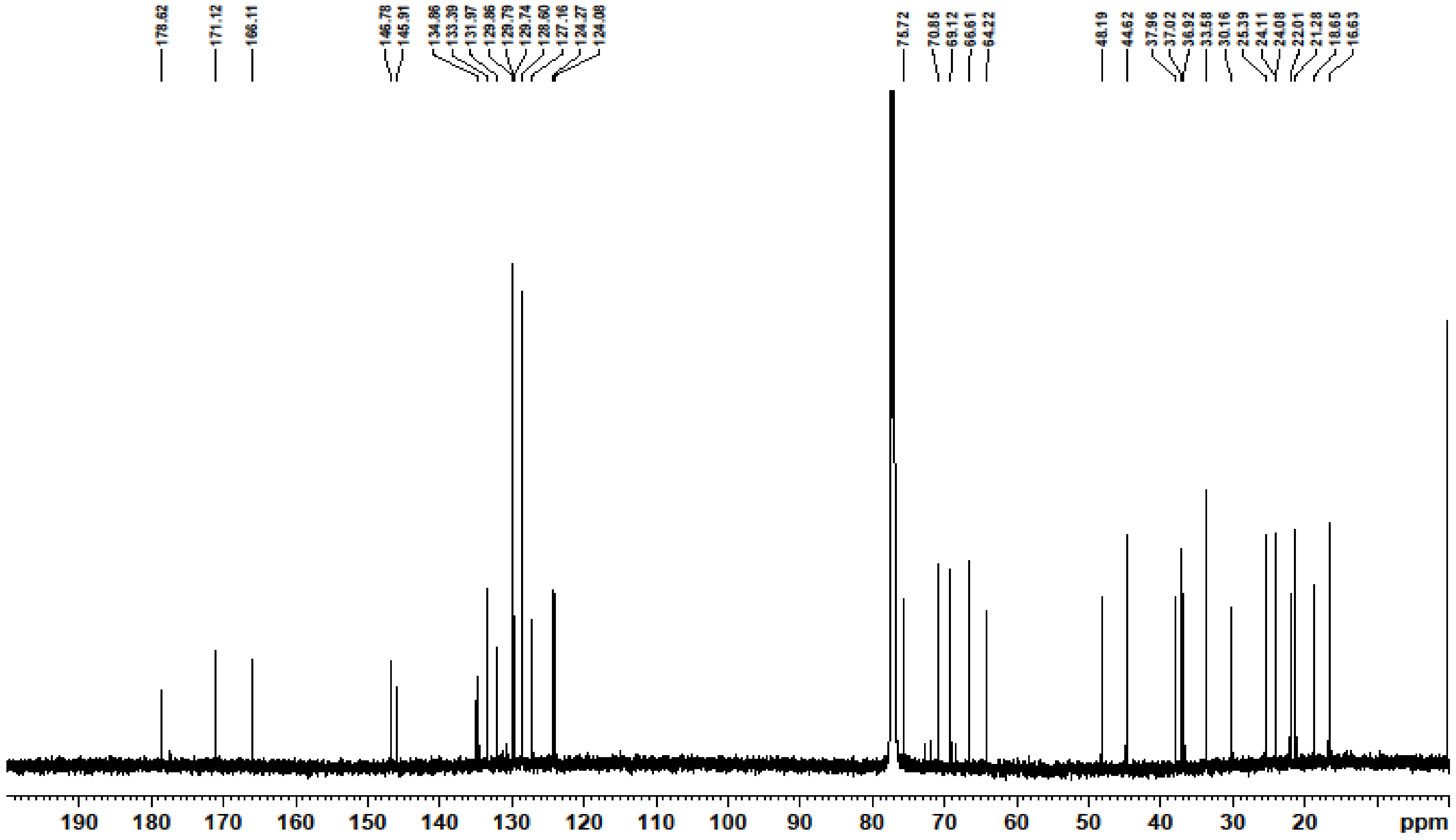

Figure S46. ^{13}C NMR (150 MHz) spectrum of compound **2** in $CDCl_3$

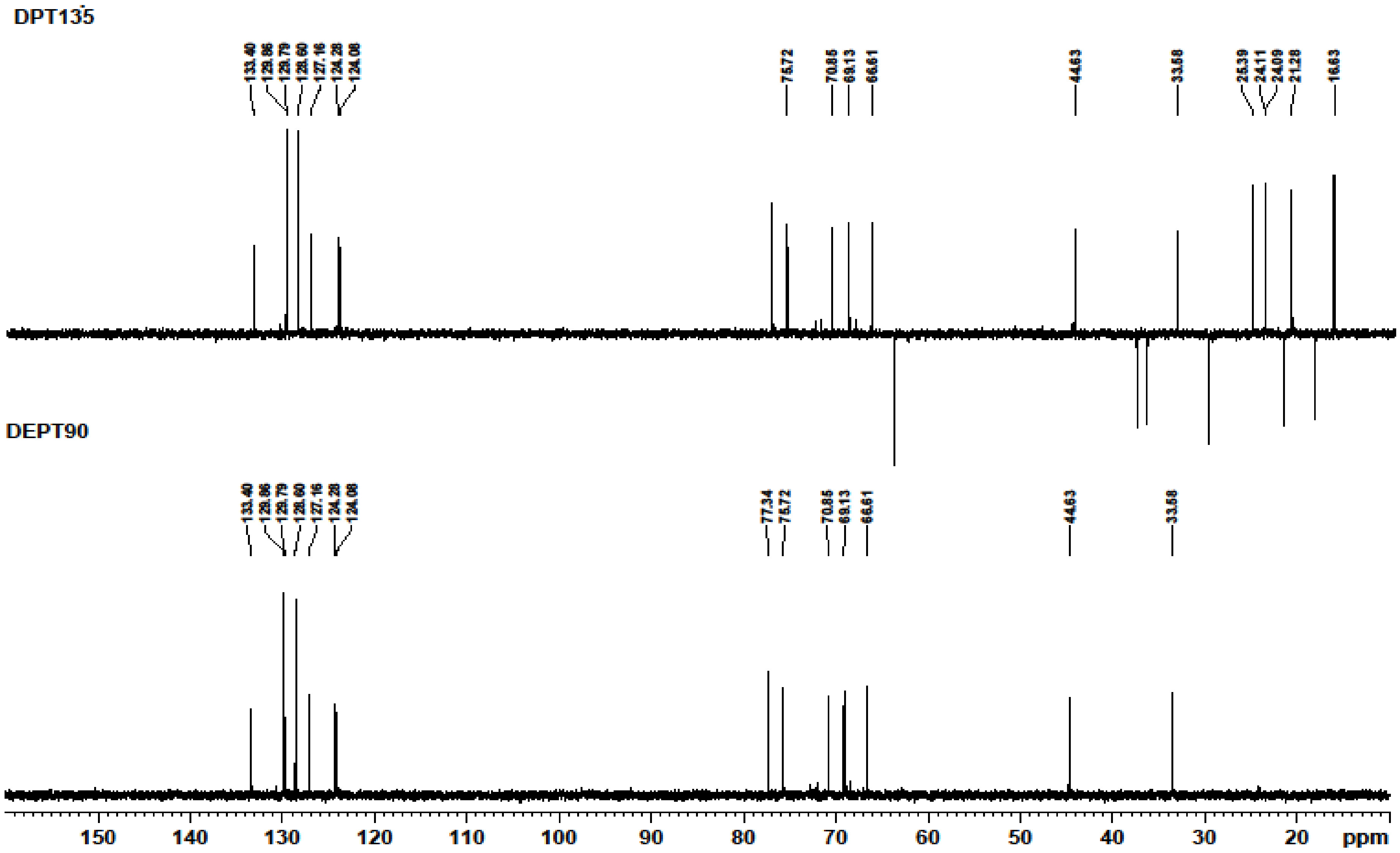

Figure S47. DEPT 135 & 90 NMR spectrum of compound **5** in $CDCl_3$

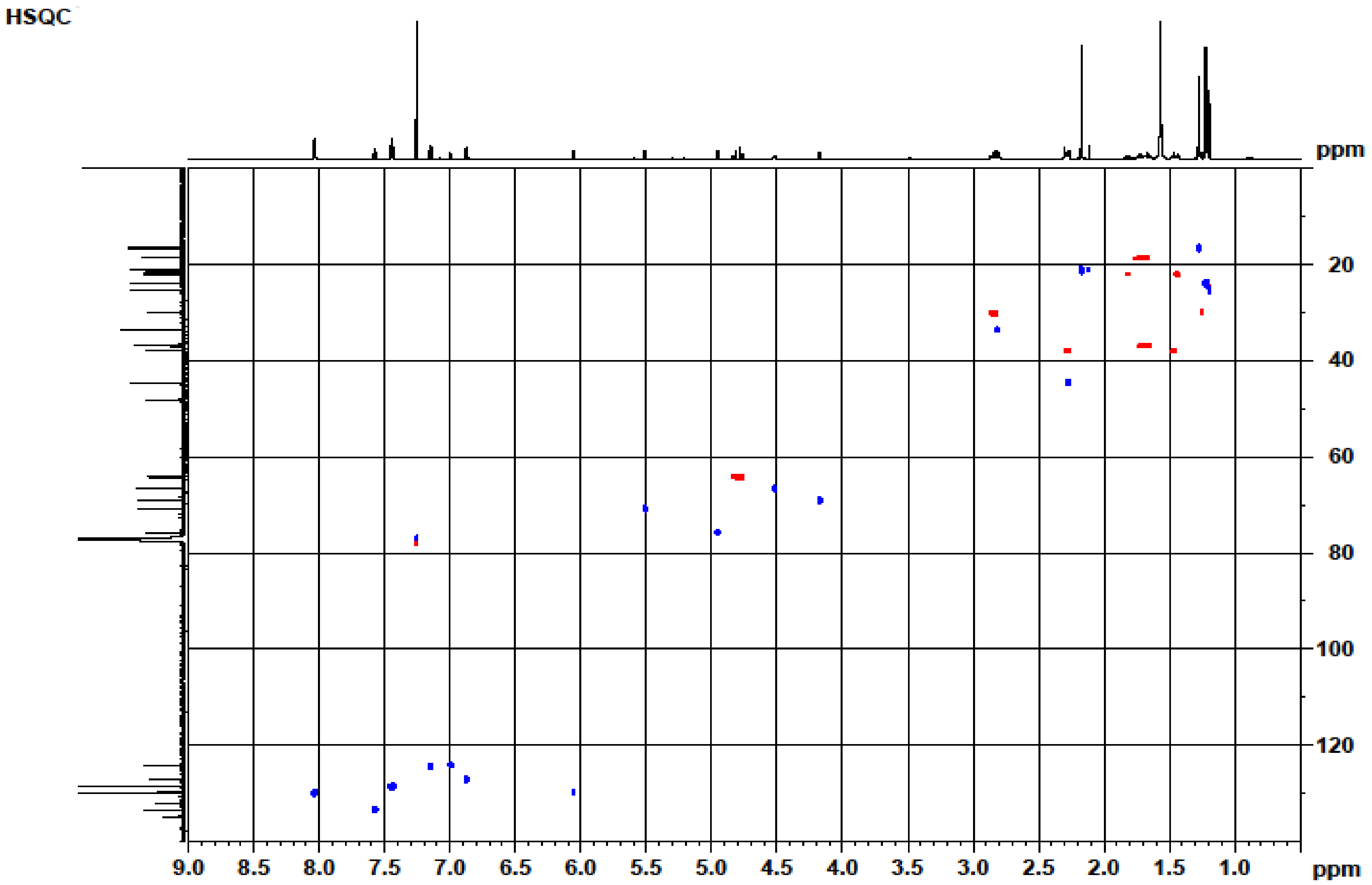

Figure S48. HSQC spectrum of compound **5** in $CDCl_3$

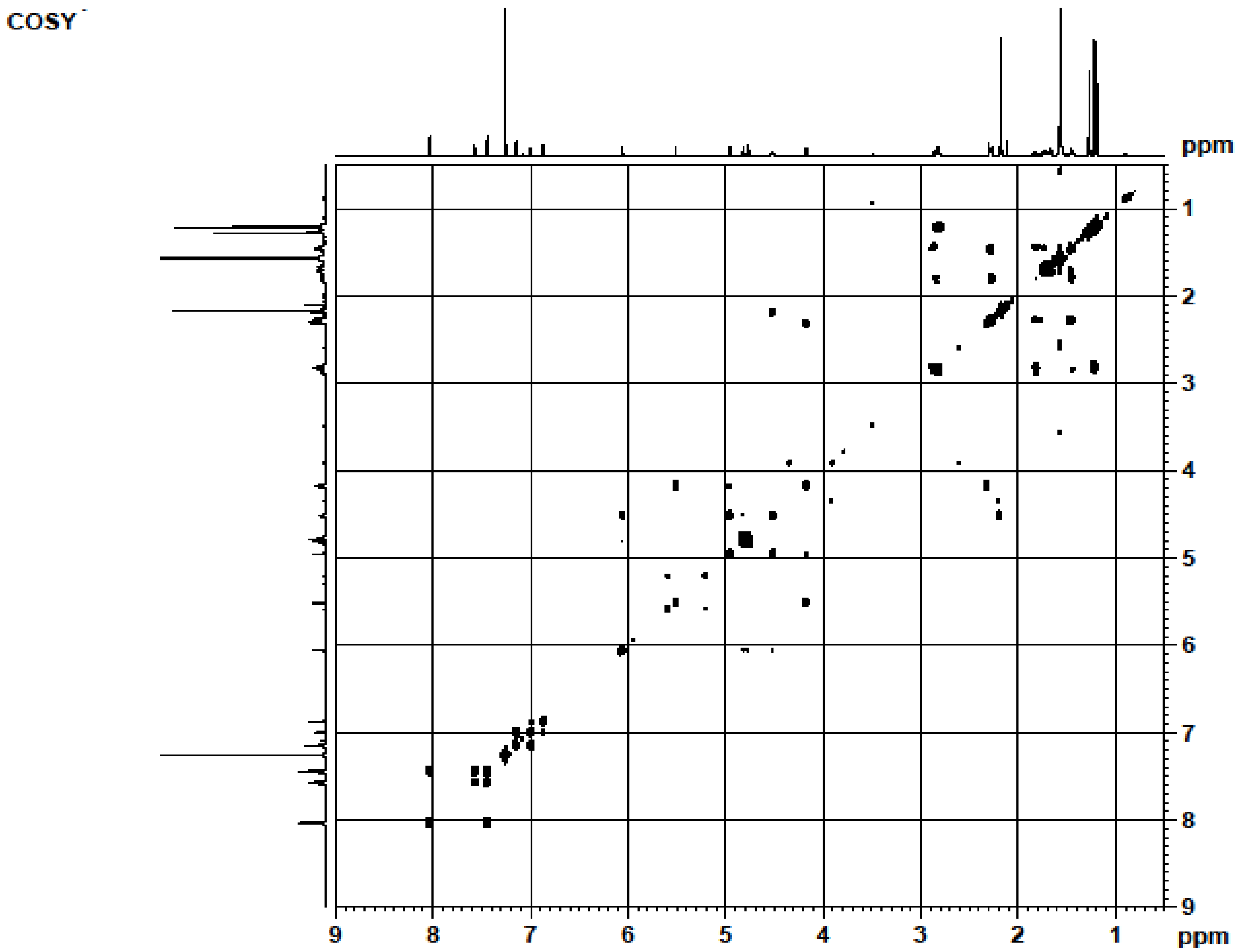

Figure S49. COSY spectrum of compound **5** in $CDCl_3$

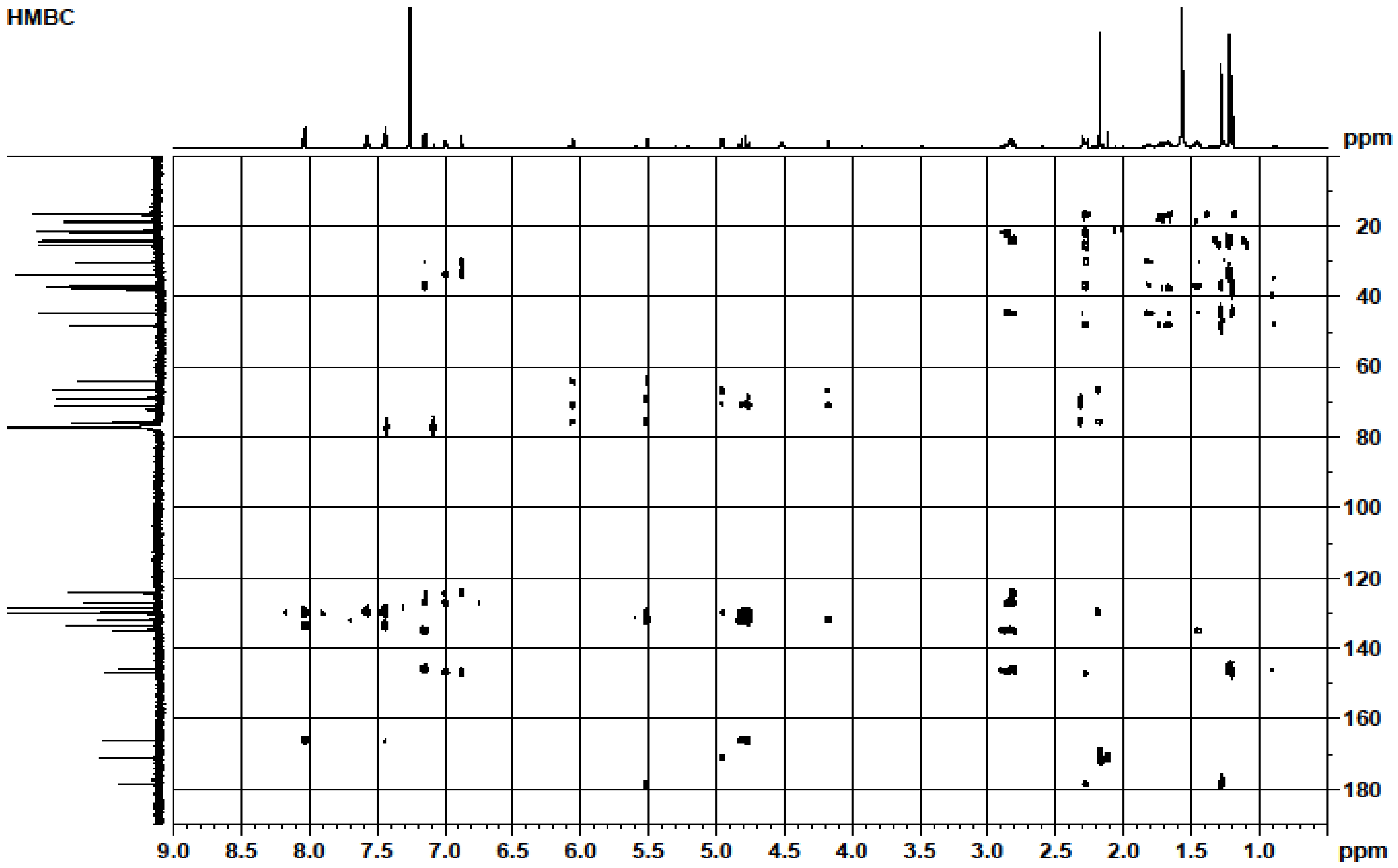

Figure S50. HMBC spectrum of compound **5** in $CDCl_3$

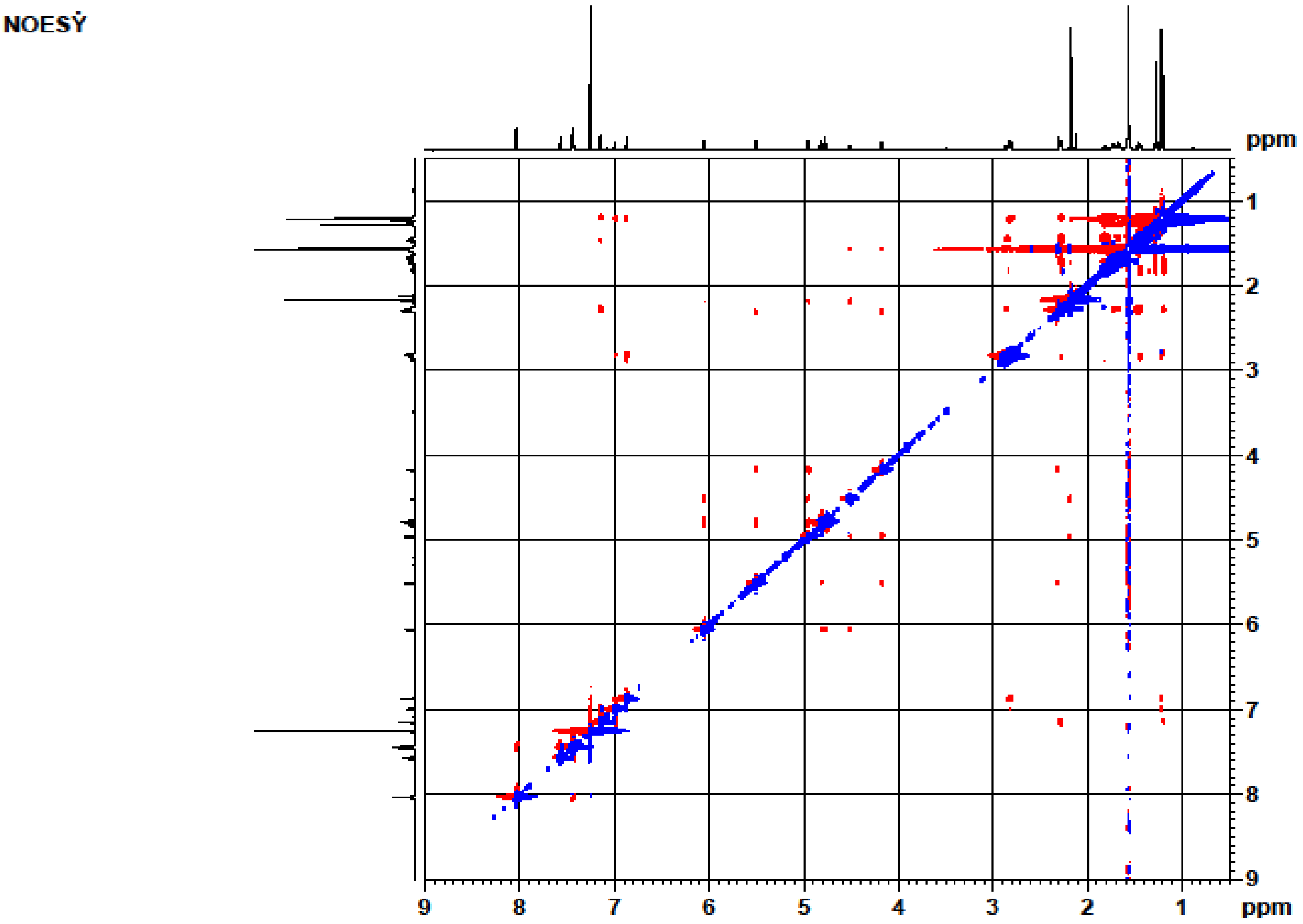

Figure S51. NOESY spectrum of **5** in $CDCl_3$

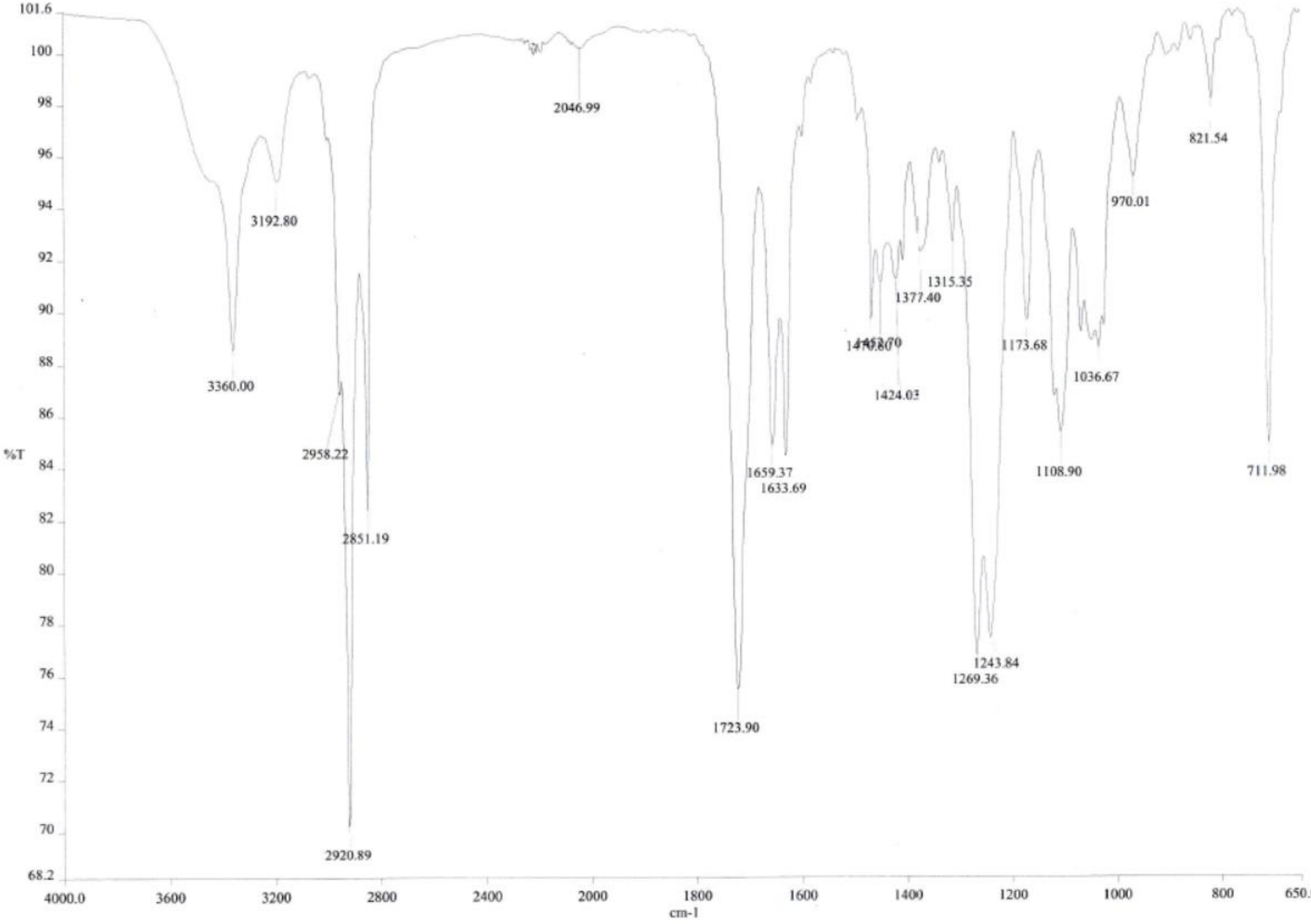

Figure S52. IR spectrum of compound **5**

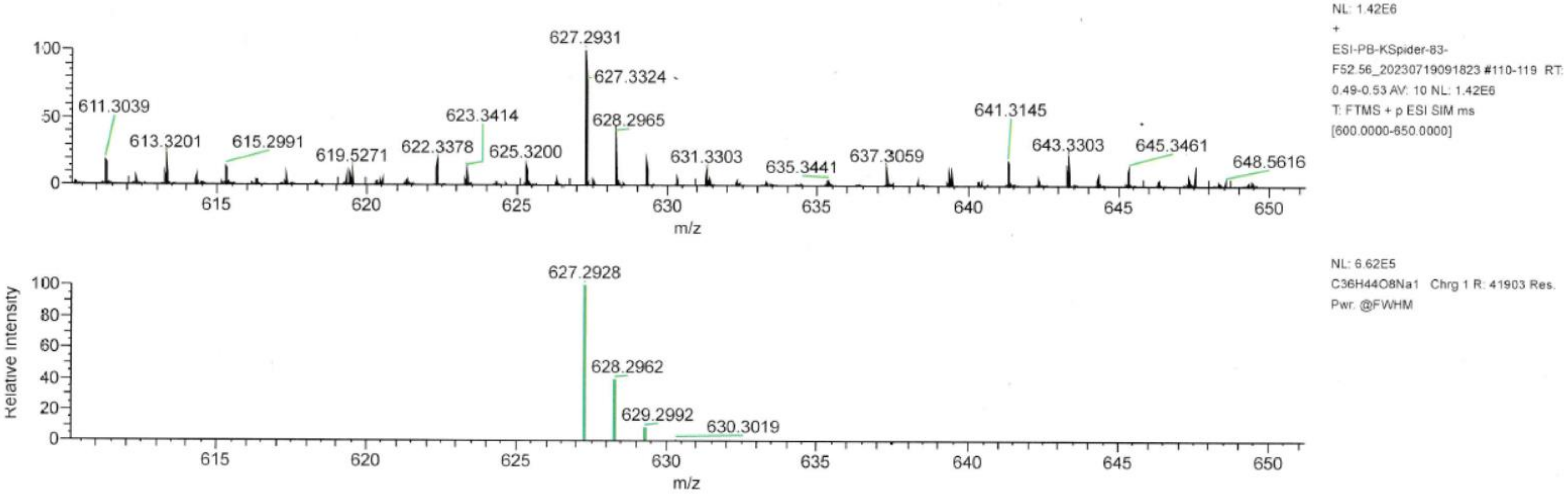

Peak Mass	Display Formula	Delta [ppm]	Theo. mass	MSMS Matched Fragments
627.2931	$C_{20}H_{51}O_{21}$	2.21	627.29173	(Collection)
627.2931	$C_{36}H_{44}O_{8}{}^{23}Na$ [M+Na]+	0.45	627.29284	(Collection)
627.2931	$C_{38}H_{43}O_{8}$	-3.38	627.29524	(Collection)
627.2931	$C_{11}H_{56}O_{26}{}^{23}Na$	-3.31	627.29520	(Collection)

Figure S53. HRESIMS spectrum of compound **5**

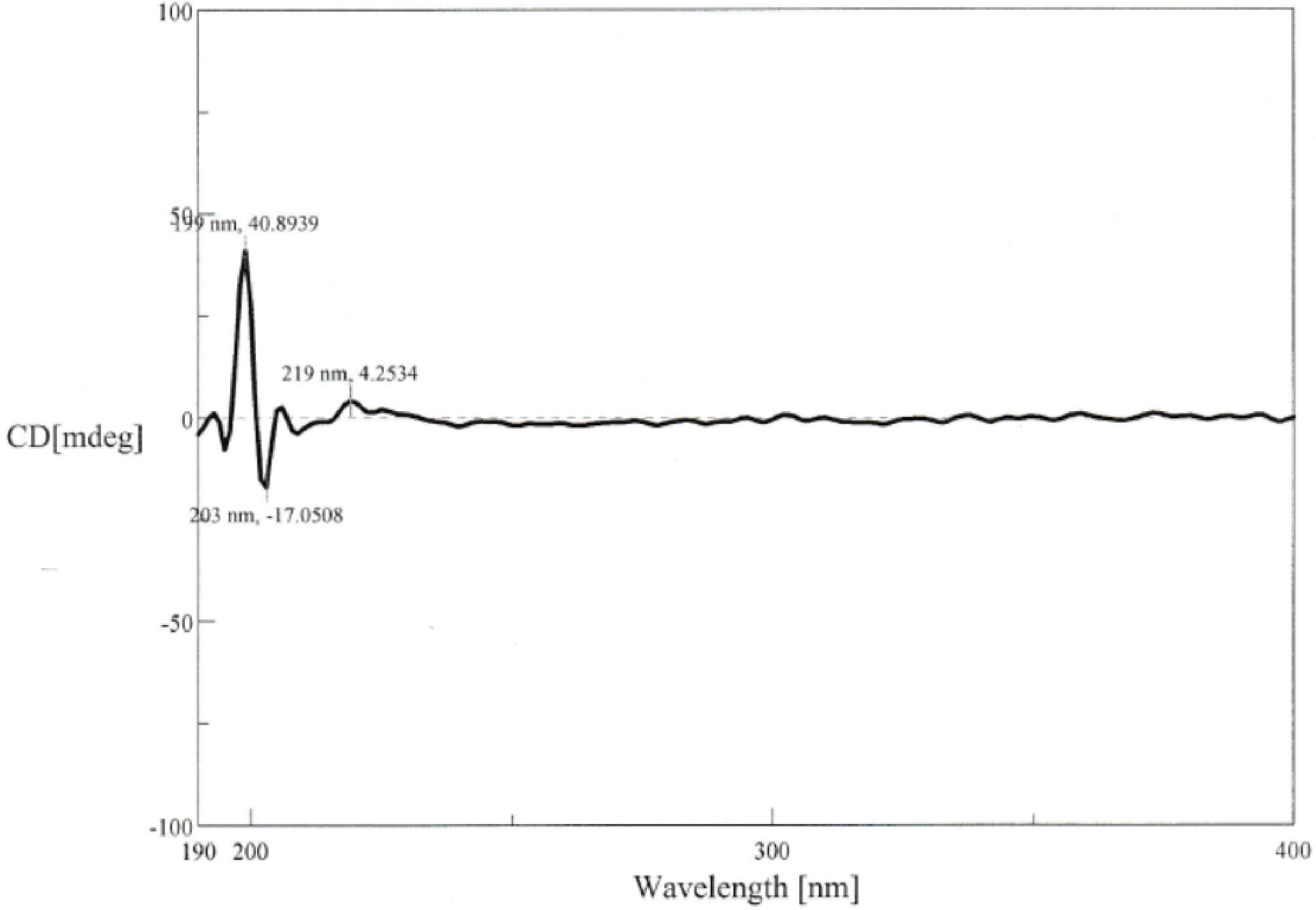

Figure S54. CD spectrum of compound **5**

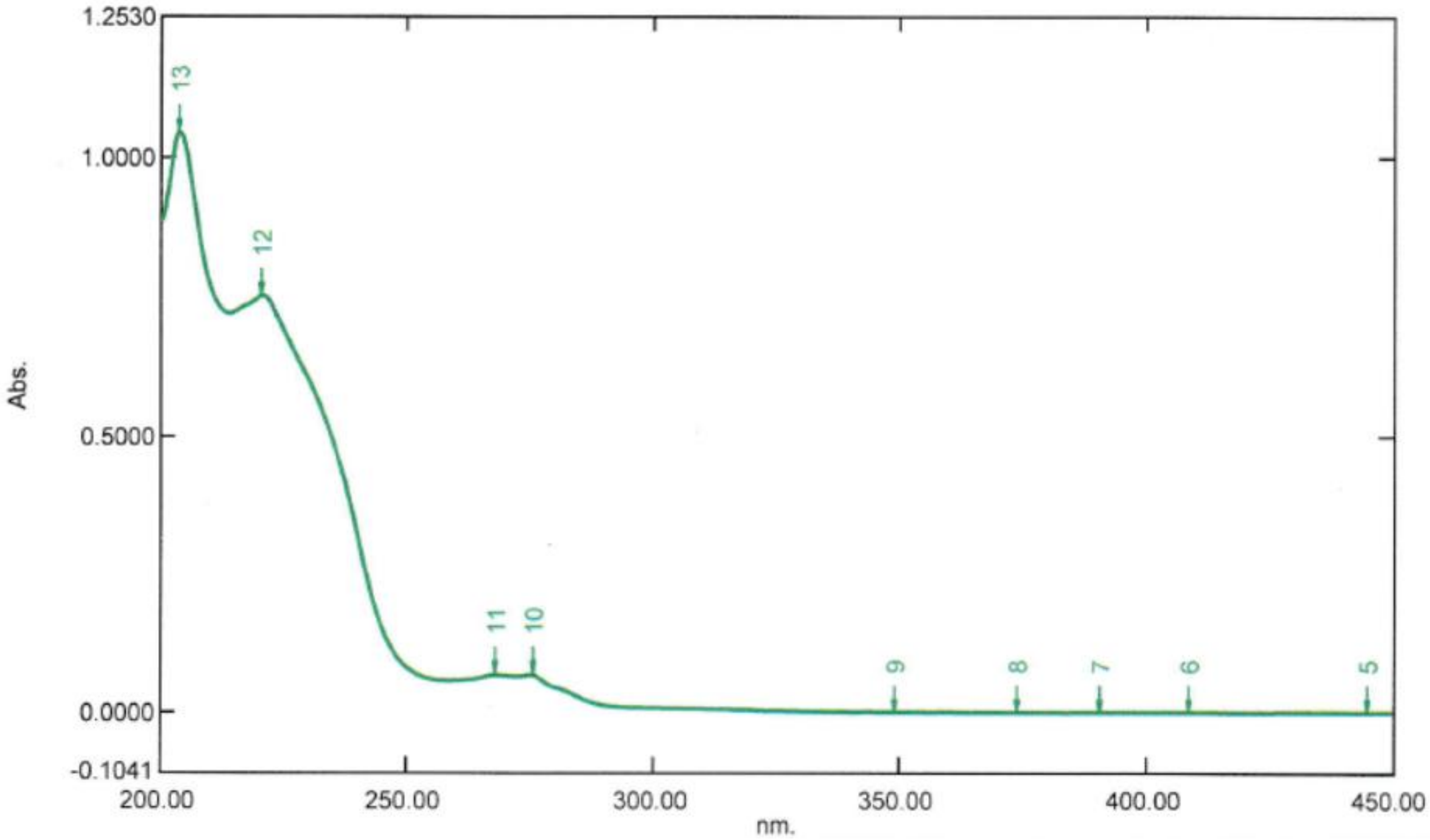

Figure S55. UV spectrum of compound **5**

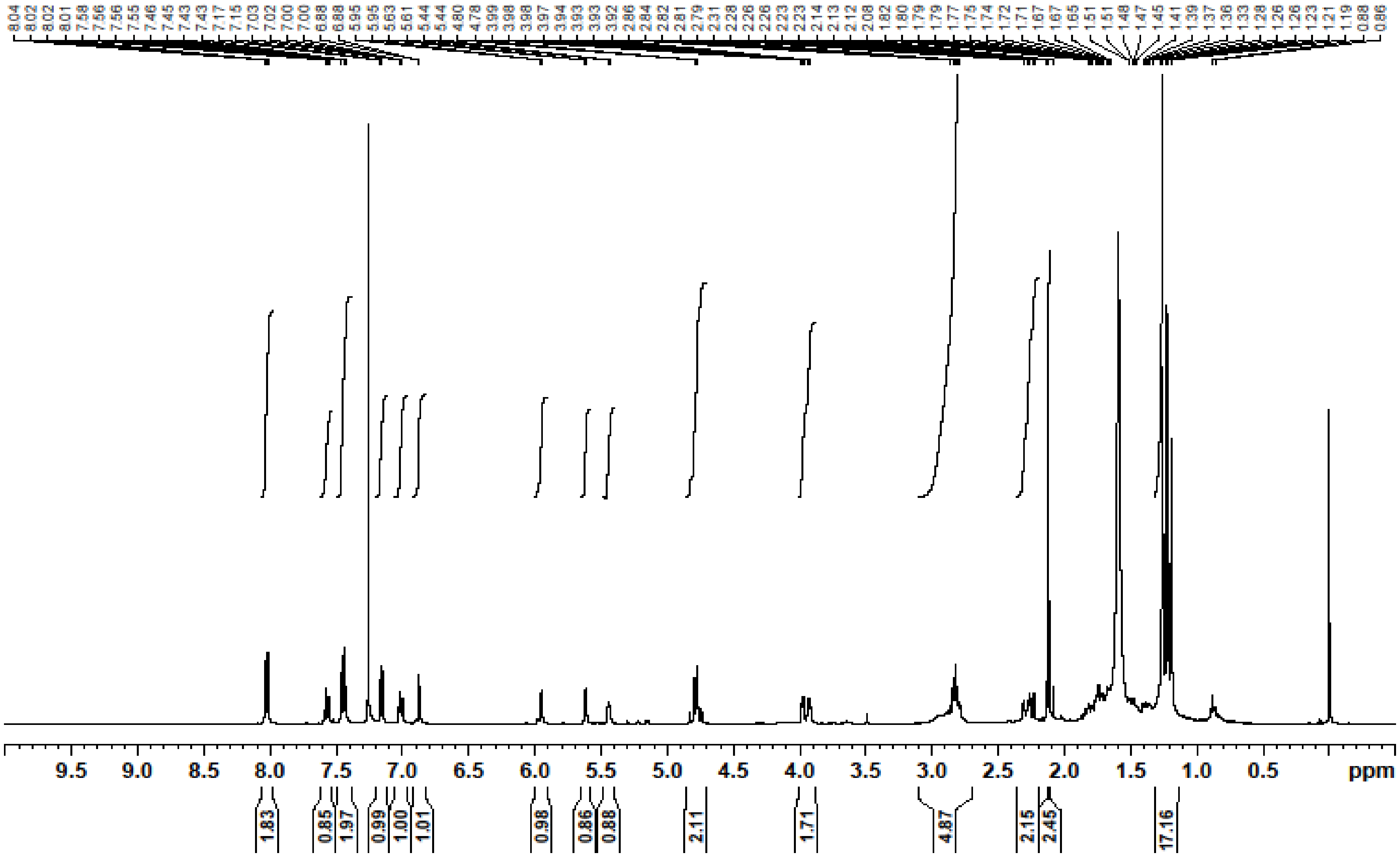

Figure S56. ^{1}H NMR (600 MHz) spectrum of compound **6** in $CDCl_3$

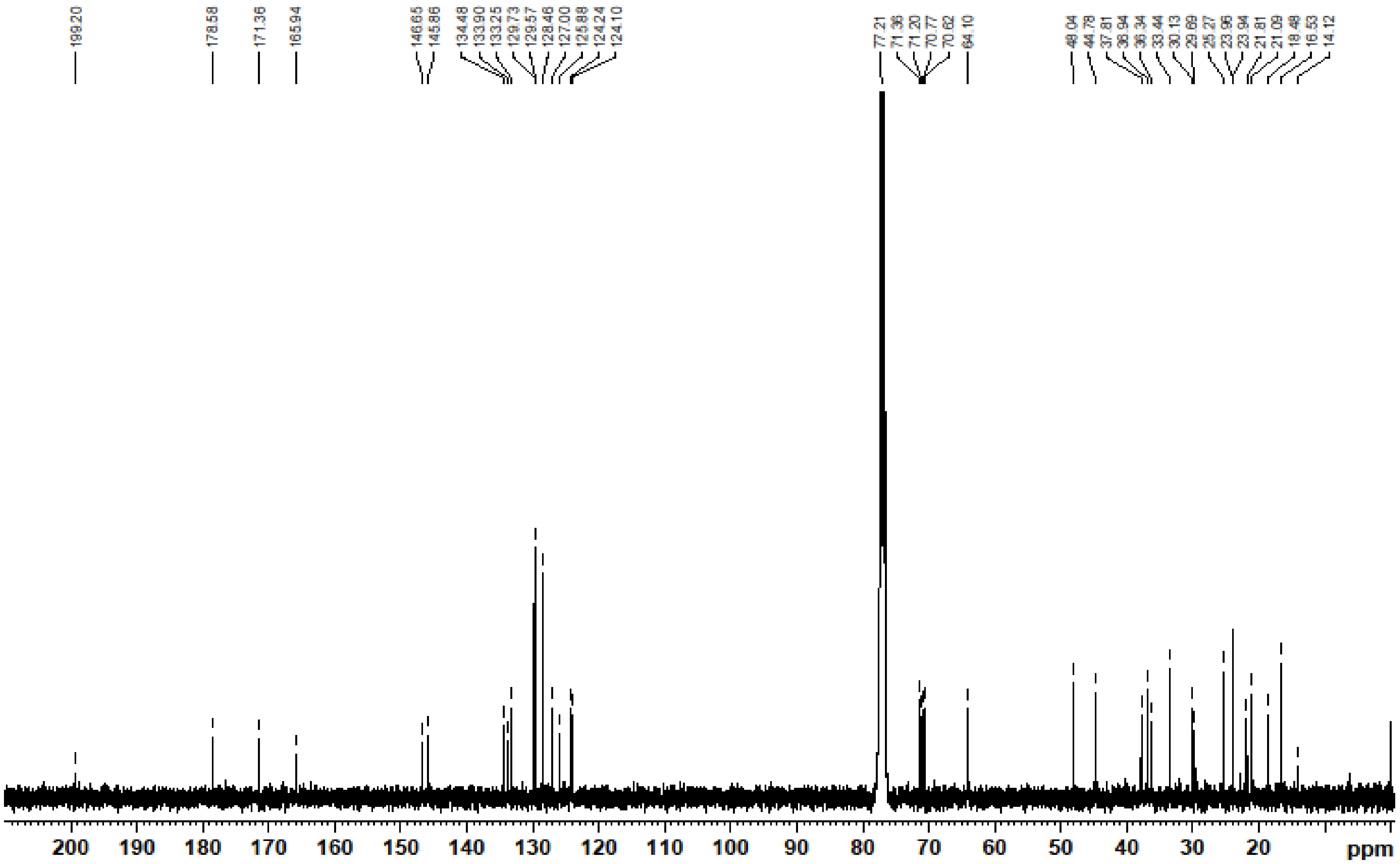

Figure S57. ^{13}C NMR (150 MHz) spectrum of compound **6** in $CDCl_3$

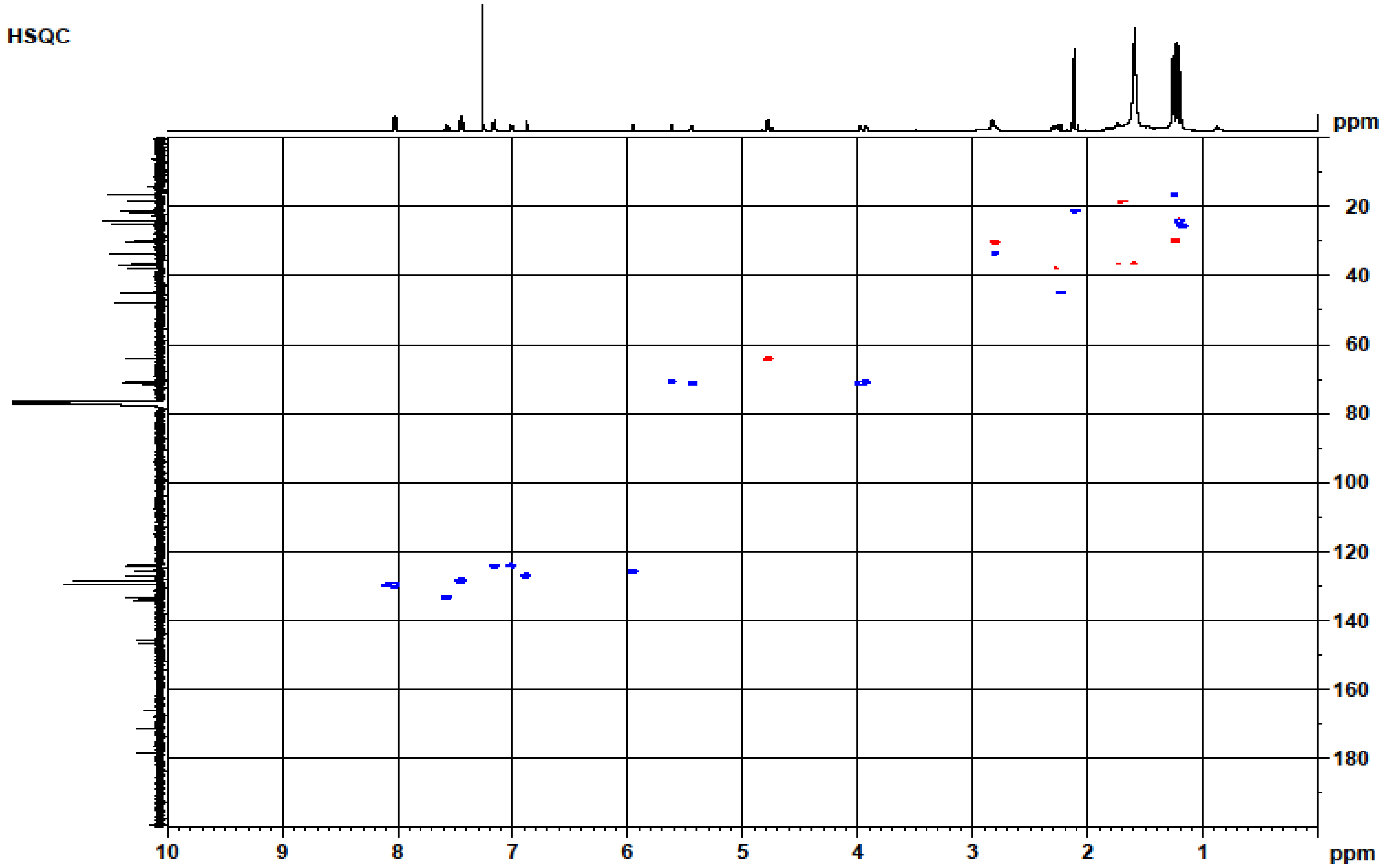

Figure S58. HSQC spectrum of compound **6** in $CDCl_3$

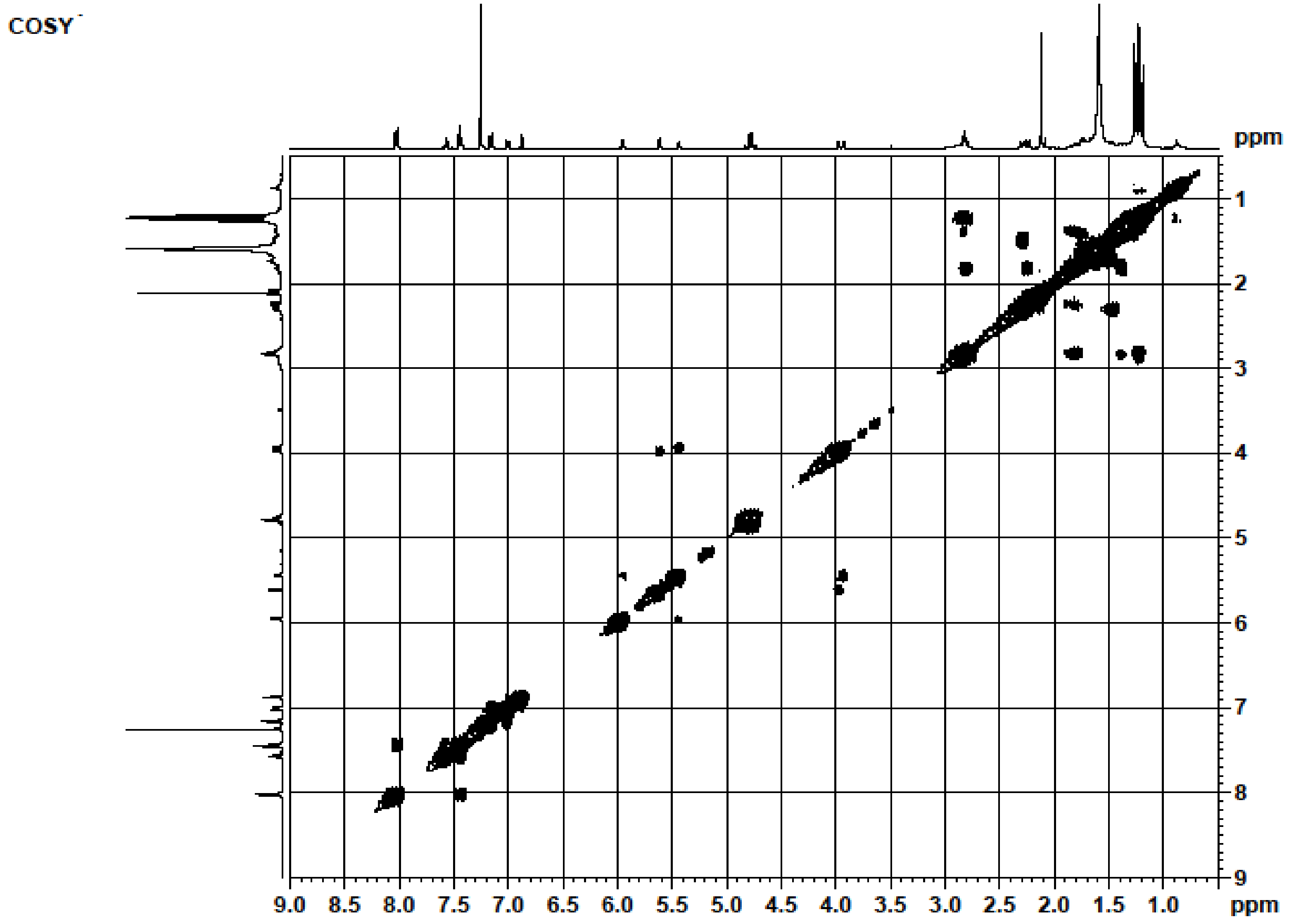

Figure S59. COSY spectrum of compound **6** in $CDCl_3$

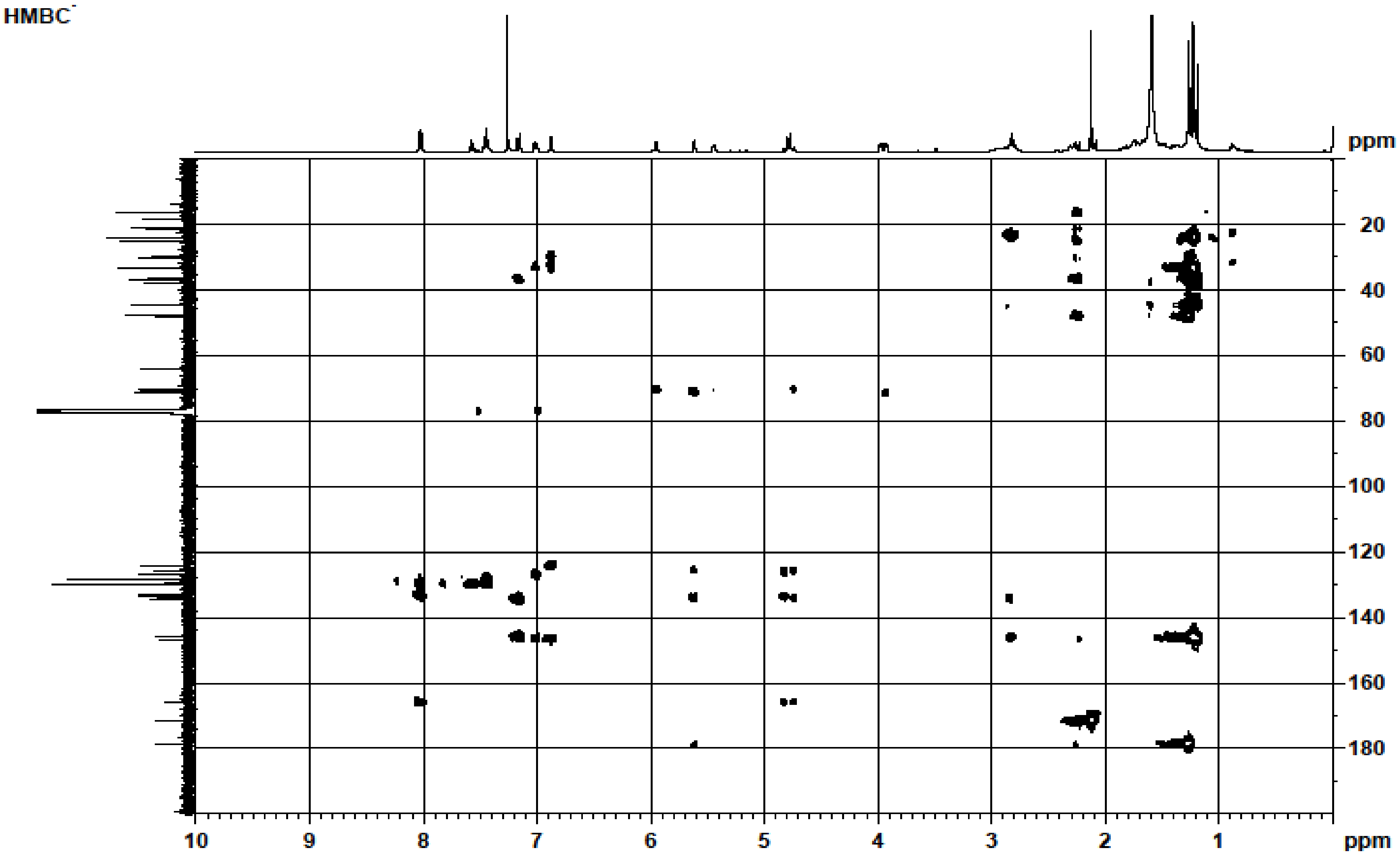

Figure S60. HMBC spectrum of compound **6** in $CDCl_3$

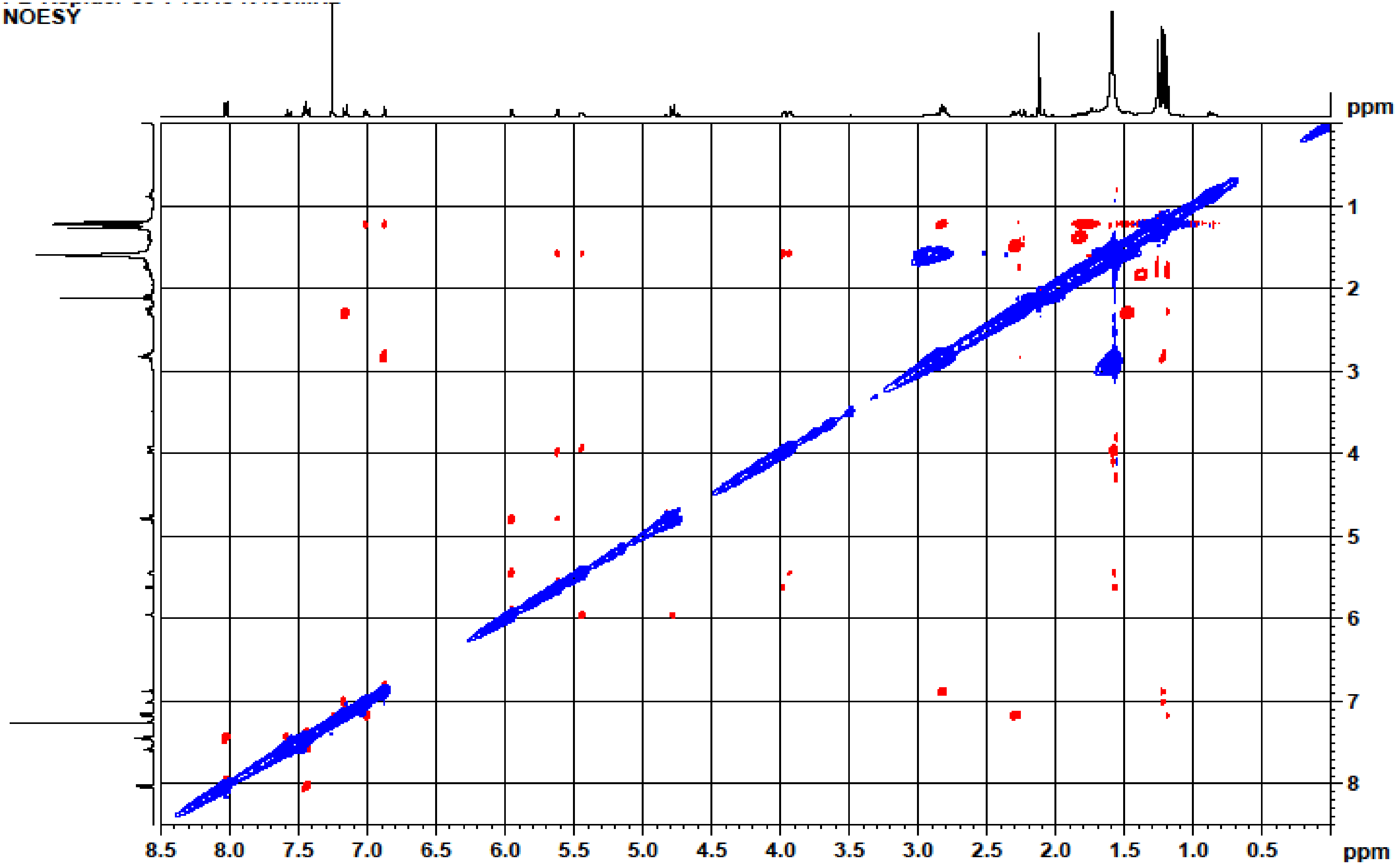

Figure S61. NOESY spectrum of **6** in $CDCl_3$

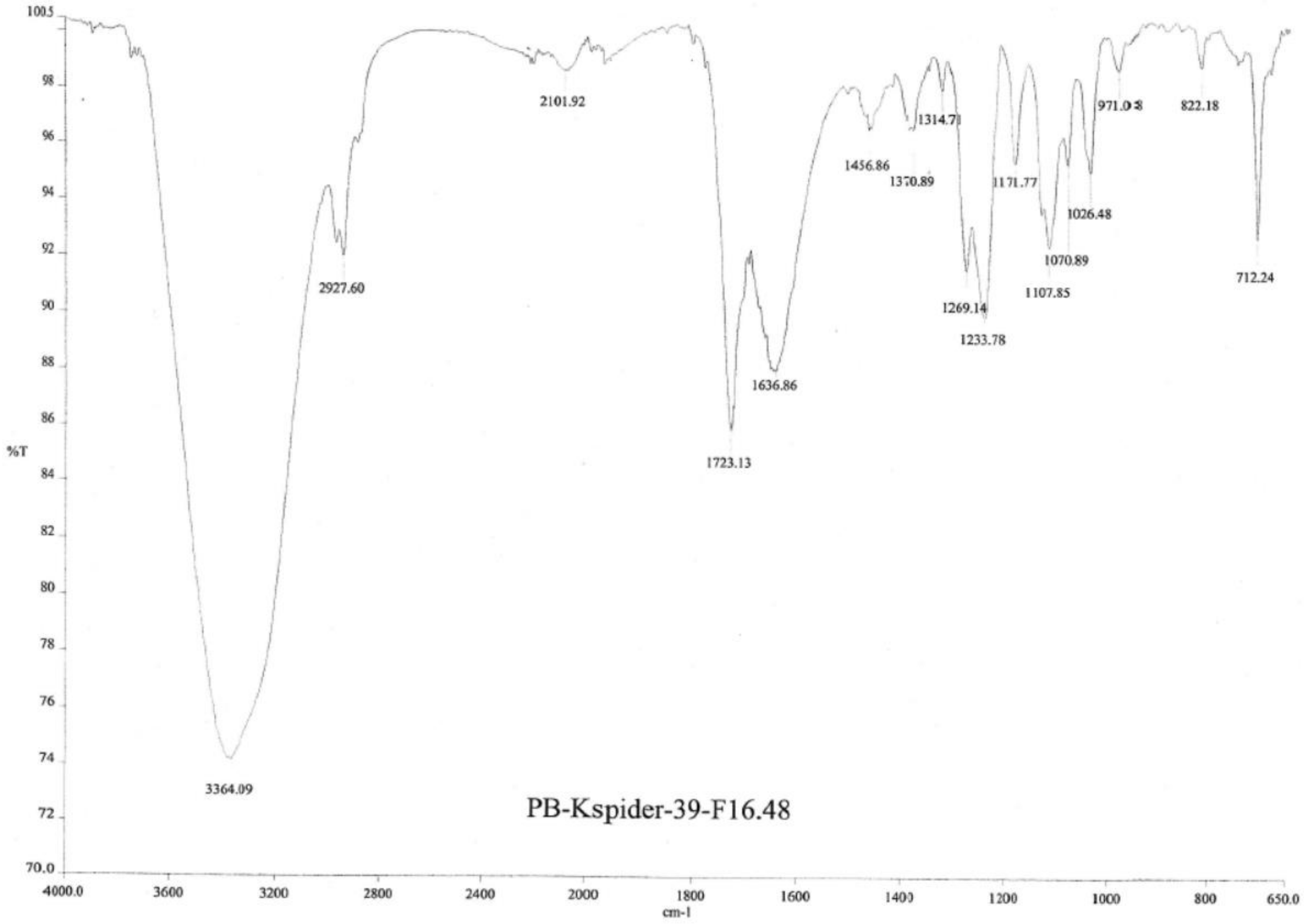

Figure S62. IR spectrum of compound **6**

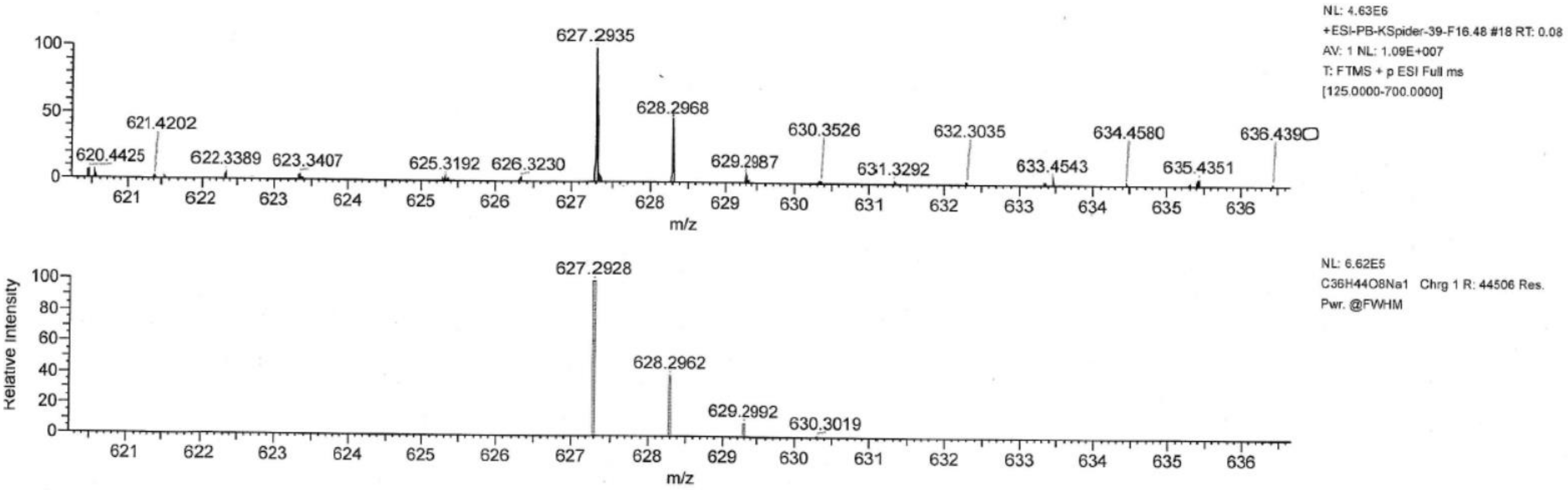

Peak Mass	Display Formula	Delta [ppm]	Theo. mass	MSMS Matched Fragments
627.2935	$C_{36}H_{44}O_8{}^{23}Na$ [M+Na]+	0.98	627.29284	(Collection)
627.2935	$C_{38}H_{43}O_8$	-2.85	627.29524	(Collection)
627.2935	$C_{11}H_{56}O_{26}{}^{23}Na$	-2.78	627.29520	(Collection)
627.2935	$C_{20}H_{51}O_{21}$	2.75	627.29173	(Collection)

Figure S63. HRESIMS spectrum of compound **6**

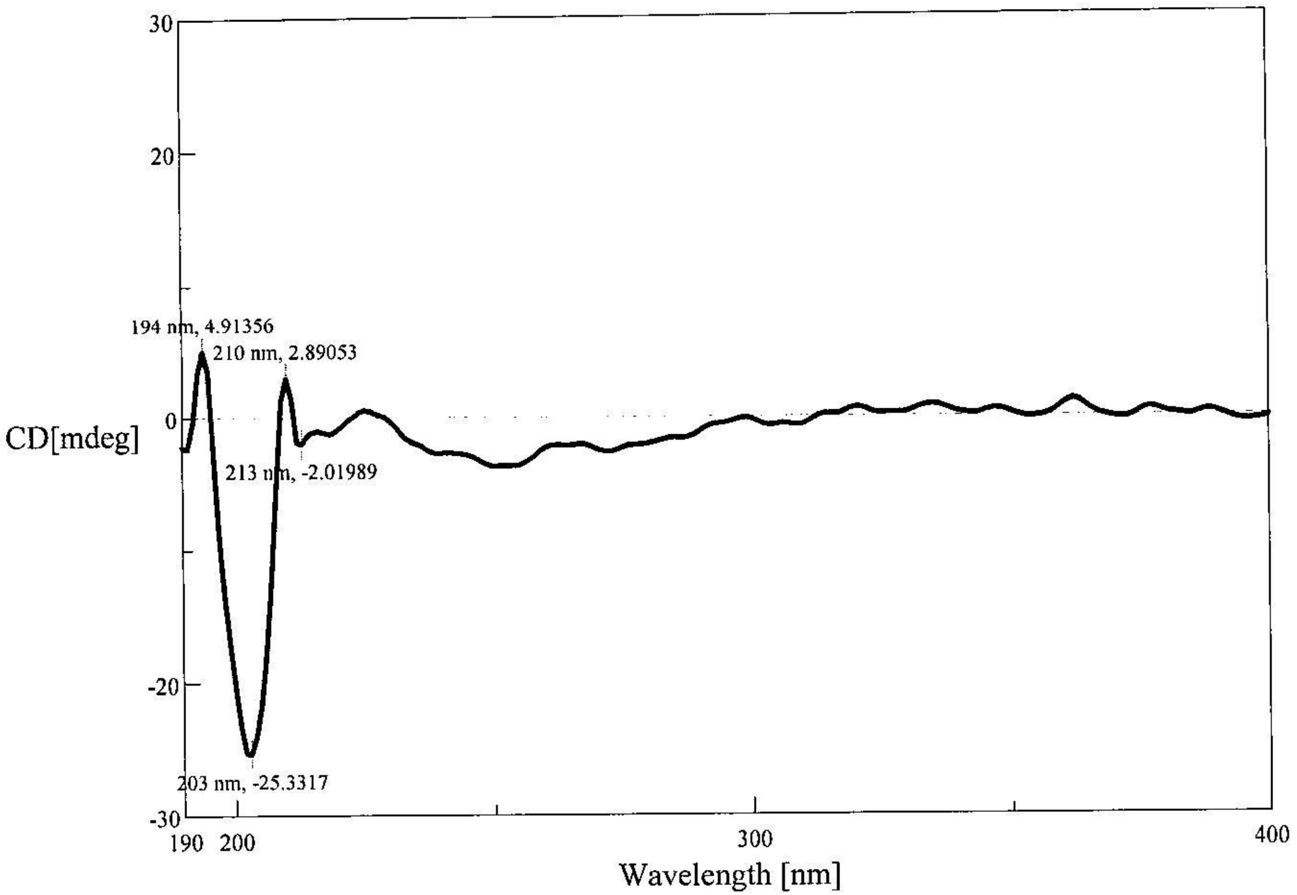

Figure S64. CD spectrum of compound **6**

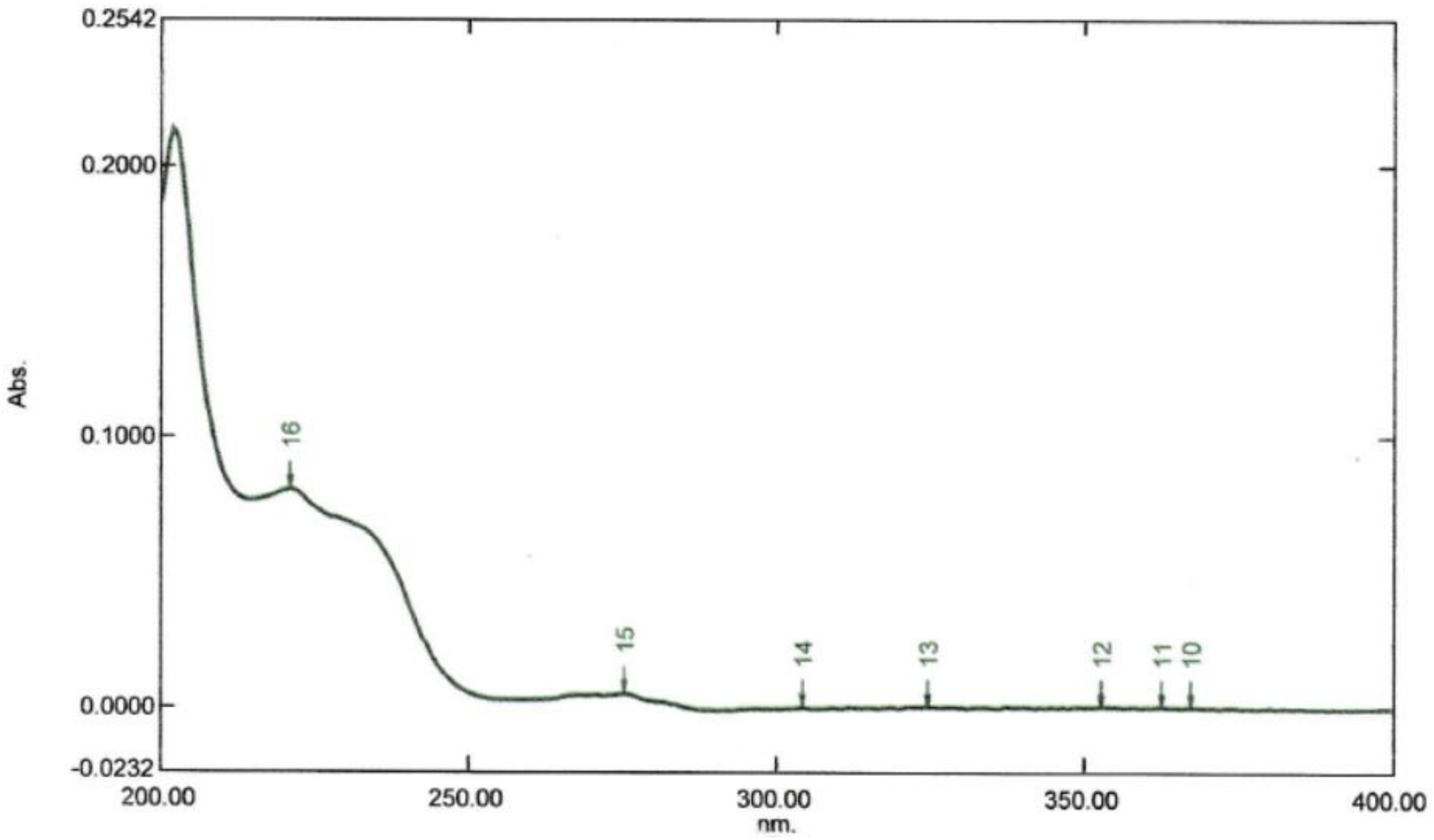

Figure S65. UV spectrum of compound **6**

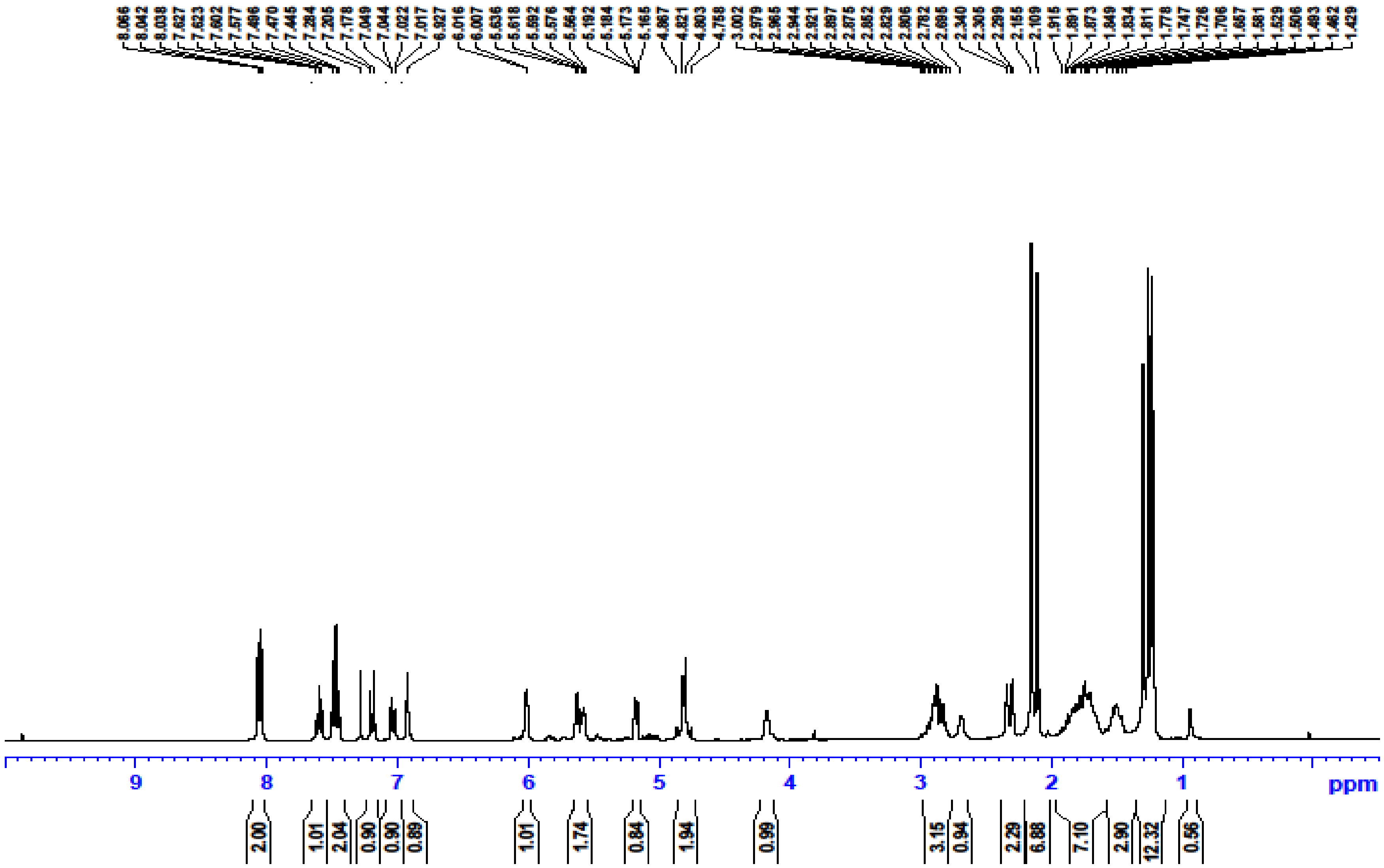

Figure S66. ^{1}H NMR (300 MHz) spectrum of compound **7** in $CDCl_3$

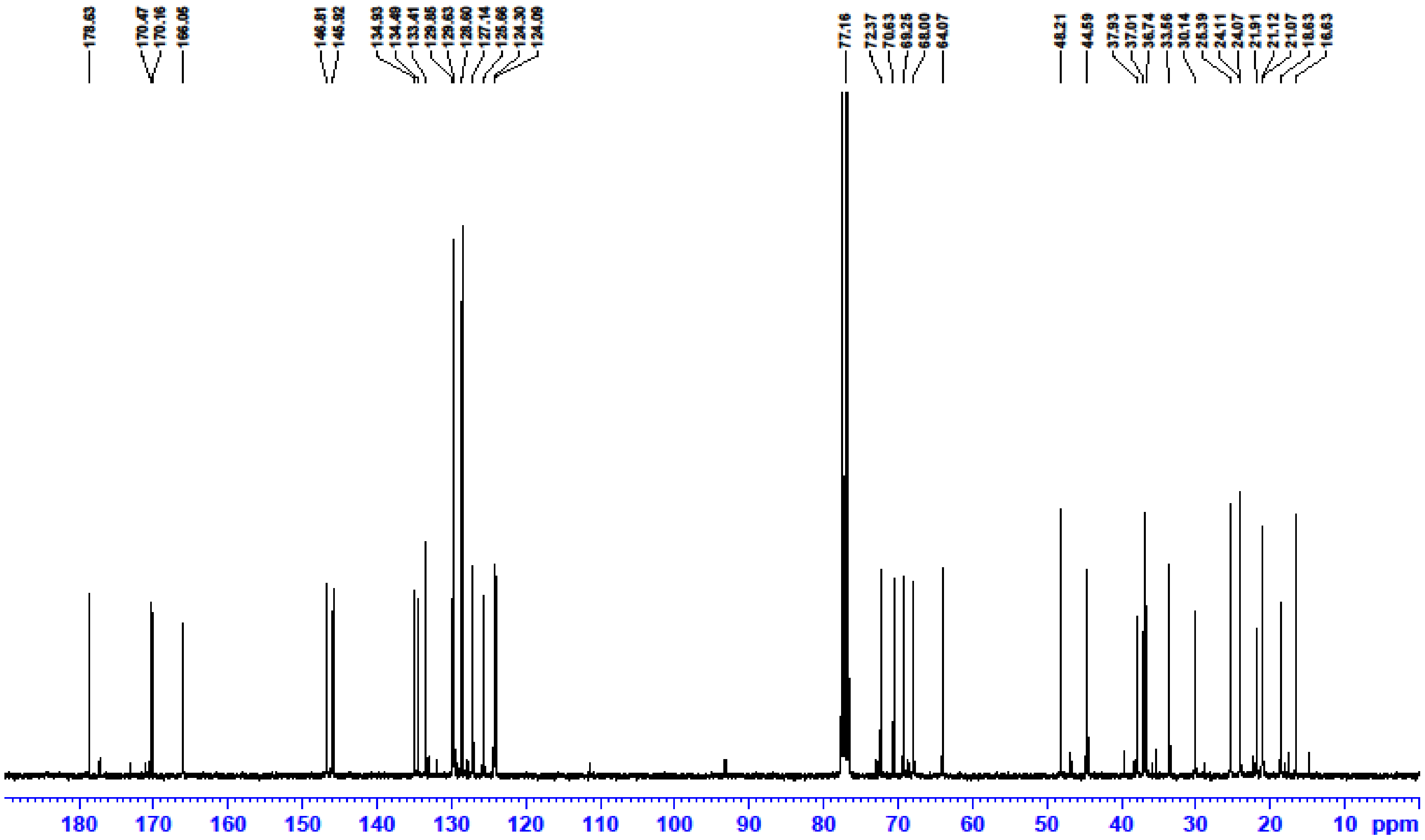

Figure S67. ^{13}C NMR (75 MHz) spectrum of compound **7** in $CDCl_3$

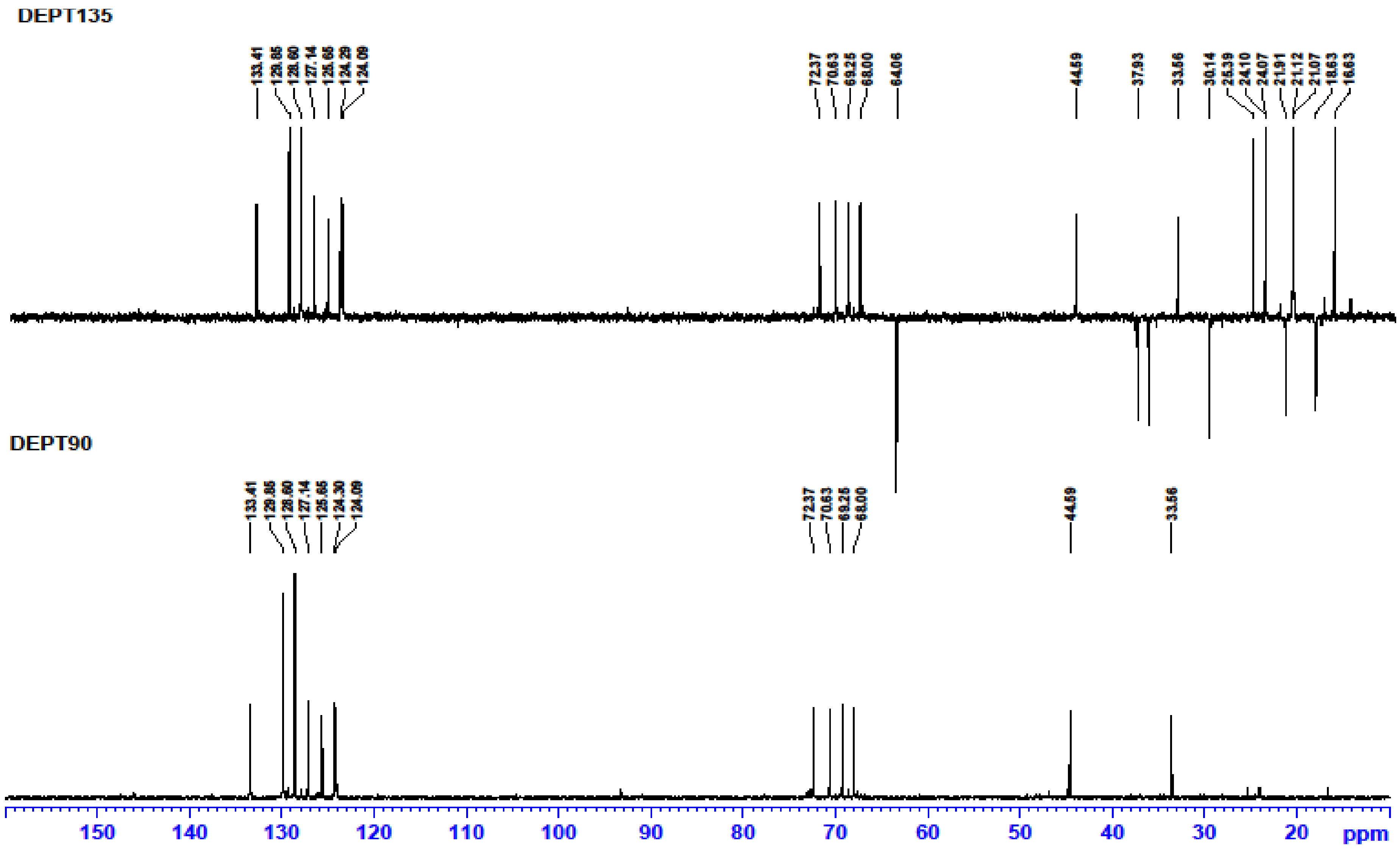

Figure S68. DEPT 135 & 90 NMR spectrum of compound **7** in $CDCl_3$

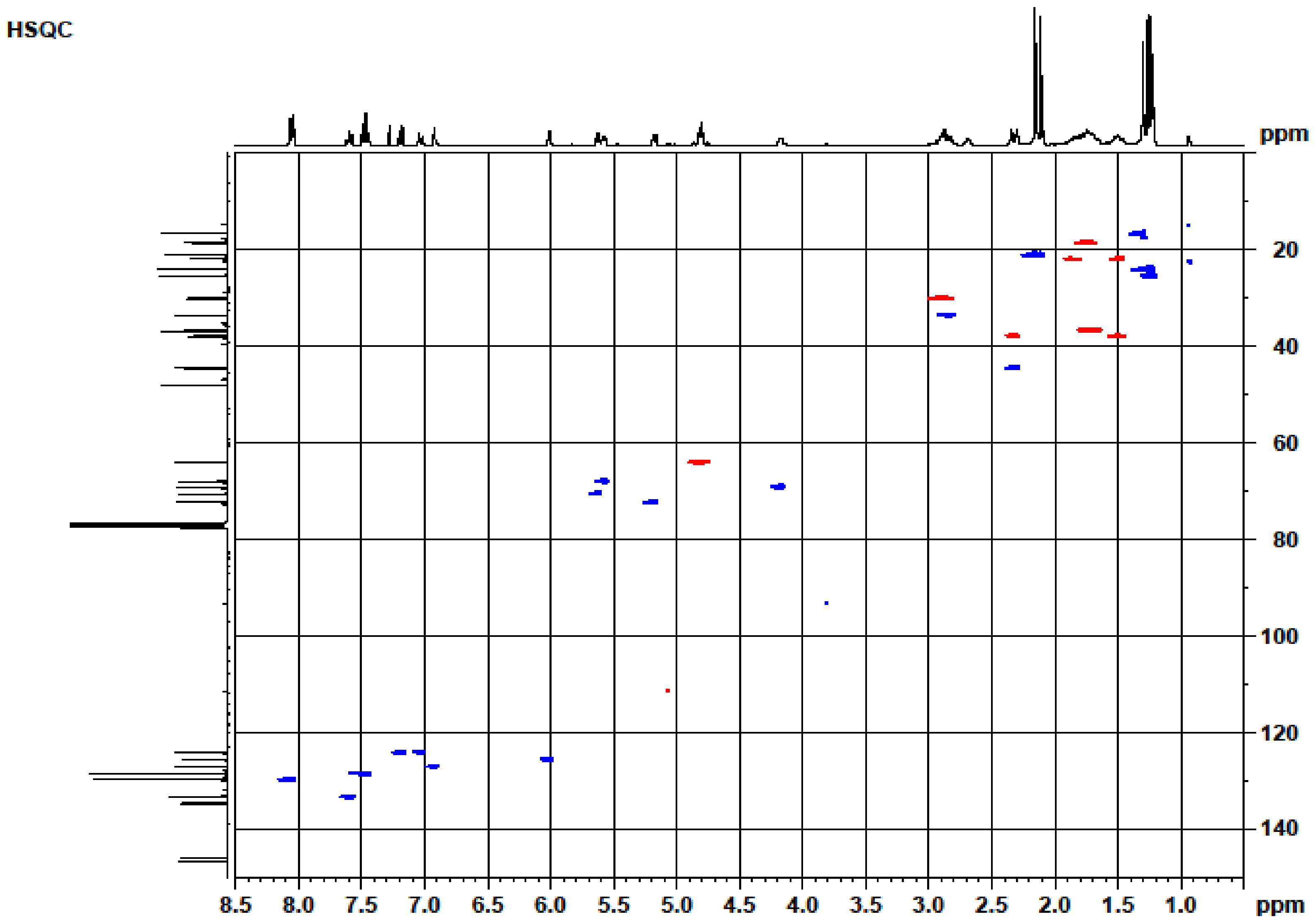

Figure S69. HSQC spectrum of compound **7** in $CDCl_3$

COSY

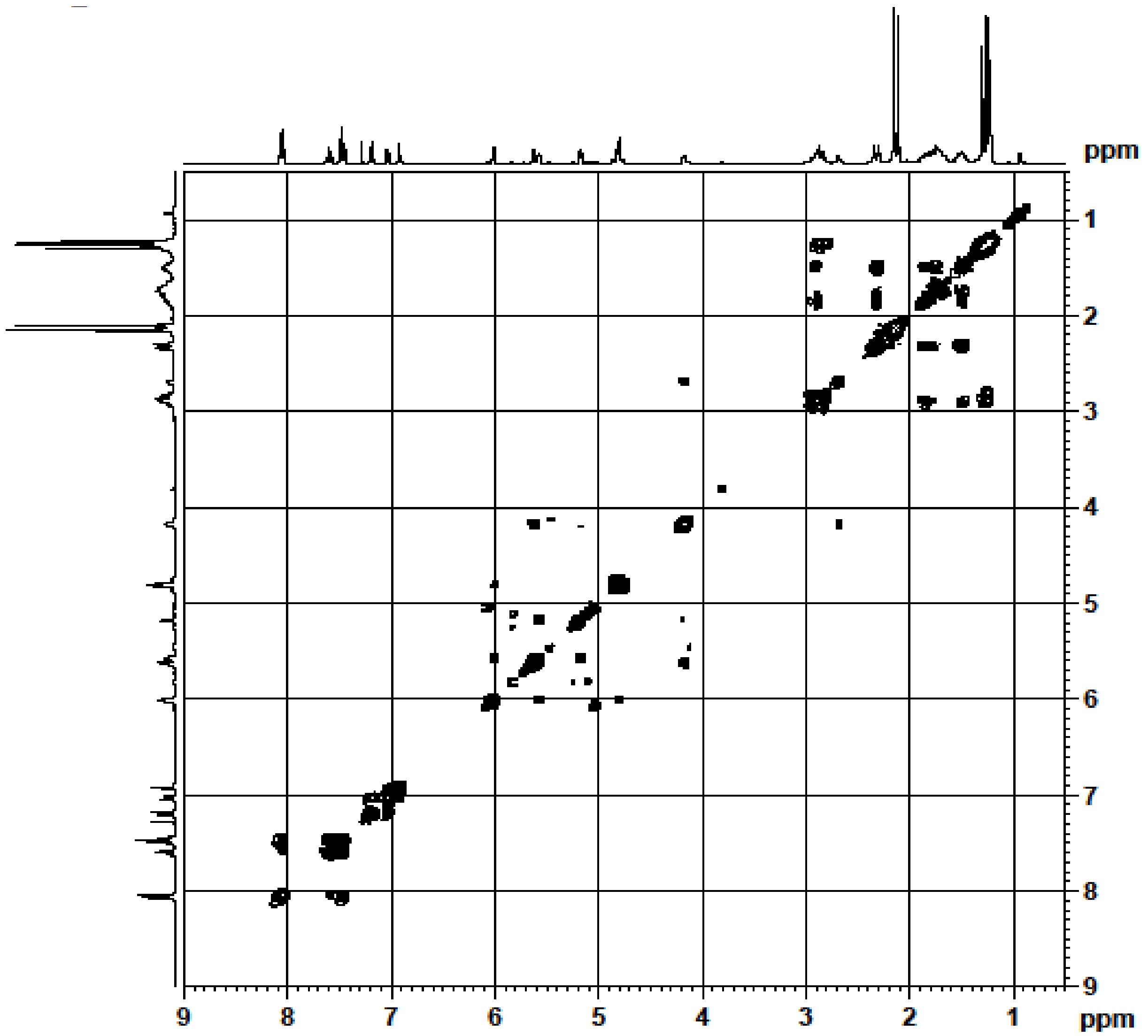

Figure S70. COSY spectrum of compound **7** in $CDCl_3$

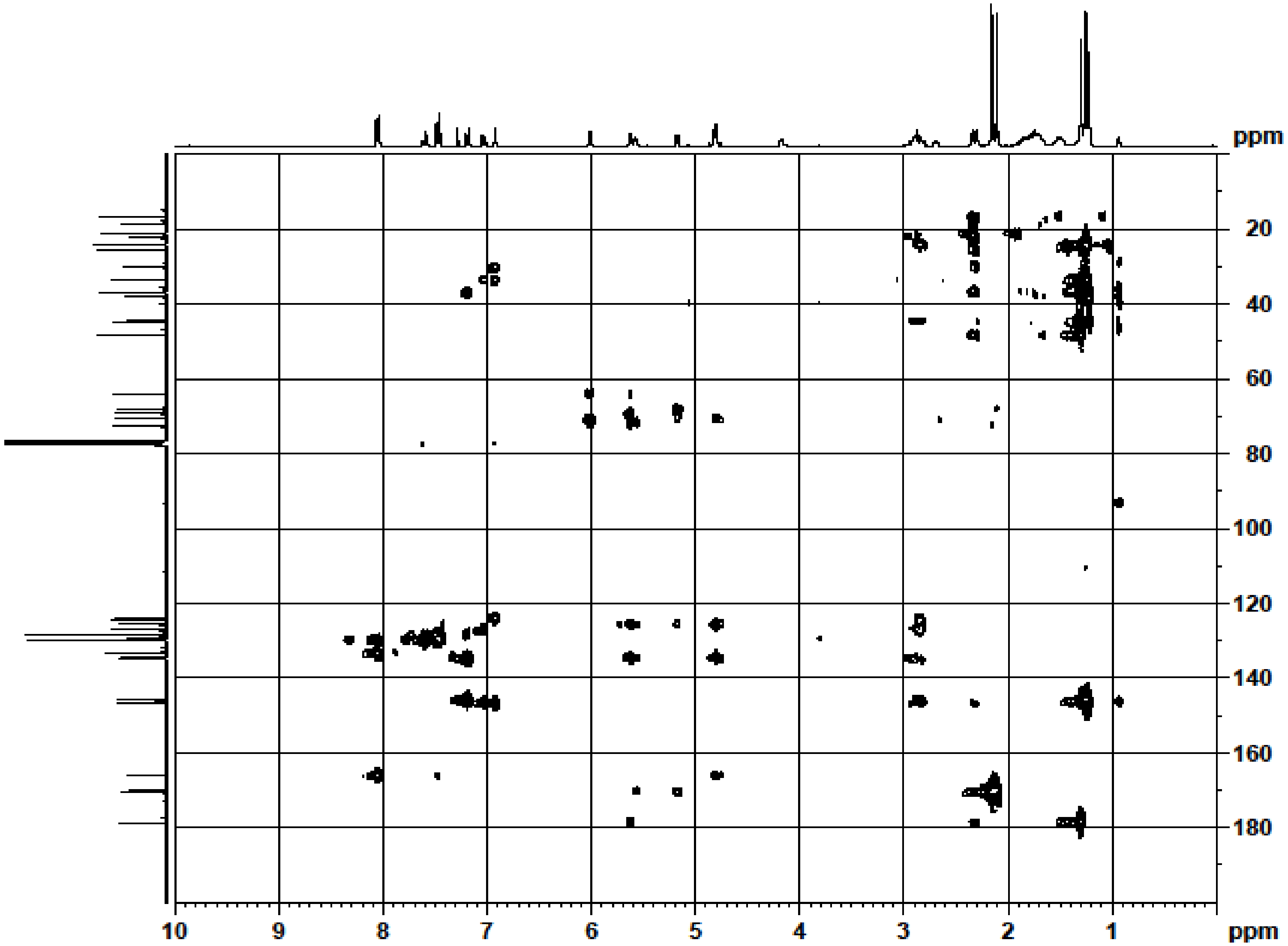

Figure S71. HMBC spectrum of compound **7** in $CDCl_3$

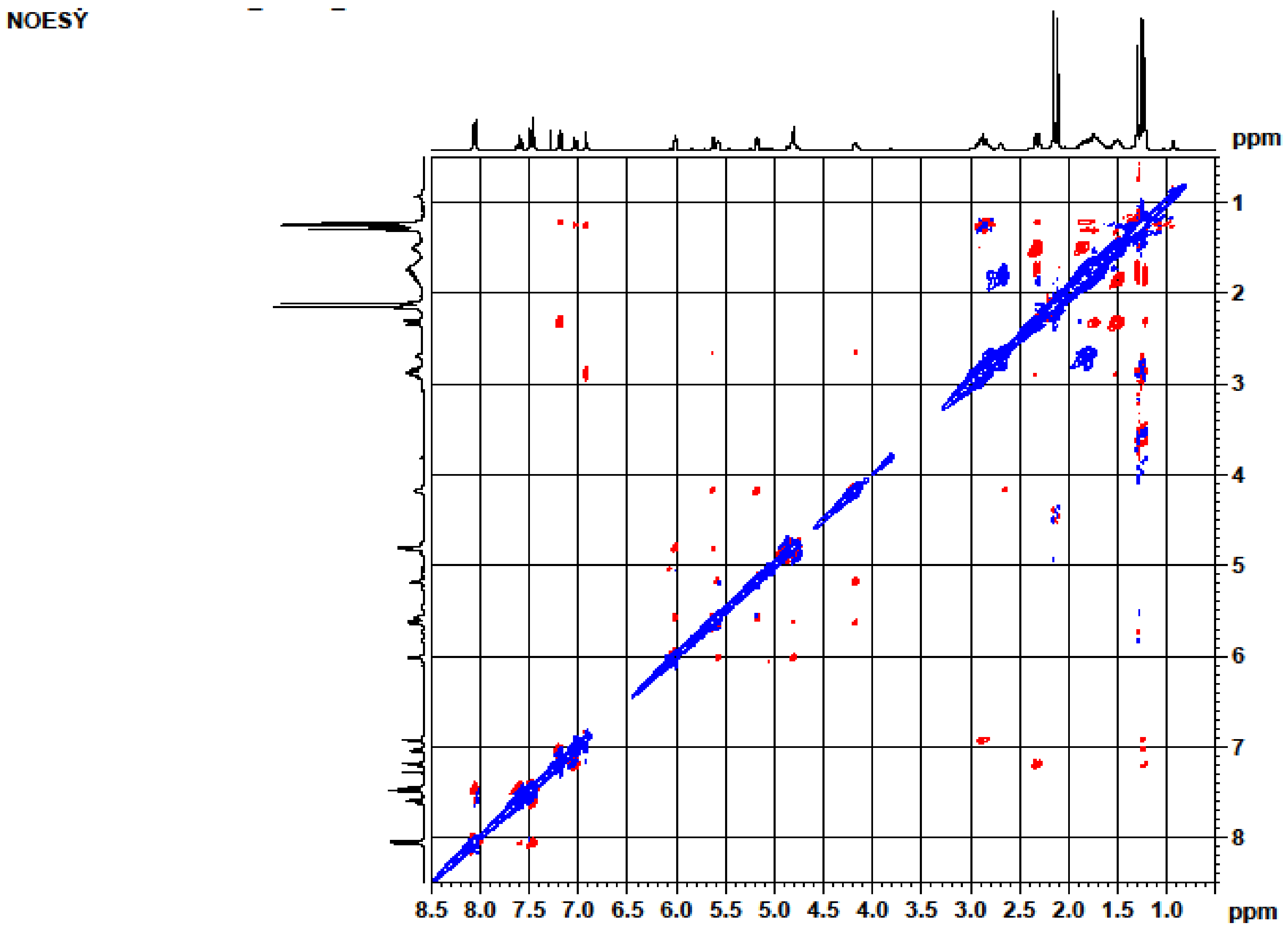

Figure S72. NOESY spectrum of **7** in $CDCl_3$

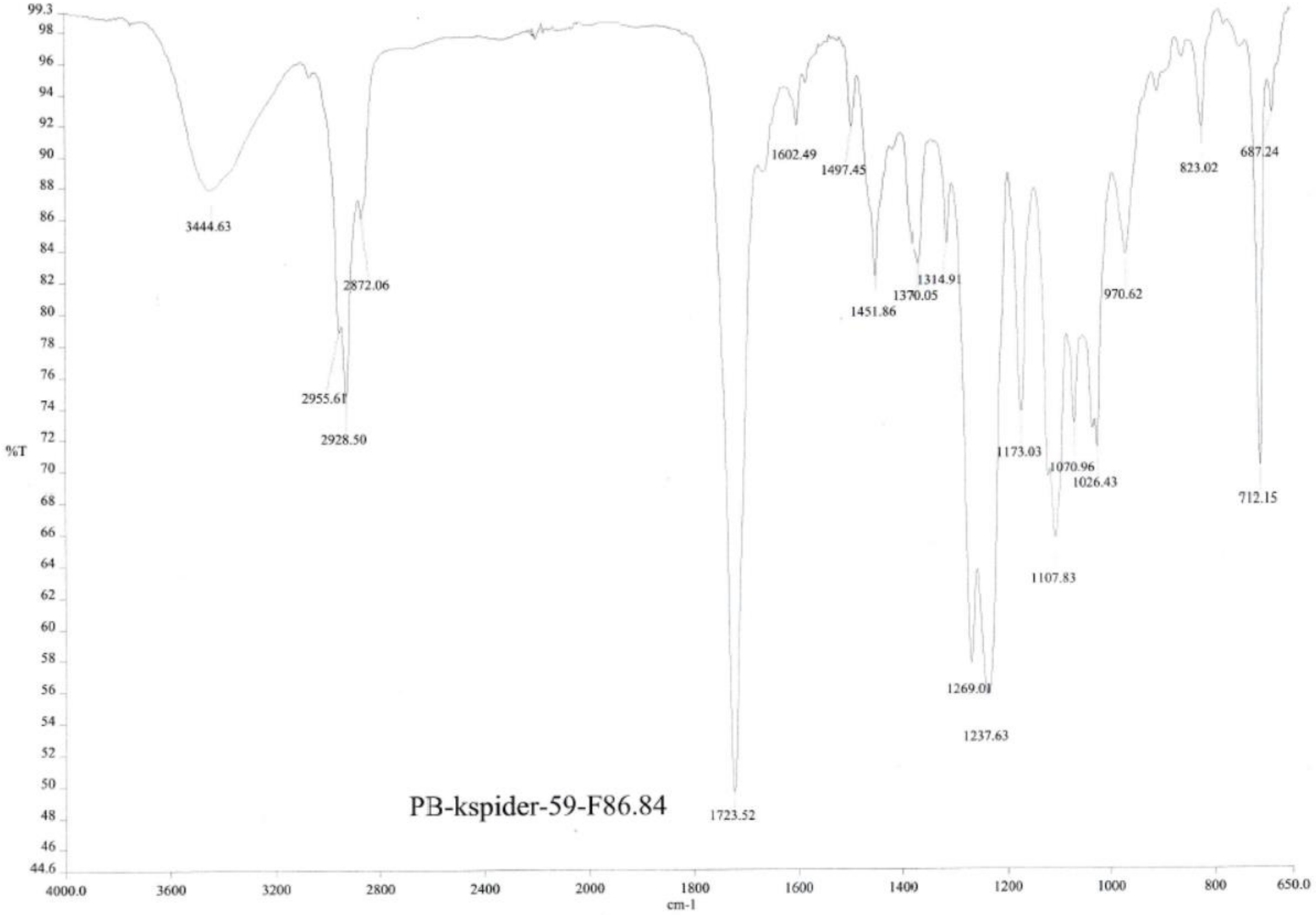

Figure S73. IR spectrum of compound **7**

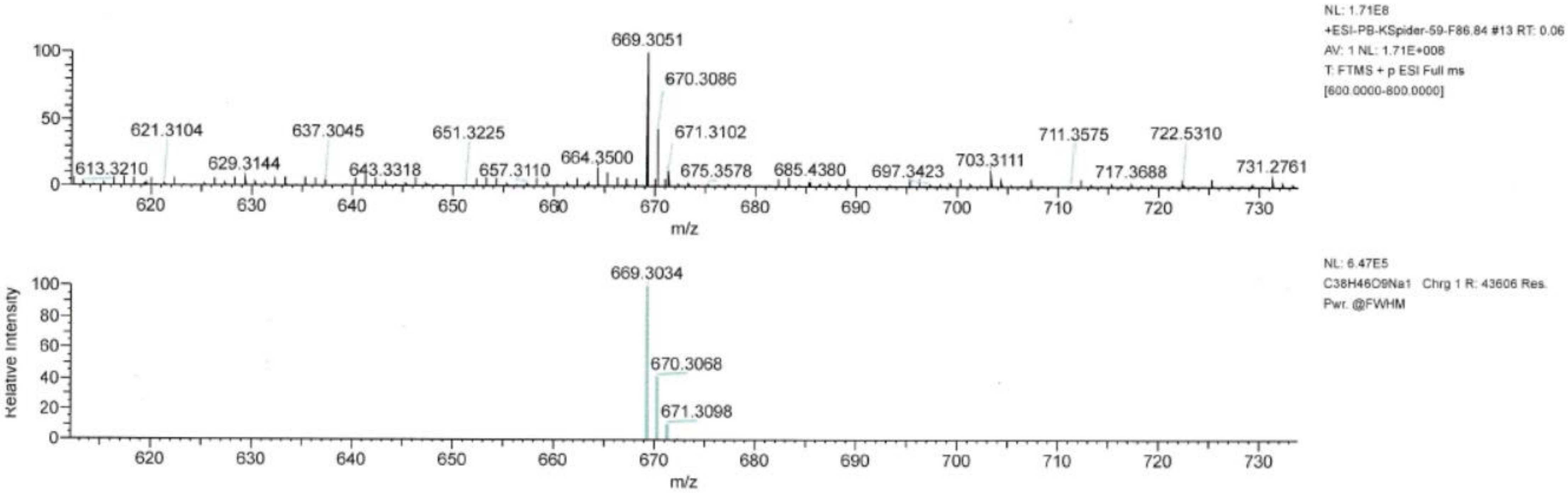

Peak Mass	Display Formula	Delta [ppm]	Theo. mass	MSMS Matched Fragments
669.3051	$C_{38}H_{46}O_9{}^{23}Na$ [M+Na]+	2.46	669.30340	(Collection)
669.3051	$C_{40}H_{45}O_9$	-1.13	669.30581	(Collection)
669.3051	$C_{13}H_{58}O_{27}{}^{23}Na$	-1.07	669.30577	(Collection)
669.3051	$C_{22}H_{53}O_{22}$	4.12	669.30230	(Collection)
669.3051	$C_{15}H_{57}O_{27}$	-4.66	669.30817	(Collection)

Figure S74. HRESIMS spectrum of compound **7**

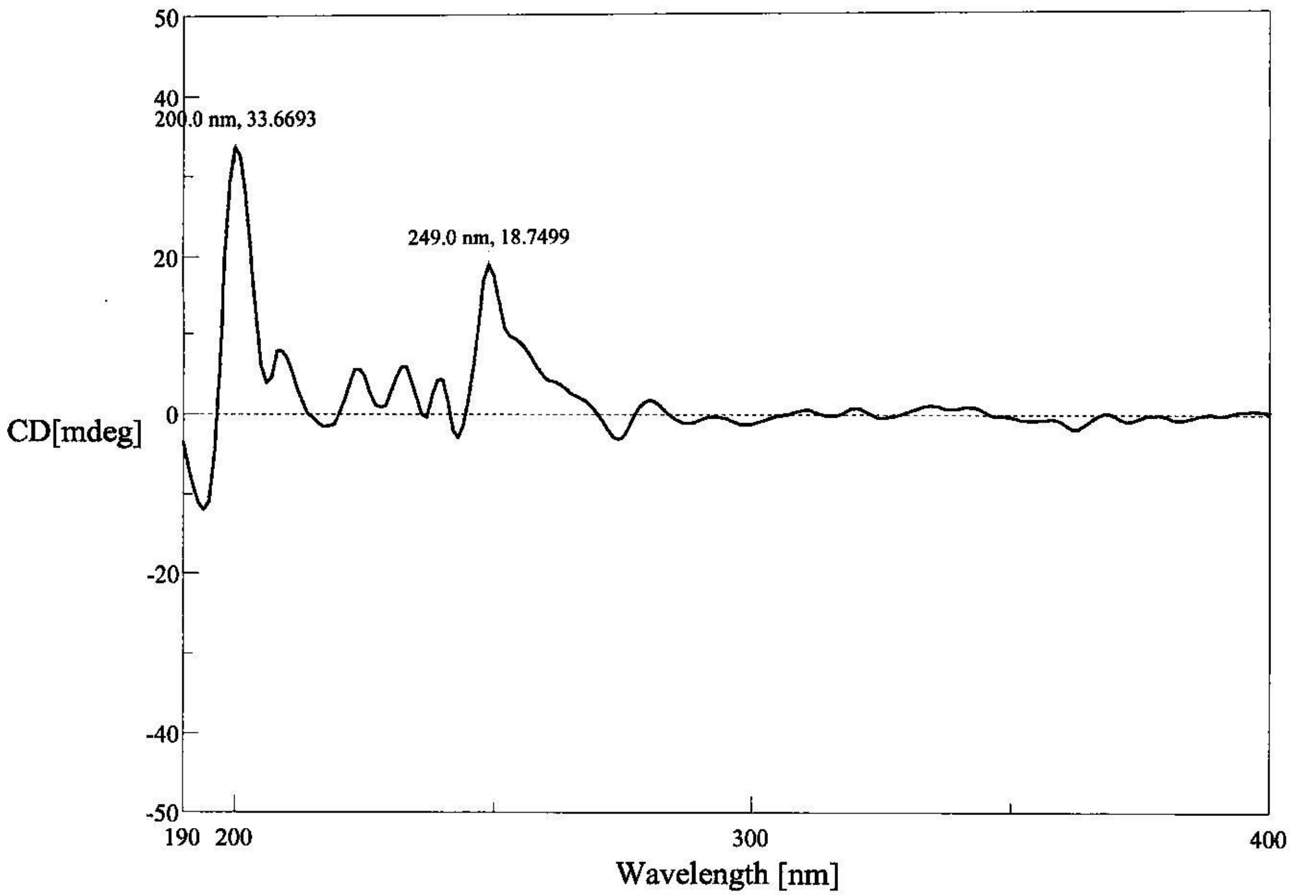

Figure S75. CD spectrum of compound **7**

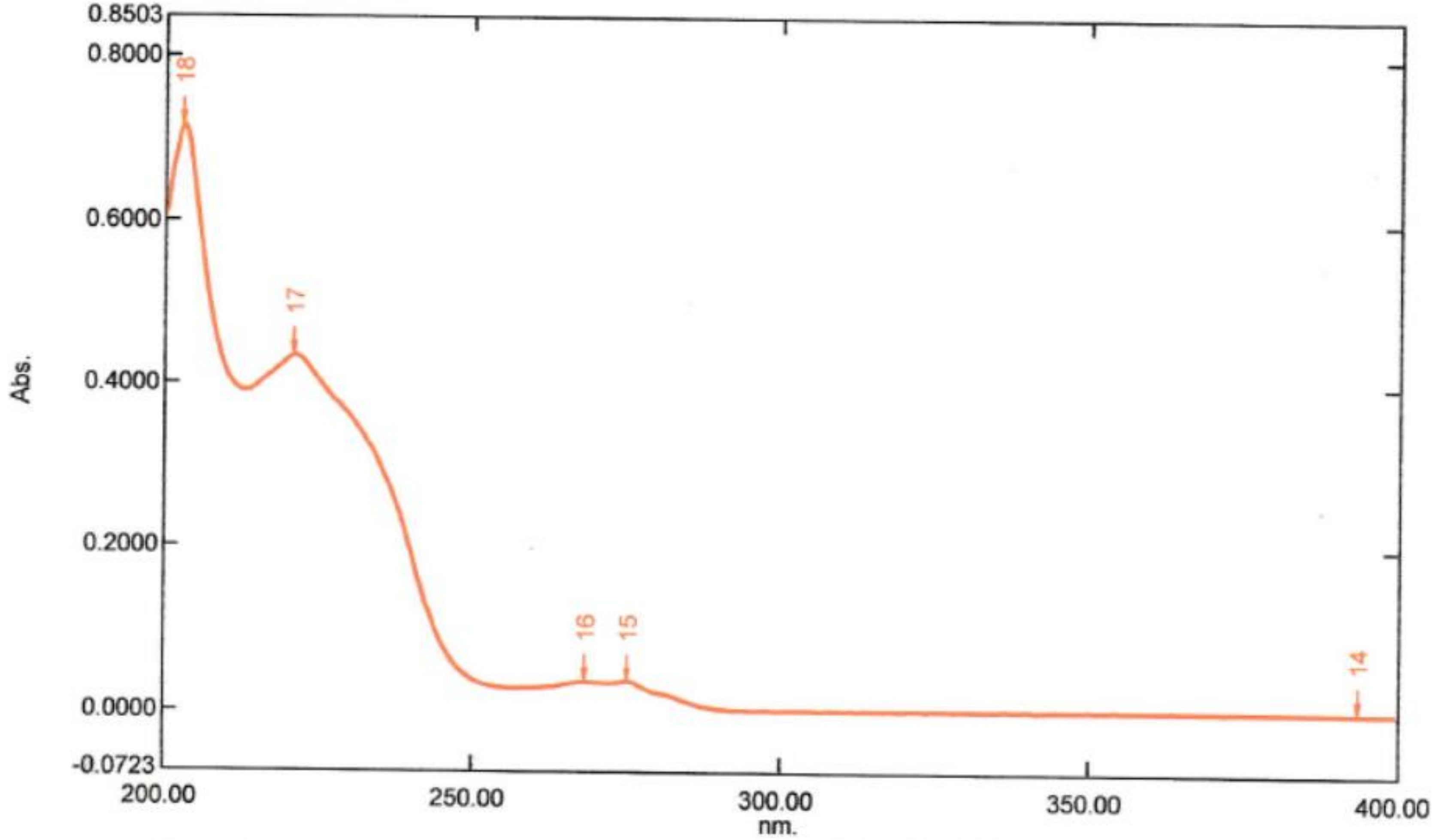

Figure S76. UV spectrum of compound **7**

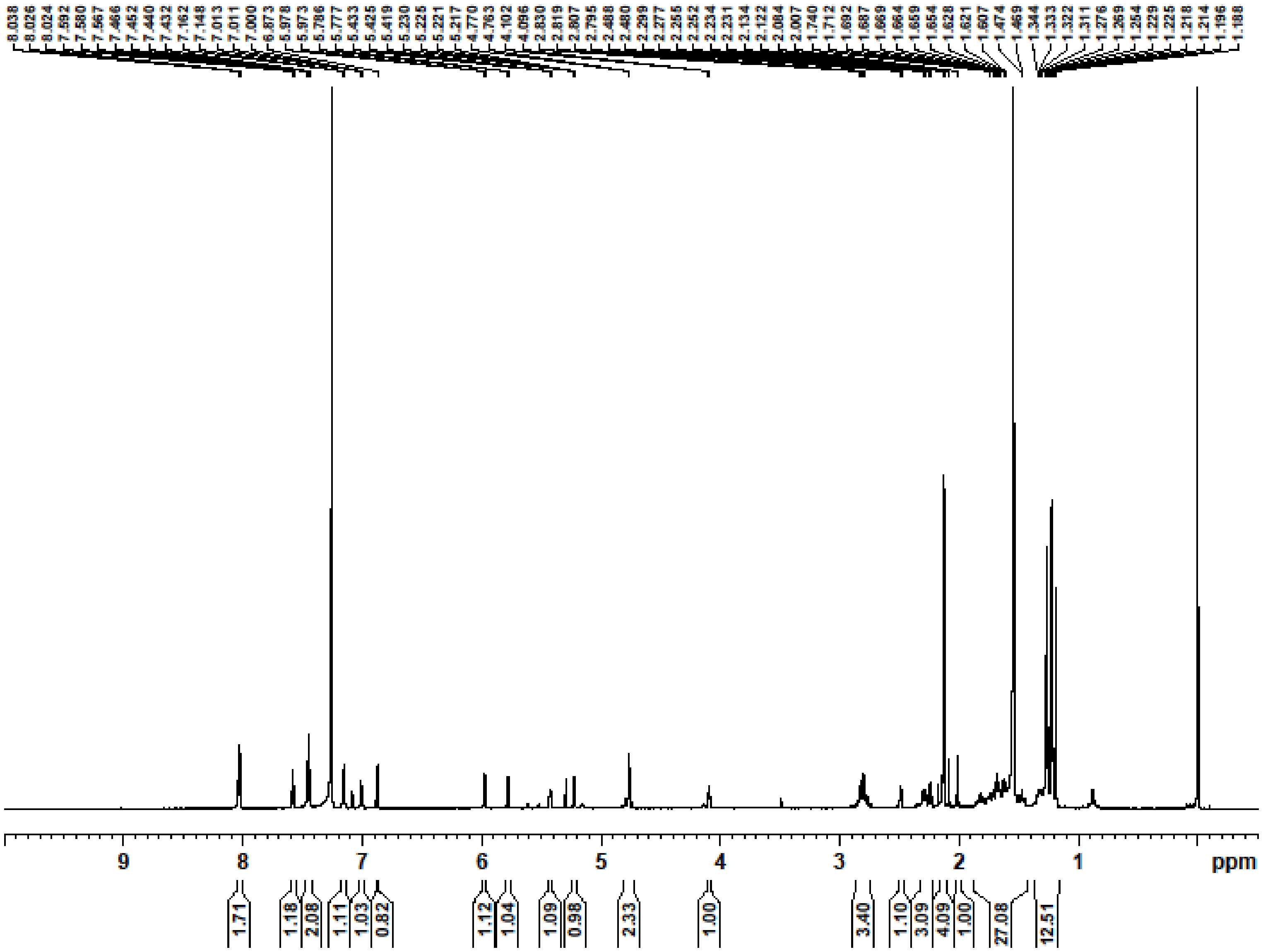

Figure S77. ^{1}H NMR (600 MHz) spectrum of compound **8** in $CDCl_3$

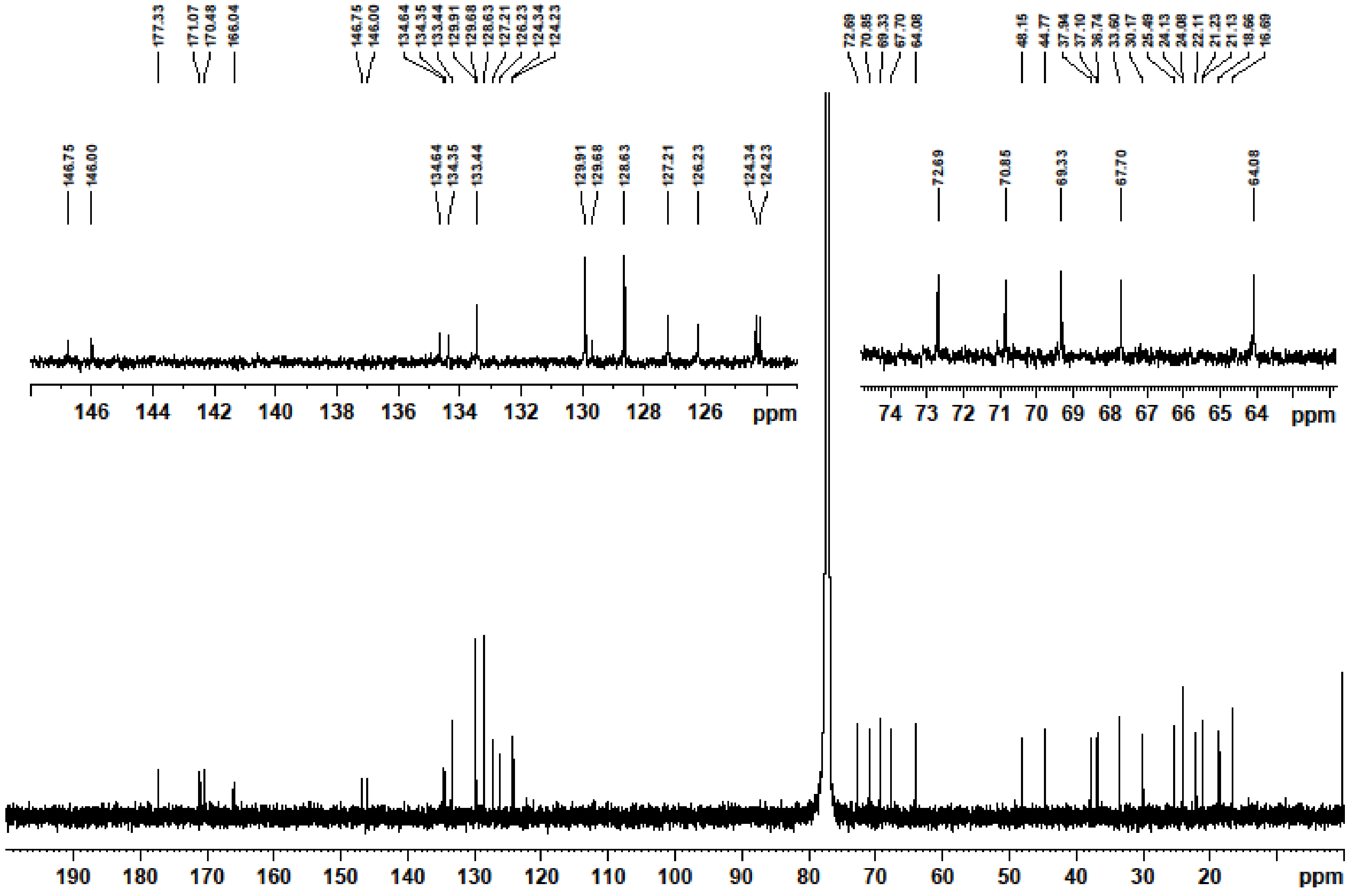

Figure S78. ^{13}C NMR (150 MHz) spectrum of compound **8** in $CDCl_3$

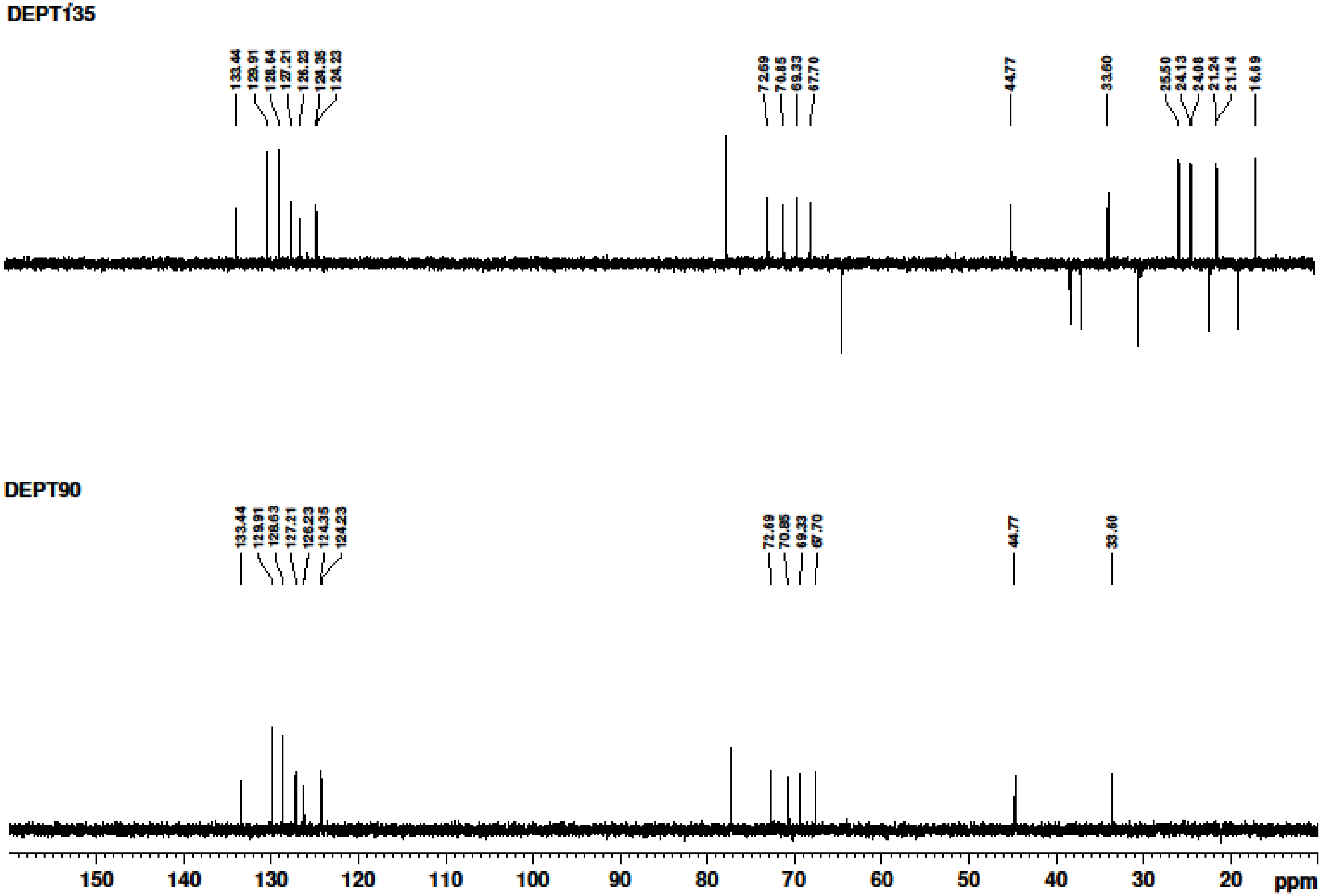

Figure S79. DEPT 135 & 90 NMR spectrum of compound **8** in $CDCl_3$

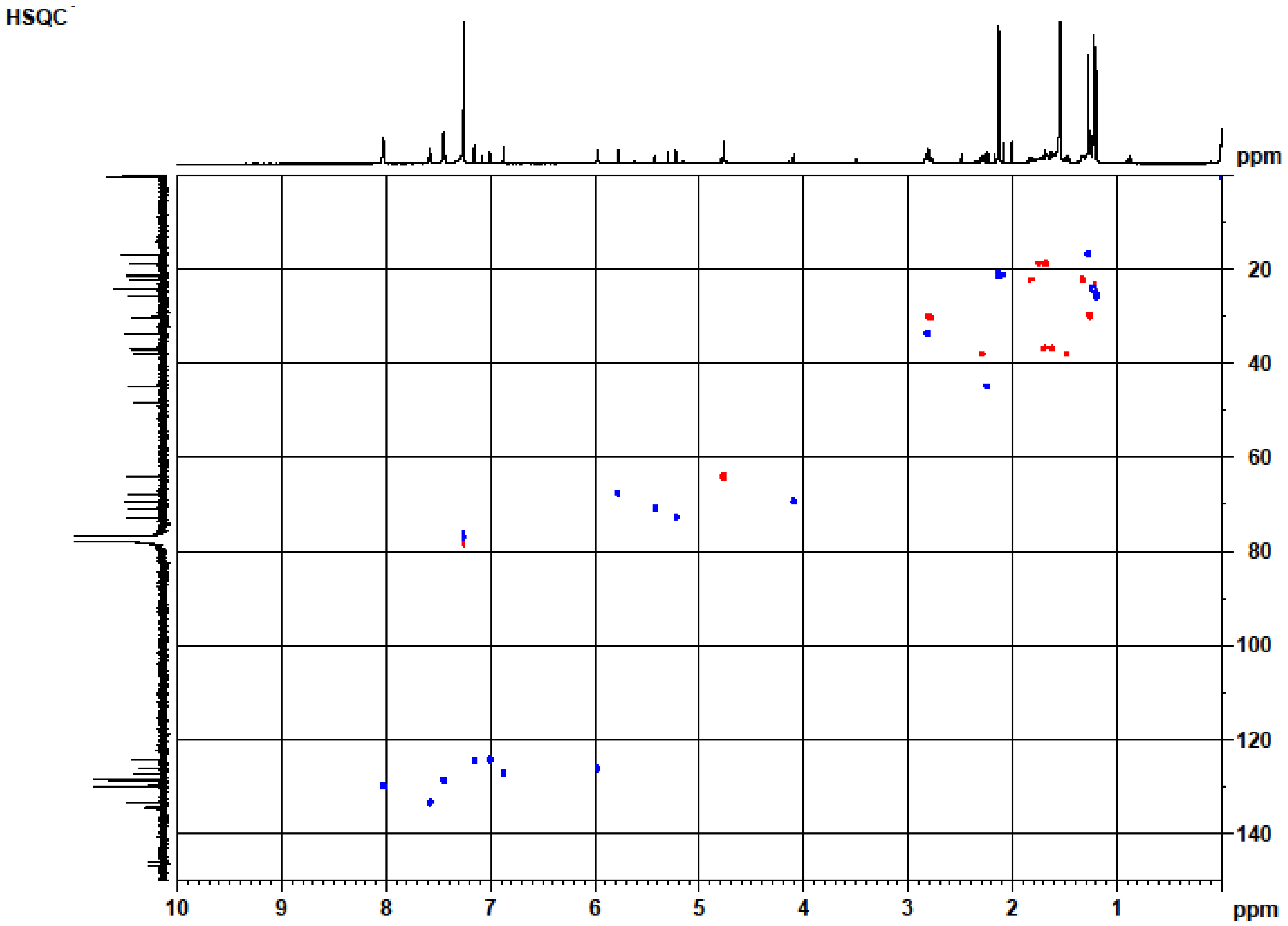

Figure S80. HSQC spectrum of compound **8** in $CDCl_3$

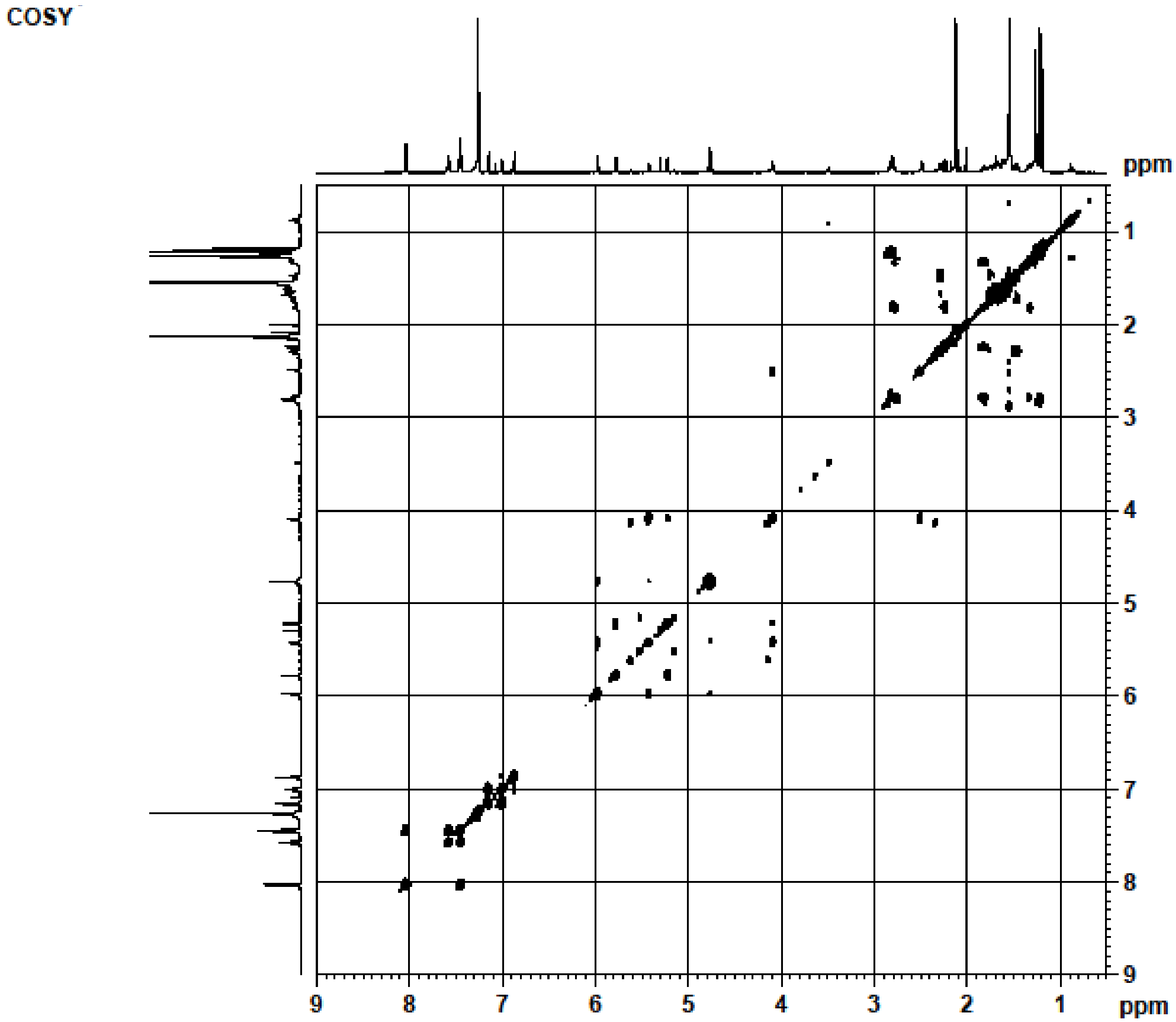

Figure S81. COSY spectrum of compound **8** in $CDCl_3$

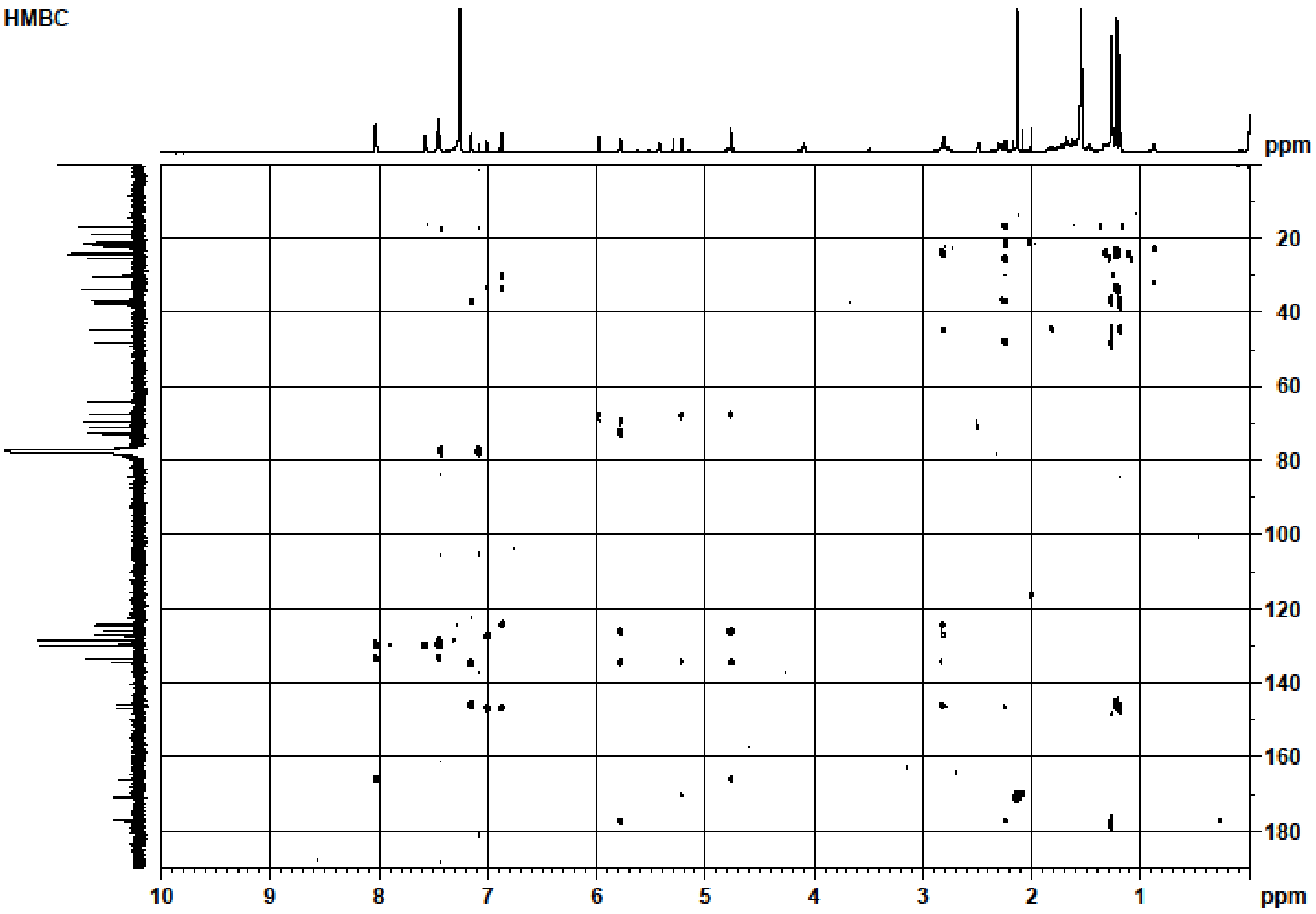

Figure S82. HMBC spectrum of compound **8** in $CDCl_3$

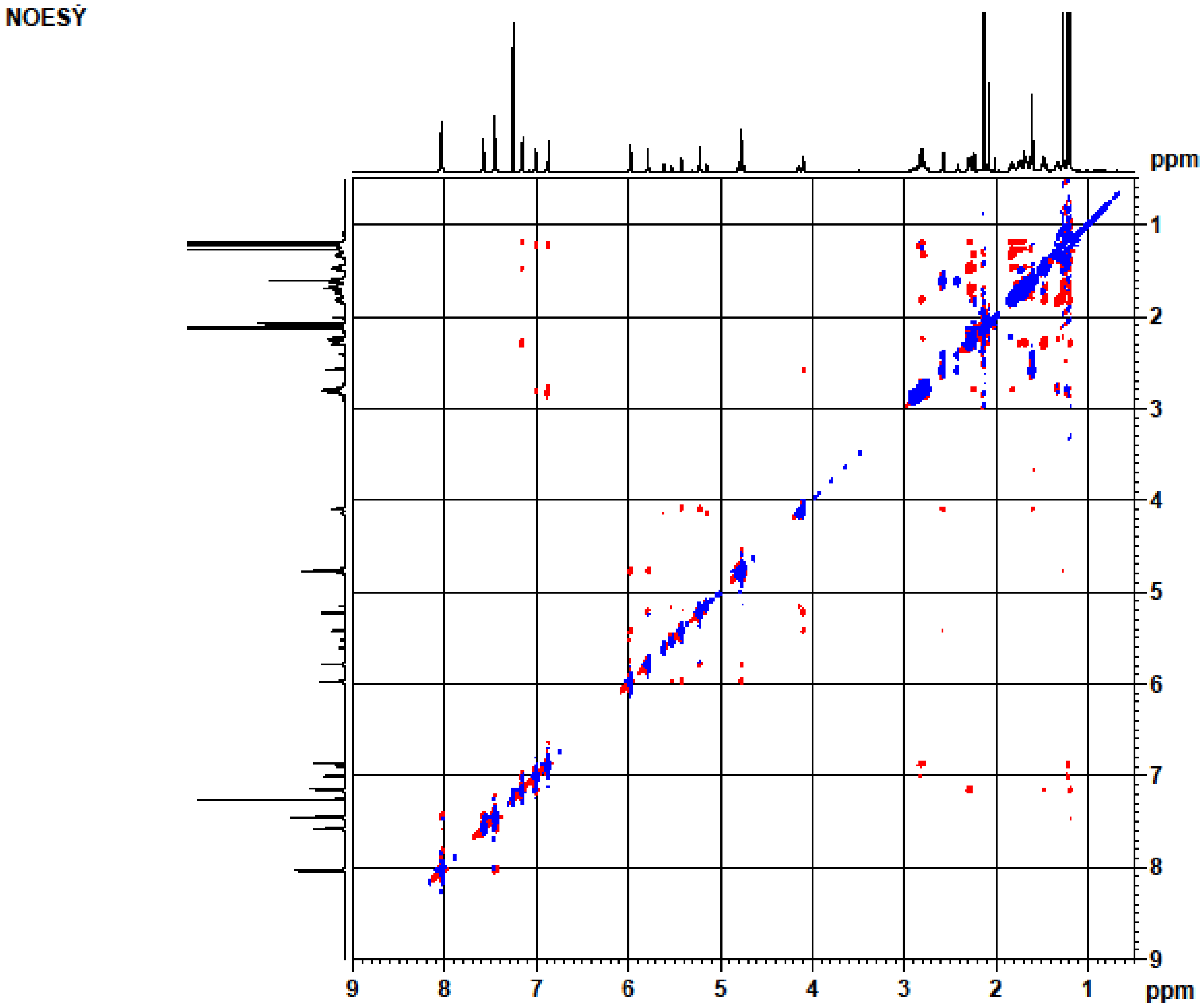

Figure S83. NOESY spectrum of **8** in $CDCl_3$

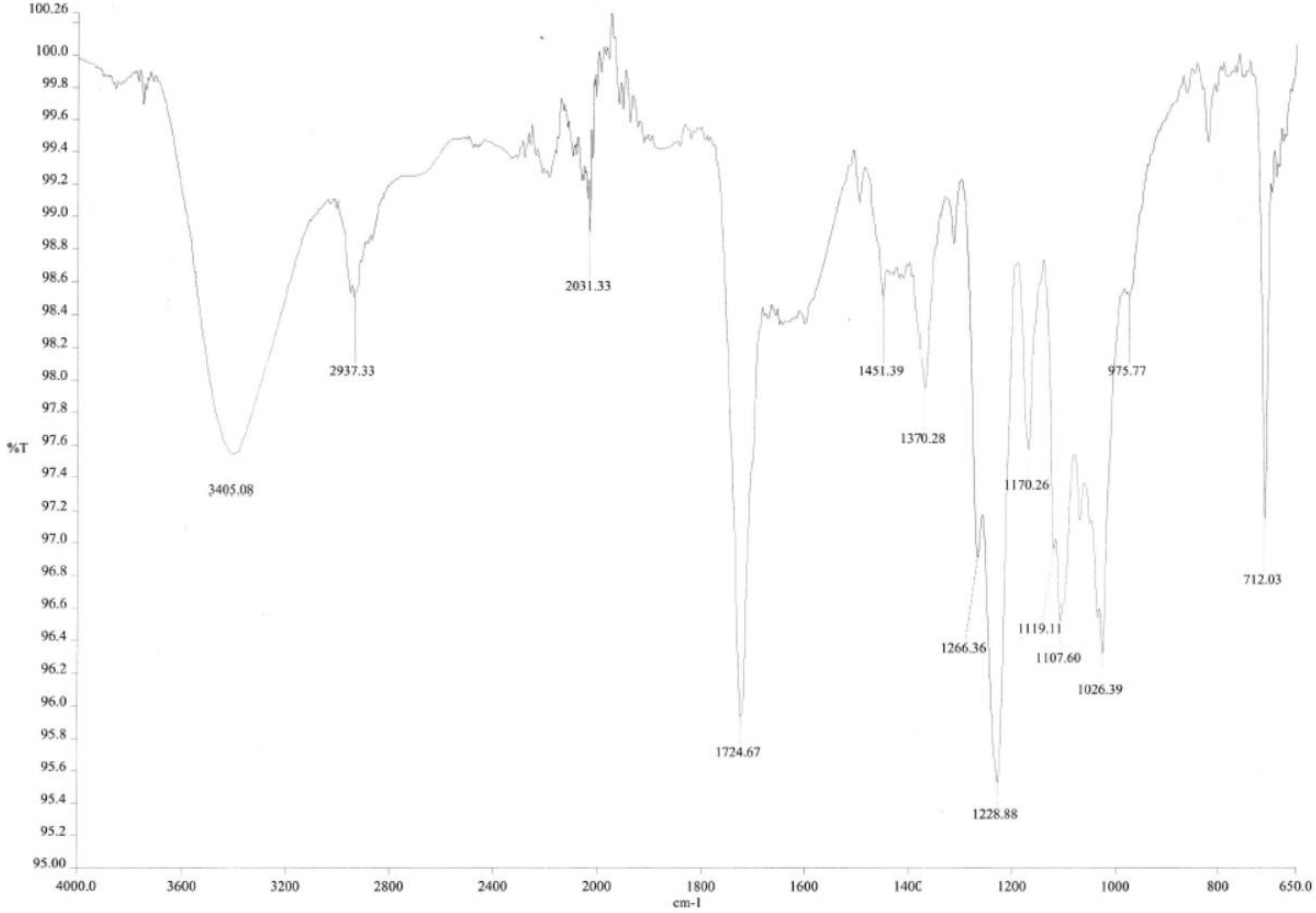

Figure S84. IR spectrum of compound **8**

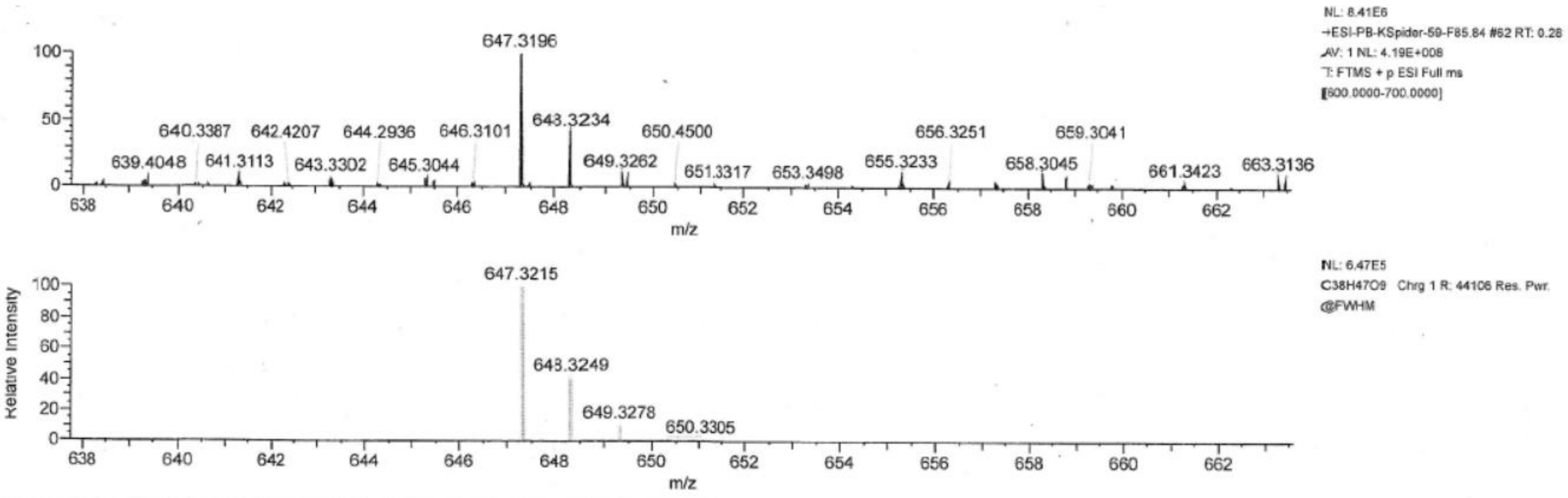

Peak Mass	Display Formula	Delta [ppm]	Theo. mass	MSMS Matched Fragments
647.3196	$C_{38}H_{47}O_{9}$ [M+H]+	-2.81	647.32146	(Collection)
647.3196	$C_{20}H_{55}O_{22}$	2.61	647.31795	(Collection)

Figure S85. HRESIMS spectrum of compound **8**

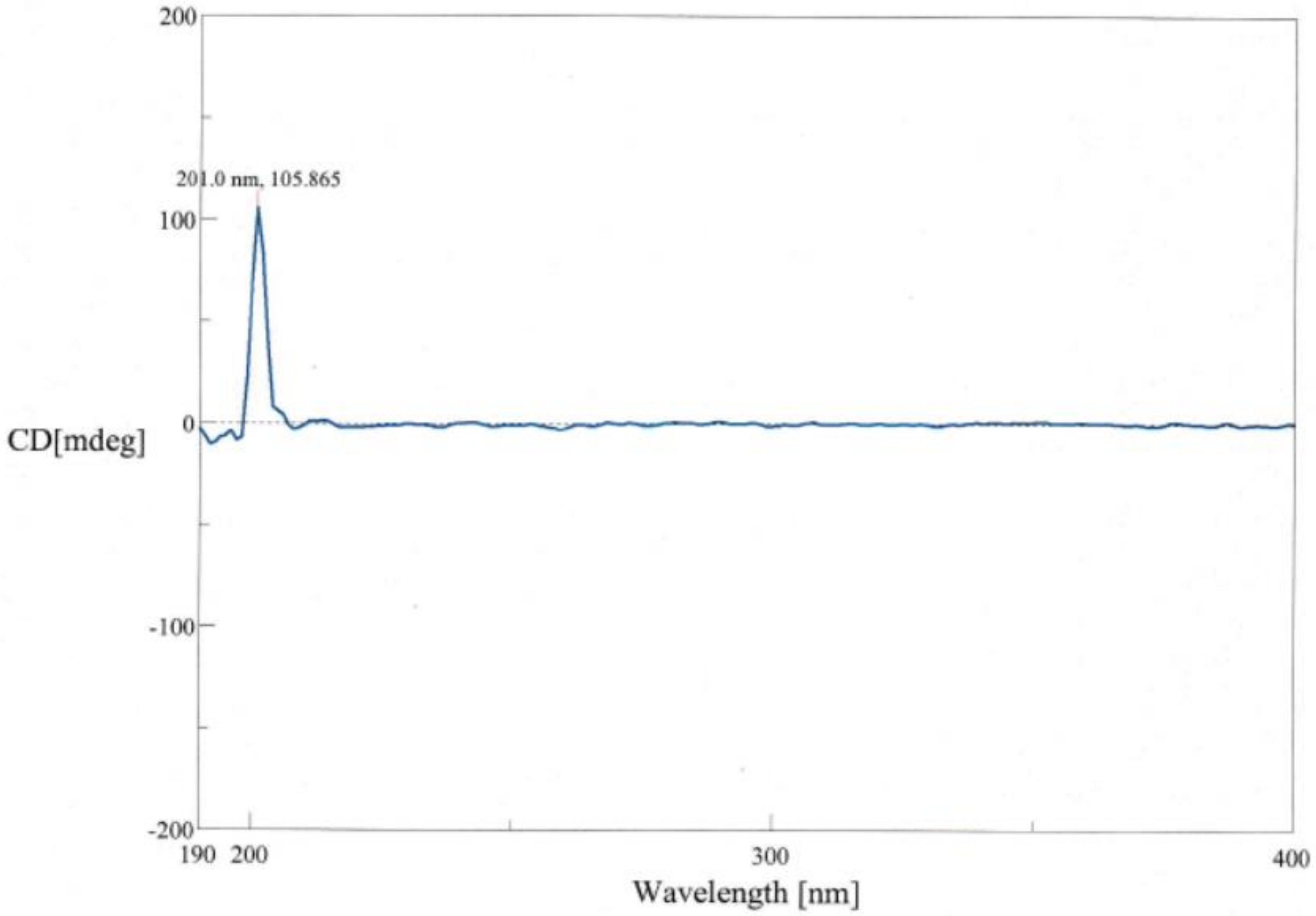

Figure S86. CD spectrum of compound **8**

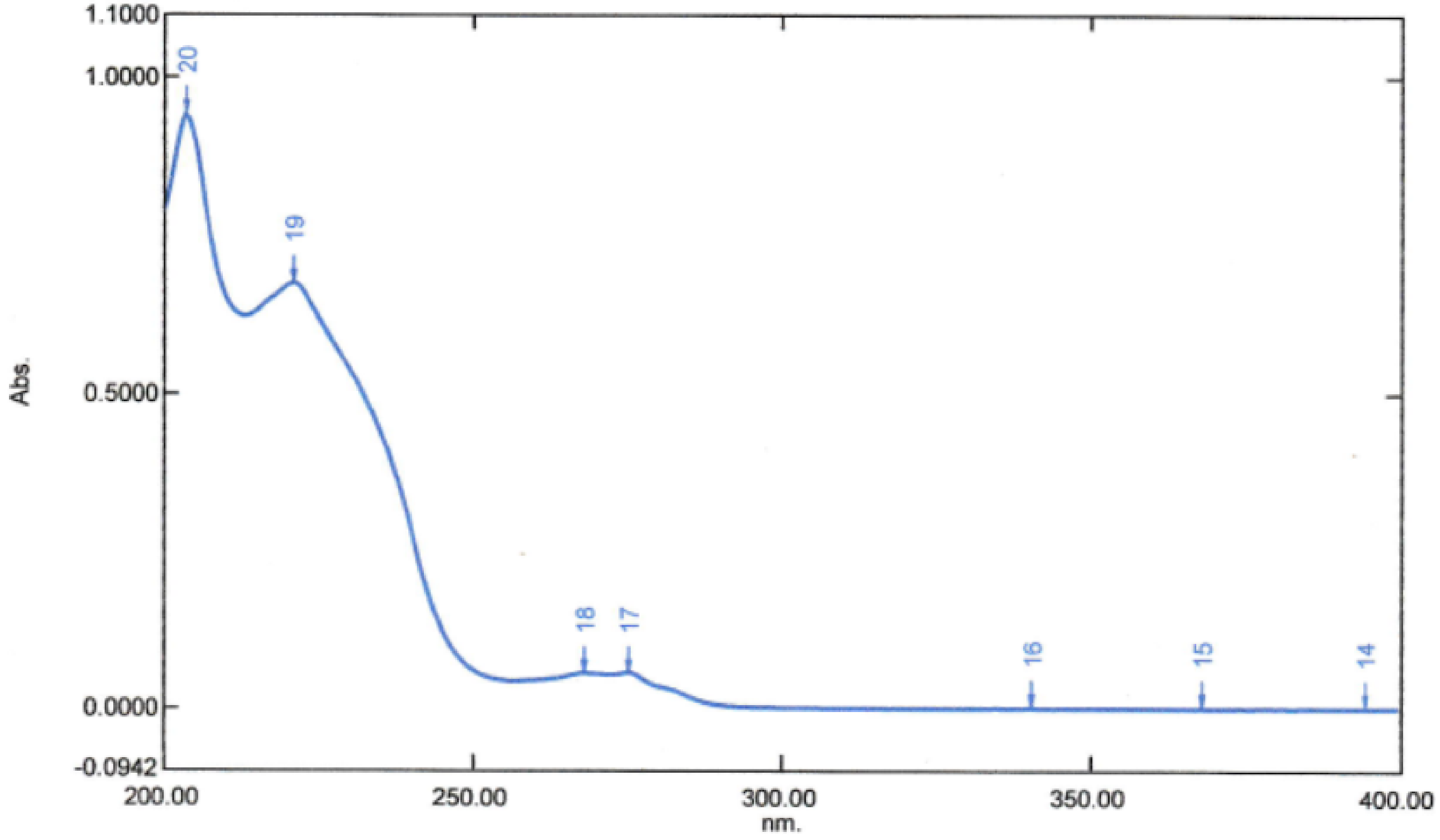

Figure S87. UV spectrum of compound **8**

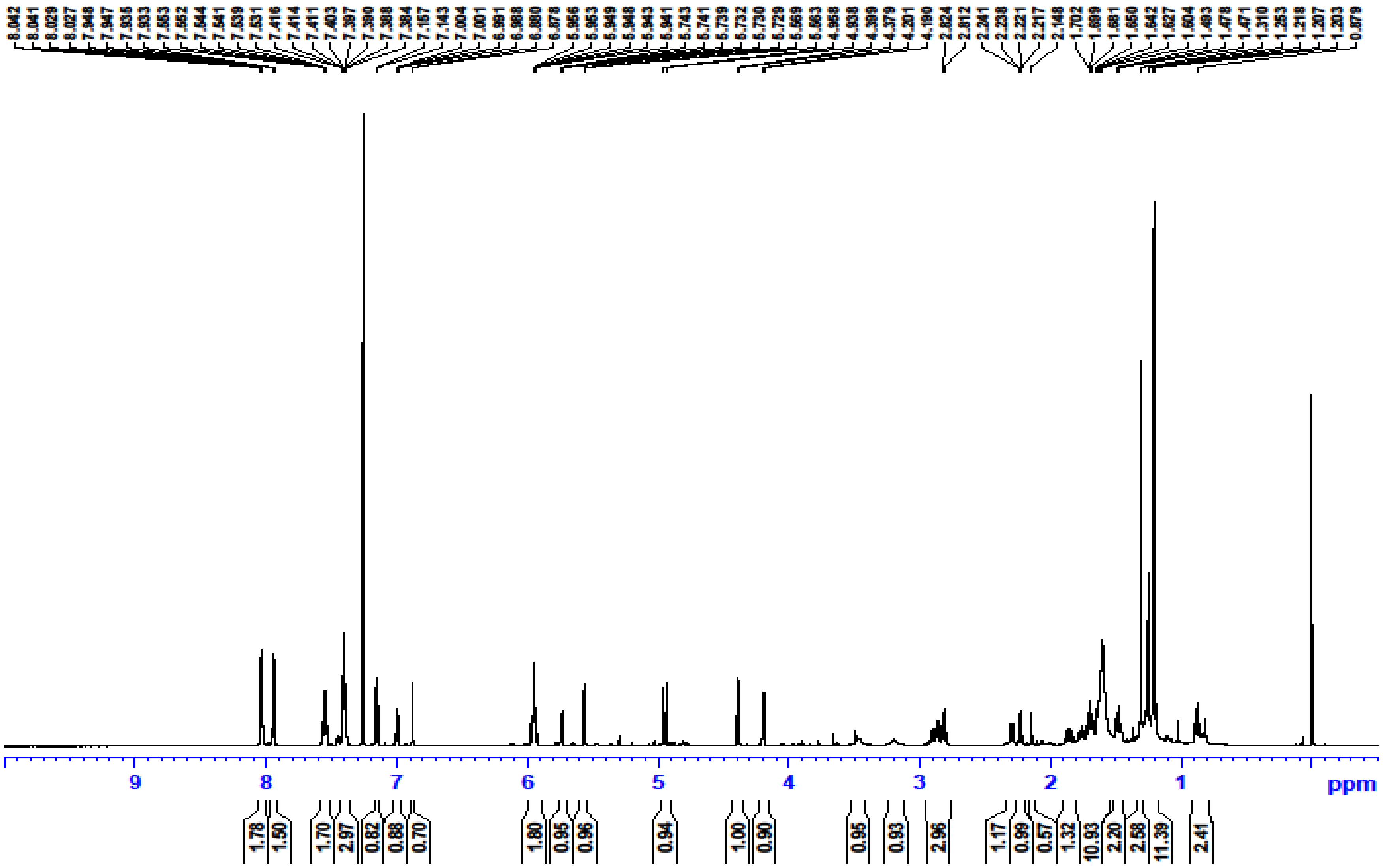

Figure S88. ^{1}H NMR (600 MHz) spectrum of compound **9** in $CDCl_3$

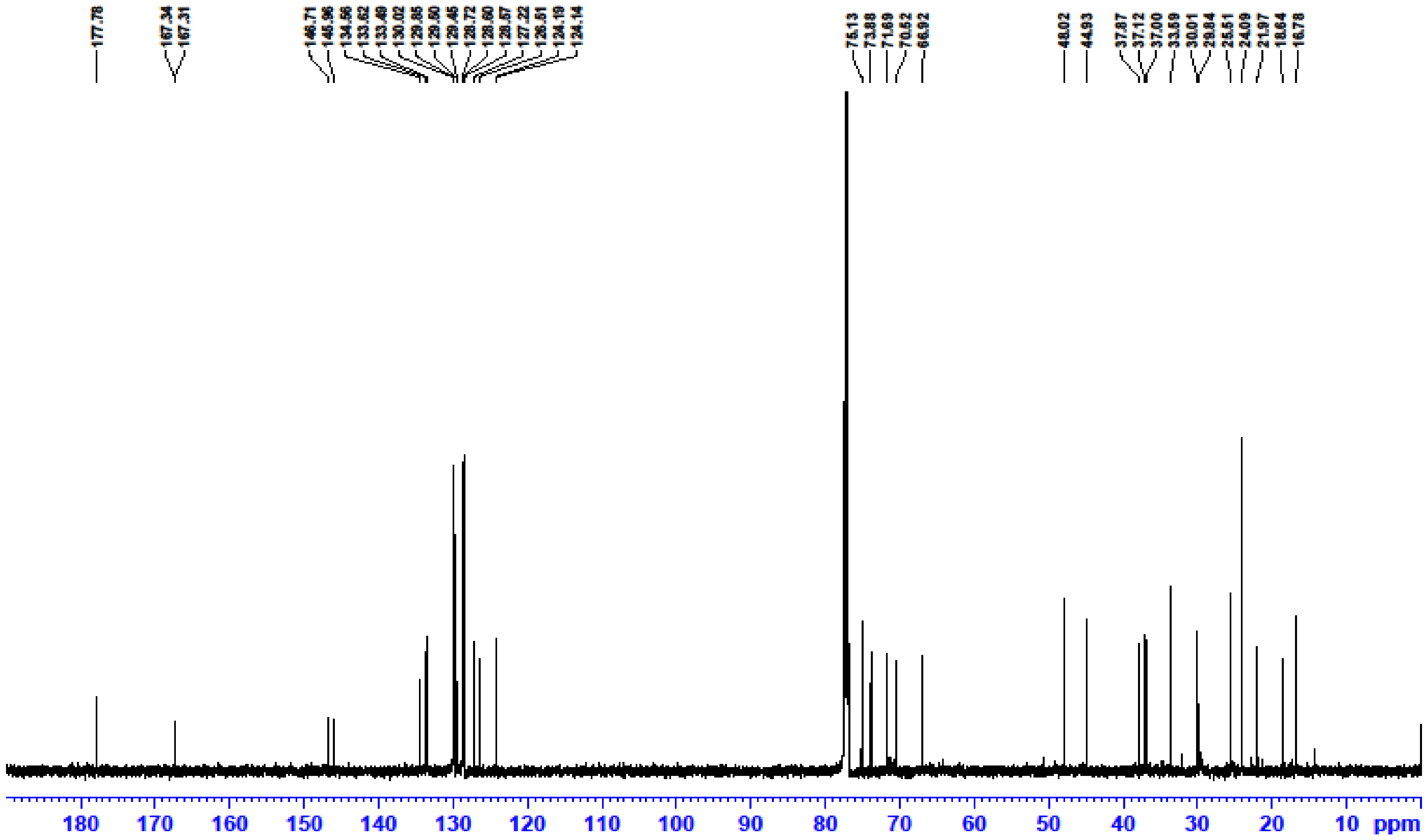

Figure S89. ^{13}C NMR (150 MHz) spectrum of compound **9** in $CDCl_3$

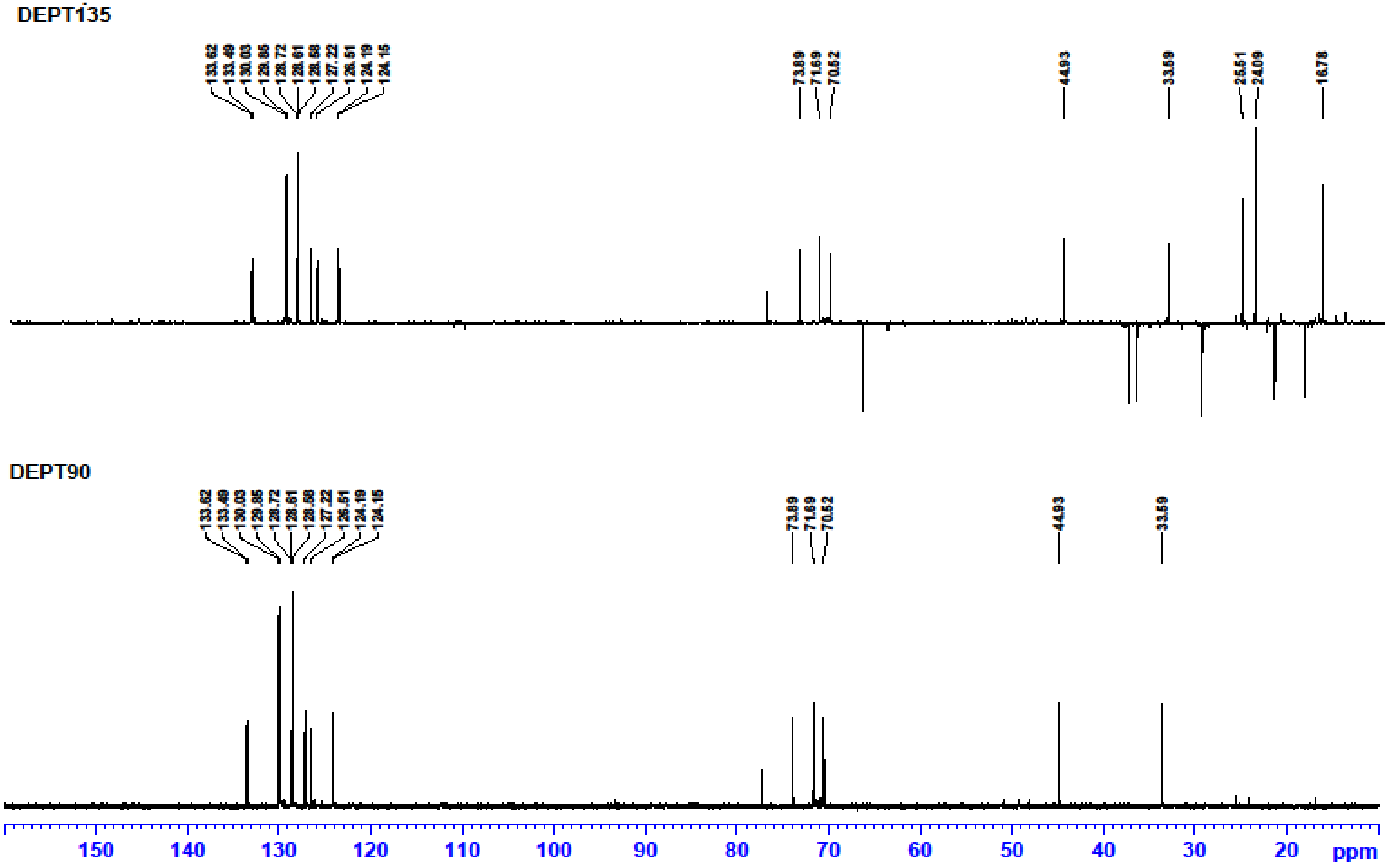

Figure S90. DEPT 135 & 90 NMR spectrum of compound **9** in $CDCl_3$

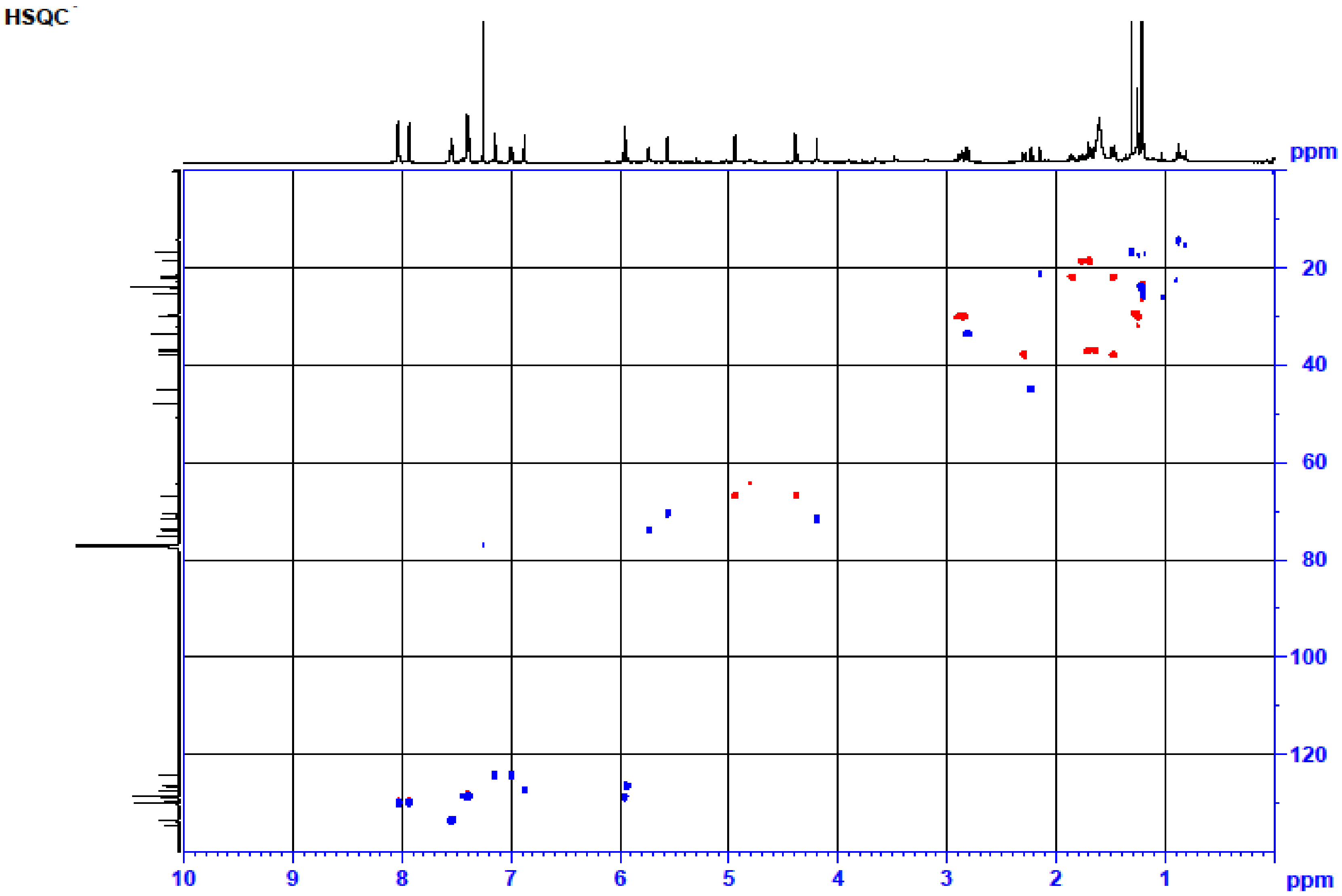

Figure S91. HSQC spectrum of compound **9** in $CDCl_3$

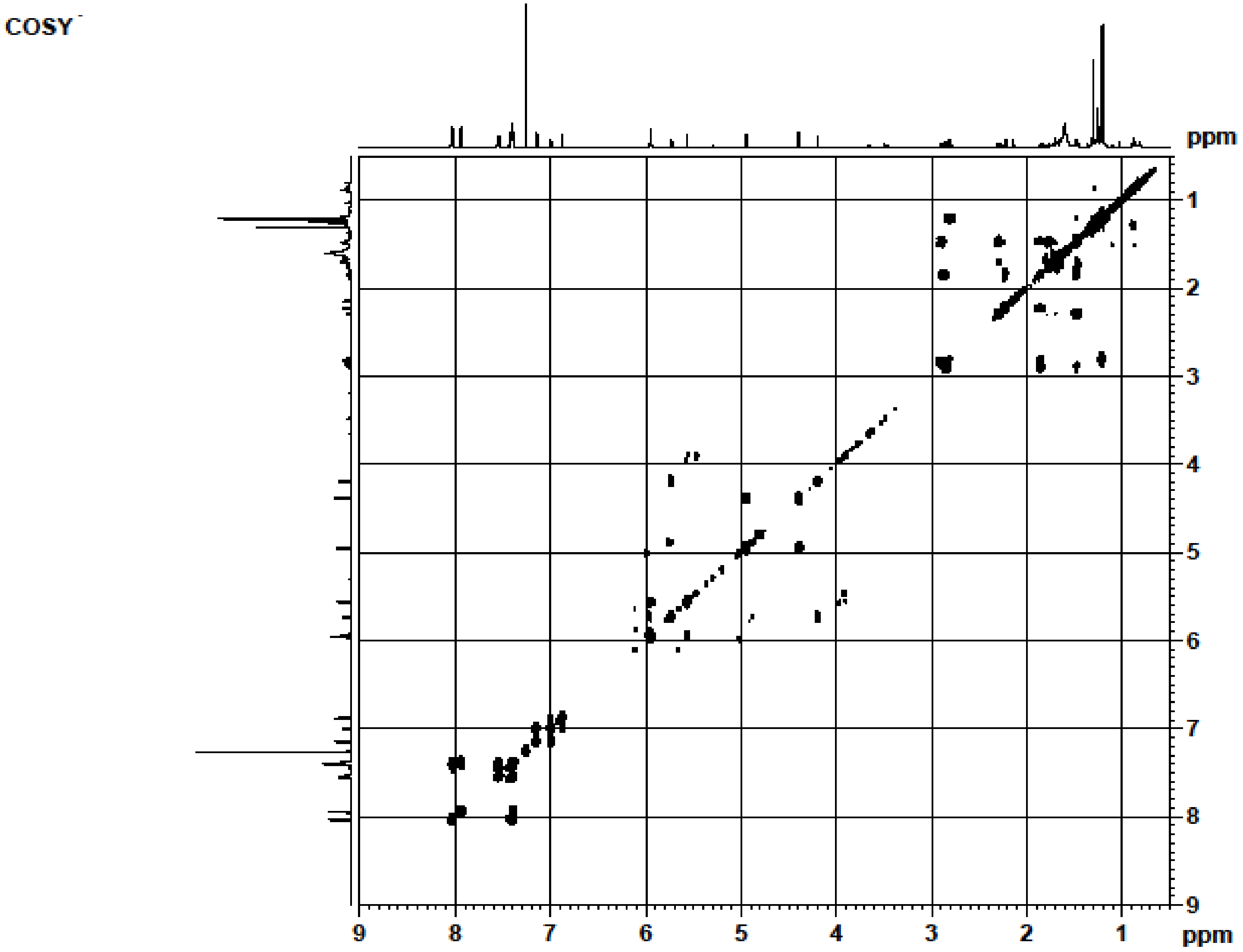

Figure S 92. COSY spectrum of compound **9** in $CDCl_3$

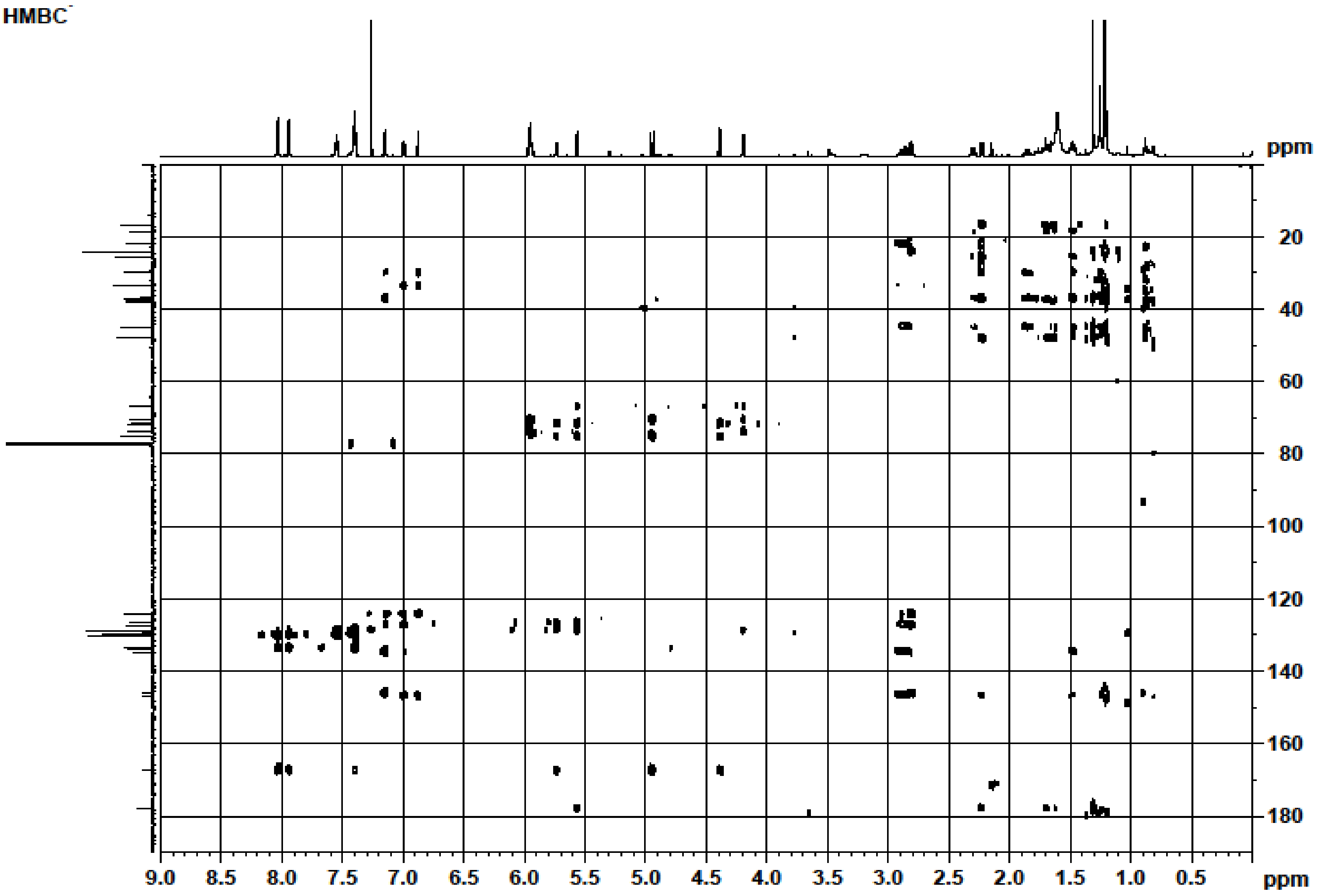

Figure S93. HMBC spectrum of compound **9** in $CDCl_3$

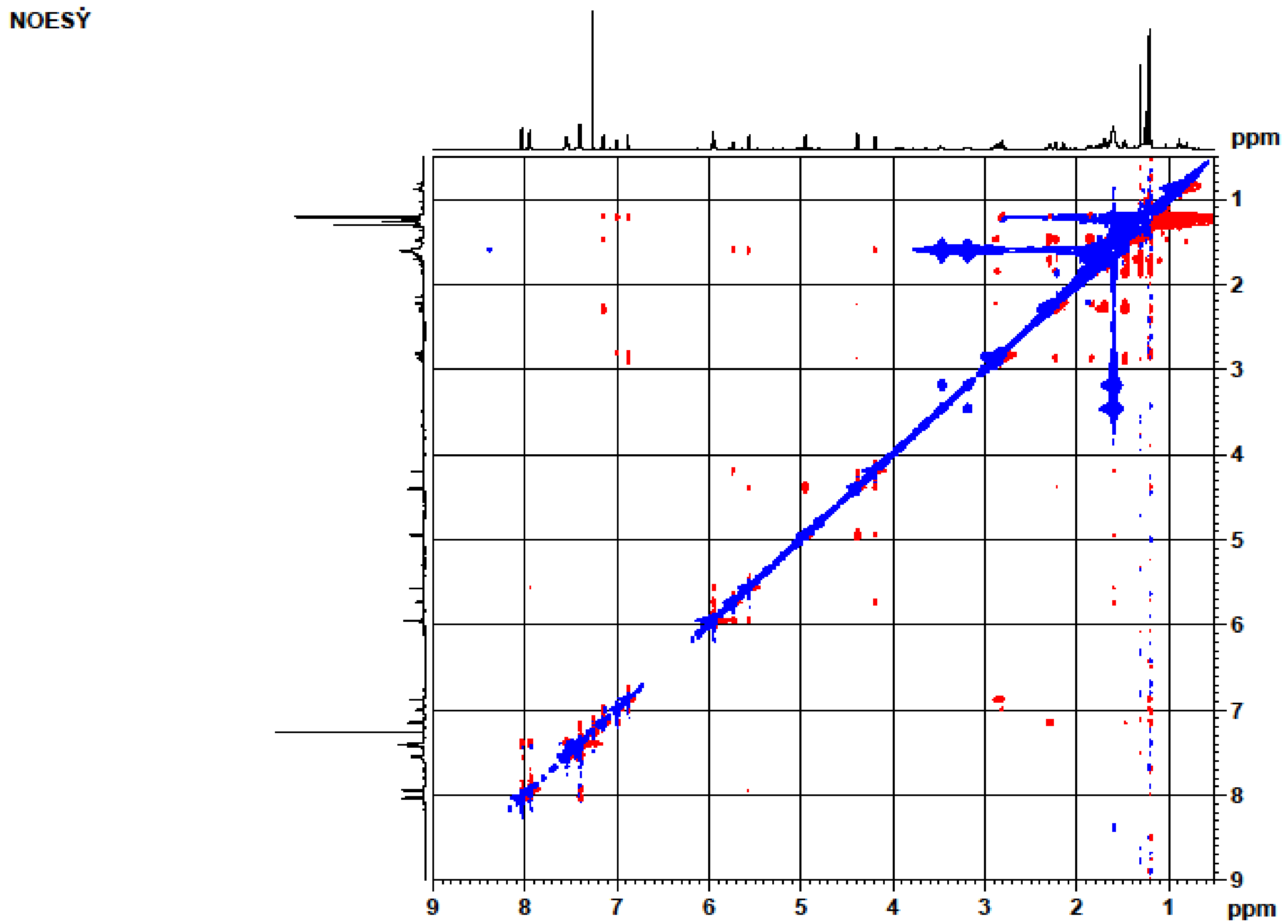

Figure S94. NOESY spectrum of **9** in $CDCl_3$

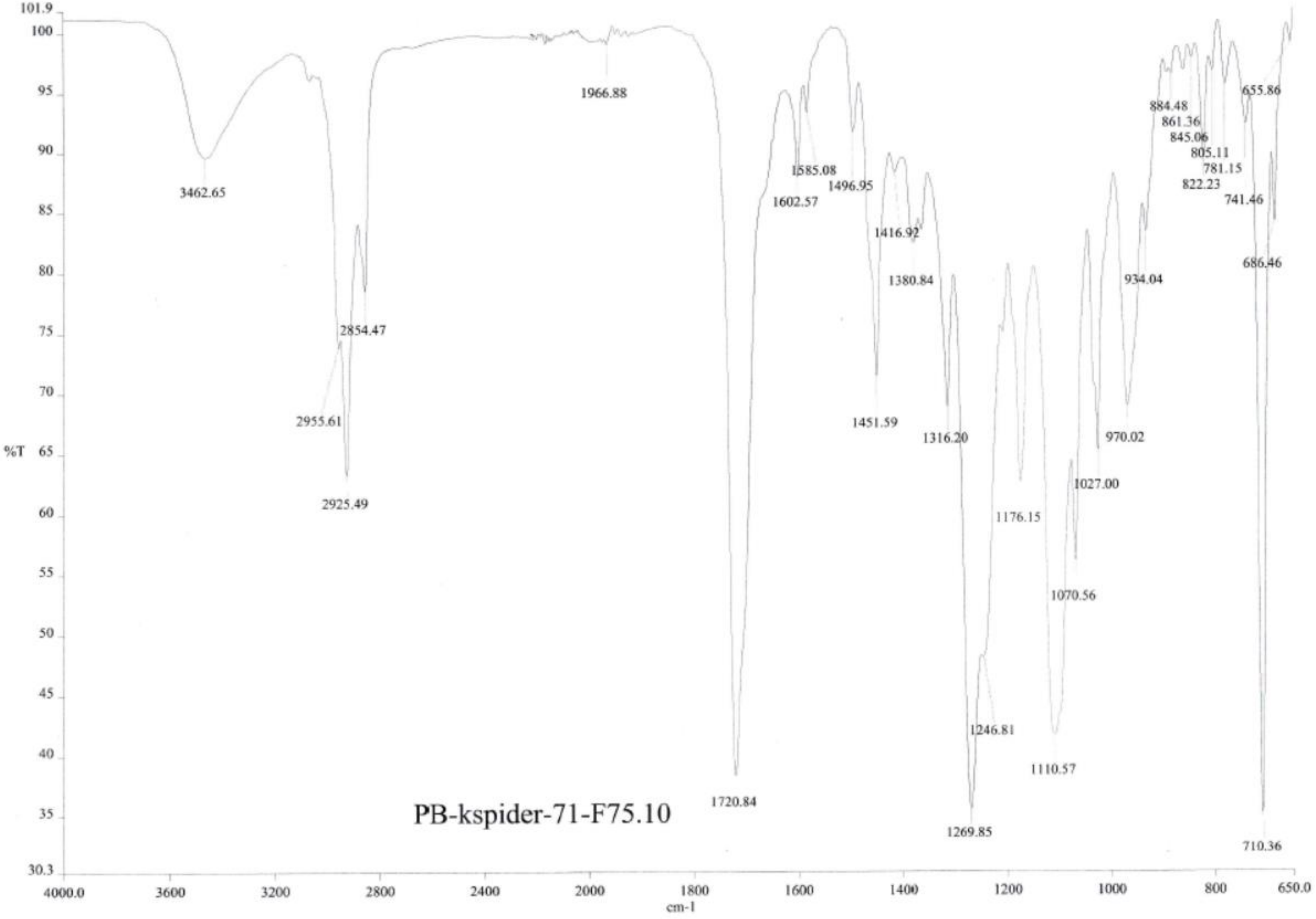

Figure S95. IR spectrum of compound **9**

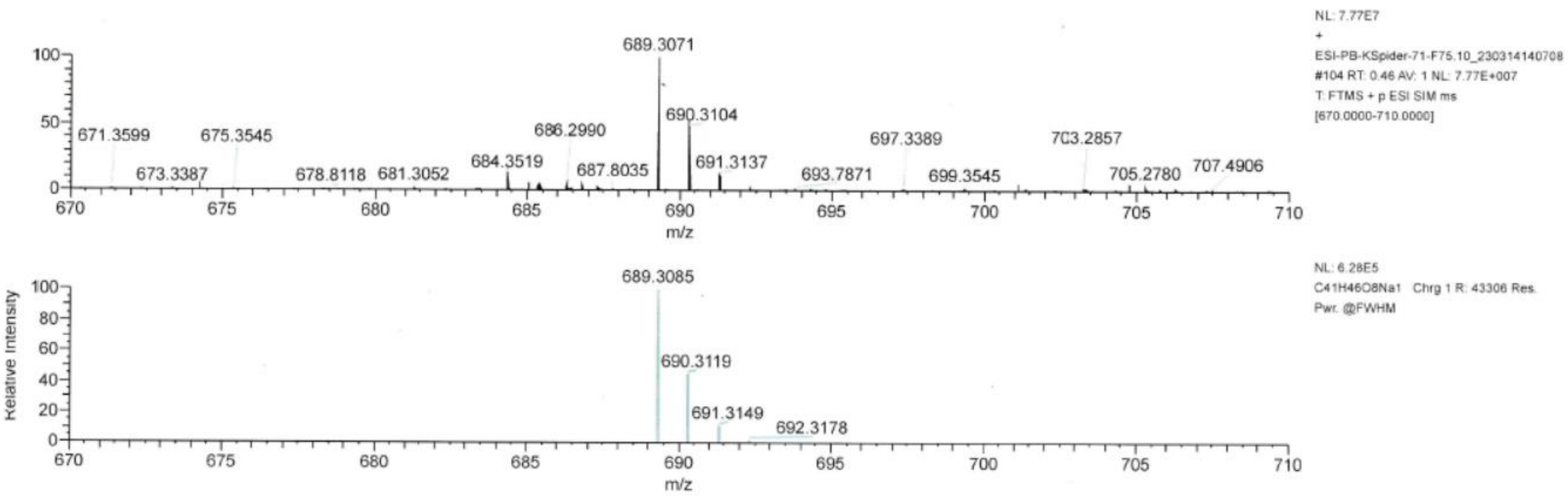

Peak Mass	Display Formula	Delta [ppm]	Theo. mass	MSMS Matched Fragments
689.3071	$C_{41}H_{46}O_8{}^{23}Na$ [M+Na]$^+$	-2.06	689.30849	(Collection)
689.3071	$C_{25}H_{53}O_{21}$	-0.46	689.30738	(Collection)
689.3071	$C_{50}H_{41}O_3$	2.97	689.30502	(Collection)
689.3071	$C_{23}H_{54}O_{21}{}^{23}Na$	3.03	689.30498	(Collection)
689.3071	$H_{65}O_{39}$	-3.89	689.30975	(Collection)
689.3071	$C_7H_{61}O_{34}$	4.63	689.30387	(Collection)

Figure S96. HRESIMS spectrum of compound **9**

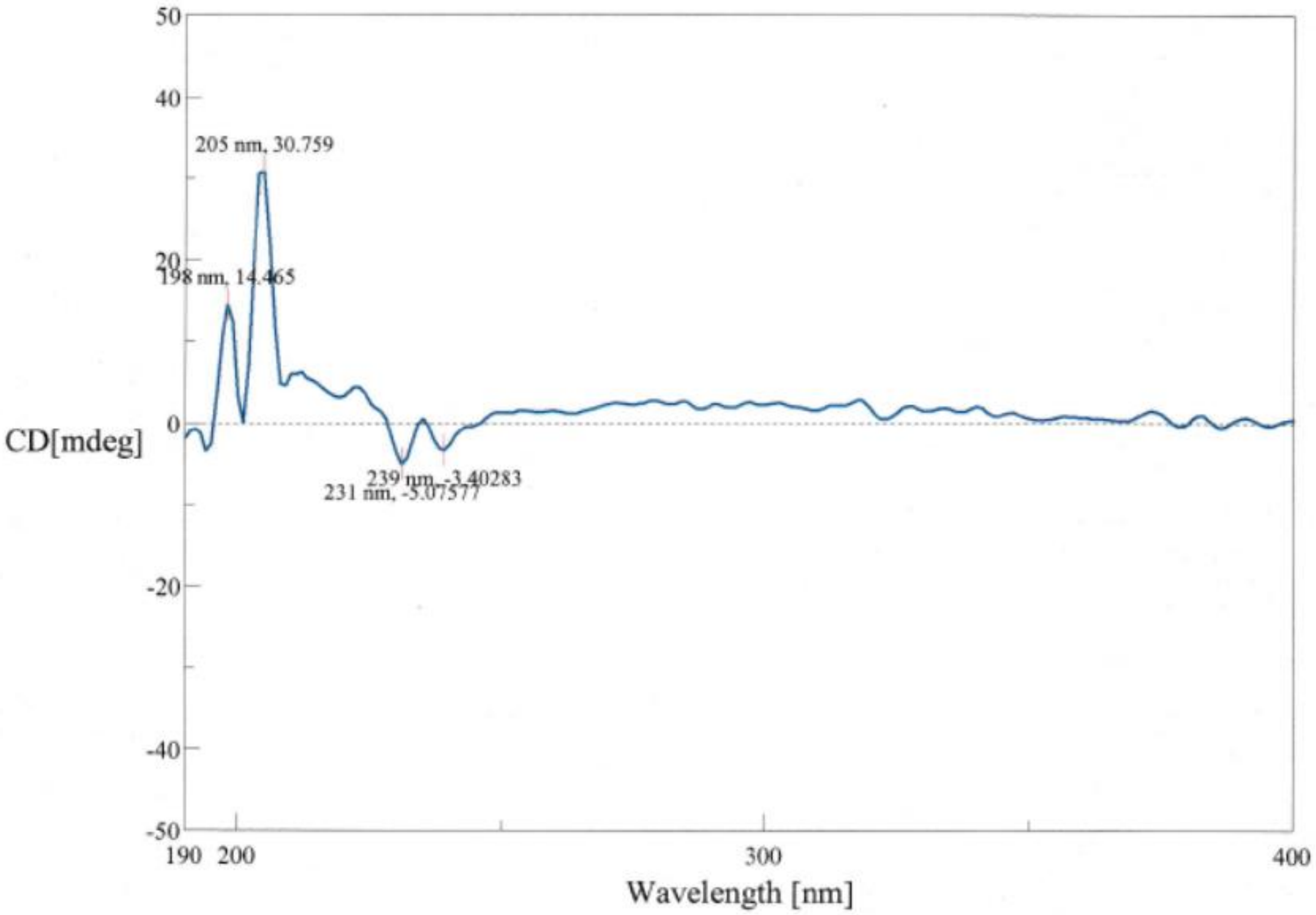

Figure S97. CD spectrum of compound **9**

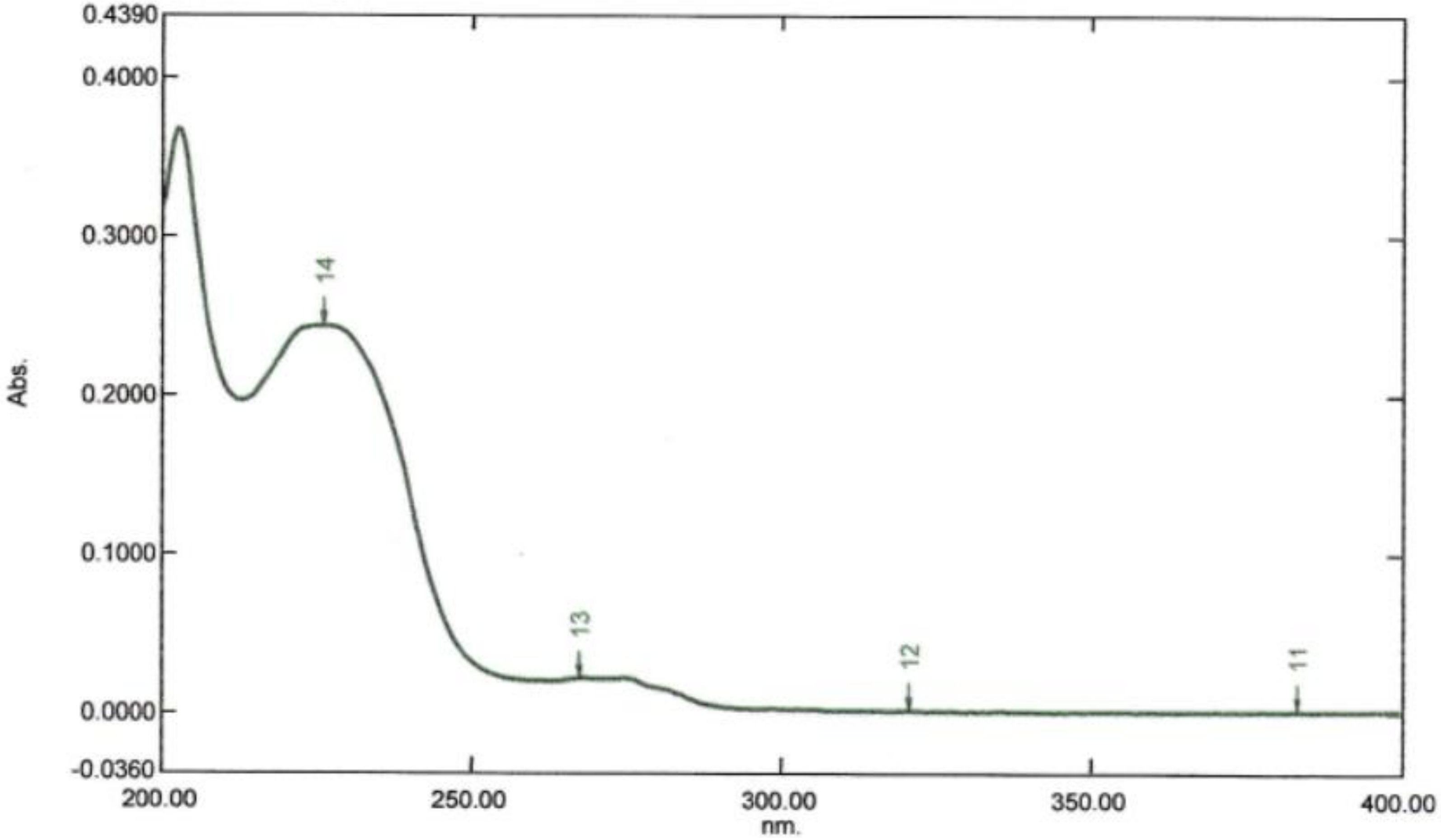

Figure S98. UV spectrum of compound **9**

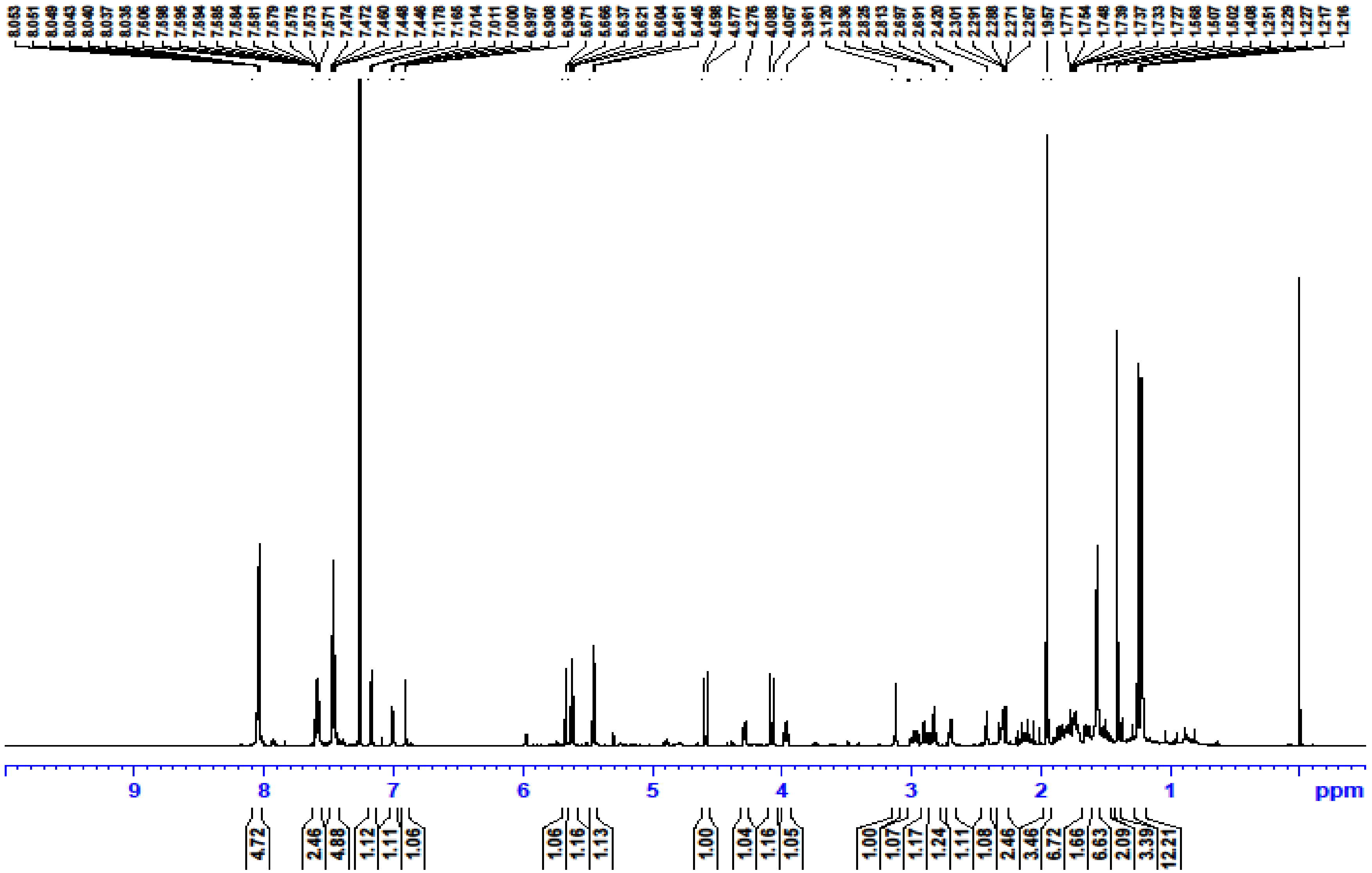

Figure S99. ^{1}H NMR (600 MHz) spectrum of compound **10** in $CDCl_3$

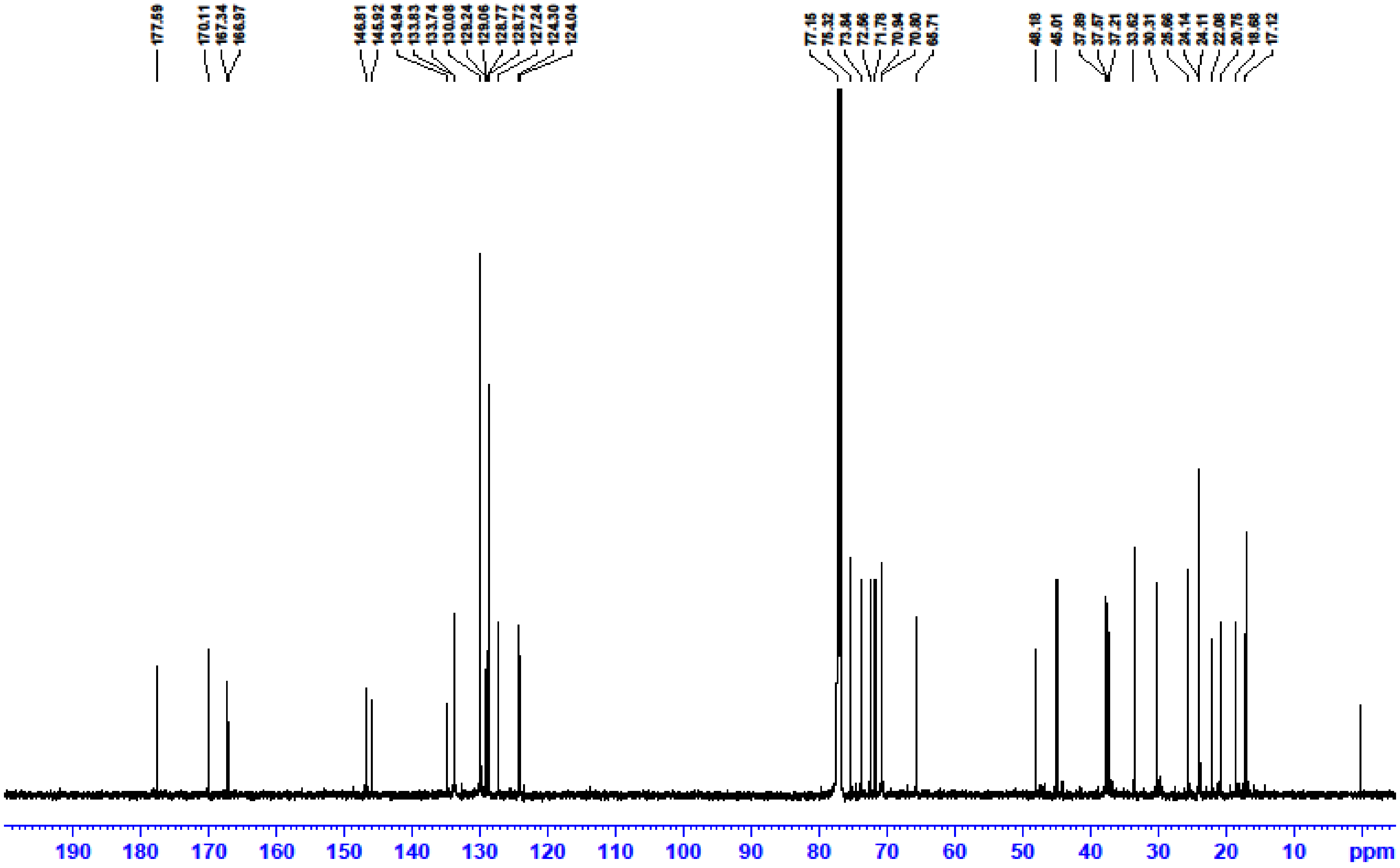

Figure S100. ^{13}C NMR (150 MHz) spectrum of compound **10** in $CDCl_3$

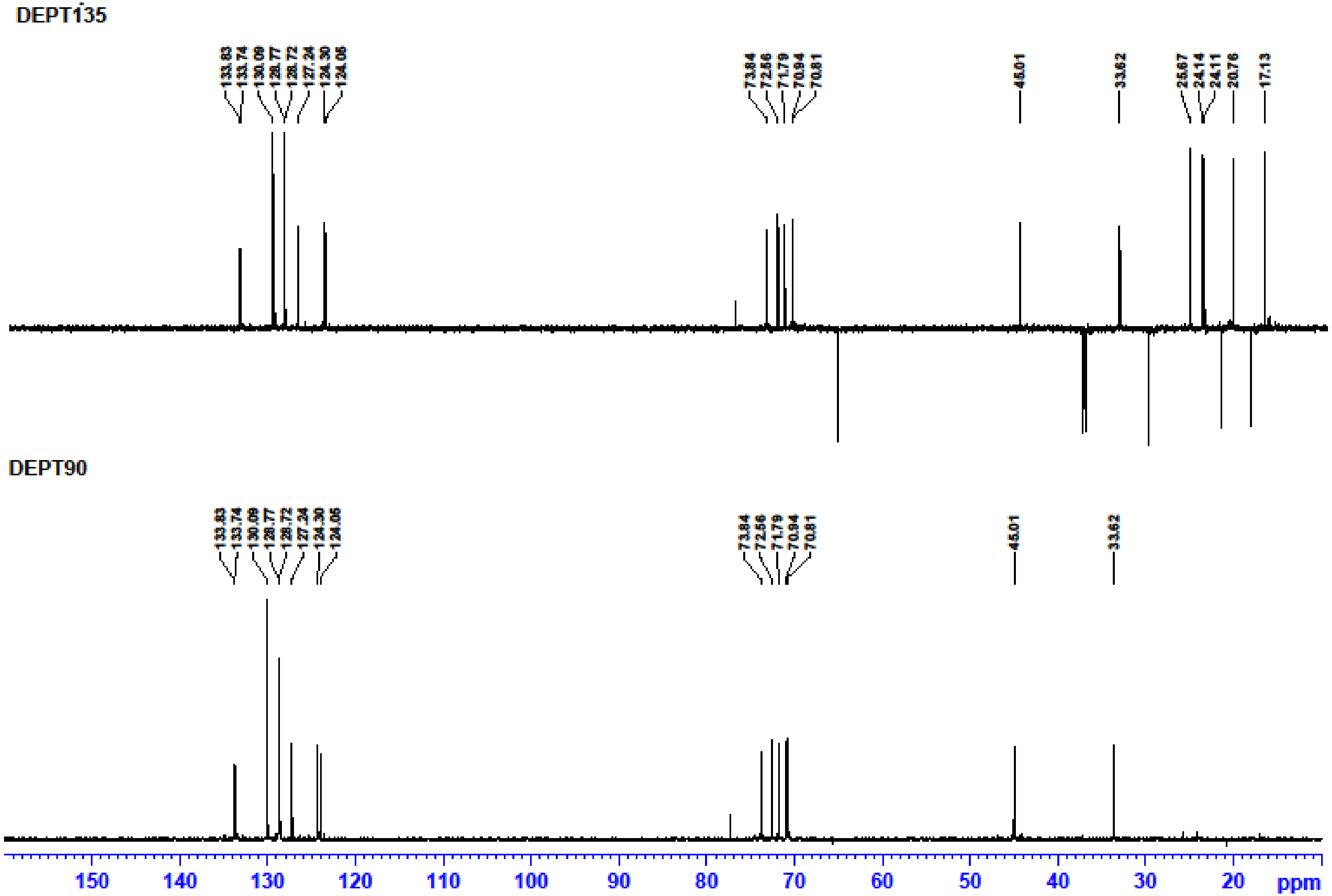

Figure S101. DEPT 135 & 90 NMR spectrum of compound **10** in $CDCl_3$

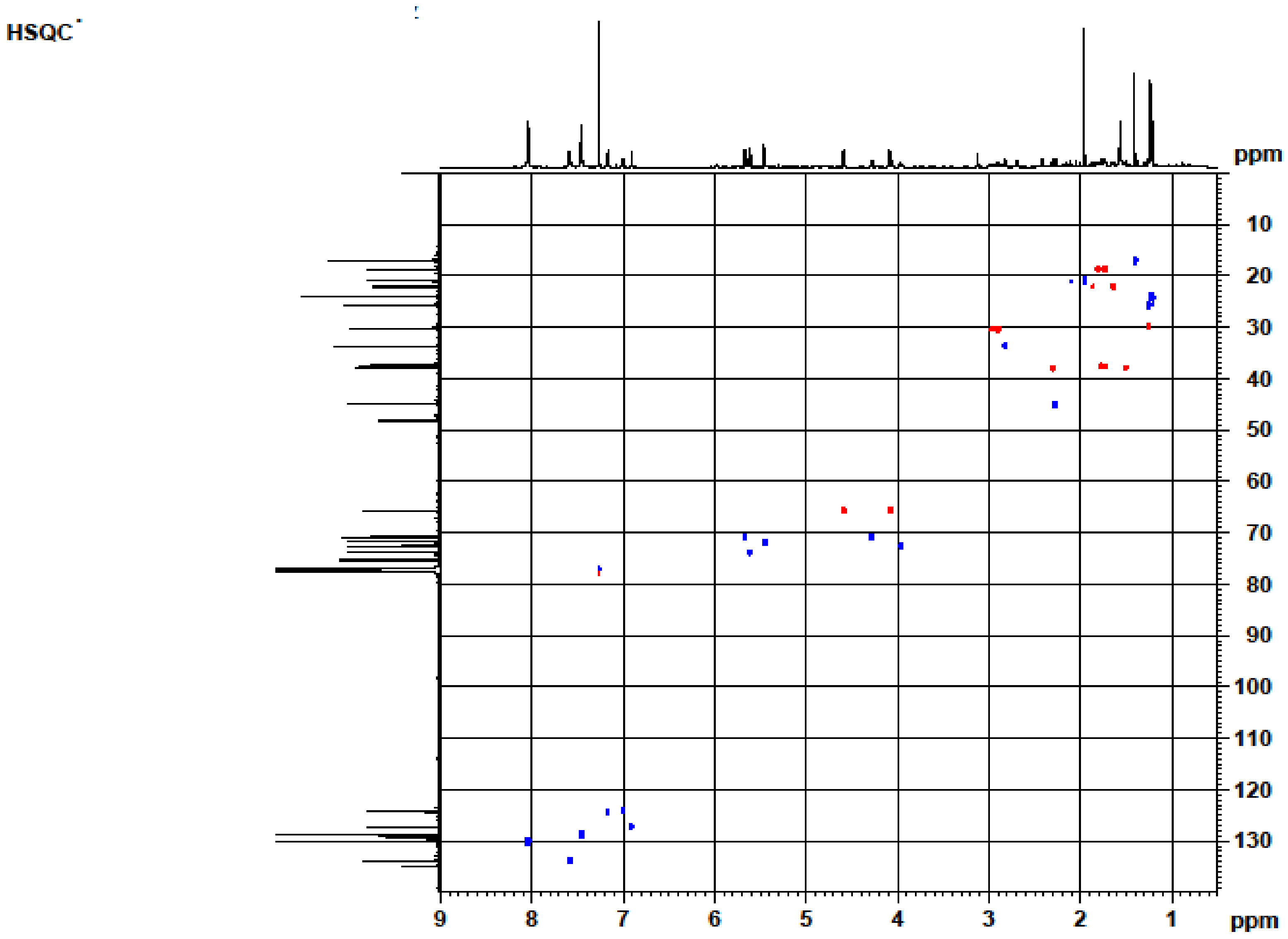

Figure S102. HSQC spectrum of compound **10** in $CDCl_3$

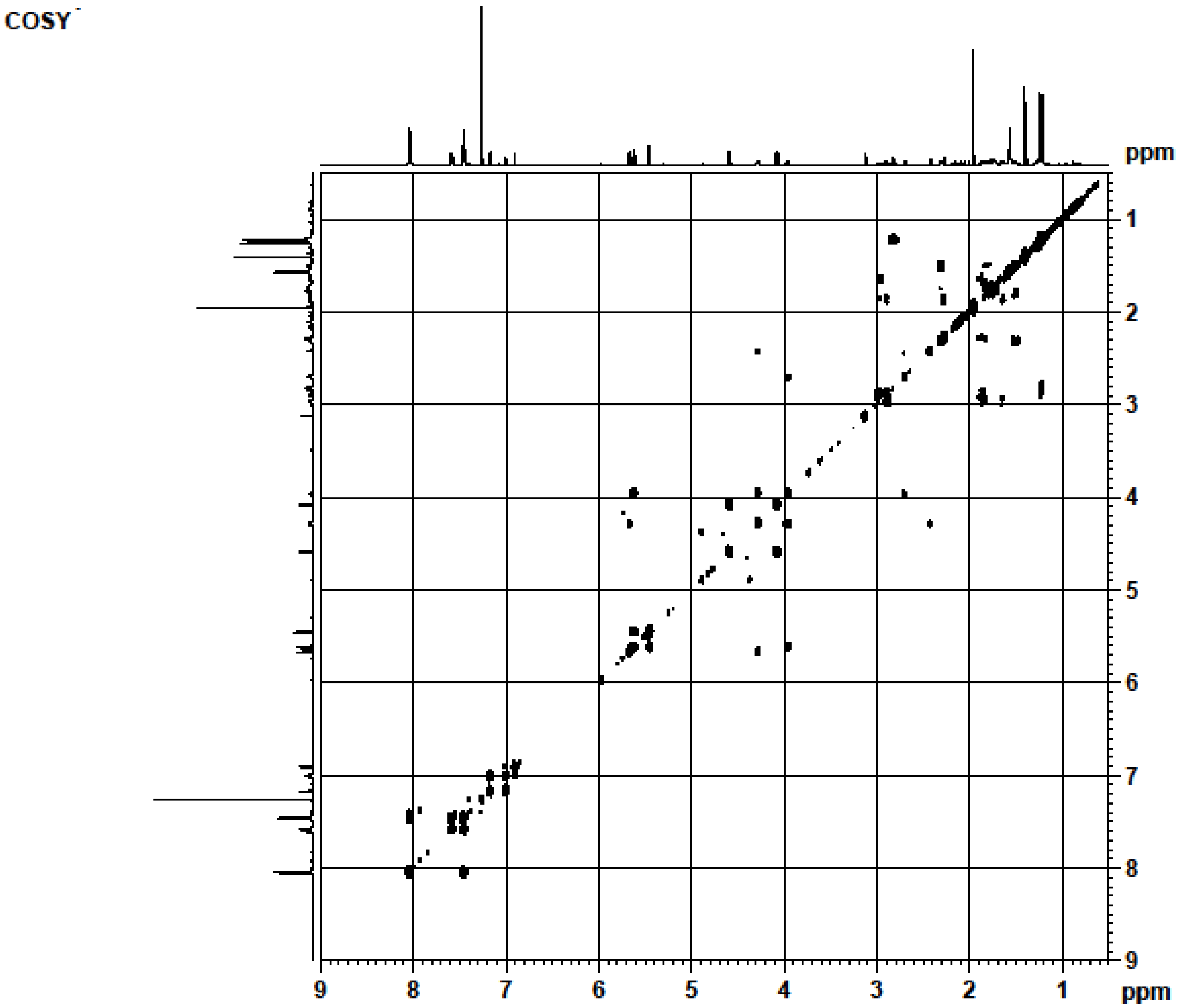

Figure S103. COSY spectrum of compound **10** in $CDCl_3$

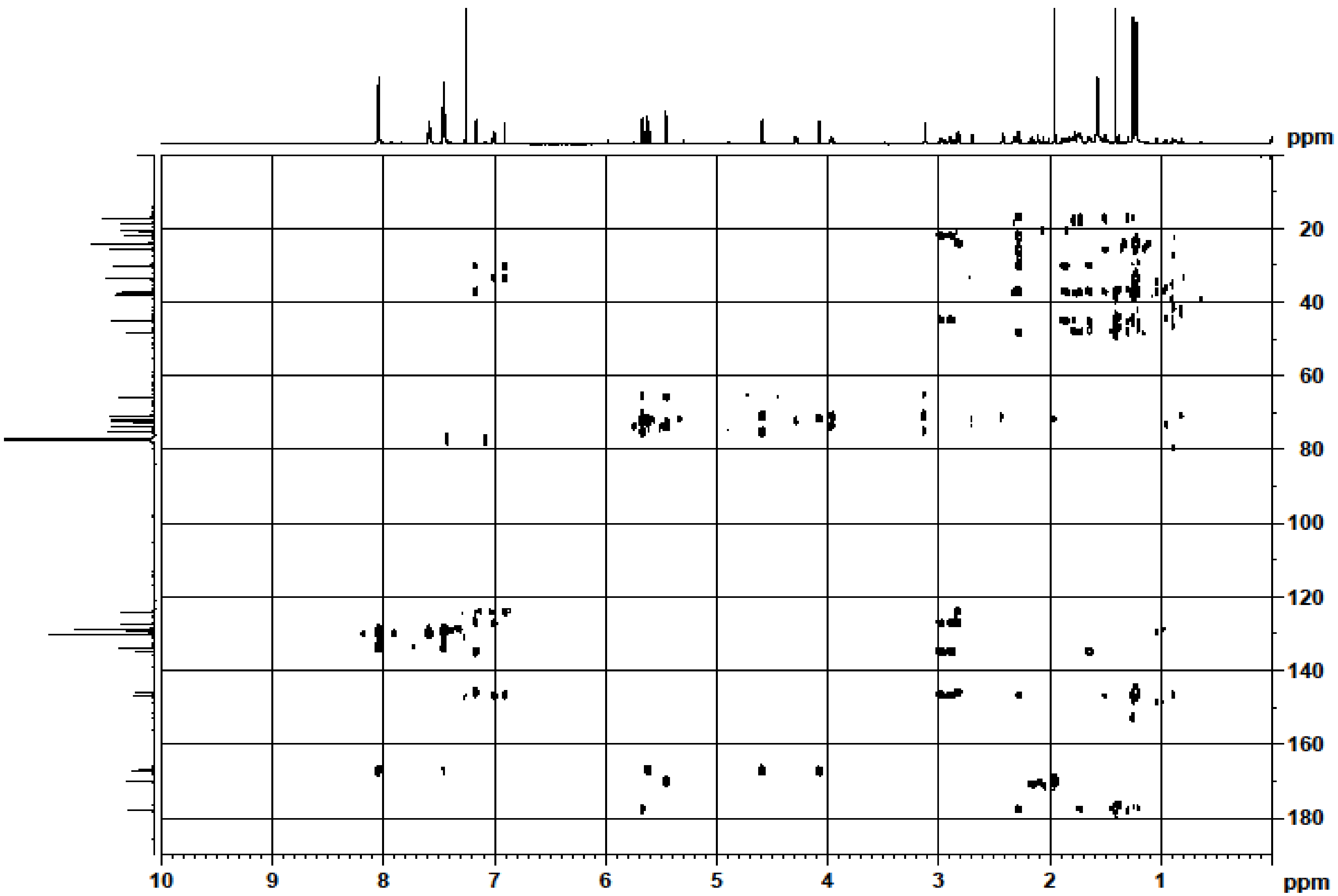

Figure S104. HMBC spectrum of compound **10** in $CDCl_3$

NOESY

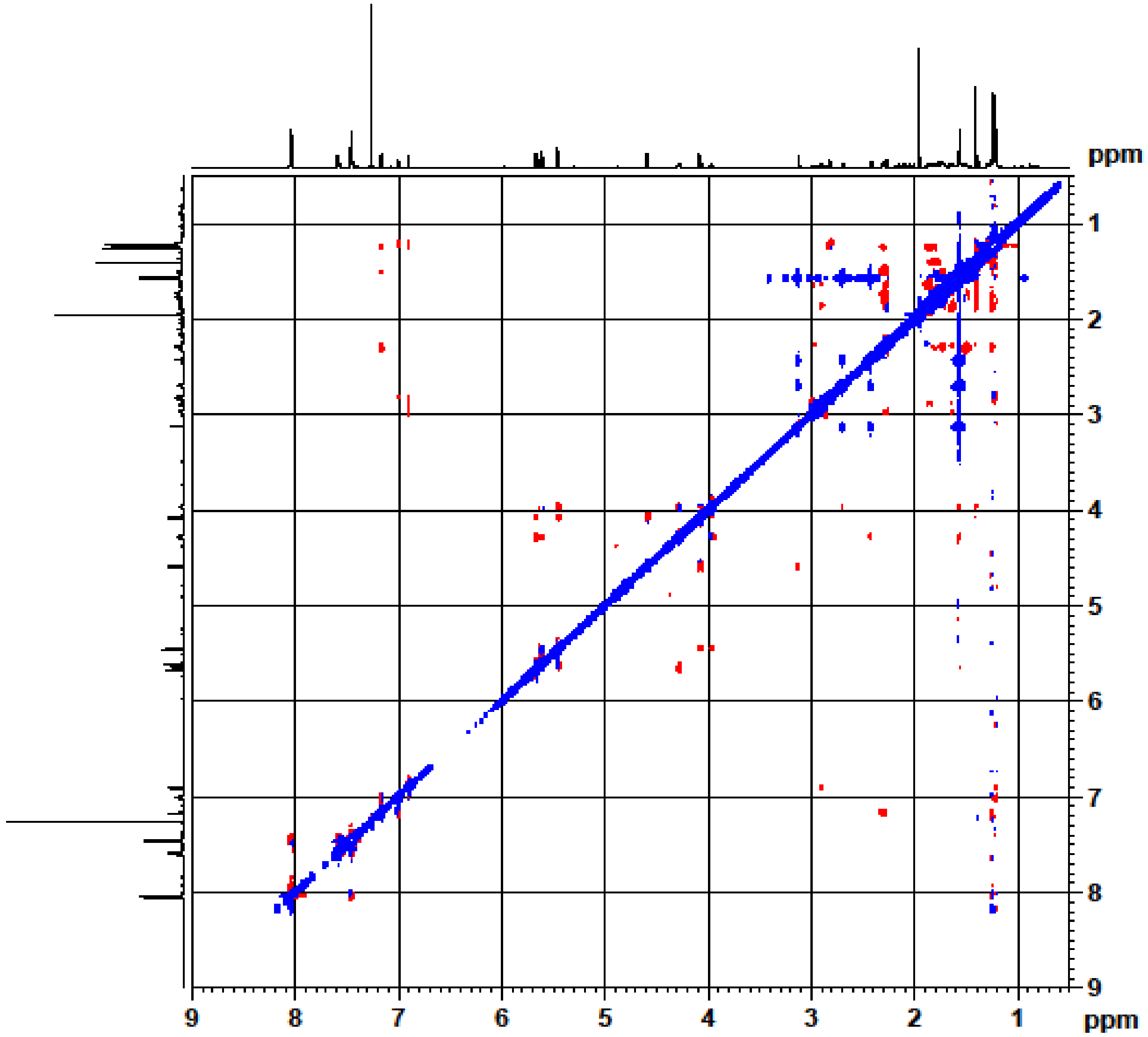

Figure S105. NOESY spectrum of **10** in $CDCl_3$

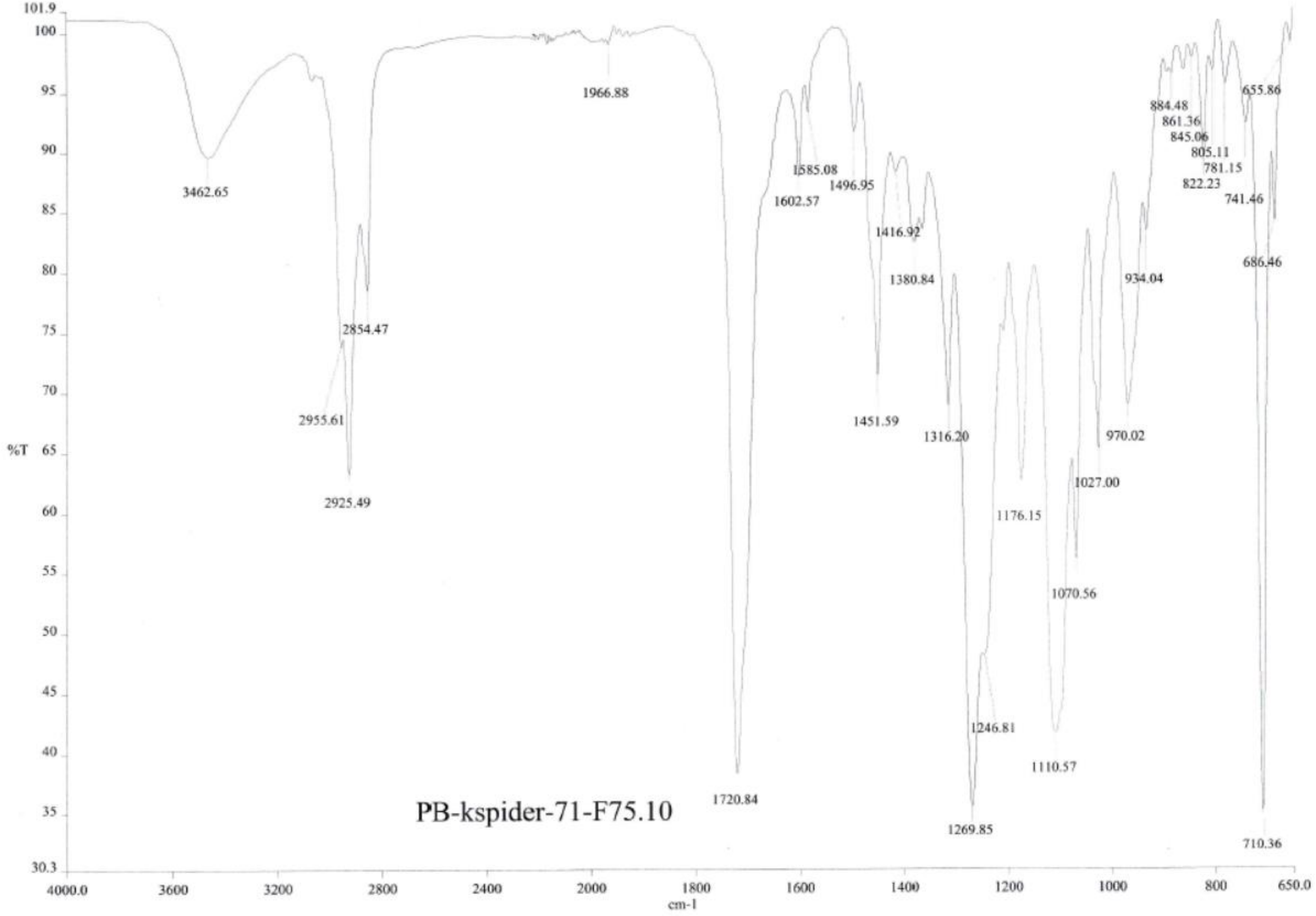

Figure S106. IR spectrum of compound **10**

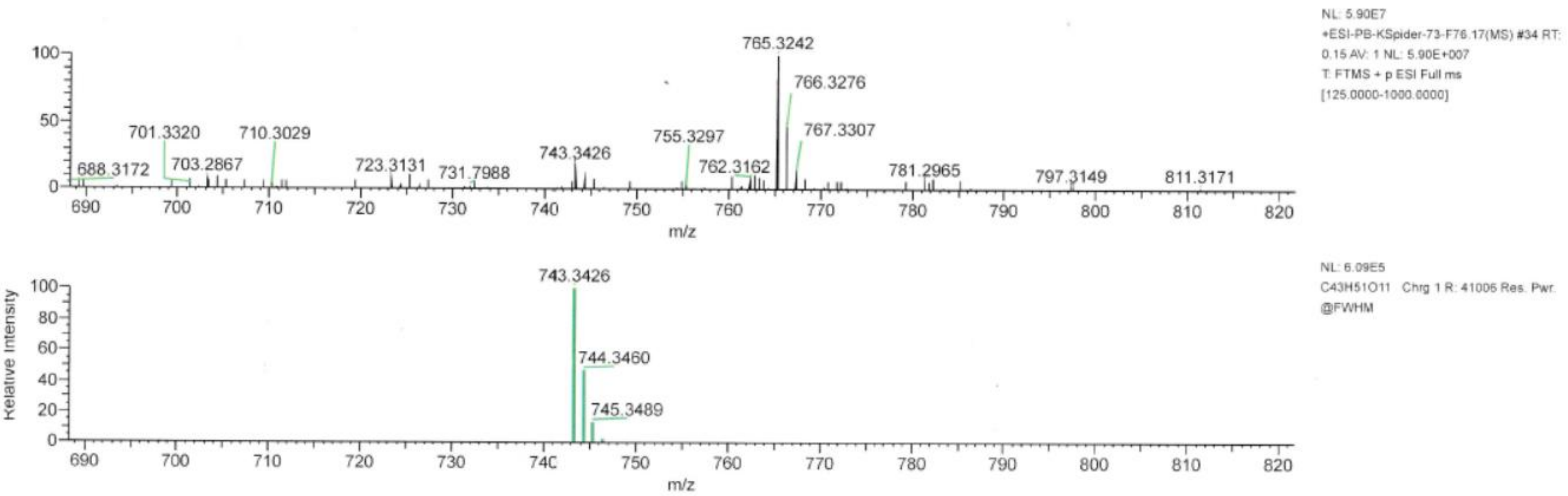

Peak Mass	Display Formula	Delta [ppm]	Theo. mass	MSMS Matched Fragments
743.3426	$C_{43}H_{51}O_{11}$ [M+H]$^+$	0.00	743.34259	(Collection)
743.3426	$H_{71}O_{42}$	1.54	743.34144	(Collection)
743.3426	$C_{18}H_{63}O_{29}$	-3.18	743.34495	(Collection)
743.3426	$C_{25}H_{59}O_{24}$	4.72	743.33908	(Collection)

Figure S107. HRESIMS spectrum of compound **10**

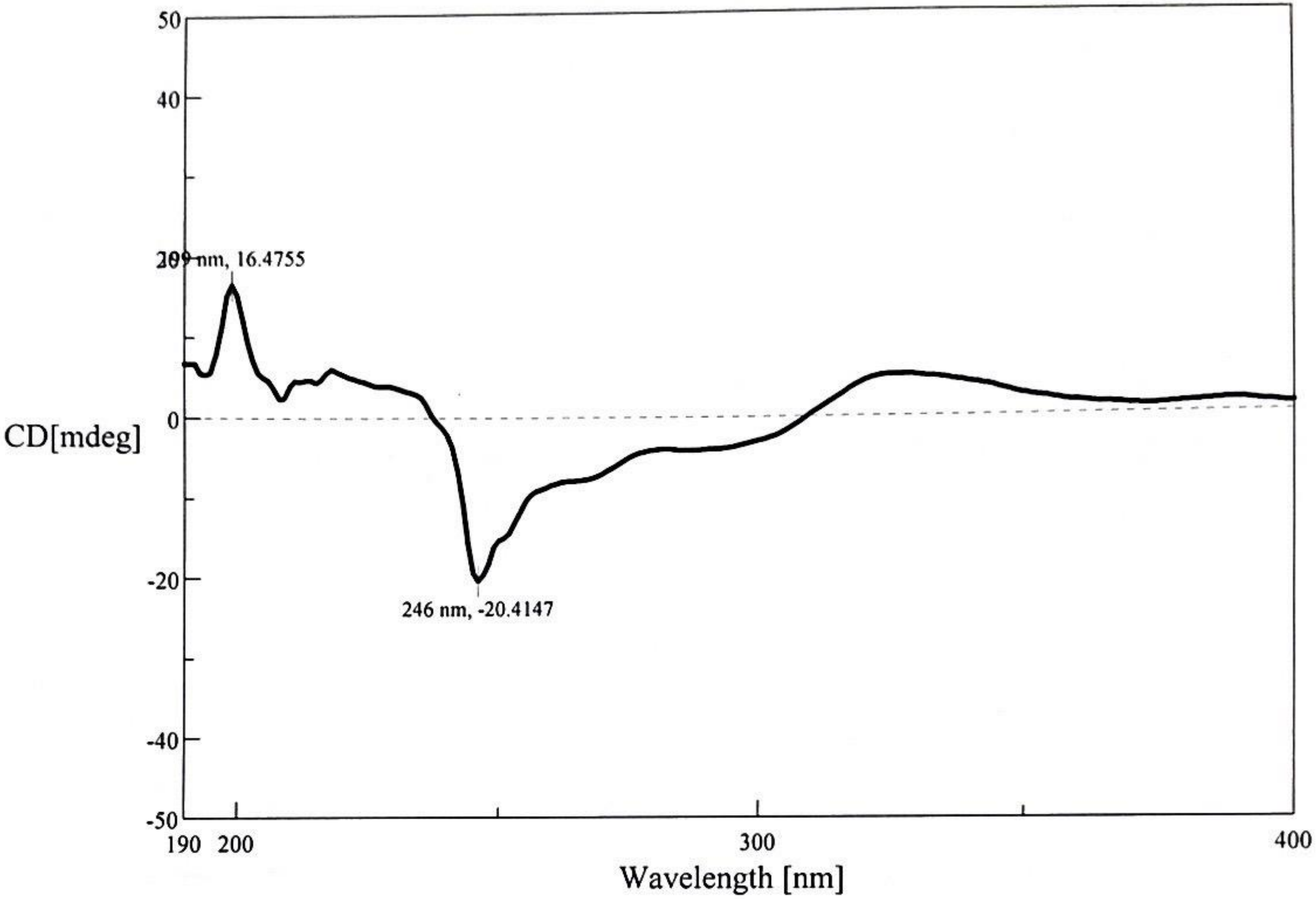

Figure S108. CD spectrum of compound **10**

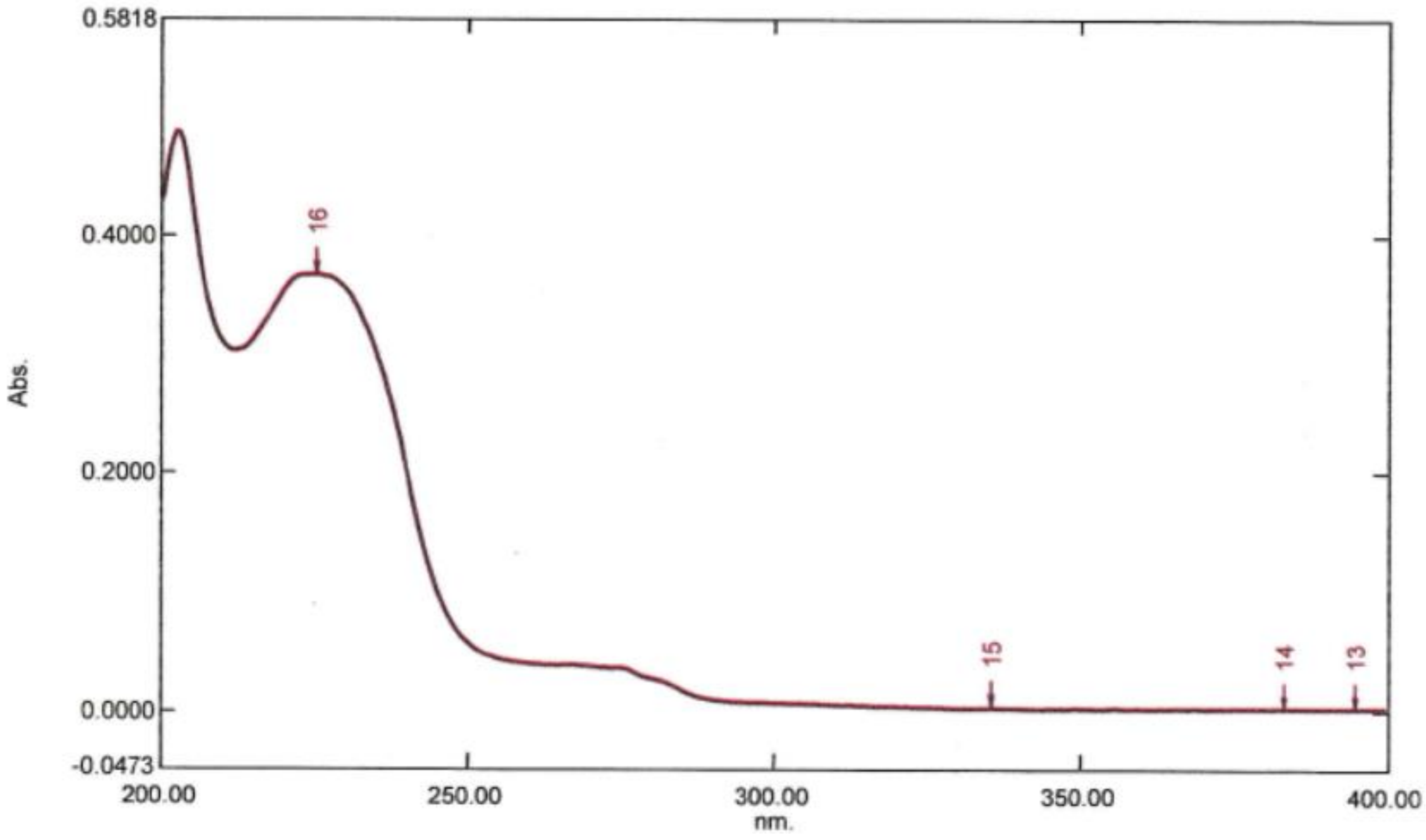

Figure S109. UV spectrum of compound **10**

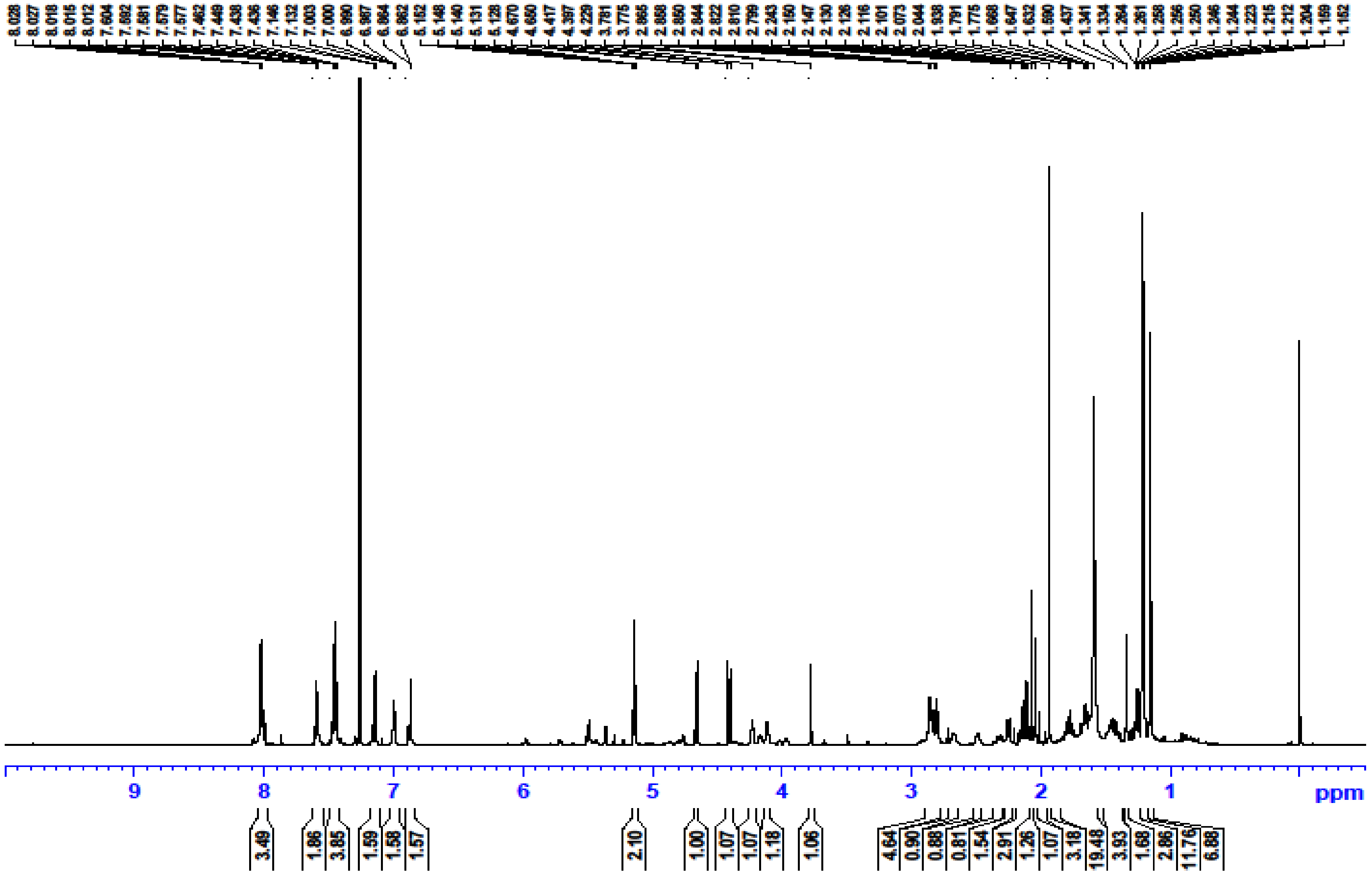

Figure S110. ^{1}H NMR (600 MHz) spectrum of compound **11** in $CDCl_3$

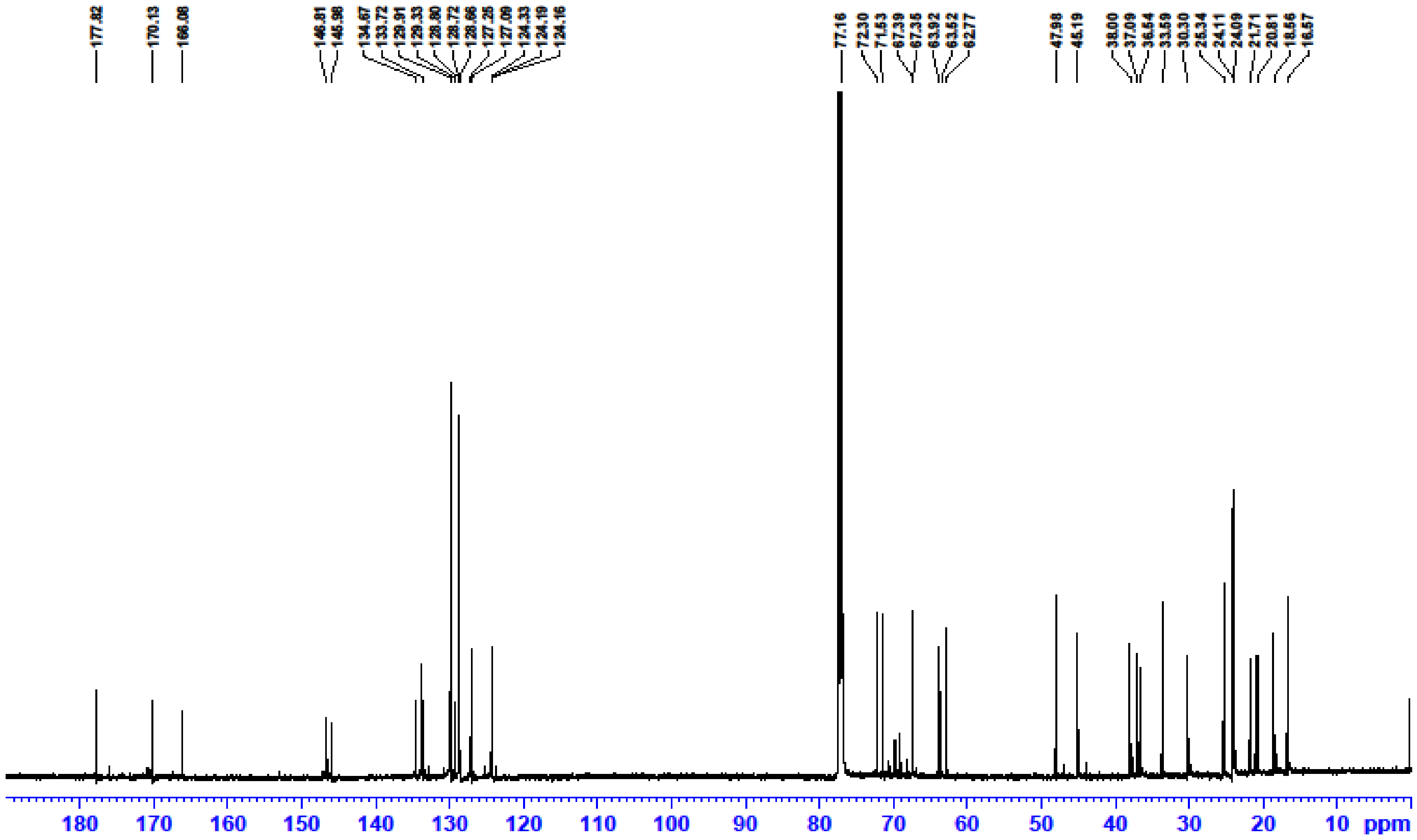

Figure S111. ^{13}C NMR (150 MHz) spectrum of compound **11** in $CDCl_3$

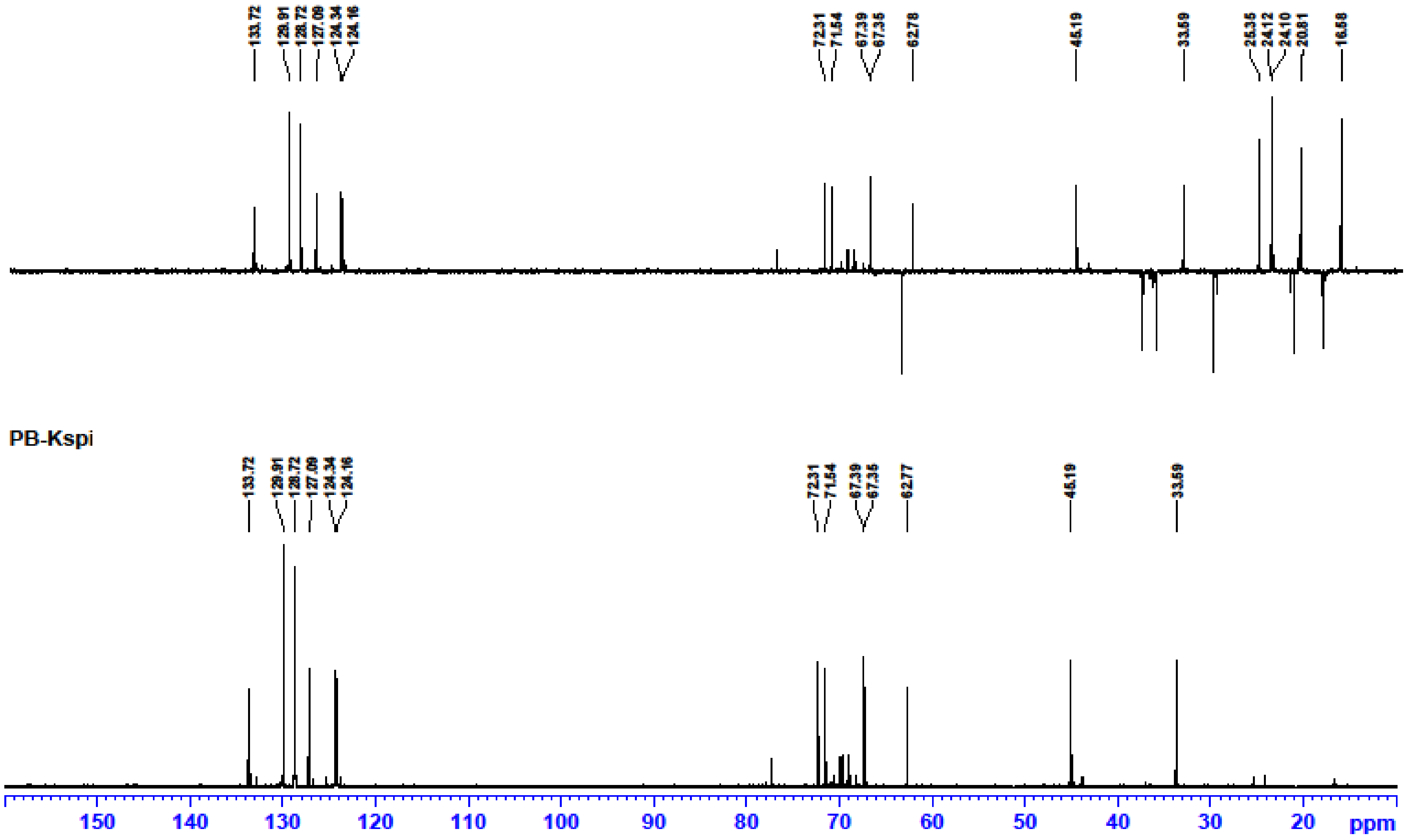

Figure S112. DEPT 135 & 90 NMR spectrum of compound **11** in $CDCl_3$

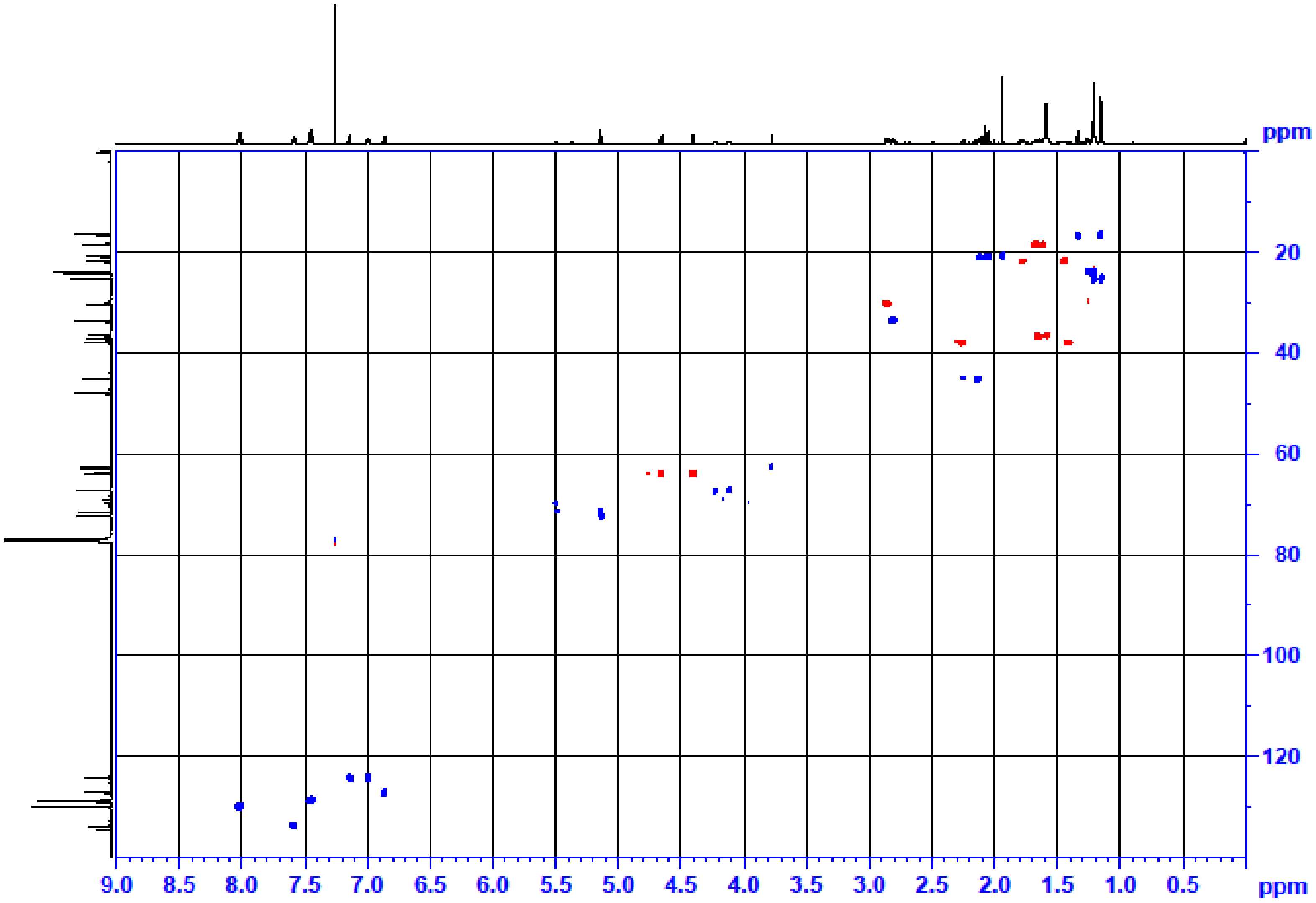

Figure S113. HSQC spectrum of compound **11** in $CDCl_3$

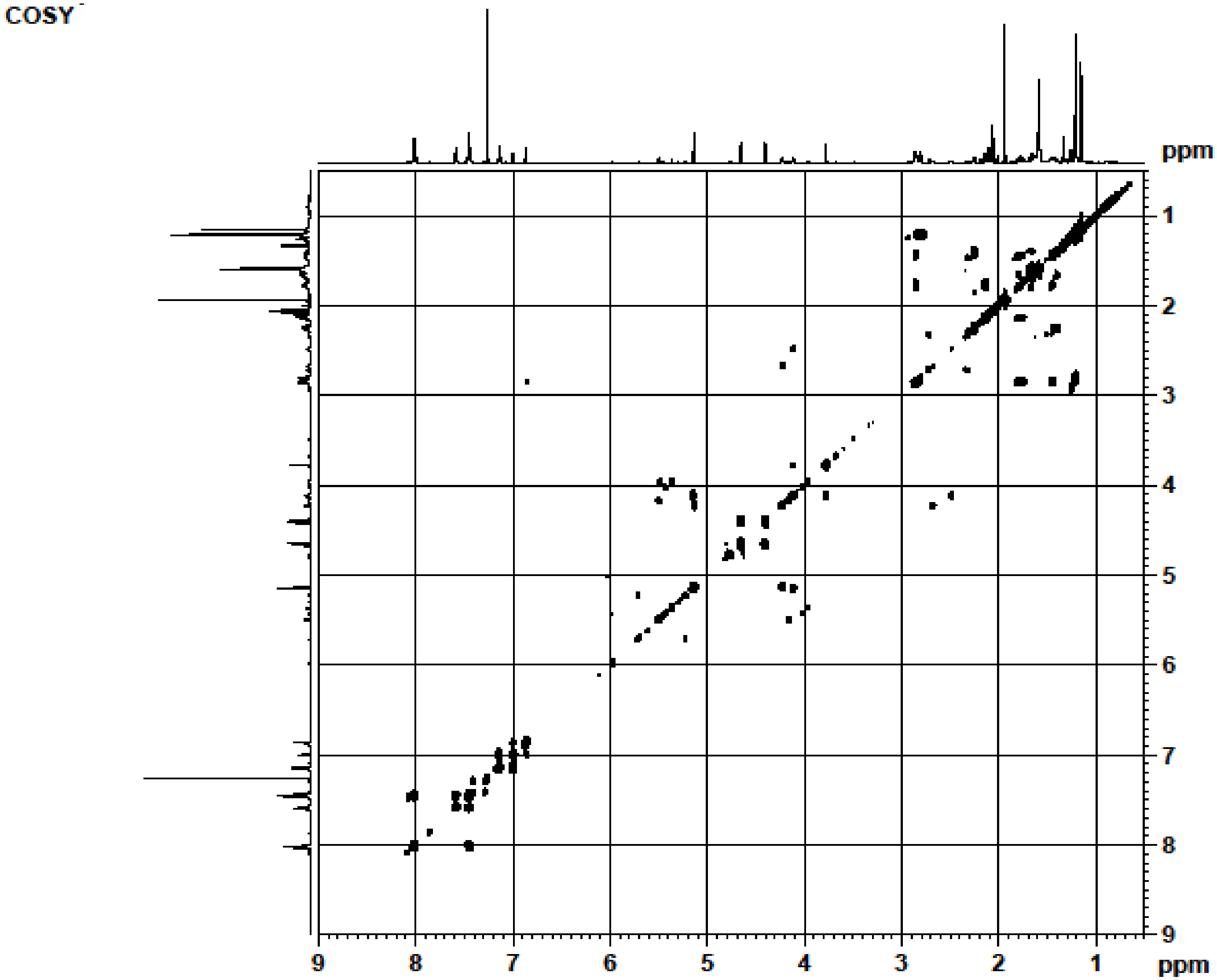

Figure S114. COSY spectrum of compound **11** in $CDCl_3$

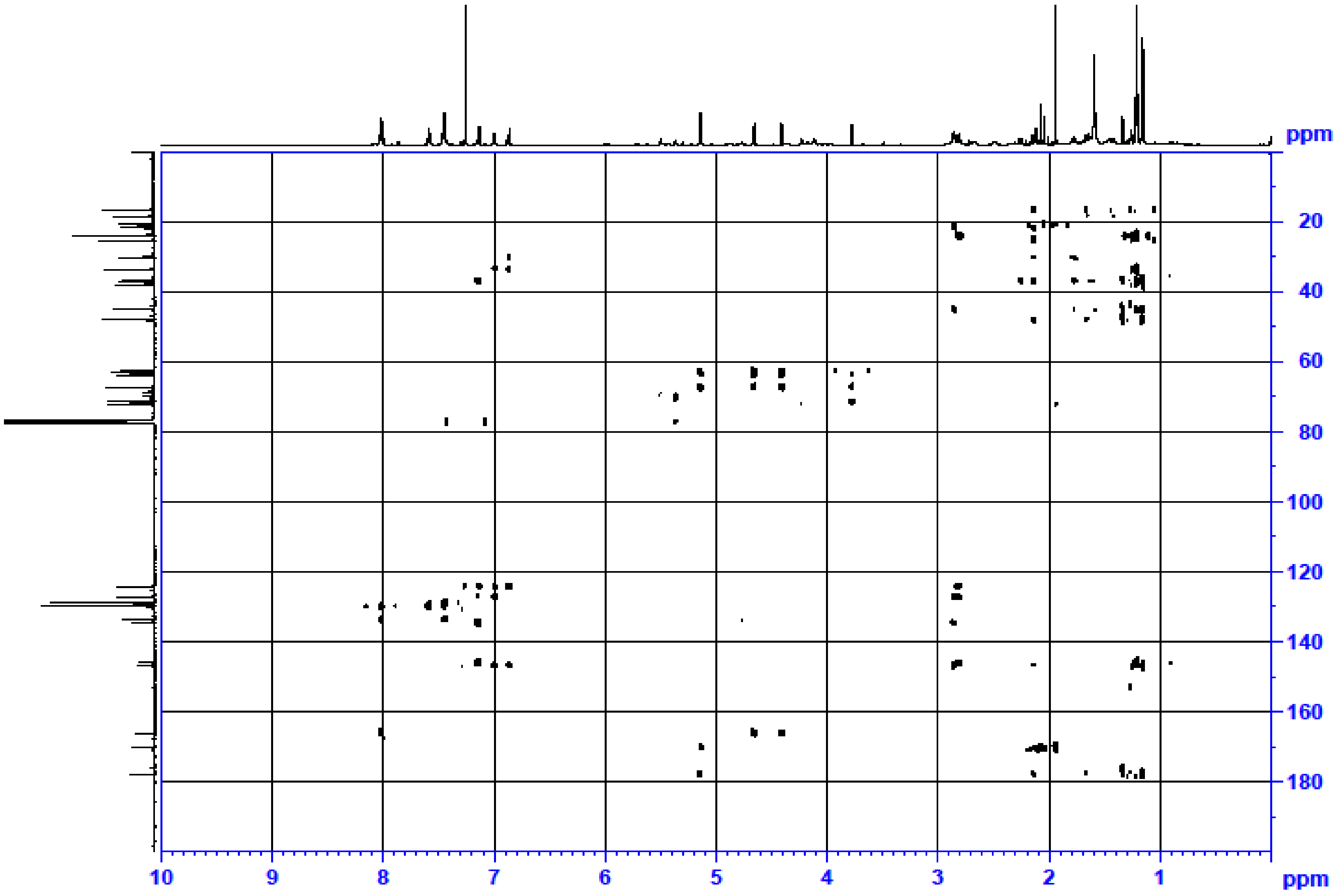

Figure S115. HMBC spectrum of compound **11** in $CDCl_3$

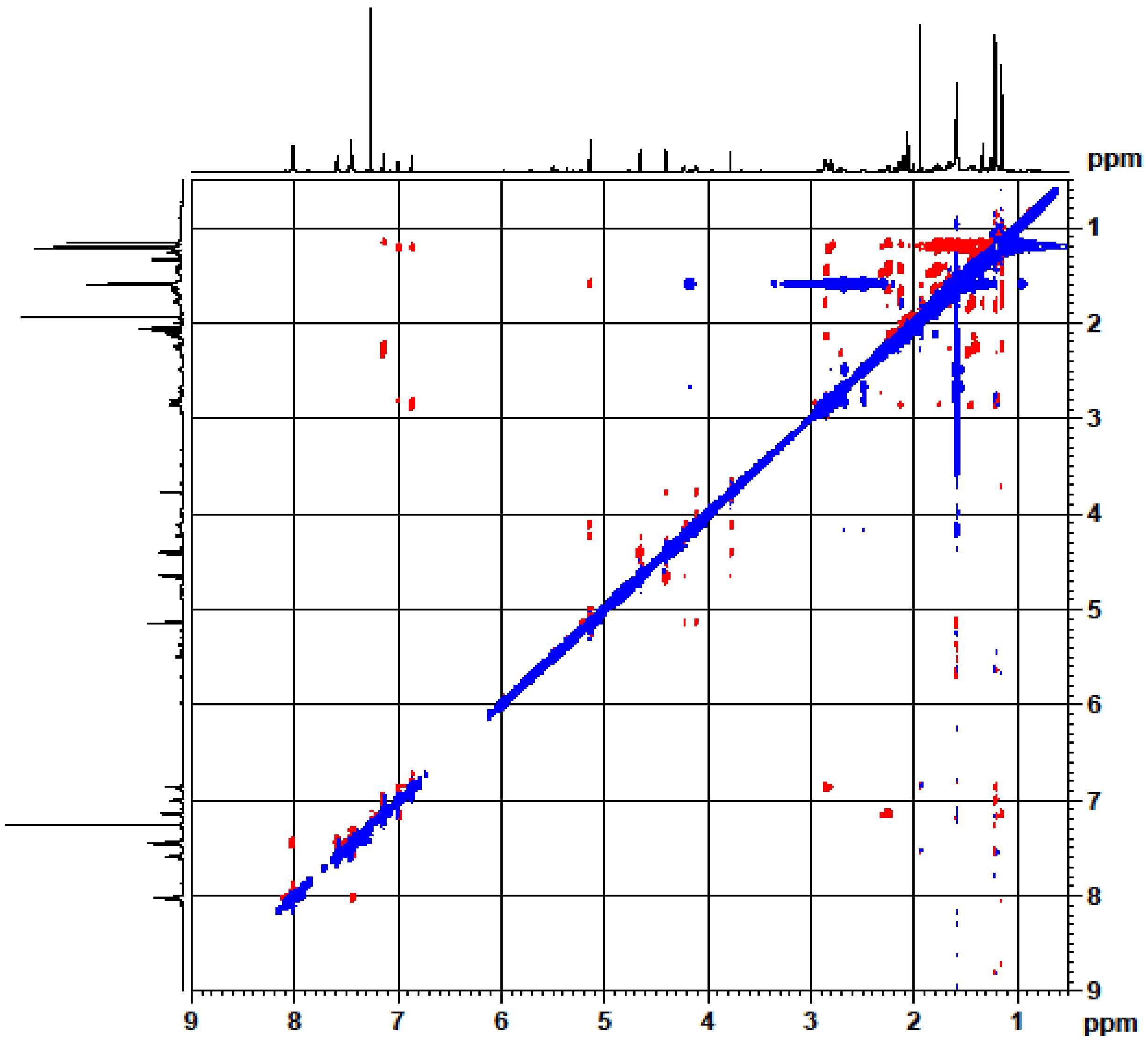

Figure S116. NOESY spectrum of **11** in $CDCl_3$

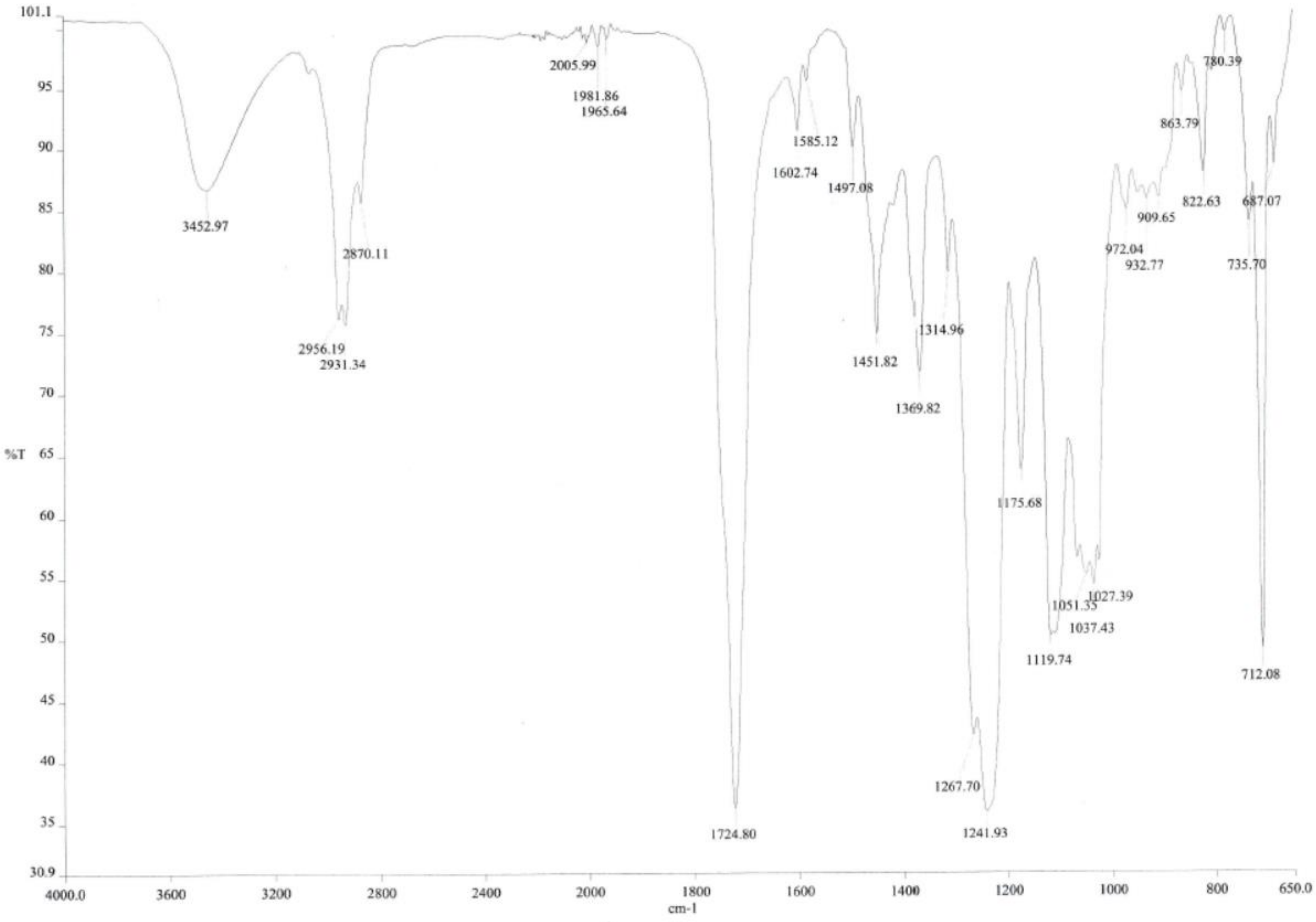

Figure S117. IR spectrum of compound **11**

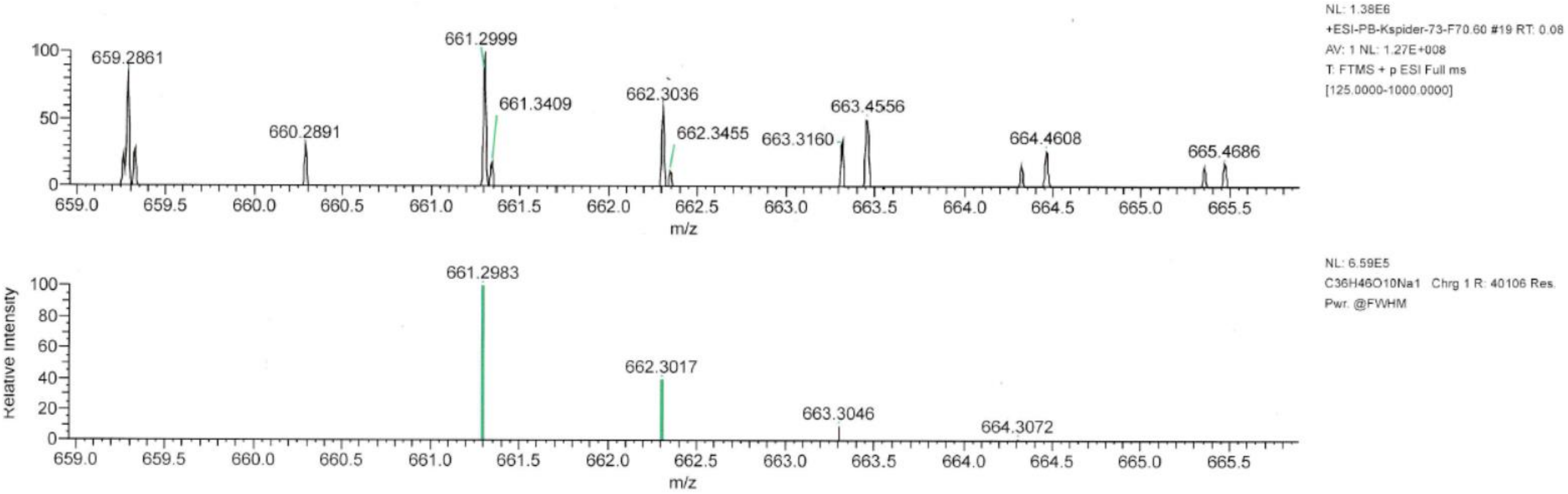

Peak Mass	Display Formula	Delta [ppm]	Theo. mass	MSMS Matched Fragments
661.2999	$C_{38}H_{45}O_{10}$	-1.30	661.30072	(Collection)
661.2999	$C_{36}H_{46}O_{10}{}^{23}Na$ [M+Na]$^+$	2.34	661.29832	(Collection)
661.2999	$C_{11}H_{58}O_{28}{}^{23}Na$	-1.23	661.30068	(Collection)
661.2999	$C_{20}H_{53}O_{23}$	4.01	661.29721	(Collection)
661.2999	$C_{13}H_{57}O_{28}$	-4.87	661.30309	(Collection)

Figure S118. HRESIMS spectrum of compound **11**

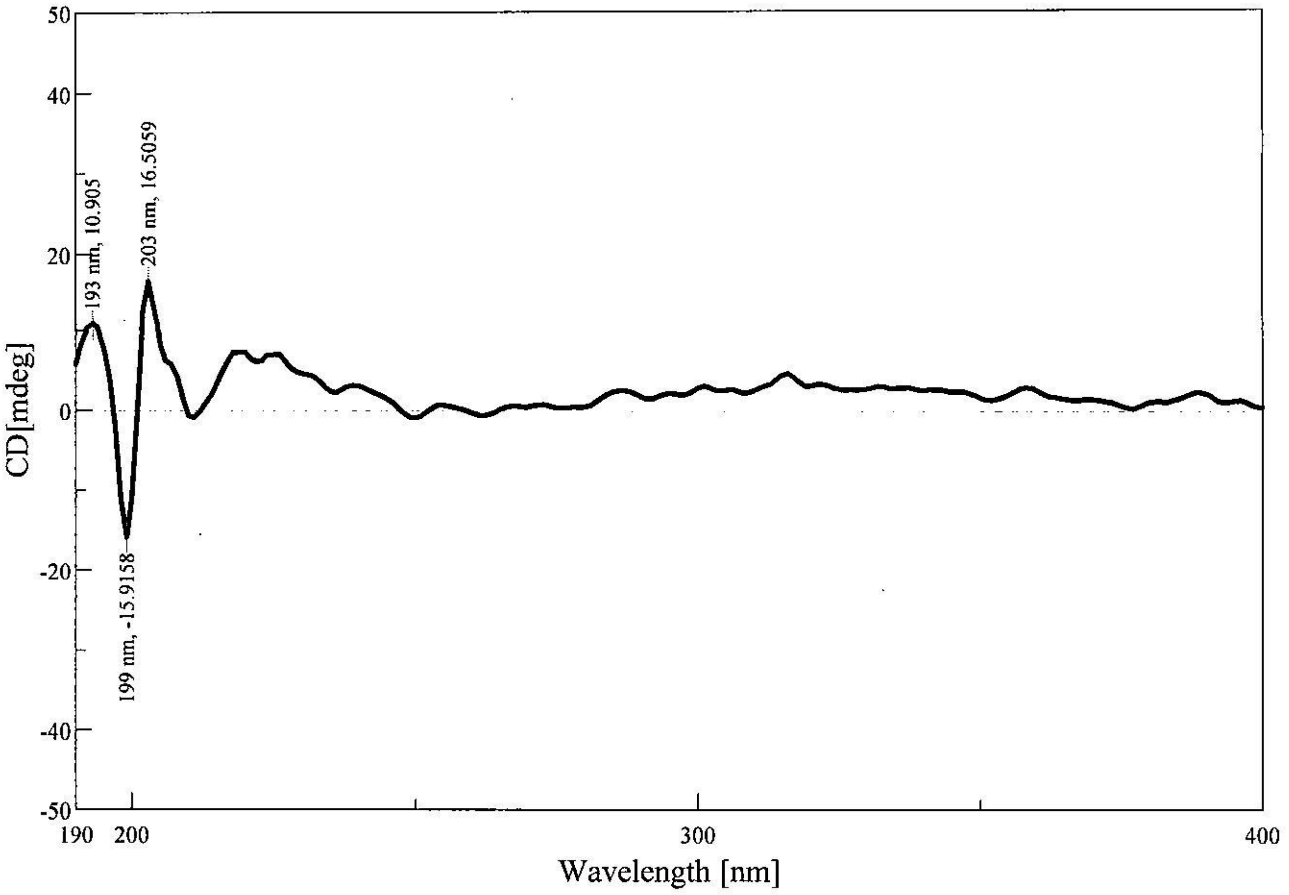

Figure S119. CD spectrum of compound **11**

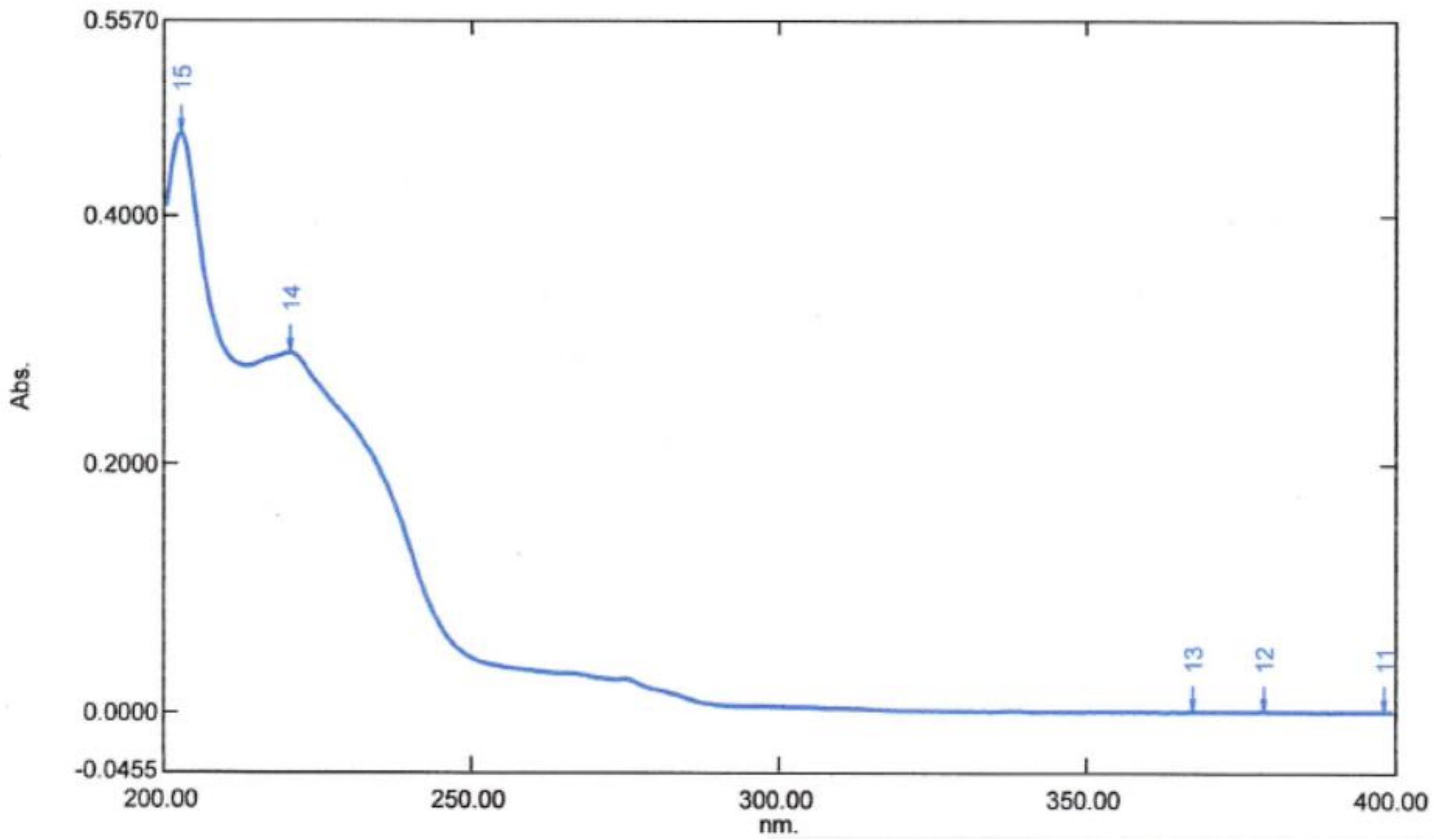

Figure S120. UV spectrum of compound **11**

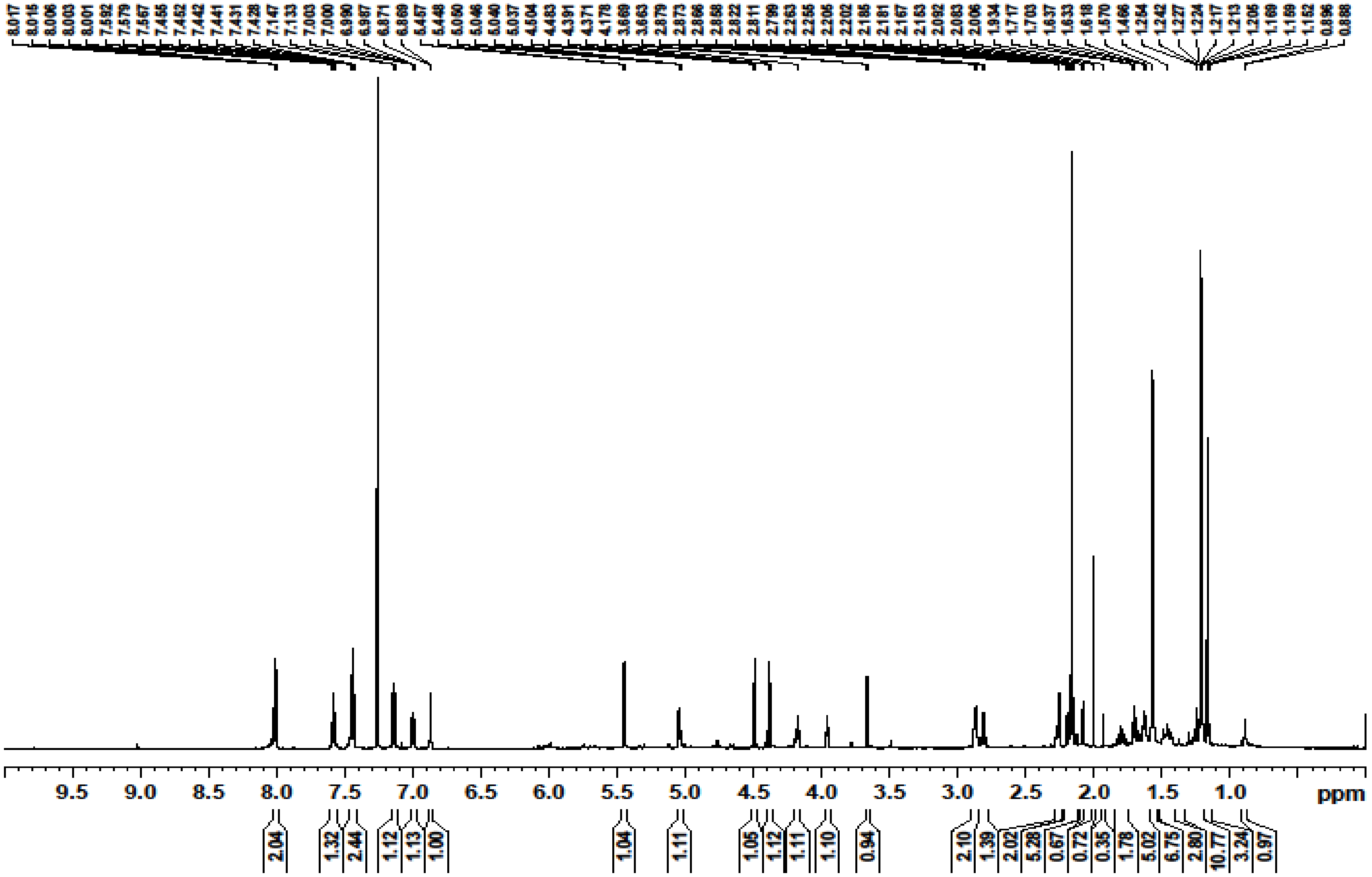

Figure S121. ^{1}H NMR (600 MHz) spectrum of compound **12** in $CDCl_3$

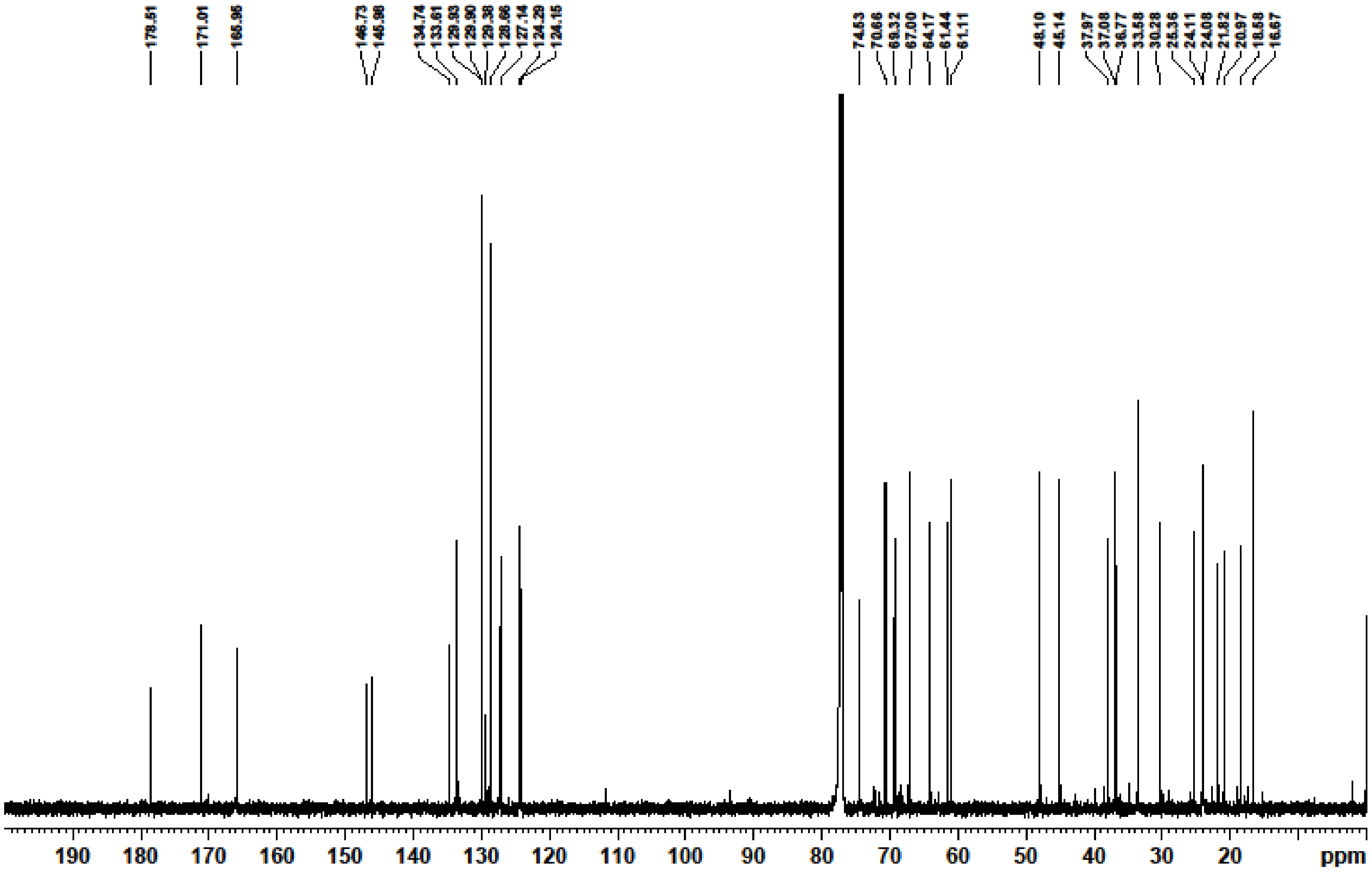

Figure S122. ^{13}C NMR (150 MHz) spectrum of compound **12** in $CDCl_3$

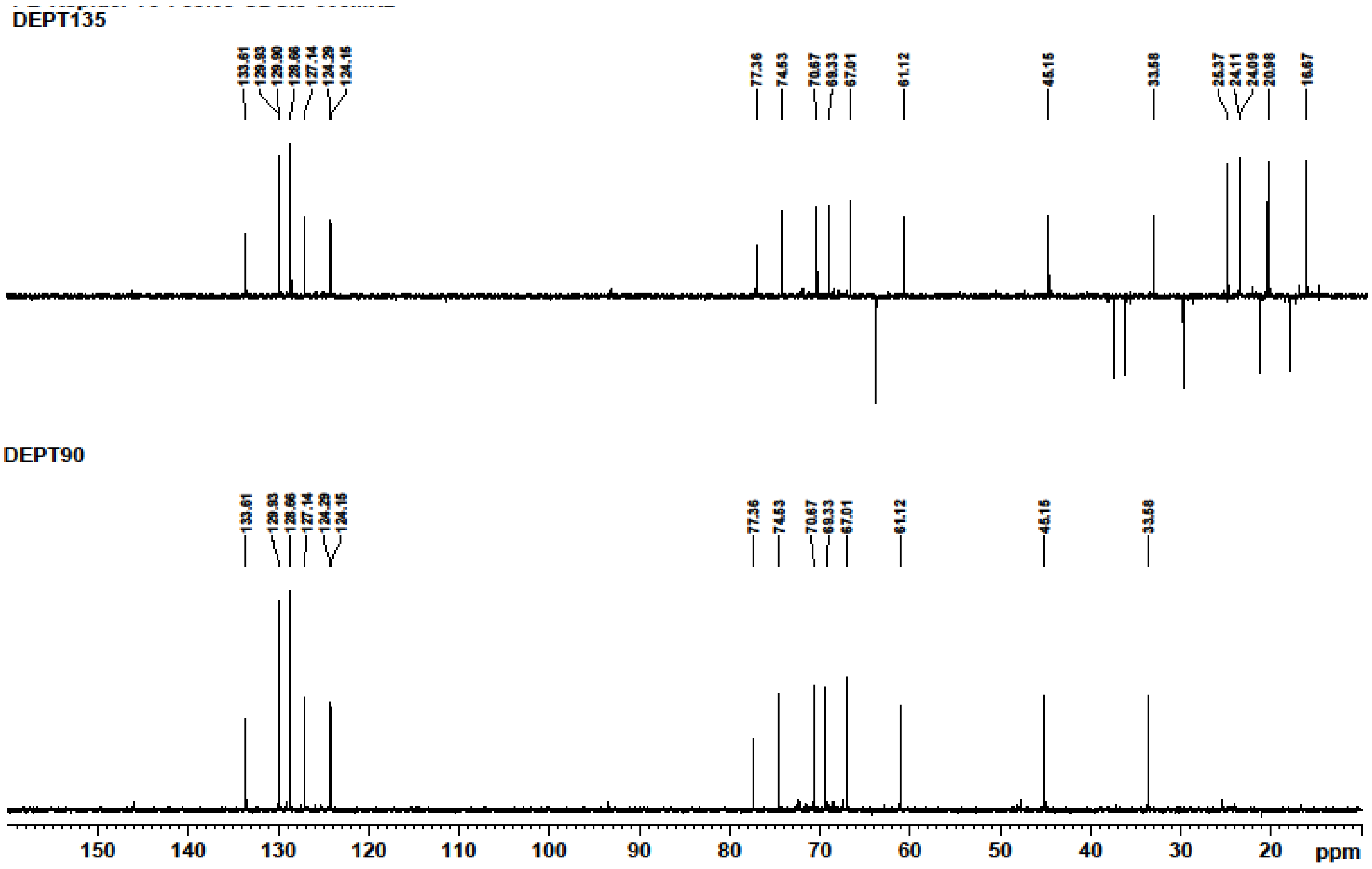

Figure S123. DEPT 135 & 90 NMR spectrum of compound **12** in $CDCl_3$

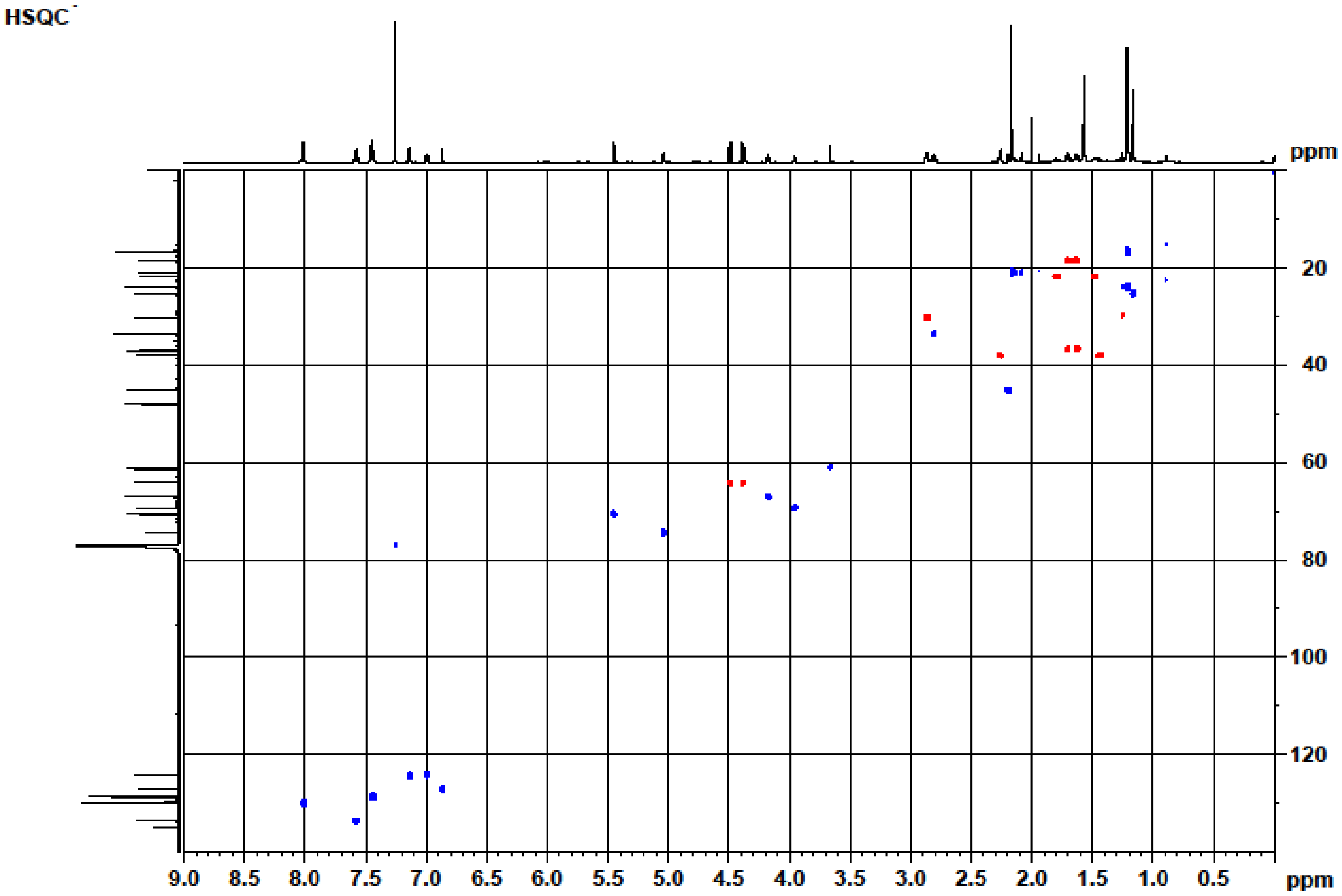

Figure S124. HSQC spectrum of compound **12** in $CDCl_3$

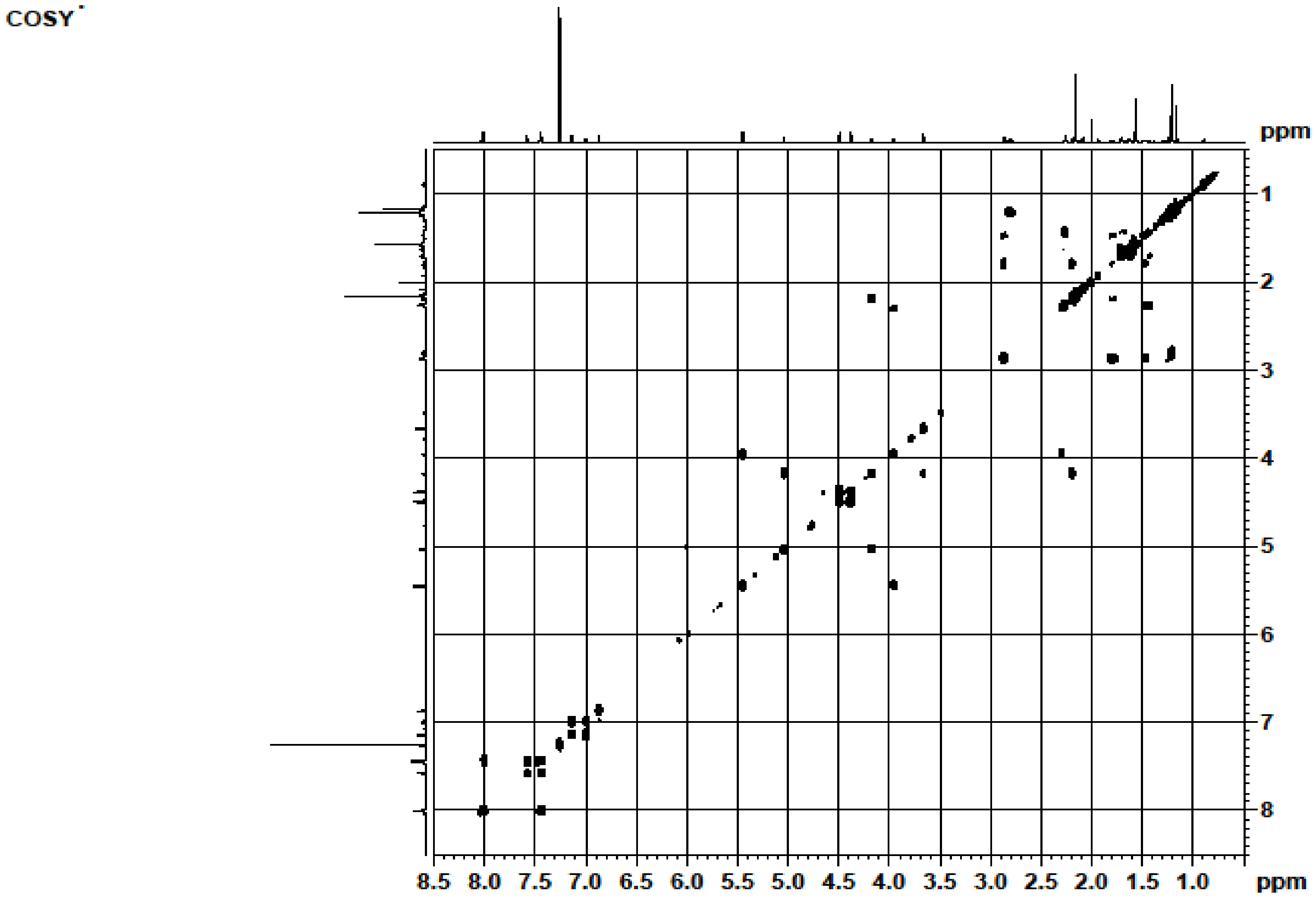

Figure S125. COSY spectrum of compound **12** in $CDCl_3$

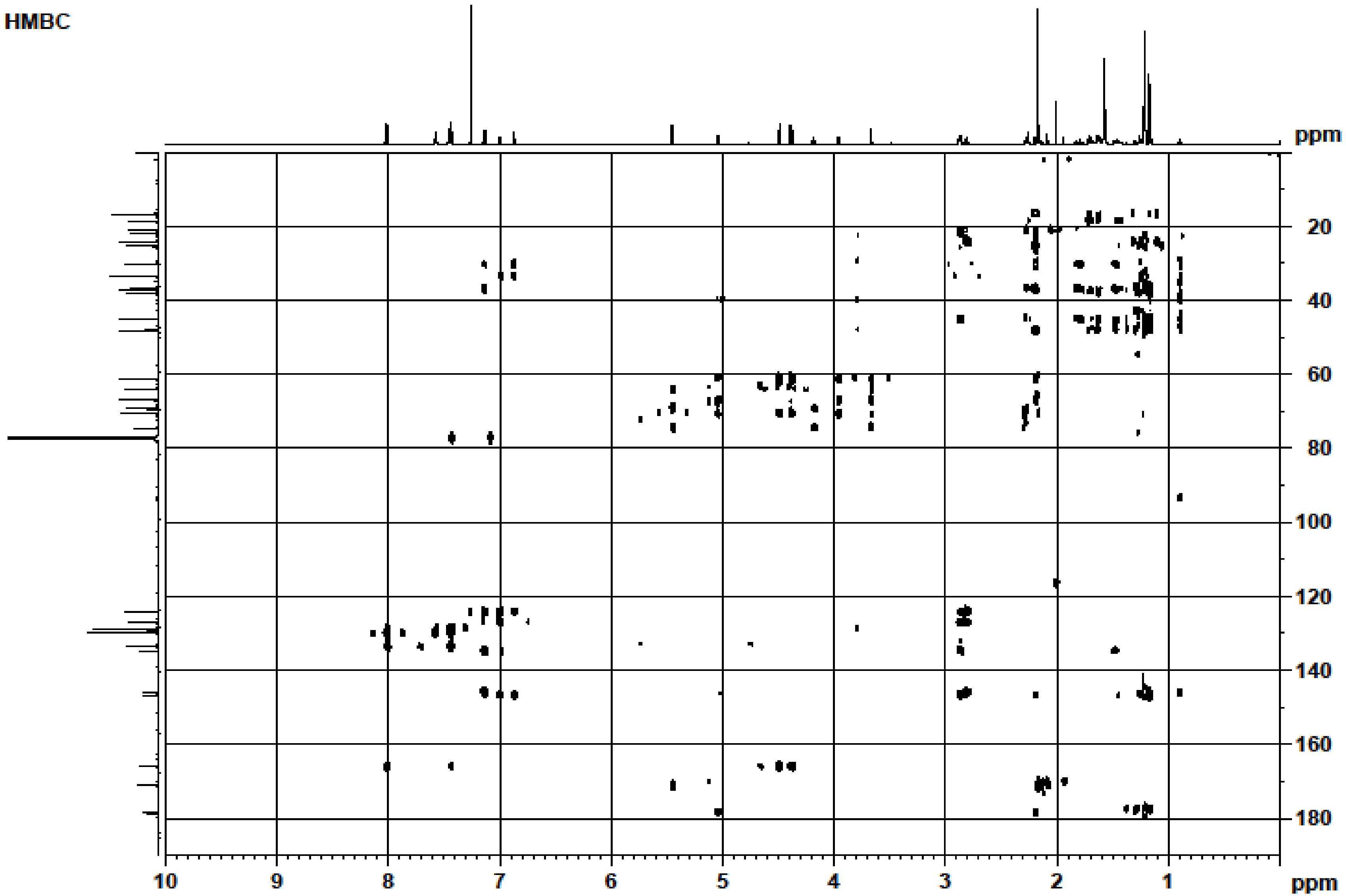

Figure S126. HMBC spectrum of compound **12** in $CDCl_3$

NOESY

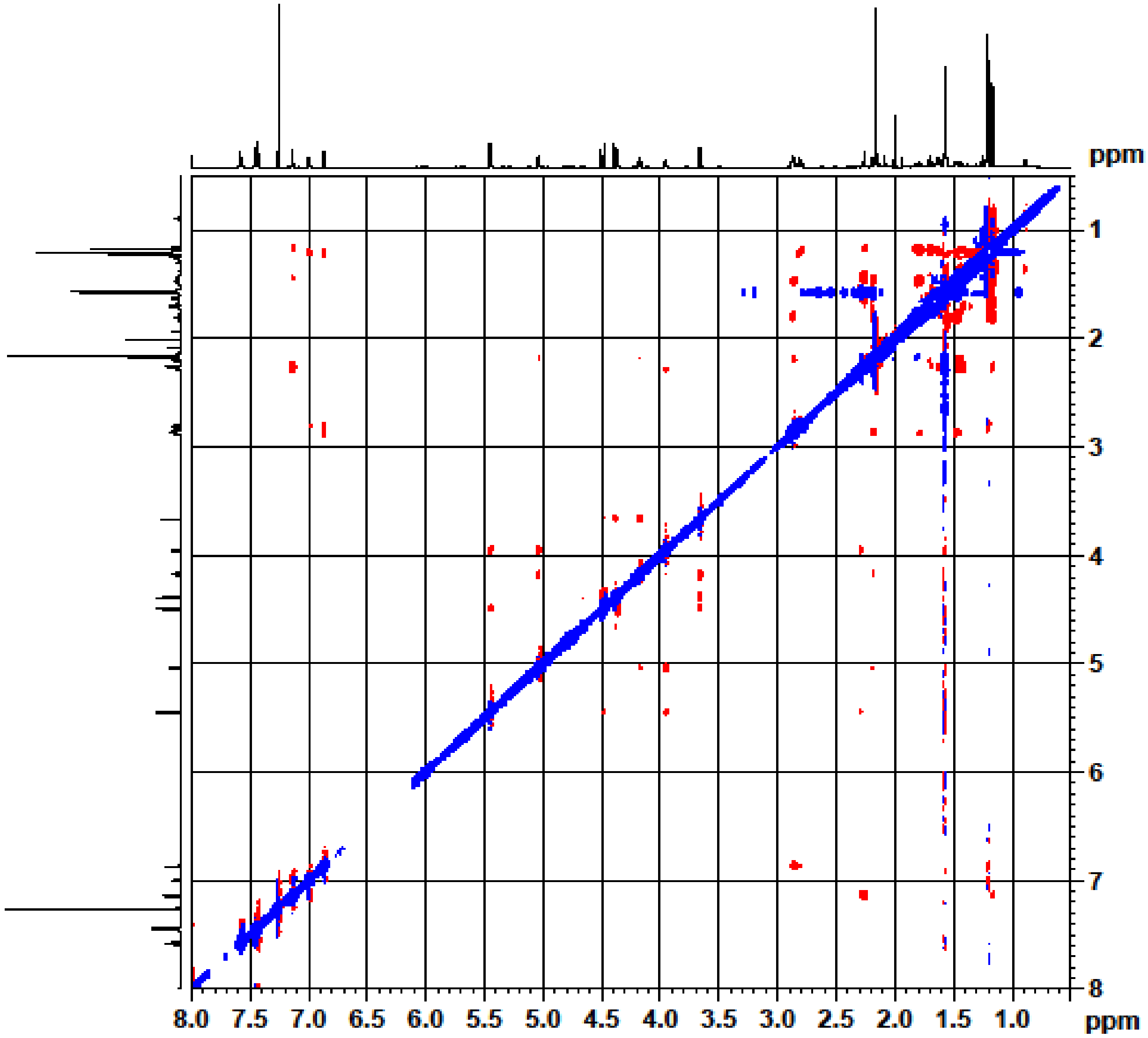

Figure S127. NOESY spectrum of **12** in $CDCl_3$

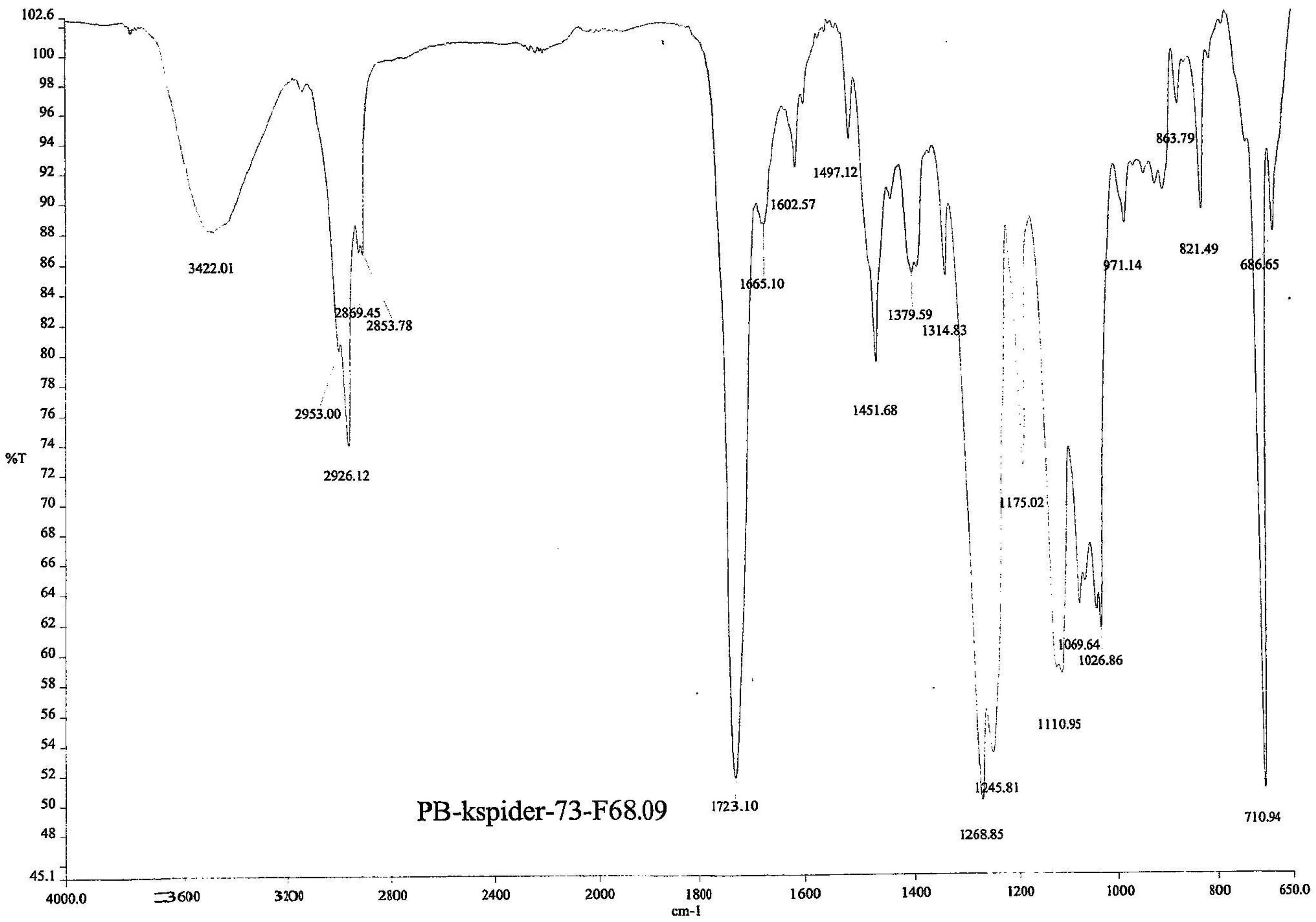

Figure S128. IR spectrum of compound **12**

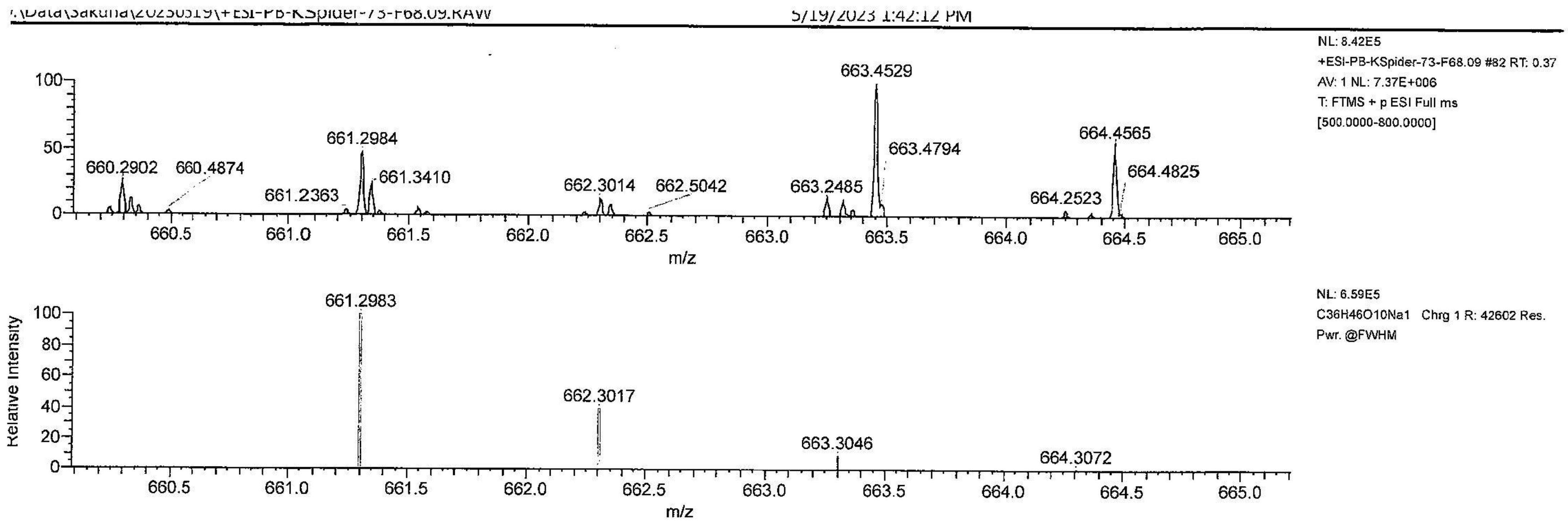

Peak Mass	Display Formula	Delta [ppm]	Theo. mass	MSMS Matched Fragments
661.2984	$C_{36}H_{46}O_{10}{}^{23}Na$ [M+ [illegible]	0.12	661.29832	(Collection)
661.2984	$C_{20}H_{53}O_{23}$	1.79	661.29721	(Collection)
661.2984	$C_{38}H_{45}O_{10}$	-3.51	661.30072	(Collection)
661.2984	$C_{11}H_{58}O_{28}{}^{23}Na$	-3.45	661.30068	(Collection)

Figure S129. HRESIMS spectrum of compound **12**

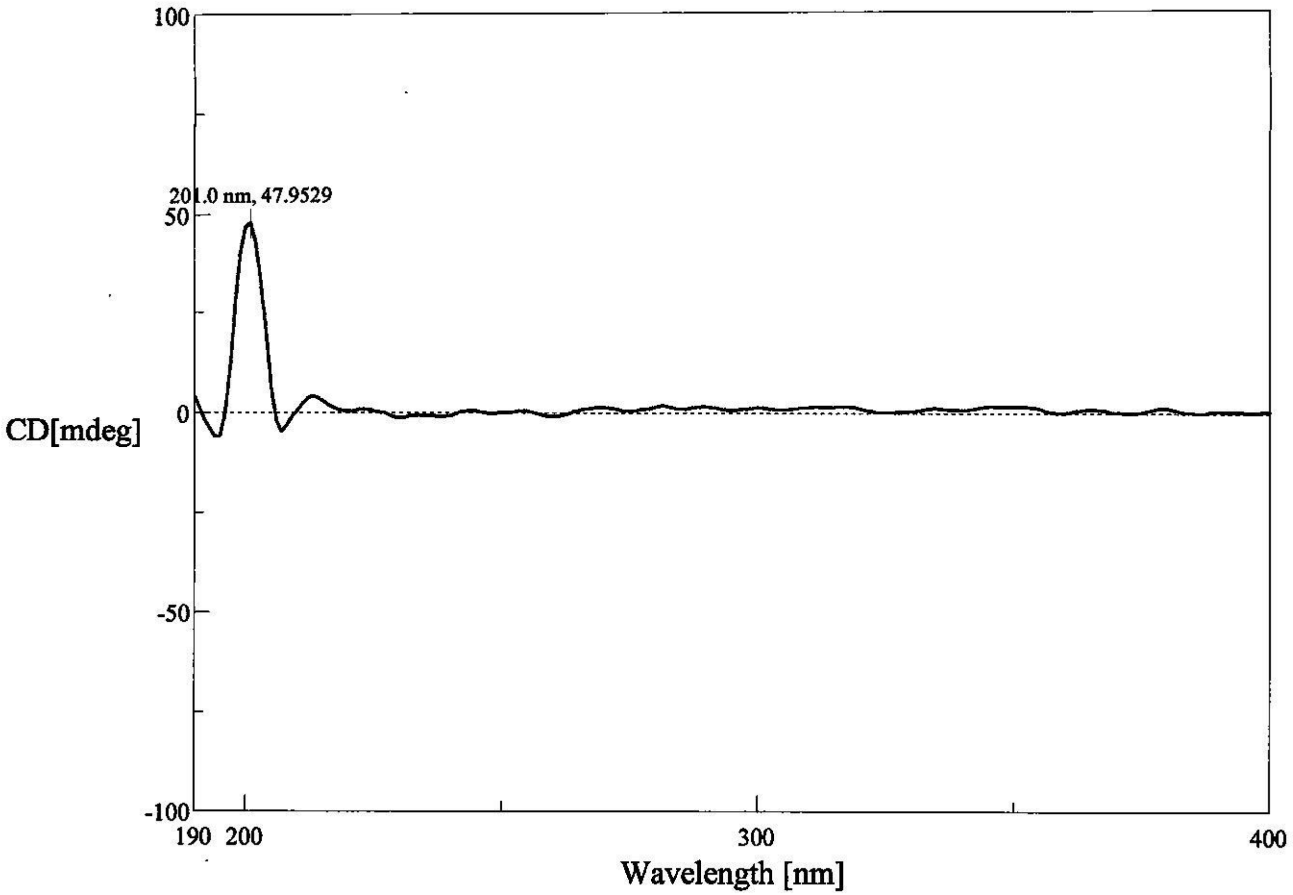

Figure S130. CD spectrum of compound **12**

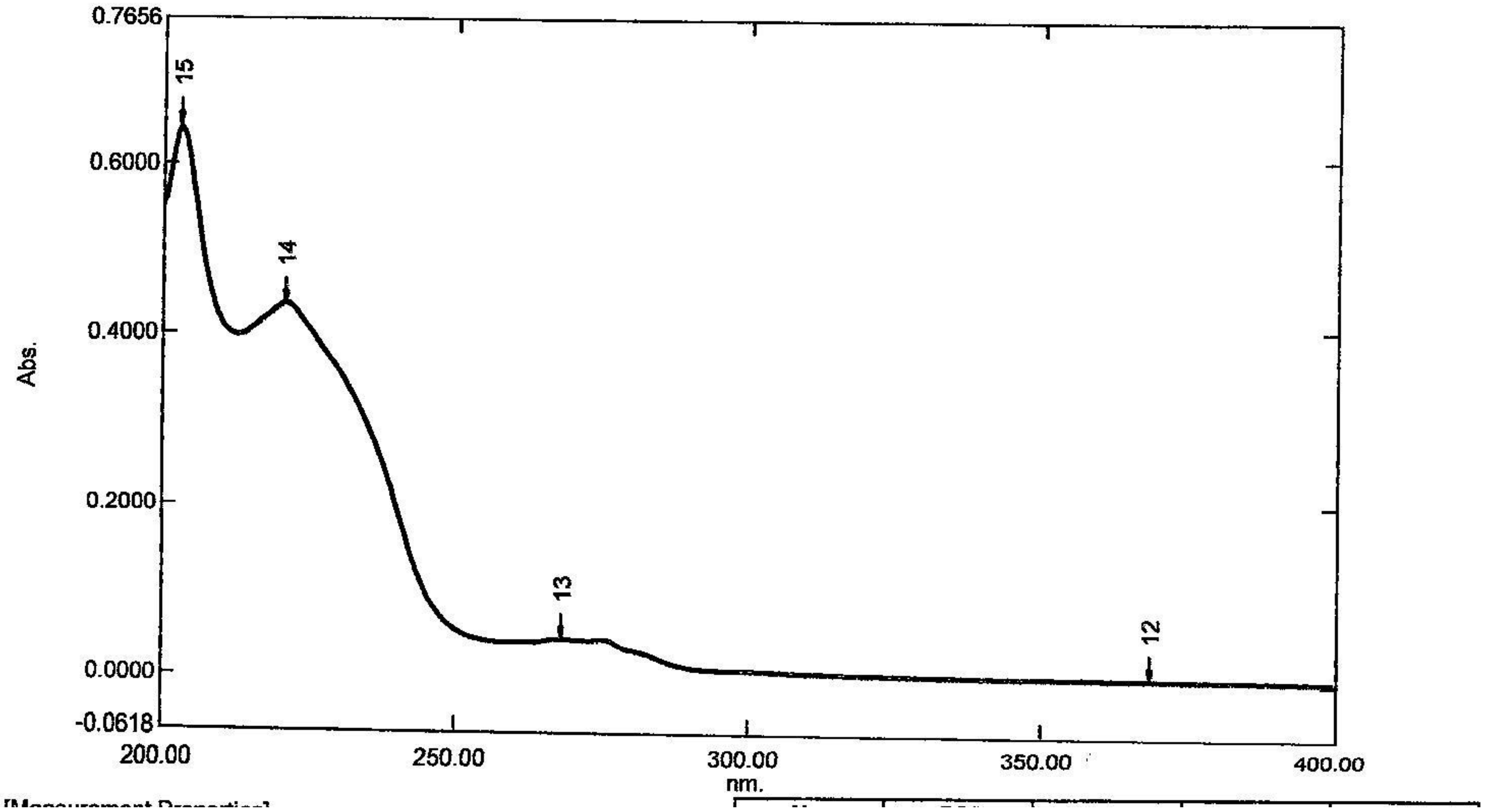

Figure S131. UV spectrum of compound **12**

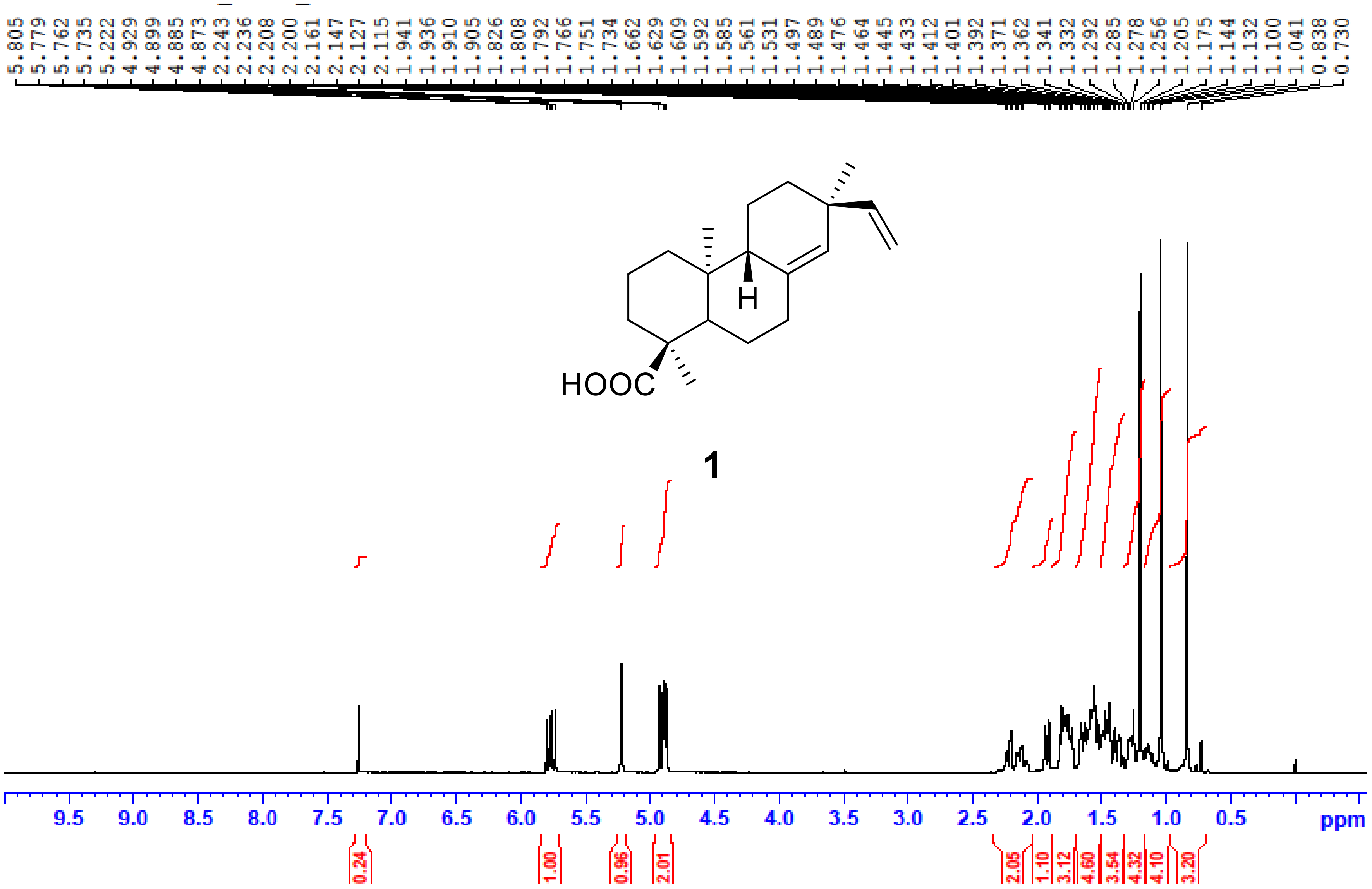

Figure S132. ^{1}H NMR (600 MHz) spectrum of compound **13** in $CDCl_3$

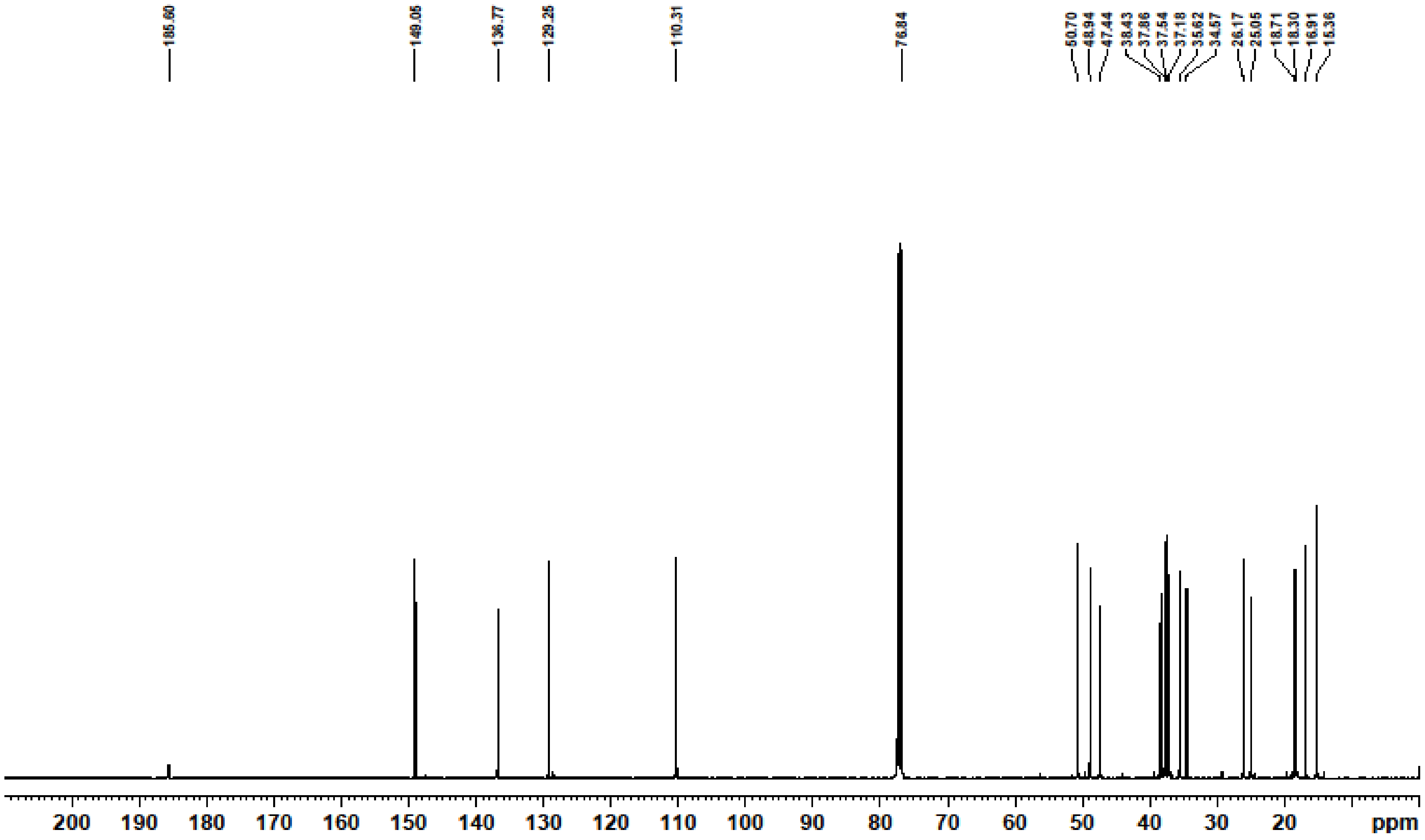

Figure S133. ^{13}C NMR (150 MHz) spectrum of compound **13** in $CDCl_3$

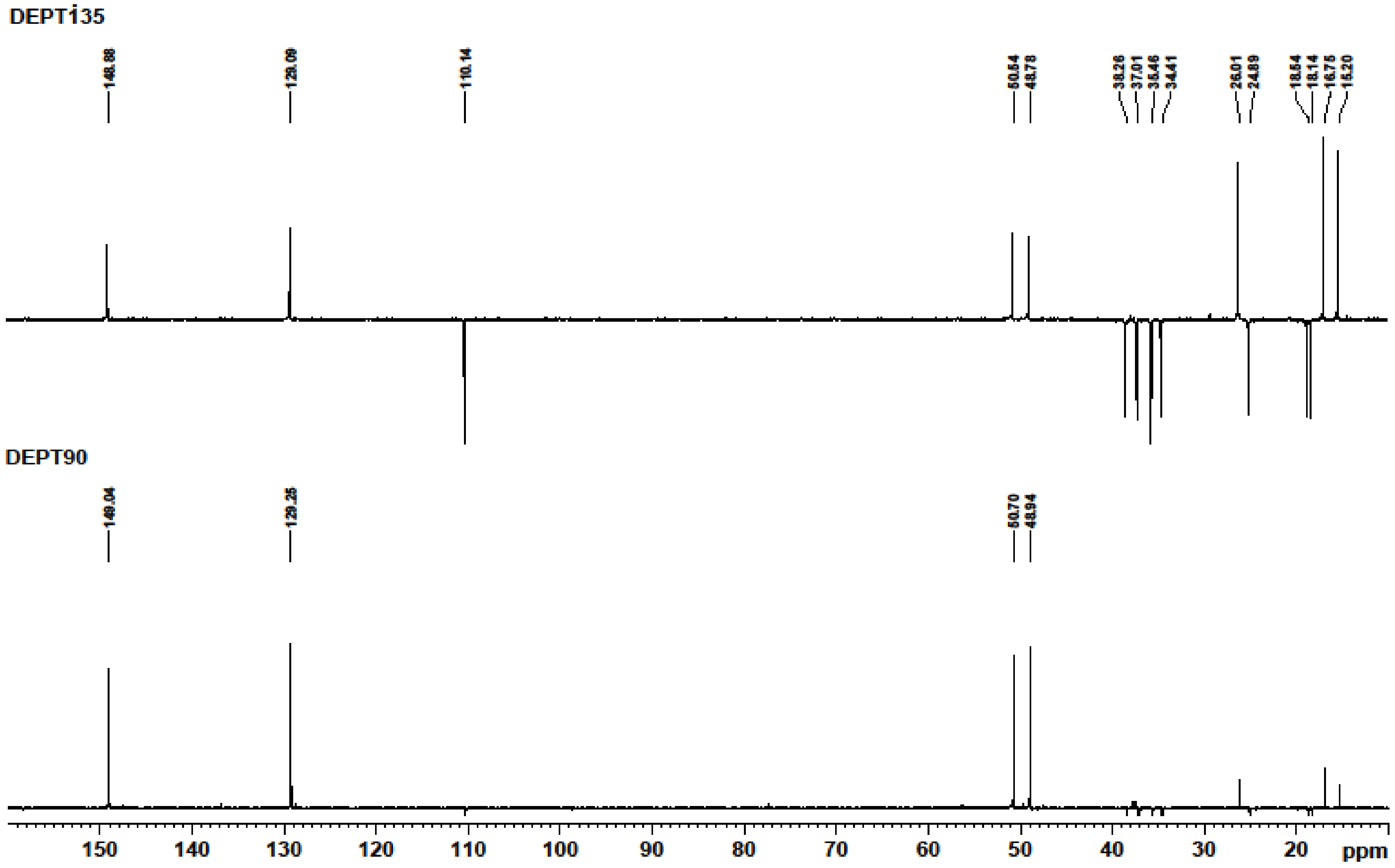

Figure S134. DEPT 135 & 90 NMR spectrum of compound **13** in $CDCl_3$

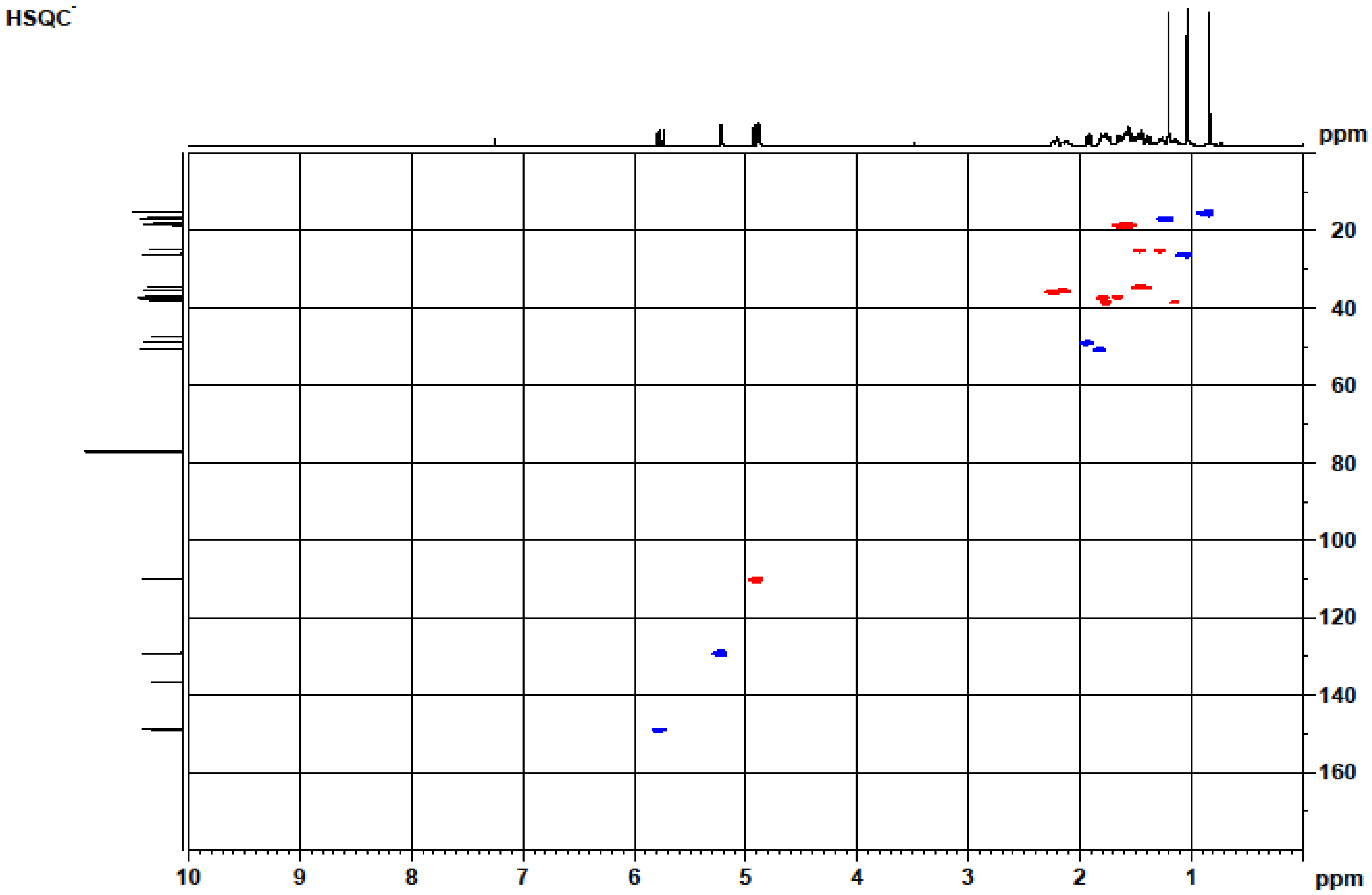

Figure S135. HSQC spectrum of compound **13** in $CDCl_3$

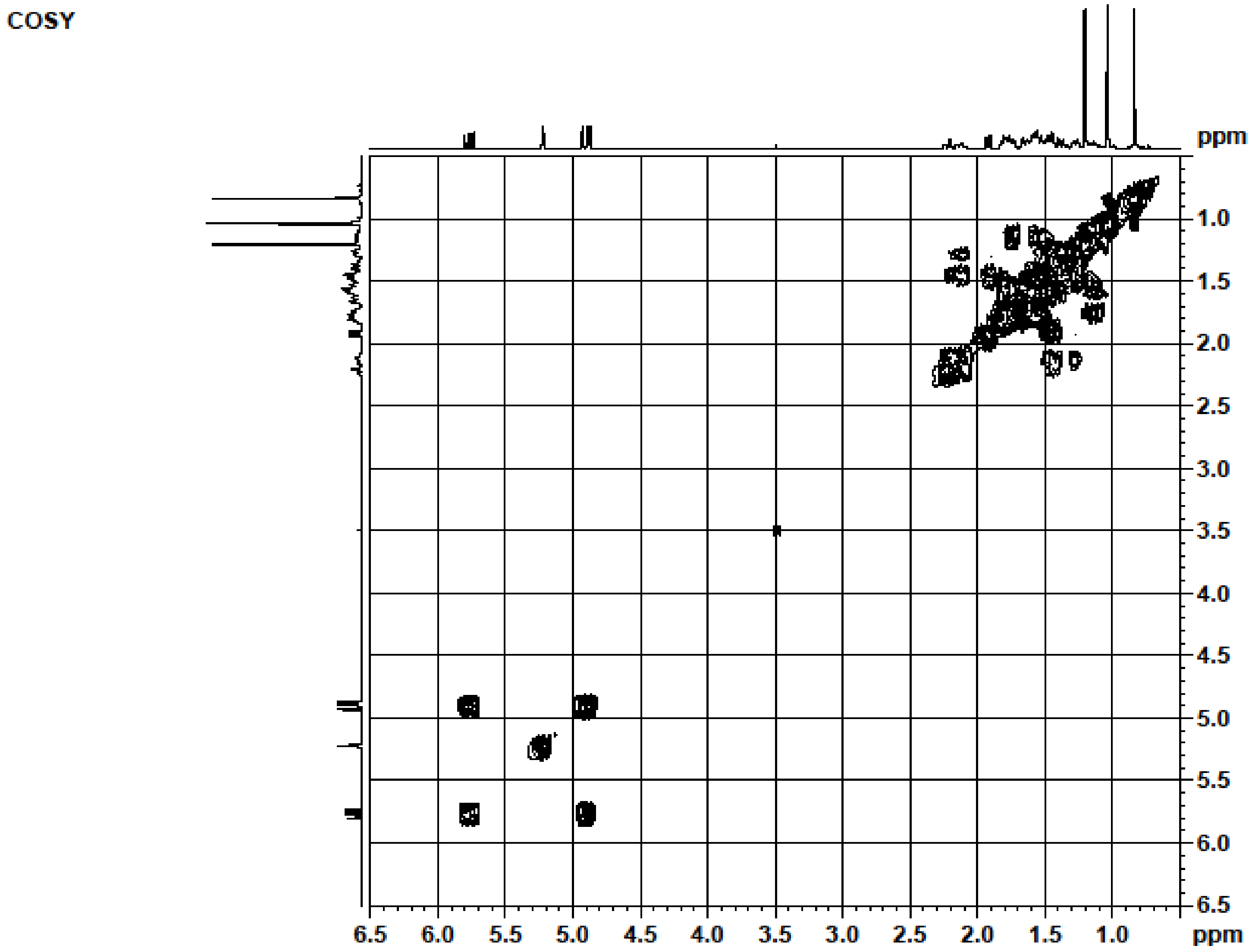

Figure S136. COSY spectrum of compound **13** in $CDCl_3$

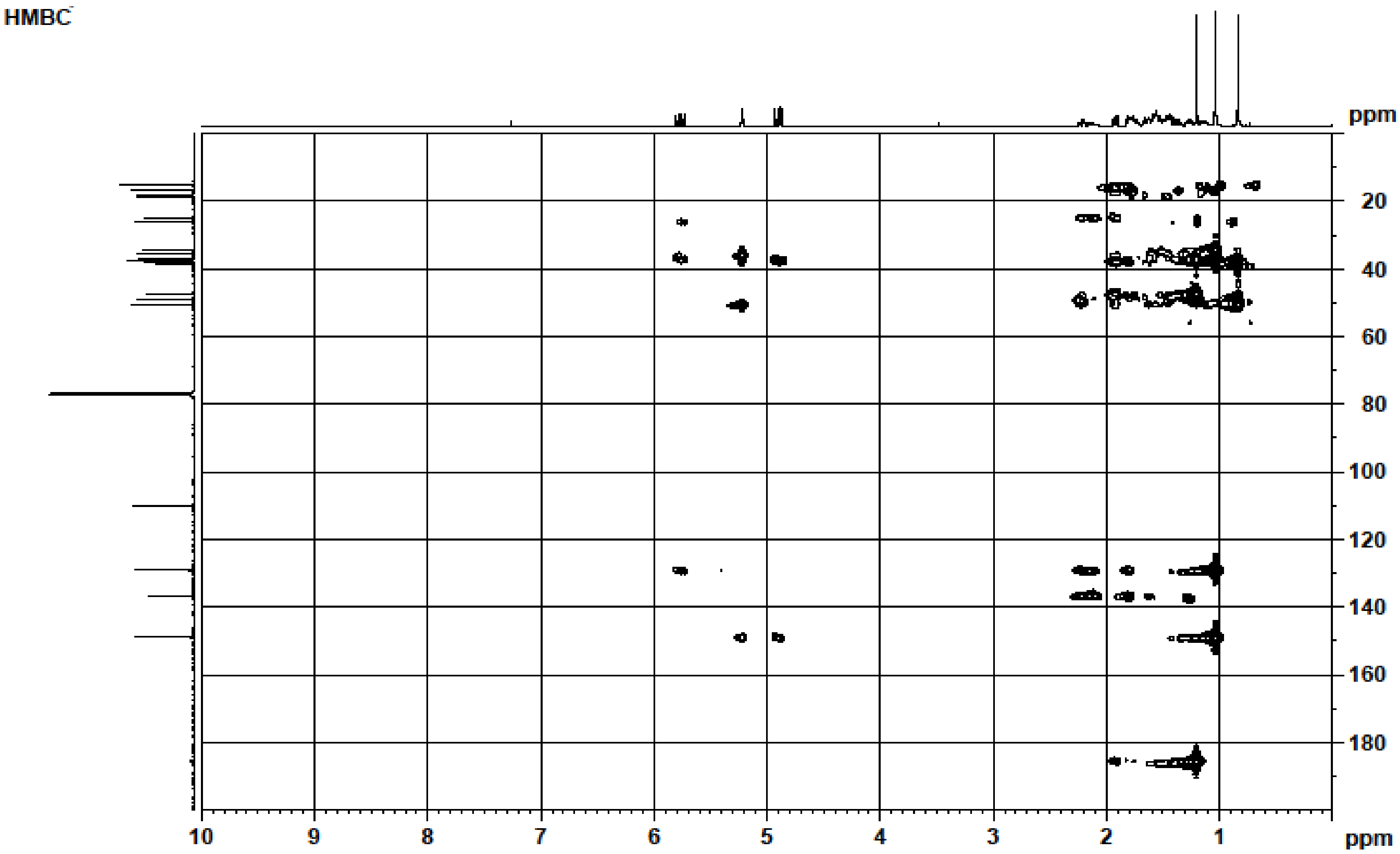

Figure S137. HMBC spectrum of compound **13** in $CDCl_3$

NOESY

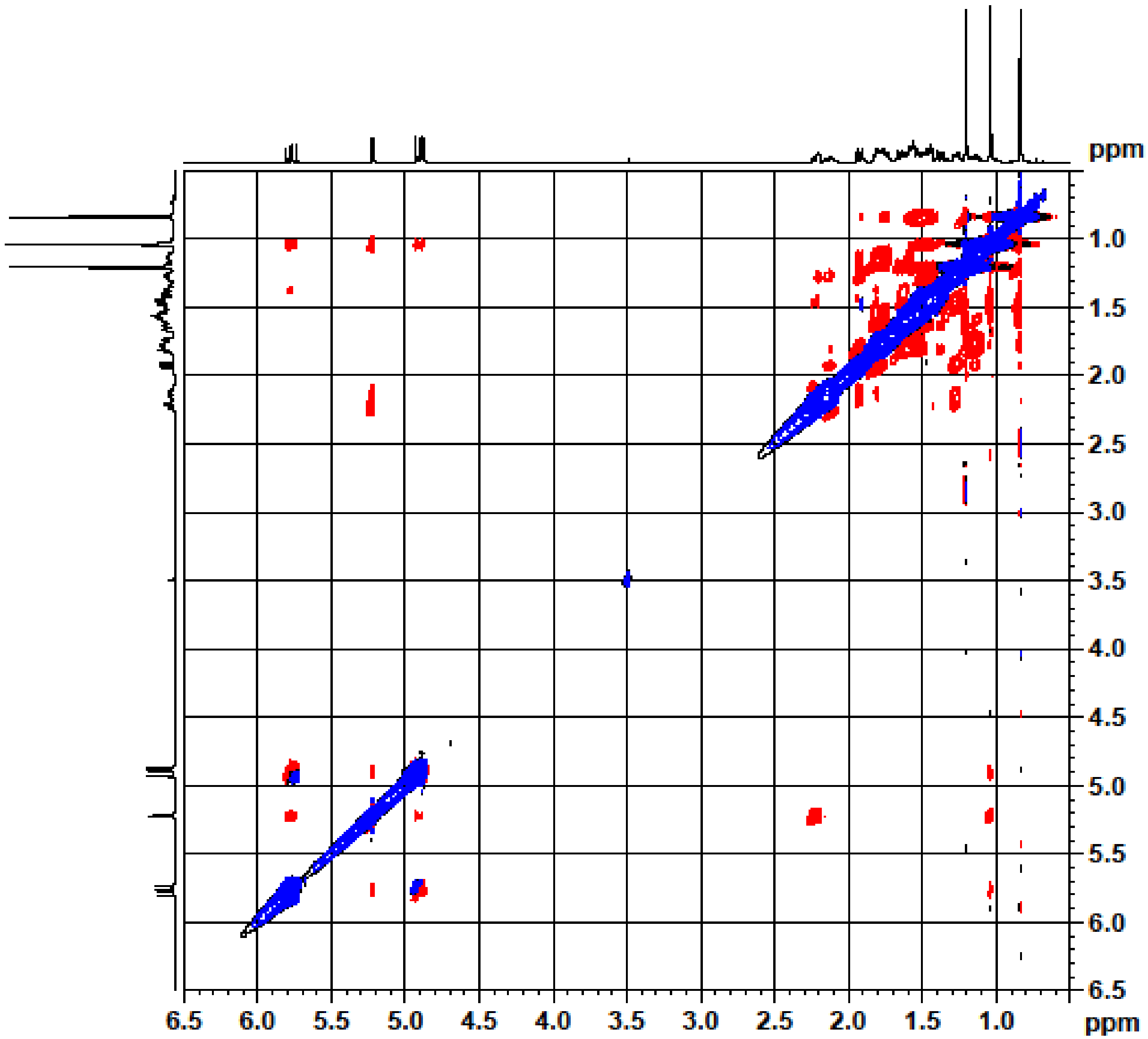

Figure S138. NOESY spectrum of **13** in $CDCl_3$

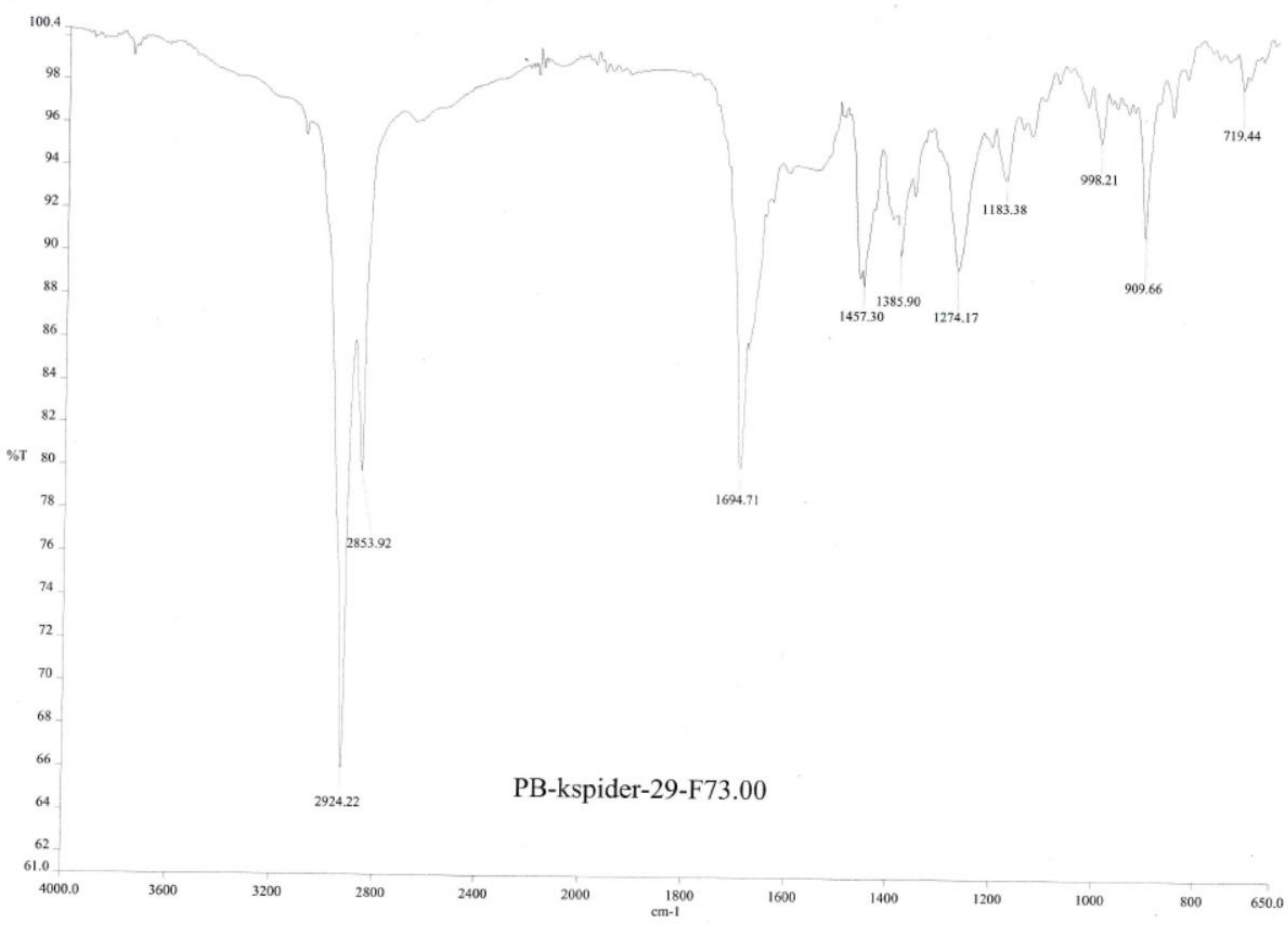

Figure S139. IR spectrum of compound **13**

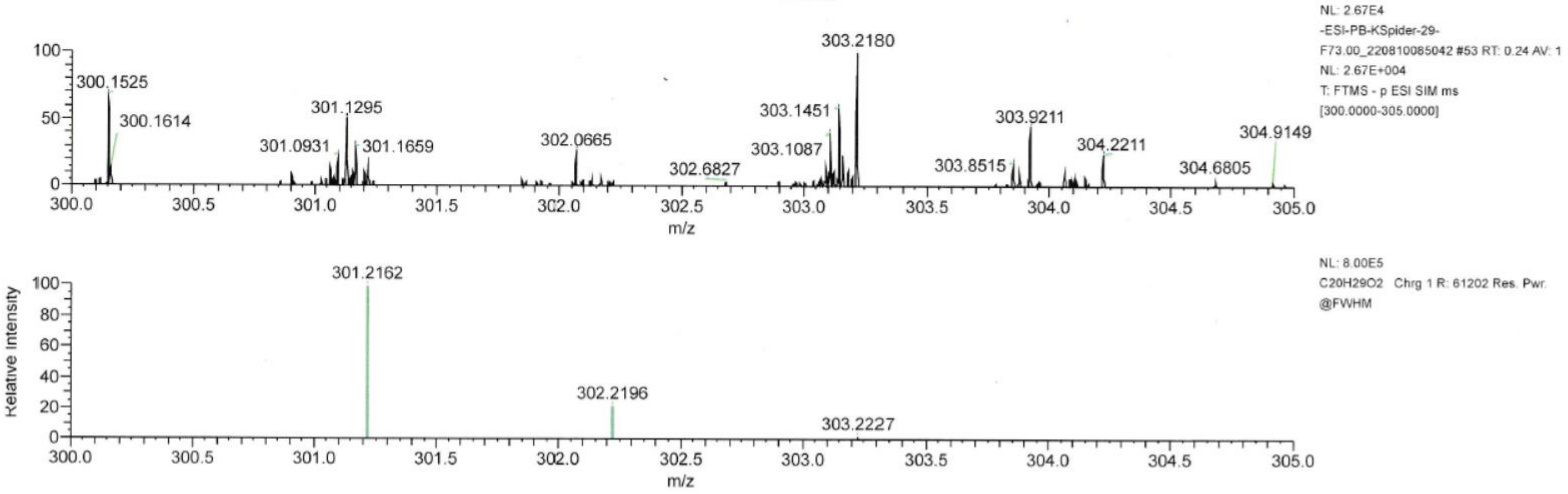

Peak Mass	Display Formula	Delta [ppm]	Theo. mass	MSMS Matched Fragments
301.2173	$C_{20}H_{29}O_2$ [M-H]⁻	3.58	301.21621	(Collection)

Figure S140. HRESIMS spectrum of compound **13**

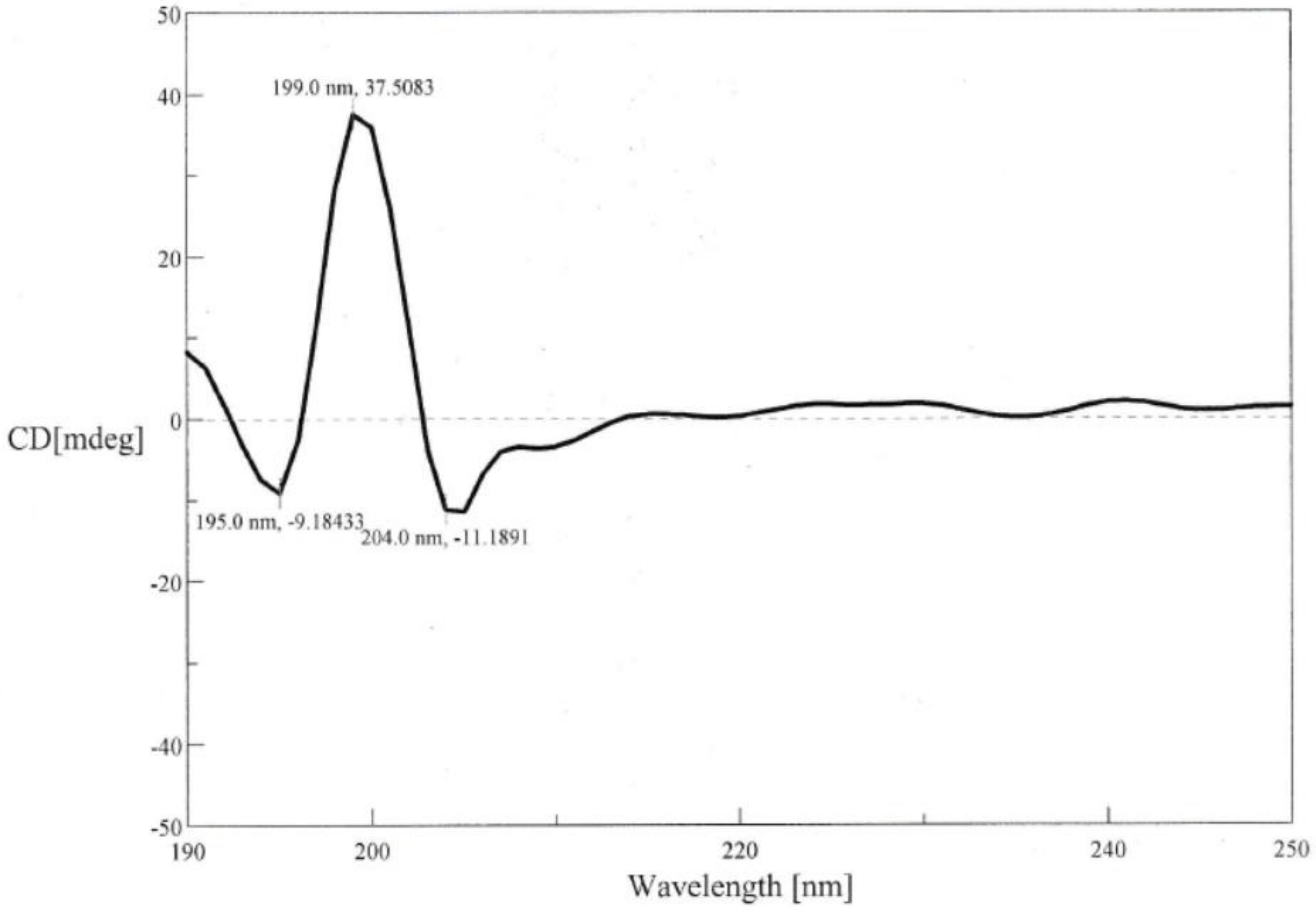

Figure S141. CD spectrum of compound **13**

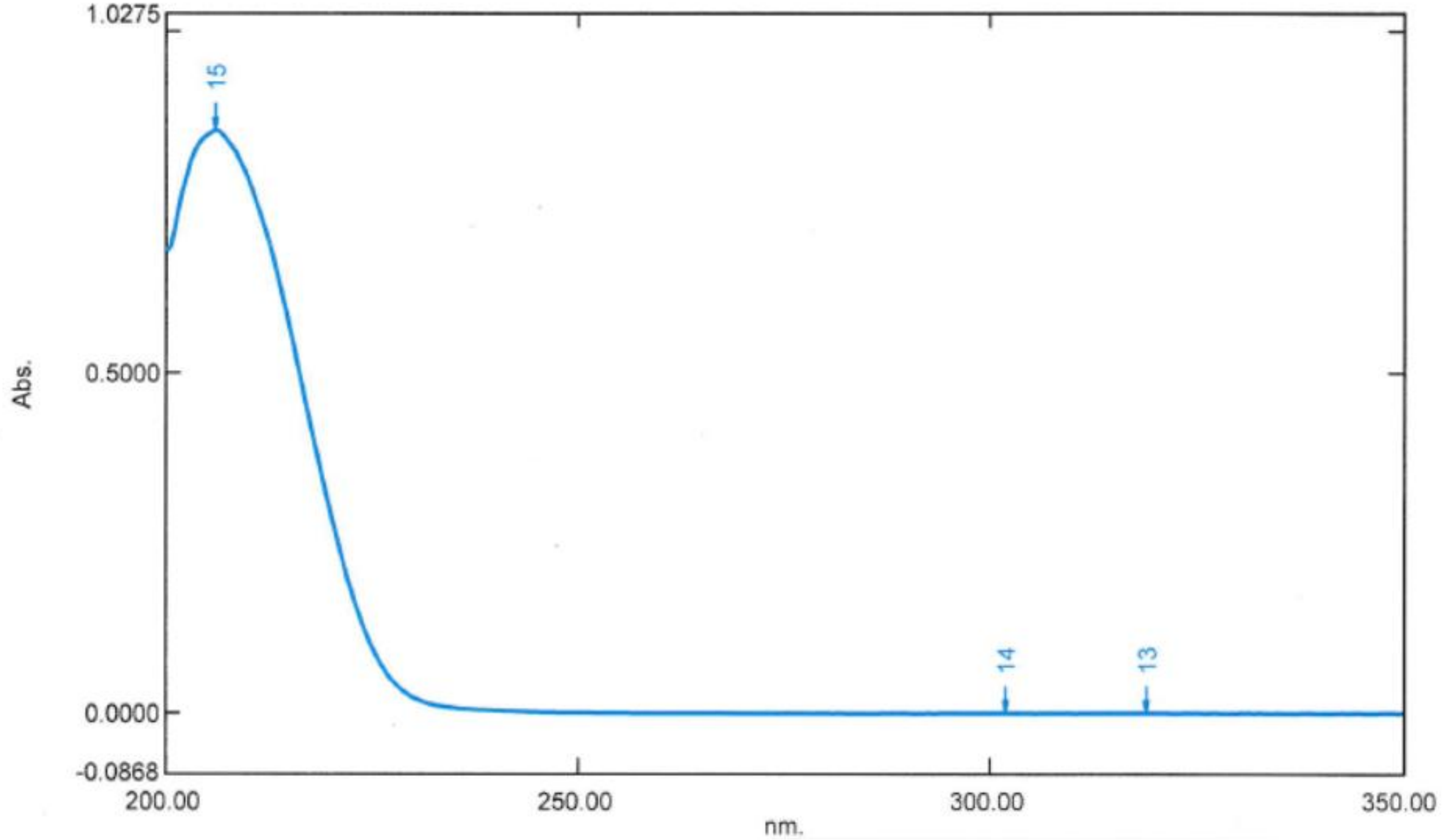

Figure S142. UV spectrum of compound **13**

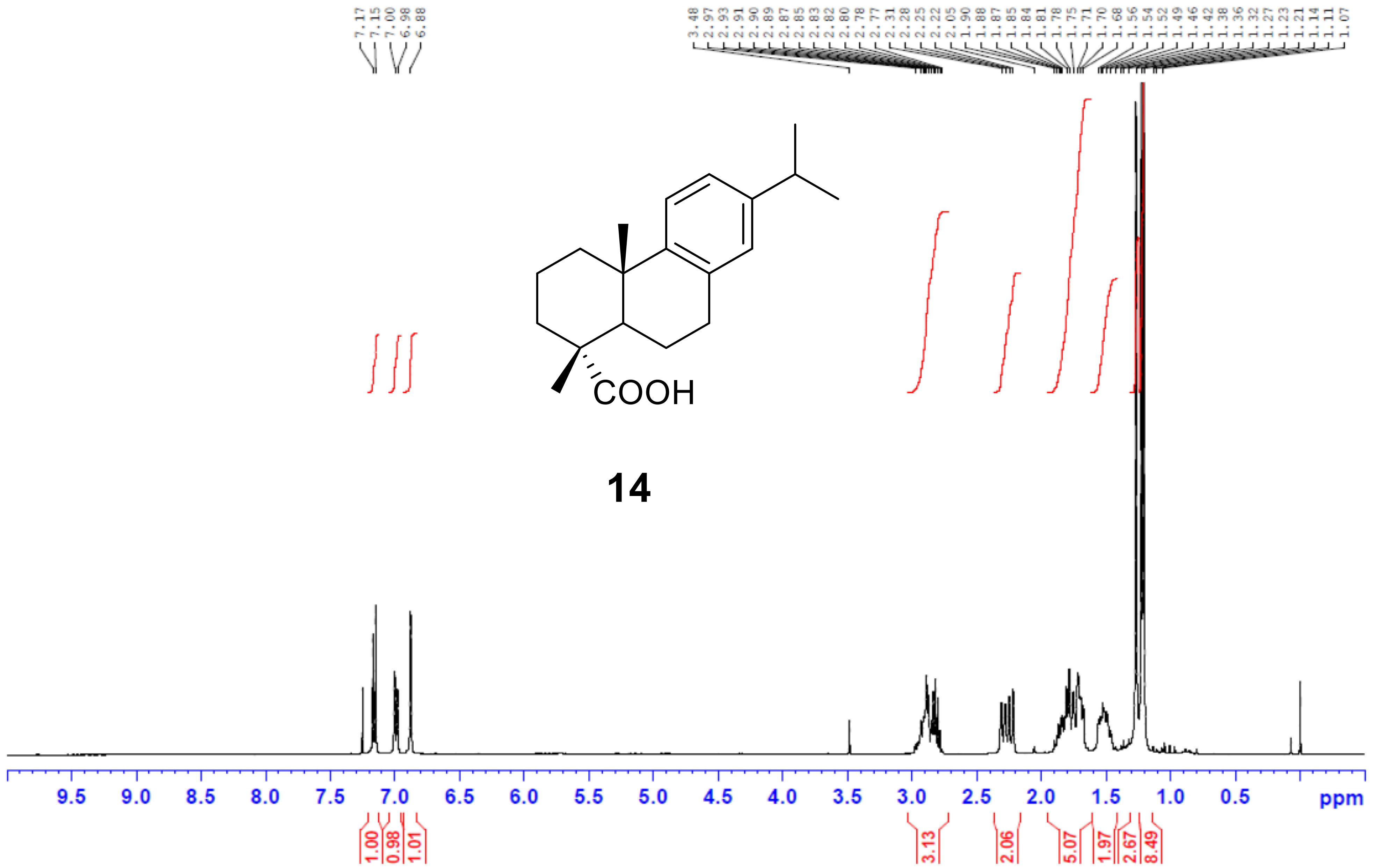

Figure S143. ^{1}H NMR (600 MHz) spectrum of compound **14** in $CDCl_3$

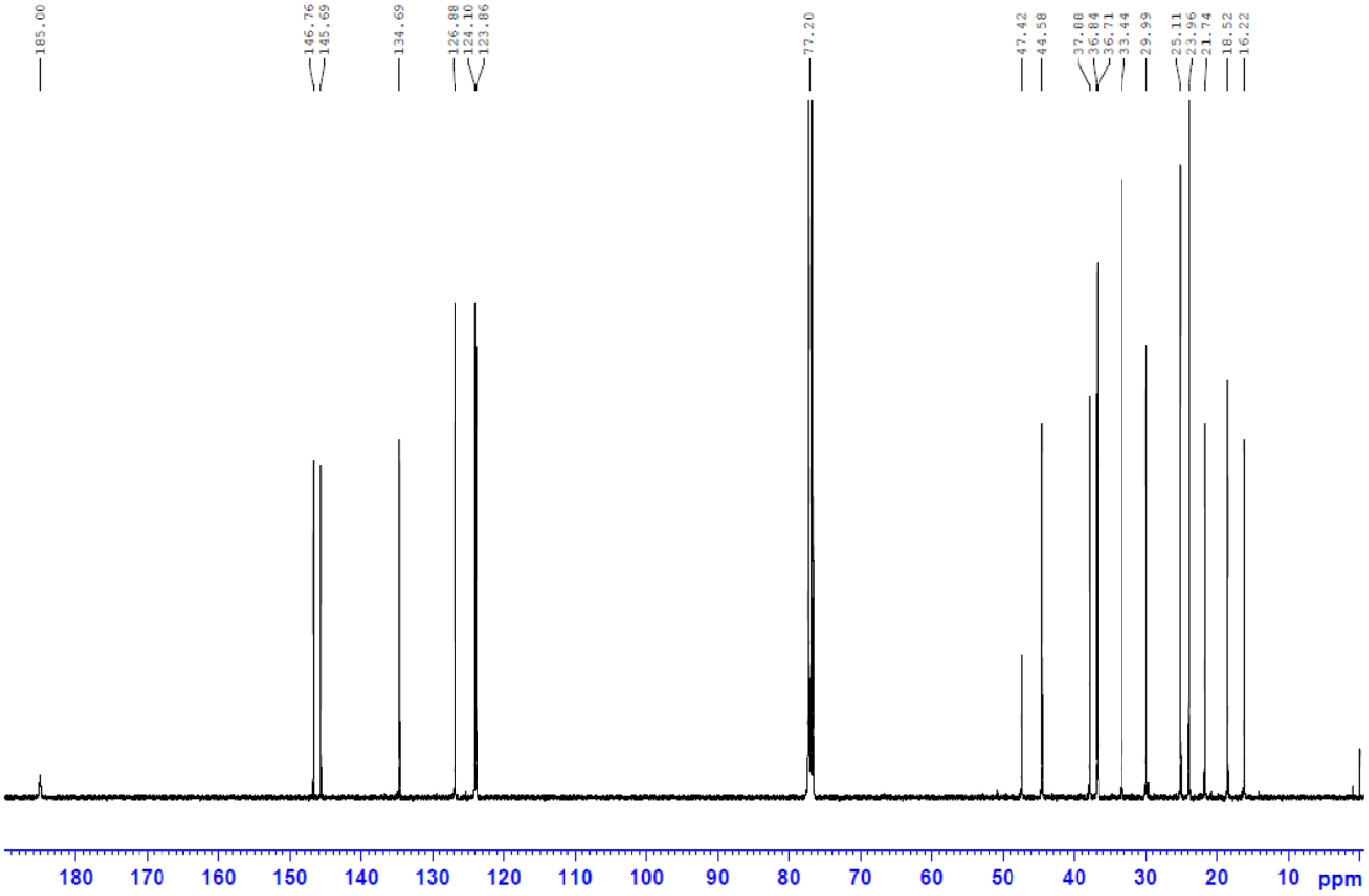

Figure S144. ^{13}C NMR (150 MHz) spectrum of compound **14** in $CDCl_3$

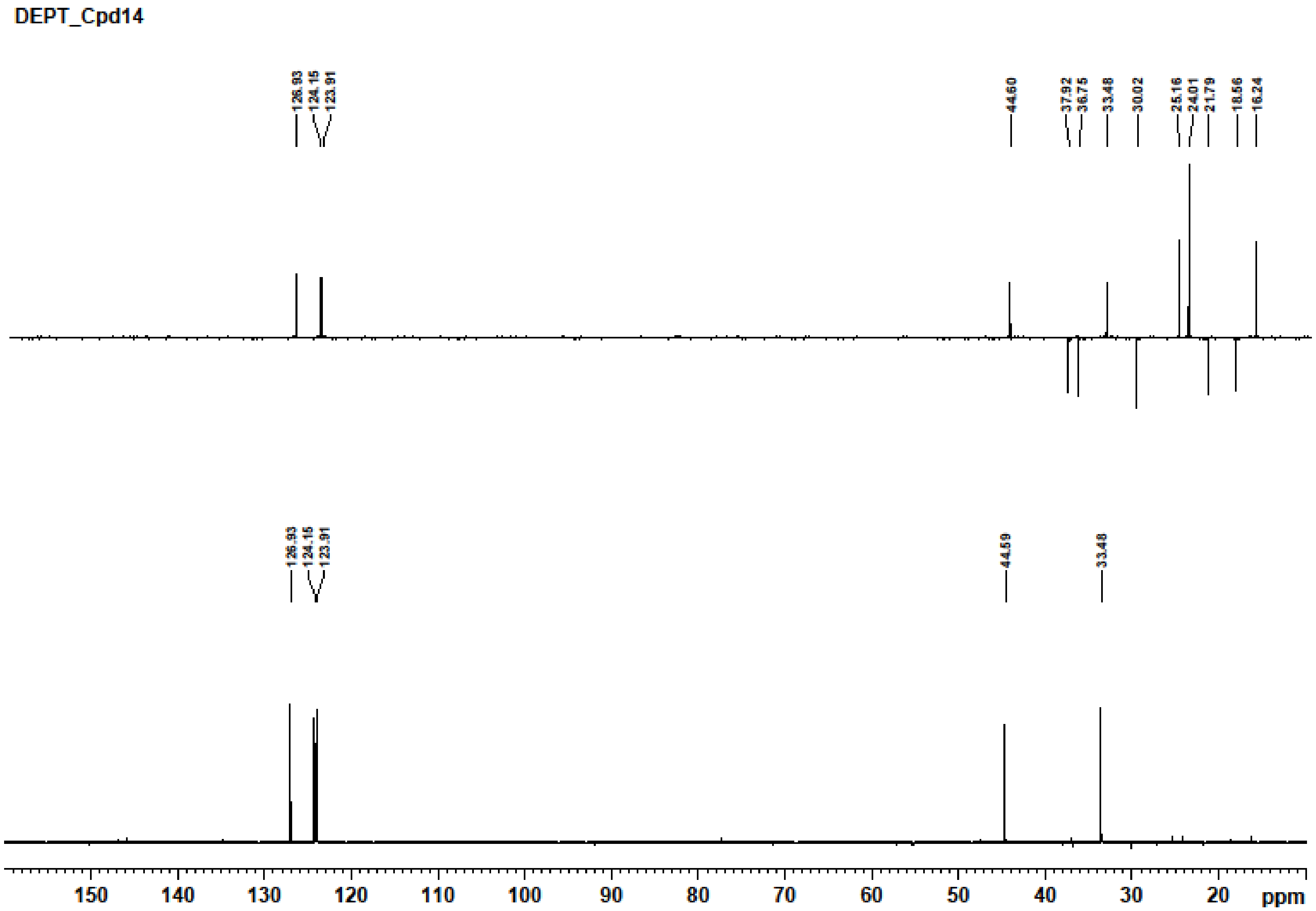

Figure S145. DEPT 135 & 90 NMR spectrum of compound **14** in $CDCl_3$

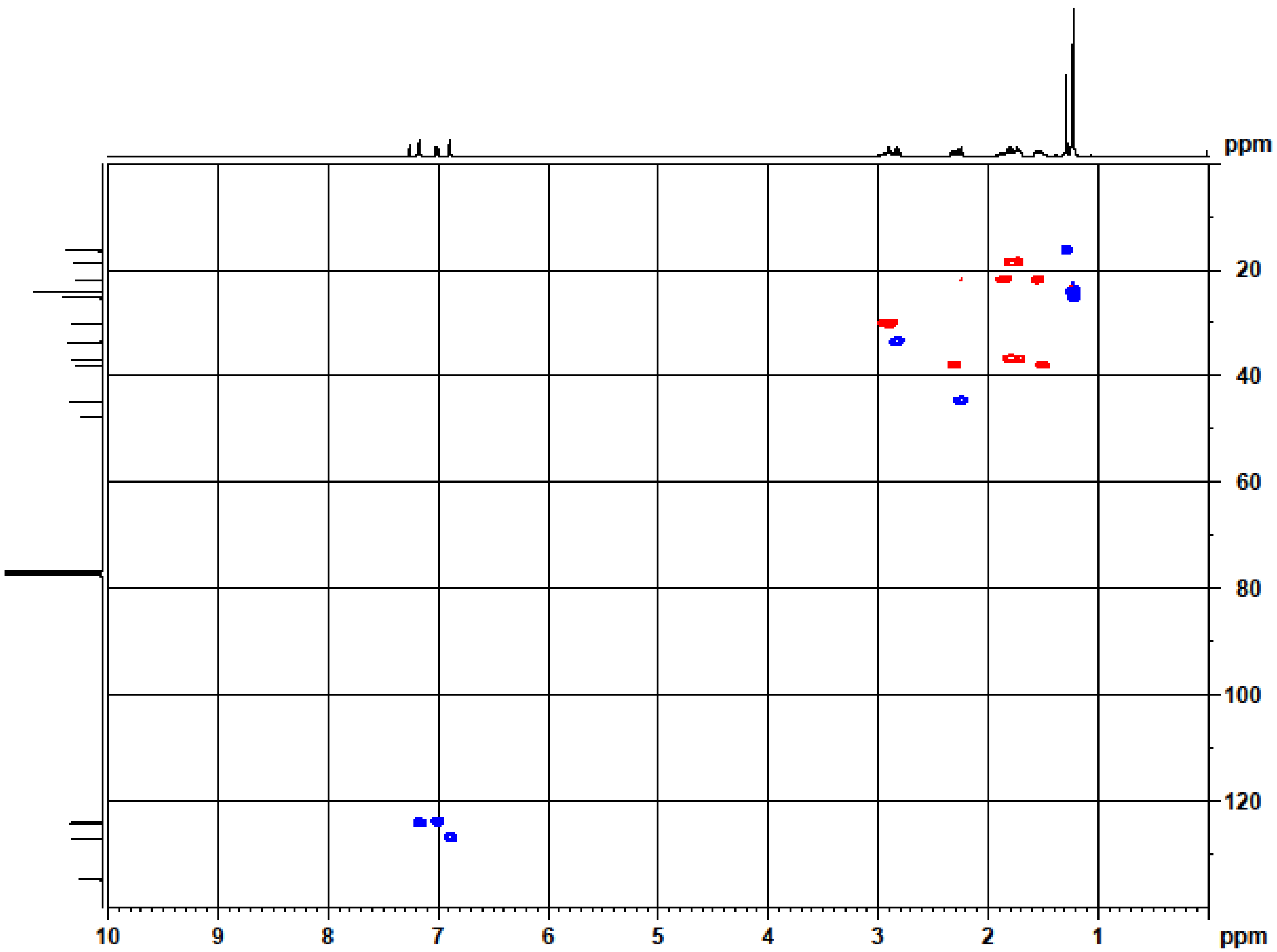

Figure S146. HSQC spectrum of compound **14** in $CDCl_3$

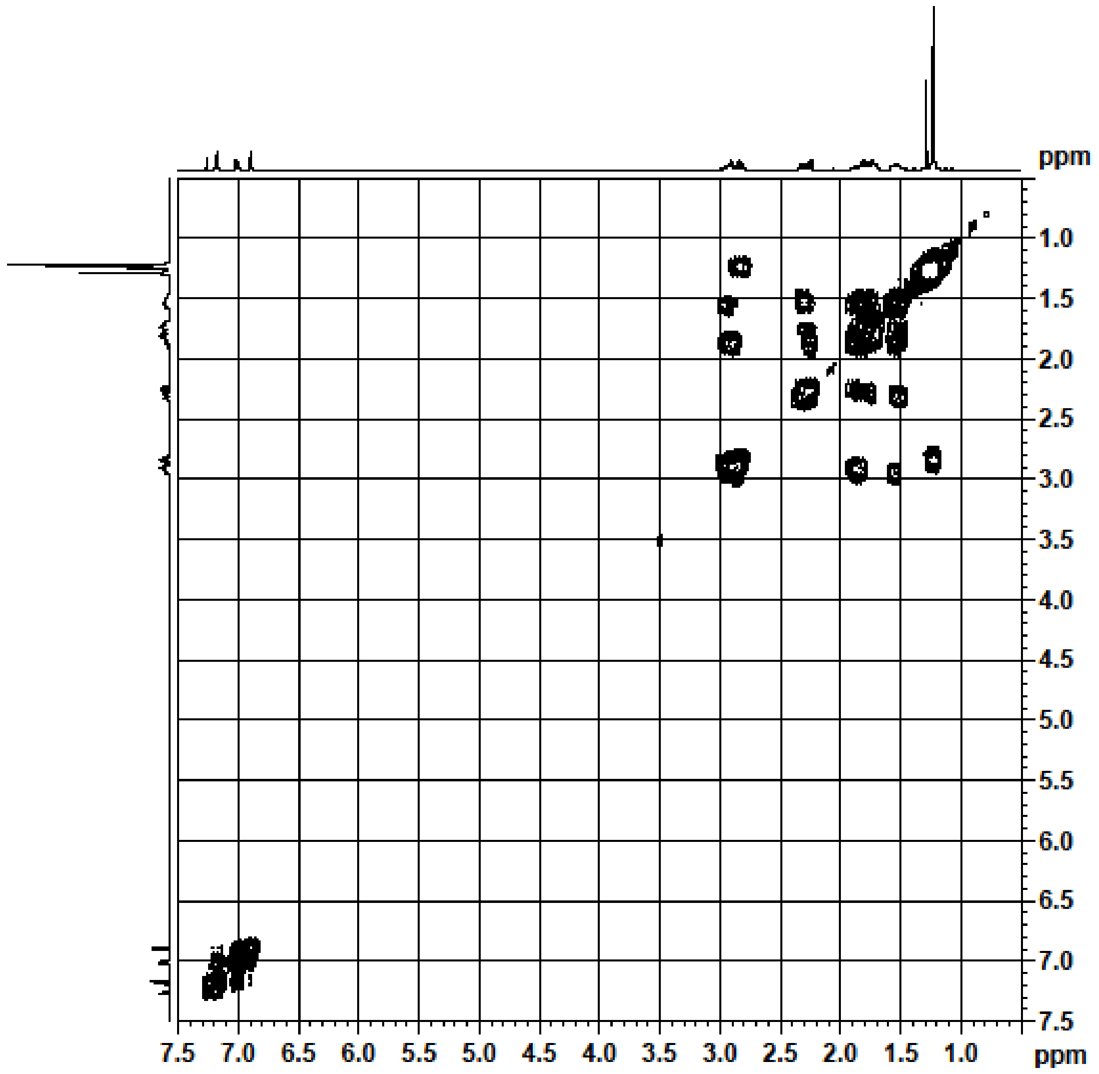

Figure S147. COSY spectrum of compound **14** in $CDCl_3$

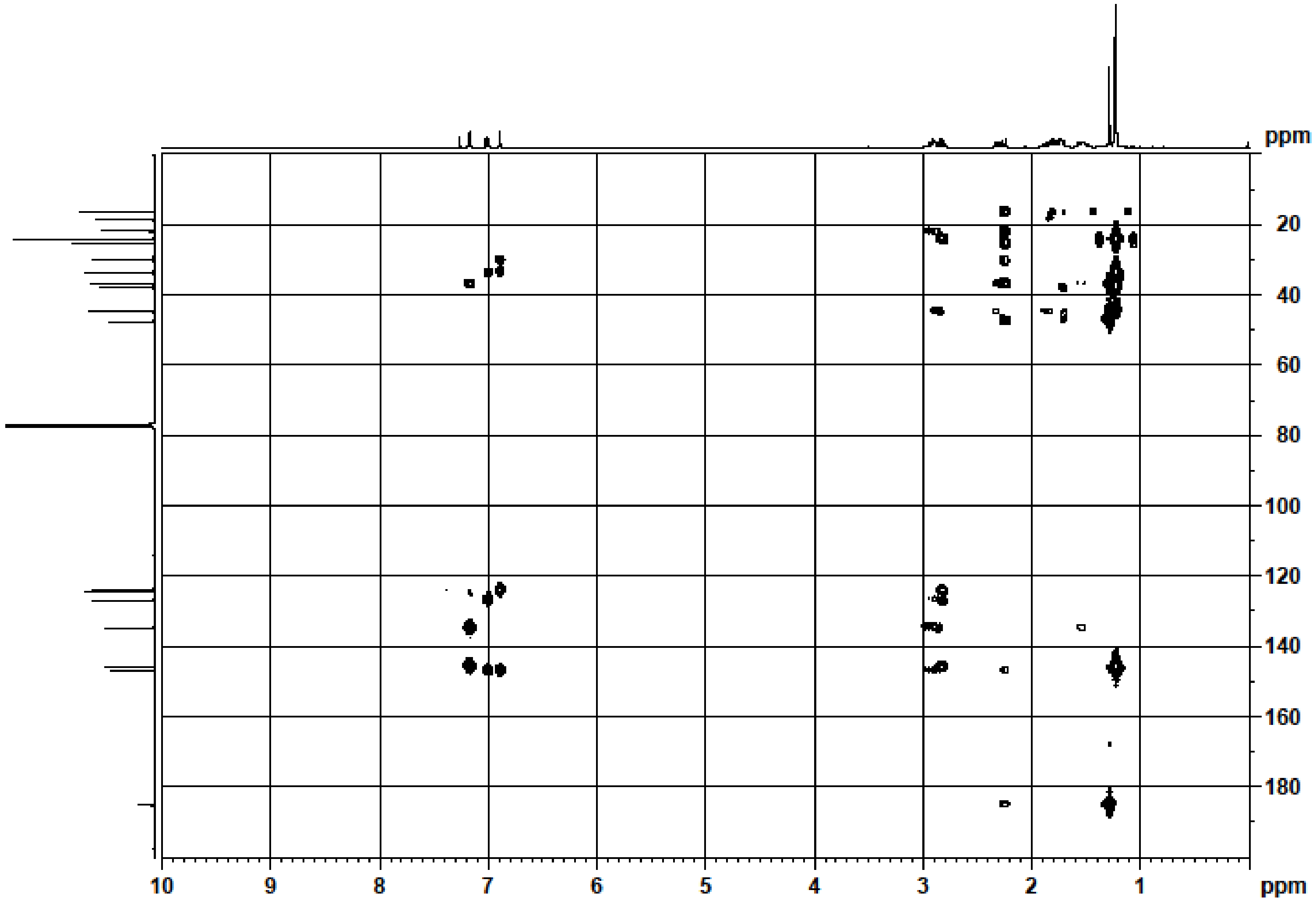

Figure S148. HMBC spectrum of compound **14** in $CDCl_3$

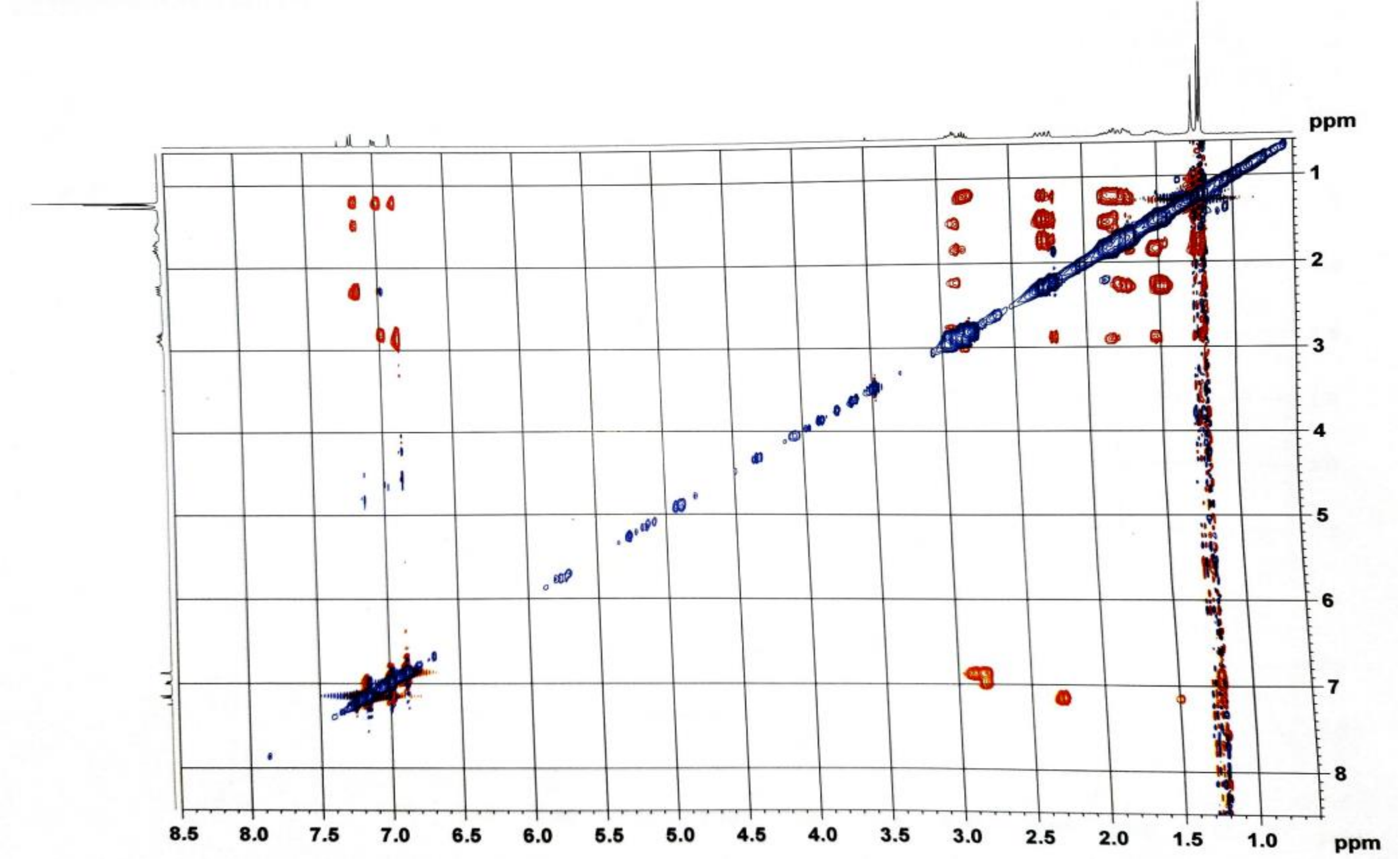

Figure S149. NOESY spectrum of **14** in $CDCl_3$

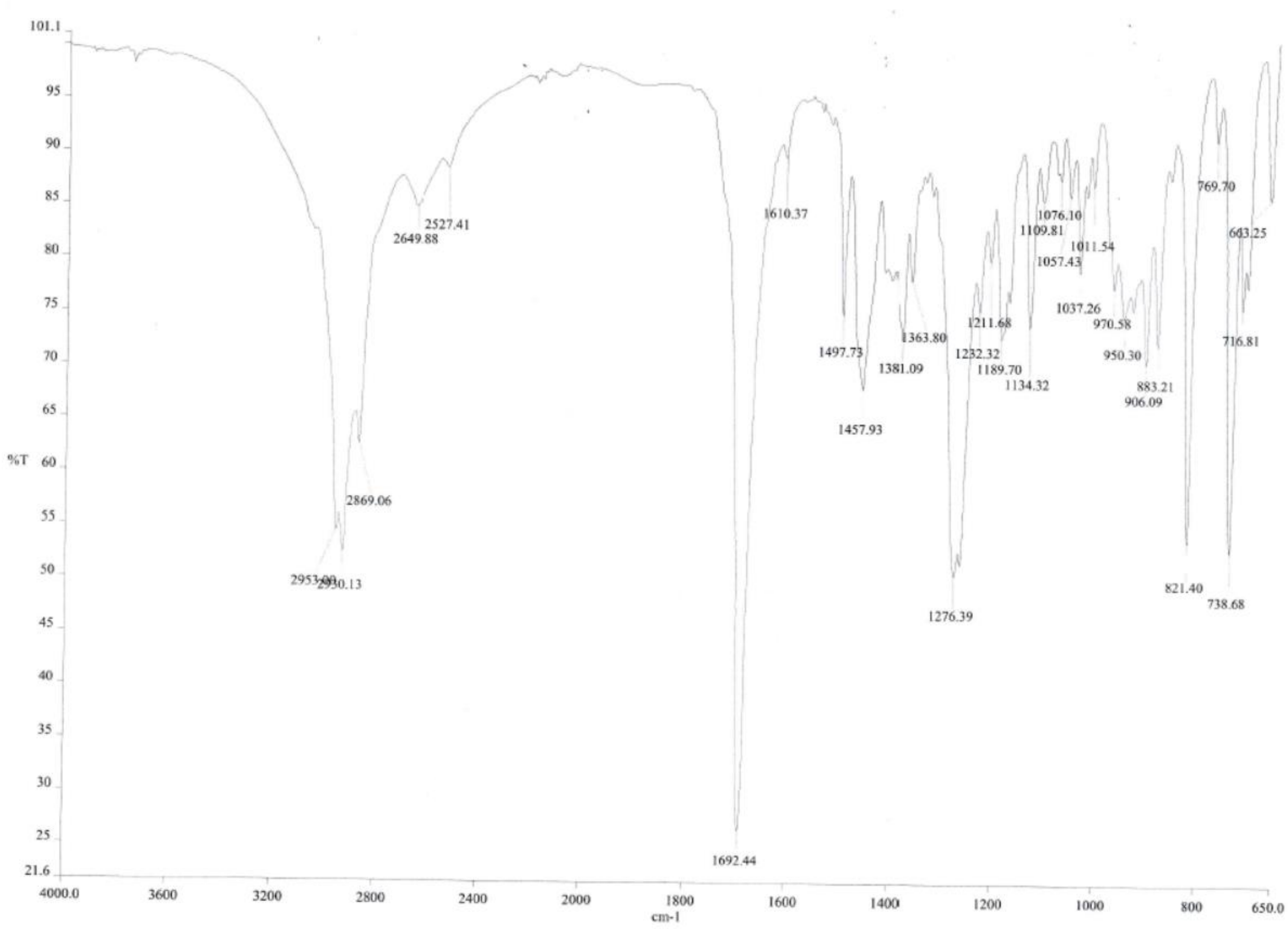

Figure S150. IR spectrum of compound **14**

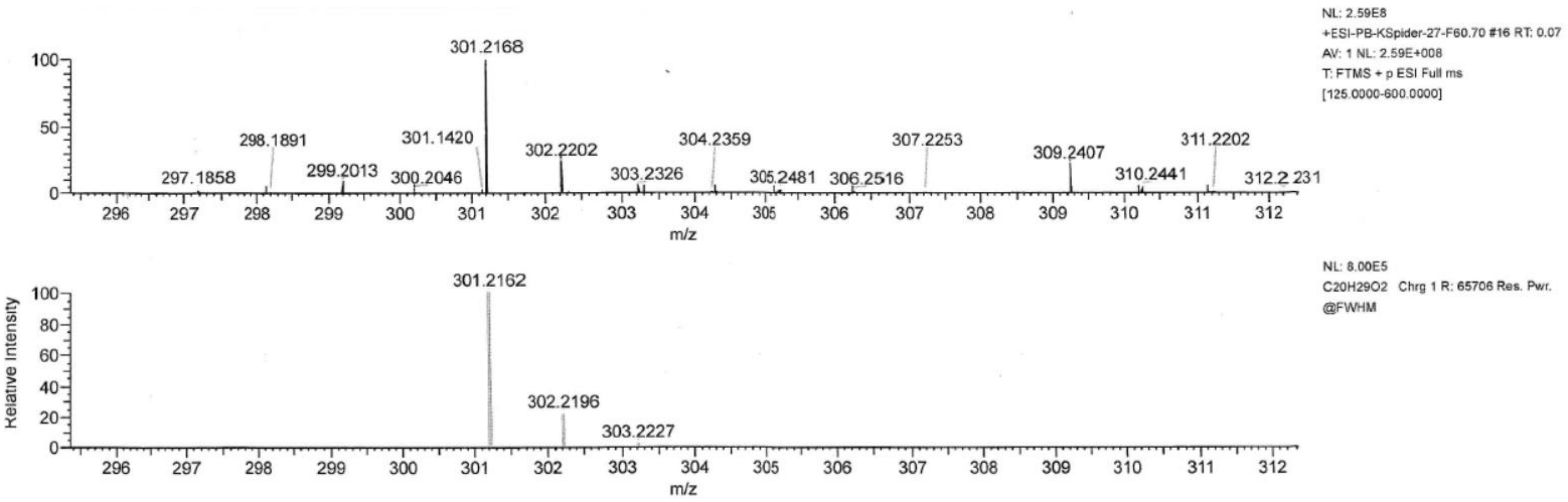

Peak Mass	Display Formula	Delta [ppm]	Theo. mass	MSMS Matched Fragments
301.2168	$C_{20}H_{29}O_2$ [M+H]$^+$	1.96	301.21621	(Collection)

Figure S151. HRESIMS spectrum of compound **14**

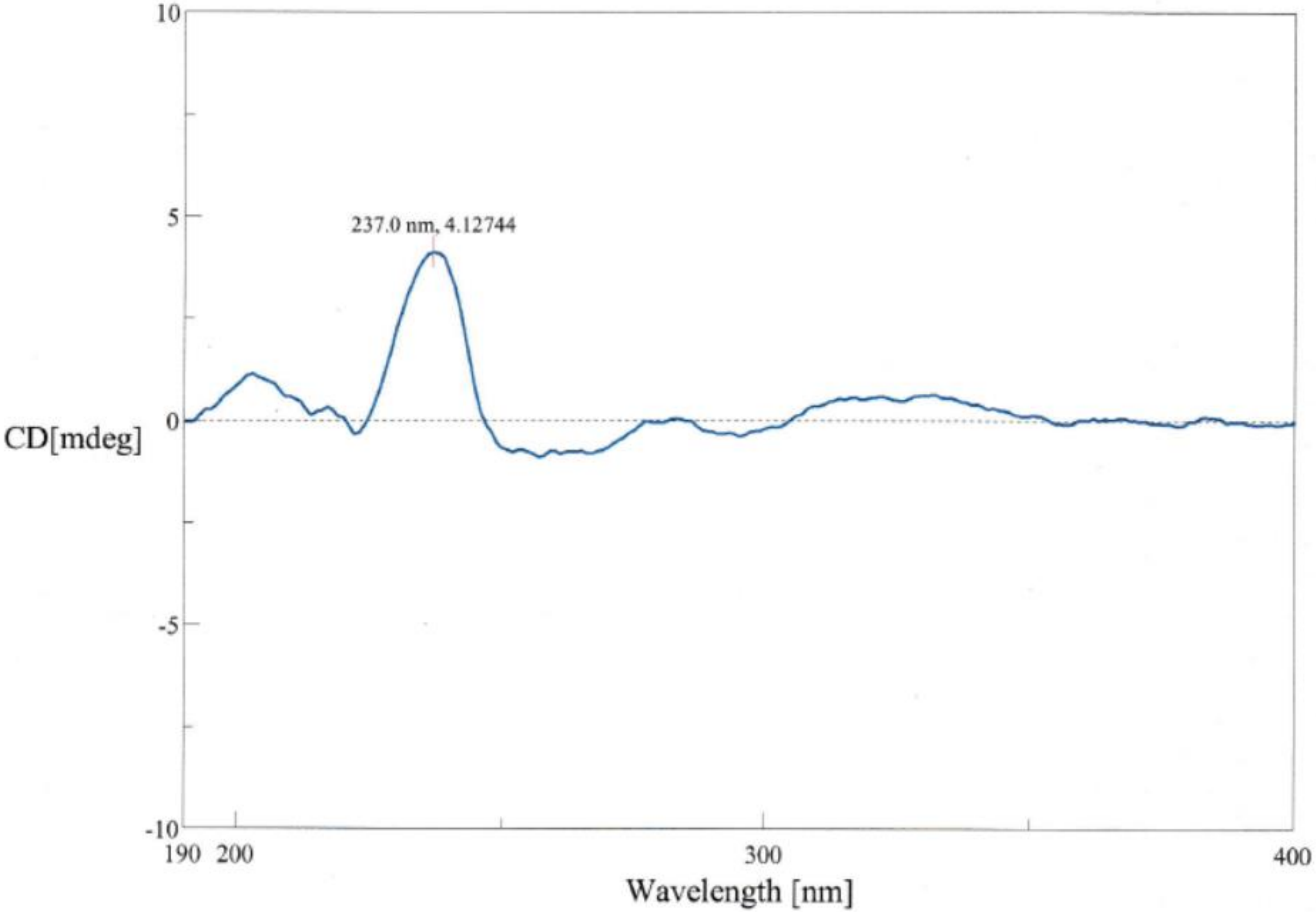

Figure S152. CD spectrum of compound **14**

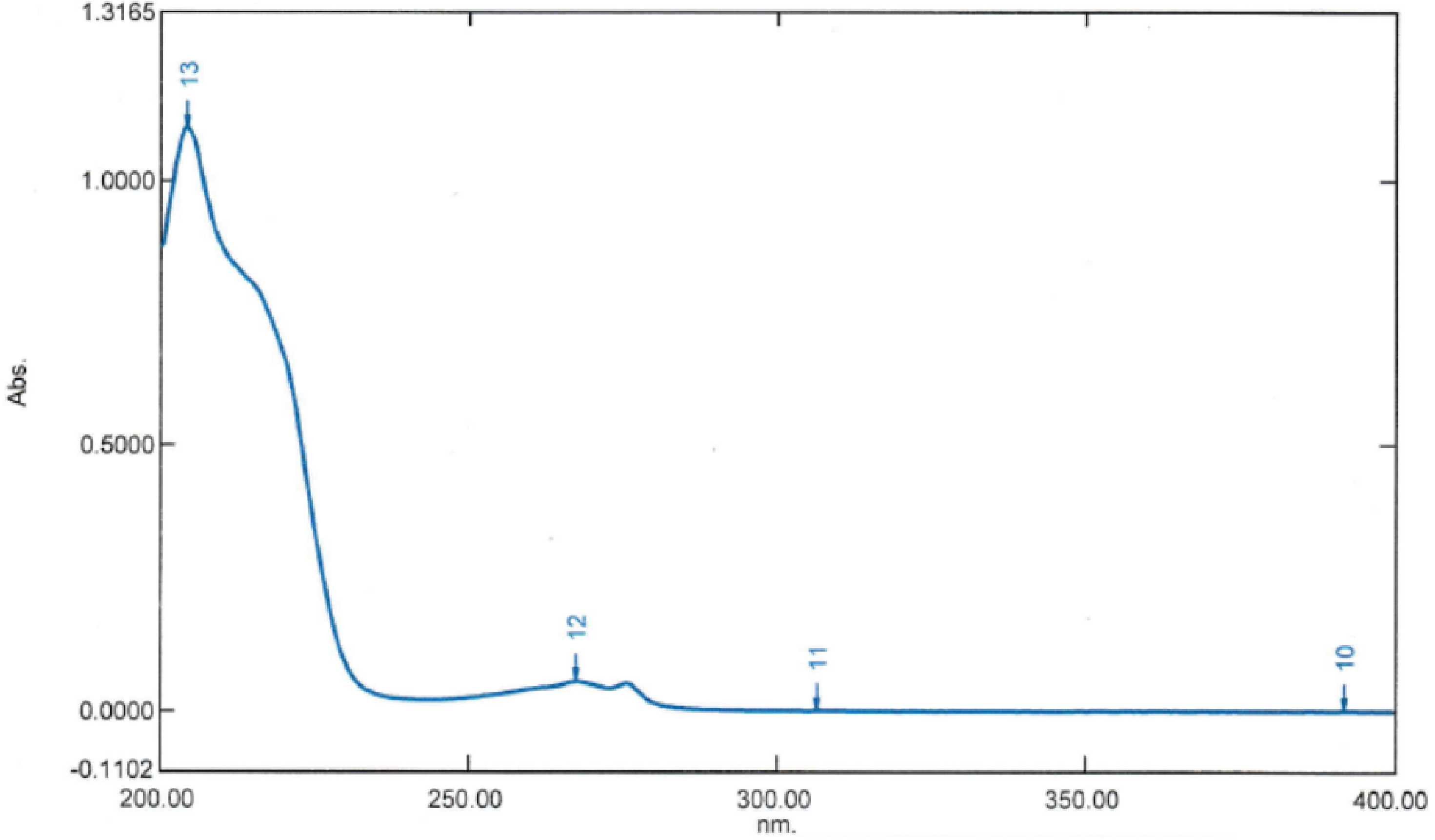

Figure S153. UV spectrum of compound **14**

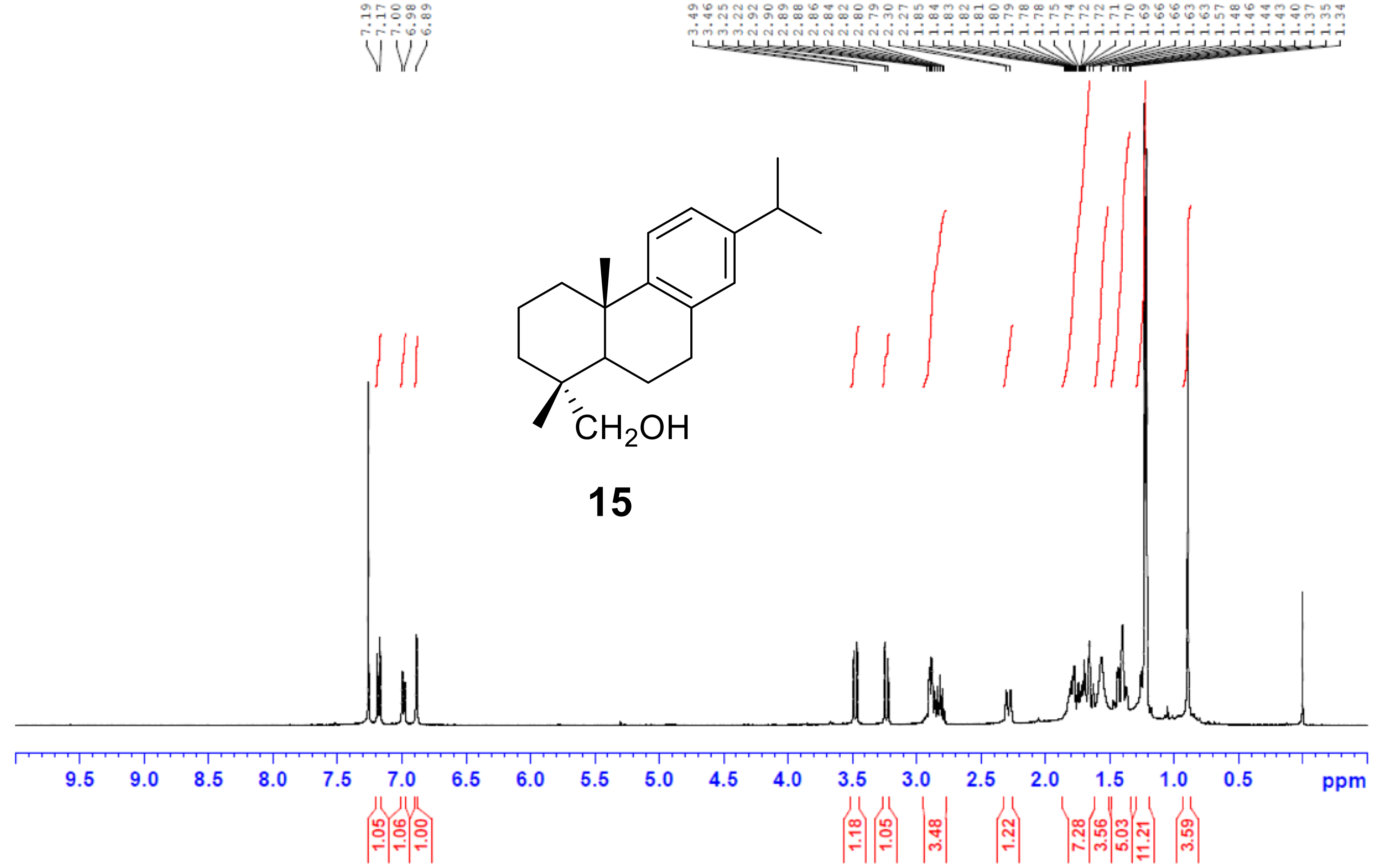

Figure S154. ^{1}H NMR (600 MHz) spectrum of compound **15** in $CDCl_3$

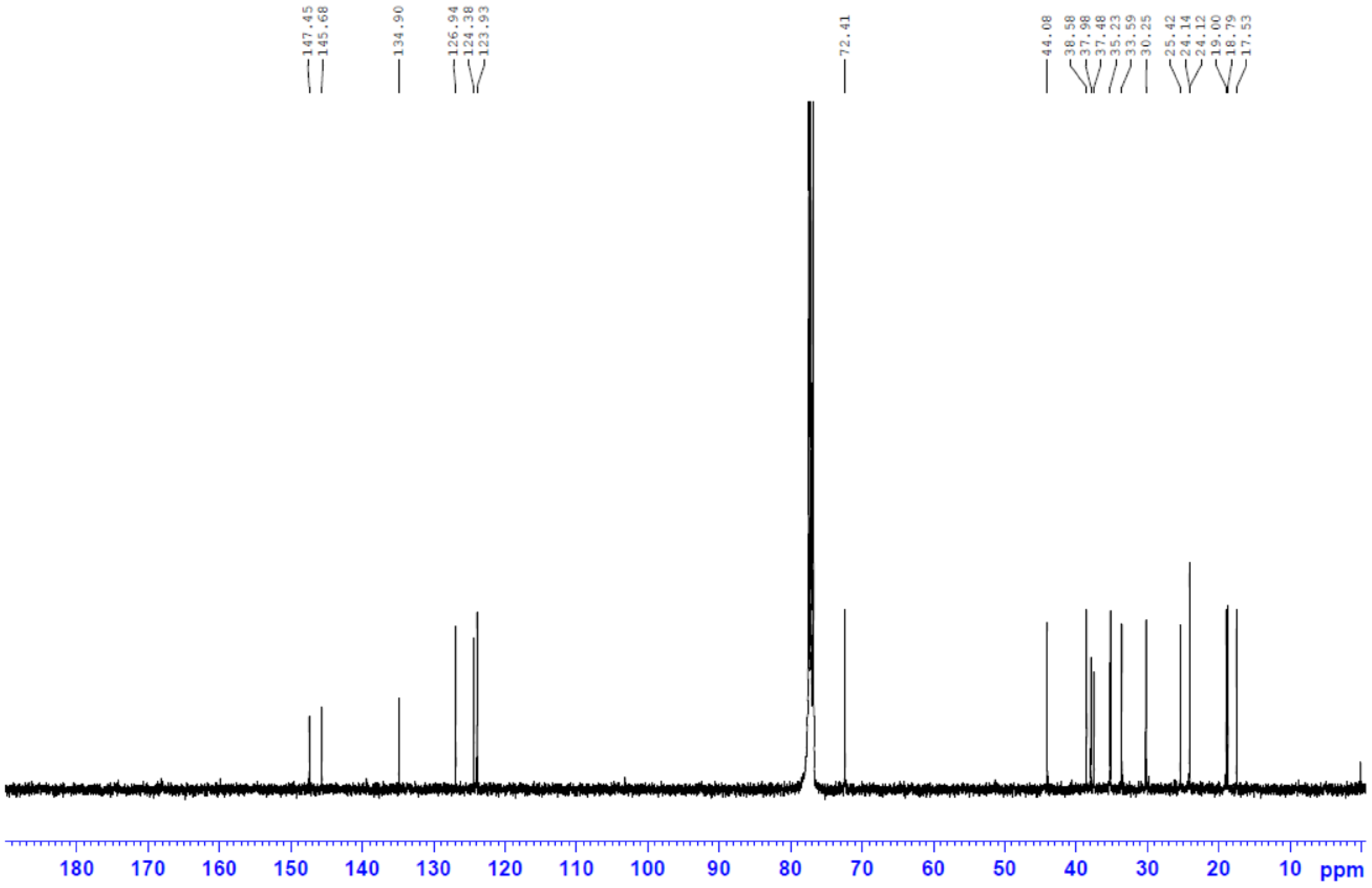

Figure S155. ^{13}C NMR (150 MHz) spectrum of compound **15** in $CDCl_3$

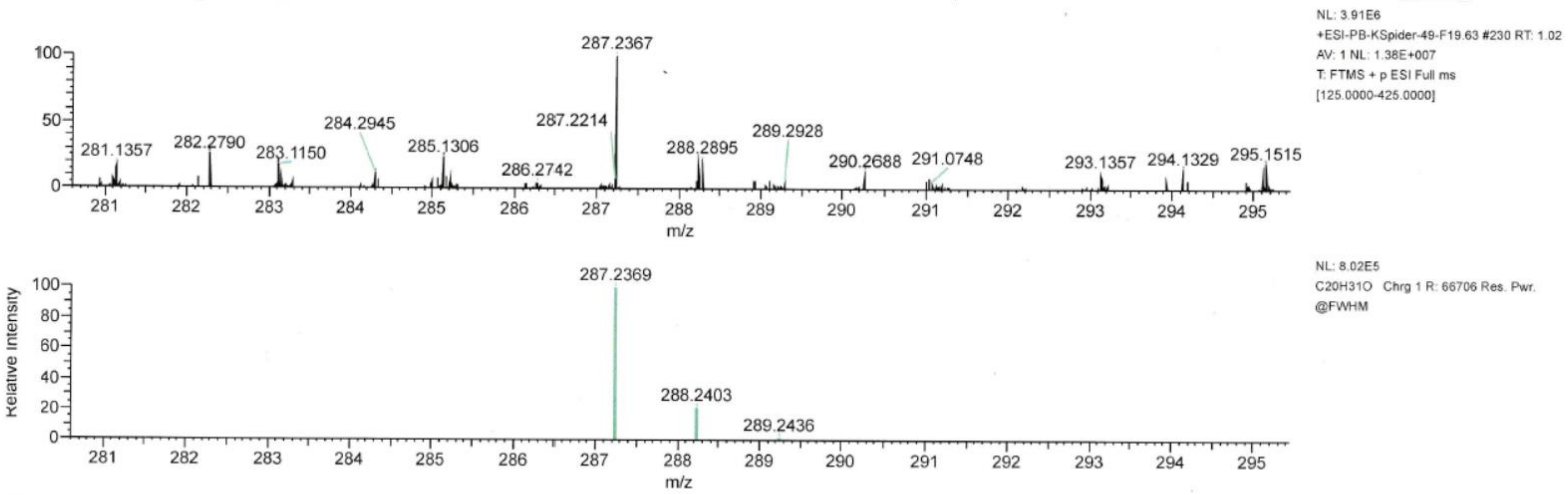

Peak Mass	Display Formula	Delta [ppm]	Theo. mass	MSMS Matched Fragments
287.2367	$C_{20}H_{31}O$ [M+H]+	-0.86	287.23694	(Collection)

Figure S156. HRESIMS spectrum of compound **15**

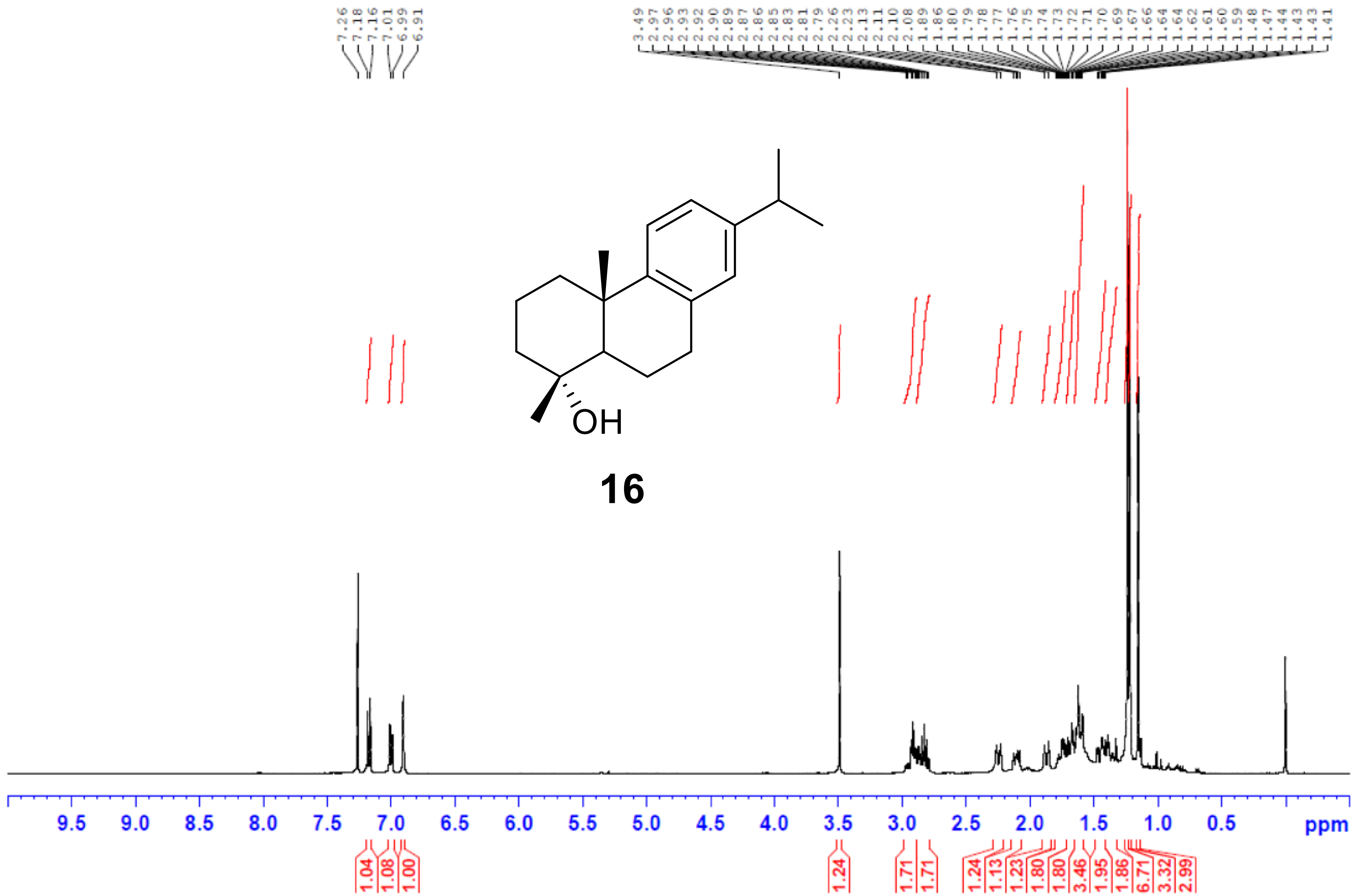

16

Figure S157. ^{1}H NMR (600 MHz) spectrum of compound **16** in $CDCl_3$

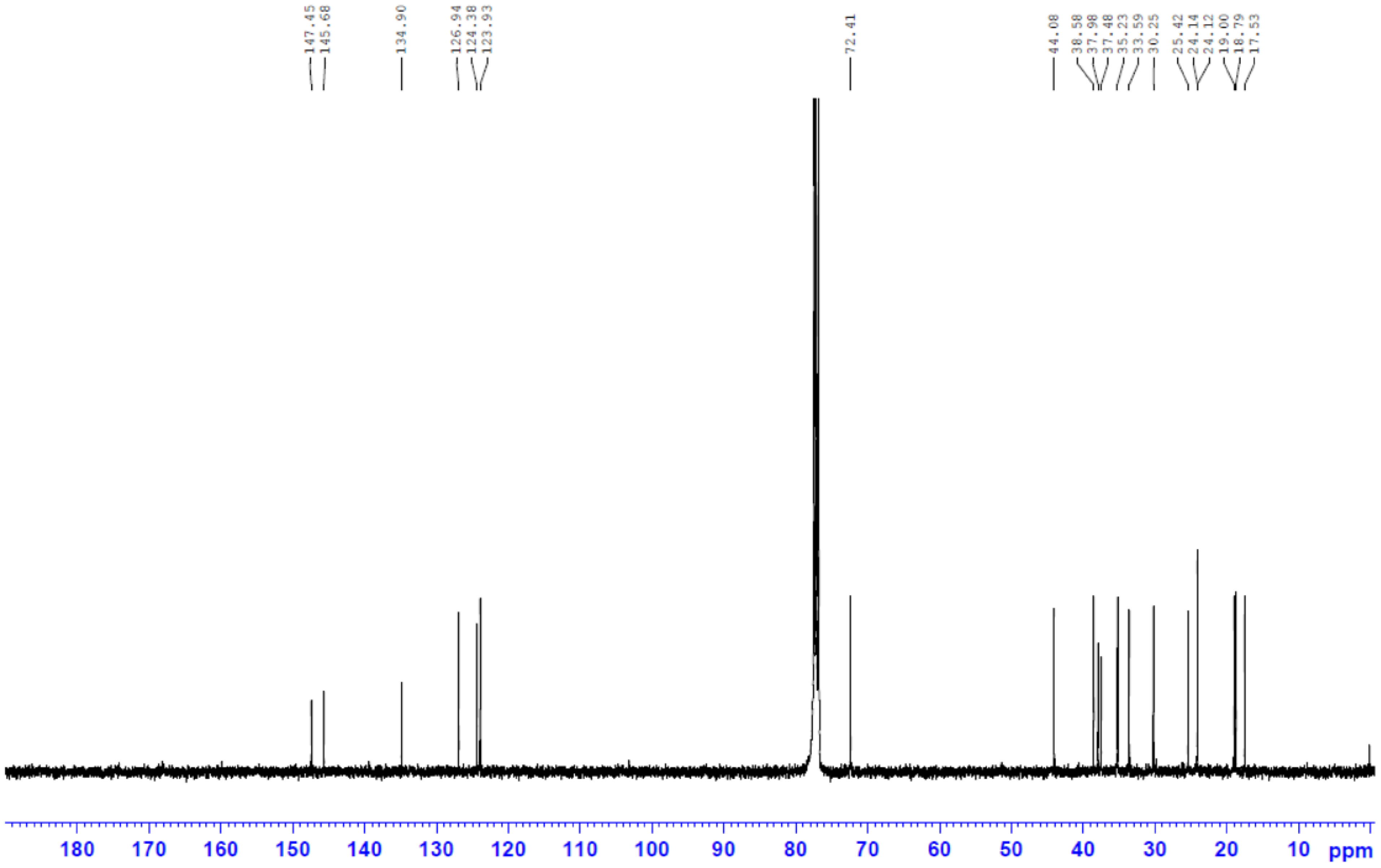

Figure S158. ^{13}C NMR (150 MHz) spectrum of compound **16** in $CDCl_3$

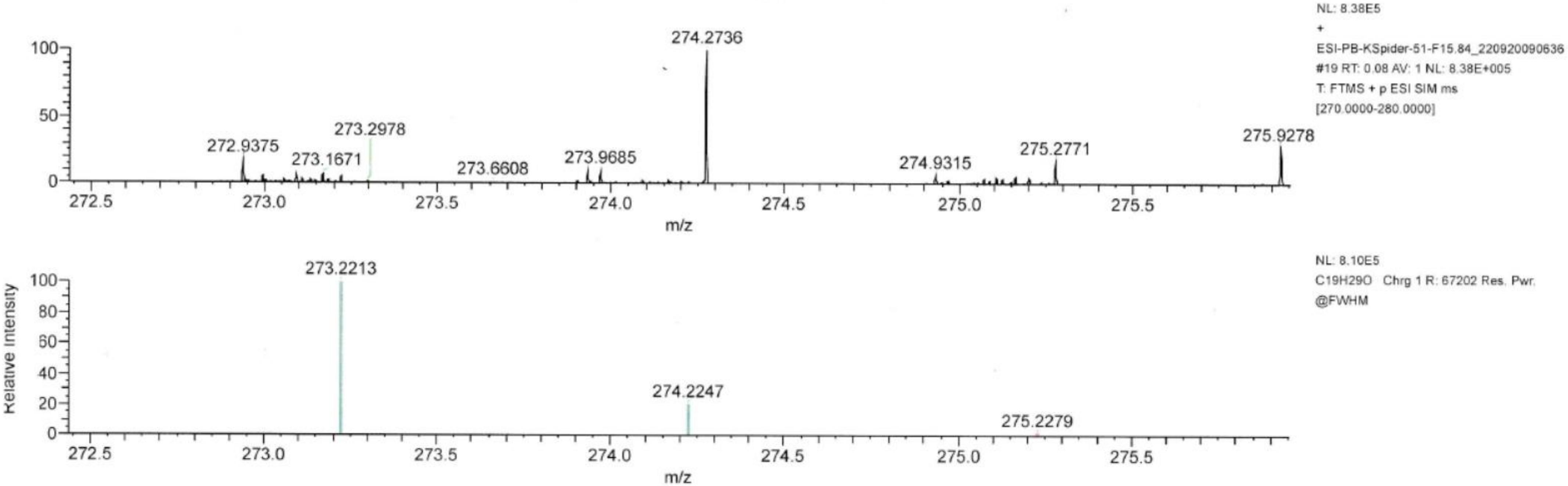

Peak Mass	Display Formula	Delta [ppm]	Theo. mass	MSMS Matched Fragments
273.2211	$C_{19}H_{29}O$ [M+H]+	-0.82	273.22129	(Collection)

Figure S159. HRESIMS spectrum of compound **16**

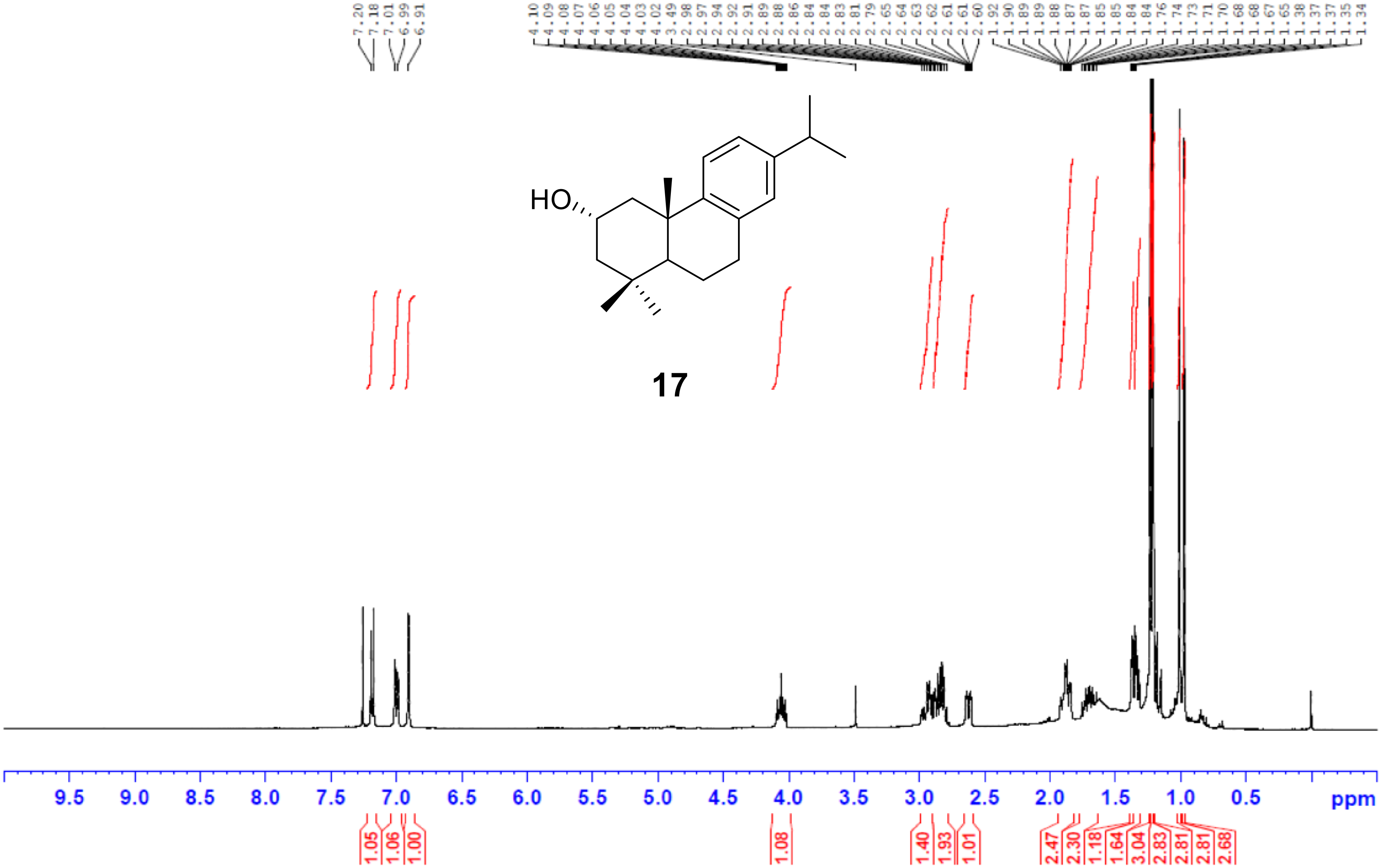

Figure S160. ^{1}H NMR (600 MHz) spectrum of compound **17** in $CDCl_3$

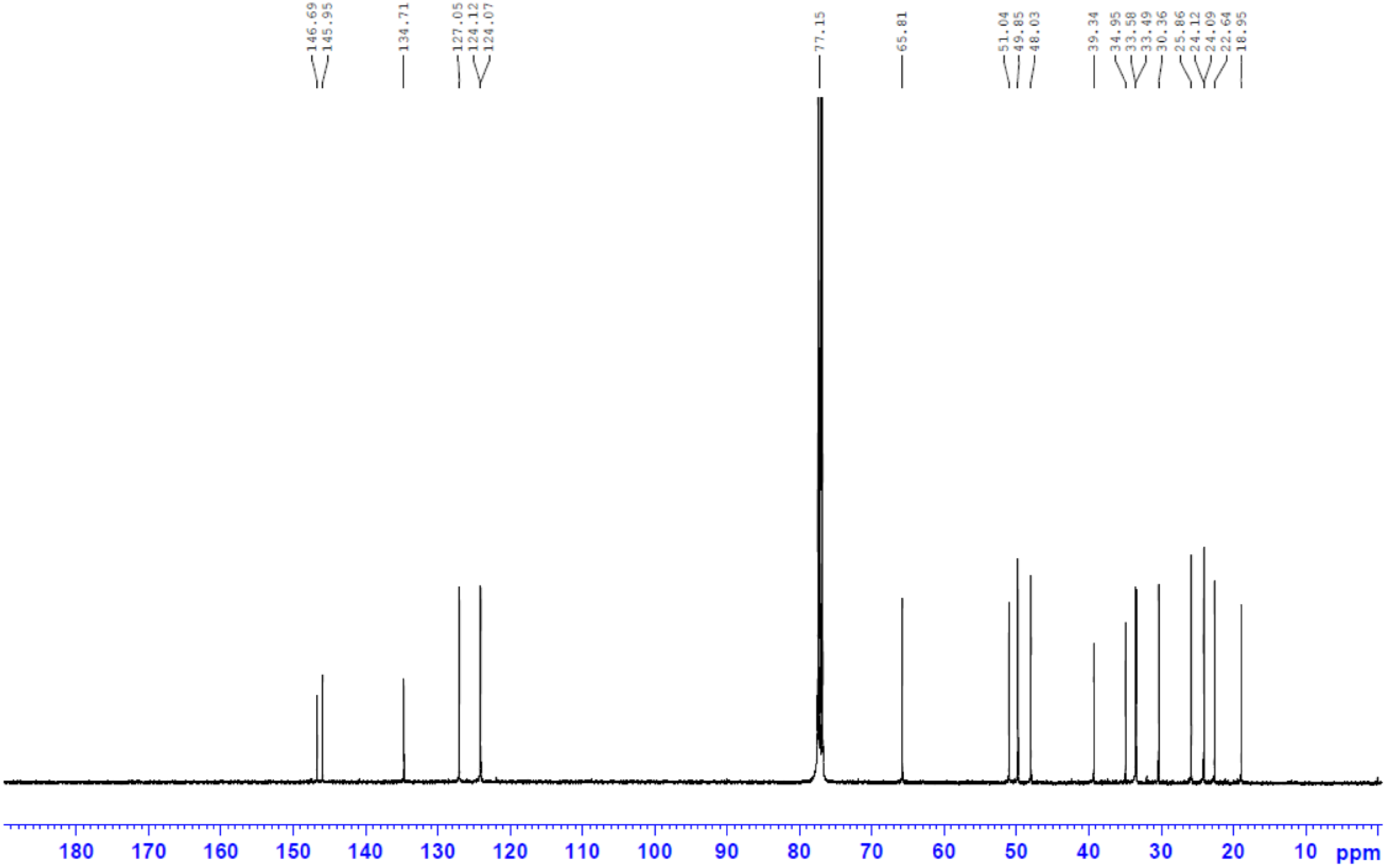

Figure S161. ^{13}C NMR (150 MHz) spectrum of compound **17** in $CDCl_3$

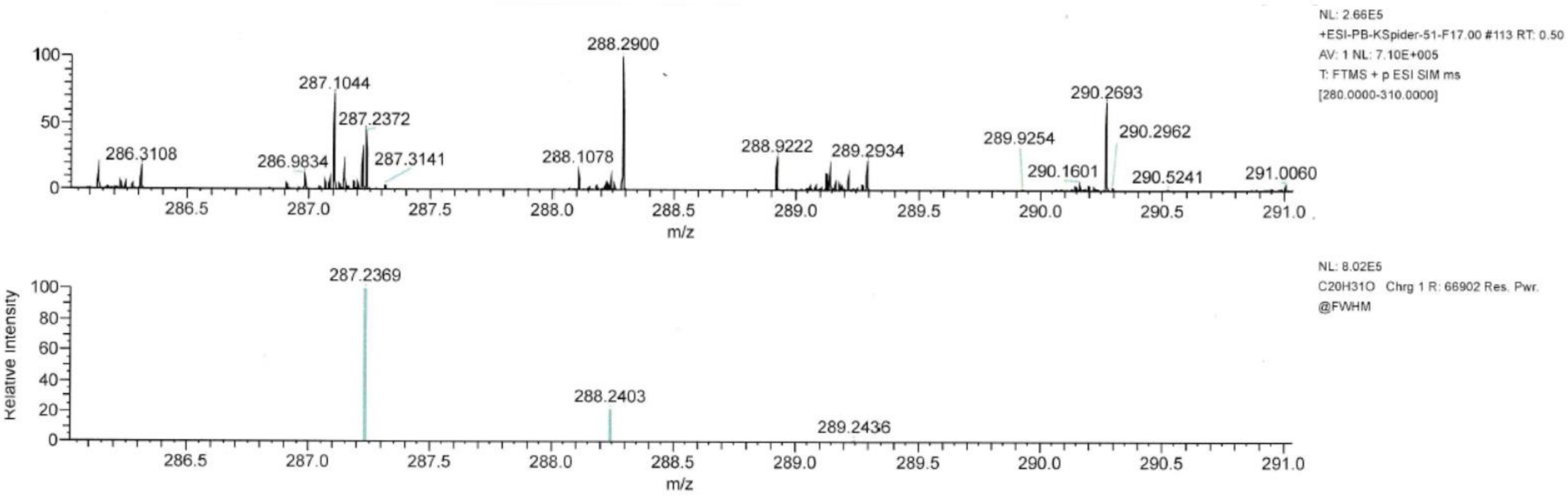

Peak Mass	Display Formula	Delta [ppm]	Theo. mass	MSMS Matched Fragments
287.2372	$C_{20}H_{31}O$ [M+H]+	0.94	287.23694	(Collection)

Figure S162. HRESIMS spectrum of compound **17**

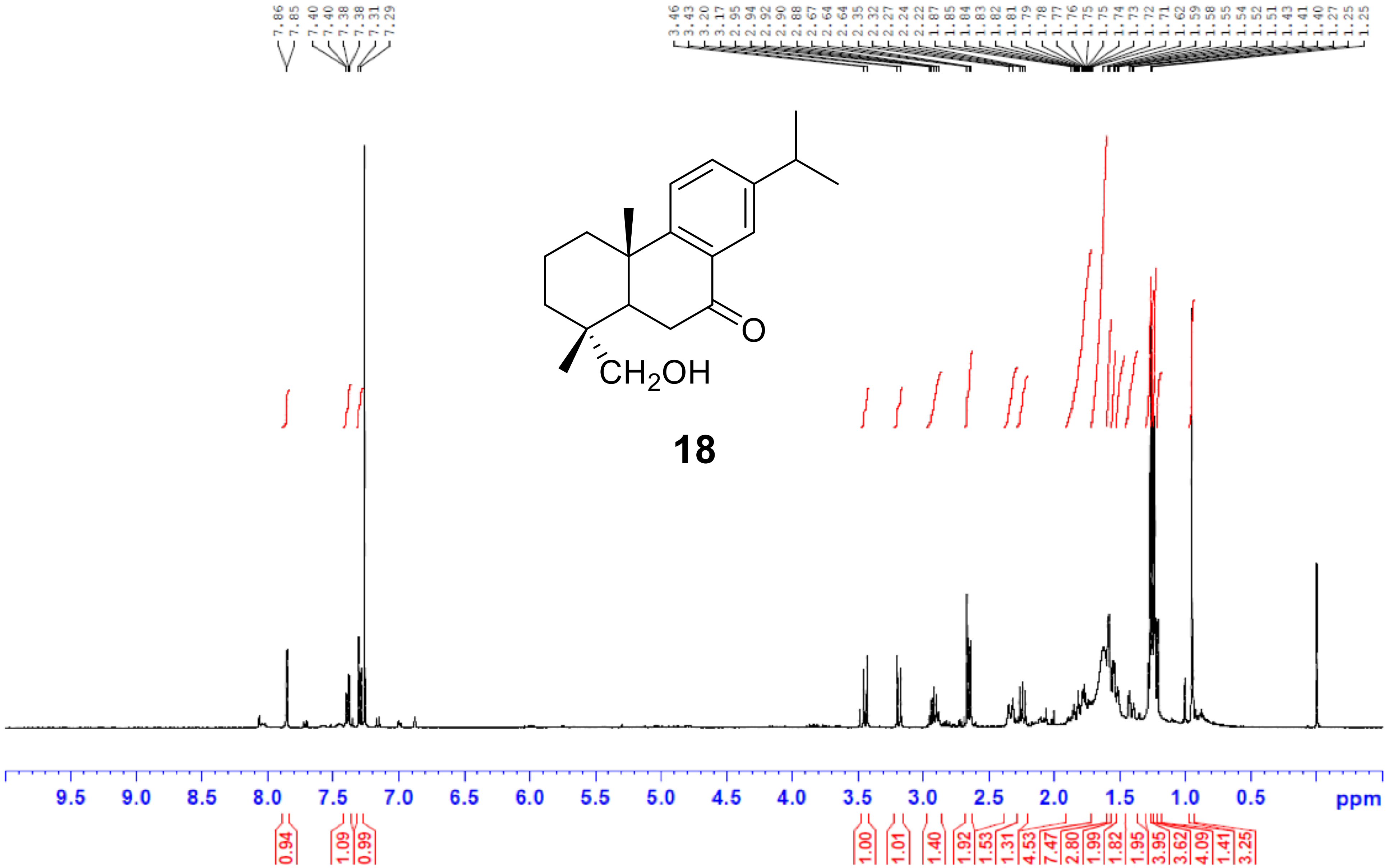

Figure S163. ^{1}H NMR (600 MHz) spectrum of compound **18** in $CDCl_3$

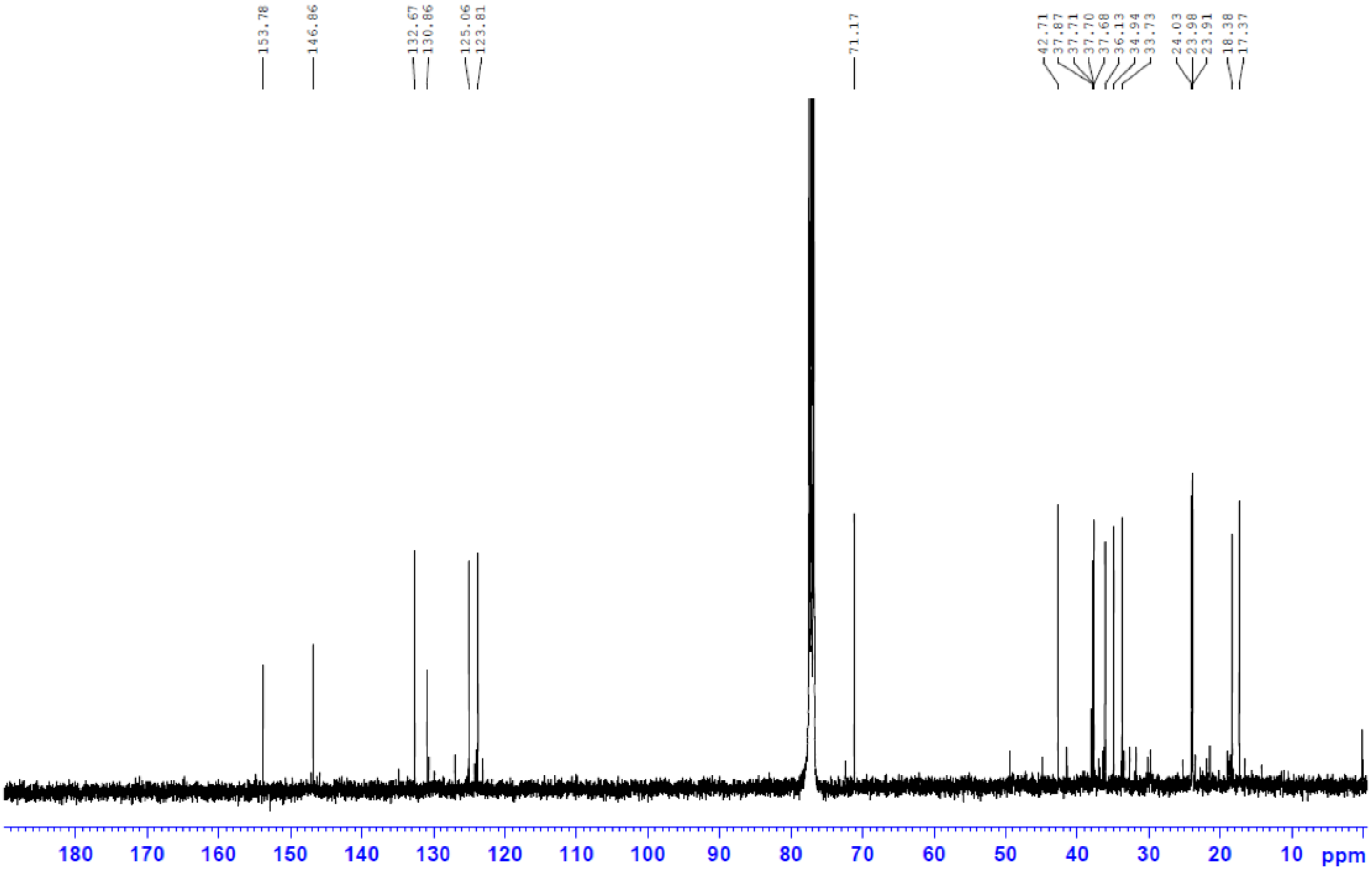

Figure S164. ^{13}C NMR (150 MHz) spectrum of compound **18** in $CDCl_3$

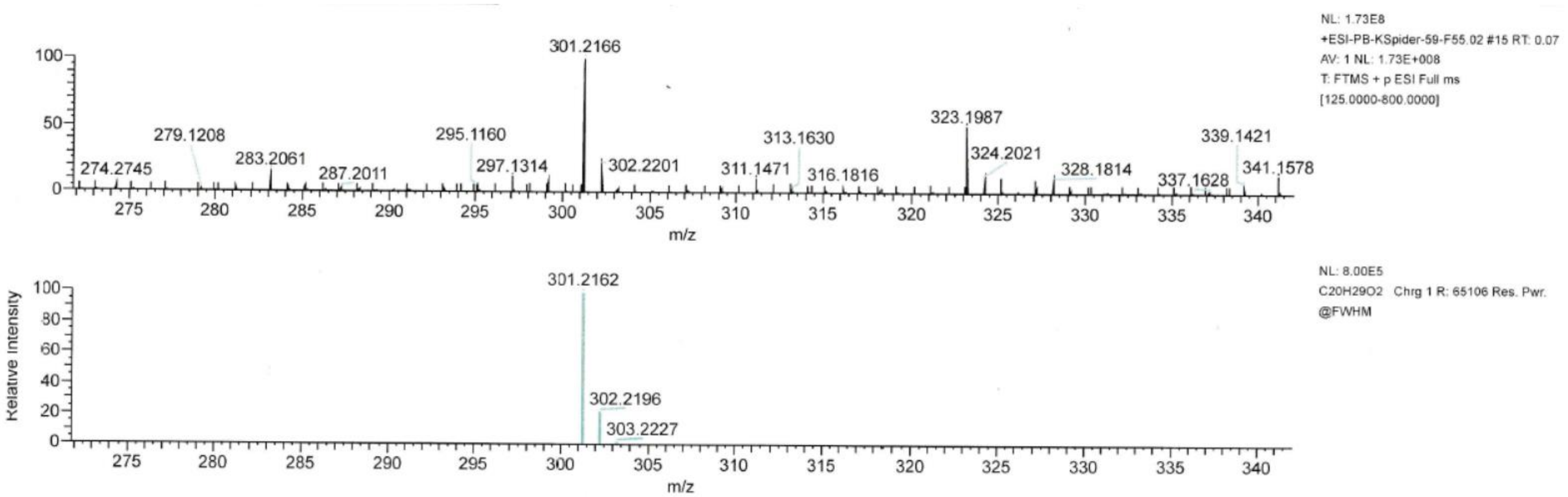

Peak Mass	Display Formula	Delta [ppm]	Theo. mass	MSMS Matched Fragments
301.2166	$C_{20}H_{29}O_2$ [M + H]+	1.35	301.21621	(Collection)

Figure S165. HRESIMS spectrum of compound **18**

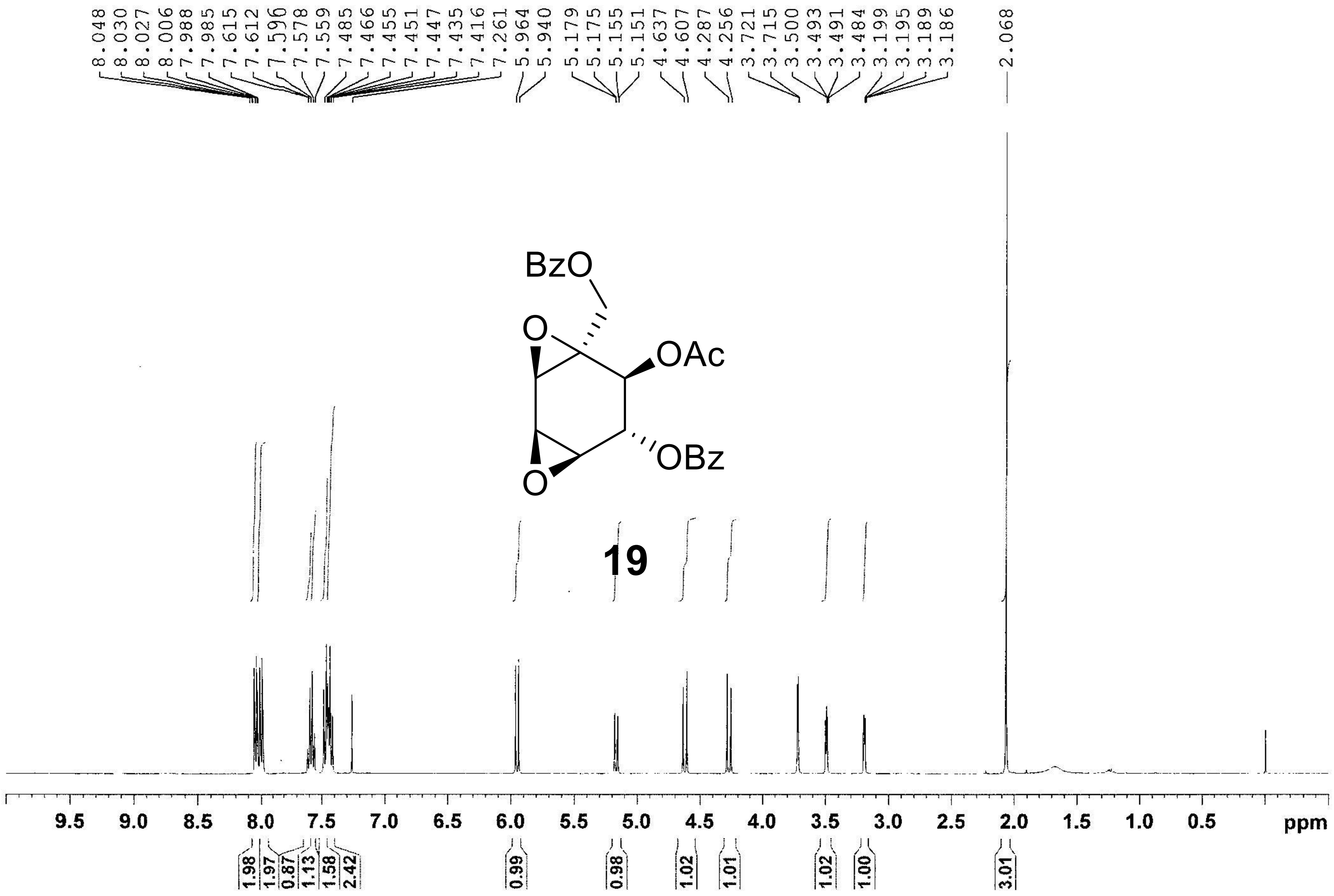

Figure S166. ^{1}H NMR (600 MHz) spectrum of compound **19** in $CDCl_3$

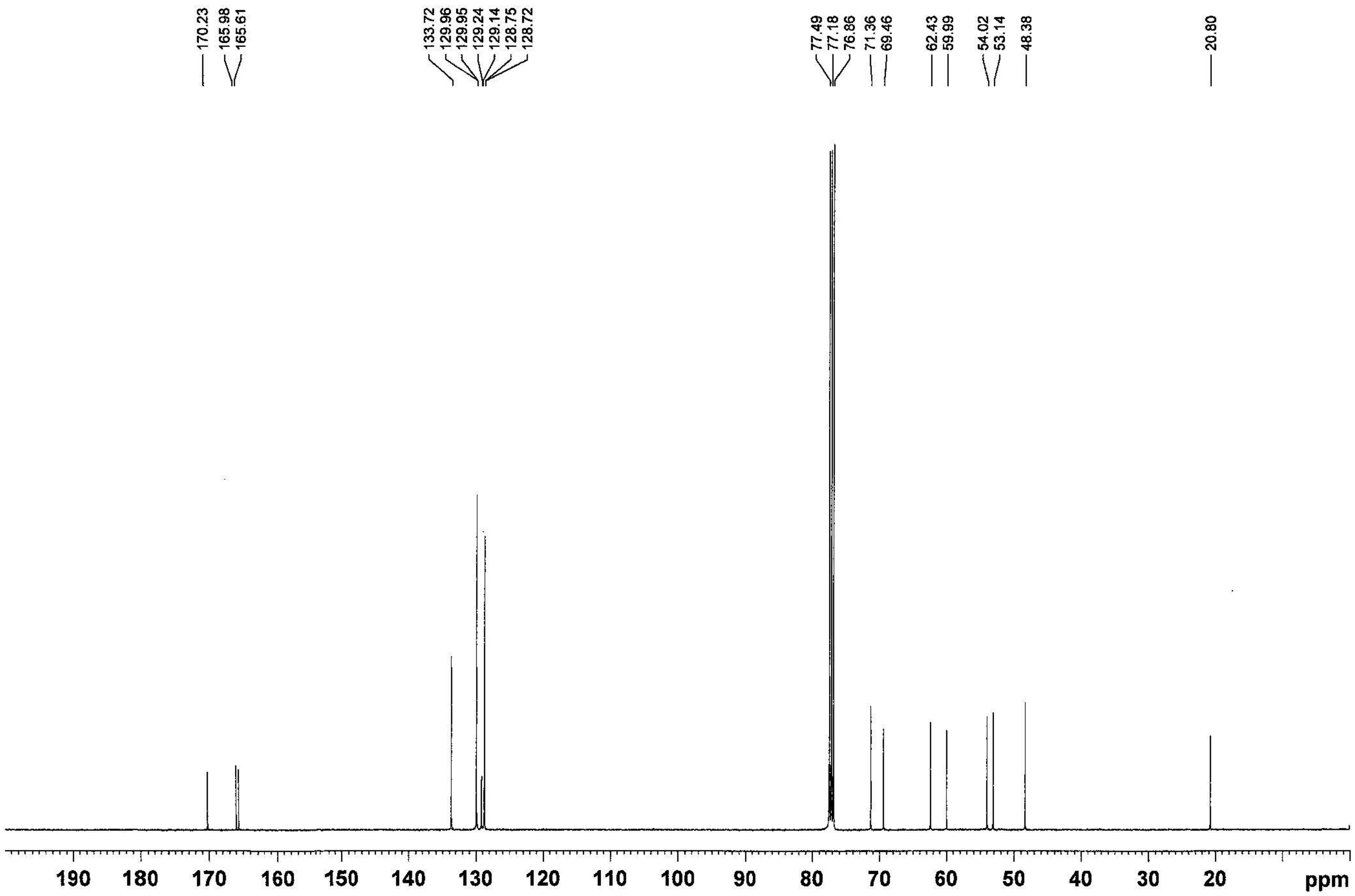

Figure S167. ^{13}C NMR (150 MHz) spectrum of compound **19** in $CDCl_3$

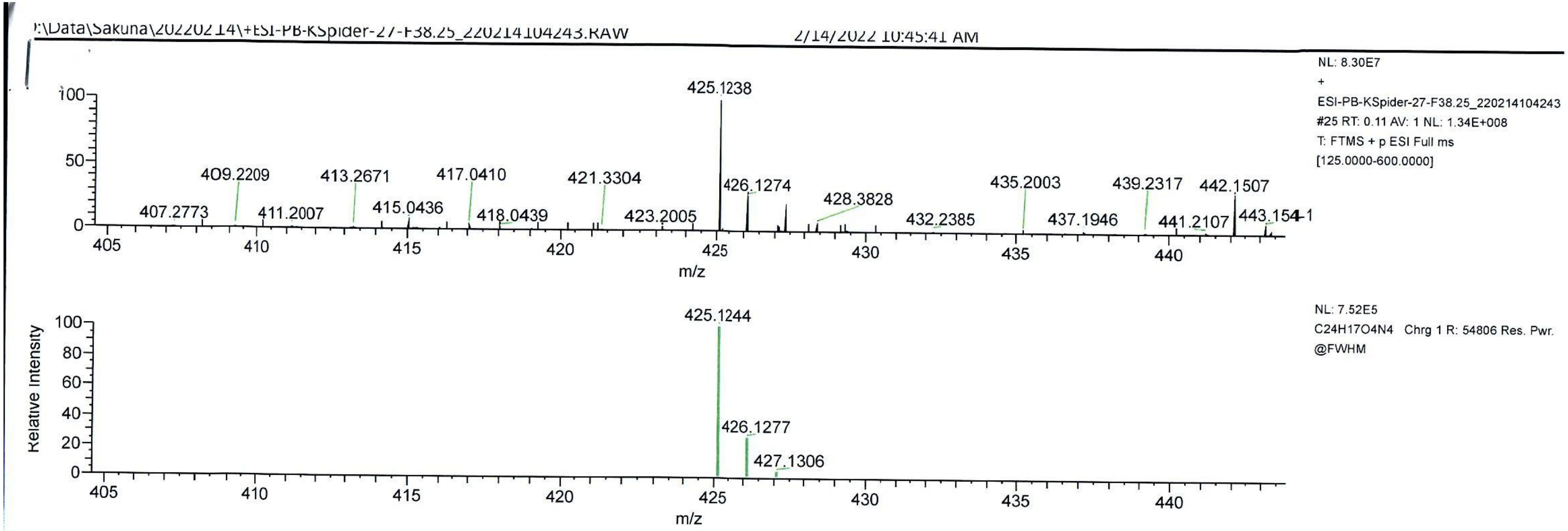

Peak Mass	Display Formula	Delta [ppm]	Theo. mass	MSMS Matched Fragments
425.1238	$C_{24}H_{17}O_4N_4$	-1.53	425.12443	(Collection)
425.1238	$C_{23}H_{21}O_8$ ✓	1.61	425.12309	(Collection)
425.1238	$C_{23}H_{14}N_8{}^{23}Na$	0.98	425.12336	(Collection)
425.1238	$C_{25}H_{13}N_8$	-4.68	425.12577	(Collection)
425.1238	$C_{22}H_{18}O_4N_4{}^{23}Na$	4.12	425.12203	(Collection)
425.1238	$C_{11}H_{18}O_7N_{10}{}^{23}Na$	-3.37	425.12521	(Collection)
425.1238	$C_{20}H_{13}O_2N_{10}$	4.78	425.12175	(Collection)

Figure S168. HRESIMS spectrum of compound **19**

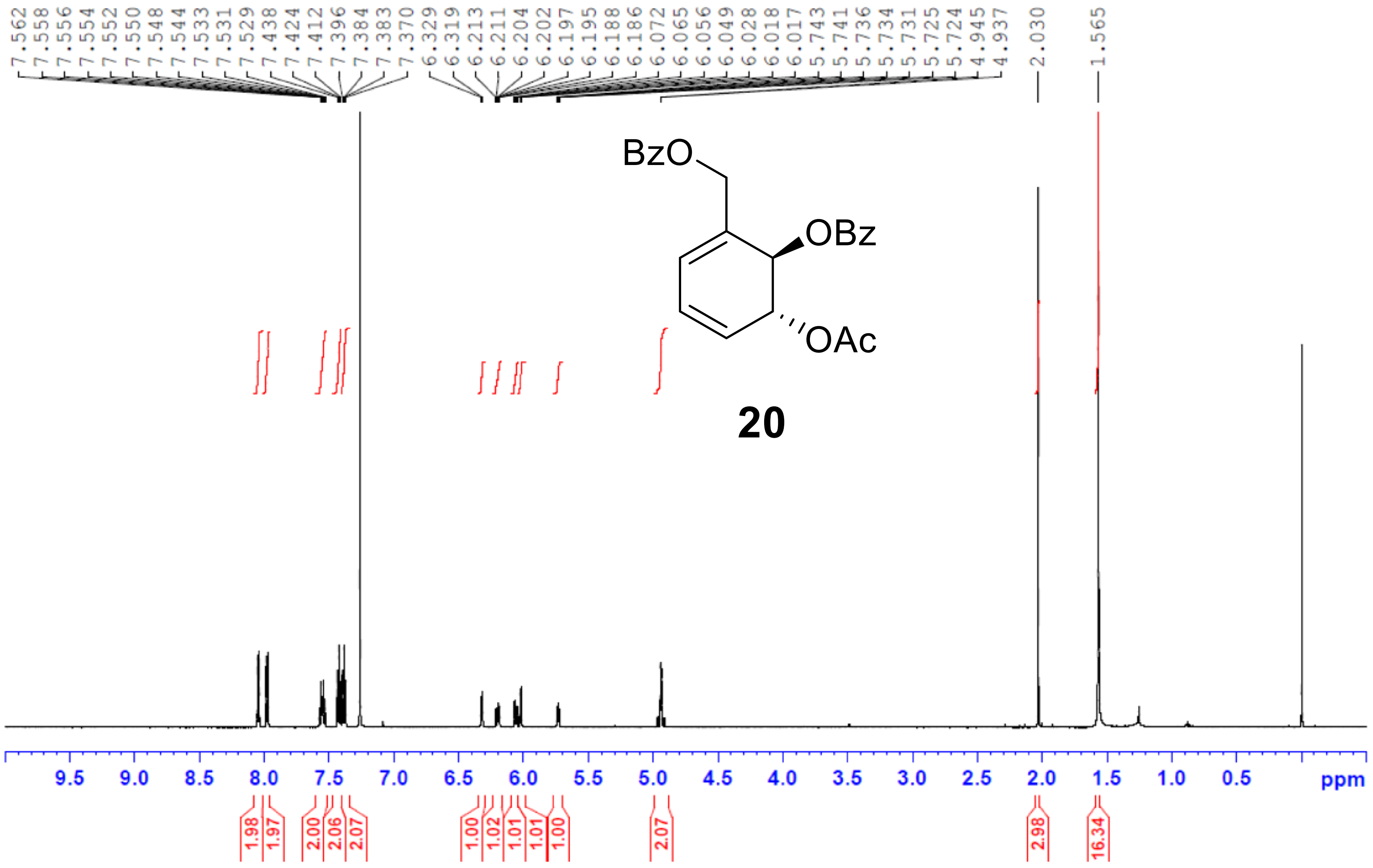

Figure S169. ^{1}H NMR (600 MHz) spectrum of compound **20** in $CDCl_3$

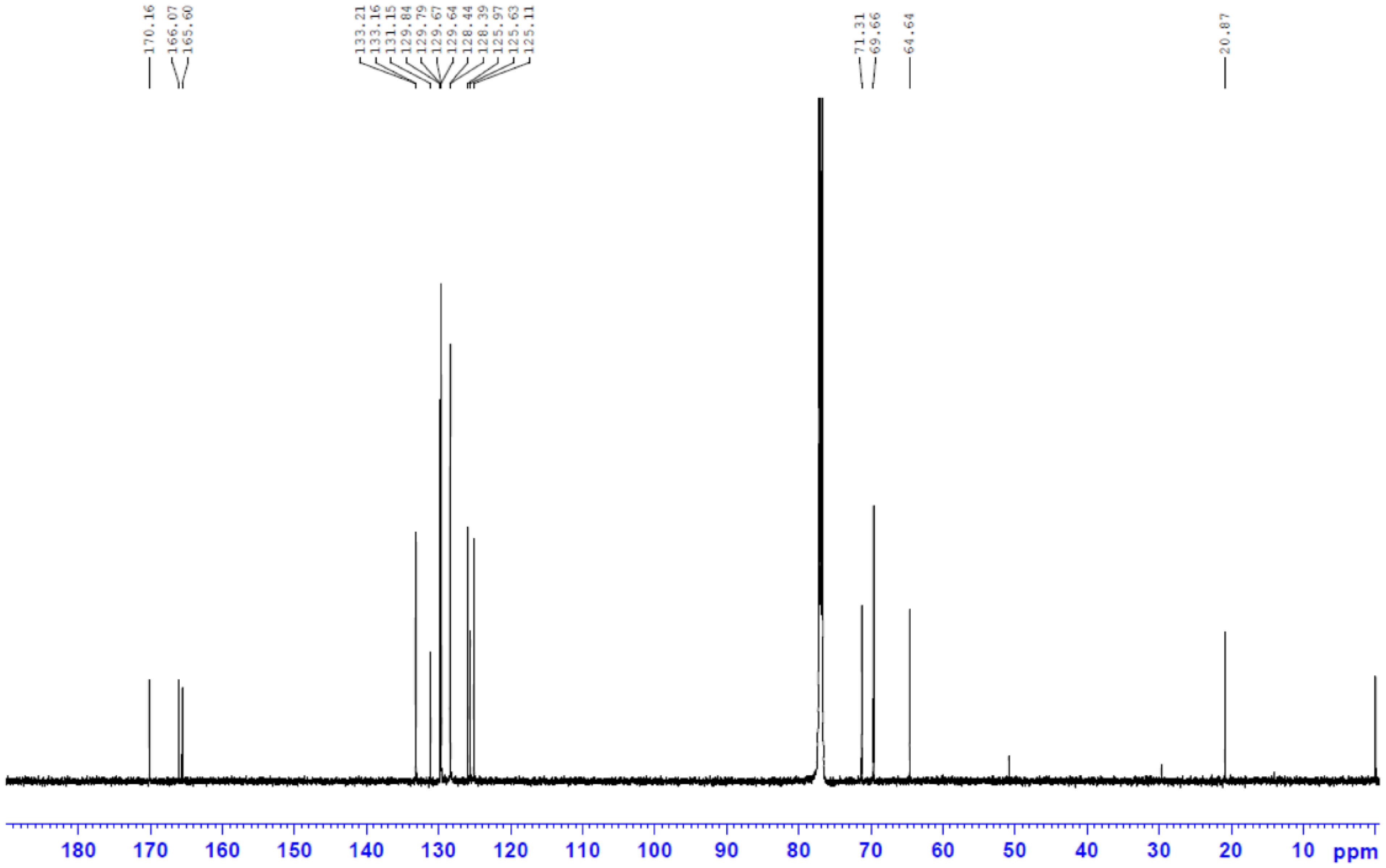

Figure S170. ^{13}C NMR (150 MHz) spectrum of compound **20** in $CDCl_3$

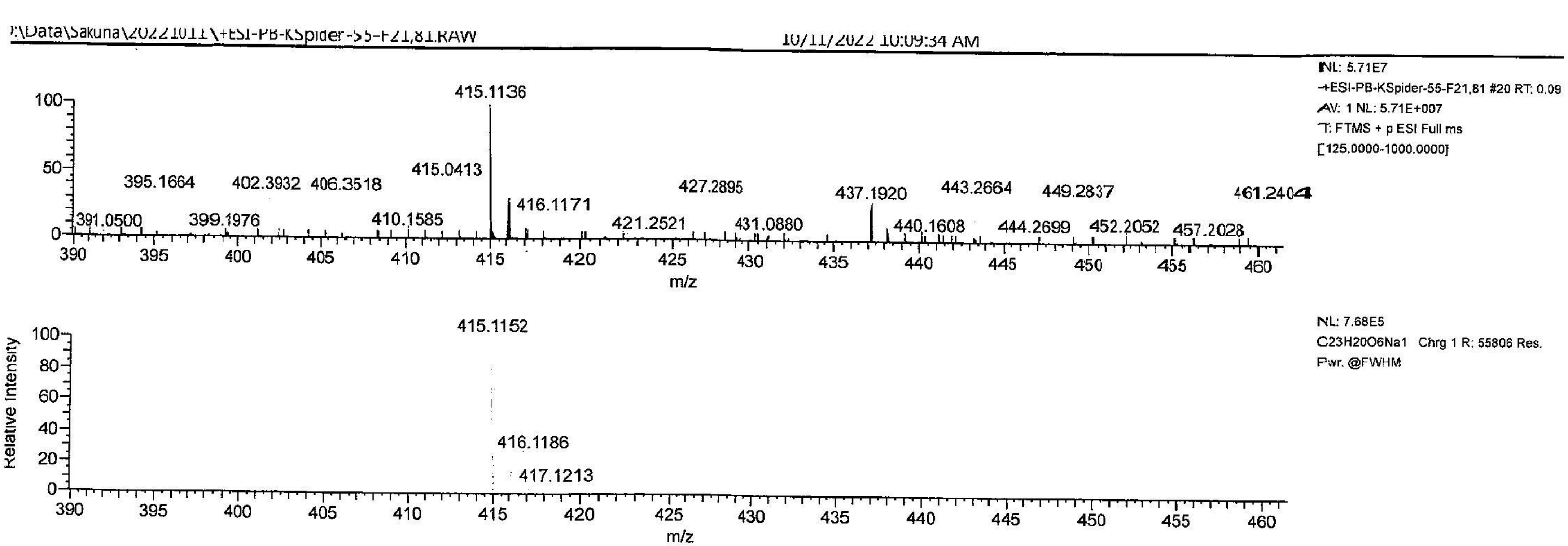

Peak Mass	Display Formula	Delta [ppm]	Theo. mass	MSMS Matched Fragments
415.1136	$C_{23}H_{20}O_6{}^{23}Na$ [M+Na]+	-3.84	415.11521	(Collection)
415.1136	$C_7H_{27}O_{19}$	-1.18	415.11410	(Collection)
415.1136	$C_{32}H_{15}O$	4.52	415.11174	(Collection)
415.1136	$C_5H_{26}O_{19}{}^{23}Na$	4.62	415.11170	(Collection)

Figure S171. HRESIMS spectrum of compound **20**

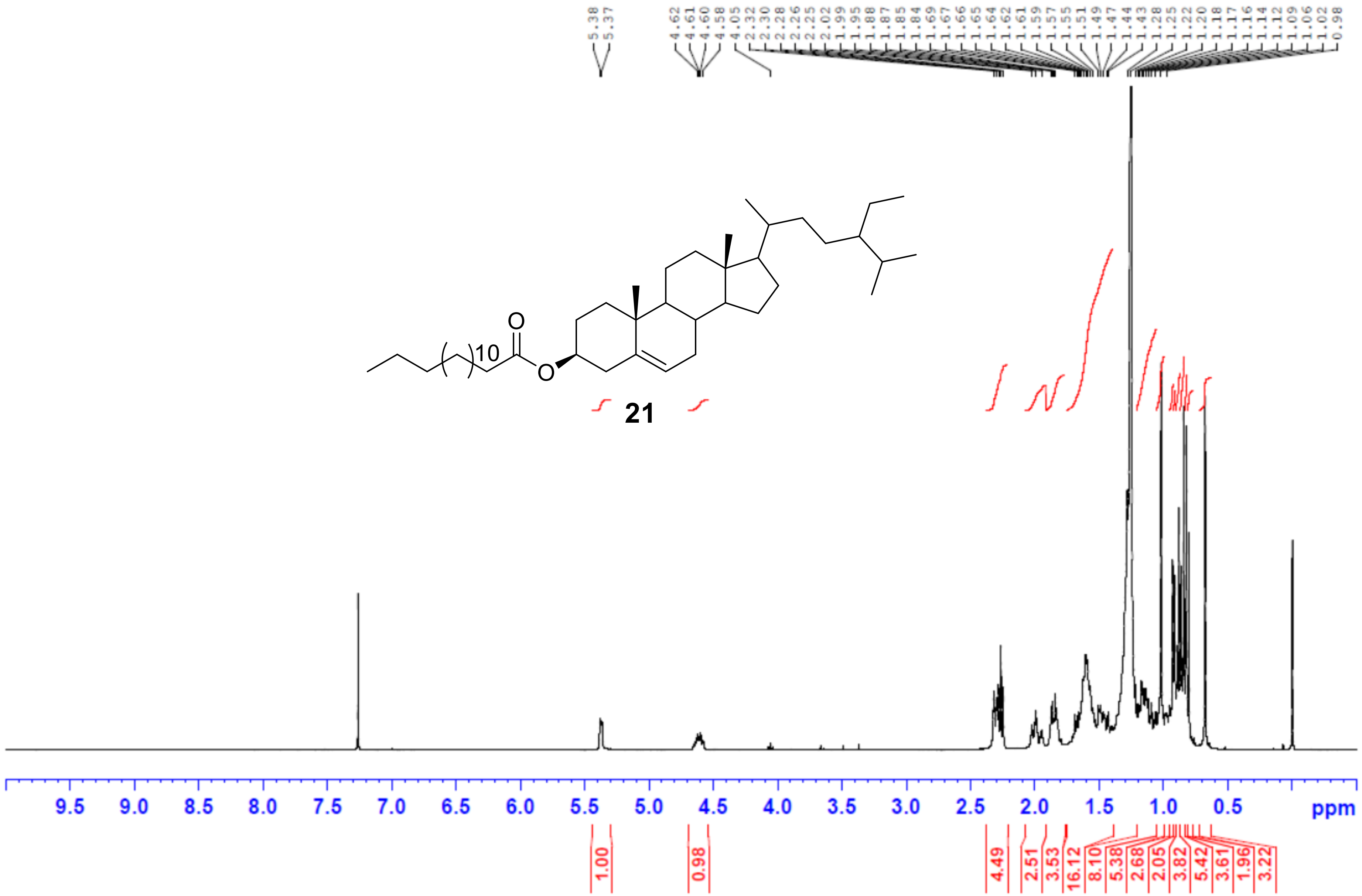

Figure S172. ^{1}H NMR (600 MHz) spectrum of compound **21** in $CDCl_3$

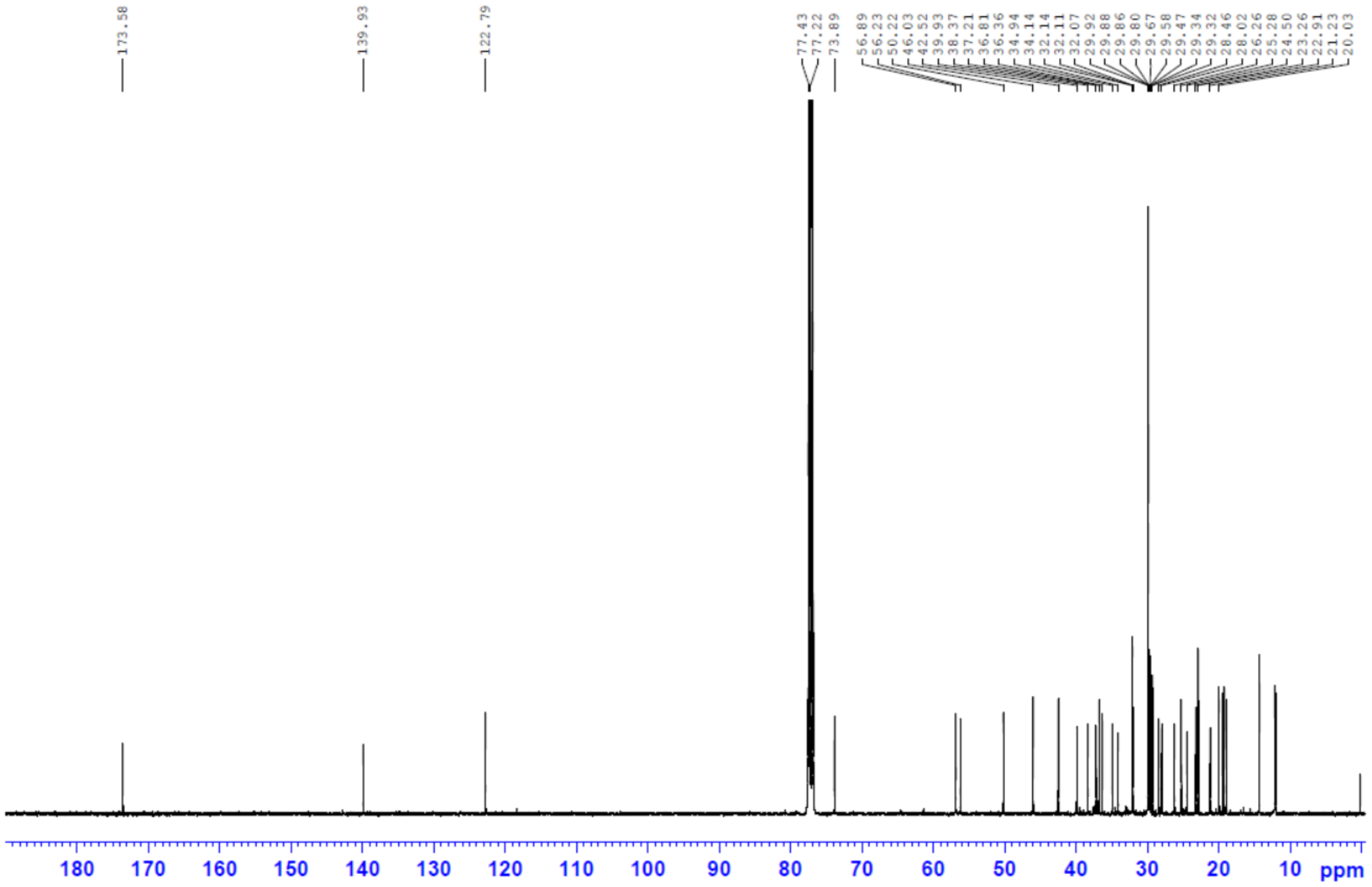

Figure S173. ^{13}C NMR (150 MHz) spectrum of compound **21** in $CDCl_3$

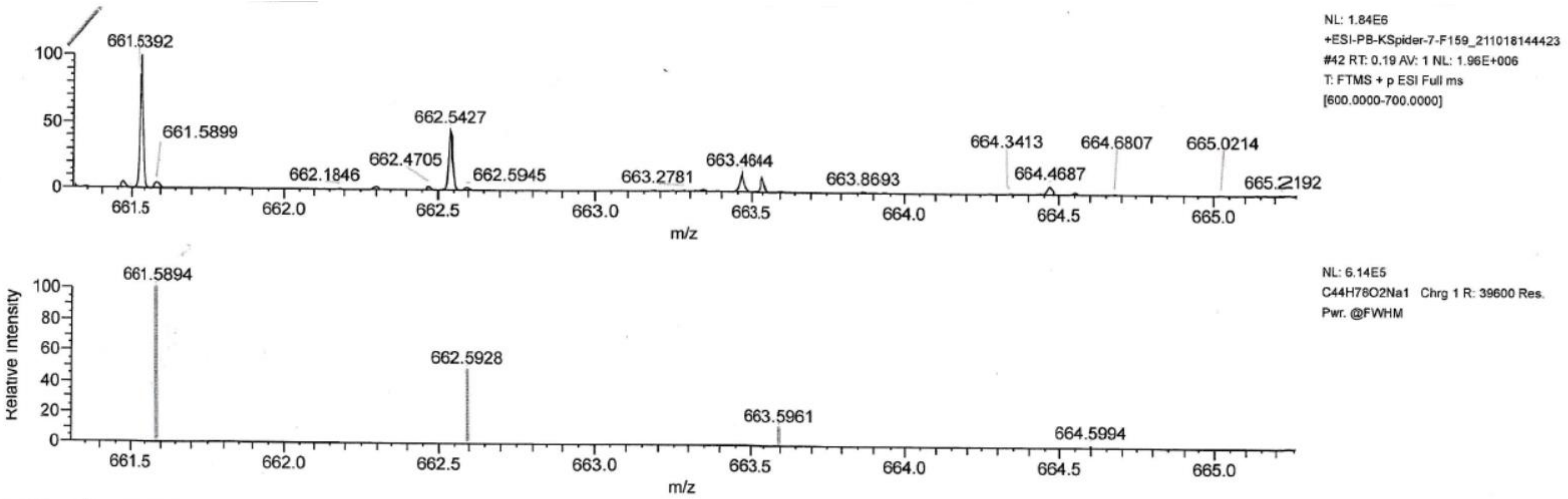

Peak Mass	Display Formula	Delta [ppm]	Theo. mass	MSMS Matched Fragments
661.5899	$C_{44}H_{78}O_2{}^{23}Na$ [M+Na]+	0.76	661.58940	(Collection)
661.5899	$C_{28}H_{85}O_{15}$	2.43	661.58830	(Collection)
661.5899	$C_{46}H_{77}O_2$	-2.88	661.59181	(Collection)
661.5899	$C_{19}H_{90}O_{20}{}^{23}Na$	-2.81	661.59177	(Collection)
661.5899	$CH_{98}O_{33}{}^{23}Na$	2.49	661.58826	(Collection)
661.5899	$C_3H_{97}O_{33}$	-1.14	661.59066	(Collection)

Figure S174. HRESIMS spectrum of compound **21**

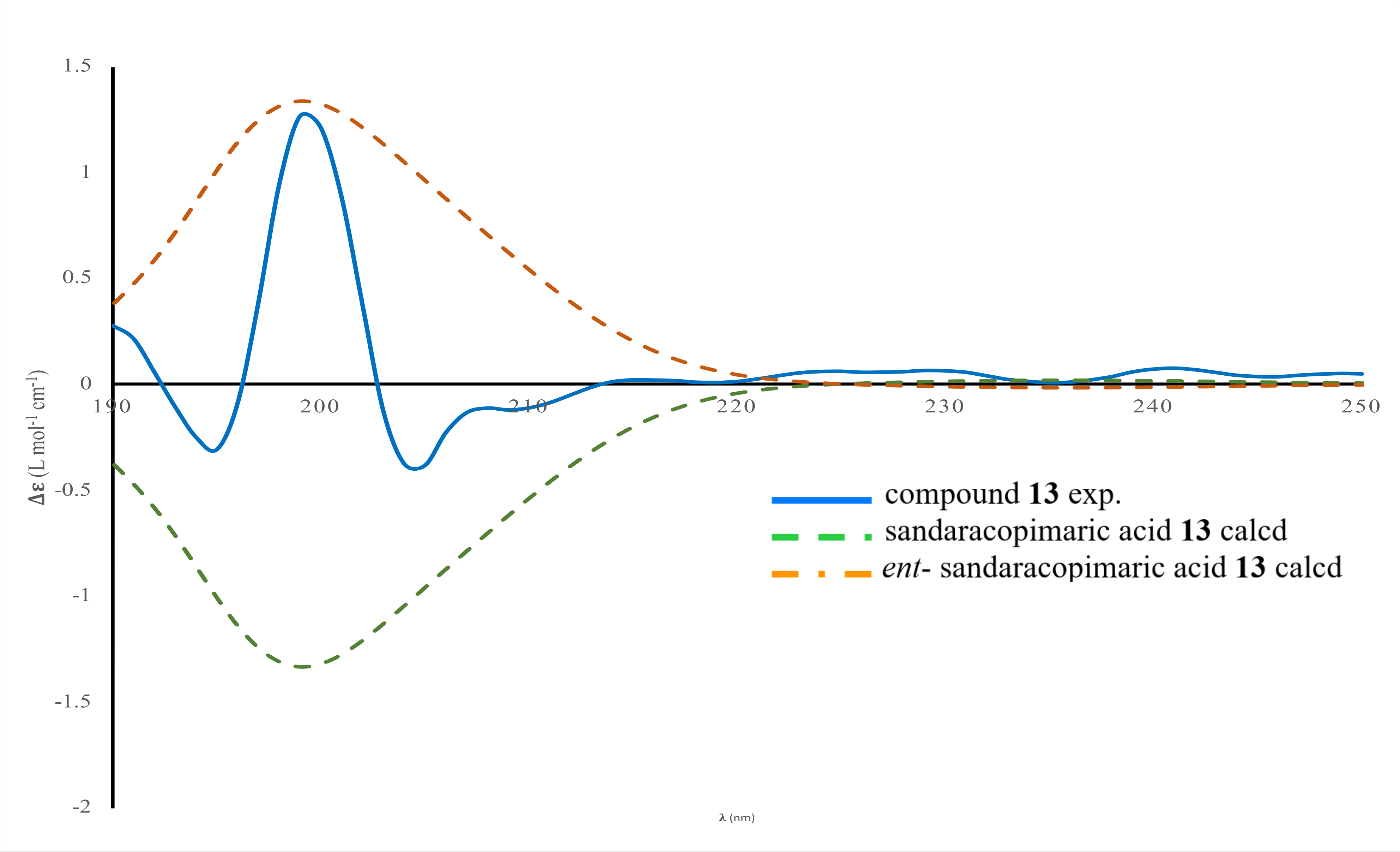

Figure S175. Simulated and calculated ECD spectra of compound **13**

Table S4 Conformational analysis of **2**

Conformer of **2**	Gibbs Free Energy (Hartree)	Gibbs Free Energy (kcal/mol)	Relative Gibbs Free Energy (kcal/mol)	Population (%)
**I**	-2152.789291	-1350875.280103	0.508275	8.3
**II**	-2152.789354	-1350875.319635	0.468743	8.8
**III**	-2152.789772	-1350875.581930	0.206448	13.7
**IV**	-2152.787354	-1350874.064635	1.723743	1.1
**V**	-2152.789014	-1350875.106285	0.682093	6.2
**VI**	-2152.789597	-1350875.472118	0.316260	11.4
**VII**	-2152.789315	-1350875.295163	0.493215	8.5
**VIII**	-2152.788403	-1350874.722883	1.065495	3.3
**IX**	-2152.788809	-1350874.977648	0.810730	5.0
**X**	-2152.787616	-1350874.229040	1.559338	1.4
**XI**	-2152.790101	-1350875.788378	0.000000	19.3
**XII**	-2152.787459	-1350874.130523	1.657855	1.2
**XIII**	-2152.78965	-1350875.505375	0.283002	12.0

Table S5 Conformational analysis of **2-dias**

Conformer of **2-dias**	Gibbs Free Energy (Hartree)	Gibbs Free Energy (kcal/mol)	Relative Gibbs Free Energy (kcal/mol)	Population (%)
**I**	-2152.790561	-1350876.077028	0.841478	14.4
**II**	-2152.789235	-1350875.244963	1.673542	3.6
**III**	-2152.788096	-1350874.530240	2.388265	1.1
**IV**	-2152.788142	-1350874.559105	2.359400	1.1
**V**	-2152.788463	-1350874.760533	2.157972	1.6
**VI**	-2152.791902	-1350876.918505	0.000000	58.8
**VII**	-2152.788911	-1350875.041653	1.876852	2.6
**VIII**	-2152.788468	-1350874.763670	2.154835	1.6
**IX**	-2152.790615	-1350876.110913	0.807593	15.2

Table S6 Conformational analysis of **3**

Conformer of **3**	Gibbs Free Energy (Hartree)	Gibbs Free Energy (kcal/mol)	Relative Gibbs Free Energy (kcal/mol)	Population (%)
**I**	-1846.463518	-1158655.857545	0.031375	35.0
**II**	-1846.463311	-1158655.727653	0.161267	28.2
**III**	-1846.463568	-1158655.888920	0.000000	36.9

Table S7 Conformational analysis of **3-dias**

Conformer of **3**	Gibbs Free Energy (Hartree)	Gibbs Free Energy (kcal/mol)	Relative Gibbs Free Energy (kcal/mol)	Population (%)
**I**	-1846.463518	-1158655.857545	0.031375	35.0
**II**	-1846.463311	-1158655.727653	0.161267	28.2
**III**	-1846.463568	-1158655.888920	0.000000	36.9

Table S8 Conformational analysis of **6**

Conformer of **6**	Gibbs Free Energy (Hartree)	Gibbs Free Energy (kcal/mol)	Relative Gibbs Free Energy (kcal/mol)	Population (%)
**I**	-1999.054576	-1254406.746440	0.044553	30.6
**II**	-1999.054605	-1254406.764638	0.026355	31.5
**III**	-1999.054647	-1254406.790993	0.000000	32.9
**IV**	-1999.052844	-1254405.659610	1.131383	5.0

Table S9 Conformational analysis of **6-dias**

Conformer of **6**	Gibbs Free Energy (Hartree)	Gibbs Free Energy (kcal/mol)	Relative Gibbs Free Energy (kcal/mol)	Population (%)
**I**	-1999.052724	-1254405.584310	0.539023	9.5
**II**	-1999.052733	-1254405.589958	0.533375	9.6
**III**	-1999.053258	-1254405.919395	0.203938	16.7
**IV**	-1999.051868	-1254405.047170	1.076163	3.9
**V**	-1999.052484	-1254405.433710	0.689622	7.4
**VI**	-1999.052136	-1254405.215340	0.907992	5.1
**VII**	-1999.053031	-1254405.776953	0.346380	13.1
**VIII**	-1999.051879	-1254405.054073	1.069260	3.9
**IX**	-1999.053583	-1254406.123333	0.000000	23.4
**X**	-1999.052467	-1254405.423043	0.700290	7.3

Table S10 Conformational analysis of **8**

Conformer of **8**	Gibbs Free Energy (Hartree)	Gibbs Free Energy (kcal/mol)	Relative Gibbs Free Energy (kcal/mol)	Population (%)
**I**	-2151.647507	-1350158.810643	0.454310	16.6
**II**	-2151.644928	-1350157.192320	2.072632	1.1
**III**	-2151.645138	-1350157.324095	1.940858	1.4
**IV**	-2151.647193	-1350158.613608	0.651345	11.9
**V**	-2151.648231	-1350159.264953	0.000000	35.4
**VI**	-2151.64756	-1350158.843900	0.421053	17.5
**VII**	-2151.647486	-1350158.797465	0.467488	16.2

Table S11 Conformational analysis of **8-dias**

Conformer of **8-dias**	Gibbs Free Energy (Hartree)	Gibbs Free Energy (kcal/mol)	Relative Gibbs Free Energy (kcal/mol)	Population (%)
**I**	-2151.647507	-1350158.810643	0.454310	16.6
**II**	-2151.644928	-1350157.192320	2.072632	1.1
**III**	-2151.645138	-1350157.324095	1.940858	1.4
**IV**	-2151.647193	-1350158.613608	0.651345	11.9
**V**	-2151.648231	-1350159.264953	0.000000	35.4
**VI**	-2151.64756	-1350158.843900	0.421053	17.5
**VII**	-2151.647486	-1350158.797465	0.467488	16.2

Table S12 Coordinates of compound **2**

	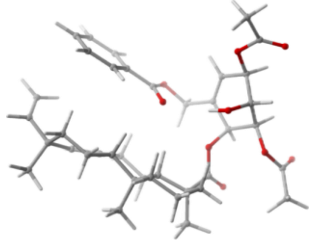 conformer **2-I**			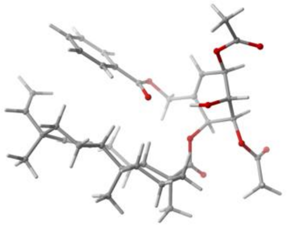 conformer **2-II**			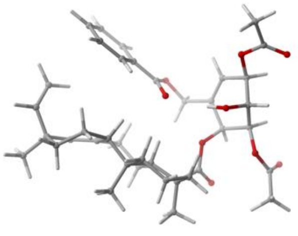 conformer **2-III**		
C	-2.246260	-3.785333	0.938578	-2.246688	-3.785408	0.938601	-2.655282	-3.367461	1.109741
C	-1.528067	-2.425686	1.021637	-1.528461	-2.425767	1.021704	-1.836262	-2.069566	1.057345
C	-0.478437	-2.216323	-0.106656	-0.478749	-2.216414	-0.106507	-0.734040	-2.048554	-0.045621
C	-1.118555	-2.387051	-1.485627	-1.118749	-2.387185	-1.485523	-1.321099	-2.234126	-1.447938
C	-2.222902	-1.350567	-1.689782	-2.223081	-1.350729	-1.689808	-2.313948	-1.113531	-1.766257
C	-3.180908	-1.234276	-0.526251	-3.181146	-1.234324	-0.526328	-3.340298	-0.959007	-0.674080
C	-4.483488	-1.020116	-0.745272	-4.483676	-1.019993	-0.745479	-4.647434	-0.908188	-0.952270
C	-5.518119	-0.725266	0.315424	-5.518416	-0.725020	0.315078	-5.755323	-0.762179	0.061554
C	-6.405058	0.435726	-0.097674	-6.404937	0.436291	-0.098029	-6.272544	0.665490	0.114236
C	-6.111417	1.374078	-0.997008	-6.110751	1.374816	-0.996998	-5.688457	1.747477	-0.399118
C	-6.408080	-1.965498	0.526169	-6.408842	-1.964987	0.525418	-6.924135	-1.682197	-0.329271
C	-4.797140	-0.324468	1.621244	-4.797577	-0.324671	1.621107	-5.228844	-1.166775	1.452528
C	-3.647428	-1.266288	1.957002	-3.648088	-1.266812	1.956764	-3.876613	-0.528874	1.757449
C	-2.570941	-1.266086	0.864419	-2.571371	-1.266248	0.864433	-2.777100	-0.853072	0.728925
H	-1.986248	-0.332864	0.962823	-1.986814	-0.332969	0.963103	-2.094942	0.013888	0.713130
H	-0.205735	-1.155927	-0.016586	-0.206069	-1.156007	-0.016471	-0.355440	-1.018239	-0.004300
C	0.884204	-2.970010	0.024782	0.883876	-2.970113	0.025081	0.541846	-2.919503	0.192495
C	0.860683	-4.452007	-0.365063	0.860388	-4.452125	-0.364723	0.372077	-4.417959	-0.077146
C	1.871437	-2.266919	-0.911041	1.871108	-2.267072	-0.910759	1.610474	-2.397548	-0.772888
O	1.870427	-0.936616	-0.686044	1.870514	-0.936783	-0.685454	1.769581	-1.066583	-0.621832
C	2.814769	-0.104561	-1.365323	2.814649	-0.104809	-1.365091	2.789186	-0.384720	-1.355960
H	3.105370	-0.602040	-2.304026	3.104951	-0.602358	-2.303849	2.997168	-0.954992	-2.275179
C	2.102546	1.203870	-1.621168	2.102392	1.203633	-1.620760	2.231197	0.987351	-1.661680
C	0.850652	1.089662	-2.453892	0.850395	1.089395	-2.453320	0.947427	0.991532	-2.453372
O	-0.190135	2.000716	-2.082277	-0.190111	2.000842	-2.081905	0.010670	2.002526	-2.055407
C	-0.710592	1.876951	-0.846667	-0.710906	1.876971	-0.846427	-0.454514	1.960305	-0.793636
C	-1.928327	2.709056	-0.657357	-1.928326	2.709537	-0.657146	-1.580266	2.905076	-0.565717
C	-2.517783	2.723256	0.610791	-2.498983	3.441561	-1.703654	-2.108876	2.984900	0.727250
C	-3.672767	3.465113	0.831682	-3.654011	4.184792	-1.477076	-3.170994	3.842171	0.991689
C	-4.240831	4.195640	-0.211814	-4.240269	4.197004	-0.211686	-3.705981	4.623144	-0.033043
C	-3.654775	4.183415	-1.477300	-3.672707	3.466005	0.831750	-3.185406	4.540048	-1.324250
C	-2.499474	3.440622	-1.703920	-2.518001	2.723705	0.610900	-2.123162	3.681593	-1.594490
O	-0.247871	1.154640	0.009771	-0.248630	1.154244	0.009889	-0.017325	1.221968	0.062835
C	2.489135	2.349911	-1.058288	2.489032	2.349638	-1.057845	2.770299	2.104462	-1.170206
C	3.586383	2.439540	-0.036637	3.586690	2.439264	-0.036634	3.897373	2.110447	-0.177668
H	4.542528	2.760799	-0.481909	4.542628	2.760519	-0.482357	4.873528	2.288584	-0.658365
O	3.162248	3.434744	0.899789	3.163007	3.434464	0.899995	3.621950	3.196455	0.712233
C	4.117082	3.940697	1.708382	4.118231	3.940431	1.708142	4.651550	3.621325	1.474423
C	3.519086	4.902431	2.695605	3.520686	4.902318	2.695484	4.200176	4.697532	2.420597
O	5.281701	3.638006	1.632150	5.282807	3.637723	1.631380	5.768486	3.174852	1.391897
C	3.783721	1.076004	0.651611	3.784360	1.075739	0.651523	3.943743	0.769214	0.577869
H	4.671935	1.124201	1.294422	4.672825	1.123968	1.293984	4.846965	0.739855	1.200649
O	2.709214	0.740990	1.490030	2.710185	0.740711	1.490375	2.857480	0.611410	1.451749
C	4.050050	0.085083	-0.477571	4.050240	0.084821	-0.477759	4.060470	-0.301552	-0.502756
H	4.867288	0.467914	-1.107518	4.867271	0.467612	-1.107998	4.901103	-0.051606	-1.167694
O	4.438393	-1.168779	0.067968	4.438723	-1.169067	0.067640	4.311431	-1.564032	0.099868
C	5.289722	-1.909174	-0.688930	5.289706	-1.909508	-0.689561	5.054104	-2.435015	-0.631967
C	5.467021	-3.275850	-0.098492	5.467061	-3.276258	-0.099302	5.088162	-3.781871	0.025718
O	5.797439	-1.502509	-1.702066	5.797113	-1.502864	-1.702868	5.581594	-2.140921	-1.673626
O	2.576232	-2.793277	-1.738645	2.575581	-2.793418	-1.738630	2.241633	-3.050431	-1.569194
C	1.450229	-2.798449	1.457660	1.449788	-2.798599	1.458004	1.089768	-2.678444	1.622231

C	0.418469	-3.147733	2.524678	0.417918	-3.147834	2.524920	0.004362	-2.797826	2.686153
C	-0.822541	-2.271183	2.383187	-0.823045	-2.271242	2.383305	-1.142740	-1.827290	2.412916
H	-1.551835	-4.617778	0.773769	-2.788872	-4.001497	1.871261	-3.340507	-3.346411	1.971095
H	-2.983227	-3.803983	0.121156	-1.552347	-4.617833	0.773345	-2.027817	-4.259531	1.224014
H	-2.788736	-4.001227	1.871106	-2.983922	-3.803852	0.121415	-3.267704	-3.502283	0.206151
H	-1.528892	-3.402200	-1.602371	-1.529076	-3.402333	-1.602284	-1.827462	-3.208522	-1.536747
H	-0.360200	-2.275394	-2.280312	-0.360320	-2.275548	-2.280139	-0.516052	-2.242089	-2.203405
H	-2.781099	-1.546265	-2.617787	-2.781258	-1.546564	-2.617794	-2.806646	-1.288169	-2.734453
H	-1.744943	-0.363119	-1.820795	-1.745165	-0.363279	-1.820959	-1.757196	-0.163564	-1.859977
H	-4.849892	-1.027568	-1.778622	-4.849961	-1.027333	-1.778871	-4.962766	-0.954738	-2.001584
H	-7.355847	0.502962	0.446274	-7.355917	0.503614	0.445571	-7.221859	0.781977	0.653993
H	-5.166644	1.370106	-1.547898	-5.165770	1.370815	-1.547535	-4.737091	1.689575	-0.935485
H	-6.803036	2.194764	-1.200543	-6.802096	2.195715	-1.200591	-6.142998	2.734842	-0.291133
H	-5.815468	-2.826787	0.867610	-6.903463	-2.251347	-0.415850	-6.588269	-2.728979	-0.391643
H	-6.902663	-2.252320	-0.414978	-7.192751	-1.764224	1.274352	-7.342687	-1.395675	-1.306912
H	-7.192002	-1.764855	1.275126	-5.816629	-2.826623	0.866670	-7.733149	-1.626255	0.416774
H	-5.521977	-0.288355	2.451316	-5.522527	-0.288591	2.451072	-5.155752	-2.264782	1.485373
H	-4.403025	0.696378	1.491333	-4.403232	0.696119	1.491471	-5.962352	-0.875899	2.223255
H	-4.042340	-2.283663	2.102177	-4.043179	-2.284212	2.101329	-3.554222	-0.804982	2.771291
H	-3.201462	-0.967517	2.917229	-3.202323	-0.968569	2.917240	-4.014379	0.564995	1.768824
H	1.887405	-4.837610	-0.429846	0.323768	-5.052566	0.380004	1.352001	-4.915094	-0.065110
H	0.391553	-4.615836	-1.344771	1.887119	-4.837691	-0.429582	-0.077593	-4.612649	-1.060326
H	0.323958	-5.052459	0.379576	0.391171	-4.616047	-1.344371	-0.254180	-4.892382	0.687893
H	0.461716	0.063424	-2.377813	0.461231	0.063278	-2.376763	0.460901	0.009723	-2.359189
H	1.040172	1.313649	-3.512026	1.039895	1.312901	-3.511561	1.121592	1.193948	-3.518243
H	-2.057262	2.143792	1.411700	-2.036214	3.421110	-2.689736	-1.673755	2.366460	1.512995
H	-4.137350	3.468977	1.818657	-4.102223	4.753735	-2.293111	-3.586240	3.899751	1.998704
H	-5.150739	4.772963	-0.038788	-5.149942	4.774701	-0.038681	-4.540131	5.295985	0.174563
H	-4.103346	4.752026	-2.293374	-4.137460	3.469859	1.818642	-3.610776	5.146171	-2.125527
H	-2.036880	3.420157	-2.690087	-2.057886	2.143831	1.411743	-1.710941	3.607836	-2.600267
H	1.931205	3.269858	-1.250689	1.930988	3.269552	-1.250036	2.320874	3.074712	-1.397173
H	2.867898	4.343815	3.383620	2.898034	5.643400	2.176276	5.067342	5.115466	2.942255
H	2.895179	5.642535	2.176524	4.320152	5.395314	3.258143	3.501080	4.259239	3.147641
H	4.318393	5.396580	3.257493	2.868266	4.344150	3.382686	3.660712	5.483171	1.874461
H	1.887051	0.749364	0.975261	1.887907	0.748713	0.975807	2.028979	0.691030	0.952884
H	6.399384	-3.718669	-0.465348	6.399617	-3.718852	-0.465937	4.173779	-4.313405	-0.279390
H	4.619242	-3.888623	-0.441444	4.619511	-3.889103	-0.442697	5.089249	-3.688291	1.118679
H	5.448714	-3.237924	0.997707	5.448369	-3.238574	0.996894	5.961126	-4.342499	-0.326032
H	1.775496	-1.756250	1.599439	1.775161	-1.756447	1.599847	1.534861	-1.673731	1.677055
H	2.350684	-3.425422	1.563491	2.350187	-3.425646	1.563881	1.906763	-3.392449	1.816963
H	0.864434	-3.000817	3.520474	0.863802	-3.000949	3.520751	0.443243	-2.581246	3.672439
H	0.141096	-4.213768	2.466980	0.140498	-4.213854	2.467187	-0.375858	-3.831466	2.742196
H	-0.518994	-1.215241	2.502603	-0.519474	-1.215306	2.502706	-0.743202	-0.797158	2.423265
H	-1.529850	-2.492364	3.197542	-1.530422	-2.492363	3.197609	-1.876679	-1.892923	3.230720

	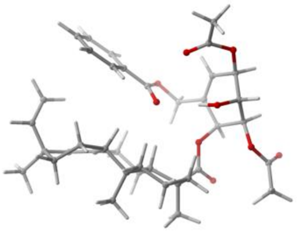 conformer **2-IV**			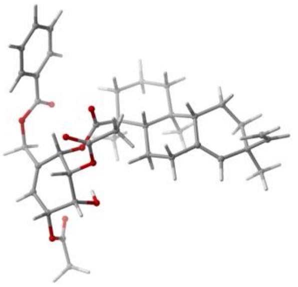 conformer **2-V**			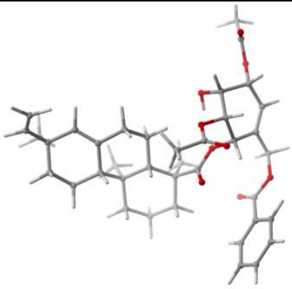 conformer **2-VI**		
C	-2.183901	-3.671017	0.986290	3.353449	0.349415	-2.316419	-3.403951	-0.661170	-2.364825
C	-1.497695	-2.297309	0.980540	2.916228	1.157199	-1.084334	-2.978972	-1.298001	-1.027555
C	-0.377077	-2.139439	-0.092046	1.605649	0.627851	-0.419622	-1.640952	-0.712747	-0.483583
C	-0.906735	-2.364237	-1.511117	1.750253	-0.818922	0.061614	-1.736093	0.804973	-0.309426
C	-2.001603	-1.346239	-1.839102	2.878000	-0.925993	1.089121	-2.860171	1.157669	0.664061
C	-3.062599	-1.302360	-0.769282	4.158900	-0.333998	0.565868	-4.169465	0.481619	0.344668
C	-4.362067	-1.347737	-1.082683	5.310031	-1.015033	0.588571	-5.326427	1.152644	0.372030
C	-5.502610	-1.307160	-0.095921	6.637491	-0.517521	0.069818	-6.692074	0.531291	0.195476
C	-6.113277	0.081900	-0.010083	7.541047	-0.053493	1.199525	-7.703930	1.111744	1.165138
C	-5.579199	1.220159	-0.451586	7.193865	0.153075	2.469116	-7.452466	1.837061	2.254154
C	-6.597647	-2.288566	-0.545217	7.350535	-1.659371	-0.674673	-7.200738	0.793135	-1.236798
C	-4.981097	-1.721983	1.293823	6.398191	0.654190	-0.902178	-6.582290	-0.982124	0.485485
C	-3.695886	-0.986134	1.660259	5.405675	1.669000	-0.341063	-5.435900	-1.633572	-0.278599
C	-2.547223	-1.173824	0.650760	4.030518	1.075149	0.021873	-4.074063	-1.005242	0.057148
H	-1.955863	-0.242824	0.668758	3.633632	1.685282	0.855179	-3.705025	-1.478339	0.988799
H	-0.117162	-1.075412	-0.028564	1.494215	1.249793	0.488693	-1.542913	-1.146119	0.529483
C	0.981986	-2.865046	0.164939	0.268174	0.888827	-1.195206	-0.325816	-1.175673	-1.204026
C	0.988594	-4.366274	-0.138457	0.014752	-0.012680	-2.411796	-0.031853	-0.510405	-2.556735
C	2.011706	-2.202733	-0.755769	-0.822858	0.677493	-0.141588	0.783821	-0.848925	-0.201679
O	2.007321	-0.865073	-0.583398	-1.524720	-0.462440	-0.324259	1.522737	0.228913	-0.557889
C	2.970939	-0.062362	-1.266390	-2.590504	-0.749480	0.605094	2.599875	0.624367	0.320438
H	3.276782	-0.594456	-2.181307	-2.905666	0.196026	1.064023	2.885128	-0.247264	0.922790
C	2.295693	1.250558	-1.593279	-3.699495	-1.358149	-0.208791	3.724093	1.058358	-0.581975
C	1.053509	1.145065	-2.444002	-4.408826	-0.439591	-1.171525	4.378703	-0.023891	-1.404261
O	0.017587	2.055808	-2.053080	-4.522452	0.874900	-0.618515	4.462468	-1.237918	-0.653907
C	-0.491191	1.899012	-0.815337	-3.754560	1.844594	-1.159905	3.658536	-2.256724	-1.028861
C	-1.665256	2.772053	-0.558967	-3.797955	3.101926	-0.368004	3.687180	-3.380187	-0.057055
C	-2.286065	3.504468	-1.574983	-4.455116	3.179421	0.863463	4.384211	-3.295488	1.151921
C	-3.406261	4.278230	-1.282445	-4.433726	4.369812	1.584193	4.350482	-4.362548	2.044674
C	-3.902628	4.323477	0.019994	-3.762219	5.481851	1.076946	3.626441	-5.512680	1.731855
C	-3.283413	3.591307	1.033643	-3.108483	5.405774	-0.153478	2.932551	-5.598536	0.524251
C	-2.165943	2.815886	0.745926	-3.124006	4.217051	-0.874588	2.960838	-4.533353	-0.368899
O	-0.056530	1.106081	-0.010607	-3.098475	1.695649	-2.166340	2.985684	-2.241242	-2.035441
C	2.695178	2.419605	-1.085618	-3.946529	-2.669416	-0.183483	4.048059	2.346557	-0.720998
C	3.786359	2.527236	-0.053912	-3.182722	-3.650918	0.667339	3.337055	3.473145	-0.025100
H	4.744029	2.760802	-0.545384	-3.805590	-3.901902	1.541038	3.942220	3.839400	0.821915
O	3.641835	3.613422	0.870418	-2.988309	-4.928569	0.049040	3.225084	4.524447	-0.988474
C	2.446453	3.909403	1.428625	-2.522824	-4.993939	-1.219582	2.975856	5.758248	-0.500367
C	2.638400	4.869692	2.569227	-2.171516	-6.412955	-1.567301	2.780301	6.741072	-1.619261
O	1.384228	3.478495	1.058992	-2.413886	-4.048698	-1.959296	2.914325	6.006298	0.677930
C	3.937028	1.198987	0.705237	-1.838698	-3.102155	1.189753	1.948252	3.064073	0.511148
H	4.820605	1.259352	1.357001	-1.512759	-3.728149	2.032906	1.612345	3.830437	1.221219
O	2.852011	0.933236	1.552087	-0.802300	-3.180529	0.250747	0.972849	3.017639	-0.497889
C	4.194027	0.140835	-0.363899	-2.113573	-1.694449	1.705879	2.149140	1.749332	1.252076
H	5.033304	0.466938	-0.996820	-2.915526	-1.737577	2.459747	2.939792	1.880115	2.006990
O	4.540773	-1.089629	0.256264	-0.934652	-1.183788	2.313472	0.941338	1.381859	1.905862
C	5.421542	-1.872473	-0.420705	-1.125291	-0.233314	3.266279	1.081992	0.593557	3.004684
C	5.535119	-3.219311	0.227531	0.180142	0.382993	3.666029	-0.253325	0.114645	3.486363
O	5.999747	-1.511608	-1.413291	-2.212545	0.057090	3.695196	2.151914	0.325870	3.487698
O	2.745387	-2.764982	-1.534260	-1.041257	1.431692	0.777637	0.990019	-1.461457	0.818351
C	1.462178	-2.596329	1.614055	0.191928	2.376178	-1.606200	-0.331542	-2.712536	-1.363403

C	0.370056	-2.863990	2.643096	1.445915	2.846648	-2.332198	-1.612904	-3.228095	-2.007117
C	-0.868123	-2.016288	2.359785	2.692141	2.631101	-1.477316	-2.832914	-2.822998	-1.185995
H	-2.748064	-3.851033	0.059423	2.563635	0.266761	-3.070987	-3.771591	0.365985	-2.225483
H	-2.898415	-3.732087	1.821814	3.666399	-0.669584	-2.046913	-4.215411	-1.238112	-2.832918
H	-1.474473	-4.498398	1.111703	4.210026	0.840692	-2.802480	-2.583886	-0.621639	-3.090044
H	-1.304940	-3.384983	-1.627469	1.963761	-1.488918	-0.786745	-1.921857	1.290731	-1.280376
H	-0.087043	-2.273650	-2.245261	0.818131	-1.183332	0.514510	-0.794897	1.223994	0.071269
H	-2.455081	-1.558588	-2.818940	3.027724	-1.971800	1.395403	-2.994000	2.247687	0.726355
H	-1.539097	-0.345934	-1.911702	2.573808	-0.363566	1.990881	-2.550636	0.818548	1.670108
H	-4.645684	-1.396699	-2.140903	5.314659	-2.018494	1.030944	-5.301020	2.234143	0.547624
H	-7.088427	0.115812	0.494113	8.579899	0.134905	0.897145	-8.746328	0.858696	0.932201
H	-4.605333	1.246828	-0.948872	6.170579	-0.016988	2.814104	-6.435054	2.107090	2.548199
H	-6.099029	2.171711	-0.319951	7.924519	0.500729	3.202870	-8.264218	2.180364	2.899075
H	-7.426770	-2.309996	0.180336	7.564770	-2.499982	0.003871	-6.518755	0.367767	-1.987222
H	-6.191753	-3.308579	-0.630881	8.306261	-1.313187	-1.100129	-7.273493	1.875037	-1.427572
H	-7.010208	-1.998761	-1.524514	6.724120	-2.033145	-1.499578	-8.200705	0.353161	-1.386541
H	-4.816239	-2.810918	1.286319	6.034803	0.240373	-1.854875	-7.534652	-1.480253	0.239228
H	-5.756021	-1.523841	2.053480	7.357078	1.153456	-1.121044	-6.423915	-1.110214	1.569177
H	-3.376155	-1.277297	2.670688	5.295522	2.508595	-1.041546	-5.635343	-1.553668	-1.357597
H	-3.926096	0.090509	1.713000	5.842699	2.095587	0.576163	-5.412087	-2.709906	-0.052665
H	0.609549	-4.584512	-1.146157	0.639570	0.289807	-3.260692	1.006830	-0.717874	-2.848403
H	0.379380	-4.920895	0.585527	-1.032735	0.081269	-2.728688	-0.175928	0.578150	-2.535275
H	2.014135	-4.758002	-0.086080	0.217248	-1.071885	-2.204135	-0.677668	-0.919950	-3.343094
H	0.662671	0.118050	-2.389822	-5.427381	-0.792148	-1.379358	5.402568	0.256935	-1.682984
H	1.245936	1.392659	-3.496188	-3.847165	-0.377099	-2.114080	3.793568	-0.216726	-2.314256
H	-1.890557	3.459843	-2.589337	-4.971234	2.303922	1.255497	4.941577	-2.390701	1.391114
H	-3.895710	4.846906	-2.074683	-4.939002	4.428385	2.549360	4.887510	-4.294234	2.991825
H	-4.781741	4.930086	0.246039	-3.745612	6.413471	1.645796	3.600198	-6.346992	2.435385
H	-3.675356	3.624793	2.051182	-2.582212	6.275984	-0.548890	2.365192	-6.498288	0.281266
H	-1.658053	2.242438	1.521310	-2.614675	4.133277	-1.834763	2.421325	-4.576530	-1.315161
H	2.172794	3.334624	-1.367457	-4.757538	-3.085090	-0.787576	4.869962	2.641039	-1.380305
H	3.056331	4.314510	3.422248	-2.044222	-6.504142	-2.651150	3.600414	6.662099	-2.345495
H	1.671760	5.299512	2.852381	-2.939733	-7.106407	-1.201591	2.720539	7.755336	-1.211286
H	3.353946	5.656939	2.298623	-1.224871	-6.664471	-1.066069	1.846139	6.494300	-2.144657
H	2.026801	0.965092	1.039849	-1.037100	-2.634584	-0.513368	1.108686	2.215610	-1.020097
H	6.480701	-3.690781	-0.061655	0.058634	0.928005	4.608069	-0.577782	-0.689808	2.808792
H	4.699118	-3.828506	-0.149121	0.463739	1.087813	2.869386	-0.995072	0.922639	3.446922
H	5.445485	-3.141840	1.318141	0.963892	-0.380843	3.748843	-0.157846	-0.282845	4.502255
H	1.784642	-1.547813	1.701105	0.057631	2.978422	-0.694331	-0.224609	-3.163874	-0.364978
H	2.351182	-3.215948	1.817323	-0.706142	2.523527	-2.225208	0.554714	-3.006149	-1.946292
H	0.756210	-2.628826	3.647141	1.344890	3.916709	-2.572579	-1.564244	-4.325573	-2.083253
H	0.104534	-3.934267	2.661160	1.554449	2.329667	-3.300091	-1.709751	-2.856908	-3.041138
H	-0.582593	-0.949910	2.404217	2.594919	3.231411	-0.554191	-2.744637	-3.274315	-0.180565
H	-1.610529	-2.181036	3.155588	3.572833	3.017947	-2.012201	-3.745961	-3.239831	-1.638557

	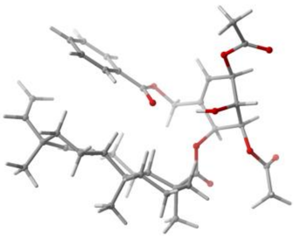 conformer **2-VII**			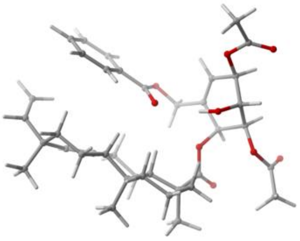 conformer **2-VIII**			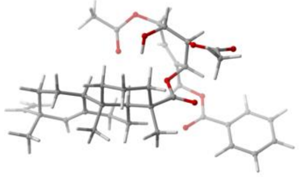 conformer **2-IX**		
C	-2.246558	-3.785362	0.938362	-2.936967	-2.252945	2.261804	-2.017328	-3.950127	0.652319
C	-1.528329	-2.425738	1.021501	-2.506566	-0.875091	1.725971	-1.343170	-2.577091	0.833084
C	-0.478684	-2.216317	-0.106768	-1.114673	-0.899356	1.031161	-0.384781	-2.200184	-0.334825
C	-1.118790	-2.386921	-1.485759	-1.082778	-1.909232	-0.116991	-1.115619	-2.285404	-1.677322
C	-2.223130	-1.350428	-1.689848	-2.119878	-1.520307	-1.170487	-2.316221	-1.336381	-1.706843
C	-3.181137	-1.234187	-0.526306	-3.491396	-1.275162	-0.590739	-3.173753	-1.371768	-0.462214
C	-4.483717	-1.020007	-0.745310	-4.591755	-1.761230	-1.176603	-4.502806	-1.234961	-0.544809
C	-5.518350	-0.725235	0.315408	-6.009184	-1.450657	-0.755082	-5.445855	-1.096767	0.635232
C	-6.405253	0.435817	-0.097615	-6.910559	-1.208679	-1.951115	-6.497545	-0.061872	0.282416
C	-6.111391	1.374440	-0.996591	-6.534463	-0.963548	-3.205749	-6.897140	0.973604	1.021353
C	-6.408396	-1.965436	0.526002	-6.586986	-2.635608	0.044956	-6.189201	-2.433865	0.854940
C	-4.797368	-0.324561	1.621262	-5.998104	-0.156284	0.088371	-4.635065	-0.698873	1.875989
C	-3.647736	-1.266530	1.956910	-4.948207	-0.198173	1.191921	-3.396555	-1.569403	2.052700
C	-2.571196	-1.266141	0.864384	-3.527701	-0.385928	0.639163	-2.436143	-1.456095	0.862307
H	-1.986531	-0.332913	0.962937	-3.157768	0.601945	0.303333	-1.881229	-0.503936	0.963352
H	-0.205955	-1.155930	-0.016642	-1.060189	0.088646	0.553849	-0.165443	-1.136411	-0.168363
C	0.883912	-2.970078	0.024635	0.146666	-0.971571	1.943174	1.018497	-2.889223	-0.367391
C	0.860307	-4.452063	-0.365266	0.484785	-2.364132	2.490115	1.036686	-4.301197	-0.963597
C	1.871220	-2.267037	-0.911143	1.377656	-0.475163	1.174359	1.945606	-2.019493	-1.222276
O	1.870307	-0.936709	-0.686144	1.040270	0.433576	0.233141	1.898514	-0.724725	-0.828915
C	2.814756	-0.104741	-1.365329	2.065367	1.210537	-0.405502	2.896917	0.187818	-1.308638
H	3.105409	-0.602207	-2.304024	3.021745	0.678246	-0.326011	3.260426	-0.174167	-2.282603
C	2.102674	1.203771	-1.621181	1.622456	1.368056	-1.833805	2.214702	1.528946	-1.426641
C	0.850748	1.089685	-2.453880	1.799021	0.175689	-2.735067	1.158023	1.607636	-2.497361
O	-0.189758	2.001055	-2.082273	3.076589	-0.431468	-2.523513	0.107046	2.533297	-2.213442
C	-0.710409	1.877074	-0.846748	3.100158	-1.589543	-1.826980	-0.757507	2.147329	-1.253417
C	-1.927922	2.709457	-0.657338	4.486249	-2.027834	-1.515980	-1.965859	3.007952	-1.179966
C	-2.498719	3.441491	-1.703772	5.607592	-1.315779	-1.952453	-2.033914	4.255287	-1.808939
C	-3.653869	4.184494	-1.477106	6.882559	-1.764923	-1.620364	-3.200737	5.008825	-1.719212
C	-4.240127	4.196467	-0.211706	7.038581	-2.918403	-0.851947	-4.303366	4.509658	-1.024461
C	-3.672397	3.465507	0.831661	5.919038	-3.625606	-0.411636	-4.241636	3.257875	-0.411521
C	-2.517558	2.723430	0.610725	4.643581	-3.181332	-0.742129	-3.070497	2.510740	-0.479942
O	-0.247961	1.154362	0.009505	2.105691	-2.189403	-1.491080	-0.576798	1.171719	-0.559960
C	2.489249	2.349756	-1.058175	0.945909	2.450035	-2.220550	2.484172	2.541159	-0.600333
C	3.586485	2.439280	-0.036504	0.629646	3.620851	-1.327176	3.455192	2.449982	0.540843
H	4.542665	2.760453	-0.481766	1.252088	4.468006	-1.656141	4.427375	2.897258	0.273399
O	3.162436	3.434523	0.899929	-0.700806	4.128482	-1.511041	2.882284	3.212887	1.606633
C	4.117309	3.940403	1.708520	-1.749051	3.279784	-1.444033	3.719182	3.571668	2.602651
C	3.519418	4.902295	2.695654	-3.041900	4.041778	-1.405660	2.969979	4.282924	3.693201
O	5.281911	3.637644	1.632272	-1.655677	2.075941	-1.415230	4.901123	3.332670	2.595668
C	3.783691	1.075724	0.651699	0.903075	3.392931	0.181530	3.680482	0.986756	0.964917
H	4.671855	1.123844	1.294582	1.081991	4.371848	0.649022	4.525094	0.943994	1.663977
O	2.709086	0.740734	1.490042	-0.194981	2.858667	0.869516	2.576405	0.463738	1.658648
C	4.050011	0.084819	-0.477503	2.178561	2.559009	0.302455	4.055583	0.231738	-0.307262
H	4.867285	0.467637	-1.107410	3.019817	3.103630	-0.153857	4.900787	0.743750	-0.792141
O	4.438317	-1.169063	0.068003	2.439919	2.347589	1.684880	4.447302	-1.093247	0.028916
C	5.289873	-1.909329	-0.688755	3.727574	2.058578	2.007122	5.343931	-1.679225	-0.807176
C	5.467195	-3.276004	-0.098318	3.825602	1.596390	3.428869	5.520969	-3.126787	-0.460330
O	5.797804	-1.502532	-1.701732	4.635826	2.144127	1.220551	5.884384	-1.093227	-1.709441
O	2.576028	-2.793440	-1.738700	2.523195	-0.770667	1.413387	2.664094	-2.403681	-2.113183
C	1.449940	-2.798618	1.457528	0.023465	0.050621	3.107946	1.658912	-2.901442	1.046936
C	0.418167	-3.147959	2.524508	-1.311871	-0.059227	3.835210	0.701378	-3.409400	2.117557

C	-0.822814	-2.271357	2.383071	-2.470898	0.158203	2.867580	-0.556824	-2.548167	2.156340
H	-1.552129	-4.617829	0.773676	-3.236596	-2.926612	1.445298	-2.518770	-4.264478	1.580065
H	-2.983431	-3.804014	0.120860	-3.796148	-2.161423	2.943218	-1.300481	-4.739612	0.396047
H	-2.789153	-4.001230	1.870833	-2.137786	-2.752038	2.822609	-2.781572	-3.928801	-0.138678
H	-1.529135	-3.402053	-1.602613	-1.286165	-2.926302	0.254676	-1.443974	-3.318326	-1.872193
H	-0.360410	-2.275184	-2.280410	-0.083692	-1.939420	-0.576888	-0.430121	-2.024051	-2.501951
H	-2.781339	-1.546101	-2.617853	-2.173608	-2.275309	-1.969329	-2.936776	-1.518990	-2.597258
H	-1.745200	-0.362960	-1.820849	-1.789553	-0.573121	-1.634900	-1.926315	-0.310074	-1.792343
H	-4.850131	-1.027368	-1.778661	-4.475834	-2.423131	-2.042502	-4.963777	-1.190438	-1.539227
H	-7.356173	0.502868	0.446128	-7.982645	-1.208264	-1.714884	-6.993427	-0.233867	-0.681930
H	-5.166446	1.370620	-1.547197	-5.481689	-0.934596	-3.498112	-6.460654	1.202239	1.996561
H	-6.802955	2.195179	-1.200097	-7.274484	-0.773750	-3.986364	-7.693422	1.634384	0.671336
H	-7.192164	-1.764878	1.275143	-6.586808	-3.550243	-0.568043	-6.900500	-2.350660	1.691944
H	-5.815861	-2.826912	0.867109	-7.625812	-2.434070	0.355640	-5.480419	-3.247857	1.067212
H	-6.903182	-2.251942	-0.415137	-5.988697	-2.838136	0.945098	-6.755090	-2.717452	-0.046441
H	-5.522194	-0.288429	2.451337	-6.998395	0.018629	0.518549	-5.268634	-0.754280	2.775978
H	-4.403175	0.696256	1.491372	-5.786400	0.688375	-0.588497	-4.318808	0.353327	1.770289
H	-4.042759	-2.283909	2.101780	-5.196806	-1.010855	1.891082	-3.707129	-2.616051	2.194206
H	-3.201811	-0.968065	2.917246	-4.997090	0.732538	1.776833	-2.876583	-1.275438	2.975973
H	0.323532	-5.052519	0.379335	0.475470	-3.124626	1.696914	2.072783	-4.652110	-1.065991
H	1.887005	-4.837733	-0.430052	-0.220172	-2.671706	3.272359	0.583806	-4.335934	-1.963445
H	0.391187	-4.615831	-1.344988	1.492514	-2.350981	2.925857	0.504438	-5.011272	-0.318776
H	0.461582	0.063556	-2.377549	1.753200	0.467013	-3.792025	0.723403	0.607236	-2.644561
H	1.040275	1.313439	-3.512063	1.014707	-0.566308	-2.525627	1.581771	1.952324	-3.450534
H	-2.035945	3.421222	-2.689859	5.473830	-0.412241	-2.546110	-1.173127	4.627673	-2.364267
H	-4.102187	4.753465	-2.293068	7.758950	-1.209831	-1.958456	-3.254227	5.986581	-2.200756
H	-5.149929	4.773948	-0.038652	8.039849	-3.266268	-0.590540	-5.220836	5.098563	-0.967223
H	-4.137110	3.469218	1.818575	6.042676	-4.523984	0.195168	-5.108753	2.851014	0.110884
H	-2.057317	2.143581	1.411518	3.752313	-3.709098	-0.402721	-3.009757	1.524754	-0.016874
H	1.931320	3.269711	-1.250530	0.595889	2.538323	-3.253395	1.949171	3.488325	-0.705030
H	2.895623	5.642447	2.176505	-3.188709	4.400106	-0.375401	2.291015	3.567309	4.179439
H	4.318783	5.396379	3.257518	-3.868181	3.375537	-1.676588	2.354477	5.088386	3.270448
H	2.868139	4.343819	3.383698	-3.005847	4.916966	-2.066339	3.678632	4.681410	4.426531
H	1.886942	0.749376	0.975248	-0.346774	1.964968	0.529914	1.812699	0.455457	1.063871
H	5.449902	-3.237874	0.997898	3.550502	0.530170	3.435498	5.460166	-3.283658	0.623714
H	6.399080	-3.719191	-0.465931	3.121361	2.139547	4.071168	6.473587	-3.489286	-0.861605
H	4.618868	-3.888536	-0.440334	4.855746	1.705875	3.784586	4.696712	-3.676886	-0.939973
H	1.775239	-1.756440	1.599378	0.122332	1.072134	2.704294	1.978025	-1.884522	1.317745
H	2.350378	-3.425622	1.563321	0.865247	-0.100022	3.802633	2.573370	-3.515741	1.014942
H	0.864130	-3.001144	3.520318	-1.349014	0.693648	4.637878	1.206041	-3.376322	3.095389
H	0.140761	-4.213981	2.466713	-1.405535	-1.040720	4.329203	0.437032	-4.465747	1.942743
H	-0.519230	-1.215433	2.502564	-2.372369	1.167472	2.426809	-0.261258	-1.505095	2.372545
H	-1.530139	-2.492567	3.197402	-3.425280	0.144191	3.416408	-1.206688	-2.870989	2.984363

	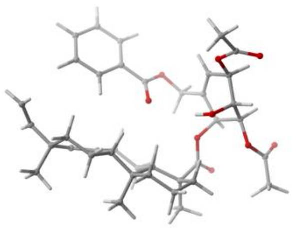 conformer **2-X**			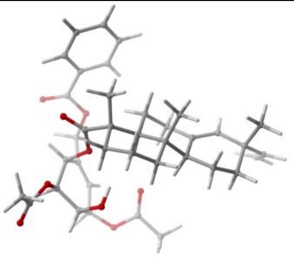 conformer **2-XI**			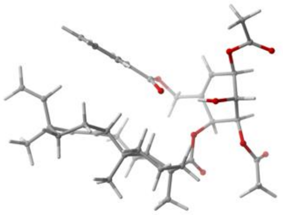 conformer **2-XII**		
C	2.636912	1.466753	2.461892	2.847006	-2.233747	-1.716258	-2.578035	-3.328391	1.153579
C	1.971158	0.217491	1.859055	2.606270	-0.764313	-1.309919	-1.753187	-2.035728	1.082010
C	0.703757	0.543919	1.017674	1.587179	-0.626457	-0.130692	-0.650737	-2.028364	-0.020733
C	1.015723	1.514993	-0.118900	2.074186	-1.467401	1.056694	-1.237007	-2.244747	-1.418288
C	2.086505	0.935062	-1.042626	3.425063	-0.944856	1.544383	-2.231477	-1.133183	-1.757616
C	3.288570	0.393385	-0.309781	4.449796	-0.801638	0.447844	-3.255279	-0.943147	-0.667855
C	4.533694	0.627202	-0.739897	5.716885	-1.193051	0.626450	-4.558476	-0.862541	-0.958312
C	5.781245	-0.000503	-0.162623	6.841540	-0.976042	-0.358297	-5.669948	-0.636362	0.045266
C	6.756283	-0.415177	-1.248306	8.114328	-0.524914	0.332883	-5.982727	0.850006	0.025602
C	6.502109	-0.551317	-2.549303	8.236202	-0.067018	1.578263	-7.145274	1.429878	-0.273053
C	6.495061	1.008475	0.759542	7.143521	-2.294263	-1.099637	-6.883980	-1.488516	-0.332777
C	5.375263	-1.276347	0.608834	6.425356	0.137918	-1.343959	-5.165957	-1.051729	1.441473
C	4.232001	-1.020593	1.583251	5.031450	-0.090285	-1.915706	-3.785204	-0.489115	1.770949
C	2.971911	-0.492673	0.880885	3.955167	-0.134323	-0.821423	-2.693057	-0.823777	0.735875
H	2.421837	-1.359657	0.468117	3.709745	0.908039	-0.548318	-2.009358	0.040762	0.712088
H	0.473729	-0.413369	0.528867	1.657956	0.431705	0.181397	-0.280538	-0.994511	0.002992
C	-0.593685	0.922388	1.795213	0.073781	-0.814581	-0.482072	0.633894	-2.880860	0.230660
C	-0.642235	2.355921	2.341362	-0.405046	-2.283767	-0.567913	0.475252	-4.383890	-0.016688
C	-1.815695	0.762242	0.882411	-0.764975	-0.221158	0.658759	1.697351	-2.361712	-0.742178
O	-1.645785	-0.243020	-0.004715	-1.984023	0.171723	0.214025	1.836794	-1.026187	-0.614483
C	-2.759061	-0.679985	-0.806091	-3.017282	0.428893	1.192057	2.843475	-0.340189	-1.361908
H	-3.567015	0.058066	-0.717007	-2.536336	0.684299	2.144315	3.058000	-0.922674	-2.272101
C	-2.237434	-0.795627	-2.216918	-3.841041	1.570296	0.655142	2.263272	1.017578	-1.691565
C	-2.341426	0.412817	-3.105233	-3.351786	2.967646	0.943955	0.970387	0.985721	-2.467500
O	-1.835547	1.587535	-2.456119	-1.934201	3.021434	1.105643	-0.019264	1.934997	-2.038362
C	-2.745986	2.451158	-1.947196	-1.197091	3.162974	-0.014277	-0.441535	1.894034	-0.762000
C	-2.091865	3.624988	-1.306911	0.263994	3.146693	0.279870	-1.686896	2.680334	-0.546827
C	-0.784041	4.008568	-1.616259	0.763938	2.836112	1.548391	-2.111829	2.878981	0.771490
C	-0.210845	5.099851	-0.968656	2.138800	2.843525	1.769349	-3.292668	3.568628	1.024200
C	-0.939084	5.801953	-0.008488	3.013411	3.166243	0.732430	-4.057627	4.051446	-0.039058
C	-2.247054	5.421945	0.296038	2.514935	3.472511	-0.535085	-3.640930	3.845229	-1.353642
C	-2.826662	4.339718	-0.357021	1.142888	3.458881	-0.762652	-2.453812	3.164683	-1.610704
O	-3.938724	2.268611	-1.994074	-1.675924	3.300295	-1.114550	0.107753	1.268672	0.119748
C	-1.672357	-1.930927	-2.633420	-4.979716	1.349079	-0.006325	2.793608	2.152875	-1.233754
C	-1.694696	-3.221768	-1.859388	-5.611352	-0.002674	-0.177636	3.926711	2.195065	-0.248648
H	-2.426007	-3.877122	-2.359026	-6.461486	-0.100203	0.519871	4.898226	2.371232	-0.739359
O	-0.483611	-3.984061	-1.964015	-6.118564	-0.042577	-1.513678	3.645172	3.299776	0.616039
C	0.690640	-3.388123	-1.674573	-7.041608	-0.992560	-1.773823	4.675140	3.754429	1.360414
C	1.794375	-4.401172	-1.585531	-7.396533	-1.001245	-3.232965	4.217985	4.847758	2.283970
O	0.827269	-2.199734	-1.507376	-7.493771	-1.726519	-0.930726	5.796570	3.318975	1.280625
C	-2.119649	-3.106401	-0.374952	-4.638376	-1.174463	0.077921	3.991072	0.873103	0.539379
H	-2.556805	-4.067766	-0.074822	-5.228191	-2.082461	0.255621	4.897880	0.869416	1.157813
O	-1.046395	-2.899534	0.503055	-3.841101	-1.452781	-1.043739	2.910700	0.722466	1.420784
C	-3.212791	-2.029924	-0.267532	-3.853625	-0.841375	1.345370	4.116753	-0.221575	-0.515935
H	-4.094437	-2.364692	-0.836648	-4.556802	-0.679611	2.176958	4.951735	0.022452	-1.190143
O	-3.576445	-1.813337	1.092439	-3.003313	-1.941180	1.653707	4.385412	-1.466698	0.114573
C	-4.413579	-2.709842	1.655411	-2.634275	-2.063815	2.954378	5.139468	-2.343752	-0.598025
C	-4.659057	-2.363832	3.095716	-1.592815	-3.131100	3.110199	5.191064	-3.675125	0.089500
O	-4.875968	-3.649498	1.057651	-3.083473	-1.374465	3.833821	5.662878	-2.066292	-1.646274
O	-2.837998	1.395624	0.960321	-0.455381	-0.129857	1.822527	2.339154	-3.020579	-1.524893
C	-0.840318	-0.107463	2.930680	-0.285674	-0.079471	-1.796140	1.179260	-2.611279	1.655856
C	0.391801	-0.307773	3.806601	0.690601	-0.362097	-2.928937	0.094699	-2.729925	2.720215

C	1.581059	-0.774277	2.971656	2.102884	0.032878	-2.523927	-1.060889	-1.773129	2.435068
H	3.438158	1.186087	3.161760	1.919482	-2.816904	-1.757986	-3.165020	-3.492037	0.238036
H	1.930569	2.088889	3.023255	3.523258	-2.743475	-1.014301	-3.289272	-3.274221	1.992530
H	3.089836	2.098422	1.683802	3.307313	-2.293292	-2.714021	-1.958164	-4.218427	1.318169
H	1.335798	2.492032	0.276908	2.164758	-2.525877	0.766107	-1.739402	-3.222638	-1.487045
H	0.103840	1.694258	-0.705147	1.354238	-1.412317	1.879893	-0.432323	-2.265533	-2.173861
H	2.400896	1.677205	-1.792814	3.821232	-1.575756	2.354431	-2.728841	-1.329828	-2.719163
H	1.633569	0.088561	-1.586386	3.254132	0.058865	1.976415	-1.674867	-0.186518	-1.879988
H	4.677368	1.307742	-1.587024	5.981045	-1.702061	1.560617	-4.869297	-0.919332	-2.008325
H	7.762903	-0.655927	-0.882414	9.008637	-0.561922	-0.302592	-5.128898	1.493362	0.264062
H	5.515166	-0.343102	-2.970280	7.380240	0.008102	2.253757	-8.038909	0.860498	-0.539060
H	7.278363	-0.887192	-3.240407	9.204788	0.258262	1.964027	-7.242186	2.518256	-0.261350
H	6.779547	1.910327	0.195507	7.973582	-2.164248	-1.813758	-7.265064	-1.226052	-1.331954
H	7.412358	0.572118	1.189084	6.262857	-2.651235	-1.653224	-7.702640	-1.358136	0.392159
H	5.841402	1.324946	1.585179	7.430289	-3.081203	-0.385013	-6.607842	-2.553834	-0.347280
H	6.248964	-1.684412	1.143908	7.165192	0.212419	-2.158101	-5.137249	-2.152559	1.472358
H	5.067080	-2.035297	-0.129696	6.447127	1.098508	-0.802189	-5.893588	-0.728686	2.203741
H	4.571004	-0.308723	2.350745	5.027914	-1.028301	-2.491580	-3.485582	-0.830303	2.771719
H	3.993274	-1.952218	2.117959	4.798534	0.710335	-2.633401	-3.857743	0.607771	1.847113
H	-1.657967	2.578229	2.693459	-0.061922	-2.875876	0.291717	0.055375	-4.596128	-1.009329
H	-0.397001	3.095854	1.565315	-0.035729	-2.762694	-1.483955	-0.174266	-4.844907	0.737229
H	0.047919	2.493849	3.182071	-1.503681	-2.323659	-0.592643	1.454779	-4.879680	0.033198
H	-3.389404	0.596144	-3.383452	-3.740983	3.302801	1.915986	0.529757	-0.019737	-2.400793
H	-1.736383	0.278955	-4.010997	-3.672414	3.665884	0.159029	1.122482	1.227393	-3.527450
H	-0.217653	3.447637	-2.359547	0.076493	2.570981	2.349644	-1.504252	2.482674	1.585890
H	0.810029	5.399714	-1.210684	2.529238	2.593419	2.756952	-3.622445	3.725880	2.052317
H	-0.484468	6.650925	0.505785	4.090744	3.168332	0.909820	-4.989197	4.584859	0.159091
H	-2.814526	5.969387	1.050183	3.200423	3.717389	-1.348198	-4.247014	4.212181	-2.183001
H	-3.840438	4.013832	-0.126197	0.729308	3.691871	-1.744828	-2.121443	2.995701	-2.634399
H	-1.235579	-1.989998	-3.635075	-5.545791	2.189015	-0.420302	2.328998	3.111743	-1.478304
H	2.762824	-3.897616	-1.679055	-7.621502	0.016522	-3.578766	3.663413	5.612712	1.723809
H	1.678315	-5.181428	-2.348105	-8.250471	-1.665815	-3.400035	5.084132	5.289529	2.787360
H	1.728980	-4.880682	-0.597015	-6.526785	-1.361072	-3.801975	3.531382	4.418309	3.028014
H	-0.690250	-2.017847	0.320910	-3.214567	-0.723868	-1.150191	2.077359	0.798520	0.928160
H	-5.448999	-3.005473	3.499575	-1.782204	-3.969405	2.428378	5.192676	-3.557044	1.180093
H	-4.930940	-1.303649	3.186911	-1.560503	-3.466307	4.152474	6.070281	-4.232805	-0.251172
H	-3.728527	-2.517037	3.661955	-0.621913	-2.683836	2.846576	4.282715	-4.224376	-0.202005
H	-1.114930	-1.076249	2.479622	-0.304845	1.006751	-1.618008	1.609938	-1.599346	1.694807
H	-1.703918	0.221517	3.530446	-1.302970	-0.358207	-2.104649	2.005913	-3.311059	1.860932
H	0.167125	-1.055261	4.583506	0.378474	0.205832	-3.818835	0.529876	-2.497841	3.704578
H	0.640699	0.623996	4.340967	0.659341	-1.425787	-3.218322	-0.275094	-3.766692	2.787116
H	1.319945	-1.741256	2.502974	2.112520	1.108508	-2.272623	-0.669707	-0.739788	2.433607
H	2.446085	-0.964031	3.626006	2.790330	-0.103226	-3.372601	-1.793215	-1.835867	3.254402

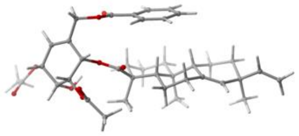

conformer **2-XIII**

C	2.878503	-2.136732	-1.692013
C	2.612637	-0.678035	-1.263254
C	1.577597	-0.573416	-0.094493
C	2.062573	-1.424586	1.086621
C	3.400938	-0.891678	1.598133
C	4.434958	-0.725692	0.513894
C	5.701233	-1.119312	0.693392
C	6.840773	-0.870413	-0.278659
C	8.027525	-0.383502	0.530645
C	9.242959	-0.925266	0.600257
C	7.162161	-2.158860	-1.048176
C	6.422047	0.265282	-1.235878
C	5.035089	0.043231	-1.827370
C	3.947056	-0.039337	-0.747147
H	3.683429	0.993498	-0.454908
H	1.628391	0.480951	0.234027
C	0.071675	-0.778736	-0.468014
C	-0.383048	-2.253371	-0.584680
C	-0.790826	-0.217357	0.671457
O	-2.011195	0.161242	0.217923
C	-3.060164	0.386116	1.187257
H	-2.594784	0.634981	2.148947
C	-3.897185	1.521450	0.657970
C	-3.436530	2.922231	0.975318
O	-2.021723	2.998813	1.152229
C	-1.276656	3.169999	0.041919
C	0.181728	3.174580	0.349952
C	1.064568	3.518060	-0.679267
C	2.434015	3.552055	-0.438551
C	2.925809	3.235157	0.828900
C	2.047617	2.880989	1.852425
C	0.675226	2.852967	1.618240
O	-1.747199	3.315573	-1.060851
C	-5.023486	1.291001	-0.021281
C	-5.629060	-0.068556	-0.222271
H	-6.487321	-0.191523	0.461149
O	-6.116422	-0.096182	-1.565991
C	-7.018237	-1.058413	-1.854167
C	-7.351542	-1.051199	-3.318379
O	-7.469274	-1.813085	-1.028948
C	-4.639774	-1.227587	0.029300
H	-5.216376	-2.147775	0.186347
O	-3.823852	-1.476375	-1.085885
C	-3.876910	-0.899944	1.311489
H	-4.593422	-0.761725	2.135915
O	-3.012652	-1.990084	1.615440
C	-2.657762	-2.124610	2.918838
C	-1.601559	-3.177466	3.073585
O	-3.128597	-1.454360	3.801751
O	-0.496754	-0.138319	1.840128
C	-0.282605	-0.027060	-1.774180
C	0.712208	-0.274829	-2.899081
C	2.113323	0.133521	-2.469239
H	1.959941	-2.731984	-1.754219
H	3.554266	-2.649169	-0.991632
H	3.350284	-2.172534	-2.685551

H	2.171245	-2.477430	0.781918
H	1.332278	-1.391295	1.901817
H	3.794671	-1.528203	2.404803
H	3.213332	0.104734	2.040070
H	5.969175	-1.631855	1.624764
H	7.823686	0.521585	1.117162
H	9.516066	-1.827268	0.047946
H	10.019556	-0.476268	1.223311
H	7.999544	-2.008787	-1.748390
H	6.287985	-2.495273	-1.623553
H	7.432246	-2.972361	-0.357395
H	7.171740	0.365840	-2.037117
H	6.426569	1.216663	-0.675342
H	5.045330	-0.878682	-2.428531
H	4.802791	0.860674	-2.526066
H	-1.480470	-2.309832	-0.623390
H	-0.040627	-2.855015	0.268575
H	0.004133	-2.710395	-1.504476
H	-3.841173	3.233698	1.948898
H	-3.761973	3.627857	0.199019
H	0.656048	3.758889	-1.661660
H	3.122485	3.821537	-1.241292
H	4.001166	3.254235	1.016848
H	2.433206	2.622458	2.839761
H	-0.014641	2.563415	2.408862
H	-5.599093	2.127476	-0.429069
H	-7.588212	-0.032260	-3.652691
H	-8.191738	-1.727168	-3.507897
H	-6.467665	-1.388362	-3.879455
H	-3.209365	-0.734961	-1.174863
H	-1.767516	-4.008158	2.376534
H	-1.578699	-3.527980	4.111074
H	-0.634306	-2.710775	2.831100
H	-0.321457	1.055456	-1.577907
H	-1.291279	-0.316414	-2.100690
H	0.402699	0.304126	-3.782768
H	0.700489	-1.333547	-3.207515
H	2.103939	1.204433	-2.198649
H	2.813542	0.022635	-3.311127

Table S13 Coordinates of compound **2-dias**

	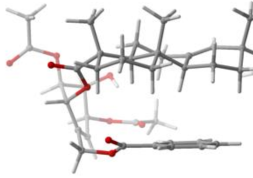 conformer **2-dias-I**			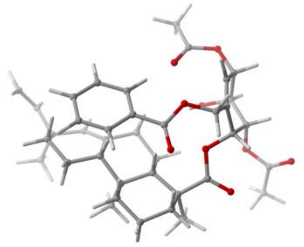 conformer **2-dias-II**			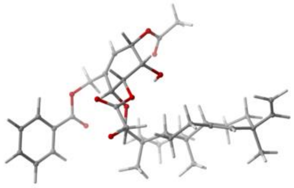 conformer **2-dias-III**		
C	-2.189377	-3.156625	-1.321949	-2.170809	-3.202952	-1.175375	2.905469	-3.139218	-1.013124
C	-1.759479	-2.205286	-0.186639	-1.736323	-2.190176	-0.097196	2.458211	-1.675915	-1.186868
C	-0.294336	-1.687745	-0.344860	-0.268438	-1.688768	-0.282217	1.219412	-1.318453	-0.317107
C	-0.122305	-0.996566	-1.698780	-0.089322	-1.070592	-1.670597	1.485464	-1.588462	1.163943
C	-1.041764	0.222528	-1.770964	-1.007976	0.143394	-1.815761	2.657992	-0.733806	1.644522
C	-2.477190	-0.110329	-1.449462	-2.443972	-0.175702	-1.484663	3.875305	-0.849110	0.761310
C	-3.479597	0.351204	-2.205883	-3.446217	0.225776	-2.274092	5.100479	-1.000109	1.277359
C	-4.948895	0.171318	-1.905998	-4.920210	0.057719	-1.957993	6.384674	-0.988423	0.481343
C	-5.731345	1.444983	-2.164361	-5.644631	1.301848	-2.436509	7.476736	-0.206766	1.186709
C	-5.238345	2.675799	-2.299890	-6.448229	2.098988	-1.733341	7.330442	0.646061	2.199994
C	-5.530122	-0.941125	-2.801092	-5.489764	-1.135770	-2.755841	6.886401	-2.433014	0.281792
C	-5.106801	-0.172018	-0.408912	-5.071114	-0.153207	-0.446225	6.115396	-0.295253	-0.872862
C	-4.168582	-1.286406	0.036906	-4.138722	-1.235740	0.084696	4.885378	-0.859850	-1.573049
C	-2.689768	-0.942123	-0.198143	-2.658132	-0.924337	-0.183074	3.610737	-0.714662	-0.727698
H	-2.353516	-0.315897	0.647101	-2.310926	-0.247916	0.618128	3.219549	0.310922	-0.876625
H	-0.200334	-0.903925	0.426404	-0.172099	-0.865158	0.446032	1.135342	-0.225522	-0.409707
C	0.844131	-2.693153	-0.011513	0.864370	-2.680288	0.107012	-0.173163	-1.843620	-0.801503
C	1.132667	-3.743733	-1.100482	1.147726	-3.790607	-0.922001	-0.458497	-3.324004	-0.520576
C	2.181535	-1.977831	0.231373	2.204825	-1.959603	0.313094	-1.211833	-1.038650	-0.015907
O	2.098841	-0.631226	0.355399	2.125386	-0.608783	0.378502	-1.115819	0.283781	-0.288769
C	3.336234	0.074211	0.587217	3.363360	0.103811	0.584001	-1.990842	1.188859	0.402105
H	4.024348	-0.620800	1.089375	4.047613	-0.568636	1.120915	-2.860281	0.625289	0.765451
C	3.005773	1.242449	1.474220	3.029574	1.311625	1.415239	-2.394109	2.230703	-0.606455
C	2.502827	0.932944	2.861951	2.514874	1.065287	2.811385	-3.339358	1.780066	-1.687340
O	1.108547	1.268577	2.900870	1.123230	1.413756	2.825327	-4.413029	1.017989	-1.124894
C	0.220452	0.298465	3.165108	0.225505	0.468063	3.140173	-4.394469	-0.317744	-1.328474
C	-1.173409	0.765487	2.929974	-1.163297	0.932891	2.872364	-5.456159	-1.015663	-0.555930
C	-2.219271	0.053586	3.524693	-1.419054	1.990902	1.993155	-6.350280	-0.326750	0.269222
C	-3.535400	0.453752	3.317307	-2.735752	2.370344	1.743782	-7.314856	-1.032158	0.983071
C	-3.806748	1.553103	2.501513	-3.787812	1.713707	2.382031	-7.386530	-2.420925	0.876189
C	-2.764122	2.247308	1.887861	-3.530349	0.655465	3.254753	-6.491909	-3.108780	0.055314
C	-1.443051	1.864516	2.106880	-2.218633	0.258472	3.493540	-5.526871	-2.407928	-0.659454
O	0.517609	-0.809550	3.552761	0.511045	-0.619213	3.590363	-3.598372	-0.874067	-2.049096
C	3.022132	2.502117	1.030495	3.051649	2.550026	0.915351	-1.834095	3.442061	-0.615591
C	3.389262	2.892947	-0.374420	3.427863	2.875737	-0.503679	-0.811117	3.905030	0.390177
H	4.430100	3.253517	-0.375862	4.470251	3.231676	-0.515840	-1.321827	4.565500	1.109158
O	2.665203	4.029933	-0.868829	2.711324	3.991509	-1.053649	0.189487	4.770663	-0.163322
C	1.325426	4.088732	-0.730929	1.370999	4.063008	-0.924587	0.840978	4.406043	-1.289675
C	0.765851	5.232398	-1.526467	0.820299	5.169066	-1.777369	1.960353	5.366433	-1.575156
O	0.670849	3.327501	-0.059028	0.710405	3.338750	-0.218839	0.559607	3.446494	-1.964983
C	3.280677	1.722892	-1.367836	3.319925	1.661966	-1.443223	-0.154754	2.760325	1.191009
H	3.824065	1.988178	-2.286213	3.866648	1.884443	-2.370895	0.292755	3.184029	2.101624
O	1.963872	1.478549	-1.774176	2.003851	1.401656	-1.842340	0.906754	2.146678	0.511929
C	3.975546	0.523166	-0.732057	4.009967	0.491065	-0.751163	-1.278852	1.813344	1.599687
H	5.016882	0.799500	-0.502086	5.050797	0.775522	-0.529066	-2.035874	2.381968	2.162341
O	3.976794	-0.545014	-1.667078	4.012983	-0.618022	-1.637128	-0.750535	0.792908	2.437820
C	5.062316	-1.364060	-1.637535	5.097030	-1.436521	-1.567172	-1.629655	0.246430	3.319452
C	4.845055	-2.557989	-2.516138	4.881613	-2.666931	-2.394436	-1.041657	-0.959787	3.985779
O	6.040210	-1.137427	-0.973087	6.072469	-1.182501	-0.909053	-2.737137	0.684591	3.499017
O	3.227769	-2.569528	0.359047	3.249730	-2.547782	0.465281	-2.016266	-1.485054	0.765718

C	0.554228	-3.402071	1.338438	0.569905	-3.311175	1.494483	-0.348200	-1.537664	-2.307261
C	-0.848891	-3.994229	1.392967	-0.836796	-3.890918	1.582635	0.832433	-2.035796	-3.134172
C	-1.895356	-2.906634	1.176550	-1.876931	-2.811416	1.303745	2.131789	-1.387287	-2.664676
H	-2.418428	-2.604651	-2.245778	-3.096375	-3.717393	-0.876704	2.069170	-3.843394	-1.091101
H	-3.094972	-3.715151	-1.041876	-1.421580	-3.981393	-1.354561	3.384791	-3.301982	-0.036418
H	-1.423293	-3.899671	-1.567669	-2.368232	-2.708714	-2.138163	3.634404	-3.423073	-1.786806
H	-0.367858	-1.689879	-2.518657	-0.329822	-1.807071	-2.453515	1.690840	-2.656977	1.336212
H	0.918251	-0.682270	-1.856260	0.952580	-0.765400	-1.838118	0.595607	-1.333607	1.755636
H	-0.969113	0.714875	-2.751825	-0.929253	0.579229	-2.822528	2.920751	-0.973557	2.686331
H	-0.703107	0.963758	-1.020953	-0.673875	0.925950	-1.106974	2.322037	0.318280	1.622700
H	-3.234749	0.916614	-3.112496	-3.202585	0.746535	-3.208080	5.200500	-1.127908	2.361358
H	-6.819944	1.308259	-2.200164	-5.483292	1.533169	-3.497938	8.480498	-0.358088	0.768817
H	-4.164113	2.874128	-2.257440	-6.664403	1.929789	-0.675924	6.354230	0.851612	2.646445
H	-5.899089	3.532149	-2.451861	-6.928860	2.960757	-2.201526	8.189645	1.178863	2.613299
H	-5.401477	-0.688620	-3.865114	-6.566388	-1.258984	-2.556595	6.143939	-3.042756	-0.253104
H	-6.607789	-1.077975	-2.611159	-4.970023	-2.069326	-2.494827	7.073578	-2.913262	1.254769
H	-5.022525	-1.900019	-2.621770	-5.359351	-0.978592	-3.838290	7.827922	-2.448454	-0.292209
H	-6.153615	-0.447206	-0.196530	-6.116588	-0.403009	-0.201003	7.000738	-0.388682	-1.523608
H	-4.887956	0.739303	0.168166	-4.833698	0.796162	0.058628	5.969257	0.780700	-0.680989
H	-4.436044	-2.212153	-0.494455	-4.416687	-2.200310	-0.365866	5.065330	-1.918810	-1.811564
H	-4.330160	-1.487224	1.106527	-4.295293	-1.346926	1.167857	4.748187	-0.349705	-2.538057
H	0.337114	-4.495748	-1.156052	0.348052	-4.540051	-0.936777	-0.222632	-3.601625	0.515504
H	2.066054	-4.268137	-0.856980	2.078503	-4.305409	-0.649732	0.112288	-3.976717	-1.192133
H	1.242378	-3.285229	-2.094095	1.259961	-3.387934	-1.939252	-1.526078	-3.529171	-0.679781
H	2.997013	1.557813	3.618567	3.009686	1.716953	3.544743	-2.806735	1.150850	-2.414603
H	2.631239	-0.123959	3.128409	2.632339	0.019563	3.123195	-3.789286	2.637519	-2.203559
H	-1.981995	-0.807433	4.151053	-0.594207	2.493126	1.487579	-6.281471	0.757381	0.350299
H	-4.353133	-0.093187	3.789471	-2.940609	3.178780	1.040423	-8.011488	-0.496368	1.629689
H	-4.839664	1.865301	2.336182	-4.817515	2.022905	2.192234	-8.142364	-2.971492	1.439551
H	-2.980599	3.088723	1.227982	-4.355467	0.137816	3.746698	-6.545699	-4.195745	-0.023201
H	-0.625456	2.397013	1.620789	-1.992225	-0.571384	4.164395	-4.811759	-2.922204	-1.301634
H	2.767672	3.317549	1.712158	2.793993	3.395716	1.557723	-2.134849	4.179037	-1.365436
H	0.747925	4.932902	-2.585161	0.807268	4.819254	-2.820569	2.317119	5.218955	-2.599904
H	1.403926	6.120748	-1.437983	1.461376	6.058303	-1.727710	2.779269	5.158386	-0.870322
H	-0.255433	5.445927	-1.193966	-0.202053	5.401823	-1.461563	1.636080	6.402914	-1.416414
H	1.424207	1.312623	-0.987752	1.463494	1.262706	-1.051213	0.577312	1.827097	-0.340681
H	4.276726	-2.288081	-3.414886	4.280210	-3.361564	-1.788857	0.033437	-0.831995	4.163565
H	4.252839	-3.281514	-1.935802	5.846541	-3.131057	-2.625193	-1.182384	-1.807331	3.297137
H	5.809965	-3.006796	-2.775575	4.322966	-2.433694	-3.309404	-1.577919	-1.162962	4.919029
H	0.648620	-2.667323	2.154258	0.668951	-2.532770	2.268246	-0.429723	-0.448210	-2.442120
H	1.321619	-4.173013	1.504755	1.332540	-4.076424	1.703651	-1.297814	-1.968629	-2.656915
H	-1.000386	-4.467700	2.375403	-0.990702	-4.306091	2.590707	0.659453	-1.794231	-4.194432
H	-0.967303	-4.795802	0.644469	-0.959433	-4.733590	0.881511	0.912894	-3.134357	-3.081517
H	-1.779614	-2.150469	1.971991	-1.756761	-2.011764	2.054702	2.043353	-0.293082	-2.797703
H	-2.906568	-3.329452	1.281479	-2.890850	-3.221306	1.432052	2.967723	-1.714619	-3.302321

	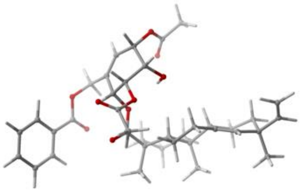			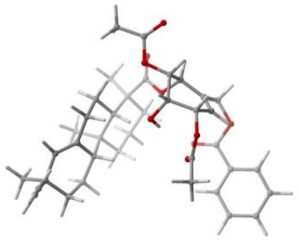			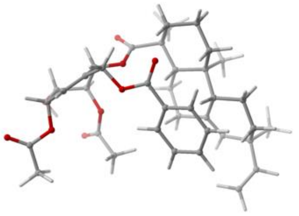		
	conformer **2-dias-IV**			conformer **2-dias-V**			conformer **2-dias-VI**		
C	2.905280	-3.139126	-1.013537	-3.809096	-0.083258	-2.480742	3.755243	2.075829	-1.641661
C	2.458069	-1.675760	-1.187061	-2.488310	-0.426945	-1.768018	2.857094	1.613682	-0.478159
C	1.219259	-1.318471	-0.317278	-1.774484	0.822479	-1.177057	1.345367	1.582112	-0.853861
C	1.485282	-1.588785	1.163721	-2.679384	1.576205	-0.200683	1.081706	0.650802	-2.037518
C	2.657773	-0.734211	1.644537	-3.103164	0.660318	0.948634	1.518406	-0.769434	-1.683751
C	3.875118	-0.849323	0.761350	-3.624740	-0.679244	0.492279	2.928563	-0.844293	-1.156233
C	5.100284	-1.000295	1.277433	-4.721429	-1.222609	1.033111	3.779666	-1.782954	-1.584517
C	6.384546	-0.988458	0.481533	-5.231735	-2.615303	0.744893	5.165612	-2.015848	-1.030963
C	7.476422	-0.206629	1.186997	-5.708669	-3.312096	2.005068	5.457065	-3.495616	-0.867232
C	7.329906	0.646102	2.200336	-5.461161	-2.956355	3.265127	4.569703	-4.490121	-0.847234
C	6.886479	-2.432994	0.282088	-6.418827	-2.542434	-0.236893	6.212613	-1.416631	-1.990474
C	6.115308	-0.295390	-0.872725	-4.070861	-3.451299	0.161324	5.257700	-1.373045	0.370501
C	4.885312	-0.860015	-1.572908	-3.365865	-2.735020	-0.983939	4.723780	0.053459	0.400893
C	3.610632	-0.714695	-0.727664	-2.780735	-1.380133	-0.556644	3.264072	0.155621	-0.068213
H	3.219593	0.310968	-0.876490	-1.797086	-1.570938	-0.084228	2.617358	-0.102802	0.786912
H	1.135169	-0.225509	-0.409644	-0.958046	0.400344	-0.572945	0.853542	1.117785	0.018123
C	-0.173274	-1.843542	-0.801781	-1.047276	1.768279	-2.186922	0.640957	2.967419	-0.996806
C	-0.458764	-3.323984	-0.521251	-1.955454	2.714138	-2.982404	1.000388	3.782731	-2.248613
C	-1.211938	-1.038670	-0.016095	-0.091007	2.646764	-1.374031	-0.870661	2.730933	-1.064618
O	-1.115715	0.283820	-0.288682	0.719572	1.898688	-0.594383	-1.320244	1.877091	-0.109315
C	-1.990743	1.188911	0.402187	1.681775	2.562240	0.236629	-2.734415	1.683890	-0.090909
H	-2.860226	0.625385	0.765462	1.911095	3.539787	-0.214083	-3.203386	2.645794	-0.349411
C	-2.393896	2.230884	-0.606285	2.889936	1.662868	0.269328	-3.163000	1.250950	1.295072
C	-3.339306	1.780499	-1.687163	3.647410	1.527921	-1.029779	-2.279372	1.622876	2.451134
O	-4.412956	1.018393	-1.124695	4.352710	0.290671	-1.085914	-1.309669	0.568850	2.531051
C	-4.394525	-0.317291	-1.328526	3.591012	-0.803655	-1.320679	-0.052108	0.864949	2.878920
C	-5.456089	-1.015304	-0.555898	4.335268	-2.068182	-1.073979	0.821779	-0.334490	2.743994
C	-5.526839	-2.407548	-0.659612	5.708251	-2.085610	-0.809901	2.049069	-0.354848	3.410714
C	-6.491753	-3.108490	0.055243	6.351781	-3.297801	-0.573800	2.884328	-1.461478	3.286471
C	-7.386208	-2.420738	0.876381	5.625909	-4.488747	-0.592458	2.504217	-2.536070	2.480957
C	-7.314491	-1.031984	0.983451	4.254259	-4.470085	-0.849558	1.286845	-2.506947	1.800871
C	-6.350037	-0.326491	0.269523	3.608990	-3.262204	-1.092590	0.438020	-1.413060	1.939813
O	-3.598651	-0.873532	-2.049471	2.439556	-0.746863	-1.676920	0.318117	1.955403	3.251493
C	-1.833661	3.442134	-0.615395	3.213875	0.946082	1.347116	-4.247428	0.490074	1.474700
C	-0.810624	3.904871	0.390423	2.431228	0.962517	2.630694	-5.094665	-0.041545	0.355071
H	-1.321283	4.565388	1.109402	2.940832	1.623928	3.349164	-6.162639	0.134322	0.543934
O	0.190114	4.770370	-0.163007	2.437608	-0.298921	3.317103	-4.897850	-1.466125	0.287696
C	0.841430	4.405715	-1.289439	2.196449	-1.441775	2.639123	-6.005744	-2.237481	0.131660
C	1.960680	5.366161	-1.575208	2.155882	-2.609574	3.583151	-5.604072	-3.633728	-0.252082
O	0.560001	3.446069	-1.964595	2.037493	-1.512731	1.444858	-7.128977	-1.841128	0.289424
C	-0.154461	2.760051	1.191251	0.996879	1.481759	2.436580	-4.713968	0.560641	-1.006536
H	0.293047	3.183679	2.101904	0.564927	1.717251	3.420012	-5.065342	1.607846	-0.990287
O	0.907011	2.146262	0.512230	0.142779	0.527023	1.869161	-5.370243	-0.054820	-2.068719
C	-1.278713	1.813204	1.599815	1.110923	2.780839	1.640402	-3.186193	0.662717	-1.132363
H	-2.035638	2.381893	2.162532	1.793326	3.465774	2.168067	-2.909637	0.983195	-2.146094
O	-0.750557	0.792598	2.437878	-0.173967	3.382233	1.559637	-2.447326	-0.534727	-0.792197
C	-1.629891	0.246011	3.319229	-0.190656	4.737012	1.444689	-2.613280	-1.667627	-1.472289
C	-1.042155	-0.960383	3.985467	-1.576507	5.225058	1.151037	-1.787233	-2.774822	-0.887185
O	-2.737400	0.684184	3.498623	0.797478	5.416870	1.551316	-3.348481	-1.798237	-2.429908
O	-2.016664	-1.485174	0.765173	-0.014422	3.851788	-1.425580	-1.625446	3.250552	-1.850774
C	-0.348300	-1.537259	-2.307526	-0.167485	0.931350	-3.149136	0.912136	3.805122	0.277535
C	0.832376	-2.035156	-3.134502	-0.945405	-0.207708	-3.800398	2.405119	3.911696	0.578754

C	2.131702	-1.386767	-2.664806	-1.523555	-1.137097	-2.736945	3.027881	2.528881	0.751165
H	3.385430	-3.301764	-0.037203	-4.190591	-0.950006	-3.040936	4.764407	2.325476	-1.282808
H	3.633487	-3.423207	-1.787821	-3.695542	0.733955	-3.201953	3.372770	2.966430	-2.148746
H	2.068826	-3.843218	-1.090571	-4.590260	0.212711	-1.765114	3.864398	1.286414	-2.401080
H	1.690689	-2.657338	1.335767	-3.566563	1.972471	-0.720118	1.614440	0.996807	-2.937138
H	0.595392	-1.334088	1.755434	-2.137687	2.437637	0.219112	0.008432	0.646087	-2.289955
H	2.920490	-0.974183	2.686307	-3.845943	1.157064	1.591574	1.388790	-1.451671	-2.537577
H	2.321807	0.317877	1.622938	-2.205976	0.479662	1.565357	0.851449	-1.122111	-0.875644
H	5.200249	-1.128204	2.361429	-5.297312	-0.632411	1.755216	3.462454	-2.450575	-2.394072
H	8.480240	-0.357718	0.769157	-6.290481	-4.224913	1.822149	6.516927	-3.734286	-0.711159
H	6.353634	0.851412	2.646766	-4.873559	-2.067977	3.510254	3.499884	-4.310845	-0.983978
H	8.188994	1.179036	2.613714	-5.834018	-3.553579	4.100167	4.886244	-5.523387	-0.689287
H	6.144259	-3.042790	-0.253089	-6.811803	-3.548976	-0.457734	7.235393	-1.595347	-1.619353
H	7.073380	-2.913267	1.255107	-6.123622	-2.069451	-1.184690	6.069956	-0.331970	-2.103354
H	7.828190	-2.448323	-0.291606	-7.237496	-1.944692	0.193080	6.128766	-1.871039	-2.989853
H	7.000670	-0.388860	-1.523444	-4.448127	-4.430977	-0.176510	6.303073	-1.393646	0.721073
H	5.969185	0.780588	-0.680949	-3.350716	-3.646348	0.973282	4.673510	-1.994858	1.064309
H	5.065203	-1.919023	-1.811276	-4.077979	-2.598985	-1.811615	5.371335	0.687815	-0.222064
H	4.748216	-0.349986	-2.537993	-2.562508	-3.376070	-1.376389	4.804990	0.445380	1.426049
H	0.111615	-3.976554	-1.193299	-2.510128	2.175645	-3.760012	0.999598	3.166873	-3.158750
H	-1.526444	-3.528890	-0.680078	-1.347286	3.484623	-3.474988	1.983076	4.257007	-2.152726
H	-0.222503	-3.602076	0.514619	-2.680349	3.232081	-2.339541	0.258170	4.577669	-2.393369
H	-2.806816	1.151361	-2.414590	4.416756	2.305260	-1.135345	-2.844380	1.643606	3.393705
H	-3.789267	2.638063	-2.203168	2.945281	1.602495	-1.872953	-1.767964	2.584672	2.318008
H	-4.811860	-2.921746	-1.302001	6.265624	-1.149272	-0.794525	2.332504	0.503145	4.021870
H	-6.545570	-4.195444	-0.023417	7.424363	-3.313790	-0.373376	3.841701	-1.483661	3.809556
H	-8.141955	-2.971368	1.439803	6.132229	-5.437489	-0.404061	3.165661	-3.397508	2.371061
H	-8.010985	-0.496278	1.630290	3.686626	-5.402043	-0.858992	1.002542	-3.341341	1.157265
H	-6.281186	0.757627	0.350760	2.537204	-3.218413	-1.284590	-0.514871	-1.366754	1.412280
H	-2.134340	4.179220	-1.365165	4.089811	0.295168	1.317839	-4.536226	0.193235	2.486399
H	1.636056	6.402671	-1.417340	2.974504	-2.549434	4.311758	-5.109229	-3.599075	-1.234181
H	2.317804	5.218055	-2.599740	2.209101	-3.541089	3.009911	-6.490492	-4.274871	-0.296890
H	2.779421	5.158894	-0.869942	1.206910	-2.575763	4.138816	-4.881418	-4.031797	0.474186
H	0.577608	1.826878	-0.340468	0.570292	0.155730	1.082620	-4.789202	-0.766776	-2.389406
H	-1.578488	-1.163517	4.918689	-2.323583	4.653250	1.716035	-2.123853	-2.947572	0.145460
H	0.032963	-0.832861	4.163311	-1.755090	5.062960	0.077033	-0.727885	-2.486413	-0.856157
H	-1.183068	-1.807849	3.296763	-1.645975	6.295439	1.372756	-1.914663	-3.683445	-1.484645
H	-0.429921	-0.447779	-2.442137	0.669896	0.494337	-2.585457	0.422709	3.326094	1.139820
H	-1.297883	-1.968212	-2.657291	0.261083	1.598948	-3.915419	0.465608	4.805586	0.156830
H	0.659450	-1.793365	-4.194722	-0.268168	-0.774544	-4.457787	2.543182	4.493715	1.502707
H	0.912859	-3.133735	-3.082110	-1.748570	0.181884	-4.448148	2.924607	4.470731	-0.217139
H	2.043273	-0.292523	-2.797539	-0.684689	-1.558618	-2.153948	2.546830	2.043503	1.618557
H	2.967653	-1.713919	-3.302526	-2.035320	-1.984838	-3.218770	4.096675	2.631617	0.994948

	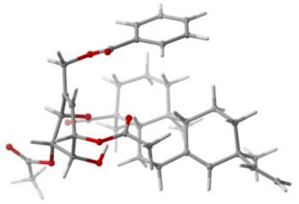 conformer **2-dias-VII**			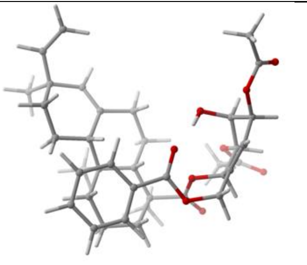 conformer **2-dias-VIII**			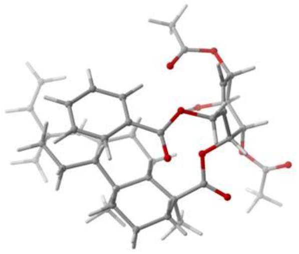 conformer **2-dias-IX**		
C	2.458065	-0.638167	-3.018499	-3.219396	-0.921503	-2.445496	-2.189315	-3.156169	-1.323348
C	1.775501	0.022369	-1.807180	-2.493612	-0.881882	-1.088954	-1.759701	-2.205435	-0.187372
C	0.332075	-0.508560	-1.538594	-0.939152	-0.843751	-1.220278	-0.294568	-1.687660	-0.345065
C	0.343117	-2.032881	-1.385713	-0.487869	0.340583	-2.079161	-0.122477	-0.995971	-1.698725
C	1.267731	-2.459648	-0.240451	-0.983016	1.660475	-1.484496	-1.041842	0.223224	-1.770371
C	2.617791	-1.785913	-0.246165	-2.437247	1.655847	-1.084514	-2.477301	-0.109703	-1.449056
C	3.735981	-2.462970	0.039061	-3.234043	2.703082	-1.326523	-3.479624	0.352563	-2.205145
C	5.107062	-1.837990	0.230697	-4.651499	2.847271	-0.822270	-4.948979	0.172769	-1.905497
C	5.721222	-2.465682	1.467049	-4.934583	4.255592	-0.334452	-5.731061	1.446918	-2.162579
C	6.875706	-3.124897	1.558846	-4.042239	5.203629	-0.050858	-5.237712	2.677727	-2.296881
C	5.968348	-2.070657	-1.018042	-5.644051	2.528224	-1.958606	-5.530436	-0.938593	-2.801779
C	4.919283	-0.331266	0.510844	-4.845791	1.892003	0.376861	-5.107103	-0.172096	-0.408784
C	3.994076	0.335453	-0.499853	-4.378533	0.476070	0.064632	-4.168926	-1.286920	0.036048
C	2.593837	-0.293254	-0.507938	-2.894244	0.426355	-0.323928	-2.690082	-0.942337	-0.198327
H	2.017828	0.156802	0.323785	-2.306455	0.407308	0.613950	-2.354129	-0.316629	0.647416
H	0.079742	-0.083205	-0.553687	-0.581816	-0.636818	-0.197264	-0.200824	-0.904114	0.426513
C	-0.796592	0.005472	-2.482880	-0.247118	-2.182388	-1.607805	0.843958	-2.693035	-0.011891
C	-0.850577	-0.658496	-3.865200	-0.400382	-2.602612	-3.078125	1.132920	-3.742984	-1.101394
C	-2.178565	-0.188682	-1.842868	1.261222	-2.115060	-1.335498	2.181242	-1.977660	0.231517
O	-2.151824	-0.051875	-0.499258	1.591642	-1.301958	-0.308513	2.098566	-0.631045	0.355493
C	-3.406704	0.014748	0.216495	2.996001	-1.148380	-0.042737	3.336086	0.074353	0.587002
H	-4.176328	0.378146	-0.477908	3.495473	-2.078303	-0.353471	4.024283	-0.620726	1.088947
C	-3.185530	0.963823	1.365538	3.178181	-0.926677	1.438484	3.006170	1.242649	1.474129
C	-3.256138	2.445092	1.099318	2.698635	-1.998024	2.381818	2.503763	0.933344	2.862082
O	-2.019764	2.948605	0.554718	1.276659	-2.227159	2.412161	1.109323	1.268285	2.901238
C	-1.032893	3.165469	1.444951	0.444354	-1.195038	2.622428	0.221711	0.297581	3.164873
C	0.214727	3.659355	0.798310	-0.947039	-1.643724	2.914688	-1.172370	0.764168	2.930161
C	0.193508	4.389874	-0.392718	-1.908732	-0.653238	3.138390	-1.442537	1.863386	2.107491
C	1.384566	4.878259	-0.924777	-3.224650	-1.010397	3.414310	-2.763774	2.245847	1.888900
C	2.595613	4.614131	-0.286023	-3.583040	-2.357970	3.465910	-3.806045	1.551122	2.502564
C	2.617178	3.874218	0.897205	-2.623061	-3.347751	3.252721	-3.534174	0.451563	3.317906
C	1.426950	3.409487	1.448089	-1.303831	-2.994201	2.982218	-2.217875	0.051723	3.524846
O	-1.148609	2.993746	2.635298	0.770914	-0.029164	2.592845	0.519386	-0.810520	3.551857
C	-2.821602	0.502909	2.564722	3.759698	0.173499	1.925447	3.022700	2.502306	1.030394
C	-2.695584	-0.950242	2.922369	4.140141	1.360761	1.089996	3.389277	2.893071	-0.374662
H	-3.500753	-1.179152	3.638303	5.209904	1.358633	0.823108	4.430124	3.253613	-0.376543
O	-1.513002	-1.229565	3.687835	3.870801	2.505750	1.902285	2.665017	4.030088	-0.868736
C	-0.311968	-0.829277	3.218665	4.430068	3.667454	1.499524	1.325316	4.088873	-0.730210
C	0.795862	-1.407034	4.050506	3.988721	4.800468	2.381197	0.765363	5.232674	-1.525296
O	-0.152003	-0.117260	2.255250	5.171397	3.753018	0.552802	0.671051	3.327587	-0.058052
C	-2.816239	-1.939286	1.737002	3.296419	1.369580	-0.193311	3.280216	1.723047	-1.368080
H	-3.214630	-2.891115	2.116587	3.650175	2.167560	-0.858430	3.823444	1.988287	-2.286564
O	-1.582691	-2.261426	1.163365	1.943329	1.634462	0.066036	1.963274	1.478975	-1.774106
C	-3.813988	-1.367979	0.729944	3.565372	0.024144	-0.859047	3.975031	0.523203	-0.732485
H	-4.798706	-1.249672	1.209274	4.653352	-0.121663	-0.939211	5.016494	0.799337	-0.502852
O	-3.917012	-2.296637	-0.340603	3.017118	0.019896	-2.168590	3.975796	-0.545010	-1.667494
C	-5.055246	-2.228679	-1.075077	3.740506	-0.617885	-3.128652	5.061194	-1.364219	-1.638237
C	-4.960977	-3.111713	-2.281772	2.952021	-0.714987	-4.399770	4.843460	-2.558288	-2.516541
O	-5.989069	-1.527416	-0.776172	4.848300	-1.049052	-2.944490	6.039342	-1.137689	-0.974125
O	-3.206362	-0.338642	-2.459690	2.084070	-2.775665	-1.925965	3.227432	-2.569400	0.359385
C	-0.691654	1.548055	-2.630918	-0.754801	-3.313688	-0.677354	0.553813	-3.402753	1.337553
C	0.706918	1.996354	-3.034387	-2.276435	-3.410655	-0.666128	-0.849219	-3.995202	1.391308

C	1.728357	1.546936	-1.997201	-2.907657	-2.092319	-0.231538	-1.895792	-2.907603	1.175370
H	2.667631	-1.703350	-2.845592	-3.048908	-0.006752	-3.030651	-3.094131	-3.715963	-1.043286
H	3.420952	-0.150445	-3.232412	-4.305823	-1.014499	-2.300144	-1.422583	-3.898141	-1.570311
H	1.856938	-0.561449	-3.932163	-2.912503	-1.773991	-3.061746	-2.419494	-2.603546	-2.246512
H	0.668083	-2.506941	-2.324634	-0.859563	0.229345	-3.110373	-0.368136	-1.688950	-2.518847
H	-0.672458	-2.409445	-1.182015	0.609973	0.372227	-2.130053	0.918102	-0.681748	-1.856162
H	1.384144	-3.553533	-0.223644	-0.779394	2.495592	-2.171595	-0.969170	0.716023	-2.751002
H	0.781347	-2.185371	0.710285	-0.382941	1.856564	-0.579638	-0.703129	0.964068	-1.020011
H	3.676473	-3.547206	0.190423	-2.839505	3.537172	-1.917902	-3.234651	0.918592	-3.111338
H	5.114341	-2.344628	2.373732	-5.999112	4.472234	-0.176222	-6.819697	1.310524	-2.198551
H	7.528680	-3.287055	0.698256	-2.968161	5.041866	-0.172249	-4.163423	2.875707	-2.254200
H	7.212338	-3.531794	2.514934	-4.360208	6.179024	0.323875	-5.898209	3.534417	-2.447999
H	6.974214	-1.636695	-0.898670	-6.686705	2.630385	-1.613879	-6.608160	-1.075347	-2.612104
H	5.498671	-1.616245	-1.901873	-5.501512	1.505672	-2.337097	-5.023120	-1.897807	-2.623365
H	6.079322	-3.146476	-1.223742	-5.496128	3.219105	-2.803019	-5.401613	-0.685063	-3.865535
H	5.903124	0.165321	0.521482	-5.905352	1.886486	0.682923	-6.153946	-0.447515	-0.196860
H	4.491624	-0.213331	1.522091	-4.267286	2.292365	1.226551	-4.888355	0.738619	0.169273
H	4.451069	0.270817	-1.498975	-5.002454	0.063878	-0.742459	-4.436229	-2.212119	-0.496349
H	3.910649	1.408550	-0.271242	-4.548769	-0.169946	0.939225	-4.330741	-1.488884	1.105417
H	-0.868095	-1.754985	-3.789813	0.293472	-3.423672	-3.298591	2.066125	-4.267655	-0.857772
H	0.008789	-0.373900	-4.483086	-0.172919	-1.773171	-3.762878	1.243181	-3.283838	-2.094652
H	-1.764404	-0.347483	-4.387610	-1.415814	-2.950587	-3.296666	0.337280	-4.494841	-1.157804
H	-3.491556	2.996273	2.019157	3.026222	-1.761983	3.405233	2.997749	1.558832	3.618305
H	-4.002113	2.677589	0.329315	3.100077	-2.980687	2.100517	2.632808	-0.123394	3.128871
H	-0.756772	4.578602	-0.893074	-1.604183	0.392584	3.089280	-0.625196	2.396321	1.621434
H	1.368677	5.461038	-1.847276	-3.974082	-0.235922	3.584802	-2.980669	3.087392	1.229324
H	3.528749	4.985935	-0.713172	-4.616824	-2.638964	3.674934	-4.839094	1.863052	2.337560
H	3.565697	3.664359	1.394417	-2.904454	-4.400974	3.297486	-4.351629	-0.095779	3.790084
H	1.415459	2.838127	2.376527	-0.546819	-3.760329	2.817099	-1.980203	-0.809448	4.150847
H	-2.603040	1.209569	3.368120	3.885383	0.290934	3.004405	2.768863	3.317837	1.712173
H	1.728076	-0.871265	3.842133	2.911499	4.965092	2.233219	0.747695	4.933709	-2.584141
H	0.915032	-2.463376	3.765275	4.544229	5.707036	2.119946	1.403089	6.121228	-1.436293
H	0.548509	-1.370311	5.118949	4.141777	4.542649	3.437847	-0.256021	5.445668	-1.192771
H	-1.228454	-1.451868	0.767247	1.634299	1.060942	0.785743	1.423773	1.313004	-0.987577
H	-4.444390	-4.049773	-2.042791	2.321635	0.171475	-4.543203	4.273458	-2.288883	-3.414367
H	-4.364566	-2.569821	-3.031133	2.298852	-1.596169	-4.308414	4.252812	-3.282278	-1.935183
H	-5.963281	-3.301387	-2.680481	3.632072	-0.859846	-5.246290	5.808304	-3.006364	-2.777496
H	-0.943518	2.011437	-1.662246	-0.402975	-3.106228	0.346668	0.647858	-2.668428	2.153780
H	-1.444092	1.888179	-3.360247	-0.300637	-4.267951	-0.988306	1.321299	-4.173640	1.503661
H	0.724728	3.093875	-3.119349	-2.582888	-4.209006	0.028658	-1.000909	-4.469432	2.373346
H	0.967068	1.607158	-4.032603	-2.648498	-3.711320	-1.659333	-0.967304	-4.796227	0.642177
H	1.469404	2.007403	-1.030204	-2.610876	-1.895689	0.809189	-1.780323	-2.151918	1.971303
H	2.727201	1.923461	-2.266725	-4.004563	-2.190109	-0.228877	-2.906972	-3.330606	1.279822

Table S14 Coordinates of compound **3**

	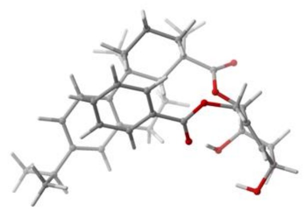 conformer **3-I**			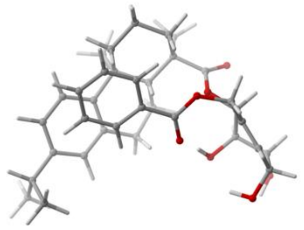 conformer **3-II**			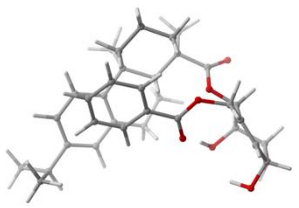 conformer **3-III**		
C	0.921042	3.602567	-2.174361	0.849565	3.344607	-2.578164	0.921436	3.601998	-2.175146
C	0.879775	2.594697	-1.018530	0.823963	2.488316	-1.305355	0.879867	2.594442	-1.018999
C	2.268890	1.948820	-0.964297	2.209467	1.841033	-1.198407	2.269085	1.948800	-0.963875
O	2.330291	0.925178	-0.079820	2.281450	0.928262	-0.200551	2.330116	0.925102	-0.079432
C	3.561662	0.190213	-0.056136	3.509811	0.192343	-0.114376	3.561529	0.190196	-0.055276
H	4.381818	0.906522	-0.222199	4.329609	0.879289	-0.377685	4.381747	0.906540	-0.220829
C	3.711325	-0.479321	1.291223	3.683499	-0.318761	1.297907	3.710683	-0.479774	1.291927
C	3.180446	0.210385	2.516331	3.181536	0.510457	2.446342	3.179667	0.209367	2.517319
O	1.758096	0.417877	2.635005	1.763812	0.747003	2.563328	1.757540	0.418782	2.634835
C	0.850943	-0.402737	2.102330	0.841300	-0.123026	2.148480	0.850157	-0.402091	2.102826
C	-0.528180	-0.068260	2.552483	-0.527159	0.277339	2.576510	-0.528919	-0.066847	2.552543
C	-0.781503	0.951582	3.477421	-0.756536	1.403638	3.375608	-0.782018	0.953915	3.476530
C	-2.090605	1.224847	3.861444	-2.056766	1.735170	3.743472	-2.091045	1.227742	3.860411
C	-3.144252	0.479307	3.330067	-3.125574	0.943571	3.319942	-3.144839	0.481797	3.329905
C	-2.891539	-0.541504	2.414853	-2.896265	-0.182392	2.530564	-2.892351	-0.539954	2.415672
C	-1.585329	-0.814135	2.022817	-1.599049	-0.515718	2.156642	-1.586222	-0.813107	2.023728
O	1.091812	-1.314657	1.336031	1.062284	-1.123155	1.494382	1.091020	-1.314744	1.337400
C	4.397905	-1.622259	1.403180	4.367352	-1.445752	1.526956	4.397175	-1.622786	1.403672
C	4.954091	-2.404424	0.239830	4.897160	-2.360024	0.450920	4.953790	-2.404623	0.240326
H	5.989858	-2.710186	0.451170	5.935855	-2.645482	0.675879	5.989517	-2.710344	0.451920
O	4.231617	-3.619100	0.078557	4.167391	-3.581010	0.445398	4.231490	-3.619354	0.078564
C	4.917318	-1.603851	-1.073669	4.837146	-1.715652	-0.945050	4.917318	-1.603765	-1.072987
H	5.728478	-0.855619	-1.069478	5.651204	-0.976957	-1.042625	5.728494	-0.855550	-1.068445
O	5.136606	-2.450164	-2.168026	5.031231	-2.682912	-1.939350	5.136742	-2.449792	-2.167541
C	3.585784	-0.851581	-1.171230	3.507187	-0.970924	-1.102331	3.585894	-0.851374	-1.170585
H	3.531989	-0.347497	-2.148305	3.435876	-0.582085	-2.129632	3.532244	-0.347087	-2.147557
O	2.494311	-1.760580	-1.064616	2.414082	-1.854483	-0.870779	2.494305	-1.760307	-1.064219
O	3.227101	2.293173	-1.611433	3.158057	2.099170	-1.897826	3.227680	2.293393	-1.610324
C	0.636080	3.298798	0.338433	0.614315	3.358149	-0.041921	0.635611	3.299048	0.337602
C	-0.804446	3.789356	0.477392	-0.818843	3.878552	0.061877	-0.804988	3.789635	0.475688
C	-1.827197	2.669150	0.278496	-1.856273	2.754780	0.021228	-1.827642	2.669352	0.276760
C	-1.663332	1.910447	-1.059692	-1.725283	1.834756	-1.215698	-1.663175	1.909989	-1.060973
C	-2.167502	2.791887	-2.222536	-2.239658	2.572513	-2.470623	-2.166895	2.790779	-2.224482
C	-0.176810	1.462491	-1.159818	-0.245207	1.359533	-1.283021	-0.176559	1.462077	-1.160285
H	-0.025592	0.839024	-0.262621	-0.083478	0.853997	-0.316128	-0.025689	0.838921	-0.262813
C	0.043644	0.527179	-2.346994	-0.055184	0.277259	-2.343648	0.044434	0.526381	-2.347060
C	-0.677959	-0.785655	-2.070448	-0.782522	-0.980167	-1.885514	-0.677363	-0.786360	-2.070499
C	-2.074602	-0.594227	-1.522893	-2.171907	-0.704925	-1.354836	-2.074136	-0.594859	-1.523295
C	-2.922032	-1.710564	-1.465410	-3.029656	-1.792506	-1.151710	-2.921407	-1.711299	-1.465452
C	-4.226696	-1.642739	-0.988093	-4.331529	-1.647009	-0.675347	-4.226102	-1.643476	-0.988228
C	-5.100312	-2.881892	-0.912778	-5.231245	-2.849288	-0.450709	-5.099397	-2.882818	-0.912192
C	-6.399830	-2.719733	-1.708502	-4.668234	-3.771774	0.637239	-6.399542	-2.721182	-1.706979
C	-5.385060	-3.264836	0.544876	-5.488433	-3.618592	-1.751323	-5.382930	-3.265592	0.545755
C	-4.694232	-0.390455	-0.568882	-4.777368	-0.348640	-0.413096	-4.693860	-0.391096	-0.569550
C	-3.862956	0.718531	-0.603574	-3.932383	0.739149	-0.591886	-3.862765	0.718013	-0.604676
C	-2.536900	0.644047	-1.056108	-2.614364	0.590792	-1.042486	-2.536652	0.643529	-1.057043
H	0.082769	4.304672	-2.132471	1.789710	3.908169	-2.627980	0.083785	4.304855	-2.133038
H	0.895145	3.096265	-3.149928	0.021947	4.059851	-2.604710	0.894657	3.095532	-3.150601
H	1.853056	4.180131	-2.131071	0.793067	2.721889	-3.482514	1.853949	4.178793	-2.132384
H	3.583482	1.231014	2.583771	3.595830	1.527335	2.391756	3.584046	1.229364	2.586242
H	3.494296	-0.344899	3.412244	3.505926	0.055596	3.393648	3.492073	-0.347356	3.412852

H	0.048421	1.526295	3.886782	0.085063	2.014558	3.699949	0.048029	1.528910	3.885253
H	-2.291069	2.022566	4.578496	-2.238607	2.615305	4.362327	-2.291325	2.026195	4.576701
H	-4.170675	0.697944	3.630584	-4.145244	1.208727	3.605539	-4.171199	0.700819	3.630361
H	-3.712896	-1.116553	1.987913	-3.729543	-0.793431	2.184147	-3.713823	-1.115477	1.989595
H	-1.373003	-1.597082	1.294757	-1.405322	-1.384520	1.527672	-1.374001	-1.596916	1.296570
H	4.507789	-2.090496	2.385431	4.494908	-1.798843	2.554192	4.506681	-2.091380	2.385800
H	3.332916	-3.336924	-0.165721	3.264534	-3.323798	0.189629	3.332765	-3.337222	-0.165665
H	4.731035	-3.293990	-1.917243	4.626074	-3.489762	-1.587066	4.731082	-3.293674	-1.917109
H	1.964739	-1.525961	-0.280906	1.900815	-1.527415	-0.109852	1.964539	-1.525528	-0.280731
H	1.340304	4.139202	0.452801	1.328039	4.198316	-0.046227	1.339761	4.139524	0.451860
H	0.850277	2.580898	1.147272	0.838539	2.743475	0.845567	0.849514	2.581486	1.146815
H	-0.993055	4.609814	-0.234101	-1.013415	4.607296	-0.741837	-0.993255	4.609798	-0.236235
H	-0.940717	4.224976	1.479904	-0.931395	4.435991	1.005277	-0.941745	4.225658	1.477960
H	-2.838544	3.097647	0.353019	-2.861958	3.200981	0.060605	-2.839011	3.097918	0.350582
H	-1.732203	1.944088	1.100963	-1.752250	2.135140	0.924742	-1.733084	1.944649	1.099574
H	-1.901640	2.373653	-3.203859	-3.335852	2.649322	-2.423999	-3.264499	2.851817	-2.185988
H	-3.265111	2.852559	-2.183854	-1.847682	3.592834	-2.556571	-1.784198	3.817350	-2.179206
H	-1.785166	3.818558	-2.176620	-1.992873	2.034315	-3.396953	-1.901058	2.371780	-3.205488
H	1.113375	0.317568	-2.497120	1.009484	0.037463	-2.484042	1.114219	0.316621	-2.496571
H	-0.320966	0.982157	-3.281399	-0.433916	0.611487	-3.322432	-0.319689	0.981088	-3.281781
H	-0.086687	-1.361794	-1.339661	-0.187570	-1.455906	-1.088315	-0.086363	-1.362464	-1.339467
H	-0.722055	-1.406308	-2.978583	-0.840258	-1.717812	-2.700504	-0.721235	-1.407120	-2.978574
H	-2.535522	-2.671668	-1.819616	-2.651446	-2.791794	-1.388786	-2.534714	-2.672489	-1.819236
H	-4.529885	-3.708295	-1.369139	-6.201368	-2.463802	-0.093403	-4.529074	-3.709132	-1.368844
H	-6.983126	-3.653721	-1.695694	-5.354372	-4.610457	0.834235	-6.982502	-3.655373	-1.693628
H	-6.192487	-2.456891	-2.756513	-4.513253	-3.227910	1.581910	-6.193039	-2.458420	-2.755179
H	-7.034513	-1.926218	-1.281935	-3.698027	-4.196881	0.333142	-7.034211	-1.927843	-1.280072
H	-5.952880	-2.470737	1.057770	-6.192910	-4.447937	-1.581548	-4.446892	-3.426534	1.102642
H	-4.449510	-3.426351	1.102409	-4.555532	-4.049163	-2.149594	-5.977389	-4.191007	0.602407
H	-5.979976	-4.189992	0.600879	-5.910696	-2.959478	-2.524499	-5.950730	-2.471610	1.058859
H	-5.716559	-0.279370	-0.199176	-5.797452	-0.187217	-0.054472	-5.716213	-0.280025	-0.199917
H	-4.260363	1.674393	-0.257873	-4.315700	1.735489	-0.364767	-4.260337	1.673959	-0.259396

Table S15 Coordinates of compound **3-dias**

	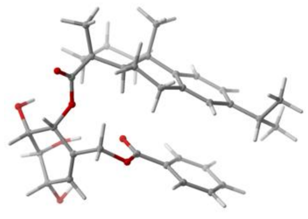 conformer **3-dias-I**			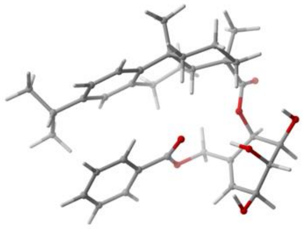 conformer **3-dias-II**			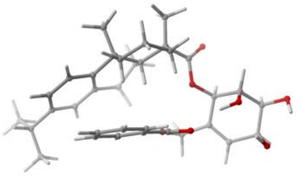 conformer **3-dias-III**		
C	-2.199606	4.040911	0.739955	2.594771	3.917894	-0.800386	0.737330	4.090560	-0.275415
C	-1.956717	2.632038	0.188414	2.210281	2.550295	-0.227357	0.759876	2.591889	0.053118
C	-2.848488	1.674987	0.987483	2.970658	1.488399	-1.029051	1.952089	2.006123	-0.710673
O	-2.842105	0.444320	0.428076	2.806860	0.267579	-0.466240	2.126107	0.685963	-0.467841
C	-3.652619	-0.592344	0.996521	3.474896	-0.866724	-1.034766	3.269750	0.080397	-1.089858
H	-4.045016	-0.218326	1.954298	3.876351	-0.560854	-2.012711	3.401897	0.559581	-2.073123
C	-2.755480	-1.792404	1.199084	2.435611	-1.956319	-1.174189	3.015661	-1.399738	-1.260096
C	-1.545686	-1.551365	2.068062	1.250079	-1.610548	-2.042803	1.618825	-1.876795	-1.540176
O	-0.335882	-2.148931	1.575955	0.015142	-2.169775	-1.576699	0.612081	-1.728438	-0.517134
C	0.092552	-1.804301	0.348388	-0.421778	-1.757706	-0.371361	0.888043	-1.692498	0.788625
C	1.448638	-2.332902	0.049916	-1.726865	-2.361320	0.005013	-0.349139	-1.708796	1.616657
C	1.998440	-2.032676	-1.198935	-2.360107	-1.865438	1.148049	-1.617312	-1.905479	1.062373
C	3.266921	-2.496675	-1.526343	-3.567371	-2.415735	1.564367	-2.737447	-1.911540	1.887914
C	3.990049	-3.255539	-0.606556	-4.140991	-3.463551	0.844130	-2.595757	-1.710939	3.259851
C	3.447556	-3.545044	0.645975	-3.514463	-3.952868	-0.302981	-1.329730	-1.516171	3.815135
C	2.176723	-3.085154	0.977356	-2.309951	-3.399760	-0.728621	-0.206252	-1.521024	2.996077
O	-0.547562	-1.127915	-0.429269	0.188329	-0.982149	0.331973	2.001152	-1.641264	1.270873
C	-2.954961	-2.955139	0.573952	2.504678	-3.106283	-0.498871	4.040199	-2.259763	-1.275014
C	-3.978881	-3.163605	-0.516743	3.530927	-3.404404	0.567895	5.473451	-1.886349	-0.990791
H	-4.872317	-3.672707	-0.117282	4.336795	-4.037240	0.159068	6.139144	-2.350888	-1.733897
O	-3.458962	-3.993809	-1.528380	2.950038	-4.127523	1.627073	5.875503	-2.424271	0.262514
C	-4.381512	-1.804604	-1.095756	4.125900	-2.090188	1.078492	5.693402	-0.362952	-0.993427
H	-5.214931	-1.937969	-1.799500	4.961789	-2.304263	1.758813	5.702538	0.004700	-2.033917
O	-3.319279	-1.264570	-1.873047	3.171035	-1.392101	1.871273	6.937724	-0.049973	-0.431317
C	-4.816136	-0.854630	0.014986	4.632638	-1.240921	-0.082443	4.530487	0.318068	-0.262456
H	-5.635700	-1.330119	0.576981	5.364142	-1.835035	-0.653025	4.729666	1.399188	-0.202453
O	-5.322617	0.348857	-0.496516	5.304718	-0.095130	0.366943	4.411619	-0.207020	1.054286
O	-3.444469	1.936145	2.001967	3.592086	1.667863	-2.045202	2.674874	2.622570	-1.454766
C	-2.353706	2.539489	-1.303982	2.628798	2.432312	1.257250	0.978692	2.345040	1.564913
C	-1.316646	3.167648	-2.228150	1.680589	3.172208	2.193502	-0.266020	2.675563	2.387524
C	0.055980	2.524707	-2.033660	0.249189	2.660915	2.039120	-1.507036	1.919669	1.907211
C	0.583698	2.662443	-0.589171	-0.301134	2.836628	0.607538	-1.810221	2.130986	0.404992
C	0.983483	4.130572	-0.327718	-0.584343	4.331414	0.348693	-2.366445	3.554079	0.180527
C	-0.500104	2.092810	0.371438	0.701354	2.172460	-0.380442	-0.507258	1.797242	-0.377191
H	-0.569600	1.032686	0.084353	0.661162	1.107301	-0.109830	-0.280606	0.756152	-0.090958
C	-0.011578	2.127675	1.820128	0.195374	2.282179	-1.819154	-0.759475	1.761863	-1.883375
C	1.175936	1.184340	1.994134	-1.097405	1.486806	-1.985034	-1.640687	0.558684	-2.195005
C	2.150229	1.196602	0.835157	-2.017074	1.526683	-0.782956	-2.828411	0.441708	-1.264331
C	3.365111	0.523886	0.992722	-3.268997	0.910687	-0.909298	-3.863609	-0.431262	-1.618721
C	4.335274	0.464908	-0.006907	-4.182784	0.846238	0.136869	-5.002121	-0.607001	-0.833836
C	5.660068	-0.224995	0.277491	-5.550590	0.198281	0.001630	-6.101125	-1.573834	-1.237341
C	6.512105	0.631450	1.225659	-5.655171	-0.789663	-1.159722	-5.591373	-3.019965	-1.259944
C	6.458937	-0.582798	-0.975079	-6.642706	1.272663	-0.099764	-6.734922	-1.188211	-2.578621
C	4.039876	1.093841	-1.218361	-3.803721	1.428584	1.351957	-5.091853	0.143404	0.341766
C	2.829540	1.756643	-1.395851	-2.566818	2.042821	1.493734	-4.064871	1.000688	0.715150
C	1.863235	1.831535	-0.385655	-1.644172	2.105357	0.438727	-2.908184	1.157972	-0.059107
H	-1.838704	4.143169	1.771962	3.688254	4.031832	-0.800954	1.752045	4.502919	-0.204050
H	-3.277007	4.258721	0.751178	2.169120	4.735079	-0.205719	0.097109	4.647566	0.415445
H	-1.707270	4.803361	0.125156	2.257697	4.034569	-1.838763	0.382467	4.276344	-1.298932
H	-1.664331	-1.987233	3.068522	1.356938	-2.006873	-3.061185	1.642391	-2.944323	-1.802496
H	-1.375359	-0.471036	2.181863	1.144390	-0.517846	-2.103720	1.190315	-1.322348	-2.388510

H	1.422121	-1.423716	-1.895101	-1.900247	-1.036843	1.687452	-1.728917	-2.044205	-0.011958
H	3.696204	-2.259400	-2.500852	-4.064602	-2.022306	2.452346	-3.725466	-2.050057	1.449295
H	4.987978	-3.616493	-0.863470	-5.087309	-3.897168	1.173139	-3.478625	-1.704985	3.901855
H	4.017818	-4.134039	1.366140	-3.968152	-4.768368	-0.868352	-1.219830	-1.361497	4.889635
H	1.742242	-3.305890	1.951783	-1.812254	-3.773152	-1.623316	0.793879	-1.376486	3.405488
H	-2.269874	-3.791853	0.740455	1.719368	-3.858670	-0.616444	3.846021	-3.326873	-1.415616
H	-2.939890	-3.402507	-2.094414	2.522011	-3.459088	2.183029	5.319123	-1.958076	0.911787
H	-2.549435	-1.106856	-1.297936	2.421060	-1.137942	1.307329	7.070978	-0.711009	0.264857
H	-4.674252	0.650365	-1.148004	4.731679	0.300890	1.038052	3.511829	-0.555535	1.178069
H	-3.339871	3.012632	-1.445941	3.660543	2.804813	1.372367	1.839720	2.936003	1.917115
H	-2.455503	1.479536	-1.580830	2.630150	1.370843	1.546906	1.236920	1.284614	1.713386
H	-1.258026	4.256075	-2.065352	1.723695	4.258503	2.012706	-0.452393	3.761872	2.371627
H	-1.637318	3.034920	-3.272972	2.011263	3.024627	3.233183	-0.076618	2.420183	3.441861
H	0.765389	2.975445	-2.744051	-0.396381	3.182988	2.761658	-2.368729	2.229661	2.518253
H	-0.010439	1.451044	-2.280742	0.220734	1.588005	2.296598	-1.367214	0.841904	2.084954
H	0.165141	4.837298	-0.514570	-0.970452	4.507399	-0.665118	-1.780686	4.326429	0.691988
H	1.335474	4.283540	0.702196	-1.357430	4.680333	1.050656	-2.416036	3.815968	-0.886155
H	1.815929	4.402866	-0.994411	0.301302	4.965046	0.488400	-3.391617	3.611106	0.574548
H	-0.816387	1.840063	2.515202	0.949619	1.908841	-2.530184	0.185028	1.683347	-2.444052
H	0.272820	3.151683	2.106244	0.031732	3.336217	-2.089571	-1.241634	2.690695	-2.226528
H	0.811293	0.149330	2.115830	-0.856267	0.426897	-2.178725	-1.034570	-0.359117	-2.092350
H	1.715670	1.414873	2.926086	-1.647107	1.828660	-2.876320	-1.993853	0.587024	-3.237511
H	3.559769	0.024784	1.948017	-3.514551	0.457233	-1.872482	-3.766837	-0.985904	-2.557398
H	5.418187	-1.166554	0.800800	-5.723195	-0.367349	0.934628	-6.886929	-1.508485	-0.465762
H	6.765873	1.593754	0.751938	-4.845012	-1.532965	-1.132282	-4.805608	-3.151074	-2.021645
H	7.451988	0.117203	1.482231	-5.617058	-0.273088	-2.132672	-6.407730	-3.719913	-1.497549
H	5.976122	0.850974	2.160946	-6.614740	-1.327261	-1.115560	-5.165638	-3.309560	-0.286810
H	6.823900	0.319245	-1.492560	-6.504313	1.875349	-1.012125	-6.002322	-1.256375	-3.399081
H	5.857859	-1.163222	-1.690327	-6.615282	1.958172	0.760511	-7.116814	-0.156700	-2.554440
H	7.341844	-1.182072	-0.705094	-7.643253	0.813627	-0.140774	-7.571954	-1.861048	-2.822395
H	4.755215	1.076618	-2.041973	-4.487595	1.395417	2.204778	-5.974405	0.048337	0.979790
H	2.646007	2.243159	-2.354815	-2.316083	2.485172	2.458637	-4.169863	1.560619	1.646000

	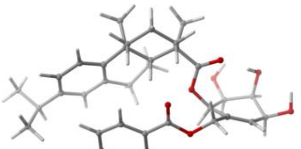 conformer **3-dias-IV**			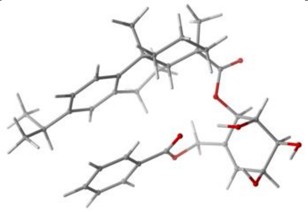 conformer **3-dias-V**			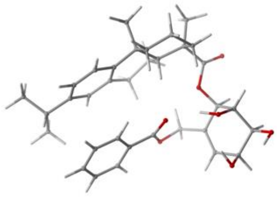 conformer **3-dias-VI**		
C	1.446534	3.505404	-0.354882	1.647922	4.321346	-0.805948	1.750225	4.326250	-0.816555
C	1.063527	2.203269	0.371201	1.557752	2.911955	-0.211845	1.624465	2.915610	-0.232814
C	2.237146	1.243345	0.149828	2.660689	2.066235	-0.862093	2.689032	2.037205	-0.904069
O	1.889658	0.024348	-0.258348	2.443269	0.750709	-0.645658	2.412815	0.728098	-0.715738
C	2.890931	-0.933865	-0.636131	3.506124	-0.176776	-0.863608	3.434013	-0.243717	-0.934514
H	2.251045	-1.785697	-0.902904	4.163617	0.221791	-1.652641	4.089422	0.107545	-1.747556
C	3.741809	-1.379751	0.530370	2.896311	-1.506526	-1.266018	2.760206	-1.558382	-1.285242
C	3.012189	-1.578577	1.831397	1.622213	-1.475722	-2.072641	1.459206	-1.501213	-2.047435
O	1.776736	-2.254525	1.558443	0.497880	-2.148192	-1.470344	0.351029	-2.162238	-1.403382
C	0.639351	-1.661300	1.967308	0.105061	-1.809660	-0.234786	-0.004530	-1.779803	-0.169400
C	-0.565171	-2.237606	1.313111	-1.212794	-2.399151	0.120567	-1.282873	-2.396258	0.273237
C	-0.471990	-3.202222	0.304785	-1.764637	-2.059110	1.358379	-1.907059	-3.418256	-0.449932
C	-1.628374	-3.662606	-0.319326	-2.997454	-2.580590	1.733412	-3.094921	-3.968867	0.020820
C	-2.873670	-3.165205	0.064701	-3.677775	-3.445068	0.876447	-3.669089	-3.489143	1.199178
C	-2.968323	-2.211161	1.076978	-3.133550	-3.776241	-0.364685	-3.054573	-2.459201	1.909781
C	-1.816287	-1.746074	1.698460	-1.902965	-3.251847	-0.747159	-1.858901	-1.916624	1.452103
O	0.606626	-0.748686	2.761502	0.737107	-1.093575	0.514918	0.633612	-1.006764	0.515989
C	5.040264	-1.654705	0.392740	3.462045	-2.660284	-0.897498	3.290153	-2.723774	-0.900189
C	5.818862	-1.435303	-0.879814	4.646627	-2.768748	0.028991	4.494868	-2.857437	-0.003684
H	5.824639	-2.375577	-1.459588	5.374424	-3.490427	-0.371615	5.183578	-3.615489	-0.406174
O	7.164003	-1.119633	-0.615695	4.240997	-3.301658	1.283712	4.102574	-3.346266	1.273167
C	5.185256	-0.328142	-1.727573	5.344291	-1.414565	0.246357	5.248346	-1.526576	0.165037
H	5.664733	-0.329608	-2.717681	5.931488	-1.157495	-0.652102	5.823444	-1.312724	-0.752357
O	5.481348	0.939302	-1.174507	6.238969	-1.493304	1.321642	6.164281	-1.614404	1.221511
C	3.678137	-0.516557	-1.931337	4.288585	-0.322770	0.439201	4.239079	-0.391564	0.354663
H	3.549228	-1.350256	-2.639991	4.791557	0.630393	0.672295	4.781920	0.546418	0.556830
O	3.128966	0.625284	-2.535722	3.418830	-0.665713	1.514099	3.378312	-0.678647	1.453906
O	3.390768	1.560458	0.378270	3.639065	2.482645	-1.430069	3.685990	2.421954	-1.461647
C	1.039256	2.414320	1.908500	1.935986	2.916468	1.294561	2.029710	2.896985	1.266622
C	-0.225713	3.113229	2.390447	0.812004	3.445057	2.178395	0.935352	3.444563	2.175198
C	-1.479150	2.361060	1.946701	-0.481906	2.658984	1.974272	-0.377146	2.685640	1.990553
C	-1.581146	2.238301	0.411461	-0.979904	2.687542	0.512175	-0.906576	2.743263	0.540788
C	-1.892362	3.628905	-0.186124	-1.523860	4.093413	0.179982	-1.435240	4.161876	0.241719
C	-0.270829	1.570110	-0.105417	0.187481	2.197561	-0.391732	0.229922	2.247244	-0.398142
H	-0.287825	0.574114	0.366781	0.364392	1.168830	-0.045928	0.383566	1.206904	-0.080282
C	-0.353087	1.335299	-1.616307	-0.256050	2.094472	-1.850222	-0.244012	2.192356	-1.849564
C	-1.400511	0.263027	-1.882723	-1.319914	1.010102	-1.997595	-1.345033	1.146798	-2.002052
C	-2.687058	0.483846	-1.118320	-2.330703	0.983550	-0.870341	-2.320568	1.101407	-0.844924
C	-3.824482	-0.231312	-1.512705	-3.459700	0.172999	-1.020997	-3.462967	0.301067	-0.992195
C	-5.038899	-0.150353	-0.838855	-4.452644	0.067165	-0.048210	-4.412999	0.158050	0.012161
C	-6.248816	-0.941349	-1.303137	-5.684063	-0.780798	-0.323699	-5.678310	-0.670049	-0.146246
C	-7.369275	-0.013360	-1.787378	-6.594183	-0.081417	-1.343935	-5.659951	-1.614771	-1.346556
C	-6.756747	-1.898765	-0.218849	-6.477631	-1.150918	0.928880	-6.911290	0.243682	-0.204532
C	-5.097987	0.690635	0.278342	-4.271997	0.797700	1.128504	-4.179794	0.842801	1.211933
C	-3.982287	1.415888	0.675885	-3.148134	1.598817	1.298974	-3.055609	1.638130	1.373398
C	-2.758353	1.336263	-0.004792	-2.157924	1.716898	0.316004	-2.100781	1.790726	0.355464
H	0.780440	4.329272	-0.075048	1.079324	5.047204	-0.213576	2.806396	4.625604	-0.838168
H	1.399019	3.386438	-1.447488	1.268813	4.352908	-1.837682	1.196777	5.060427	-0.219434
H	2.471973	3.790600	-0.083919	2.696627	4.645125	-0.832029	1.373954	4.374506	-1.848620
H	2.790522	-0.619430	2.320802	1.746695	-2.006616	-3.023982	1.539288	-2.028117	-3.005829
H	3.600176	-2.196828	2.522742	1.323011	-0.440561	-2.281033	1.171346	-0.459612	-2.237029
H	0.506341	-3.585379	0.015616	-1.218636	-1.376767	2.009392	-1.454075	-3.779770	-1.372585
H	-1.557966	-4.410563	-1.110793	-3.431275	-2.308507	2.696703	-3.579484	-4.772996	-0.535434

H	-3.777765	-3.516726	-0.436546	-4.644408	-3.857057	1.172863	-4.605583	-3.918303	1.560835
H	-3.939532	-1.804664	1.360860	-3.671219	-4.447699	-1.036138	-3.511417	-2.072188	2.821649
H	-1.868248	-0.989263	2.481155	-1.468747	-3.502681	-1.714277	-1.367449	-1.104715	1.987701
H	5.610438	-2.067980	1.230024	2.997543	-3.605643	-1.193908	2.778159	-3.656678	-1.154827
H	7.165595	-0.162360	-0.460978	3.644990	-2.624235	1.649751	3.540934	-2.639228	1.637648
H	4.888612	1.085212	-0.408824	5.823762	-2.105014	1.948462	5.742722	-2.198412	1.870204
H	3.688148	1.364719	-2.250172	2.499480	-0.592577	1.203183	2.456617	-0.570252	1.161119
H	1.940136	2.972127	2.205291	2.853784	3.509617	1.434743	2.964500	3.465709	1.394396
H	1.087528	1.428724	2.397128	2.171036	1.884499	1.603585	2.244946	1.857405	1.564184
H	-0.255401	4.155463	2.032924	0.643365	4.516706	1.983934	0.785061	4.520521	1.989492
H	-0.203418	3.170951	3.489639	1.120896	3.374545	3.232770	1.262956	3.361185	3.222938
H	-2.366019	2.871185	2.351839	-1.252419	3.060731	2.649469	-1.125187	3.092760	2.687567
H	-1.457371	1.348591	2.383727	-0.315409	1.607424	2.265850	-0.222554	1.627560	2.264859
H	-1.170044	4.398119	0.114524	-1.837386	4.174113	-0.870581	-1.782958	4.259155	-0.796549
H	-1.929031	3.607013	-1.283980	-2.413393	4.293677	0.796401	-2.299147	4.372823	0.890532
H	-2.884094	3.956638	0.161221	-0.798020	4.891760	0.378900	-0.686762	4.943912	0.422843
H	0.614913	1.019017	-2.025758	0.601606	1.859673	-2.502752	0.593561	1.944147	-2.522874
H	-0.625374	2.266211	-2.137755	-0.647247	3.059630	-2.206959	-0.609042	3.177902	-2.176963
H	-0.991907	-0.716607	-1.572278	-0.834910	0.019455	-2.035477	-0.892516	0.143576	-2.089033
H	-1.617918	0.181483	-2.959128	-1.848148	1.115839	-2.958237	-1.898661	1.302399	-2.941448
H	-3.744063	-0.890035	-2.383416	-3.565147	-0.399981	-1.948391	-3.592490	-0.224801	-1.940855
H	-5.925082	-1.551919	-2.163313	-5.324086	-1.718377	-0.782087	-5.762912	-1.290009	0.764528
H	-7.744057	0.616393	-0.964138	-6.966709	0.872826	-0.937191	-6.552398	-2.258968	-1.335622
H	-8.219039	-0.594822	-2.178628	-7.463493	-0.711073	-1.591823	-4.770665	-2.261359	-1.339204
H	-7.011608	0.655349	-2.584470	-6.055868	0.140976	-2.277222	-5.672483	-1.056372	-2.296848
H	-5.966894	-2.592933	0.107062	-6.960289	-0.267008	1.376536	-6.976003	0.896303	0.678695
H	-7.603006	-2.497379	-0.590834	-5.837941	-1.611258	1.696408	-7.837192	-0.350548	-0.258156
H	-7.104721	-1.347464	0.669634	-7.276860	-1.864411	0.676517	-6.867080	0.890763	-1.095600
H	-6.027617	0.792367	0.843701	-5.011459	0.751771	1.929325	-4.893255	0.750695	2.035519
H	-4.076824	2.074999	1.540148	-3.054089	2.160163	2.229611	-2.922857	2.160362	2.321805

	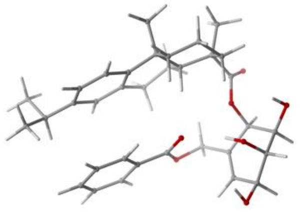 conformer **3-dias-VII**			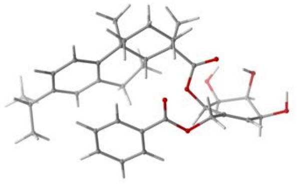 conformer **3-dias-VIII**			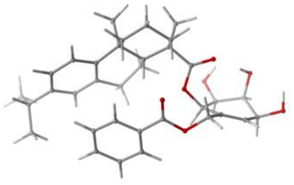 conformer **3-dias-IX**		
C	2.198983	4.040982	-0.740011	1.396428	3.483324	-0.578977	-1.358923	-2.802208	2.130615
C	1.956295	2.632035	-0.188569	1.040139	2.238576	0.254099	-1.007502	-1.406615	1.593199
C	2.848157	1.675129	-0.987739	2.201761	1.259942	0.052412	-2.204244	-0.944128	0.753794
O	2.841992	0.444430	-0.428361	1.838589	0.014338	-0.247066	-1.862052	-0.252164	-0.328357
C	3.652829	-0.592088	-0.996705	2.827365	-0.970129	-0.590811	-2.857822	0.260748	-1.224992
H	4.045049	-0.218109	-1.954564	2.180650	-1.841788	-0.759019	-2.221223	0.806628	-1.934455
C	2.755978	-1.792449	-1.198984	3.732405	-1.318542	0.568524	-3.758029	1.290064	-0.579261
C	1.546119	-1.551866	-2.068003	3.060204	-1.423511	1.911276	-3.113518	2.221903	0.409475
O	0.336464	-2.149575	-1.575719	1.831589	-2.143956	1.742612	-1.794426	2.532498	-0.053922
C	-0.092091	-1.804555	-0.348275	0.689593	-1.525116	2.098338	-0.904494	2.876661	0.894021
C	-1.448242	-2.332990	-0.049810	-0.509151	-2.174984	1.505529	0.501817	2.792735	0.416151
C	-1.998283	-2.032130	1.198789	-0.406241	-3.266511	0.636451	0.836329	2.193881	-0.802627
C	-3.266837	-2.495939	1.526182	-1.555224	-3.799774	0.059701	2.173480	2.058735	-1.163821
C	-3.989799	-3.255250	0.606630	-2.802885	-3.245468	0.347416	3.176508	2.523578	-0.314847
C	-3.447068	-3.545387	-0.645655	-2.906628	-2.160764	1.217261	2.844103	3.128508	0.897409
C	-2.176166	-3.085686	-0.977018	-1.761814	-1.626996	1.796717	1.509442	3.260529	1.265529
O	0.547989	-1.128004	0.429255	0.650041	-0.540032	2.799898	-1.225774	3.174213	2.021969
C	2.955694	-2.954972	-0.573531	5.025413	-1.598996	0.395419	-5.048088	1.397004	-0.904216
C	3.979681	-3.162918	0.517218	5.745418	-1.480805	-0.924146	-5.770601	0.439143	-1.817159
H	4.873264	-3.671867	0.117878	5.731019	-2.465144	-1.425385	-5.749230	0.836801	-2.847401
O	3.459960	-3.992979	1.529066	7.099187	-1.141970	-0.747236	-7.126751	0.314983	-1.464101
C	4.381950	-1.803648	1.095860	5.067455	-0.448227	-1.829646	-5.102829	-0.938536	-1.796766
H	5.215415	-1.936587	1.799630	5.501970	-0.529216	-2.837028	-5.546034	-1.553587	-2.593862
O	3.319537	-1.263685	1.872914	5.379671	0.861081	-1.395747	-5.410421	-1.613488	-0.592081
C	4.816411	-0.853890	-0.015157	3.553882	-0.657341	-1.949265	-3.591089	-0.872793	-2.032294
H	5.636008	-1.329399	-0.577083	3.399268	-1.546643	-2.581093	-3.437197	-0.632190	-3.096257
O	5.322853	0.349751	0.496034	2.970659	0.429768	-2.619230	-3.017234	-2.134367	-1.809848
O	3.444045	1.936424	-2.002242	3.364207	1.590363	0.205020	-3.360748	-1.125846	1.090623
C	2.353422	2.539502	1.303824	1.084799	2.558373	1.772237	-0.941699	-0.370525	2.746264
C	1.316285	3.167291	2.228163	-0.154619	3.297877	2.260054	0.347027	-0.469328	3.554263
C	-0.056255	2.524165	2.033631	-1.430147	2.527312	1.923612	1.582803	-0.339108	2.665343
C	-0.584042	2.662075	0.589163	-1.598142	2.298596	0.406711	1.636383	-1.407830	1.551779
C	-0.983795	4.130235	0.327910	-1.921095	3.648179	-0.274308	1.963858	-2.780768	2.176928
C	0.499764	2.092619	-0.371524	-0.316554	1.581829	-0.117644	0.297915	-1.337410	0.759709
H	0.569434	1.032482	-0.084484	-0.316832	0.622109	0.424399	0.302792	-0.317377	0.342318
C	0.011063	2.127403	-1.820178	-0.464924	1.241880	-1.603306	0.322865	-2.290866	-0.436094
C	-1.176141	1.183667	-1.994052	-1.540383	0.174432	-1.753720	1.336170	-1.773903	-1.448267
C	-2.150533	1.196091	-0.835151	-2.798573	0.480014	-0.971903	2.636733	-1.320131	-0.821878
C	-3.365445	0.523417	-0.992716	-3.969800	-0.209588	-1.298076	3.724198	-1.067393	-1.668995
C	-4.335686	0.464607	0.006851	-5.161038	-0.050291	-0.590127	4.950986	-0.603883	-1.204858
C	-5.660479	-0.225306	-0.277559	-6.425214	-0.794583	-0.983035	6.094673	-0.320871	-2.162620
C	-6.512495	0.631101	-1.225765	-6.258497	-2.312956	-0.855832	7.361695	-1.099880	-1.793813
C	-6.459381	-0.583101	0.975006	-6.889164	-0.406893	-2.391842	6.376831	1.183621	-2.257481
C	-4.040374	1.093752	1.218216	-5.148334	0.828283	0.494818	5.078072	-0.396271	0.173708
C	-2.830029	1.756543	1.395692	-3.995251	1.534674	0.823284	4.012326	-0.646823	1.026419
C	-1.863592	1.831179	0.385596	-2.805100	1.391582	0.100060	2.771956	-1.103596	0.557862
H	1.837243	4.143575	-1.771702	1.295750	3.288774	-1.657033	-2.383365	-2.799172	2.525248
H	3.276397	4.258644	-0.752039	2.435592	3.775924	-0.377474	-0.683089	-3.102722	2.938895
H	1.707216	4.803345	-0.124629	0.750974	4.331940	-0.326619	-1.299707	-3.564023	1.339313
H	1.664892	-1.988003	-3.068335	2.836244	-0.432759	2.331734	-3.035397	1.747137	1.400496
H	1.375544	-0.471613	-2.182105	3.687898	-1.974140	2.624221	-3.699900	3.144225	0.529288
H	-1.422102	-1.422808	1.894756	0.573798	-3.690749	0.419625	0.045688	1.817840	-1.450944
H	-3.696313	-2.258135	2.500480	-1.477948	-4.649941	-0.620097	2.439468	1.570843	-2.102240

H	-4.987776	-3.616078	0.863539	-3.701411	-3.657022	-0.115016	4.222804	2.396774	-0.594613
H	-4.017197	-4.134746	-1.365630	-3.877821	-1.709051	1.423032	3.630928	3.487463	1.562681
H	-1.741502	-3.306879	-1.951264	-1.822348	-0.770234	2.467645	1.224686	3.710581	2.217278
H	2.270703	-3.791827	-0.739720	5.634610	-1.942783	1.236613	-5.656079	2.209608	-0.495927
H	2.940923	-3.401592	2.095042	7.102816	-0.175443	-0.670080	-7.136691	-0.344530	-0.753469
H	2.549663	-1.106360	1.297746	4.822693	1.066087	-0.616705	-4.842600	-1.250803	0.118057
H	4.674295	0.651620	1.147160	3.539840	1.190715	-2.424763	-3.578811	-2.561401	-1.144156
H	3.339449	3.012961	1.445712	1.999950	3.129825	1.988323	-1.824723	-0.491625	3.392230
H	2.455558	1.479577	1.580626	1.151250	1.609027	2.325818	-0.996902	0.644836	2.322078
H	1.257490	4.255752	2.065644	-0.194998	4.313987	1.834911	0.375053	-1.417485	4.115572
H	1.637043	3.034346	3.272935	-0.083727	3.429389	3.350818	0.353791	0.331216	4.309753
H	-0.765678	2.974700	2.744128	-2.295606	3.072772	2.328947	2.482099	-0.398345	3.297031
H	0.010342	1.450468	2.280518	-1.395330	1.548243	2.430453	1.589120	0.658311	2.194819
H	-1.816209	4.402515	0.994652	-1.177348	4.426317	-0.063102	1.286105	-3.050223	2.996830
H	-0.165393	4.836870	0.514839	-2.008501	3.548220	-1.364948	1.937128	-3.590000	1.433904
H	-1.335762	4.283364	-0.701987	-2.892155	4.013572	0.092959	2.983932	-2.756494	2.589504
H	0.815889	1.840160	-2.515374	0.480718	0.877892	-2.025439	-0.664718	-2.357325	-0.913210
H	-0.273835	3.151307	-2.106196	-0.741873	2.138711	-2.179325	0.592217	-3.309439	-0.115787
H	-0.811184	0.148725	-2.115380	-1.140553	-0.786017	-1.377452	0.893029	-0.908145	-1.974197
H	-1.715862	1.413756	-2.926120	-1.790684	0.012458	-2.813603	1.541854	-2.529369	-2.222514
H	-3.560048	0.024205	-1.947965	-3.934233	-0.905135	-2.142307	3.596031	-1.243575	-2.742317
H	-5.418580	-1.166872	-0.800851	-7.213383	-0.486593	-0.275283	5.772171	-0.662528	-3.160777
H	-6.766338	1.593397	-0.752059	-5.964649	-2.597344	0.166530	8.150669	-0.937159	-2.544646
H	-7.452339	0.116802	-1.482384	-5.486110	-2.684929	-1.548885	7.159535	-2.179664	-1.734102
H	-5.976489	0.850657	-2.161035	-7.199581	-2.832024	-1.096196	7.760855	-0.777886	-0.818527
H	-5.858346	-1.163642	1.690204	-7.031974	0.680742	-2.475740	6.689755	1.588610	-1.280746
H	-7.342348	-1.182276	0.704999	-7.841612	-0.901122	-2.640249	5.481589	1.736204	-2.582568
H	-6.824241	0.318956	1.492537	-6.147849	-0.707415	-3.149938	7.183562	1.389139	-2.978548
H	-4.755788	1.076712	2.041768	-6.057509	0.978569	1.082836	6.020430	-0.034623	0.592188
H	-2.646601	2.243282	2.354566	-4.039054	2.233080	1.660225	4.155610	-0.478631	2.094653

	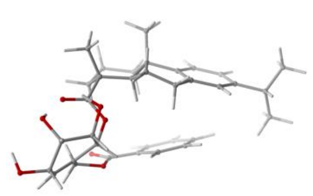 conformer **3-dias-X**			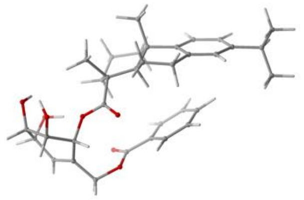 conformer **3-dias-XI**		
C	1.371760	-2.861990	0.347567	1.403446	-2.872428	0.256229
C	0.808789	-1.479629	-0.041525	0.826451	-1.476278	-0.055241
C	1.551448	-0.466009	0.840614	1.606906	-0.496689	0.832884
O	2.818872	-0.286868	0.400679	2.854735	-0.299836	0.346480
C	3.717327	0.498872	1.216715	3.787832	0.457186	1.150802
H	3.149557	0.866733	2.079220	3.258214	0.794905	2.049100
C	4.262072	1.621728	0.370253	4.297243	1.609090	0.321484
C	3.662566	2.990016	0.535262	3.706430	2.970264	0.559816
O	2.230343	2.934158	0.543962	2.275705	2.912458	0.624806
C	1.636076	2.654881	-0.633079	1.633656	2.681166	-0.537557
C	0.157310	2.533241	-0.510709	0.161204	2.552701	-0.359067
C	-0.577372	2.348621	-1.687072	-0.441174	2.535064	0.903171
C	-1.957718	2.187901	-1.628598	-1.819838	2.369372	1.006222
C	-2.610214	2.208854	-0.396089	-2.598985	2.219751	-0.140792
C	-1.880448	2.408690	0.775446	-1.999702	2.254028	-1.399736
C	-0.498376	2.569971	0.724331	-0.622998	2.418715	-1.510081
O	2.251304	2.514071	-1.666184	2.205868	2.584059	-1.599898
C	5.210302	1.345944	-0.530034	5.205313	1.365013	-0.628007
C	5.631919	-0.074273	-0.797047	5.613628	-0.045057	-0.961553
H	6.613784	-0.105879	-1.287184	6.572753	-0.059335	-1.495497
O	4.730251	-0.643511	-1.750028	4.670301	-0.582443	-1.892370
C	5.687255	-0.965096	0.481062	5.724600	-0.978487	0.281924
H	6.733831	-1.025693	0.816580	6.784612	-1.047474	0.570343
O	5.276135	-2.275970	0.153892	5.301680	-2.278609	-0.072098
C	4.838811	-0.433037	1.672817	4.926843	-0.489537	1.526268
H	5.495133	0.173339	2.320509	5.610059	0.094247	2.166850
O	4.328513	-1.497225	2.420883	4.447572	-1.579621	2.257301
O	1.114019	0.114668	1.801988	1.210993	0.046682	1.833218
C	1.156644	-1.166894	-1.516998	1.112270	-1.106808	-1.531135
C	0.220635	-1.840546	-2.509969	0.132887	-1.739215	-2.509314
C	-1.226679	-1.443111	-2.250575	-1.301043	-1.351092	-2.172936
C	-1.705724	-1.853354	-0.844864	-1.720655	-1.817201	-0.765767
C	-1.888378	-3.387123	-0.813046	-1.904682	-3.350923	-0.788152
C	-0.713074	-1.293447	0.223355	-0.682494	-1.301096	0.280982
H	-0.858040	-0.199731	0.181025	-0.827126	-0.206727	0.288681
C	-1.169666	-1.743358	1.614348	-1.080806	-1.806495	1.671002
C	-2.487784	-1.056575	1.956388	-2.383426	-1.136033	2.095488
C	-3.441217	-0.908041	0.789690	-3.383861	-0.941688	0.976341
C	-4.701243	-0.357192	1.040426	-4.635523	-0.404106	1.301693
C	-5.632421	-0.114420	0.030345	-5.605947	-0.119511	0.344973
C	-6.979999	0.517499	0.330757	-6.938920	0.492228	0.737111
C	-6.817607	1.962330	0.819061	-8.119776	-0.384439	0.306002
C	-7.795660	-0.316084	1.325021	-7.076441	1.917827	0.188700
C	-5.266735	-0.455078	-1.273482	-5.293428	-0.401108	-0.989965
C	-4.018159	-1.008499	-1.540124	-4.059336	-0.941348	-1.328678
C	-3.078795	-1.237613	-0.528566	-3.077684	-1.214452	-0.366175
H	0.989839	-3.648131	-0.317006	0.993489	-3.632266	-0.422134
H	1.113812	-3.131905	1.381158	1.187731	-3.182260	1.288292
H	2.468445	-2.850621	0.264424	2.495828	-2.857463	0.128542
H	4.010742	3.668476	-0.256139	4.022301	3.676979	-0.220301
H	3.912129	3.413323	1.517950	3.996176	3.358076	1.546119
H	-0.044170	2.322715	-2.638545	0.177366	2.616746	1.795150
H	-2.530144	2.027721	-2.543601	-2.290150	2.339140	1.990469
H	-3.688325	2.045121	-0.347319	-3.674223	2.052150	-0.054611

H	-2.392793	2.421997	1.738900	-2.610898	2.133524	-2.295570
H	0.081020	2.691505	1.637727	-0.130996	2.434851	-2.483699
H	5.578585	2.120770	-1.206557	5.544948	2.163015	-1.292442
H	3.849165	-0.300876	-1.534293	3.799147	-0.253112	-1.622085
H	4.814867	-2.178748	-0.699624	4.804572	-2.153664	-0.901526
H	4.355100	-2.254415	1.811321	4.448619	-2.315253	1.621364
H	2.191437	-1.481661	-1.714713	2.136209	-1.416376	-1.785321
H	1.114492	-0.077038	-1.679350	1.067460	-0.011341	-1.649339
H	0.334668	-2.936090	-2.466059	0.245536	-2.835795	-2.513803
H	0.510043	-1.546507	-3.530618	0.379573	-1.405062	-3.528820
H	-1.870104	-1.898440	-3.018623	-1.977185	-1.775196	-2.930655
H	-1.321363	-0.350530	-2.355342	-1.397336	-0.255132	-2.229974
H	-2.328806	-3.732580	0.131646	-2.634319	-3.615596	-1.569238
H	-2.581658	-3.684639	-1.615109	-0.971967	-3.889228	-1.006477
H	-0.946753	-3.932304	-0.966276	-2.302127	-3.734558	0.160838
H	-0.426123	-1.478858	2.373227	-0.305900	-1.571983	2.408075
H	-1.282851	-2.837314	1.642874	-1.193554	-2.900815	1.660137
H	-2.258712	-0.041495	2.324675	-2.139479	-0.136352	2.495225
H	-2.996679	-1.575221	2.784422	-2.857476	-1.688652	2.922245
H	-4.949575	-0.098777	2.074800	-4.849964	-0.190480	2.354170
H	-7.540862	0.547381	-0.618763	-6.954262	0.555072	1.838407
H	-6.258499	1.998082	1.768384	-8.026845	-1.402494	0.712749
H	-7.798812	2.432611	0.990054	-8.177861	-0.464830	-0.791526
H	-6.270477	2.571588	0.083159	-9.071471	0.042992	0.658728
H	-8.794155	0.123624	1.475003	-8.024389	2.374505	0.514286
H	-7.300903	-0.361305	2.308659	-7.063630	1.920612	-0.913473
H	-7.923346	-1.348174	0.965829	-6.251437	2.558849	0.535952
H	-5.967489	-0.285500	-2.095107	-6.020399	-0.198181	-1.780070
H	-3.775395	-1.260218	-2.572929	-3.858377	-1.148029	-2.380285

Table S16 Coordinates of compound **6**

	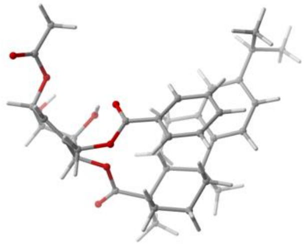 conformer **6-I**			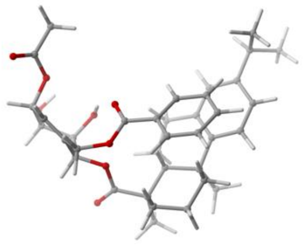 conformer **6-II**			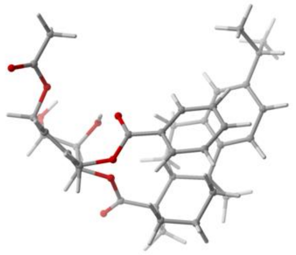 conformer **6-III**		
C	0.450035	-4.307916	-1.581546	0.450208	-4.307728	-1.581831	0.626742	-4.175939	-1.868544
C	0.162704	-3.144412	-0.623894	0.162819	-3.144306	-0.624110	0.297803	-3.100108	-0.825397
C	-1.331082	-2.831928	-0.765999	-1.330969	-2.831867	-0.766224	-1.199998	-2.808232	-0.970496
O	-1.704949	-1.757144	-0.036167	-1.704926	-1.757209	-0.036250	-1.611871	-1.804955	-0.163415
C	-3.062047	-1.332310	-0.193233	-3.062046	-1.332409	-0.193284	-2.976089	-1.399546	-0.312452
H	-3.687306	-2.235123	-0.292926	-3.687279	-2.235240	-0.292986	-3.577998	-2.305680	-0.492907
C	-3.482635	-0.568783	1.039554	-3.482589	-0.568972	1.039564	-3.435909	-0.744036	0.967539
C	-2.897209	-0.959808	2.368171	-2.897088	-0.960075	2.368126	-2.863703	-1.223453	2.272733
O	-1.489719	-0.731827	2.568201	-1.489573	-0.732194	2.568053	-1.465638	-0.979099	2.515082
C	-0.822712	0.260347	1.967532	-0.822582	0.260106	1.967566	-0.813493	0.071238	2.003762
C	0.576314	0.358451	2.469810	0.576431	0.358156	2.469891	0.576410	0.158454	2.532188
C	1.031639	-0.395724	3.557398	1.438279	1.250138	1.825427	1.428889	1.110563	1.966402
C	2.346381	-0.256177	3.991815	2.751569	1.384935	2.261812	2.734589	1.234445	2.427599
C	3.205772	0.631379	3.342709	3.205863	0.630932	3.342900	3.189893	0.412010	3.457053
C	2.751461	1.385168	2.261472	2.346467	-0.256754	3.991812	2.338816	-0.534734	4.029034
C	1.438157	1.250292	1.825145	1.031737	-0.396220	3.557346	1.032433	-0.665531	3.567568
O	-1.275379	0.998393	1.117456	-1.275237	0.998245	1.117564	-1.271407	0.864089	1.207539
C	-4.428470	0.373065	0.980197	-4.428436	0.372871	0.980321	-4.402689	0.178173	0.963590
C	-4.990863	0.916855	-0.301541	-4.990934	0.916737	-0.301336	-4.953879	0.809474	-0.282390
H	-6.076717	1.067608	-0.226099	-6.076789	1.067428	-0.225806	-6.043697	0.933412	-0.216473
O	-4.389740	2.215135	-0.531248	-4.389897	2.215066	-0.530988	-4.374741	2.132733	-0.398420
C	-4.890655	3.247571	0.175256	-4.890973	3.247477	0.175435	-4.908187	3.096923	0.377366
C	-4.170972	4.522028	-0.167592	-4.171520	4.522028	-0.167544	-4.204410	4.406168	0.153172
O	-5.785808	3.135024	0.975891	-5.786117	3.134860	0.976068	-5.816759	2.905861	1.147406
C	-4.652255	0.058247	-1.528318	-4.652363	0.058241	-1.528197	-4.576272	0.055554	-1.565127
H	-5.353378	-0.792214	-1.536435	-5.353476	-0.792227	-1.536373	-5.257828	-0.806450	-1.650653
O	-4.855912	0.766078	-2.718514	-4.856072	0.766185	-2.718318	-4.776809	0.848192	-2.701185
C	-3.223686	-0.494747	-1.461492	-3.223786	-0.494740	-1.461473	-3.136850	-0.470518	-1.515598
H	-3.074838	-1.141879	-2.337381	-3.074976	-1.141785	-2.337425	-2.959333	-1.045335	-2.435431
O	-2.270523	0.538477	-1.563584	-2.270633	0.538506	-1.563517	-2.205565	0.587444	-1.522148
O	-2.117719	-3.455878	-1.436328	-2.117549	-3.455745	-1.436685	-1.960048	-3.391859	-1.704816
C	0.429537	-3.541498	0.848203	0.429700	-3.541456	0.847953	0.548874	-3.609674	0.614456
C	1.923674	-3.648871	1.151022	1.923845	-3.648874	1.150688	2.040031	-3.711579	0.934555
C	2.685589	-2.367214	0.807610	2.685766	-2.367196	0.807358	2.780871	-2.391978	0.708101
C	2.484711	-1.901359	-0.653342	2.484800	-1.901195	-0.653533	2.593506	-1.814792	-0.714331
C	3.272653	-2.823636	-1.608368	3.272761	-2.823357	-1.608666	3.411932	-2.643719	-1.727658
C	0.951919	-1.834385	-0.909553	0.952006	-1.834248	-0.909692	1.063457	-1.755455	-0.988365
H	0.582767	-1.128173	-0.146378	0.582870	-1.128108	-0.146445	0.669294	-1.123493	-0.174158
C	0.640060	-1.178812	-2.252966	0.640100	-1.178559	-2.253042	0.757516	-0.993392	-2.275664
C	1.019983	0.293188	-2.165376	1.019899	0.293456	-2.165232	1.102074	0.472860	-2.052278
C	2.377838	0.519719	-1.539912	2.377823	0.519950	-1.539927	2.450671	0.672064	-1.397546
C	2.960615	1.790296	-1.656878	2.960605	1.790515	-1.656889	3.009070	1.955910	-1.402187
C	4.209610	2.099198	-1.128054	4.209589	2.099420	-1.128041	4.251780	2.240166	-0.837946
C	4.792070	3.495436	-1.255513	4.792018	3.495674	-1.255459	4.824720	3.646394	-0.851880
C	6.159287	3.488532	-1.947509	6.159404	3.488833	-1.947105	3.951822	4.615086	-0.044937
C	4.864744	4.187934	0.111124	4.864299	4.188242	0.111167	5.042755	4.156892	-2.280727
C	4.898558	1.075590	-0.464770	4.898517	1.075824	-0.464734	4.952766	1.177691	-0.260847
C	4.328545	-0.180869	-0.326508	4.328529	-0.180655	-0.326538	4.404086	-0.098122	-0.231282
C	3.058197	-0.485583	-0.838420	3.058215	-0.485383	-0.838505	3.144542	-0.379111	-0.775996

H	-0.338372	-5.066227	-1.492710	-0.338061	-5.066174	-1.492928	-0.149028	-4.952116	-1.859613
H	1.409346	-4.787327	-1.363519	1.409625	-4.786983	-1.363960	1.589991	-4.655453	-1.669136
H	0.464336	-3.972973	-2.628525	0.464302	-3.972734	-2.628793	0.656671	-3.755841	-2.884025
H	-3.002549	-2.041184	2.535272	-3.002478	-2.041448	2.535201	-2.945257	-2.316654	2.354098
H	-3.427687	-0.429446	3.172384	-3.427484	-0.429713	3.172392	-3.420225	-0.769165	3.105382
H	0.353330	-1.087763	4.055070	1.070197	1.823753	0.974789	1.061313	1.738844	1.155086
H	2.703746	-0.844258	4.838714	3.425318	2.066032	1.742506	3.402707	1.959824	1.963770
H	4.238958	0.733344	3.679983	4.239039	0.732831	3.680213	4.217406	0.505910	3.813418
H	3.425206	2.066141	1.741987	2.703828	-0.845009	4.838588	2.696726	-1.176760	4.835549
H	1.070056	1.823764	0.974414	0.353420	-1.088360	4.054859	0.360671	-1.404376	4.003086
H	-4.751972	0.881360	1.891363	-4.751884	0.881101	1.891538	-4.753531	0.606246	1.905362
H	-4.619214	5.355823	0.382571	-4.619392	5.355665	0.383151	-4.232039	4.670499	-0.912998
H	-4.225178	4.704634	-1.249798	-4.226572	4.704930	-1.249660	-3.148449	4.301861	0.441648
H	-3.108996	4.420047	0.098581	-3.109342	4.420002	0.097774	-4.683144	5.187415	0.752569
H	-4.150170	1.428466	-2.735718	-4.150375	1.428621	-2.735472	-4.083311	1.522178	-2.658170
H	-2.113504	0.907142	-0.677803	-2.113657	0.907172	-0.677728	-2.074823	0.893642	-0.608763
H	-0.076428	-4.494761	1.073959	-0.076272	-4.494719	1.073683	0.059898	-4.588047	0.753609
H	-0.013482	-2.772961	1.502399	-0.013276	-2.772944	1.502202	0.078734	-2.905560	1.320248
H	2.357622	-4.506888	0.612013	2.357749	-4.506850	0.611579	2.500196	-4.515175	0.336637
H	2.058010	-3.869418	2.221928	2.058232	-3.869521	2.221563	2.161354	-4.014636	1.986665
H	3.755064	-2.525795	1.015567	3.755251	-2.525800	1.015230	3.850082	-2.544943	0.921837
H	2.352034	-1.558726	1.475668	2.352263	-1.558773	1.475517	2.419175	-1.646154	1.432170
H	3.013199	-2.645101	-2.661673	4.349322	-2.623931	-1.502921	4.482822	-2.432935	-1.591733
H	3.118568	-3.888410	-1.397101	3.013071	-2.644965	-2.661931	3.161653	-2.387911	-2.767210
H	4.349239	-2.624419	-1.502423	3.118926	-3.888141	-1.397269	3.276943	-3.724508	-1.602856
H	-0.431155	-1.249716	-2.491456	-0.431098	-1.249547	-2.491561	-0.307795	-1.065722	-2.538465
H	1.183330	-1.675814	-3.072371	1.183450	-1.675399	-3.072492	1.325608	-1.404693	-3.125246
H	0.253011	0.804553	-1.560121	0.252991	0.804593	-1.559705	0.319321	0.908913	-1.409240
H	0.989975	0.766491	-3.158929	0.989641	0.766992	-3.158661	1.067204	1.033681	-2.998924
H	2.407126	2.564780	-2.197803	2.407138	2.564994	-2.197837	2.439621	2.758063	-1.882091
H	4.100846	4.077863	-1.887811	4.100917	4.078033	-1.887952	5.811507	3.597430	-0.360744
H	6.102416	3.000579	-2.931930	6.102817	3.000805	-2.931501	3.824561	4.269820	0.992747
H	6.909596	2.948759	-1.347463	6.909610	2.949175	-1.346834	2.948713	4.710239	-0.491374
H	6.527545	4.516301	-2.092183	6.527599	4.516620	-2.091759	4.402794	5.619533	-0.017345
H	5.535109	3.641531	0.795183	5.250613	5.215062	0.013680	5.685456	3.470937	-2.852464
H	3.870990	4.241110	0.582207	5.534527	3.641911	0.795413	5.518283	5.150294	-2.272449
H	5.251114	5.214732	0.013579	3.870422	4.241352	0.581989	4.086107	4.248703	-2.819987
H	5.888653	1.261648	-0.041466	5.888593	1.261883	-0.041397	5.937920	1.352745	0.179608
H	4.892936	-0.949760	0.204142	4.892928	-0.949553	0.204087	4.978993	-0.898709	0.237782

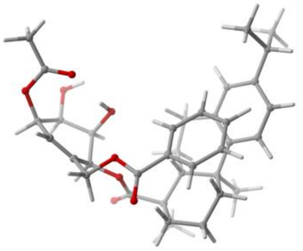

conformer **6-IV**

C	0.633134	-3.792262	-2.554474
C	0.300599	-2.869379	-1.375550
C	-1.171669	-2.479468	-1.528228
O	-1.609220	-1.711448	-0.501770
C	-2.905741	-1.137896	-0.659146
H	-3.520599	-1.841121	-1.244542
C	-3.529959	-0.894380	0.691800
C	-2.949173	-1.567356	1.897240
O	-1.748711	-0.846907	2.195342
C	-0.780756	-1.515497	2.841195
C	0.481843	-0.730755	2.891068
C	1.493913	-1.164203	3.751471
C	2.702085	-0.475323	3.799800
C	2.902146	0.634773	2.978699
C	1.897486	1.059215	2.109625
C	0.682965	0.381946	2.066120
O	-0.929806	-2.621841	3.308116
C	-4.540300	-0.028191	0.786617
C	-5.128574	0.701770	-0.389813
H	-6.057745	0.188407	-0.677161
O	-5.626489	1.993018	-0.015837
C	-4.768139	2.902333	0.457913
C	-5.432341	4.236900	0.623440
O	-3.605027	2.676629	0.718730
C	-4.205063	0.766211	-1.670418
H	-4.701098	0.155663	-2.441027
O	-4.091131	2.066696	-2.176100
C	-2.805107	0.174259	-1.437148
H	-2.361409	-0.036707	-2.420348
O	-1.953031	1.115794	-0.826827
O	-1.882710	-2.773080	-2.459308
C	0.498989	-3.590412	-0.023453
C	1.973941	-3.745409	0.341141
C	2.714143	-2.406647	0.350901
C	2.605315	-1.636749	-0.985418
C	3.483824	-2.325581	-2.051220
C	1.095168	-1.528290	-1.338830
H	0.655925	-0.983515	-0.485461
C	0.883506	-0.635412	-2.560773
C	1.222332	0.799757	-2.179000
C	2.508294	0.912556	-1.389235
C	3.053094	2.187429	-1.195551
C	4.226458	2.404062	-0.474504
C	4.767067	3.803006	-0.238130
C	3.847024	4.591509	0.702541
C	5.007203	4.563290	-1.546540
C	4.874519	1.281850	0.047896
C	4.343100	0.010755	-0.129523
C	3.148228	-0.205086	-0.826978
H	1.592709	-4.299517	-2.409050
H	0.674938	-3.241334	-3.504250

H	-0.144056	-4.561375	-2.658687
H	-2.704308	-2.623519	1.718757
H	-3.633431	-1.506391	2.756098
H	1.316470	-2.045613	4.368950
H	3.493891	-0.809498	4.472426
H	3.855865	1.165206	2.997735
H	2.076098	1.903698	1.442640
H	-0.102369	0.687505	1.374032
H	-5.015888	0.159563	1.753032
H	-4.797243	4.893682	1.226786
H	-6.424623	4.121780	1.078100
H	-5.570673	4.673084	-0.377101
H	-3.238435	2.392186	-1.845685
H	-2.389261	1.452335	-0.020827
H	0.005942	-4.575897	-0.053538
H	0.002786	-3.011356	0.768236
H	2.465976	-4.449203	-0.350366
H	2.045790	-4.202603	1.340131
H	3.770350	-2.589302	0.601699
H	2.304834	-1.772512	1.152125
H	3.302705	-3.404992	-2.120780
H	4.543807	-2.188406	-1.789875
H	3.340360	-1.891613	-3.050993
H	-0.157150	-0.683843	-2.915402
H	1.506135	-0.971627	-3.404612
H	0.393098	1.200708	-1.571128
H	1.289201	1.437375	-3.074692
H	2.522948	3.040154	-1.630919
H	5.742314	3.690408	0.265439
H	4.263607	5.588097	0.918489
H	3.709073	4.063131	1.658593
H	2.851351	4.732855	0.251070
H	5.467515	5.543791	-1.347524
H	4.062659	4.743910	-2.084478
H	5.672964	3.999095	-2.216599
H	5.805477	1.404587	0.607806
H	4.875173	-0.838102	0.302939

Table S17 Coordinates of compound **6-dias**

	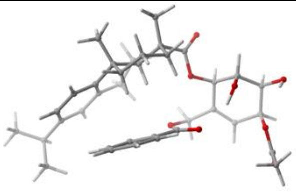			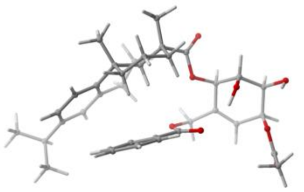			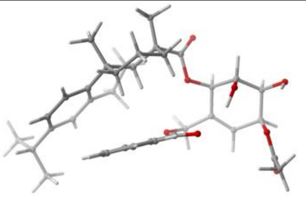		
	conformer **6-dias-I**			conformer **6-dias-II**			conformer **6-dias-III**		
C	-0.309322	4.393186	0.353871	-0.309210	4.393124	0.354014	-0.375465	4.400753	0.104254
C	-0.033141	2.884784	0.394370	-0.033041	2.884714	0.394414	-0.082523	2.901432	0.244962
C	1.216915	2.652089	-0.459343	1.216981	2.652036	-0.459354	1.141551	2.617312	-0.630935
O	1.551392	1.345277	-0.532859	1.551467	1.345234	-0.532903	1.488129	1.311497	-0.621013
C	2.796543	1.051334	-1.173333	2.796615	1.051303	-1.173384	2.718805	0.984122	-1.273668
H	2.885694	1.708075	-2.055174	2.885730	1.708000	-2.055262	2.775373	1.573910	-2.204121
C	2.776755	-0.387704	-1.628807	2.776843	-0.387754	-1.628828	2.705017	-0.484854	-1.621029
C	1.464671	-0.992181	-2.044889	1.464761	-0.992200	-2.044965	1.389924	-1.132442	-1.954156
O	0.490826	-1.227712	-1.007843	0.490877	-1.227743	-1.007981	0.448783	-1.302245	-0.874928
C	0.823938	-1.464597	0.267032	0.823915	-1.464552	0.266929	0.819427	-1.439435	0.404197
C	-0.370559	-1.795740	1.093239	-0.370607	-1.795807	1.093046	-0.348601	-1.718737	1.285382
C	-1.619924	-2.065865	0.527703	-0.202150	-1.847509	2.481194	-1.611189	-2.034041	0.775486
C	-2.694597	-2.390704	1.350171	-1.283242	-2.154169	3.300092	-2.661502	-2.299978	1.648673
C	-2.529182	-2.427563	2.733521	-2.529357	-2.427639	2.733147	-2.458403	-2.236604	3.026021
C	-1.283032	-2.154041	3.300367	-2.694675	-2.390732	1.349787	-1.198224	-1.920502	3.536972
C	-0.202005	-1.847387	2.481379	-1.619938	-2.065890	0.527411	-0.141794	-1.669551	2.668403
O	1.947375	-1.418814	0.722014	1.947317	-1.418650	0.721983	1.954030	-1.348136	0.823229
C	3.902183	-1.099070	-1.736121	3.902255	-1.099152	-1.736093	3.835925	-1.190375	-1.706901
C	5.223621	-0.653155	-1.179336	5.223685	-0.653235	-1.179282	5.166423	-0.690953	-1.221909
H	6.055771	-0.937045	-1.838183	6.055856	-0.937170	-1.838082	5.983561	-1.013904	-1.881565
O	5.414053	-1.339207	0.083668	5.414077	-1.339188	0.083773	5.400167	-1.280163	0.081879
C	5.776799	-2.635068	0.022529	5.776674	-2.635088	0.022759	5.779964	-2.572357	0.105414
C	5.880457	-3.219943	1.403244	5.880228	-3.219878	1.403516	5.929306	-3.052826	1.521766
O	5.966978	-3.226115	-1.011790	5.966800	-3.226255	-1.011503	5.950951	-3.235311	-0.887845
C	5.273373	0.846529	-0.863840	5.273449	0.846467	-0.863893	5.205928	0.828382	-1.019023
H	5.421296	1.375785	-1.819562	5.421383	1.375670	-1.819645	5.322080	1.287179	-2.014714
O	6.366729	1.159887	-0.047565	6.366817	1.159863	-0.047641	6.316574	1.212412	-0.258145
C	3.967058	1.345458	-0.232223	3.967143	1.345477	-0.232310	3.910942	1.359707	-0.390394
H	4.054740	2.433158	-0.106130	4.054865	2.433183	-0.106310	3.988863	2.454606	-0.348238
O	3.775232	0.816495	1.058280	3.775305	0.816627	1.058231	3.760487	0.926829	0.940775
O	1.863438	3.512245	-1.006877	1.863449	3.512200	-1.006939	1.760945	3.441103	-1.259692
C	0.289144	2.403157	1.830745	0.289254	2.403008	1.830751	0.291989	2.528869	1.701009
C	-0.956896	2.349428	2.713424	-0.956766	2.349289	2.713458	-0.923567	2.530609	2.626959
C	-2.075767	1.499572	2.106338	-2.075694	1.499511	2.106371	-2.054125	1.631189	2.121400
C	-2.474966	1.943655	0.679274	-2.474890	1.943663	0.679326	-2.504461	1.967577	0.680218
C	-3.259056	3.272574	0.744700	-3.258921	3.272604	0.744822	-3.295281	3.294147	0.674515
C	-1.169991	1.984053	-0.165708	-1.169930	1.984027	-0.165665	-1.228178	1.953156	-0.208488
H	-0.766425	0.960494	-0.086408	-0.766392	0.960457	-0.086361	-0.812294	0.941360	-0.066262
C	-1.474691	2.190683	-1.648192	-1.474642	2.190633	-1.648150	-1.583584	2.045192	-1.691106
C	-2.165760	0.939728	-2.176262	-2.165689	0.939650	-2.176185	-2.276293	0.751240	-2.099574
C	-3.283259	0.462032	-1.274793	-3.283233	0.462040	-1.274721	-3.363675	0.337339	-1.131627
C	-4.188798	-0.480993	-1.782463	-4.188814	-0.480955	-1.782372	-4.275712	-0.643456	-1.538505
C	-5.252534	-0.980327	-1.038149	-5.252609	-0.980183	-1.038071	-5.316623	-1.088004	-0.724778
C	-6.194695	-2.021540	-1.614845	-6.194816	-2.021363	-1.614740	-6.281701	-2.163815	-1.190210
C	-7.652461	-1.549293	-1.599959	-7.652567	-1.549074	-1.599832	-5.561121	-3.497075	-1.424151
C	-6.038098	-3.364925	-0.891452	-6.038255	-3.364740	-0.891331	-7.061185	-1.728761	-2.436275
C	-5.409098	-0.492873	0.265825	-5.409181	-0.492661	0.265875	-5.437158	-0.502468	0.538462
C	-4.512629	0.427837	0.785971	-4.512666	0.428009	0.786005	-4.529210	0.459138	0.962675
C	-3.424353	0.912873	0.044872	-3.424338	0.912942	0.044918	-3.467603	0.888303	0.155851
H	-1.001199	4.701115	1.143928	0.629048	4.945960	0.489370	-1.036621	4.761716	0.898120
H	-0.732648	4.702531	-0.612511	-1.000828	4.701058	1.144294	-0.842297	4.634264	-0.863406
H	0.628894	4.946029	0.489522	-0.732853	4.702486	-0.612222	0.561975	4.968553	0.158370

H	1.643586	-1.956681	-2.541591	1.643688	-1.956696	-2.541670	1.567370	-2.128654	-2.384340
H	0.931132	-0.331748	-2.743920	0.931279	-0.331749	-2.744028	0.828432	-0.530707	-2.683616
H	-1.752059	-2.017925	-0.552274	0.783305	-1.638691	2.898395	-1.772796	-2.066417	-0.300993
H	-3.668316	-2.595124	0.905659	-1.155159	-2.183316	4.383349	-3.646996	-2.533439	1.246162
H	-3.378110	-2.670145	3.375518	-3.378333	-2.670232	3.375074	-3.288655	-2.433008	3.707032
H	-1.154870	-2.183142	4.383618	-3.668361	-2.595128	0.905195	-1.040223	-1.871326	4.615536
H	0.783483	-1.638553	2.898502	-1.751984	-2.017898	-0.552571	0.853380	-1.426930	3.041977
H	3.867094	-2.123765	-2.112535	3.867138	-2.123846	-2.112490	3.803124	-2.240291	-2.005981
H	6.533805	-2.598968	2.031141	4.880713	-3.221440	1.862737	6.325081	-4.073728	1.523968
H	6.265926	-4.242917	1.341253	6.533164	-2.598650	2.031583	4.943416	-3.029698	2.008974
H	4.881066	-3.221117	1.862736	6.266068	-4.242716	1.341638	6.592764	-2.380866	2.083058
H	6.128898	0.815088	0.825357	6.128994	0.815114	0.825302	6.106537	0.929682	0.643753
H	3.402311	-0.076791	0.974397	3.402313	-0.076633	0.974405	3.391579	0.028025	0.934262
H	1.050076	3.061805	2.279410	1.050221	3.061598	2.279438	1.060757	3.224296	2.074484
H	0.739655	1.400030	1.770269	0.739729	1.399871	1.770223	0.750357	1.527483	1.698769
H	-1.321862	3.370024	2.914727	-1.321682	3.369889	2.914825	-1.291380	3.560497	2.766068
H	-0.682049	1.931876	3.694750	-0.681905	1.931677	3.694750	-0.612199	2.187501	3.626082
H	-2.949171	1.532323	2.775930	-2.949083	1.532291	2.775975	-2.904934	1.706319	2.816152
H	-1.750809	0.448043	2.063861	-1.750802	0.447963	2.063863	-1.720988	0.581628	2.143869
H	-4.263609	3.084737	1.151115	-2.782729	4.017309	1.392716	-2.804405	4.085803	1.251866
H	-3.392366	3.723005	-0.249460	-4.263443	3.084800	1.151325	-4.285376	3.131662	1.124743
H	-2.782939	4.017292	1.392642	-3.392307	3.723041	-0.249324	-3.462301	3.671074	-0.344850
H	-0.552088	2.373242	-2.221433	-0.552053	2.373213	-2.221401	-0.682603	2.192686	-2.307214
H	-2.112060	3.075621	-1.802936	-2.112053	3.075540	-1.802900	-2.236590	2.909698	-1.890166
H	-1.418900	0.129446	-2.253310	-1.418836	0.129357	-2.253149	-1.523082	-0.056058	-2.134129
H	-2.558778	1.103162	-3.191794	-2.558668	1.103033	-3.191737	-2.699373	0.831322	-3.112969
H	-4.052909	-0.828545	-2.811886	-4.052911	-0.828569	-2.811770	-4.161719	-1.063523	-2.542848
H	-5.903595	-2.173857	-2.667916	-5.903744	-2.173700	-2.667816	-7.011005	-2.316317	-0.376594
H	-8.306441	-2.290437	-2.085438	-8.306564	-2.290182	-2.085339	-4.820899	-3.410068	-2.236132
H	-7.764231	-0.591260	-2.129210	-7.764318	-0.591020	-2.129042	-6.276459	-4.285320	-1.706704
H	-8.017573	-1.408979	-0.569744	-8.017679	-1.408799	-0.569614	-5.027976	-3.827969	-0.519527
H	-4.999594	-3.726522	-0.945744	-6.309448	-3.273620	0.173507	-6.387102	-1.580811	-3.295470
H	-6.690416	-4.131705	-1.338032	-4.999777	-3.726394	-0.945656	-7.593290	-0.782019	-2.260273
H	-6.309343	-3.273846	0.173377	-6.690635	-4.131492	-1.337865	-7.800959	-2.493433	-2.720616
H	-6.235504	-0.842981	0.889085	-6.235620	-0.842689	0.889128	-6.248038	-0.810388	1.203825
H	-4.663615	0.776699	1.809012	-4.663644	0.776910	1.809032	-4.652752	0.884325	1.960235

	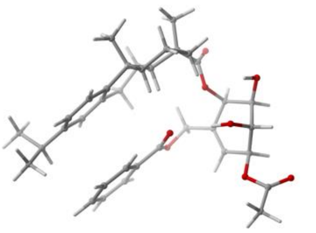 conformer **6-dias-IV**			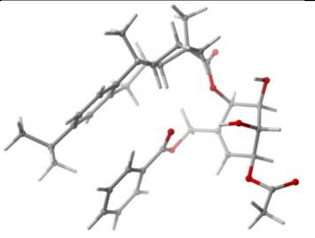 conformer **6-dias-V**			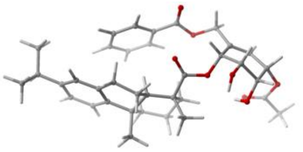 conformer **6-dias-VI**		
C	0.595243	4.825834	-0.258782	0.773648	4.899643	-0.385006	1.030302	-2.422012	1.347771
C	0.782700	3.337817	0.054721	0.893679	3.414484	-0.029999	0.451172	-1.263124	0.514718
C	1.812202	2.791871	-0.941268	1.859128	2.778619	-1.036025	1.089560	0.033438	1.027442
O	2.170318	1.533497	-0.603678	2.136801	1.503671	-0.675930	2.418356	0.019477	0.795653
C	3.165229	0.854555	-1.375500	3.060693	0.738961	-1.456781	3.206625	1.176030	1.149698
H	3.351406	1.453069	-2.280459	3.234909	1.283667	-2.397359	2.551090	1.890214	1.665281
C	2.590888	-0.499642	-1.724452	2.399679	-0.597548	-1.706272	3.766015	1.745463	-0.131977
C	1.282513	-0.472935	-2.474928	1.084041	-0.542402	-2.443657	2.978256	2.805156	-0.848789
O	0.332536	-1.456512	-2.039976	0.150900	-1.545796	-2.026958	1.625626	2.393690	-1.077856
C	-0.061746	-1.425778	-0.751956	-0.289766	-1.465504	-0.754764	0.657263	3.068196	-0.409398
C	-1.193159	-2.349581	-0.480711	-1.284703	-2.518454	-0.421821	-0.703124	2.603712	-0.795023
C	-1.677089	-2.409590	0.828599	-1.567995	-3.581748	-1.285284	-1.761480	2.899544	0.068988
C	-2.740837	-3.249830	1.135272	-2.481484	-4.559045	-0.899476	-3.045961	2.459769	-0.231231
C	-3.326945	-4.025564	0.135481	-3.118114	-4.469906	0.339259	-3.283952	1.740776	-1.403042
C	-2.853064	-3.956878	-1.175111	-2.847795	-3.399262	1.191683	-2.234738	1.473540	-2.280153
C	-1.785736	-3.119945	-1.486431	-1.931008	-2.424310	0.813954	-0.941522	1.892608	-1.973978
O	0.441962	-0.716086	0.091609	0.092500	-0.627070	0.030209	0.881902	3.937956	0.396167
C	3.142158	-1.643534	-1.315701	2.891282	-1.742983	-1.230245	4.941408	1.323300	-0.603792
C	4.306922	-1.699290	-0.373440	4.088772	-1.814040	-0.331525	5.887772	0.430742	0.150540
H	5.262978	-1.846964	-0.902765	5.006710	-2.065461	-0.888536	6.711932	1.071942	0.502654
O	4.074447	-2.834292	0.469899	3.818822	-2.868819	0.599859	6.582989	-0.505003	-0.692166
C	5.145126	-3.316428	1.134459	4.883002	-3.374366	1.257767	5.874747	-1.302710	-1.515349
C	4.741476	-4.446115	2.039355	4.445476	-4.408270	2.256336	6.789472	-2.238572	-2.253396
O	6.261040	-2.877793	1.006751	6.018337	-3.021042	1.057350	4.673271	-1.267429	-1.636585
C	4.393343	-0.407055	0.444615	4.302152	-0.479025	0.387231	5.293278	-0.256087	1.393081
H	5.315916	-0.419134	1.039502	5.252093	-0.512578	0.936120	6.111017	-0.479559	2.093555
O	3.336680	-0.259210	1.369530	3.308212	-0.203767	1.353385	4.693916	-1.505164	1.132857
C	4.452002	0.778922	-0.520232	4.380649	0.636803	-0.656863	4.304504	0.686492	2.101607
H	5.308798	0.647671	-1.199954	5.193386	0.409344	-1.364792	4.847951	1.575871	2.460998
O	4.658515	1.979085	0.172024	4.691815	1.862339	-0.053971	3.756306	0.047070	3.219789
O	2.225792	3.356972	-1.922338	2.287408	3.289881	-2.039324	0.527020	0.968078	1.541639
C	1.333656	3.130751	1.485497	1.478633	3.226654	1.389639	0.874236	-1.380431	-0.971111
C	0.263884	3.302611	2.557795	0.449010	3.478163	2.484857	0.065890	-2.422587	-1.730013
C	-0.905528	2.346038	2.328343	-0.760331	2.559017	2.321315	-1.426387	-2.114454	-1.668160
C	-1.589388	2.551935	0.959269	-1.482890	2.750560	0.970524	-1.969583	-2.071905	-0.224852
C	-2.383434	3.875812	0.977061	-2.242291	4.093818	0.982607	-2.053975	-3.517912	0.311693
C	-0.493361	2.455208	-0.141164	-0.430910	2.595898	-0.165929	-1.088892	-1.100344	0.623209
H	-0.121549	1.424066	-0.045707	-0.110306	1.547928	-0.077032	-1.282908	-0.105139	0.186160
C	-1.112387	2.575292	-1.534320	-1.091660	2.741633	-1.537073	-1.615219	-1.050883	2.059463
C	-2.024089	1.381552	-1.806779	-2.082174	1.602853	-1.768217	-2.983484	-0.378943	2.066101
C	-2.848934	0.948195	-0.613327	-2.836759	1.166685	-0.530223	-3.868592	-0.756335	0.897870
C	-3.855155	0.000868	-0.821606	-3.835138	0.197507	-0.693193	-5.178981	-0.269391	0.886644
C	-4.674140	-0.474042	0.201974	-4.567071	-0.312802	0.373353	-6.060445	-0.498937	-0.169416
C	-5.795775	-1.445855	-0.128978	-5.662232	-1.352274	0.202322	-7.465109	0.077240	-0.165812
C	-6.927708	-0.717812	-0.868757	-5.564126	-2.144696	-1.100873	-7.436773	1.610375	-0.194735
C	-6.350711	-2.194673	1.081981	-7.045139	-0.699546	0.338407	-8.291198	-0.440038	1.017025
C	-4.436009	0.017551	1.487268	-4.268879	0.183914	1.647566	-5.592862	-1.264578	-1.239615
C	-3.429461	0.951215	1.713582	-3.282055	1.144016	1.826215	-4.292585	-1.759564	-1.243246
C	-2.620007	1.442694	0.682495	-2.540439	1.654970	0.750475	-3.401203	-1.509903	-0.193264
H	1.571990	5.329477	-0.284709	0.311703	5.047950	-1.369990	0.728071	-2.349707	2.402988
H	-0.020867	5.322025	0.500221	1.771610	5.359246	-0.423517	2.128576	-2.416341	1.307251
H	0.126074	4.980554	-1.239546	0.179068	5.442090	0.360026	0.690603	-3.393944	0.969364

H	1.418482	-0.690347	-3.542352	1.211817	-0.720266	-3.519687	3.418293	3.010788	-1.834229
H	0.821907	0.520838	-2.381799	0.630227	0.449214	-2.305301	2.958902	3.731078	-0.255577
H	-1.215164	-1.781399	1.589897	-1.066590	-3.640801	-2.251065	-1.549192	3.453573	0.983425
H	-3.119353	-3.294128	2.157545	-2.700058	-5.393229	-1.568004	-3.869734	2.662688	0.455461
H	-4.165801	-4.681517	0.376710	-3.836533	-5.235995	0.637239	-4.287273	1.370008	-1.620450
H	-3.317531	-4.559912	-1.957039	-3.356696	-3.320679	2.153680	-2.424120	0.921089	-3.202085
H	-1.407023	-3.058129	-2.506153	-1.715397	-1.570615	1.457191	-0.113031	1.672477	-2.647534
H	2.681645	-2.600031	-1.576727	2.368786	-2.684612	-1.417238	5.318333	1.706566	-1.557343
H	4.164015	-5.194145	1.479337	5.325090	-4.890515	2.695068	7.653855	-1.696188	-2.658180
H	4.090079	-4.050913	2.832362	3.795850	-5.151506	1.774506	6.233035	-2.739277	-3.052512
H	5.634618	-4.899655	2.481380	3.856779	-3.915052	3.043450	7.168075	-2.988544	-1.543221
H	2.488002	-0.359862	0.906446	2.435853	-0.269193	0.933379	4.061943	-1.377685	0.408065
H	4.039591	1.953770	0.916243	4.118023	1.917867	0.723987	3.552329	-0.851434	2.920677
H	2.171148	3.828065	1.654699	2.349272	3.892427	1.512040	1.950155	-1.593351	-1.040670
H	1.735794	2.109900	1.567063	1.841128	2.193106	1.493825	0.724747	-0.398164	-1.450905
H	-0.092731	4.344981	2.589893	0.132882	4.534046	2.487971	0.270241	-3.430998	-1.333675
H	0.708571	3.104704	3.545337	0.914371	3.298299	3.466275	0.395878	-2.439740	-2.780392
H	-1.635246	2.472525	3.142417	-1.458190	2.734700	3.154210	-1.975103	-2.864997	-2.257492
H	-0.537302	1.307010	2.382410	-0.426903	1.509451	2.394630	-1.607470	-1.136782	-2.142244
H	-3.188228	3.805111	1.724719	-2.766776	4.278396	0.034576	-2.452442	-3.560378	1.334304
H	-2.862805	4.080652	0.009411	-1.587644	4.952778	1.180219	-1.084632	-4.034461	0.302770
H	-1.763942	4.742959	1.238465	-3.008325	4.069942	1.773079	-2.742671	-4.096285	-0.323471
H	-0.331423	2.628100	-2.309608	-0.336281	2.739332	-2.339105	-0.936421	-0.478819	2.702619
H	-1.680442	3.513687	-1.624108	-1.602511	3.713110	-1.616114	-1.679231	-2.065062	2.481966
H	-1.416425	0.516940	-2.126498	-1.544423	0.718406	-2.152685	-2.828463	0.713153	2.021594
H	-2.694290	1.596012	-2.654086	-2.801515	1.873132	-2.557527	-3.515091	-0.577661	3.010212
H	-4.005382	-0.379047	-1.837851	-4.023169	-0.170084	-1.704767	-5.509547	0.326804	1.743276
H	-5.369927	-2.194351	-0.819815	-5.546545	-2.069116	1.034856	-7.956780	-0.265084	-1.092215
H	-6.559336	-0.219891	-1.777925	-5.793094	-1.513090	-1.974709	-6.869156	1.982090	-1.061687
H	-7.375351	0.054758	-0.222562	-6.291463	-2.970999	-1.098532	-6.963795	2.014407	0.715303
H	-7.722456	-1.422077	-1.162173	-4.560495	-2.573028	-1.238860	-8.456993	2.021552	-0.251452
H	-6.884281	-1.515487	1.766593	-7.149379	-0.172205	1.298514	-7.860684	-0.111449	1.976795
H	-5.554354	-2.693795	1.653311	-7.844651	-1.454575	0.273212	-8.326928	-1.539693	1.024534
H	-7.071001	-2.961390	0.758064	-7.202578	0.036910	-0.466327	-9.324054	-0.060863	0.967597
H	-5.038317	-0.321030	2.331632	-4.814800	-0.193186	2.517020	-6.252535	-1.475201	-2.085426
H	-3.286869	1.315995	2.731679	-3.084907	1.502160	2.837389	-3.968474	-2.352920	-2.099044

	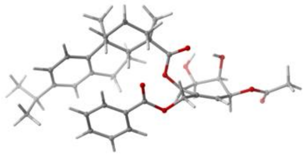 conformer **6-dias-VII**			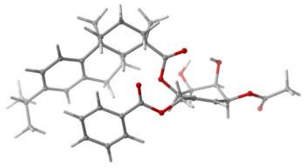 conformer **6-dias-VIII**			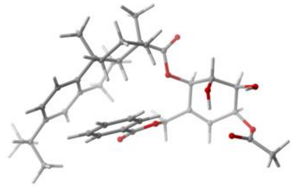 conformer **6-dias-IX**		
C	0.694993	3.596121	-0.465663	0.648967	3.563238	-0.711587	-0.265298	4.466760	-0.412184
C	0.323407	2.341418	0.345489	0.295597	2.383152	0.212145	-0.012128	2.997301	-0.053883
C	1.554097	1.430150	0.283536	1.520831	1.462945	0.191193	1.268296	2.581939	-0.786455
O	1.296109	0.169129	-0.069131	1.250751	0.176625	-0.040099	1.508065	1.256185	-0.685960
C	2.372629	-0.743829	-0.321734	2.321024	-0.757074	-0.240861	2.770328	0.805099	-1.171887
H	1.807077	-1.656364	-0.554306	1.750423	-1.686815	-0.370445	3.021405	1.390825	-2.072150
C	3.182695	-1.046091	0.917641	3.170683	-0.943895	0.995200	2.686735	-0.664684	-1.502728
C	2.399826	-1.194074	2.193886	2.427325	-0.980731	2.303176	1.342645	-1.311392	-1.637268
O	1.223047	-1.963479	1.914321	1.258830	-1.792780	2.131631	0.824846	-1.392310	-0.307074
C	0.032585	-1.405604	2.209526	0.060901	-1.217037	2.355427	-0.437177	-1.823278	-0.170276
C	-1.097877	-2.095923	1.534605	-1.055838	-1.991135	1.752541	-0.905927	-1.679242	1.234765
C	-0.893463	-3.129397	0.614807	-0.833279	-3.150491	1.001775	-0.056810	-1.209476	2.244740
C	-1.983693	-3.697498	-0.038840	-1.908473	-3.803338	0.406001	-0.541656	-1.076176	3.542339
C	-3.273556	-3.238023	0.227183	-3.201185	-3.300335	0.556296	-1.870280	-1.396370	3.828622
C	-3.479215	-2.214579	1.151432	-3.424257	-2.147415	1.307990	-2.716798	-1.848665	2.816869
C	-2.393458	-1.642944	1.802588	-2.353556	-1.494565	1.906864	-2.235014	-1.994013	1.519717
O	-0.094499	-0.439288	2.926515	-0.080820	-0.177617	2.957916	-1.093532	-2.255423	-1.091079
C	4.499525	-1.249872	0.866301	4.485865	-1.151285	0.921449	3.816675	-1.367506	-1.597795
C	5.297580	-1.090774	-0.390842	5.243832	-1.108894	-0.369376	5.187128	-0.768801	-1.434842
H	5.365642	-2.052575	-0.928694	5.298312	-2.116435	-0.817520	5.605602	-0.604327	-2.438565
O	6.617802	-0.703078	0.003468	6.574497	-0.685935	-0.054357	6.117044	-1.714525	-0.892859
C	7.599577	-0.916340	-0.896509	7.528613	-0.983245	-0.960193	5.889875	-2.208306	0.329754
C	8.911334	-0.393097	-0.384240	8.854198	-0.414548	-0.540283	7.057615	-3.018012	0.809662
O	7.420033	-1.449377	-1.963631	7.317725	-1.614437	-1.966298	4.872542	-2.024664	0.964132
C	4.685907	-0.054820	-1.339677	4.599814	-0.165917	-1.390924	5.230351	0.612466	-0.667326
H	5.215380	-0.118580	-2.299989	5.098719	-0.319509	-2.357413	5.554575	1.366246	-1.401851
O	4.853609	1.269125	-0.903546	4.778377	1.192963	-1.086120	6.167304	0.608176	0.372996
C	3.202193	-0.366414	-1.604936	3.109012	-0.499706	-1.578796	3.857976	1.044377	-0.123136
H	3.158717	-1.248995	-2.262700	3.046625	-1.438994	-2.150930	3.902601	2.121132	0.090457
O	2.625741	0.704748	-2.301090	2.508706	0.503501	-2.351808	3.584405	0.418193	1.107826
O	2.668482	1.815086	0.583266	2.644873	1.867965	0.420279	2.028620	3.331621	-1.351852
C	0.185983	2.679563	1.853637	0.206150	2.847193	1.690359	0.293575	2.829393	1.456909
C	-1.140647	3.345680	2.194869	-1.105479	3.550171	2.014815	-0.957808	2.973904	2.318914
C	-2.322267	2.489719	1.742689	-2.305566	2.670397	1.669837	-2.065016	2.007166	1.898864
C	-2.317587	2.236209	0.220421	-2.347020	2.291601	0.174590	-2.461799	2.137032	0.410660
C	-2.658796	3.554173	-0.510497	-2.696443	3.549455	-0.653415	-3.268926	3.436288	0.201469
C	-0.944624	1.602268	-0.159240	-0.991176	1.613640	-0.190992	-1.157345	2.016848	-0.432213
H	-0.942716	0.645856	0.389314	-0.977411	0.706615	0.435454	-0.759835	1.021130	-0.170925
C	-0.918185	1.248662	-1.648978	-1.010606	1.137114	-1.646157	-1.470908	1.977595	-1.926345
C	-1.891841	0.103721	-1.891824	-2.008780	-0.005921	-1.770997	-2.226577	0.694816	-2.256682
C	-3.233484	0.309490	-1.224067	-3.335398	0.283149	-1.104331	-3.269369	0.319078	-1.226590
C	-4.305079	-0.497999	-1.624235	-4.436339	-0.510168	-1.439380	-4.121149	-0.751968	-1.521856
C	-5.561016	-0.436117	-1.028921	-5.682122	-0.368970	-0.827960	-5.121653	-1.177435	-0.652641
C	-6.695528	-1.331309	-1.494185	-6.866960	-1.229834	-1.229692	-5.979991	-2.392889	-0.955305
C	-7.837715	-0.512446	-2.107217	-6.615468	-2.715692	-0.949599	-5.194054	-3.678414	-0.661343
C	-7.208725	-2.234683	-0.366789	-7.253678	-1.002794	-2.695682	-6.532475	-2.398090	-2.383398
C	-5.733212	0.483380	0.012058	-5.800012	0.602179	0.168072	-5.268726	-0.477535	0.549435
C	-4.684125	1.300599	0.412835	-4.718215	1.411017	0.502972	-4.416830	0.571230	0.866443
C	-3.418363	1.239520	-0.188374	-3.473231	1.284002	-0.124685	-3.387641	0.977925	0.005691
H	1.684511	3.956543	-0.153444	-0.061651	4.389248	-0.594602	0.671967	5.033018	-0.339670
H	-0.026993	4.404733	-0.303989	0.648561	3.260974	-1.769214	-0.995984	4.927975	0.259706
H	0.730810	3.382328	-1.544078	1.650362	3.938877	-0.461658	-0.633316	4.574857	-1.442640
H	2.098423	-0.216014	2.595177	2.119101	0.025856	2.619701	1.419216	-2.317458	-2.074074
H	2.985882	-1.721490	2.958323	3.043709	-1.425664	3.095445	0.655610	-0.714157	-2.255862

H	0.118846	-3.480686	0.416425	0.180969	-3.533064	0.890389	0.970912	-0.938157	2.002806
H	-1.826448	-4.499458	-0.761975	-1.737822	-4.706721	-0.181919	0.116875	-0.711068	4.332146
H	-4.125382	-3.674005	-0.298630	-4.041396	-3.805827	0.077625	-2.249623	-1.282879	4.846159
H	-4.485032	-1.837901	1.340247	-4.430246	-1.736669	1.403060	-3.761271	-2.077416	3.034393
H	-2.531972	-0.830999	2.516497	-2.506975	-0.583684	2.485357	-2.883981	-2.323414	0.707833
H	5.049550	-1.562834	1.757483	5.064193	-1.381613	1.819862	3.784500	-2.432791	-1.841557
H	9.107236	-0.779088	0.625289	9.082187	-0.703979	0.494486	6.764615	-3.607751	1.684393
H	8.852704	0.702820	-0.314104	8.794967	0.683124	-0.571741	7.861428	-2.319730	1.086842
H	9.714689	-0.680588	-1.070344	9.636671	-0.765612	-1.220909	7.437264	-3.664052	0.007739
H	4.290247	1.409516	-0.119244	4.241534	1.405749	-0.299497	5.644097	0.467161	1.179087
H	3.152134	1.480667	-2.044592	3.042362	1.298823	-2.185377	3.682620	-0.545208	0.989840
H	1.037139	3.307895	2.155805	1.069504	3.492007	1.912710	1.057725	3.563758	1.760118
H	0.250913	1.742526	2.428153	0.283597	1.960457	2.338231	0.727884	1.829243	1.615109
H	-1.199544	4.350841	1.746413	-1.171836	4.515650	1.487224	-1.323655	4.013371	2.286599
H	-1.193471	3.496832	3.284148	-1.124407	3.790662	3.089039	-0.692007	2.781688	3.370333
H	-3.258957	2.979738	2.048379	-3.229134	3.193494	1.960171	-2.941926	2.172865	2.543189
H	-2.276943	1.519313	2.265286	-2.251471	1.747041	2.271049	-1.730716	0.973857	2.079610
H	-3.680981	3.861361	-0.241277	-2.010643	4.386921	-0.474959	-2.788829	4.316169	0.646629
H	-2.637142	3.437202	-1.602626	-3.707822	3.889086	-0.382704	-4.255056	3.327539	0.677031
H	-1.987462	4.380063	-0.243965	-2.706499	3.342285	-1.732308	-3.446895	3.647625	-0.862657
H	0.088429	0.956557	-1.973742	-0.017333	0.800330	-1.969157	-0.544956	2.021558	-2.522562
H	-1.202961	2.121131	-2.257460	-1.296236	1.961114	-2.318479	-2.062872	2.858296	-2.220128
H	-1.454909	-0.825974	-1.482080	-1.581534	-0.903168	-1.284843	-1.518789	-0.147456	-2.327963
H	-2.036593	-0.070494	-2.969437	-2.172998	-0.273593	-2.826304	-2.706484	0.772550	-3.245008
H	-4.136479	-1.216165	-2.433170	-4.298646	-1.274433	-2.210567	-3.972359	-1.271926	-2.471712
H	-6.288845	-1.983255	-2.286083	-7.720597	-0.914238	-0.606258	-6.841175	-2.359896	-0.265942
H	-8.294429	0.152588	-1.356295	-5.774540	-3.094571	-1.553437	-5.803810	-4.572333	-0.868327
H	-8.629023	-1.172522	-2.496302	-7.503125	-3.317534	-1.200014	-4.879309	-3.717759	0.393107
H	-7.473949	0.115773	-2.933926	-6.376976	-2.886228	0.111774	-4.284666	-3.728824	-1.282188
H	-7.638794	-1.640953	0.455976	-6.443055	-1.317726	-3.372614	-7.229971	-3.238544	-2.523252
H	-6.399780	-2.851489	0.054175	-7.458468	0.060460	-2.890457	-5.728464	-2.513593	-3.127627
H	-7.995717	-2.911366	-0.735053	-8.153066	-1.582087	-2.957675	-7.068988	-1.463803	-2.606925
H	-6.699767	0.573877	0.513872	-6.755994	0.742925	0.679194	-6.052923	-0.768487	1.253684
H	-4.865882	2.017670	1.214764	-4.863797	2.176613	1.266310	-4.554456	1.080350	1.821544

	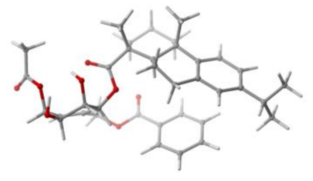 conformer **6-dias-X**		
C	-1.141043	3.325319	0.612112
C	-0.677145	2.121358	-0.227208
C	-1.803754	1.085247	-0.120438
O	-1.384126	-0.148628	0.213332
C	-2.347811	-1.185736	0.458498
H	-1.697905	-2.067452	0.554385
C	-3.237325	-1.466565	-0.730797
C	-2.528578	-1.591090	-2.053527
O	-1.272198	-2.250123	-1.849388
C	-0.154528	-1.570030	-2.173727
C	1.063449	-2.152254	-1.550290
C	2.292036	-1.546826	-1.831979
C	3.451931	-2.007158	-1.221544
C	3.387750	-3.073770	-0.325872
C	2.165997	-3.687575	-0.049074
C	1.001873	-3.229762	-0.660737
O	-0.150058	-0.583451	-2.873717
C	-4.553191	-1.656380	-0.617336
C	-5.271352	-1.383820	0.674483
H	-6.224133	-1.927782	0.727487
O	-5.557647	0.031717	0.808191
C	-6.375862	0.576957	-0.119015
C	-6.400043	2.072188	0.013159
O	-6.966603	-0.077444	-0.939816
C	-4.391397	-1.748026	1.872566
H	-4.198741	-2.830940	1.803449
O	-5.028552	-1.513628	3.095901
C	-3.046346	-0.996787	1.826632
H	-2.395195	-1.426549	2.601221
O	-3.214086	0.345290	2.220945
O	-2.965285	1.342992	-0.354645
C	-0.641213	2.479695	-1.735904
C	0.589837	3.287715	-2.125515
C	1.875360	2.560909	-1.734591
C	1.964986	2.296338	-0.216696
C	2.202233	3.637597	0.513044
C	0.683216	1.519919	0.214222
H	0.758811	0.571075	-0.342141
C	0.760624	1.159038	1.700237
C	1.854395	0.117141	1.889105
C	3.136348	0.463526	1.164291
C	4.300182	-0.236135	1.505646
C	5.514156	-0.045924	0.853185
C	6.750857	-0.831880	1.250235
C	7.851267	0.088521	1.790978
C	7.270838	-1.693085	0.093216
C	5.545011	0.893376	-0.184066
C	4.402661	1.605446	-0.526205
C	3.178748	1.414300	0.131778
H	-2.168304	3.594513	0.331823
H	-0.502755	4.200233	0.443983
H	-1.130762	3.095126	1.687572
H	-2.343418	-0.600268	-2.493157
H	-3.118870	-2.185595	-2.763234

H	2.318927	-0.704124	-2.522741
H	4.402911	-1.511630	-1.420194
H	4.296574	-3.423634	0.167894
H	2.119710	-4.524611	0.649543
H	0.042557	-3.703580	-0.453631
H	-5.149262	-1.943362	-1.484911
H	-5.413898	2.452755	-0.294089
H	-6.564963	2.364965	1.058962
H	-7.181218	2.487864	-0.631786
H	-4.902830	-0.569322	3.270305
H	-3.749569	0.775477	1.535615
H	-1.567238	3.013815	-1.996373
H	-0.630144	1.544175	-2.316270
H	0.559869	4.289917	-1.667511
H	0.579053	3.451927	-3.214093
H	2.739916	3.151364	-2.073551
H	1.912303	1.595818	-2.267527
H	2.236166	3.514185	1.604216
H	1.442313	4.393927	0.279996
H	3.177257	4.046414	0.206716
H	-0.196996	0.764378	2.063430
H	0.979166	2.052529	2.305151
H	1.495933	-0.849264	1.487777
H	2.065166	-0.048077	2.957251
H	4.241861	-0.973227	2.313006
H	6.453133	-1.512404	2.066126
H	7.484668	0.687943	2.637587
H	8.202083	0.785821	1.012946
H	8.719597	-0.497049	2.131929
H	6.494636	-2.381353	-0.275419
H	8.136497	-2.295100	0.411114
H	7.593169	-1.068016	-0.755198
H	6.472766	1.082347	-0.729709
H	4.474611	2.342256	-1.327553

Table S18 Coordinates of compound **8**

	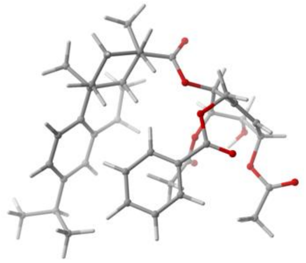			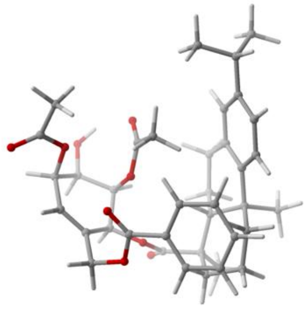			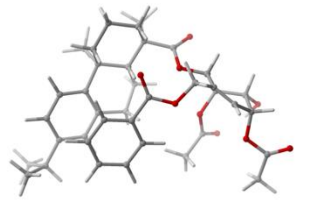		
	conformer **8-I**			conformer **8-II**			conformer **8-III**		
C	-1.554120	-4.456291	-0.091632	-1.574331	-4.282464	0.081290	0.724035	-2.359722	-1.161129
C	-1.309763	-2.970011	0.184561	-1.287510	-2.792466	0.297747	0.086041	-0.997003	-0.807754
C	-2.161887	-2.168637	-0.803591	-2.201046	-2.018813	-0.655435	0.776681	-0.587034	0.501491
O	-1.944860	-0.846443	-0.645240	-2.037375	-0.685630	-0.523283	1.931734	0.076314	0.259308
C	-2.729243	0.097336	-1.380188	-2.924932	0.172219	-1.255585	2.865745	0.239571	1.351248
H	-3.067430	-0.380032	-2.313378	-3.247037	-0.367735	-2.159034	2.308204	0.181360	2.294185
C	-1.815191	1.271988	-1.647484	-2.143584	1.405167	-1.618932	3.513274	1.586369	1.163564
C	-0.580368	0.957078	-2.455625	-0.921083	1.216424	-2.482697	2.795281	2.779403	1.742434
O	0.564385	1.727461	-2.074262	0.250180	1.265090	-1.646691	1.377582	2.607554	1.752252
C	1.039918	1.517631	-0.829215	0.822894	2.475722	-1.481769	0.722567	2.912388	0.612742
C	2.253106	2.322353	-0.529855	1.857864	2.478361	-0.414677	-0.736406	2.630219	0.714171
C	2.900112	2.078217	0.685003	2.676117	3.606577	-0.303093	-1.548484	3.012683	-0.359230
C	4.022682	2.821597	1.032649	3.613068	3.686098	0.721565	-2.914504	2.754703	-0.326282
C	4.499984	3.808637	0.169997	3.724602	2.643824	1.643795	-3.476900	2.114089	0.777971
C	3.861418	4.045299	-1.048122	2.905476	1.521293	1.535476	-2.670361	1.741640	1.852966
C	2.739625	3.300801	-1.402970	1.974920	1.434365	0.505235	-1.300971	1.995736	1.825712
O	0.524637	0.757971	-0.040779	0.514885	3.450424	-2.130860	1.269471	3.365128	-0.364885
C	-2.026831	2.476959	-1.114323	-2.421259	2.600560	-1.089927	4.693402	1.702934	0.549879
C	-3.121562	2.767990	-0.128955	-3.468211	2.825447	-0.032492	5.537034	0.541975	0.106189
H	-3.998568	3.230872	-0.611143	-4.370860	3.247110	-0.501573	6.374817	0.408643	0.812748
O	-2.565985	3.702278	0.801119	-3.092603	3.857888	0.896219	6.076242	0.902607	-1.167938
C	-3.445876	4.373469	1.573473	-1.855572	3.858115	1.433326	7.143843	0.191485	-1.587555
C	-2.724327	5.247603	2.559452	-1.685067	5.024535	2.363296	7.527403	0.585890	-2.984880
O	-4.641931	4.261198	1.469693	-1.003654	3.034873	1.194487	7.688417	-0.647552	-0.914117
C	-3.559933	1.472399	0.577544	-3.866796	1.524811	0.686694	4.761879	-0.791928	0.018457
H	-4.449163	1.678770	1.186574	-4.796990	1.698165	1.248403	5.487539	-1.614921	0.026970
O	-2.581212	0.993716	1.462760	-2.924236	1.088607	1.626885	4.059542	-0.917961	-1.190617
C	-3.944911	0.506876	-0.539614	-4.160952	0.508952	-0.415065	3.890221	-0.891493	1.268892
H	-4.669023	0.999126	-1.206522	-4.916996	0.926904	-1.098067	4.527500	-0.831605	2.164628
O	-4.548881	-0.651030	0.021406	-4.681524	-0.673226	0.178052	3.216906	-2.146631	1.252660
C	-5.481021	-1.268873	-0.750247	-5.596814	-1.356495	-0.557744	2.826545	-2.627383	2.460359
C	-5.902798	-2.567145	-0.130491	-5.904720	-2.678600	0.078134	1.956075	-3.836659	2.293272
O	-5.876782	-0.819262	-1.794754	-6.062621	-0.939539	-1.586834	3.132438	-2.111354	3.504743
O	-2.927046	-2.618241	-1.622729	-2.974205	-2.511387	-1.442790	0.409833	-0.834021	1.625557
C	-1.798658	-2.581481	1.602938	-1.647007	-2.357607	1.739416	0.430032	0.039273	-1.903056
C	-0.814796	-2.997025	2.690686	-0.595160	-2.786840	2.758346	-0.443910	-0.086576	-3.141874
C	0.572522	-2.404954	2.446193	0.803419	-2.292236	2.385091	-1.915644	0.064344	-2.782957
C	1.184721	-2.845247	1.098453	1.264299	-2.781019	0.992731	-2.392505	-1.017973	-1.796595
C	1.598045	-4.329404	1.180965	1.560077	-4.295262	1.048611	-2.487950	-2.363255	-2.551430
C	0.162211	-2.499130	-0.020425	0.171644	-2.353954	-0.028325	-1.451517	-1.042649	-0.546660
H	0.107007	-1.403037	0.018648	0.142720	-1.253147	0.047198	-1.663492	-0.090821	-0.028956
C	0.715344	-2.858881	-1.398983	0.601768	-2.667221	-1.460837	-1.907735	-2.167943	0.388465
C	1.938059	-2.001623	-1.715649	1.786631	-1.784475	-1.832847	-3.281580	-1.832293	0.961769
C	2.819760	-1.703660	-0.521527	2.823279	-1.672758	-0.736513	-4.216410	-1.131372	-0.000171
C	4.010435	-1.005599	-0.760689	4.044021	-1.068115	-1.051451	-5.511123	-0.841299	0.439592
C	4.883007	-0.638515	0.257986	5.055807	-0.878893	-0.113322	-6.429317	-0.134125	-0.336847
C	6.181504	0.107642	0.002126	6.359069	-0.195889	-0.485196	-7.815736	0.200393	0.184106
C	6.194134	0.891578	-1.310217	6.121253	1.197693	-1.077802	-7.741574	1.188600	1.354787

C	7.374576	-0.855832	0.072842	7.193582	-1.068063	-1.430772	-8.605043	-1.056048	0.567163
C	4.529586	-1.001084	1.562798	4.824549	-1.347617	1.181788	-6.012927	0.278179	-1.604263
C	3.351676	-1.690993	1.816840	3.614926	-1.946617	1.513687	-4.729869	-0.008626	-2.060033
C	2.466642	-2.052237	0.790060	2.582308	-2.102428	0.579462	-3.804288	-0.704965	-1.274754
H	-2.630478	-4.675736	-0.056490	-2.655751	-4.467494	0.138578	0.412316	-2.689346	-2.160975
H	-1.056298	-5.086474	0.654928	-1.087769	-4.900231	0.843577	0.439726	-3.139764	-0.441156
H	-1.195653	-4.753391	-1.086666	-1.238481	-4.625183	-0.906988	1.820963	-2.274403	-1.157402
H	-0.719022	1.183238	-3.521175	-0.840794	1.986741	-3.259512	3.065118	3.698055	1.204130
H	-0.346368	-0.112287	-2.349003	-0.902588	0.220265	-2.941796	3.054215	2.886667	2.805564
H	2.518551	1.290756	1.335752	2.555192	4.415636	-1.024160	-1.085266	3.504337	-1.215932
H	4.530130	2.626170	1.978638	4.255440	4.564378	0.806208	-3.546795	3.037304	-1.169272
H	5.380780	4.392552	0.443936	4.456256	2.707084	2.451851	-4.544157	1.886034	0.788297
H	4.240882	4.812646	-1.724689	2.993570	0.702930	2.249807	-3.110037	1.238400	2.715664
H	2.233506	3.476627	-2.351978	1.328620	0.563136	0.419674	-0.663411	1.683950	2.651446
H	-1.326790	3.292513	-1.312684	-1.839961	3.471914	-1.399840	5.131806	2.692128	0.387335
H	-1.978602	5.867788	2.043941	-1.989484	5.955151	1.866049	7.585883	1.678940	-3.072818
H	-2.185958	4.606661	3.272729	-0.640087	5.080526	2.684964	6.742834	0.238331	-3.672947
H	-3.446689	5.874409	3.092420	-2.338119	4.883542	3.236780	8.483887	0.122861	-3.248669
H	-1.760395	0.833077	0.972500	-2.038800	1.234153	1.260627	3.326185	-0.287760	-1.173025
H	-5.156886	-3.321882	-0.423266	-5.870854	-2.609240	1.172393	2.282018	-4.444004	1.439925
H	-6.882539	-2.861981	-0.521872	-5.125163	-3.382061	-0.252330	0.934685	-3.477309	2.093199
H	-5.916605	-2.496353	0.964126	-6.880332	-3.037308	-0.267664	1.953526	-4.421243	3.219618
H	-2.786671	-3.037506	1.778742	-2.633152	-2.771303	2.006083	1.486782	-0.064532	-2.186643
H	-1.947553	-1.492956	1.652249	-1.757386	-1.264010	1.769816	0.316742	1.056110	-1.497707
H	-0.754193	-4.095711	2.760286	-0.590461	-3.883720	2.867867	-0.265753	-1.048590	-3.649738
H	-1.191717	-2.652030	3.665872	-0.870085	-2.387349	3.746768	-0.155535	0.694237	-3.862244
H	1.234959	-2.691891	3.277275	1.514568	-2.615442	3.160383	-2.519106	0.029792	-3.702640
H	0.501557	-1.303653	2.455075	0.809404	-1.187654	2.389352	-2.072705	1.056267	-2.330221
H	0.767698	-4.988981	1.465428	1.779528	-4.707411	0.053515	-1.512167	-2.724435	-2.904229
H	2.387812	-4.444781	1.939360	0.740616	-4.879074	1.484850	-3.137471	-2.236901	-3.431477
H	2.011412	-4.694824	0.230183	2.451283	-4.466103	1.670923	-2.938489	-3.153218	-1.936002
H	-0.049866	-2.706526	-2.178525	-0.225286	-2.491753	-2.167238	-1.202935	-2.289897	1.216107
H	0.975643	-3.927349	-1.444954	0.865169	-3.731559	-1.562536	-1.936775	-3.123573	-0.155311
H	1.609504	-1.032579	-2.129878	1.420783	-0.766103	-2.045758	-3.139043	-1.160144	1.825794
H	2.543277	-2.470102	-2.507943	2.267954	-2.145165	-2.755317	-3.770010	-2.737027	1.357698
H	4.239969	-0.734797	-1.794129	4.192672	-0.724488	-2.079480	-5.797702	-1.174777	1.442141
H	6.291041	0.837175	0.824200	6.933297	-0.069140	0.448500	-8.357813	0.697454	-0.638158
H	7.090634	1.528136	-1.363898	5.599315	1.135869	-2.046377	-7.212425	2.109314	1.064238
H	5.309271	1.538379	-1.403191	7.078661	1.714131	-1.250806	-7.205109	0.747079	2.210641
H	6.222286	0.218073	-2.182270	5.505757	1.815604	-0.407877	-8.749792	1.468009	1.699123
H	7.302253	-1.615053	-0.722889	8.159297	-0.588678	-1.656556	-8.668463	-1.757437	-0.278125
H	7.407612	-1.384942	1.037099	6.665976	-1.227589	-2.385342	-9.629038	-0.793577	0.875884
H	8.325718	-0.314935	-0.055049	7.393068	-2.056018	-0.988902	-8.129759	-1.585627	1.408584
H	5.184302	-0.730076	2.395928	5.597068	-1.231852	1.946421	-6.701427	0.832514	-2.247340
H	3.115367	-1.951293	2.849408	3.474281	-2.297215	2.537829	-4.449229	0.331291	-3.057191

	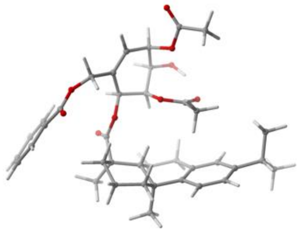 conformer **8-IV**			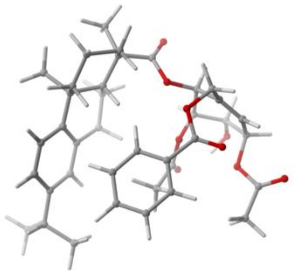 conformer **8-V**			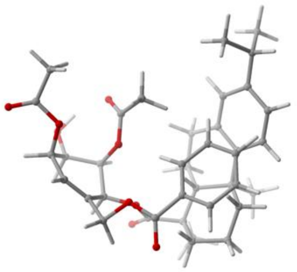 conformer **8-VI**		
C	-2.507960	-2.991274	-0.965050	-1.748768	4.735279	-0.738297	1.295197	-3.480152	-2.995364
C	-1.672623	-1.953610	-0.208619	-1.130831	3.490395	-0.092751	0.881305	-2.745394	-1.715017
C	-1.939038	-0.591565	-0.845357	0.301717	3.377367	-0.621406	-0.640457	-2.593154	-1.765005
O	-1.478524	0.415480	-0.062914	0.948569	2.317868	-0.079698	-1.138940	-2.023243	-0.636358
C	-1.265848	1.673105	-0.696464	2.239764	2.014553	-0.604321	-2.516878	-1.655800	-0.685338
H	-1.940978	1.741657	-1.562187	2.630898	2.929847	-1.073200	-3.028662	-2.365504	-1.352618
C	-1.564896	2.786914	0.282720	3.158484	1.558889	0.511568	-3.121011	-1.709115	0.703165
C	-2.677275	2.547930	1.264049	2.826639	1.978615	1.918239	-2.473612	-2.605758	1.718791
O	-3.756009	1.876723	0.603365	1.746788	1.143877	2.378563	-1.381139	-1.830926	2.227120
C	-4.310377	0.844828	1.272068	2.081160	-0.116236	2.717492	-0.291372	-2.476884	2.663622
C	-5.181804	0.004273	0.404858	0.905623	-1.029942	2.713810	0.840805	-1.526639	2.852081
C	-5.309998	0.229858	-0.968067	1.080521	-2.333699	3.188500	1.959520	-1.946746	3.573498
C	-6.079244	-0.634803	-1.741844	0.022470	-3.236774	3.140987	3.042415	-1.085058	3.730990
C	-6.723196	-1.719489	-1.148180	-1.208752	-2.838475	2.616879	3.014075	0.182721	3.149982
C	-6.602376	-1.942113	0.224911	-1.381928	-1.541251	2.135325	1.904468	0.594641	2.410893
C	-5.832879	-1.081979	1.000316	-0.324701	-0.637143	2.181862	0.812984	-0.254994	2.267121
O	-4.094410	0.613677	2.438685	3.209527	-0.468136	2.971583	-0.241494	-3.669663	2.854632
C	-0.854761	3.920332	0.291941	4.168039	0.714464	0.272526	-4.134445	-0.906289	1.043034
C	0.334789	4.169081	-0.596557	4.401319	0.044850	-1.054353	-4.719499	0.141934	0.141052
H	0.282269	5.182600	-1.018034	5.465086	0.068073	-1.327721	-5.816736	0.089900	0.121313
O	1.565200	4.079530	0.153076	4.007715	-1.339205	-0.938237	-4.357106	1.429119	0.674477
C	1.937661	5.175613	0.841137	4.902854	-2.182790	-0.384403	-5.328726	2.377321	0.723785
C	3.268353	4.952518	1.505749	4.315158	-3.560845	-0.259182	-4.713487	3.721462	0.993877
O	1.272118	6.178781	0.908028	6.002063	-1.845991	-0.024591	-6.499367	2.149823	0.576270
C	0.426280	3.147422	-1.733806	3.561853	0.662232	-2.175789	-4.178751	0.051647	-1.294465
H	-0.436639	3.354480	-2.391712	3.992452	1.663940	-2.352635	-4.642636	-0.843397	-1.745903
O	1.564275	3.295672	-2.521336	3.662687	-0.018087	-3.384960	-4.560998	1.131381	-2.085509
C	0.179002	1.736097	-1.189194	2.132227	0.934891	-1.677474	-2.674835	-0.255335	-1.272651
H	0.356603	0.990926	-1.980128	1.502390	1.266009	-2.517193	-2.265296	-0.201098	-2.292146
O	0.955120	1.383504	-0.025547	1.482716	-0.164126	-1.002045	-1.874771	0.573908	-0.397656
C	2.277896	1.221718	-0.094997	1.197848	-1.294179	-1.656416	-1.775259	1.890199	-0.572099
C	2.832558	0.763661	1.218776	0.499856	-2.279875	-0.769379	-0.879597	2.504452	0.462834
O	2.935915	1.406863	-1.100129	1.456638	-1.487388	-2.826441	-2.329071	2.513518	-1.456069
O	-2.405950	-0.401484	-1.942076	0.811313	4.108261	-1.434600	-1.344830	-2.896041	-2.696616
C	-2.051703	-1.927964	1.286815	-1.082292	3.618314	1.447065	1.266643	-3.556459	-0.459958
C	-1.429253	-3.081762	2.062558	-2.461108	3.442460	2.077782	2.763339	-3.499956	-0.173117
C	0.092876	-3.053269	1.953940	-3.082381	2.094791	1.711259	3.249704	-2.061295	-0.005989
C	0.595907	-3.179072	0.500222	-3.212302	1.871523	0.185670	2.955785	-1.163525	-1.228989
C	0.398459	-4.629970	0.016926	-4.375079	2.728601	-0.354553	3.931470	-1.519728	-2.369335
C	-0.118311	-2.100893	-0.367357	-1.825037	2.146445	-0.461598	1.444196	-1.296614	-1.578851
H	0.276228	-1.148524	0.026907	-1.164854	1.378465	-0.024116	0.925773	-0.887379	-0.694228
C	0.335672	-2.207281	-1.823925	-1.859884	1.859829	-1.961808	1.083832	-0.394478	-2.758008
C	1.822416	-1.877232	-1.941122	-2.030807	0.360617	-2.176206	1.245820	1.071568	-2.365992
C	2.652048	-2.270569	-0.736841	-2.975899	-0.293317	-1.189686	2.375925	1.336348	-1.392924
C	4.015982	-1.965049	-0.768057	-3.257678	-1.652755	-1.362262	2.591642	2.659969	-0.992267
C	4.874343	-2.247773	0.292893	-4.116393	-2.357285	-0.520117	3.617371	3.024508	-0.120955
C	6.339151	-1.849613	0.251299	-4.369466	-3.840982	-0.716374	3.795246	4.462426	0.331822
C	6.487817	-0.332823	0.431926	-3.066551	-4.647418	-0.657693	2.601720	4.922156	1.178641
C	7.042327	-2.326621	-1.023411	-5.131136	-4.111557	-2.019207	4.038860	5.411514	-0.846451
C	4.323108	-2.868112	1.416615	-4.739287	-1.641233	0.505531	4.464016	2.009602	0.331690
C	2.965569	-3.168914	1.469556	-4.461309	-0.292165	0.698537	4.250409	0.687701	-0.041045

C	2.099329	-2.867491	0.410589	-3.552892	0.401385	-0.112174	3.193514	0.315110	-0.879818
H	-3.571107	-2.707579	-0.928411	-1.891987	4.603357	-1.819459	2.356056	-3.753087	-2.974531
H	-2.408443	-3.986933	-0.514470	-1.081980	5.597823	-0.603378	1.111023	-2.875504	-3.893347
H	-2.222655	-3.057632	-2.022734	-2.718055	4.982247	-0.291182	0.710054	-4.404004	-3.102147
H	-3.045055	3.495636	1.681909	2.432902	2.999741	1.977286	-2.090035	-3.544212	1.295358
H	-2.331583	1.914339	2.093283	3.693137	1.867971	2.581807	-3.163140	-2.844161	2.541127
H	-4.787917	1.066471	-1.428734	2.054798	-2.620084	3.586833	1.964709	-2.950369	4.000714
H	-6.165861	-0.466689	-2.816079	0.156738	-4.253677	3.513765	3.916090	-1.406467	4.300442
H	-7.321408	-2.398142	-1.759253	-2.041529	-3.543825	2.579966	3.870643	0.851052	3.254103
H	-7.107600	-2.790534	0.689243	-2.337600	-1.241071	1.705493	1.911552	1.570930	1.921535
H	-5.714194	-1.238329	2.073193	-0.430406	0.369481	1.779859	-0.056804	0.036424	1.678484
H	-1.117254	4.726356	0.981608	4.814964	0.397629	1.092400	-4.551066	-0.964019	2.051974
H	3.995813	4.576745	0.773352	3.743930	-3.821806	-1.159700	-4.074216	3.671502	1.886506
H	3.159028	4.187417	2.288560	3.623223	-3.564514	0.597155	-5.501618	4.469243	1.129910
H	3.617530	5.888938	1.953175	5.114758	-4.286889	-0.077241	-4.075246	3.992614	0.139659
H	2.261579	2.745887	-2.123294	2.979265	-0.710762	-3.377809	-3.865513	1.807327	-1.999836
H	2.311608	1.264882	2.044101	-0.512005	-1.917129	-0.533758	-0.854232	3.590805	0.327313
H	3.909211	0.958931	1.247934	1.038086	-2.375015	0.183536	0.137689	2.095519	0.368486
H	2.669945	-0.321652	1.312663	0.433556	-3.245162	-1.282361	-1.257650	2.249245	1.462752
H	-3.147833	-1.943123	1.381450	-0.655280	4.597085	1.720671	0.932607	-4.599950	-0.577008
H	-1.708562	-0.984796	1.734486	-0.406300	2.846362	1.848743	0.731956	-3.148184	0.407597
H	-1.821572	-4.049049	1.708084	-3.128620	4.266171	1.776978	3.329177	-4.010610	-0.969610
H	-1.722680	-3.004504	3.120594	-2.370562	3.512155	3.172667	2.969501	-4.058114	0.753055
H	0.516806	-3.860320	2.570791	-4.069970	2.013840	2.190462	4.330495	-2.073195	0.202573
H	0.461329	-2.102004	2.376771	-2.462049	1.289642	2.138402	2.761827	-1.622546	0.876776
H	-0.655591	-4.939027	0.021643	-4.464920	2.665653	-1.447905	3.911310	-2.585891	-2.628817
H	0.951640	-5.311351	0.681672	-4.285250	3.787596	-0.081469	4.957051	-1.277008	-2.051851
H	0.792321	-4.783679	-0.997160	-5.322048	2.358972	0.067290	3.734627	-0.942790	-3.283828
H	-0.240753	-1.527305	-2.467738	-0.934317	2.197018	-2.453502	0.049245	-0.570738	-3.090473
H	0.132075	-3.218730	-2.204919	-2.675517	2.417761	-2.445503	1.716169	-0.632796	-3.625837
H	1.952124	-0.791431	-2.087027	-1.045733	-0.123897	-2.083809	0.308060	1.429376	-1.913388
H	2.249693	-2.345113	-2.842210	-2.372895	0.146578	-3.200588	1.391391	1.696219	-3.261333
H	4.404805	-1.464476	-1.658946	-2.773686	-2.172591	-2.195093	1.922446	3.430222	-1.389126
H	6.832724	-2.340263	1.107391	-5.007183	-4.172677	0.120482	4.691290	4.489972	0.974672
H	7.546796	-0.032566	0.391981	-3.273804	-5.727546	-0.710183	1.673671	4.923166	0.583543
H	6.080390	-0.011857	1.403260	-2.512878	-4.445589	0.271842	2.757565	5.944771	1.556522
H	5.945567	0.212079	-0.358379	-2.403767	-4.395784	-1.501466	2.447421	4.257422	2.042818
H	8.120033	-2.106510	-0.974537	-6.080547	-3.556215	-2.046399	3.163940	5.450741	-1.515276
H	6.644082	-1.820692	-1.917278	-5.355229	-5.184500	-2.125723	4.904151	5.087833	-1.443821
H	6.917953	-3.410649	-1.164719	-4.536403	-3.804122	-2.894670	4.229809	6.435282	-0.488709
H	4.961937	-3.112478	2.269231	-5.438803	-2.148625	1.174623	5.294386	2.254275	0.999328
H	2.575713	-3.646033	2.369356	-4.952464	0.225999	1.523707	4.918900	-0.077282	0.355512

	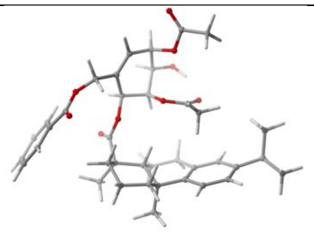 conformer **8-VII**		
C	-2.536779	-2.993313	-0.949960
C	-1.674494	-1.959148	-0.219553
C	-1.976401	-0.591142	-0.826977
O	-1.495188	0.409364	-0.048385
C	-1.304956	1.674355	-0.674159
H	-2.002848	1.748825	-1.521183
C	-1.582562	2.776689	0.324126
C	-2.666262	2.523223	1.333781
O	-3.759421	1.848813	0.700728
C	-4.277032	0.799018	1.371372
C	-5.171180	-0.036676	0.522729
C	-5.782515	-1.143819	1.121760
C	-6.571396	-1.999433	0.360899
C	-6.751653	-1.751078	-1.001162
C	-6.147671	-0.645381	-1.598091
C	-5.358929	0.214512	-0.838882
O	-4.015319	0.550069	2.524894
C	-0.876419	3.912661	0.327055
C	0.290372	4.174676	-0.587454
H	0.225520	5.192667	-0.996266
O	1.538605	4.079466	0.131317
C	1.923608	5.166691	0.826414
C	3.270587	4.938219	1.455370
O	1.256954	6.166690	0.922990
C	0.357320	3.165662	-1.737579
H	-0.521424	3.377112	-2.372695
O	1.475888	3.325129	-2.550273
C	0.126961	1.747800	-1.202888
H	0.286525	1.012085	-2.006465
O	0.932173	1.383279	-0.062977
C	2.253815	1.228219	-0.161459
C	2.835405	0.746939	1.132218
O	2.891878	1.433508	-1.175692
O	-2.483953	-0.390625	-1.903528
C	-1.984375	-1.954441	1.291932
C	-1.316203	-3.110713	2.024742
C	0.199035	-3.064752	1.847210
C	0.635999	-3.171648	0.370868
C	0.430028	-4.619802	-0.116868
C	-0.128019	-2.092905	-0.452916
H	0.277506	-1.140906	-0.069142
C	0.257702	-2.183604	-1.930319
C	1.733482	-1.835778	-2.113715
C	2.621794	-2.231636	-0.953356
C	3.978450	-1.910929	-1.042424
C	4.888509	-2.177312	-0.020957
C	6.326481	-1.707925	-0.167373
C	7.330418	-2.511906	0.658500
C	6.420844	-0.209807	0.158313
C	4.399019	-2.813125	1.122967
C	3.049026	-3.137411	1.229627
C	2.130116	-2.842005	0.214803
H	-2.299860	-3.044455	-2.020354

H	-3.599026	-2.717879	-0.860879
H	-2.409785	-3.993724	-0.516978
H	-3.028834	3.465716	1.767611
H	-2.292783	1.887084	2.148854
H	-5.618235	-1.319469	2.185592
H	-7.045420	-2.864212	0.827912
H	-7.365180	-2.425924	-1.601161
H	-6.281172	-0.456906	-2.664108
H	-4.868311	1.067983	-1.303112
H	-1.123716	4.710215	1.032011
H	3.987048	4.601811	0.693373
H	3.189374	4.141388	2.209289
H	3.616784	5.862235	1.929987
H	2.185723	2.778045	-2.170917
H	3.897360	1.009719	1.177133
H	2.747094	-0.350489	1.168943
H	2.282202	1.169576	1.979759
H	-3.074816	-1.980416	1.436810
H	-1.629497	-1.013403	1.734696
H	-1.714488	-4.078130	1.677278
H	-1.561751	-3.048439	3.095899
H	0.659171	-3.872921	2.436000
H	0.576462	-2.113504	2.262103
H	1.014859	-5.302565	0.518749
H	0.783261	-4.762019	-1.147422
H	-0.620871	-4.936577	-0.071871
H	-0.355031	-1.505259	-2.541269
H	0.047734	-3.194484	-2.309297
H	1.844201	-0.746358	-2.250303
H	2.123357	-2.287250	-3.039730
H	4.331133	-1.406517	-1.947464
H	6.593208	-1.831980	-1.231210
H	8.358139	-2.200112	0.418262
H	7.244873	-3.590972	0.460407
H	7.187226	-2.351206	1.739377
H	6.181096	-0.034939	1.220869
H	5.714779	0.376023	-0.450266
H	7.437865	0.170874	-0.026255
H	5.066885	-3.056705	1.950844
H	2.707985	-3.627017	2.142450

Table S19 Coordinates of compound **8-dias**

	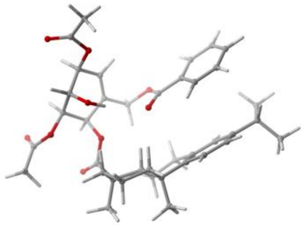 conformer **8-dias -I**			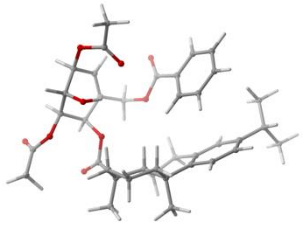 conformer **8-dias -II**			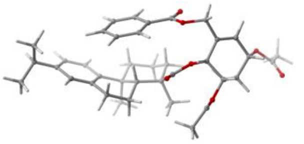 conformer **8-dias -III**		
C	-1.554120	-4.456291	-0.091632	-1.574331	-4.282464	0.081290	0.724035	-2.359722	-1.161129
C	-1.309763	-2.970011	0.184561	-1.287510	-2.792466	0.297747	0.086041	-0.997003	-0.807754
C	-2.161887	-2.168637	-0.803591	-2.201046	-2.018813	-0.655435	0.776681	-0.587034	0.501491
O	-1.944860	-0.846443	-0.645240	-2.037375	-0.685630	-0.523283	1.931734	0.076314	0.259308
C	-2.729243	0.097336	-1.380188	-2.924932	0.172219	-1.255585	2.865745	0.239571	1.351248
H	-3.067430	-0.380032	-2.313378	-3.247037	-0.367735	-2.159034	2.308204	0.181360	2.294185
C	-1.815191	1.271988	-1.647484	-2.143584	1.405167	-1.618932	3.513274	1.586369	1.163564
C	-0.580368	0.957078	-2.455625	-0.921083	1.216424	-2.482697	2.795281	2.779403	1.742434
O	0.564385	1.727461	-2.074262	0.250180	1.265090	-1.646691	1.377582	2.607554	1.752252
C	1.039918	1.517631	-0.829215	0.822894	2.475722	-1.481769	0.722567	2.912388	0.612742
C	2.253106	2.322353	-0.529855	1.857864	2.478361	-0.414677	-0.736406	2.630219	0.714171
C	2.900112	2.078217	0.685003	2.676117	3.606577	-0.303093	-1.548484	3.012683	-0.359230
C	4.022682	2.821597	1.032649	3.613068	3.686098	0.721565	-2.914504	2.754703	-0.326282
C	4.499984	3.808637	0.169997	3.724602	2.643824	1.643795	-3.476900	2.114089	0.777971
C	3.861418	4.045299	-1.048122	2.905476	1.521293	1.535476	-2.670361	1.741640	1.852966
C	2.739625	3.300801	-1.402970	1.974920	1.434365	0.505235	-1.300971	1.995736	1.825712
O	0.524637	0.757971	-0.040779	0.514885	3.450424	-2.130860	1.269471	3.365128	-0.364885
C	-2.026831	2.476959	-1.114323	-2.421259	2.600560	-1.089927	4.693402	1.702934	0.549879
C	-3.121562	2.767990	-0.128955	-3.468211	2.825447	-0.032492	5.537034	0.541975	0.106189
H	-3.998568	3.230872	-0.611143	-4.370860	3.247110	-0.501573	6.374817	0.408643	0.812748
O	-2.565985	3.702278	0.801119	-3.092603	3.857888	0.896219	6.076242	0.902607	-1.167938
C	-3.445876	4.373469	1.573473	-1.855572	3.858115	1.433326	7.143843	0.191485	-1.587555
C	-2.724327	5.247603	2.559452	-1.685067	5.024535	2.363296	7.527403	0.585890	-2.984880
O	-4.641931	4.261198	1.469693	-1.003654	3.034873	1.194487	7.688417	-0.647552	-0.914117
C	-3.559933	1.472399	0.577544	-3.866796	1.524811	0.686694	4.761879	-0.791928	0.018457
H	-4.449163	1.678770	1.186574	-4.796990	1.698165	1.248403	5.487539	-1.614921	0.026970
O	-2.581212	0.993716	1.462760	-2.924236	1.088607	1.626885	4.059542	-0.917961	-1.190617
C	-3.944911	0.506876	-0.539614	-4.160952	0.508952	-0.415065	3.890221	-0.891493	1.268892
H	-4.669023	0.999126	-1.206522	-4.916996	0.926904	-1.098067	4.527500	-0.831605	2.164628
O	-4.548881	-0.651030	0.021406	-4.681524	-0.673226	0.178052	3.216906	-2.146631	1.252660
C	-5.481021	-1.268873	-0.750247	-5.596814	-1.356495	-0.557744	2.826545	-2.627383	2.460359
C	-5.902798	-2.567145	-0.130491	-5.904720	-2.678600	0.078134	1.956075	-3.836659	2.293272
O	-5.876782	-0.819262	-1.794754	-6.062621	-0.939539	-1.586834	3.132438	-2.111354	3.504743
O	-2.927046	-2.618241	-1.622729	-2.974205	-2.511387	-1.442790	0.409833	-0.834021	1.625557
C	-1.798658	-2.581481	1.602938	-1.647007	-2.357607	1.739416	0.430032	0.039273	-1.903056
C	-0.814796	-2.997025	2.690686	-0.595160	-2.786840	2.758346	-0.443910	-0.086576	-3.141874
C	0.572522	-2.404954	2.446193	0.803419	-2.292236	2.385091	-1.915644	0.064344	-2.782957
C	1.184721	-2.845247	1.098453	1.264299	-2.781019	0.992731	-2.392505	-1.017973	-1.796595
C	1.598045	-4.329404	1.180965	1.560077	-4.295262	1.048611	-2.487950	-2.363255	-2.551430
C	0.162211	-2.499130	-0.020425	0.171644	-2.353954	-0.028325	-1.451517	-1.042649	-0.546660
H	0.107007	-1.403037	0.018648	0.142720	-1.253147	0.047198	-1.663492	-0.090821	-0.028956
C	0.715344	-2.858881	-1.398983	0.601768	-2.667221	-1.460837	-1.907735	-2.167943	0.388465
C	1.938059	-2.001623	-1.715649	1.786631	-1.784475	-1.832847	-3.281580	-1.832293	0.961769
C	2.819760	-1.703660	-0.521527	2.823279	-1.672758	-0.736513	-4.216410	-1.131372	-0.000171
C	4.010435	-1.005599	-0.760689	4.044021	-1.068115	-1.051451	-5.511123	-0.841299	0.439592
C	4.883007	-0.638515	0.257986	5.055807	-0.878893	-0.113322	-6.429317	-0.134125	-0.336847
C	6.181504	0.107642	0.002126	6.359069	-0.195889	-0.485196	-7.815736	0.200393	0.184106
C	6.194134	0.891578	-1.310217	6.121253	1.197693	-1.077802	-7.741574	1.188600	1.354787
C	7.374576	-0.855832	0.072842	7.193582	-1.068063	-1.430772	-8.605043	-1.056048	0.567163
C	4.529586	-1.001084	1.562798	4.824549	-1.347617	1.181788	-6.012927	0.278179	-1.604263
C	3.351676	-1.690993	1.816840	3.614926	-1.946617	1.513687	-4.729869	-0.008626	-2.060033

C	2.466642	-2.052237	0.790060	2.582308	-2.102428	0.579462	-3.804288	-0.704965	-1.274754
H	-2.630478	-4.675736	-0.056490	-2.655751	-4.467494	0.138578	0.412316	-2.689346	-2.160975
H	-1.056298	-5.086474	0.654928	-1.087769	-4.900231	0.843577	0.439726	-3.139764	-0.441156
H	-1.195653	-4.753391	-1.086666	-1.238481	-4.625183	-0.906988	1.820963	-2.274403	-1.157402
H	-0.719022	1.183238	-3.521175	-0.840794	1.986741	-3.259512	3.065118	3.698055	1.204130
H	-0.346368	-0.112287	-2.349003	-0.902588	0.220265	-2.941796	3.054215	2.886667	2.805564
H	2.518551	1.290756	1.335752	2.555192	4.415636	-1.024160	-1.085266	3.504337	-1.215932
H	4.530130	2.626170	1.978638	4.255440	4.564378	0.806208	-3.546795	3.037304	-1.169272
H	5.380780	4.392552	0.443936	4.456256	2.707084	2.451851	-4.544157	1.886034	0.788297
H	4.240882	4.812646	-1.724689	2.993570	0.702930	2.249807	-3.110037	1.238400	2.715664
H	2.233506	3.476627	-2.351978	1.328620	0.563136	0.419674	-0.663411	1.683950	2.651446
H	-1.326790	3.292513	-1.312684	-1.839961	3.471914	-1.399840	5.131806	2.692128	0.387335
H	-1.978602	5.867788	2.043941	-1.989484	5.955151	1.866049	7.585883	1.678940	-3.072818
H	-2.185958	4.606661	3.272729	-0.640087	5.080526	2.684964	6.742834	0.238331	-3.672947
H	-3.446689	5.874409	3.092420	-2.338119	4.883542	3.236780	8.483887	0.122861	-3.248669
H	-1.760395	0.833077	0.972500	-2.038800	1.234153	1.260627	3.326185	-0.287760	-1.173025
H	-5.156886	-3.321882	-0.423266	-5.870854	-2.609240	1.172393	2.282018	-4.444004	1.439925
H	-6.882539	-2.861981	-0.521872	-5.125163	-3.382061	-0.252330	0.934685	-3.477309	2.093199
H	-5.916605	-2.496353	0.964126	-6.880332	-3.037308	-0.267664	1.953526	-4.421243	3.219618
H	-2.786671	-3.037506	1.778742	-2.633152	-2.771303	2.006083	1.486782	-0.064532	-2.186643
H	-1.947553	-1.492956	1.652249	-1.757386	-1.264010	1.769816	0.316742	1.056110	-1.497707
H	-0.754193	-4.095711	2.760286	-0.590461	-3.883720	2.867867	-0.265753	-1.048590	-3.649738
H	-1.191717	-2.652030	3.665872	-0.870085	-2.387349	3.746768	-0.155535	0.694237	-3.862244
H	1.234959	-2.691891	3.277275	1.514568	-2.615442	3.160383	-2.519106	0.029792	-3.702640
H	0.501557	-1.303653	2.455075	0.809404	-1.187654	2.389352	-2.072705	1.056267	-2.330221
H	0.767698	-4.988981	1.465428	1.779528	-4.707411	0.053515	-1.512167	-2.724435	-2.904229
H	2.387812	-4.444781	1.939360	0.740616	-4.879074	1.484850	-3.137471	-2.236901	-3.431477
H	2.011412	-4.694824	0.230183	2.451283	-4.466103	1.670923	-2.938489	-3.153218	-1.936002
H	-0.049866	-2.706526	-2.178525	-0.225286	-2.491753	-2.167238	-1.202935	-2.289897	1.216107
H	0.975643	-3.927349	-1.444954	0.865169	-3.731559	-1.562536	-1.936775	-3.123573	-0.155311
H	1.609504	-1.032579	-2.129878	1.420783	-0.766103	-2.045758	-3.139043	-1.160144	1.825794
H	2.543277	-2.470102	-2.507943	2.267954	-2.145165	-2.755317	-3.770010	-2.737027	1.357698
H	4.239969	-0.734797	-1.794129	4.192672	-0.724488	-2.079480	-5.797702	-1.174777	1.442141
H	6.291041	0.837175	0.824200	6.933297	-0.069140	0.448500	-8.357813	0.697454	-0.638158
H	7.090634	1.528136	-1.363898	5.599315	1.135869	-2.046377	-7.212425	2.109314	1.064238
H	5.309271	1.538379	-1.403191	7.078661	1.714131	-1.250806	-7.205109	0.747079	2.210641
H	6.222286	0.218073	-2.182270	5.505757	1.815604	-0.407877	-8.749792	1.468009	1.699123
H	7.302253	-1.615053	-0.722889	8.159297	-0.588678	-1.656556	-8.668463	-1.757437	-0.278125
H	7.407612	-1.384942	1.037099	6.665976	-1.227589	-2.385342	-9.629038	-0.793577	0.875884
H	8.325718	-0.314935	-0.055049	7.393068	-2.056018	-0.988902	-8.129759	-1.585627	1.408584
H	5.184302	-0.730076	2.395928	5.597068	-1.231852	1.946421	-6.701427	0.832514	-2.247340
H	3.115367	-1.951293	2.849408	3.474281	-2.297215	2.537829	-4.449229	0.331291	-3.057191

	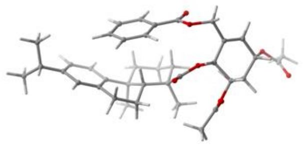 conformer **8-dias -IV**			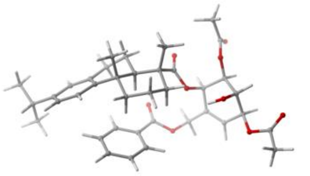 conformer **8-dias -V**			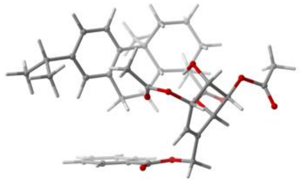 conformer **8-dias -VI**		
C	0.747219	-2.375227	-1.131418	0.425278	2.899860	1.647977	1.787150	1.164229	-3.547388
C	0.093959	-1.026018	-0.755206	0.128375	1.535876	0.993183	1.661984	1.217363	-2.019594
C	0.815878	-0.603188	0.532808	1.004099	1.536770	-0.268079	2.495371	0.057565	-1.452322
O	1.952384	0.078777	0.256572	1.783240	0.433983	-0.380202	2.041725	-0.369613	-0.252993
C	2.915475	0.257099	1.320546	2.822854	0.456360	-1.382841	2.922683	-1.151073	0.586024
H	2.387356	0.189276	2.279641	2.479527	1.083305	-2.217113	3.671165	-1.634586	-0.054586
C	3.535390	1.614359	1.115594	3.034800	-0.971332	-1.820053	2.087368	-2.154944	1.332230
C	2.815855	2.794646	1.717929	1.964997	-1.610908	-2.663894	1.850018	-3.499338	0.700586
O	1.401363	2.602799	1.762777	0.957255	-2.188641	-1.816977	0.800522	-3.454741	-0.283490
C	0.713762	2.904595	0.641846	-0.234070	-1.571410	-1.742993	-0.447561	-3.626627	0.187632
C	-0.738137	2.601064	0.777640	-1.152192	-2.265486	-0.798004	-1.470241	-3.596351	-0.895996
C	-1.582124	2.982261	-0.271285	-0.676159	-3.148594	0.177296	-2.815520	-3.650009	-0.520717
C	-2.942614	2.701858	-0.208085	-1.569855	-3.744239	1.063030	-3.810719	-3.588973	-1.490373
C	-3.467379	2.038873	0.901449	-2.935772	-3.472168	0.967743	-3.463750	-3.480252	-2.838228
C	-2.629336	1.669041	1.952948	-3.411289	-2.601209	-0.011531	-2.121370	-3.439375	-3.214715
C	-1.265321	1.946401	1.895843	-2.519688	-1.994611	-0.889214	-1.122785	-3.498694	-2.245817
O	1.229531	3.370349	-0.346508	-0.523859	-0.578829	-2.372936	-0.706679	-3.797440	1.356907
C	4.694988	1.750592	0.467708	4.106320	-1.669202	-1.429084	1.521171	-1.821797	2.495369
C	5.544049	0.603707	-0.001580	5.193559	-1.131599	-0.545108	1.768076	-0.530960	3.230935
H	6.403609	0.483194	0.680705	6.077787	-0.868384	-1.150958	2.308921	-0.796240	4.153055
O	6.041047	0.973767	-1.290049	5.549210	-2.198436	0.337594	0.572623	0.079853	3.735183
C	7.108473	0.281100	-1.739912	6.742788	-2.084765	0.958816	-0.437805	0.298606	2.862552
C	7.446337	0.682796	-3.146925	6.943494	-3.211592	1.931361	-1.477902	1.208895	3.440428
O	7.685727	-0.548988	-1.082783	7.517126	-1.185201	0.748079	-0.476680	-0.182049	1.756159
C	4.788044	-0.742276	-0.068710	4.747991	0.118276	0.238910	2.618460	0.528548	2.473691
H	5.526490	-1.553709	-0.082586	5.640158	0.607356	0.650346	3.189136	1.104371	3.216197
O	4.052688	-0.878200	-1.256887	3.939238	-0.201698	1.340468	1.837717	1.479555	1.802569
C	3.955348	-0.856921	1.206767	4.100951	1.051034	-0.778957	3.613371	-0.200218	1.564222
H	4.618071	-0.786591	2.083036	4.804368	1.218169	-1.609253	4.304127	-0.801061	2.175527
O	3.302942	-2.123130	1.209122	3.822401	2.296722	-0.152554	4.348407	0.785852	0.846028
C	2.956981	-2.611707	2.427143	3.848411	3.384811	-0.968233	5.544300	0.386724	0.343526
C	2.106512	-3.838260	2.284168	3.372016	4.605926	-0.242471	6.088400	1.410750	-0.605696
O	3.282619	-2.090195	3.462788	4.195075	3.333985	-2.120095	6.052868	-0.670270	0.618515
O	0.485399	-0.854169	1.667221	1.049910	2.436060	-1.072231	3.499237	-0.379426	-1.962545
C	0.383251	0.018337	-1.858726	0.535254	0.377578	1.926475	2.391594	2.470170	-1.457479
C	-0.528345	-0.121052	-3.068603	-0.433357	0.176113	3.084833	1.583185	3.748492	-1.638238
C	-1.989741	0.003662	-2.660767	-1.852064	-0.070838	2.578645	0.206218	3.626693	-0.991109
C	-2.415279	-1.089630	-1.663357	-2.382475	1.083224	1.699816	-0.635755	2.476491	-1.583386
C	-2.513440	-2.433770	-2.419856	-2.670794	2.311247	2.590834	-1.099754	2.856239	-3.005001
C	-1.433255	-1.102942	-0.445315	-1.356951	1.331269	0.547860	0.201639	1.165466	-1.490364
H	-1.646422	-0.158236	0.084263	-1.333876	0.374901	-0.003133	0.279524	1.002622	-0.406568
C	-1.836750	-2.241650	0.497711	-1.910114	2.365596	-0.434380	-0.587562	-0.029605	-2.019338
C	-3.199612	-1.939236	1.114884	-3.083861	1.741904	-1.178459	-1.808443	-0.278729	-1.140958
C	-4.175183	-1.245212	0.189392	-4.045535	1.005424	-0.272763	-2.470205	0.969995	-0.608283
C	-5.462160	-0.979284	0.673338	-5.279206	0.604501	-0.798030	-3.657573	0.813607	0.113759
C	-6.414608	-0.280657	-0.063322	-6.233657	-0.078916	-0.045331	-4.331444	1.885377	0.697743
C	-7.789136	0.014227	0.509830	-7.555658	-0.516417	-0.650577	-5.623138	1.681184	1.470660
C	-8.907750	-0.573031	-0.357846	-7.344206	-1.554691	-1.759182	-5.391303	0.871103	2.751796
C	-7.987744	1.519167	0.727884	-8.374194	0.676506	-1.156801	-6.709423	1.039017	0.600338
C	-6.045394	0.153555	-1.341677	-5.923332	-0.348859	1.290074	-3.770054	3.155564	0.545744
C	-4.775943	-0.110609	-1.839941	-4.696367	0.029057	1.822298	-2.588483	3.328382	-0.167813
C	-3.813252	-0.802295	-1.091961	-3.726019	0.690181	1.058519	-1.913346	2.251242	-0.755592
H	1.841702	-2.267810	-1.164713	0.019403	2.958133	2.663731	1.229479	0.316774	-3.971092
H	0.408057	-2.710532	-2.120459	0.006248	3.725697	1.057378	2.841301	1.041155	-3.827875

H	0.503604	-3.161221	-0.403083	1.513895	3.047435	1.718729	1.411492	2.083070	-4.012688
H	3.059079	3.719367	1.177402	2.376982	-2.448338	-3.240749	1.593906	-4.247751	1.460940
H	3.100231	2.900722	2.774673	1.491968	-0.887953	-3.340809	2.733179	-3.829967	0.139207
H	-1.147624	3.489821	-1.133732	0.392287	-3.354475	0.239645	-3.058462	-3.726930	0.539518
H	-3.599774	2.983515	-1.032212	-1.199441	-4.423274	1.832862	-4.861228	-3.623313	-1.196893
H	-4.529879	1.790811	0.932524	-3.633567	-3.936583	1.666987	-4.244507	-3.428288	-3.599403
H	-3.039532	1.148020	2.819583	-4.474397	-2.367332	-0.077774	-1.850926	-3.358165	-4.268702
H	-0.602719	1.634869	2.701794	-2.875880	-1.300379	-1.649930	-0.071166	-3.463126	-2.528895
H	5.112403	2.746905	0.293859	4.233561	-2.703998	-1.760245	0.863323	-2.536619	2.996671
H	8.402316	0.235347	-3.438010	6.226091	-3.095617	2.756858	-2.330443	1.258905	2.753518
H	7.485024	1.776736	-3.234886	7.966364	-3.183060	2.320799	-1.033052	2.208686	3.550289
H	6.648439	0.323537	-3.813302	6.739265	-4.175802	1.446728	-1.787565	0.864949	4.436614
H	3.309846	-0.260293	-1.216525	3.069447	-0.467611	1.013907	1.389878	1.016218	1.080154
H	1.072548	-3.500377	2.113765	2.271866	4.577797	-0.249909	5.848989	2.427926	-0.271819
H	2.142442	-4.422831	3.209841	3.714484	5.503567	-0.768509	5.595558	1.239046	-1.575501
H	2.420244	-4.438832	1.421497	3.712781	4.603664	0.800528	7.168873	1.272886	-0.721376
H	1.431988	-0.065747	-2.176544	1.558788	0.547735	2.295997	3.374783	2.555751	-1.946638
H	0.264603	1.032244	-1.447619	0.551566	-0.554771	1.340370	2.582280	2.325474	-0.382408
H	-0.350178	-1.079116	-3.583871	-0.410225	1.039689	3.769237	1.485333	3.997643	-2.707516
H	-0.277522	0.665781	-3.796409	-0.102788	-0.688204	3.681596	2.130864	4.586487	-1.179810
H	-2.622705	-0.038498	-3.560114	-2.517708	-0.232902	3.439847	-0.326448	4.583497	-1.099939
H	-2.148141	0.991403	-2.199550	-1.867093	-1.000270	1.985032	0.334146	3.450868	0.091233
H	-3.192473	-2.315092	-3.278470	-1.813964	2.604098	3.209472	-0.267698	3.104809	-3.676938
H	-2.931694	-3.232753	-1.793393	-3.500836	2.074209	3.273418	-1.755551	3.738621	-2.947620
H	-1.543655	-2.778224	-2.804701	-2.979702	3.185860	2.001919	-1.684443	2.051206	-3.471846
H	-1.104246	-2.351808	1.302725	-1.145541	2.675179	-1.154273	0.042811	-0.931856	-2.011311
H	-1.861514	-3.195286	-0.049764	-2.229583	3.275296	0.097964	-0.884494	0.133733	-3.067057
H	-3.045320	-1.275124	1.983134	-2.678572	1.030848	-1.919674	-1.492868	-0.855298	-0.258951
H	-3.658960	-2.857854	1.513785	-3.634549	2.505562	-1.749765	-2.550224	-0.893937	-1.672570
H	-5.719856	-1.326236	1.679603	-5.492414	0.849475	-1.843306	-4.057572	-0.199900	0.217928
H	-7.839808	-0.475914	1.496875	-8.134073	-1.000499	0.154619	-5.982772	2.680751	1.768683
H	-8.772137	-1.655559	-0.500157	-6.771322	-1.127049	-2.597995	-6.330832	0.746418	3.313224
H	-8.928504	-0.102894	-1.354323	-8.309230	-1.906138	-2.157061	-4.664359	1.369641	3.410718
H	-9.891195	-0.406462	0.108974	-6.789342	-2.429363	-1.386147	-5.001455	-0.133505	2.519703
H	-7.208021	1.932236	1.386303	-7.858759	1.190693	-1.984019	-7.659899	0.966538	1.152177
H	-8.967287	1.723467	1.188191	-8.539570	1.412170	-0.355554	-6.422990	0.019919	0.293859
H	-7.945450	2.066624	-0.227798	-9.355974	0.345552	-1.530292	-6.883997	1.627084	-0.313082
H	-6.755799	0.705506	-1.961852	-6.648822	-0.868341	1.921588	-4.265083	4.024477	0.987194
H	-4.531364	0.243295	-2.841766	-4.492604	-0.207683	2.867649	-2.190057	4.338720	-0.268945

	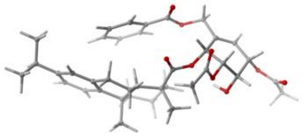 conformer **8-dias -VI**			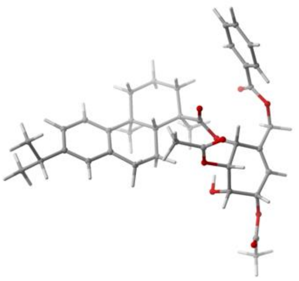 conformer **8-dias -VII**		
C	0.753353	-2.601485	0.496353	0.246038	0.431910	-2.708707
C	0.094191	-1.348444	-0.120723	-0.279277	1.040004	-1.401537
C	0.793023	-0.161431	0.553348	0.727572	0.824808	-0.268584
O	1.985896	0.088420	-0.030966	1.641481	-0.136263	-0.543592
C	2.879151	1.027241	0.609510	2.652668	-0.407927	0.452566
H	2.293022	1.666790	1.282560	2.755798	0.482948	1.084222
C	3.522529	1.824774	-0.496982	3.915717	-0.684228	-0.318626
C	2.754928	3.003251	-1.035314	4.520275	0.485865	-1.055363
O	1.380785	2.675060	-1.273369	4.336256	1.690993	-0.309032
C	0.485940	3.090286	-0.345854	3.467670	2.600764	-0.801998
C	-0.903622	2.681427	-0.688125	3.196428	3.697203	0.163272
C	-1.253469	2.203177	-1.954414	3.658034	3.652348	1.482187
C	-2.570006	1.826718	-2.212095	3.339591	4.683601	2.360640
C	-3.532321	1.907558	-1.206573	2.567440	5.759799	1.924324
C	-3.182978	2.392251	0.054365	2.109897	5.807090	0.606905
C	-1.874045	2.783576	0.313949	2.421170	4.776030	-0.272454
O	0.791682	3.705300	0.647311	2.962516	2.516607	-1.899187
C	4.738405	1.508706	-0.951971	4.407739	-1.921047	-0.427886
C	5.611539	0.437147	-0.363936	3.767004	-3.139247	0.176603
H	6.392235	0.901405	0.263322	4.318449	-3.450048	1.080539
O	6.247949	-0.210676	-1.469129	3.885777	-4.174362	-0.803388
C	7.350149	-0.935275	-1.183003	3.730738	-5.439393	-0.358181
C	7.847168	-1.650048	-2.406998	3.775026	-6.412544	-1.501455
O	7.841244	-0.991037	-0.083257	3.570363	-5.719481	0.803516
C	4.836781	-0.581009	0.500759	2.289427	-2.910855	0.563611
H	5.558071	-1.124370	1.124263	1.976741	-3.724506	1.230264
O	4.193037	-1.556523	-0.278791	1.427811	-2.966275	-0.543851
C	3.904901	0.220717	1.406227	2.250938	-1.594517	1.327343
H	4.496439	0.926805	2.008817	2.975601	-1.641628	2.154936
O	3.221428	-0.681993	2.269797	0.950511	-1.387851	1.864461
C	2.796330	-0.167389	3.452694	0.893501	-0.591724	2.965713
C	1.912695	-1.146579	4.166357	-0.527283	-0.294693	3.335553
O	3.086799	0.936824	3.833393	1.875548	-0.188827	3.534633
O	0.392099	0.484035	1.491429	0.722831	1.427381	0.777664
C	0.392219	-1.276331	-1.637022	-0.450882	2.569331	-1.539711
C	-0.496847	-2.191856	-2.464563	-1.694605	2.952294	-2.332073
C	-1.966980	-1.860440	-2.246490	-2.959747	2.351379	-1.721712
C	-2.397599	-2.030096	-0.776885	-2.910444	0.810361	-1.625735
C	-2.466382	-3.539548	-0.454719	-3.045783	0.206368	-3.040540
C	-1.435147	-1.212782	0.145825	-1.608974	0.415634	-0.866672
H	-1.655447	-0.157514	-0.095596	-1.730195	0.873850	0.132878
C	-1.851235	-1.425001	1.604820	-1.539148	-1.096305	-0.650993
C	-3.211241	-0.775627	1.840647	-2.661038	-1.532467	0.283120
C	-4.179702	-0.906618	0.685047	-3.984667	-0.846308	0.024564
C	-5.467433	-0.388543	0.848761	-5.119145	-1.330957	0.689704
C	-6.420333	-0.401027	-0.169881	-6.377899	-0.755585	0.550776
C	-7.798303	0.204125	0.029686	-7.582111	-1.312629	1.288558
C	-7.711084	1.716035	0.271756	-8.654521	-1.817754	0.316410
C	-8.570687	-0.494617	1.154112	-8.162066	-0.289061	2.271630
C	-6.049825	-0.977222	-1.386743	-6.487280	0.353398	-0.296598
C	-4.771842	-1.497650	-1.566646	-5.374039	0.845743	-0.962584

C	-3.808516	-1.462087	-0.552004	-4.102829	0.269714	-0.817275
H	0.482473	-2.719926	1.554990	0.237730	-0.666709	-2.692616
H	1.849180	-2.525595	0.436457	1.275788	0.770252	-2.887574
H	0.446134	-3.511202	-0.036122	-0.360070	0.756573	-3.562238
H	3.167434	3.327704	-1.999173	4.040921	0.607541	-2.036775
H	2.793618	3.835413	-0.317326	5.600759	0.350126	-1.193686
H	-0.492692	2.132723	-2.732009	4.254608	2.804889	1.817482
H	-2.846495	1.457218	-3.201046	3.690832	4.644137	3.392716
H	-4.553912	1.572570	-1.396516	2.317852	6.566172	2.616555
H	-3.938938	2.450243	0.839374	1.505458	6.649400	0.266833
H	-1.574276	3.153522	1.294499	2.069328	4.789865	-1.304246
H	5.182003	2.076658	-1.775484	5.327460	-2.101457	-0.992297
H	7.119286	-2.429025	-2.677360	2.881765	-6.262730	-2.125340
H	8.819381	-2.107502	-2.196402	3.789633	-7.435982	-1.112719
H	7.919866	-0.954675	-3.253929	4.655040	-6.223495	-2.130803
H	3.428525	-1.140810	-0.699837	1.530266	-2.151204	-1.053380
H	0.905152	-1.055785	3.731982	-1.138528	-1.205517	3.292350
H	1.866151	-0.894504	5.231272	-0.920031	0.423753	2.600410
H	2.262029	-2.175642	4.016003	-0.560844	0.150827	4.335175
H	1.449681	-1.516554	-1.816403	0.457386	2.984622	-2.002486
H	0.245616	-0.237925	-1.977015	-0.522252	2.997602	-0.528244
H	-0.297857	-3.248593	-2.221995	-1.594561	2.641761	-3.385113
H	-0.243549	-2.073404	-3.529541	-1.785408	4.049626	-2.351804
H	-2.582945	-2.498566	-2.897889	-3.829417	2.667975	-2.317150
H	-2.147145	-0.817347	-2.552059	-3.102628	2.762549	-0.707267
H	-1.488424	-4.035364	-0.524297	-2.959663	-0.889109	-3.029430
H	-3.143068	-4.030404	-1.171344	-2.304886	0.598447	-3.748197
H	-2.872436	-3.731556	0.547273	-4.043179	0.445857	-3.439557
H	-1.123430	-0.971051	2.284324	-0.579271	-1.402889	-0.215001
H	-1.887110	-2.499208	1.838692	-1.618961	-1.624470	-1.613614
H	-3.051188	0.302459	2.015629	-2.362108	-1.304905	1.320560
H	-3.678282	-1.168616	2.757767	-2.794816	-2.624552	0.241588
H	-5.721458	0.053462	1.817596	-5.003293	-2.201064	1.344260
H	-8.358147	0.047811	-0.907954	-7.229635	-2.177522	1.875825
H	-8.715924	2.159098	0.355111	-8.242315	-2.570020	-0.372725
H	-7.180940	2.221064	-0.550318	-9.060478	-0.992517	-0.290969
H	-7.170344	1.936673	1.206641	-9.494462	-2.273879	0.863796
H	-8.070464	-0.356142	2.126274	-7.397312	0.053419	2.984830
H	-8.649407	-1.576209	0.968301	-8.996822	-0.725144	2.842782
H	-9.588805	-0.084013	1.241749	-8.546888	0.596973	1.741007
H	-6.768298	-1.016148	-2.209604	-7.454049	0.841005	-0.443089
H	-4.524910	-1.934561	-2.534662	-5.504904	1.705397	-1.621274
